# Supplementary material for: Differential Alternative Splicing Genes and Isoform Regulation Networks of Rapeseed (Brassica napus L.) Infected with Sclerotinia sclerotiorum
Source: Genes (Basel). 2020 Jul 13;11(7):784. doi: 10.3390/genes11070784 (PMC7397149; doi:10.3390/genes11070784)
Supplement: Supplementary file 1 [file genes-11-00784-s001.zip › Fig. S1 Enriched GO terms of 79 DAS genes.zip!/Fig. S1/go.pdf]

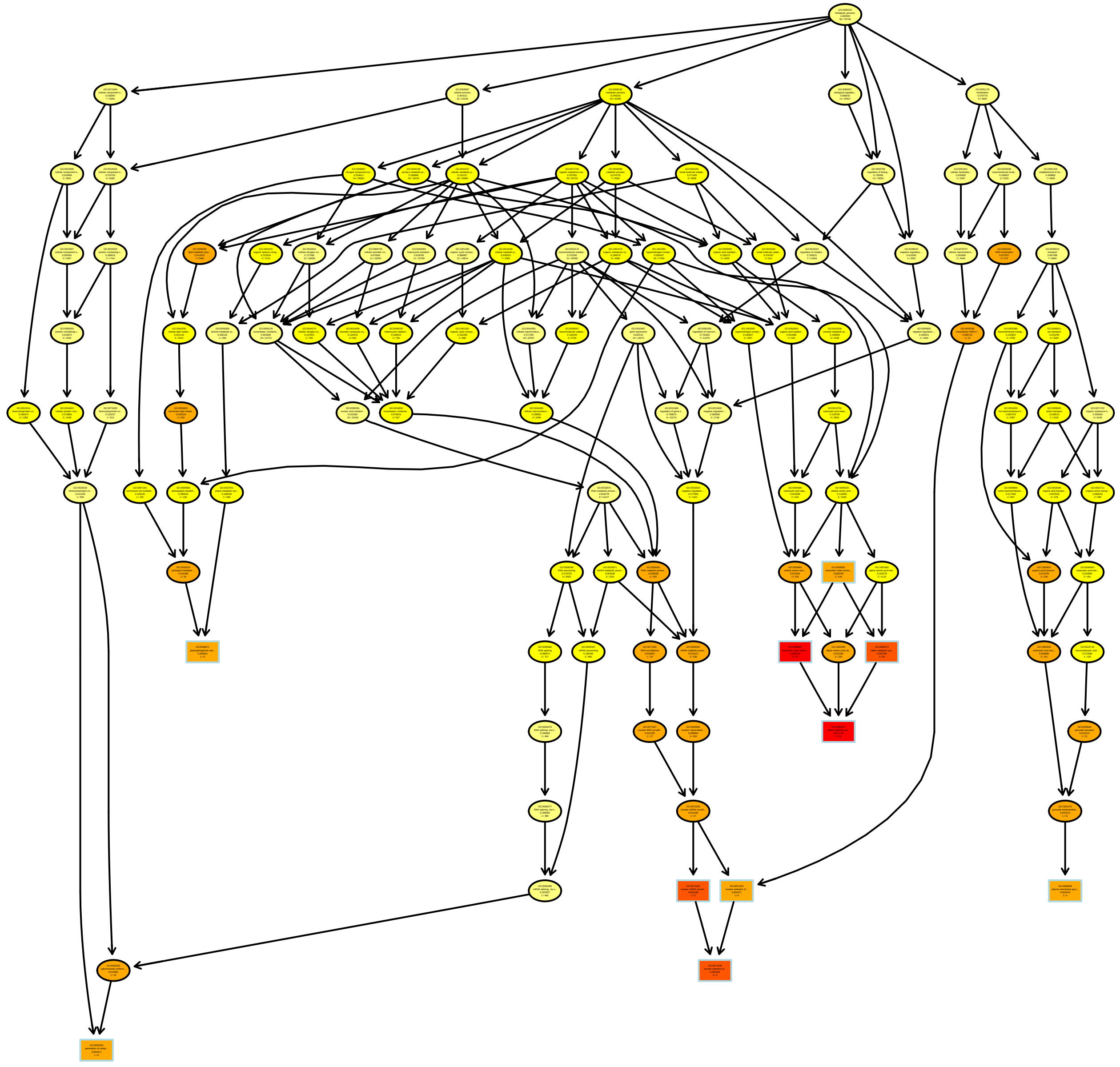

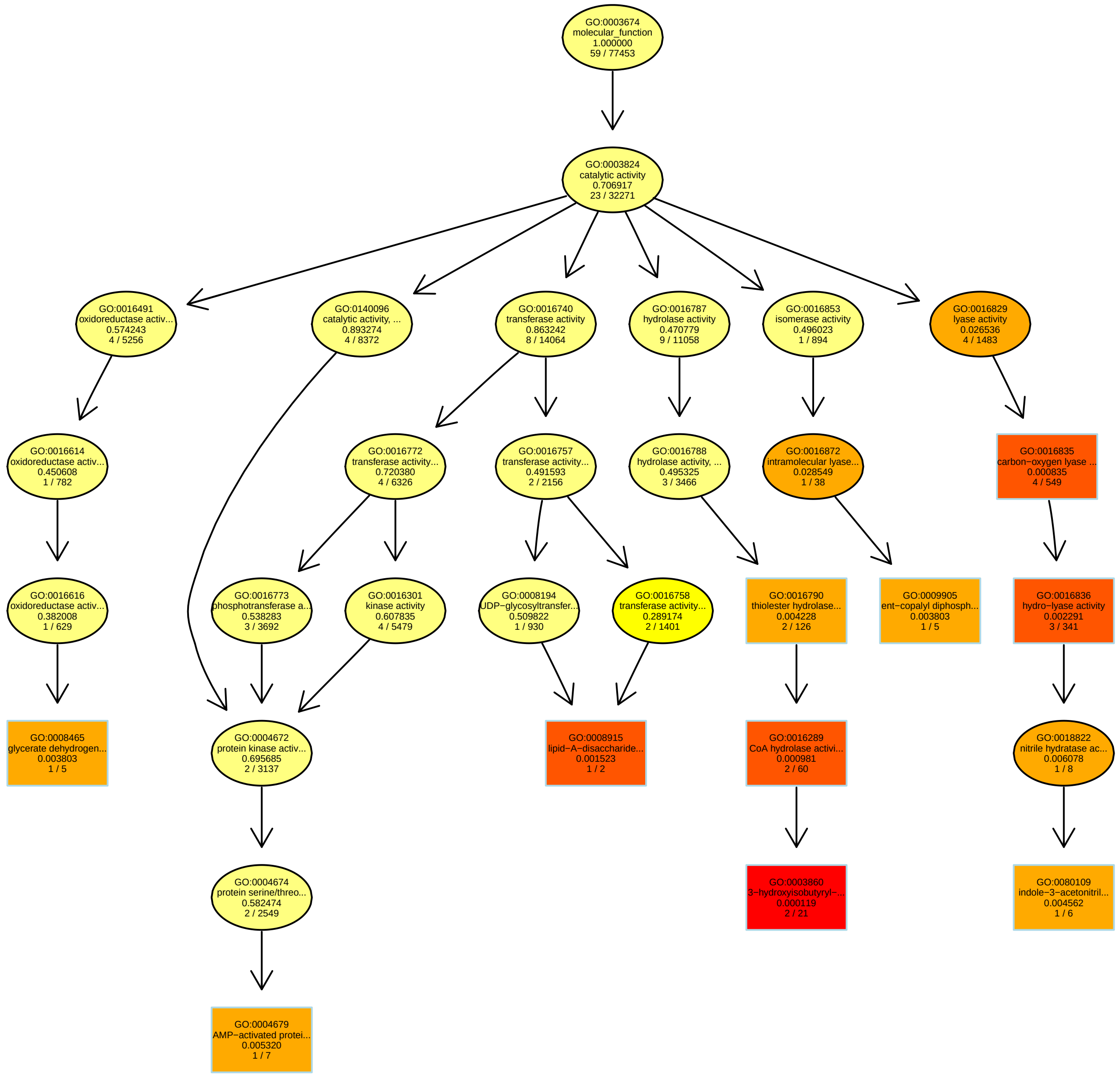

GO:0003674
molecular_function
1.000000
59 / 77453
GO:0003824
catalytic activity
0.706917
23 / 32271
GO:0016491
oxidoreductase activ...
0.574243
4 / 5256
GO:0140096
catalytic activity, ...
0.893274
4 / 8372
GO:0016740
transferase activity
0.863242
8 / 14064
GO:0016787
hydrolase activity
0.470779
9 / 11058
GO:0016853
isomerase activity
0.496023
1 / 894
GO:0016829
lyase activity
0.026536
4 / 1483
GO:0016614
oxidoreductase activ...
0.450608
1 / 782
GO:0016772
transferase activity...
0.720380
4 / 6326
GO:0016757
transferase activity...
0.491593
2 / 2156
GO:0016788
hydrolase activity, ...
0.495325
3 / 3466
GO:0016872
intramolecular lyase...
0.028549
1 / 38
GO:0016835
carbon−oxygen lyase ...
0.000835
4 / 549
GO:0016616
oxidoreductase activ...
0.382008
1 / 629
GO:0016773
phosphotransferase a...
0.538283
3 / 3692
GO:0016301
kinase activity
0.607835
4 / 5479
GO:0008194
UDP−glycosyltransfer...
0.509822
1 / 930
GO:0016758
transferase activity...
0.289174
2 / 1401
GO:0016790
thiolester hydrolase...
0.004228
2 / 126
GO:0009905
ent−copalyl diphosph...
0.003803
1 / 5
GO:0016836
hydro−lyase activity
0.002291
3 / 341
GO:0008465
glycerate dehydrogen...
0.003803
1 / 5
GO:0004672
protein kinase activ...
0.695685
2 / 3137
GO:0008915
lipid−A−disaccharide...
0.001523
1 / 2
GO:0016289
CoA hydrolase activi...
0.000981
2 / 60
GO:0018822
nitrile hydratase ac...
0.006078
1 / 8
GO:0004674
protein serine/threo...
0.582474
2 / 2549
GO:0003860
3−hydroxyisobutyryl−...
0.000119
2 / 21
GO:0080109
indole−3−acetonitril...
0.004562
1 / 6
GO:0004679
AMP−activated protei...
0.005320
1 / 7

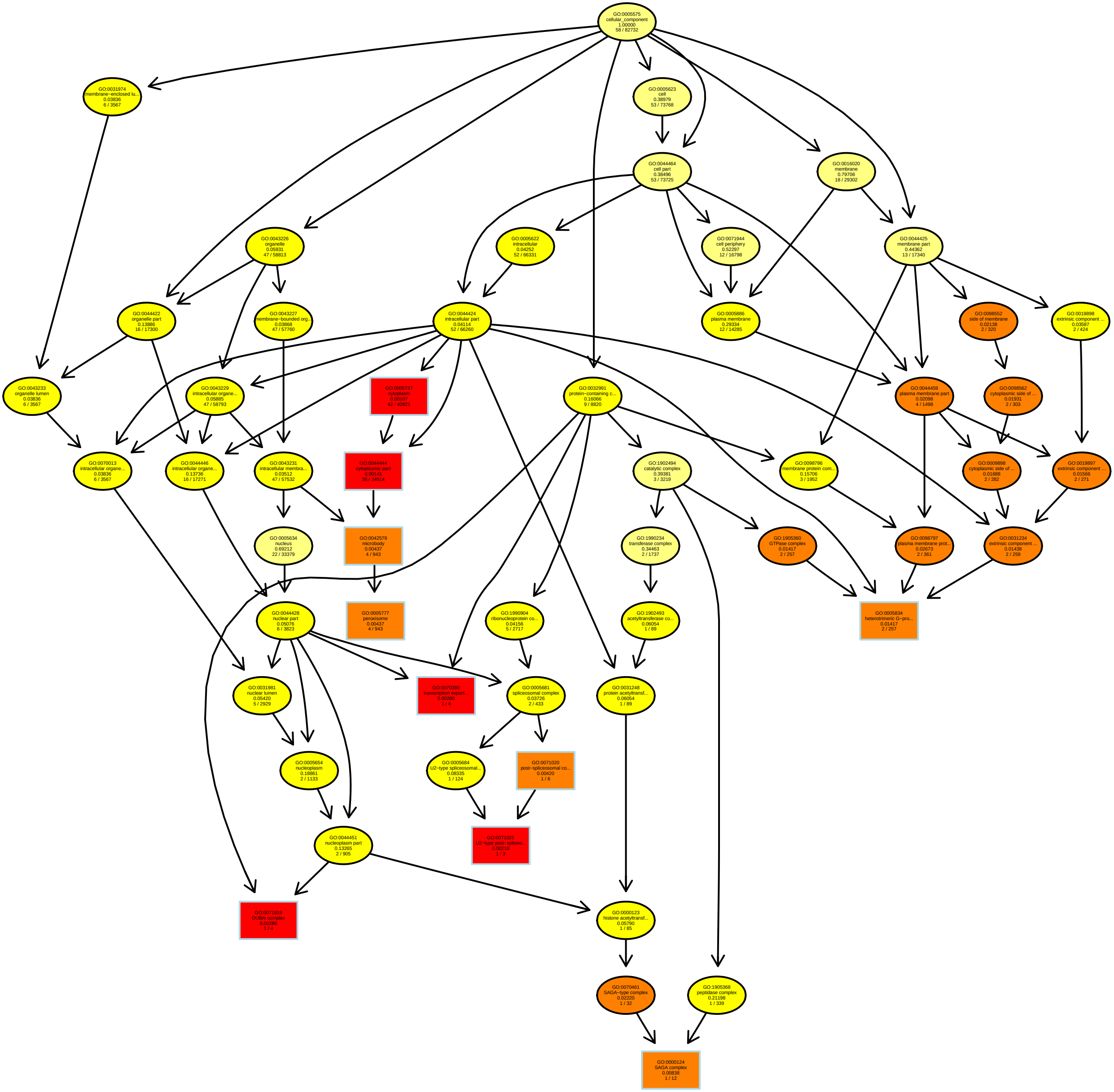
GO:0005575 cellular_component 1.00000 58 / 82732
GO:0031974 membrane−enclosed lu... 0.03836 6 / 3567
GO:0005623 cell 0.38979 53 / 73768
GO:0044464 cell part 0.38496 53 / 73725
GO:0016020 membrane 0.79706 18 / 29302
GO:0043226 organelle 0.05931 47 / 58813
GO:0005622 intracellular 0.04252 52 / 66331
GO:0071944 cell periphery 0.52297 12 / 16798
GO:0044425 membrane part 0.44362 13 / 17340
GO:0044422 organelle part 0.13886 16 / 17300
GO:0043227 membrane−bounded org... 0.03868 47 / 57760
GO:0044424 intracellular part 0.04114 52 / 66260
GO:0005886 plasma membrane 0.29334 12 / 14285
GO:0098552 side of membrane 0.02138 2 / 320
GO:0019898 extrinsic component ... 0.03587 2 / 424
GO:0043233 organelle lumen 0.03836 6 / 3567
GO:0043229 intracellular organe... 0.05885 47 / 58793
GO:0005737 cytoplasm 0.00107 42 / 42821
GO:0032991 protein−containing c... 0.16066 9 / 8820
GO:0044459 plasma membrane part 0.02098 4 / 1498
GO:0098562 cytoplasmic side of ... 0.01931 2 / 303
GO:0070013 intracellular organe... 0.03836 6 / 3567
GO:0044446 intracellular organe... 0.13736 16 / 17271
GO:0043231 intracellular membra... 0.03512 47 / 57532
GO:0044444 cytoplasmic part 0.00141 36 / 34514
GO:1902494 catalytic complex 0.39381 3 / 3219
GO:0098796 membrane protein com... 0.15706 3 / 1952
GO:0009898 cytoplasmic side of ... 0.01688 2 / 282
GO:0019897 extrinsic component ... 0.01566 2 / 271
GO:0005634 nucleus 0.69212 22 / 33379
GO:0042579 microbody 0.00437 4 / 943
GO:1990234 transferase complex 0.34463 2 / 1737
GO:1905360 GTPase complex 0.01417 2 / 257
GO:0098797 plasma membrane prot... 0.02673 2 / 361
GO:0031234 extrinsic component ... 0.01438 2 / 259
GO:0044428 nuclear part 0.05076 6 / 3823
GO:0005777 peroxisome 0.00437 4 / 943
GO:1990904 ribonucleoprotein co... 0.04156 5 / 2717
GO:1902493 acetyltransferase co... 0.06054 1 / 89
GO:0005834 heterotrimeric G−pro... 0.01417 2 / 257
GO:0031981 nuclear lumen 0.05420 5 / 2929
GO:0070390 transcription export... 0.00280 1 / 4
GO:0005681 spliceosomal complex 0.03726 2 / 433
GO:0031248 protein acetyltransf... 0.06054 1 / 89
GO:0005654 nucleoplasm 0.18861 2 / 1133
GO:0005684 U2−type spliceosomal... 0.08335 1 / 124
GO:0071020 post−spliceosomal co... 0.00420 1 / 6
GO:0044451 nucleoplasm part 0.13265 2 / 905
GO:0071021 U2−type post−spliceo... 0.00210 1 / 3
GO:0071819 DUBm complex 0.00280 1 / 4
GO:0000123 histone acetyltransf... 0.05790 1 / 85
GO:0070461 SAGA−type complex 0.02220 1 / 32
GO:1905368 peptidase complex 0.21198 1 / 339
GO:0000124 SAGA complex 0.00838 1 / 12
