# Supplementary material for: Differential Alternative Splicing Genes and Isoform Regulation Networks of Rapeseed (Brassica napus L.) Infected with Sclerotinia sclerotiorum
Source: Genes (Basel). 2020 Jul 13;11(7):784. doi: 10.3390/genes11070784 (PMC7397149; doi:10.3390/genes11070784)
Supplement: Supplementary file 1 [file genes-11-00784-s001.zip › Fig. S1 Enriched GO terms of 79 DAS genes.zip!/Fig. S1/pvalFis.pdf]

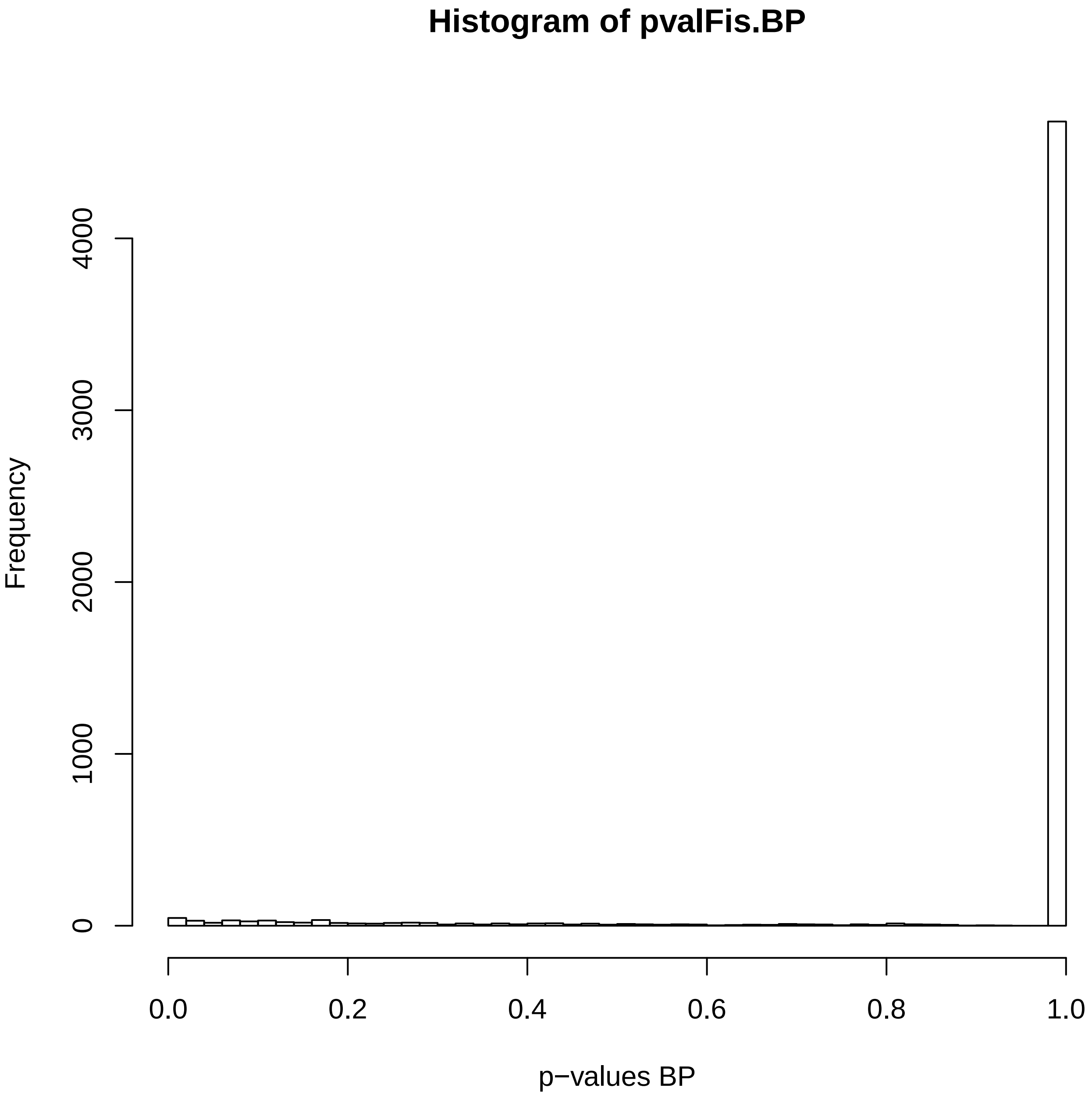
Histogram of pvalFis.BP
Frequency
0
1000
2000
3000
4000
0.0
0.2
0.4
0.6
0.8
1.0
p−values BP

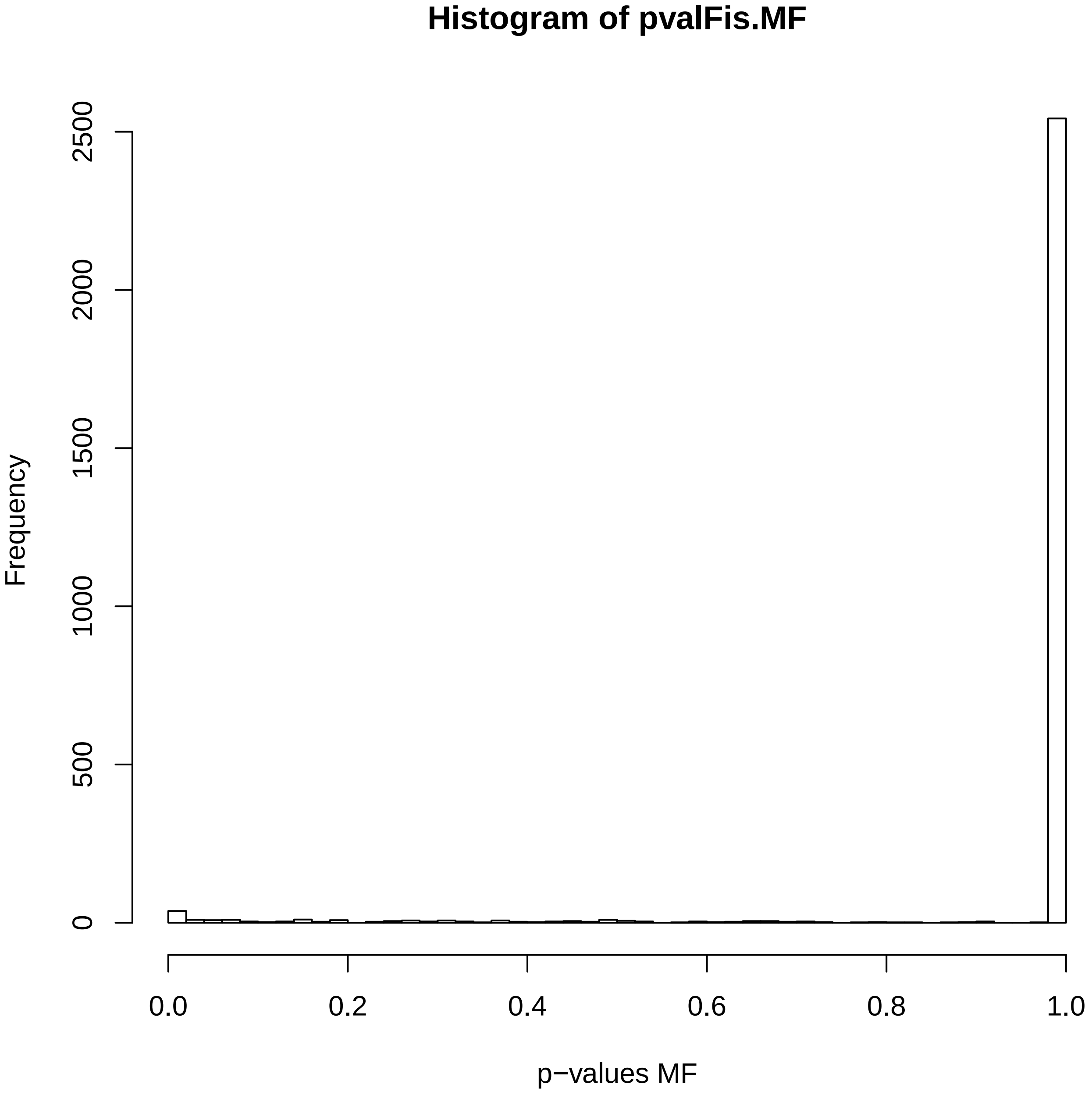

Histogram of pvalFis.MF
Frequency
0
500
1000
1500
2000
2500
0.0
0.2
0.4
0.6
0.8
1.0
p−values MF

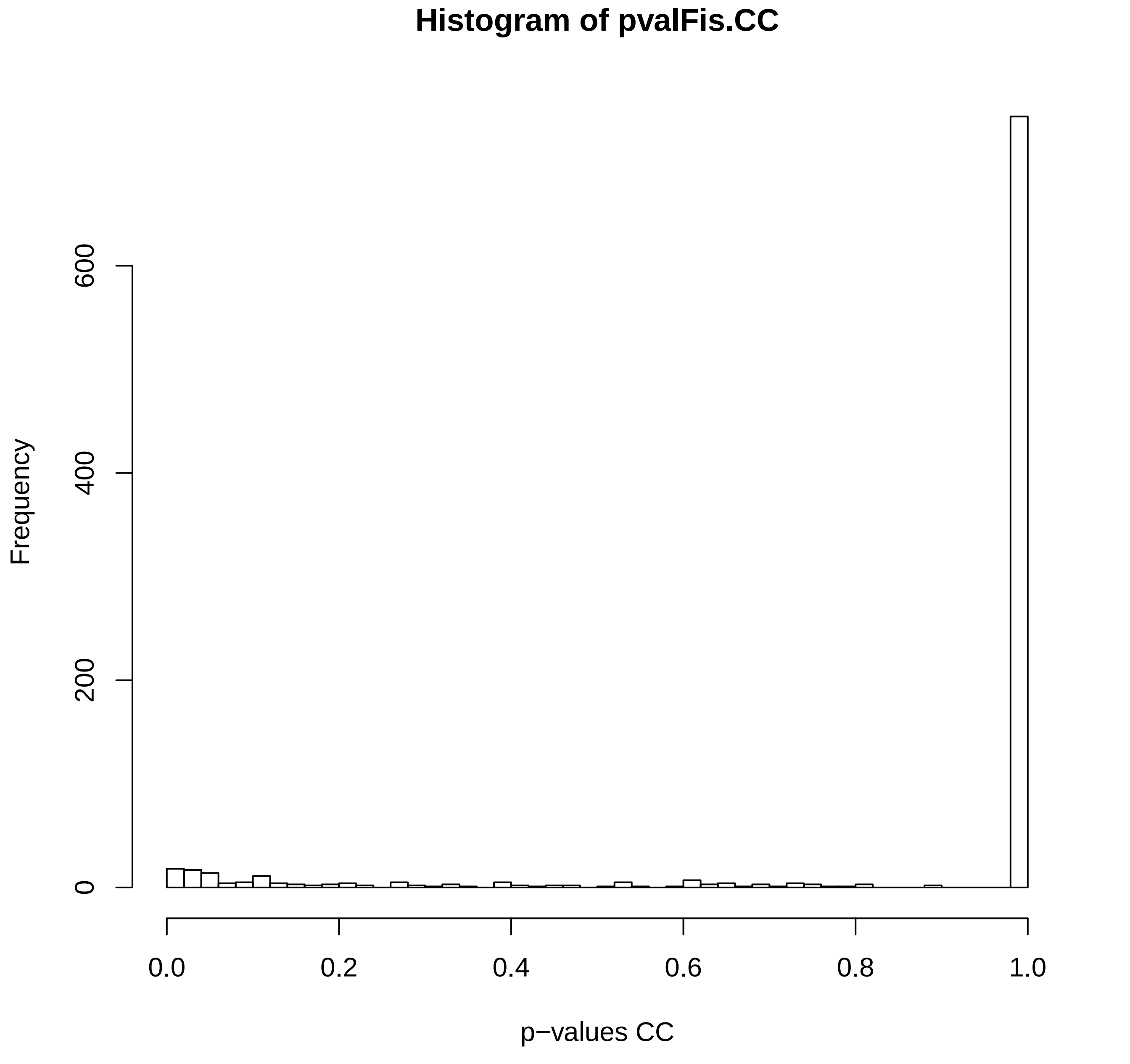
Histogram of pvalFis.CC
Frequency
0
200
400
600
0.0
0.2
0.4
0.6
0.8
1.0
p−values CC
