# Supplementary material for: Differential Alternative Splicing Genes and Isoform Regulation Networks of Rapeseed (Brassica napus L.) Infected with Sclerotinia sclerotiorum
Source: Genes (Basel). 2020 Jul 13;11(7):784. doi: 10.3390/genes11070784 (PMC7397149; doi:10.3390/genes11070784)
Supplement: Supplementary file 1 [file genes-11-00784-s001.zip › Fig. S2 Enriched GO terms of genes in the 85 WGCNA modules.zip!/Fig. S2/CytoNodeantiquewhite4-go.pdf]

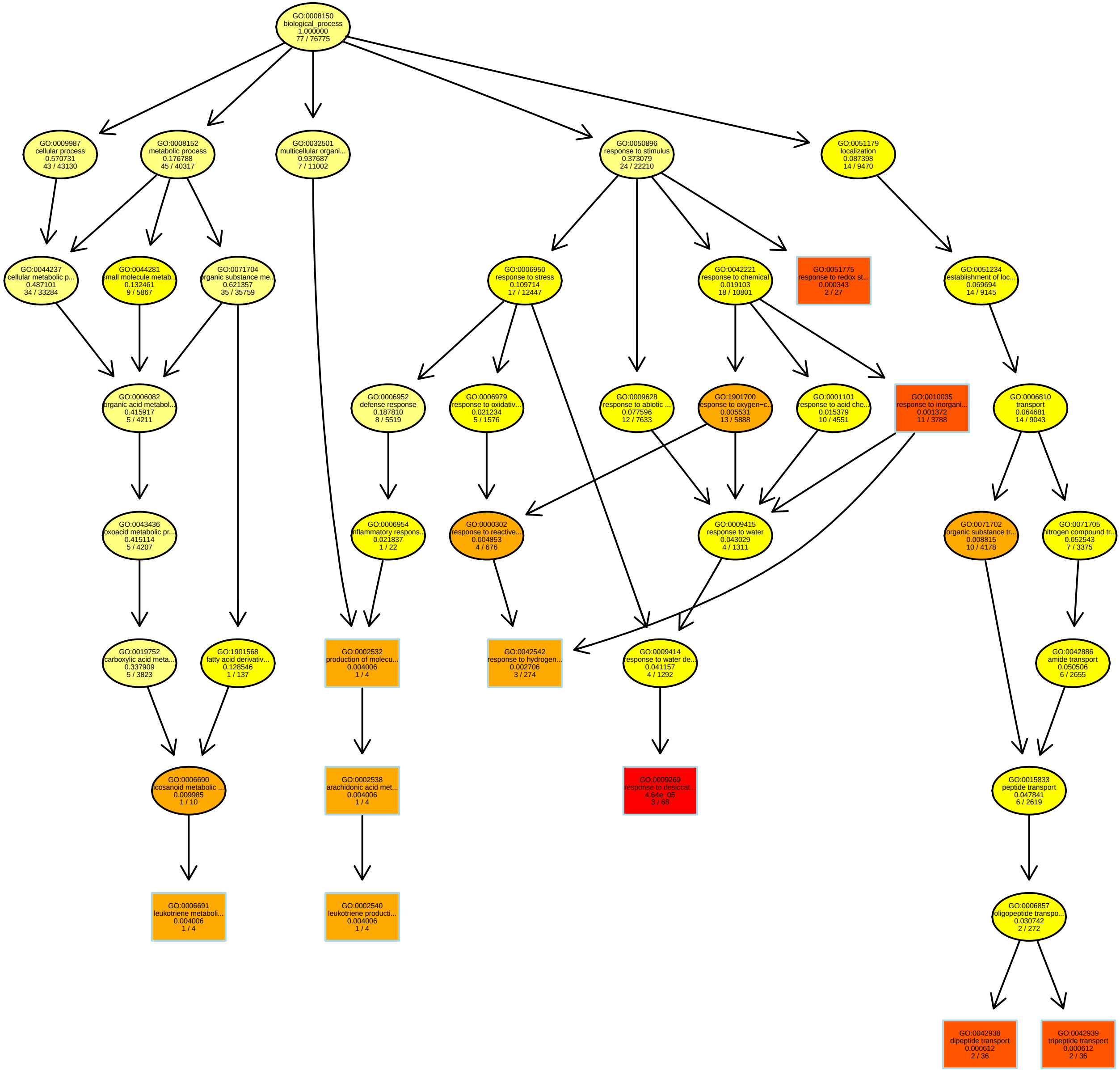
GO:0008150 biological_process 1.000000 77 / 76775
GO:0009987 cellular process 0.570731 43 / 43130
GO:0008152 metabolic process 0.176788 45 / 40317
GO:0032501 multicellular organi... 0.937687 7 / 11002
GO:0050896 response to stimulus 0.373079 24 / 22210
GO:0051179 localization 0.087398 14 / 9470
GO:0044237 cellular metabolic p... 0.487101 34 / 33284
GO:0044281 small molecule metab... 0.132461 9 / 5867
GO:0071704 organic substance me... 0.621357 35 / 35759
GO:0006950 response to stress 0.109714 17 / 12447
GO:0042221 response to chemical 0.019103 18 / 10801
GO:0051775 response to redox st... 0.000343 2 / 27
GO:0051234 establishment of loc... 0.069694 14 / 9145
GO:0006082 organic acid metabol... 0.415917 5 / 4211
GO:0006952 defense response 0.187810 8 / 5519
GO:0006979 response to oxidativ... 0.021234 5 / 1576
GO:0009628 response to abiotic ... 0.077596 12 / 7633
GO:1901700 response to oxygen−c... 0.005531 13 / 5888
GO:0001101 response to acid che... 0.015379 10 / 4551
GO:0010035 response to inorgani... 0.001372 11 / 3788
GO:0006810 transport 0.064681 14 / 9043
GO:0043436 oxoacid metabolic pr... 0.415114 5 / 4207
GO:0006954 inflammatory respons... 0.021837 1 / 22
GO:0000302 response to reactive... 0.004853 4 / 676
GO:0009415 response to water 0.043029 4 / 1311
GO:0071702 organic substance tr... 0.008815 10 / 4178
GO:0071705 nitrogen compound tr... 0.052543 7 / 3375
GO:0019752 carboxylic acid meta... 0.337909 5 / 3823
GO:1901568 fatty acid derivativ... 0.128546 1 / 137
GO:0002532 production of molecu... 0.004006 1 / 4
GO:0042542 response to hydrogen... 0.002706 3 / 274
GO:0009414 response to water de... 0.041157 4 / 1292
GO:0042886 amide transport 0.050506 6 / 2655
GO:0006690 icosanoid metabolic ... 0.009985 1 / 10
GO:0002538 arachidonic acid met... 0.004006 1 / 4
GO:0009269 response to desiccat... 4.64e−05 3 / 68
GO:0015833 peptide transport 0.047841 6 / 2619
GO:0006691 leukotriene metaboli... 0.004006 1 / 4
GO:0002540 leukotriene producti... 0.004006 1 / 4
GO:0006857 oligopeptide transpo... 0.030742 2 / 272
GO:0042938 dipeptide transport 0.000612 2 / 36
GO:0042939 tripeptide transport 0.000612 2 / 36

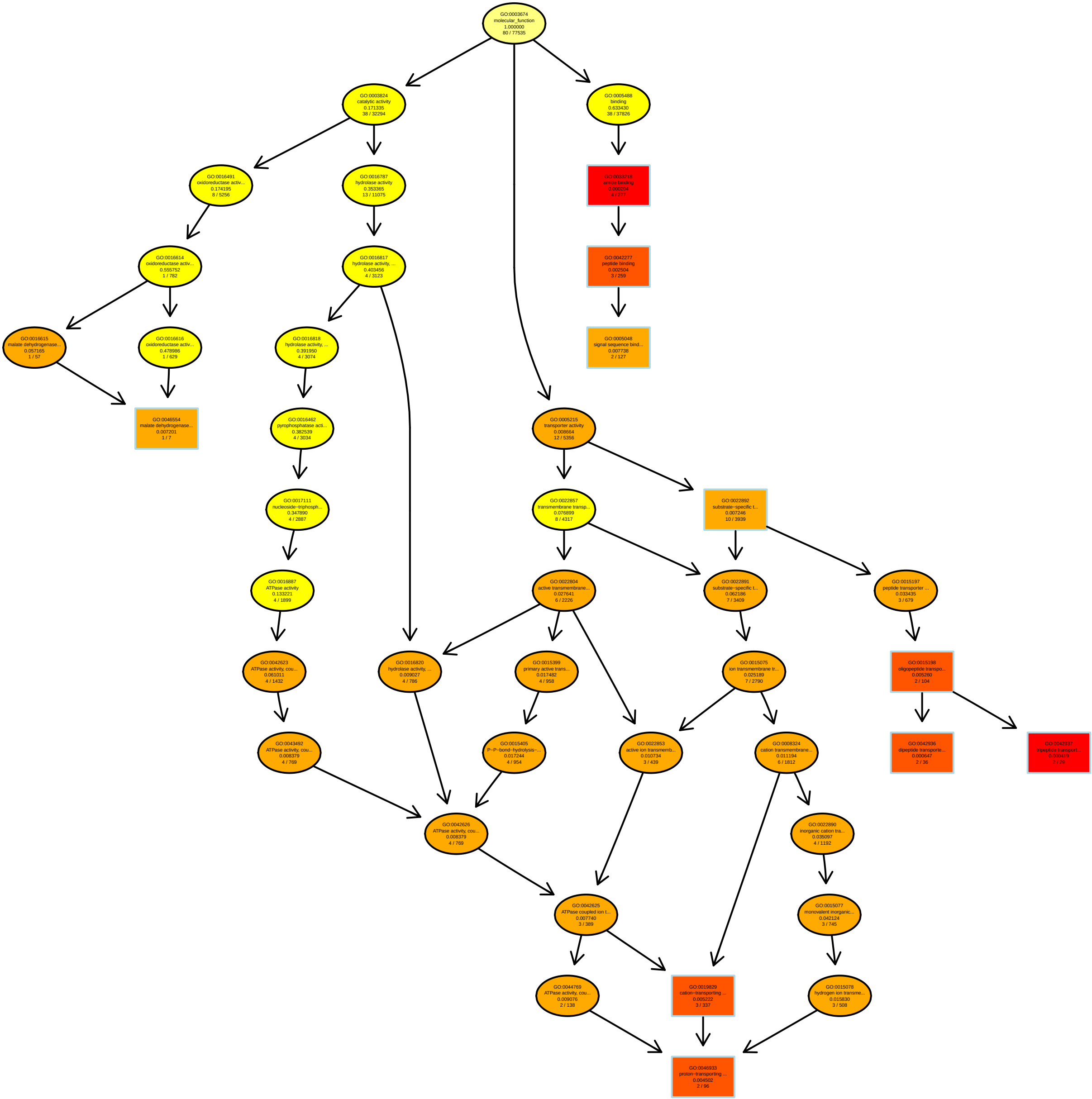
GO:0003674 molecular_function 1.000000 80 / 77535
GO:0003824 catalytic activity 0.171335 38 / 32294
GO:0005488 binding 0.633430 38 / 37826
GO:0016491 oxidoreductase activ... 0.174195 8 / 5256
GO:0016787 hydrolase activity 0.353365 13 / 11075
GO:0033218 amide binding 0.000204 4 / 277
GO:0016614 oxidoreductase activ... 0.555752 1 / 782
GO:0016817 hydrolase activity, ... 0.403456 4 / 3123
GO:0042277 peptide binding 0.002504 3 / 259
GO:0016615 malate dehydrogenase... 0.057165 1 / 57
GO:0016616 oxidoreductase activ... 0.478986 1 / 629
GO:0016818 hydrolase activity, ... 0.391950 4 / 3074
GO:0005048 signal sequence bind... 0.007738 2 / 127
GO:0046554 malate dehydrogenase... 0.007201 1 / 7
GO:0016462 pyrophosphatase acti... 0.382539 4 / 3034
GO:0005215 transporter activity 0.008664 12 / 5356
GO:0017111 nucleoside−triphosph... 0.347890 4 / 2887
GO:0022857 transmembrane transp... 0.076899 8 / 4317
GO:0022892 substrate−specific t... 0.007246 10 / 3939
GO:0016887 ATPase activity 0.133221 4 / 1899
GO:0022804 active transmembrane... 0.027641 6 / 2226
GO:0022891 substrate−specific t... 0.062186 7 / 3409
GO:0015197 peptide transporter ... 0.033435 3 / 679
GO:0042623 ATPase activity, cou... 0.061011 4 / 1432
GO:0016820 hydrolase activity, ... 0.009027 4 / 786
GO:0015399 primary active trans... 0.017482 4 / 958
GO:0015075 ion transmembrane tr... 0.025189 7 / 2790
GO:0015198 oligopeptide transpo... 0.005260 2 / 104
GO:0043492 ATPase activity, cou... 0.008379 4 / 769
GO:0015405 P−P−bond−hydrolysis−... 0.017244 4 / 954
GO:0022853 active ion transmemb... 0.010734 3 / 439
GO:0008324 cation transmembrane... 0.011194 6 / 1812
GO:0042936 dipeptide transporte... 0.000647 2 / 36
GO:0042937 tripeptide transport... 0.000419 2 / 29
GO:0042626 ATPase activity, cou... 0.008379 4 / 769
GO:0022890 inorganic cation tra... 0.035097 4 / 1192
GO:0042625 ATPase coupled ion t... 0.007740 3 / 389
GO:0015077 monovalent inorganic... 0.042124 3 / 745
GO:0044769 ATPase activity, cou... 0.009076 2 / 138
GO:0019829 cation−transporting ... 0.005222 3 / 337
GO:0015078 hydrogen ion transme... 0.015830 3 / 508
GO:0046933 proton−transporting ... 0.004502 2 / 96

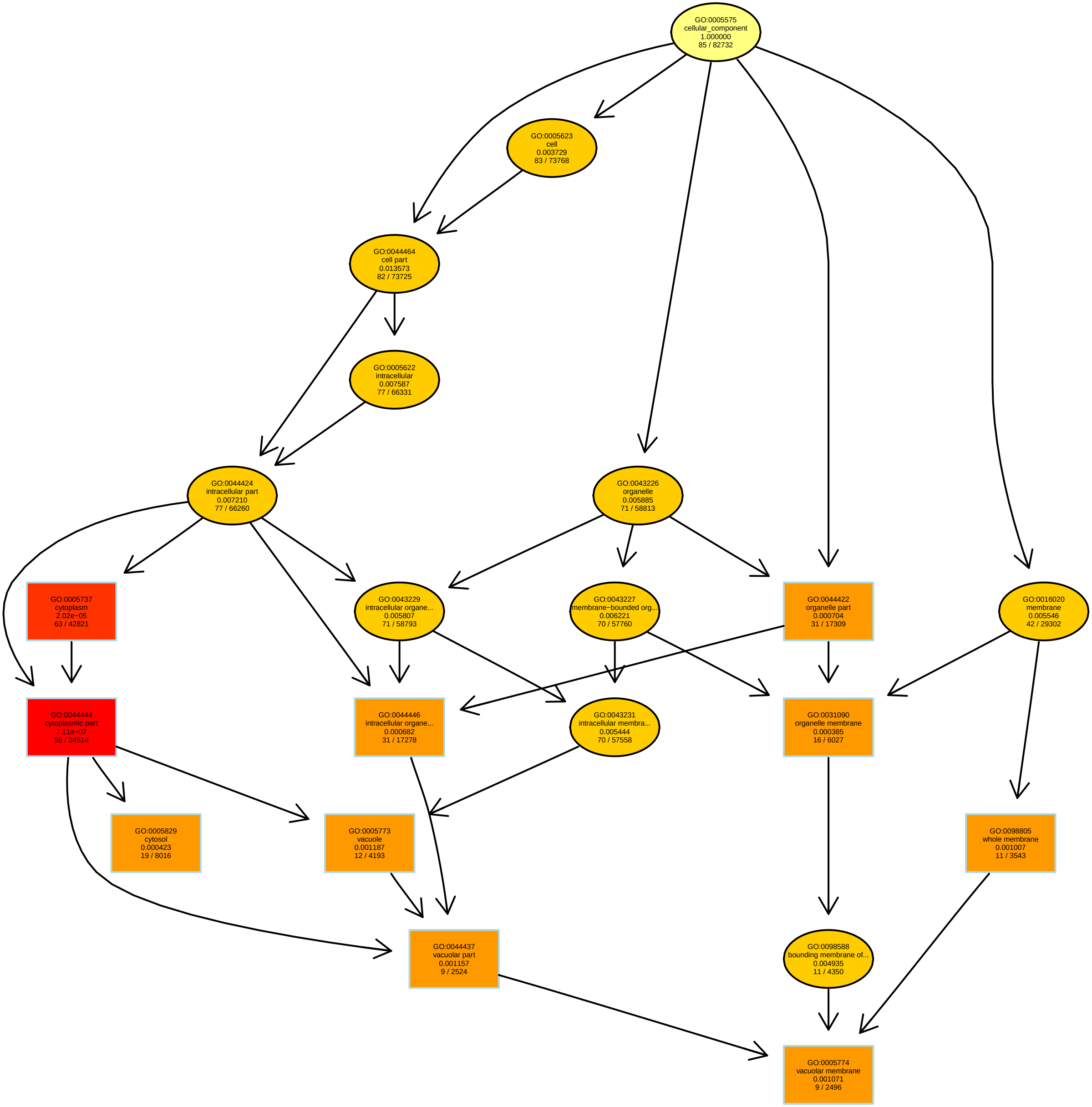
GO:0005575
cellular_component
1.000000
85 / 82732
GO:0005623
cell
0.003729
83 / 73768
GO:0044464
cell part
0.013573
82 / 73725
GO:0005622
intracellular
0.007587
77 / 66331
GO:0044424
intracellular part
0.007210
77 / 66260
GO:0043226
organelle
0.005885
71 / 58813
GO:0005737
cytoplasm
2.02e−05
63 / 42821
GO:0043229
intracellular organe...
0.005807
71 / 58793
GO:0043227
membrane−bounded org...
0.006221
70 / 57760
GO:0044422
organelle part
0.000704
31 / 17309
GO:0016020
membrane
0.005546
42 / 29302
GO:0044444
cytoplasmic part
7.11e−07
58 / 34514
GO:0044446
intracellular organe...
0.000682
31 / 17278
GO:0043231
intracellular membra...
0.005444
70 / 57558
GO:0031090
organelle membrane
0.000385
16 / 6027
GO:0005829
cytosol
0.000423
19 / 8016
GO:0005773
vacuole
0.001187
12 / 4193
GO:0098805
whole membrane
0.001007
11 / 3543
GO:0044437
vacuolar part
0.001157
9 / 2524
GO:0098588
bounding membrane of...
0.004935
11 / 4350
GO:0005774
vacuolar membrane
0.001071
9 / 2496
