# Supplementary material for: Differential Alternative Splicing Genes and Isoform Regulation Networks of Rapeseed (Brassica napus L.) Infected with Sclerotinia sclerotiorum
Source: Genes (Basel). 2020 Jul 13;11(7):784. doi: 10.3390/genes11070784 (PMC7397149; doi:10.3390/genes11070784)
Supplement: Supplementary file 1 [file genes-11-00784-s001.zip › Fig. S2 Enriched GO terms of genes in the 85 WGCNA modules.zip!/Fig. S2/CytoNodebisque4-go.pdf]

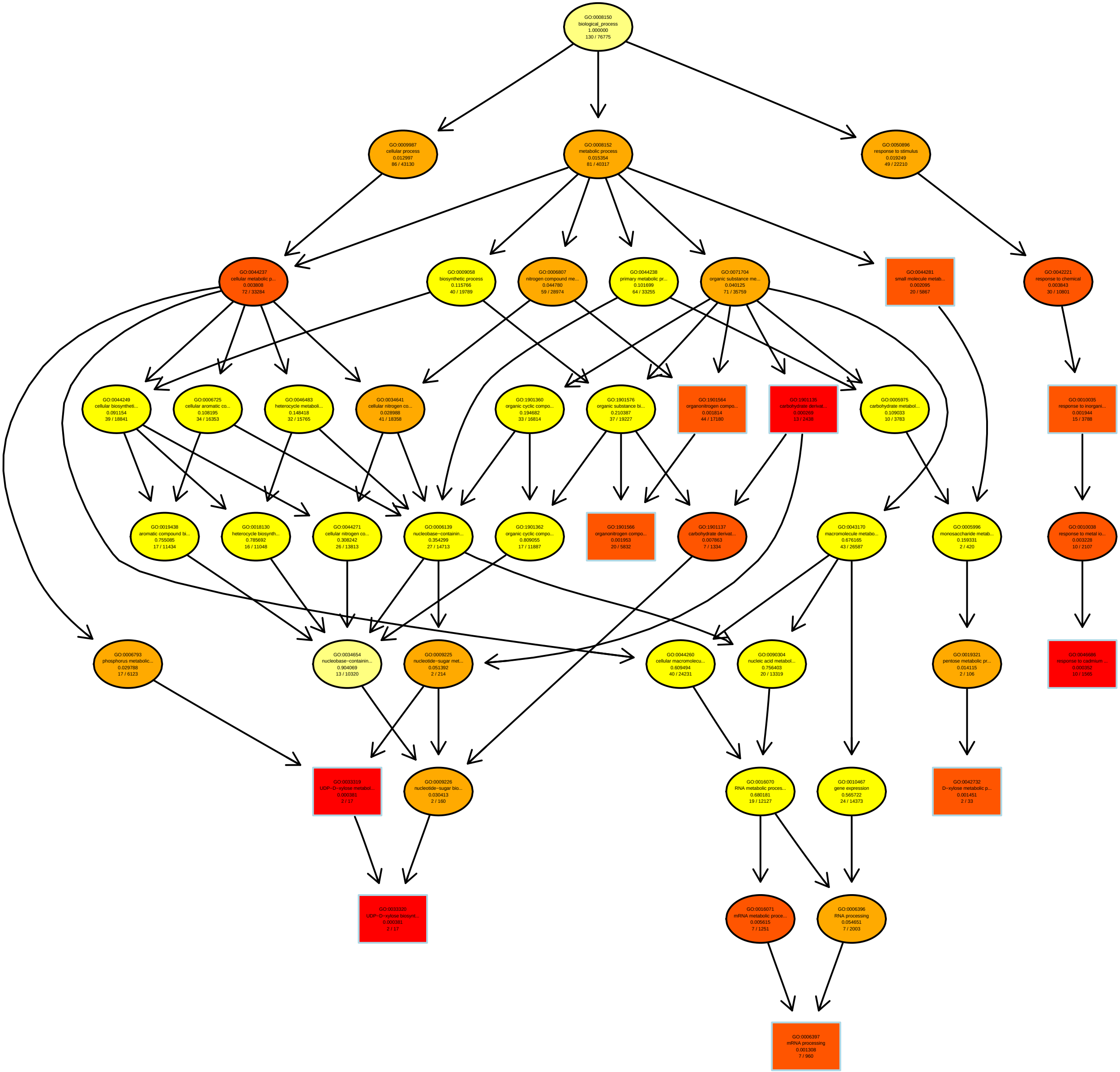

GO:0008150 biological_process 1.000000 130 / 76775
GO:0009987 cellular process 0.012997 86 / 43130
GO:0008152 metabolic process 0.015354 81 / 40317
GO:0050896 response to stimulus 0.019249 49 / 22210
GO:0044237 cellular metabolic p... 0.003808 72 / 33284
GO:0009058 biosynthetic process 0.115766 40 / 19789
GO:0006807 nitrogen compound me... 0.044780 59 / 28974
GO:0044238 primary metabolic pr... 0.101699 64 / 33255
GO:0071704 organic substance me... 0.040125 71 / 35759
GO:0044281 small molecule metab... 0.002095 20 / 5867
GO:0042221 response to chemical 0.003843 30 / 10801
GO:0044249 cellular biosyntheti... 0.091154 39 / 18841
GO:0006725 cellular aromatic co... 0.108195 34 / 16353
GO:0046483 heterocycle metaboli... 0.148418 32 / 15765
GO:0034641 cellular nitrogen co... 0.028988 41 / 18358
GO:1901360 organic cyclic compo... 0.194682 33 / 16814
GO:1901576 organic substance bi... 0.210387 37 / 19227
GO:1901564 organonitrogen compo... 0.001814 44 / 17180
GO:1901135 carbohydrate derivat... 0.000269 13 / 2438
GO:0005975 carbohydrate metabol... 0.109033 10 / 3783
GO:0010035 response to inorgani... 0.001944 15 / 3788
GO:0019438 aromatic compound bi... 0.755085 17 / 11434
GO:0018130 heterocycle biosynth... 0.785692 16 / 11048
GO:0044271 cellular nitrogen co... 0.308242 26 / 13813
GO:0006139 nucleobase−containin... 0.354299 27 / 14713
GO:1901362 organic cyclic compo... 0.809055 17 / 11887
GO:1901566 organonitrogen compo... 0.001953 20 / 5832
GO:1901137 carbohydrate derivat... 0.007863 7 / 1334
GO:0043170 macromolecule metabo... 0.676165 43 / 26587
GO:0005996 monosaccharide metab... 0.159331 2 / 420
GO:0010038 response to metal io... 0.003228 10 / 2107
GO:0006793 phosphorus metabolic... 0.029788 17 / 6123
GO:0034654 nucleobase−containin... 0.904069 13 / 10320
GO:0009225 nucleotide−sugar met... 0.051392 2 / 214
GO:0044260 cellular macromolecu... 0.609494 40 / 24231
GO:0090304 nucleic acid metabol... 0.756403 20 / 13319
GO:0019321 pentose metabolic pr... 0.014115 2 / 106
GO:0046686 response to cadmium ... 0.000352 10 / 1565
GO:0033319 UDP−D−xylose metabol... 0.000381 2 / 17
GO:0009226 nucleotide−sugar bio... 0.030413 2 / 160
GO:0016070 RNA metabolic proces... 0.680181 19 / 12127
GO:0010467 gene expression 0.565722 24 / 14373
GO:0042732 D−xylose metabolic p... 0.001451 2 / 33
GO:0033320 UDP−D−xylose biosynt... 0.000381 2 / 17
GO:0016071 mRNA metabolic proce... 0.005615 7 / 1251
GO:0006396 RNA processing 0.054651 7 / 2003
GO:0006397 mRNA processing 0.001308 7 / 960

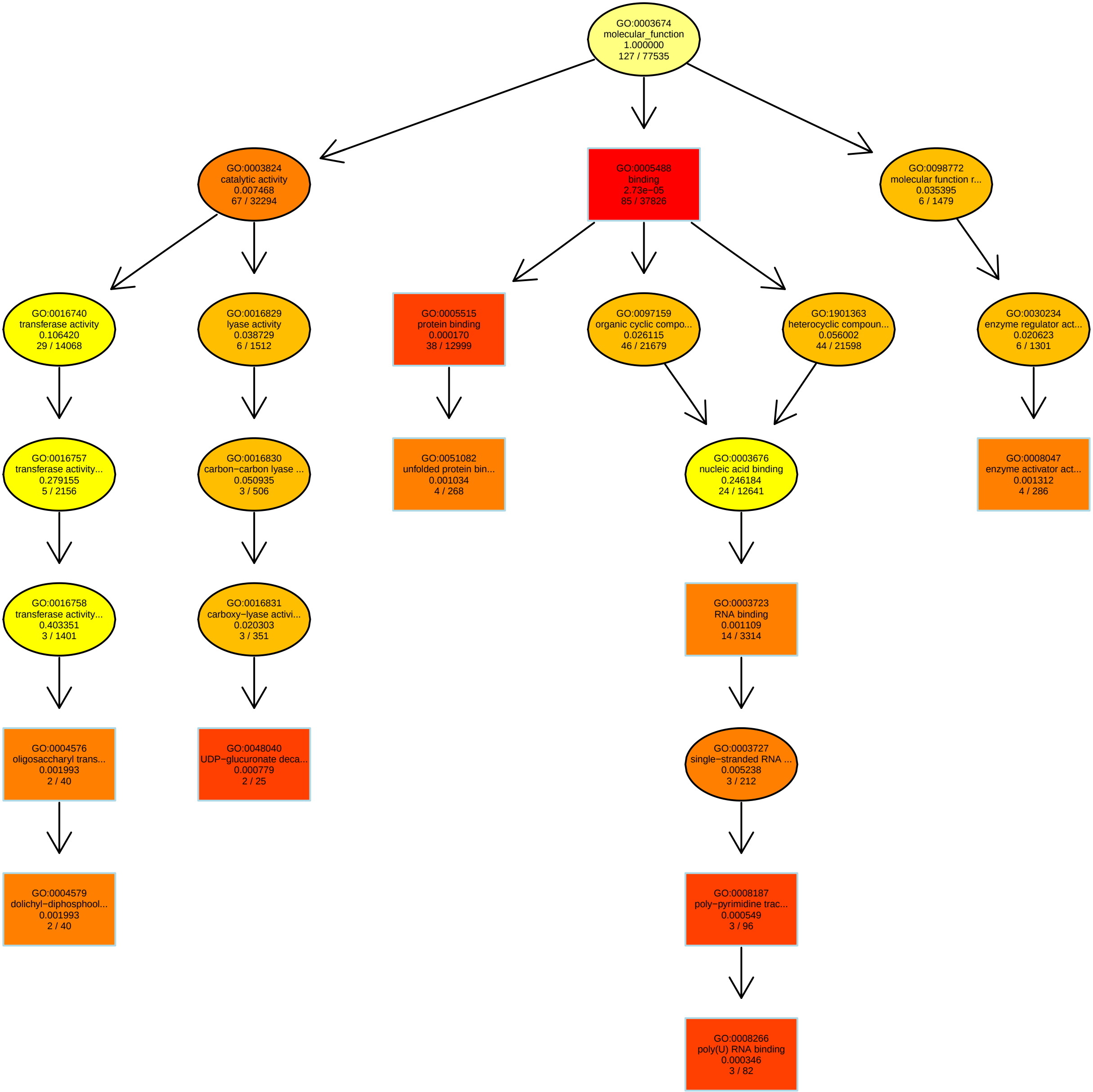
GO:0003674 molecular_function 1.000000 127 / 77535
GO:0003824 catalytic activity 0.007468 67 / 32294
GO:0005488 binding 2.73e−05 85 / 37826
GO:0098772 molecular function r... 0.035395 6 / 1479
GO:0016740 transferase activity 0.106420 29 / 14068
GO:0016829 lyase activity 0.038729 6 / 1512
GO:0005515 protein binding 0.000170 38 / 12999
GO:0097159 organic cyclic compo... 0.026115 46 / 21679
GO:1901363 heterocyclic compoun... 0.056002 44 / 21598
GO:0030234 enzyme regulator act... 0.020623 6 / 1301
GO:0016757 transferase activity... 0.279155 5 / 2156
GO:0016830 carbon−carbon lyase ... 0.050935 3 / 506
GO:0051082 unfolded protein bin... 0.001034 4 / 268
GO:0003676 nucleic acid binding 0.246184 24 / 12641
GO:0008047 enzyme activator act... 0.001312 4 / 286
GO:0016758 transferase activity... 0.403351 3 / 1401
GO:0016831 carboxy−lyase activi... 0.020303 3 / 351
GO:0003723 RNA binding 0.001109 14 / 3314
GO:0004576 oligosaccharyl trans... 0.001993 2 / 40
GO:0048040 UDP−glucuronate deca... 0.000779 2 / 25
GO:0003727 single−stranded RNA ... 0.005238 3 / 212
GO:0004579 dolichyl−diphosphool... 0.001993 2 / 40
GO:0008187 poly−pyrimidine trac... 0.000549 3 / 96
GO:0008266 poly(U) RNA binding 0.000346 3 / 82

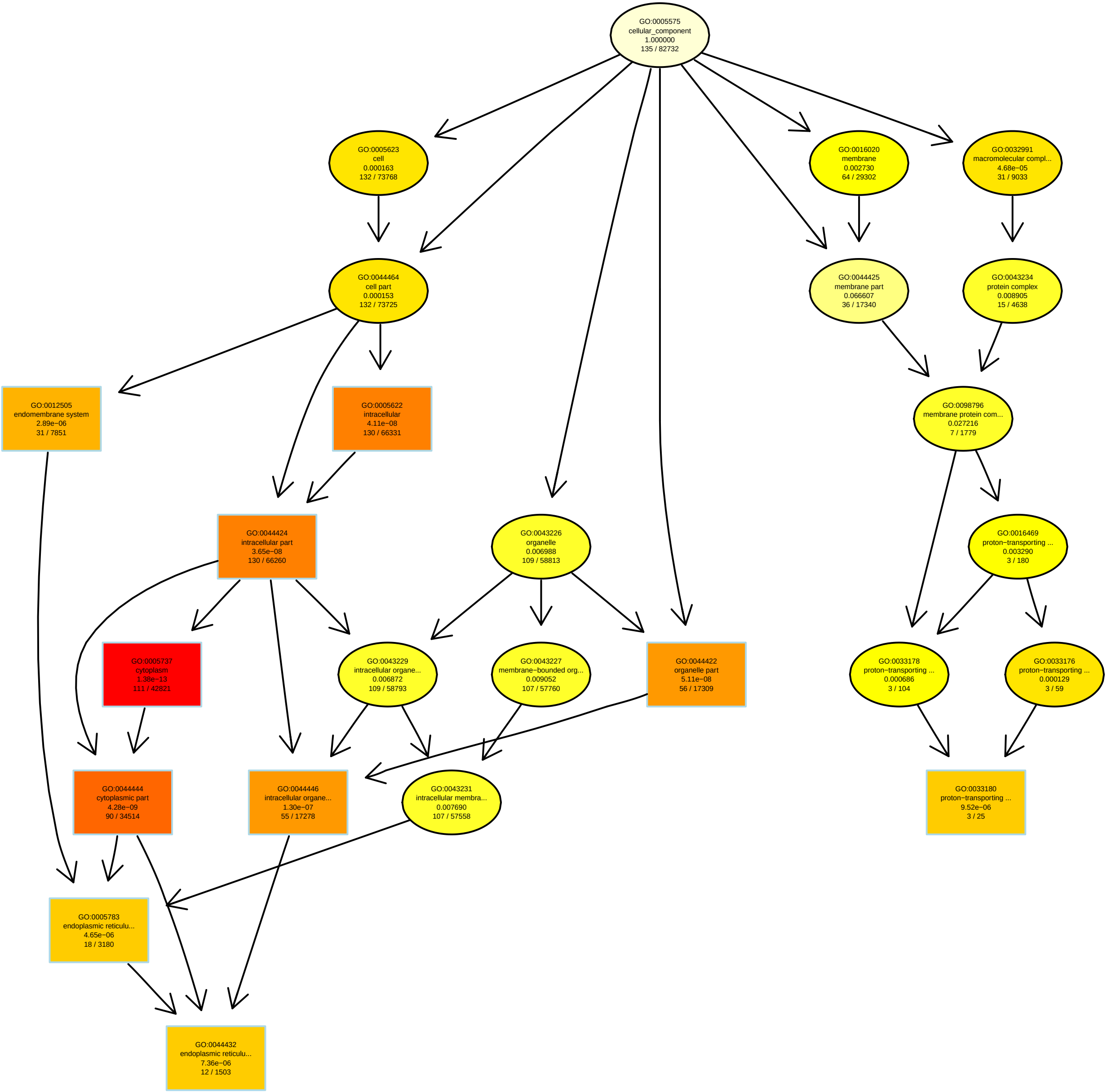

GO:0005575
cellular_component
1.000000
135 / 82732
GO:0005623
cell
0.000163
132 / 73768
GO:0016020
membrane
0.002730
64 / 29302
GO:0032991
macromolecular compl...
4.68e−05
31 / 9033
GO:0044464
cell part
0.000153
132 / 73725
GO:0044425
membrane part
0.066607
36 / 17340
GO:0043234
protein complex
0.008905
15 / 4638
GO:0012505
endomembrane system
2.89e−06
31 / 7851
GO:0005622
intracellular
4.11e−08
130 / 66331
GO:0098796
membrane protein com...
0.027216
7 / 1779
GO:0044424
intracellular part
3.65e−08
130 / 66260
GO:0043226
organelle
0.006988
109 / 58813
GO:0016469
proton−transporting ...
0.003290
3 / 180
GO:0005737
cytoplasm
1.38e−13
111 / 42821
GO:0043229
intracellular organe...
0.006872
109 / 58793
GO:0043227
membrane−bounded org...
0.009052
107 / 57760
GO:0044422
organelle part
5.11e−08
56 / 17309
GO:0033178
proton−transporting ...
0.000686
3 / 104
GO:0033176
proton−transporting ...
0.000129
3 / 59
GO:0044444
cytoplasmic part
4.28e−09
90 / 34514
GO:0044446
intracellular organe...
1.30e−07
55 / 17278
GO:0043231
intracellular membra...
0.007690
107 / 57558
GO:0033180
proton−transporting ...
9.52e−06
3 / 25
GO:0005783
endoplasmic reticulu...
4.65e−06
18 / 3180
GO:0044432
endoplasmic reticulu...
7.36e−06
12 / 1503
