# Supplementary material for: Differential Alternative Splicing Genes and Isoform Regulation Networks of Rapeseed (Brassica napus L.) Infected with Sclerotinia sclerotiorum
Source: Genes (Basel). 2020 Jul 13;11(7):784. doi: 10.3390/genes11070784 (PMC7397149; doi:10.3390/genes11070784)
Supplement: Supplementary file 1 [file genes-11-00784-s001.zip › Fig. S2 Enriched GO terms of genes in the 85 WGCNA modules.zip!/Fig. S2/CytoNodebrown2-go.pdf]

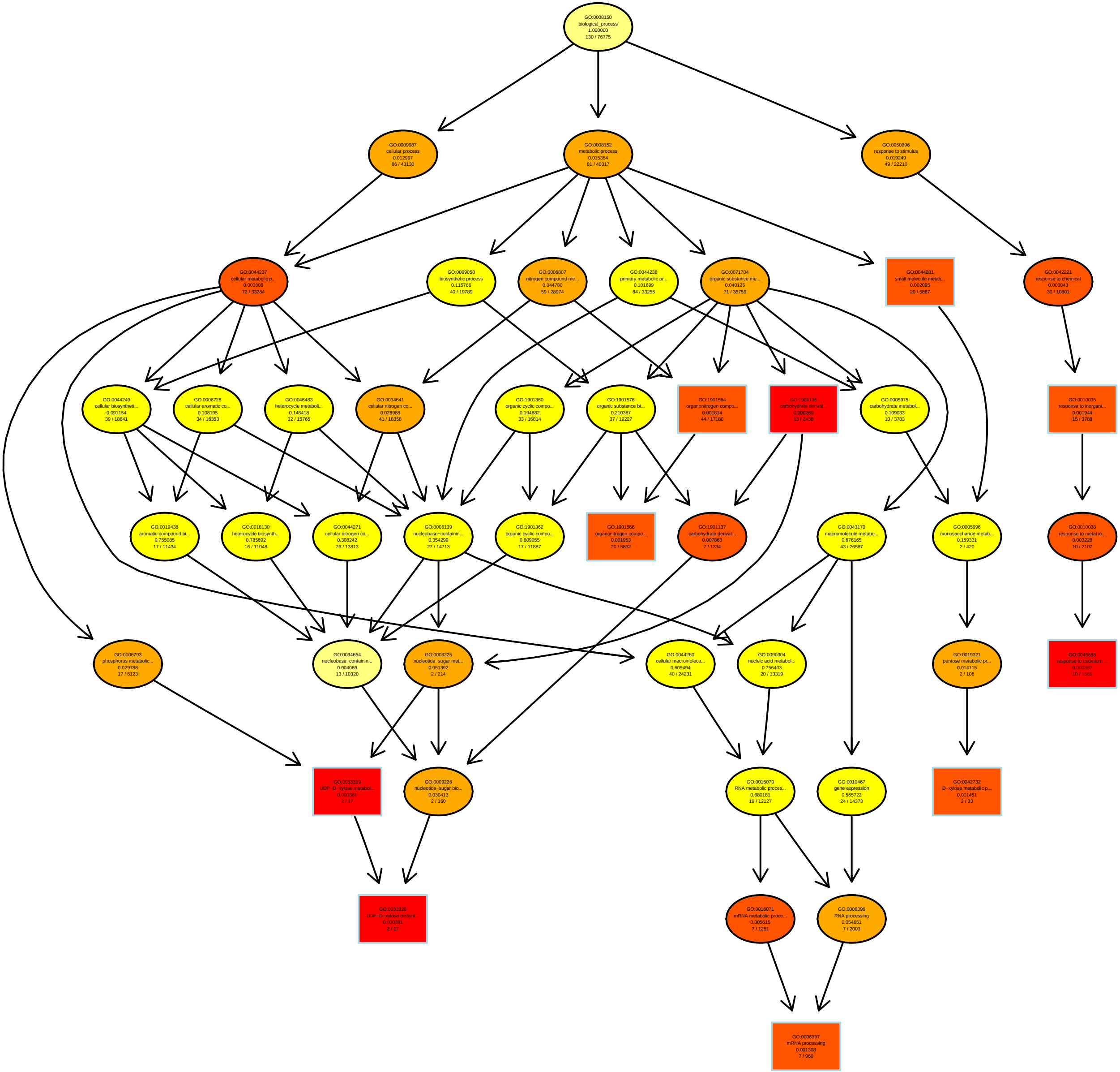
GO:0008150
biological_process
1.000000
130 / 76775
GO:0009987
cellular process
0.012997
86 / 43130
GO:0008152
metabolic process
0.015354
81 / 40317
GO:0050896
response to stimulus
0.019249
49 / 22210
GO:0044237
cellular metabolic p...
0.003808
72 / 33284
GO:0009058
biosynthetic process
0.115766
40 / 19789
GO:0006807
nitrogen compound me...
0.044780
59 / 28974
GO:0044238
primary metabolic pr...
0.101699
64 / 33255
GO:0071704
organic substance me...
0.040125
71 / 35759
GO:0044281
small molecule metab...
0.002095
20 / 5867
GO:0042221
response to chemical
0.003843
30 / 10801
GO:0044249
cellular biosyntheti...
0.091154
39 / 18841
GO:0006725
cellular aromatic co...
0.108195
34 / 16353
GO:0046483
heterocycle metaboli...
0.148418
32 / 15765
GO:0034641
cellular nitrogen co...
0.028988
41 / 18358
GO:1901360
organic cyclic compo...
0.194682
33 / 16814
GO:1901576
organic substance bi...
0.210387
37 / 19227
GO:1901564
organonitrogen compo...
0.001814
44 / 17180
GO:1901135
carbohydrate derivat...
0.000269
13 / 2438
GO:0005975
carbohydrate metabol...
0.109033
10 / 3783
GO:0010035
response to inorgani...
0.001944
15 / 3788
GO:0019438
aromatic compound bi...
0.755085
17 / 11434
GO:0018130
heterocycle biosynth...
0.785692
16 / 11048
GO:0044271
cellular nitrogen co...
0.308242
26 / 13813
GO:0006139
nucleobase−containin...
0.354299
27 / 14713
GO:1901362
organic cyclic compo...
0.809055
17 / 11887
GO:1901566
organonitrogen compo...
0.001953
20 / 5832
GO:1901137
carbohydrate derivat...
0.007863
7 / 1334
GO:0043170
macromolecule metabo...
0.676165
43 / 26587
GO:0005996
monosaccharide metab...
0.159331
2 / 420
GO:0010038
response to metal io...
0.003228
10 / 2107
GO:0006793
phosphorus metabolic...
0.029788
17 / 6123
GO:0034654
nucleobase−containin...
0.904069
13 / 10320
GO:0009225
nucleotide−sugar met...
0.051392
2 / 214
GO:0044260
cellular macromolecu...
0.609494
40 / 24231
GO:0090304
nucleic acid metabol...
0.756403
20 / 13319
GO:0019321
pentose metabolic pr...
0.014115
2 / 106
GO:0046686
response to cadmium ...
0.000352
10 / 1565
GO:0033319
UDP−D−xylose metabol...
0.000381
2 / 17
GO:0009226
nucleotide−sugar bio...
0.030413
2 / 160
GO:0016070
RNA metabolic proces...
0.680181
19 / 12127
GO:0010467
gene expression
0.565722
24 / 14373
GO:0042732
D−xylose metabolic p...
0.001451
2 / 33
GO:0033320
UDP−D−xylose biosynt...
0.000381
2 / 17
GO:0016071
mRNA metabolic proce...
0.005615
7 / 1251
GO:0006396
RNA processing
0.054651
7 / 2003
GO:0006397
mRNA processing
0.001308
7 / 960

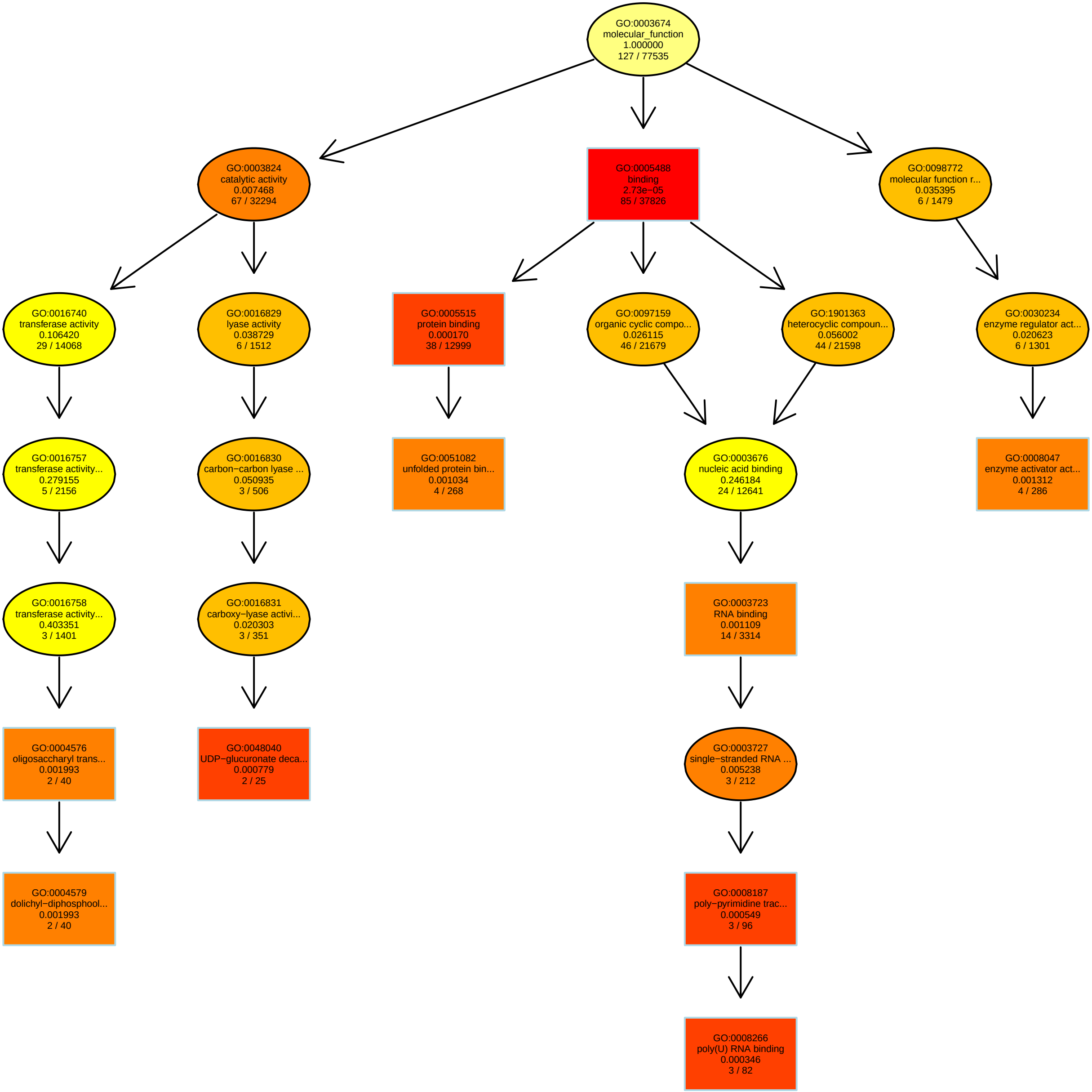
GO:0003674
molecular_function
1.000000
127 / 77535
GO:0003824
catalytic activity
0.007468
67 / 32294
GO:0005488
binding
2.73e−05
85 / 37826
GO:0098772
molecular function r...
0.035395
6 / 1479
GO:0016740
transferase activity
0.106420
29 / 14068
GO:0016829
lyase activity
0.038729
6 / 1512
GO:0005515
protein binding
0.000170
38 / 12999
GO:0097159
organic cyclic compo...
0.026115
46 / 21679
GO:1901363
heterocyclic compoun...
0.056002
44 / 21598
GO:0030234
enzyme regulator act...
0.020623
6 / 1301
GO:0016757
transferase activity...
0.279155
5 / 2156
GO:0016830
carbon−carbon lyase ...
0.050935
3 / 506
GO:0051082
unfolded protein bin...
0.001034
4 / 268
GO:0003676
nucleic acid binding
0.246184
24 / 12641
GO:0008047
enzyme activator act...
0.001312
4 / 286
GO:0016758
transferase activity...
0.403351
3 / 1401
GO:0016831
carboxy−lyase activi...
0.020303
3 / 351
GO:0003723
RNA binding
0.001109
14 / 3314
GO:0004576
oligosaccharyl trans...
0.001993
2 / 40
GO:0048040
UDP−glucuronate deca...
0.000779
2 / 25
GO:0003727
single−stranded RNA ...
0.005238
3 / 212
GO:0004579
dolichyl−diphosphool...
0.001993
2 / 40
GO:0008187
poly−pyrimidine trac...
0.000549
3 / 96
GO:0008266
poly(U) RNA binding
0.000346
3 / 82

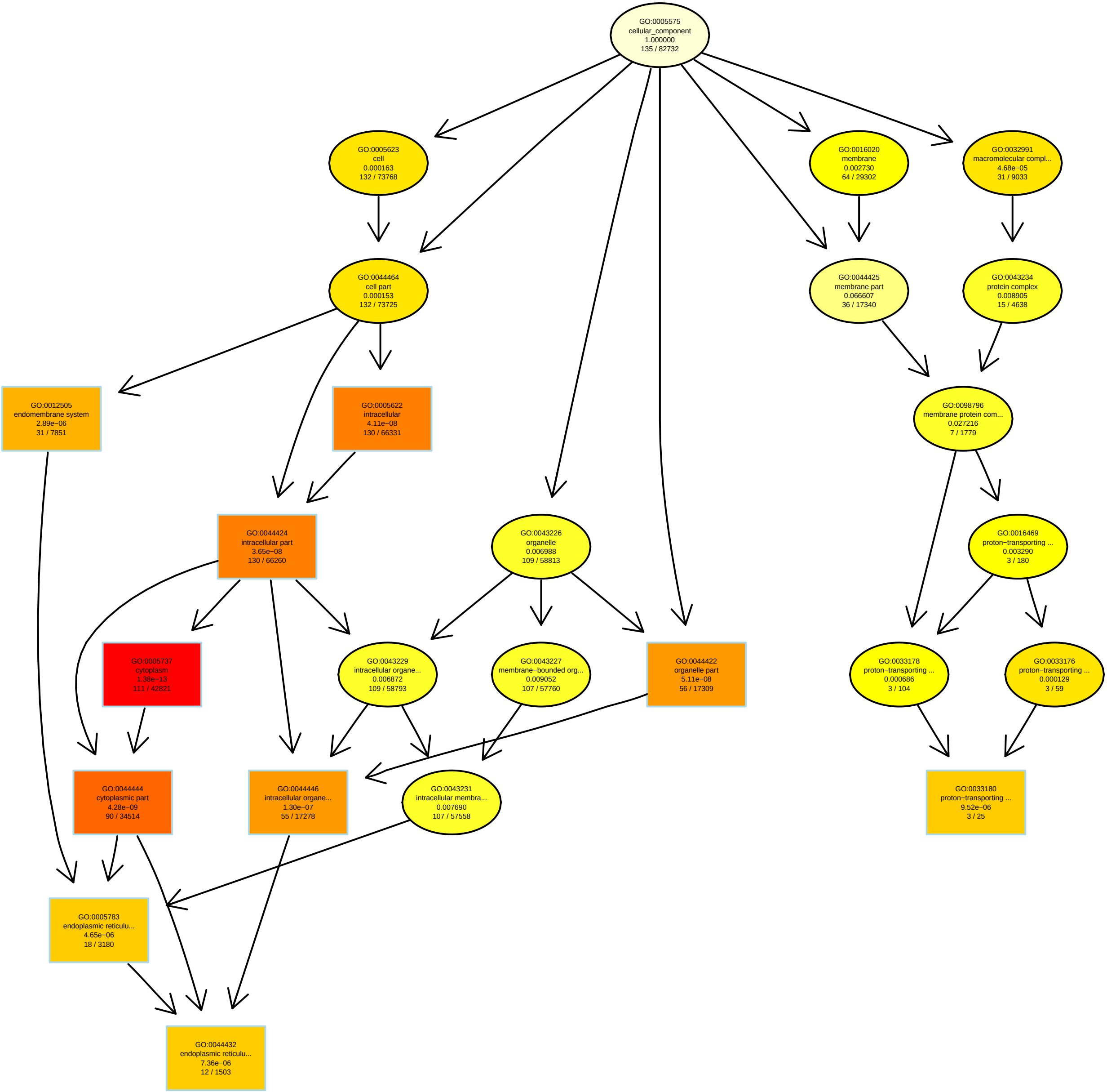

GO:0005575
cellular_component
1.000000
135 / 82732
GO:0005623
cell
0.000163
132 / 73768
GO:0016020
membrane
0.002730
64 / 29302
GO:0032991
macromolecular compl...
4.68e−05
31 / 9033
GO:0044464
cell part
0.000153
132 / 73725
GO:0044425
membrane part
0.066607
36 / 17340
GO:0043234
protein complex
0.008905
15 / 4638
GO:0012505
endomembrane system
2.89e−06
31 / 7851
GO:0005622
intracellular
4.11e−08
130 / 66331
GO:0098796
membrane protein com...
0.027216
7 / 1779
GO:0044424
intracellular part
3.65e−08
130 / 66260
GO:0043226
organelle
0.006988
109 / 58813
GO:0016469
proton−transporting ...
0.003290
3 / 180
GO:0005737
cytoplasm
1.38e−13
111 / 42821
GO:0043229
intracellular organe...
0.006872
109 / 58793
GO:0043227
membrane−bounded org...
0.009052
107 / 57760
GO:0044422
organelle part
5.11e−08
56 / 17309
GO:0033178
proton−transporting ...
0.000686
3 / 104
GO:0033176
proton−transporting ...
0.000129
3 / 59
GO:0044444
cytoplasmic part
4.28e−09
90 / 34514
GO:0044446
intracellular organe...
1.30e−07
55 / 17278
GO:0043231
intracellular membra...
0.007690
107 / 57558
GO:0033180
proton−transporting ...
9.52e−06
3 / 25
GO:0005783
endoplasmic reticulu...
4.65e−06
18 / 3180
GO:0044432
endoplasmic reticulu...
7.36e−06
12 / 1503
