# Supplementary material for: Differential Alternative Splicing Genes and Isoform Regulation Networks of Rapeseed (Brassica napus L.) Infected with Sclerotinia sclerotiorum
Source: Genes (Basel). 2020 Jul 13;11(7):784. doi: 10.3390/genes11070784 (PMC7397149; doi:10.3390/genes11070784)
Supplement: Supplementary file 1 [file genes-11-00784-s001.zip › Fig. S2 Enriched GO terms of genes in the 85 WGCNA modules.zip!/Fig. S2/CytoNodebrown4-go.pdf]

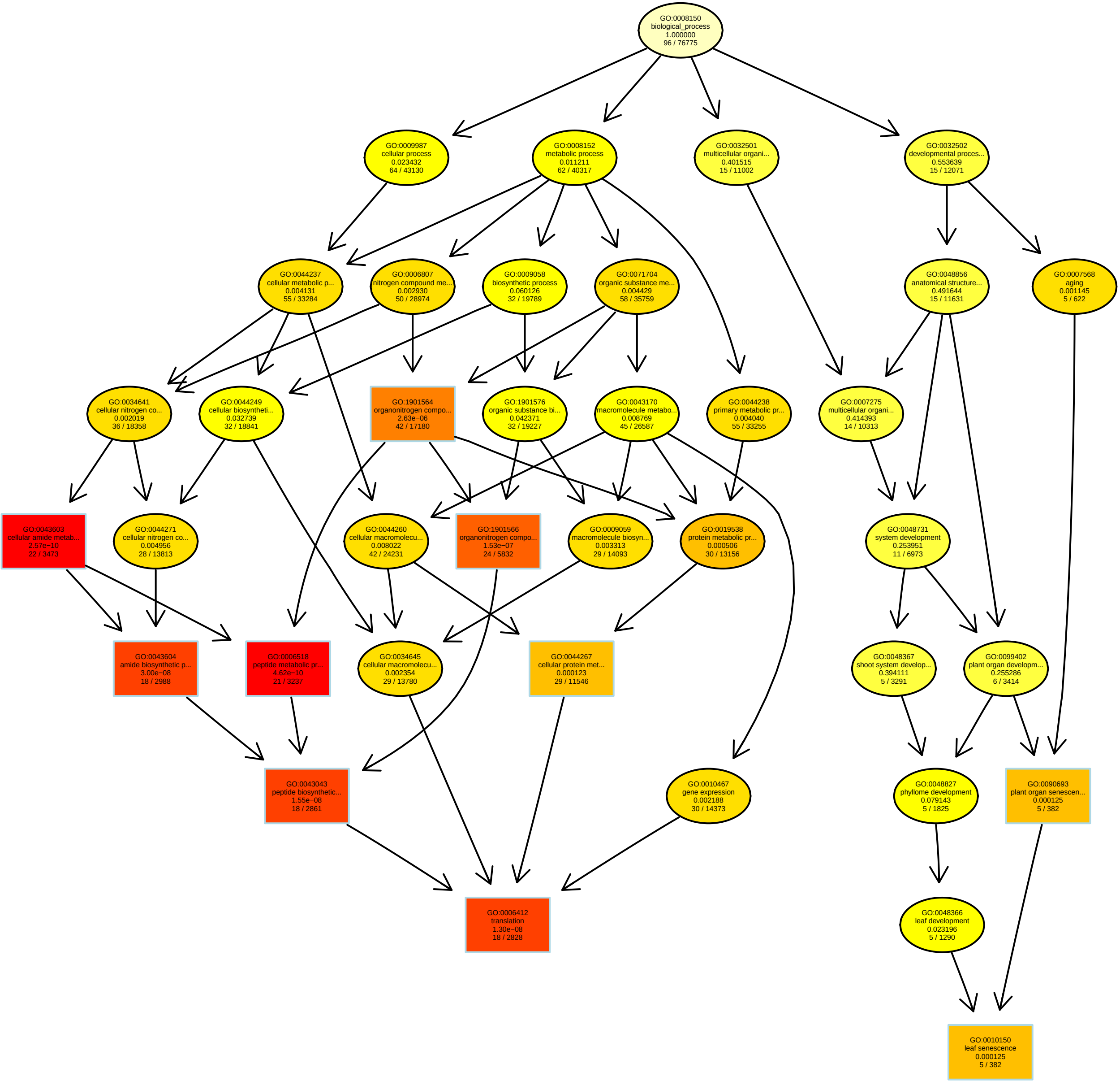
GO:0008150 biological_process 1.000000 96 / 76775
GO:0009987 cellular process 0.023432 64 / 43130
GO:0008152 metabolic process 0.011211 62 / 40317
GO:0032501 multicellular organi... 0.401515 15 / 11002
GO:0032502 developmental proces... 0.553639 15 / 12071
GO:0044237 cellular metabolic p... 0.004131 55 / 33284
GO:0006807 nitrogen compound me... 0.002930 50 / 28974
GO:0009058 biosynthetic process 0.060126 32 / 19789
GO:0071704 organic substance me... 0.004429 58 / 35759
GO:0048856 anatomical structure... 0.491644 15 / 11631
GO:0007568 aging 0.001145 5 / 622
GO:0034641 cellular nitrogen co... 0.002019 36 / 18358
GO:0044249 cellular biosyntheti... 0.032739 32 / 18841
GO:1901564 organonitrogen compo... 2.63e−06 42 / 17180
GO:1901576 organic substance bi... 0.042371 32 / 19227
GO:0043170 macromolecule metabo... 0.008769 45 / 26587
GO:0044238 primary metabolic pr... 0.004040 55 / 33255
GO:0007275 multicellular organi... 0.414393 14 / 10313
GO:0043603 cellular amide metab... 2.57e−10 22 / 3473
GO:0044271 cellular nitrogen co... 0.004956 28 / 13813
GO:0044260 cellular macromolecu... 0.008022 42 / 24231
GO:1901566 organonitrogen compo... 1.53e−07 24 / 5832
GO:0009059 macromolecule biosyn... 0.003313 29 / 14093
GO:0019538 protein metabolic pr... 0.000506 30 / 13156
GO:0048731 system development 0.253951 11 / 6973
GO:0043604 amide biosynthetic p... 3.00e−08 18 / 2988
GO:0006518 peptide metabolic pr... 4.62e−10 21 / 3237
GO:0034645 cellular macromolecu... 0.002354 29 / 13780
GO:0044267 cellular protein met... 0.000123 29 / 11546
GO:0048367 shoot system develop... 0.394111 5 / 3291
GO:0099402 plant organ developm... 0.255286 6 / 3414
GO:0043043 peptide biosynthetic... 1.55e−08 18 / 2861
GO:0010467 gene expression 0.002188 30 / 14373
GO:0048827 phyllome development 0.079143 5 / 1825
GO:0090693 plant organ senescen... 0.000125 5 / 382
GO:0006412 translation 1.30e−08 18 / 2828
GO:0048366 leaf development 0.023196 5 / 1290
GO:0010150 leaf senescence 0.000125 5 / 382

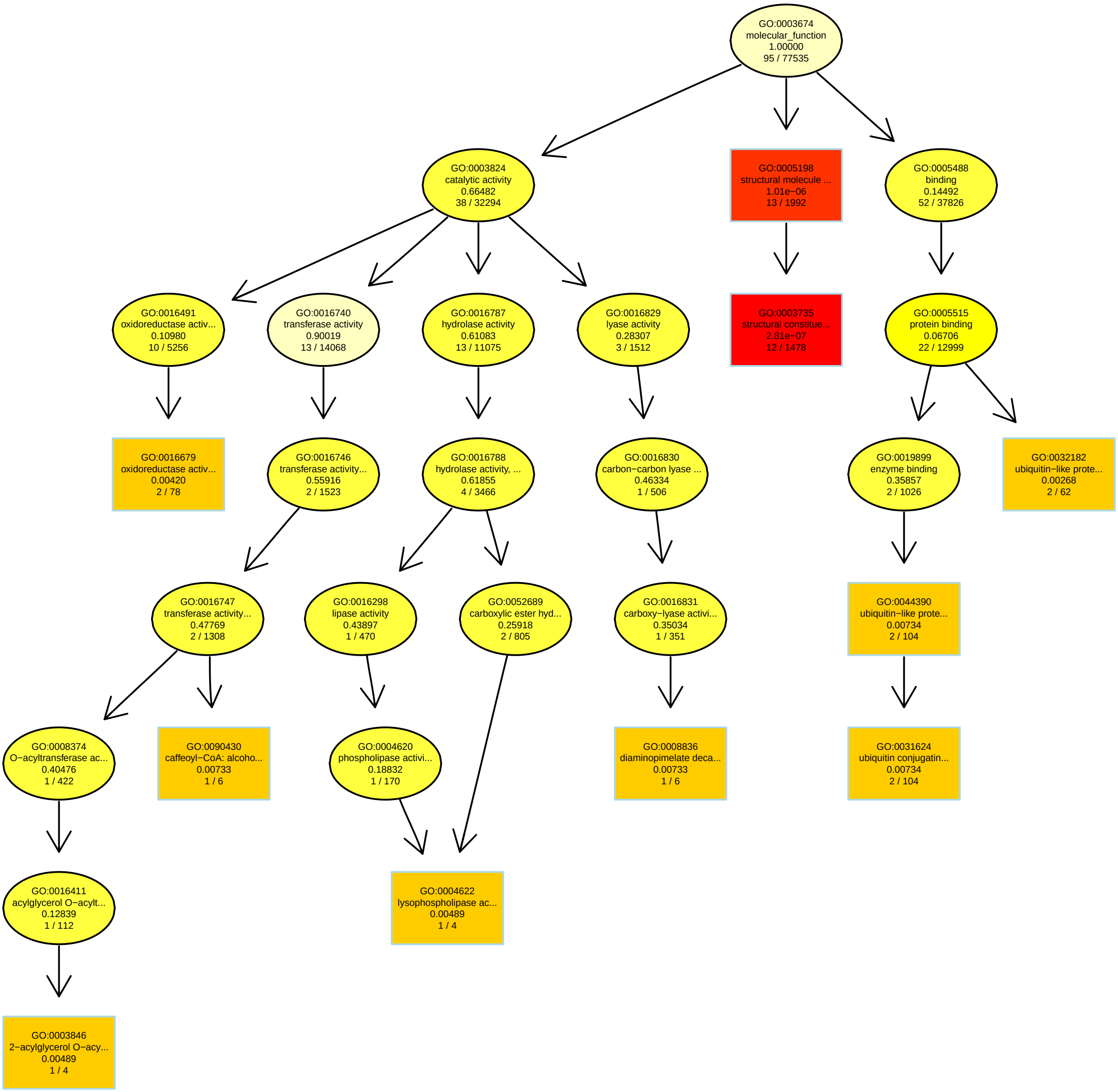

GO:0003674
molecular_function
1.00000
95 / 77535
GO:0003824
catalytic activity
0.66482
38 / 32294
GO:0005198
structural molecule ...
1.01e−06
13 / 1992
GO:0005488
binding
0.14492
52 / 37826
GO:0016491
oxidoreductase activ...
0.10980
10 / 5256
GO:0016740
transferase activity
0.90019
13 / 14068
GO:0016787
hydrolase activity
0.61083
13 / 11075
GO:0016829
lyase activity
0.28307
3 / 1512
GO:0003735
structural constitue...
2.81e−07
12 / 1478
GO:0005515
protein binding
0.06706
22 / 12999
GO:0016679
oxidoreductase activ...
0.00420
2 / 78
GO:0016746
transferase activity...
0.55916
2 / 1523
GO:0016788
hydrolase activity, ...
0.61855
4 / 3466
GO:0016830
carbon−carbon lyase ...
0.46334
1 / 506
GO:0019899
enzyme binding
0.35857
2 / 1026
GO:0032182
ubiquitin−like prote...
0.00268
2 / 62
GO:0016747
transferase activity...
0.47769
2 / 1308
GO:0016298
lipase activity
0.43897
1 / 470
GO:0052689
carboxylic ester hyd...
0.25918
2 / 805
GO:0016831
carboxy−lyase activi...
0.35034
1 / 351
GO:0044390
ubiquitin−like prote...
0.00734
2 / 104
GO:0008374
O−acyltransferase ac...
0.40476
1 / 422
GO:0090430
caffeoyl−CoA: alcoho...
0.00733
1 / 6
GO:0004620
phospholipase activi...
0.18832
1 / 170
GO:0008836
diaminopimelate deca...
0.00733
1 / 6
GO:0031624
ubiquitin conjugatin...
0.00734
2 / 104
GO:0016411
acylglycerol O−acylt...
0.12839
1 / 112
GO:0004622
lysophospholipase ac...
0.00489
1 / 4
GO:0003846
2−acylglycerol O−acy...
0.00489
1 / 4

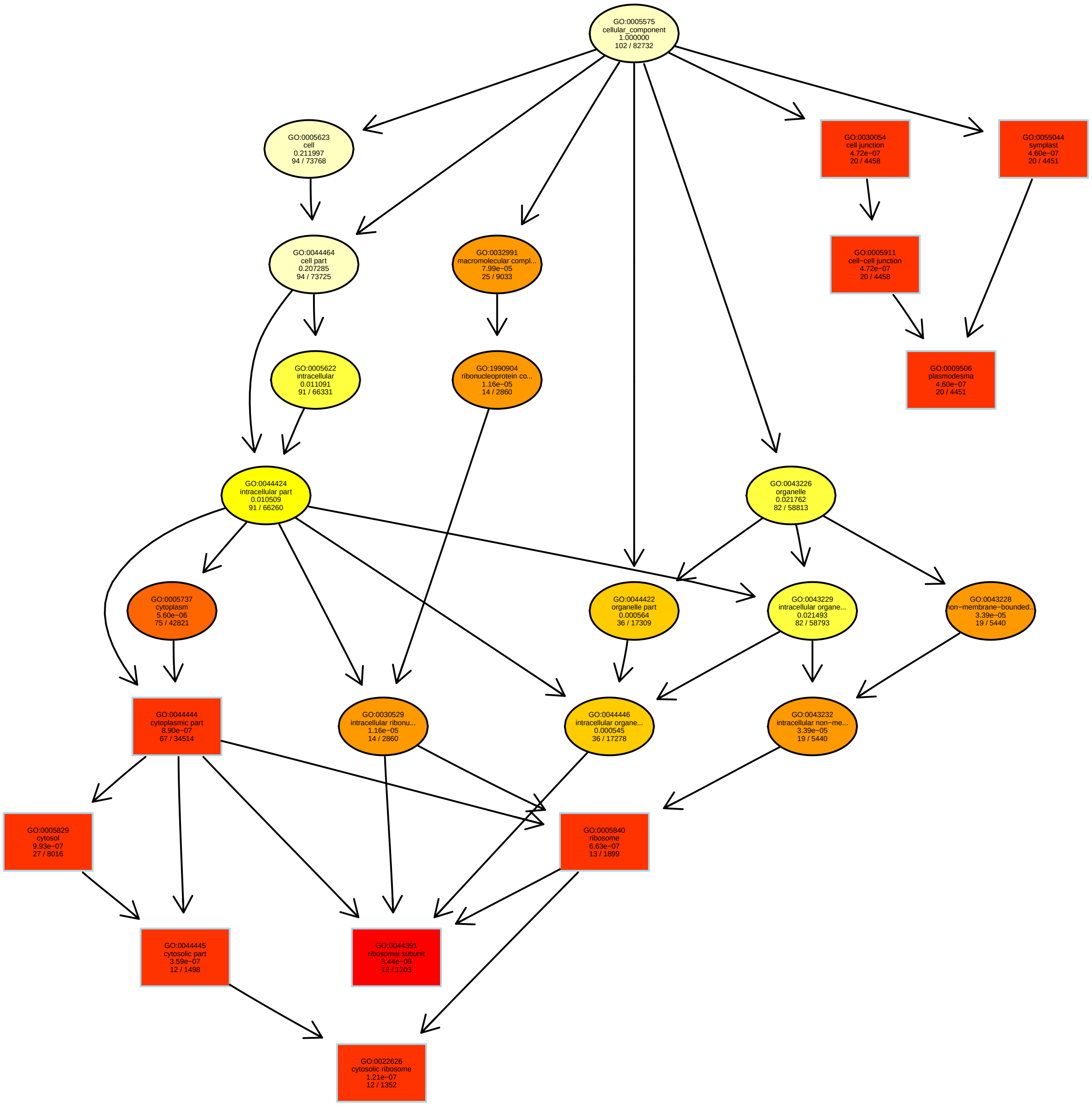
GO:0005575
cellular_component
1.000000
102 / 82732
GO:0005623
cell
0.211997
94 / 73768
GO:0030054
cell junction
4.72e−07
20 / 4458
GO:0055044
symplast
4.60e−07
20 / 4451
GO:0044464
cell part
0.207285
94 / 73725
GO:0032991
macromolecular compl...
7.99e−05
25 / 9033
GO:0005911
cell−cell junction
4.72e−07
20 / 4458
GO:0005622
intracellular
0.011091
91 / 66331
GO:1990904
ribonucleoprotein co...
1.16e−05
14 / 2860
GO:0009506
plasmodesma
4.60e−07
20 / 4451
GO:0044424
intracellular part
0.010509
91 / 66260
GO:0043226
organelle
0.021762
82 / 58813
GO:0005737
cytoplasm
5.60e−06
75 / 42821
GO:0044422
organelle part
0.000564
36 / 17309
GO:0043229
intracellular organe...
0.021493
82 / 58793
GO:0043228
non−membrane−bounded...
3.39e−05
19 / 5440
GO:0044444
cytoplasmic part
8.90e−07
67 / 34514
GO:0030529
intracellular ribonu...
1.16e−05
14 / 2860
GO:0044446
intracellular organe...
0.000545
36 / 17278
GO:0043232
intracellular non−me...
3.39e−05
19 / 5440
GO:0005829
cytosol
9.93e−07
27 / 8016
GO:0005840
ribosome
6.63e−07
13 / 1899
GO:0044445
cytosolic part
3.59e−07
12 / 1498
GO:0044391
ribosomal subunit
3.44e−08
12 / 1203
GO:0022626
cytosolic ribosome
1.21e−07
12 / 1352
