# Supplementary material for: Differential Alternative Splicing Genes and Isoform Regulation Networks of Rapeseed (Brassica napus L.) Infected with Sclerotinia sclerotiorum
Source: Genes (Basel). 2020 Jul 13;11(7):784. doi: 10.3390/genes11070784 (PMC7397149; doi:10.3390/genes11070784)
Supplement: Supplementary file 1 [file genes-11-00784-s001.zip › Fig. S2 Enriched GO terms of genes in the 85 WGCNA modules.zip!/Fig. S2/CytoNodecoral1-go.pdf]

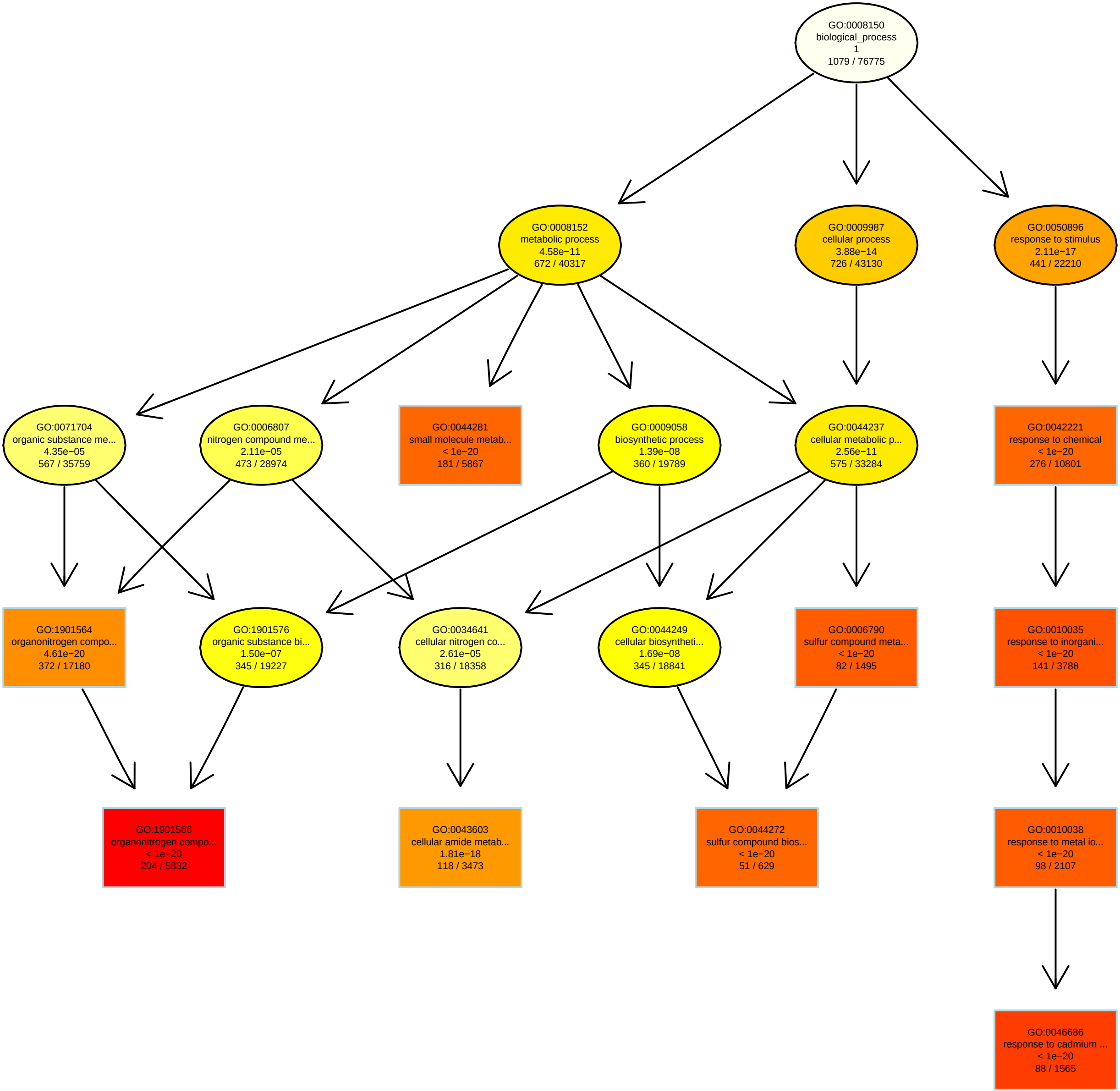
GO:0008150
biological_process
1
1079 / 76775
GO:0008152
metabolic process
4.58e−11
672 / 40317
GO:0009987
cellular process
3.88e−14
726 / 43130
GO:0050896
response to stimulus
2.11e−17
441 / 22210
GO:0071704
organic substance me...
4.35e−05
567 / 35759
GO:0006807
nitrogen compound me...
2.11e−05
473 / 28974
GO:0044281
small molecule metab...
< 1e−20
181 / 5867
GO:0009058
biosynthetic process
1.39e−08
360 / 19789
GO:0044237
cellular metabolic p...
2.56e−11
575 / 33284
GO:0042221
response to chemical
< 1e−20
276 / 10801
GO:1901564
organonitrogen compo...
4.61e−20
372 / 17180
GO:1901576
organic substance bi...
1.50e−07
345 / 19227
GO:0034641
cellular nitrogen co...
2.61e−05
316 / 18358
GO:0044249
cellular biosyntheti...
1.69e−08
345 / 18841
GO:0006790
sulfur compound meta...
< 1e−20
82 / 1495
GO:0010035
response to inorgani...
< 1e−20
141 / 3788
GO:1901566
organonitrogen compo...
< 1e−20
204 / 5832
GO:0043603
cellular amide metab...
1.81e−18
118 / 3473
GO:0044272
sulfur compound bios...
< 1e−20
51 / 629
GO:0010038
response to metal io...
< 1e−20
98 / 2107
GO:0046686
response to cadmium ...
< 1e−20
88 / 1565

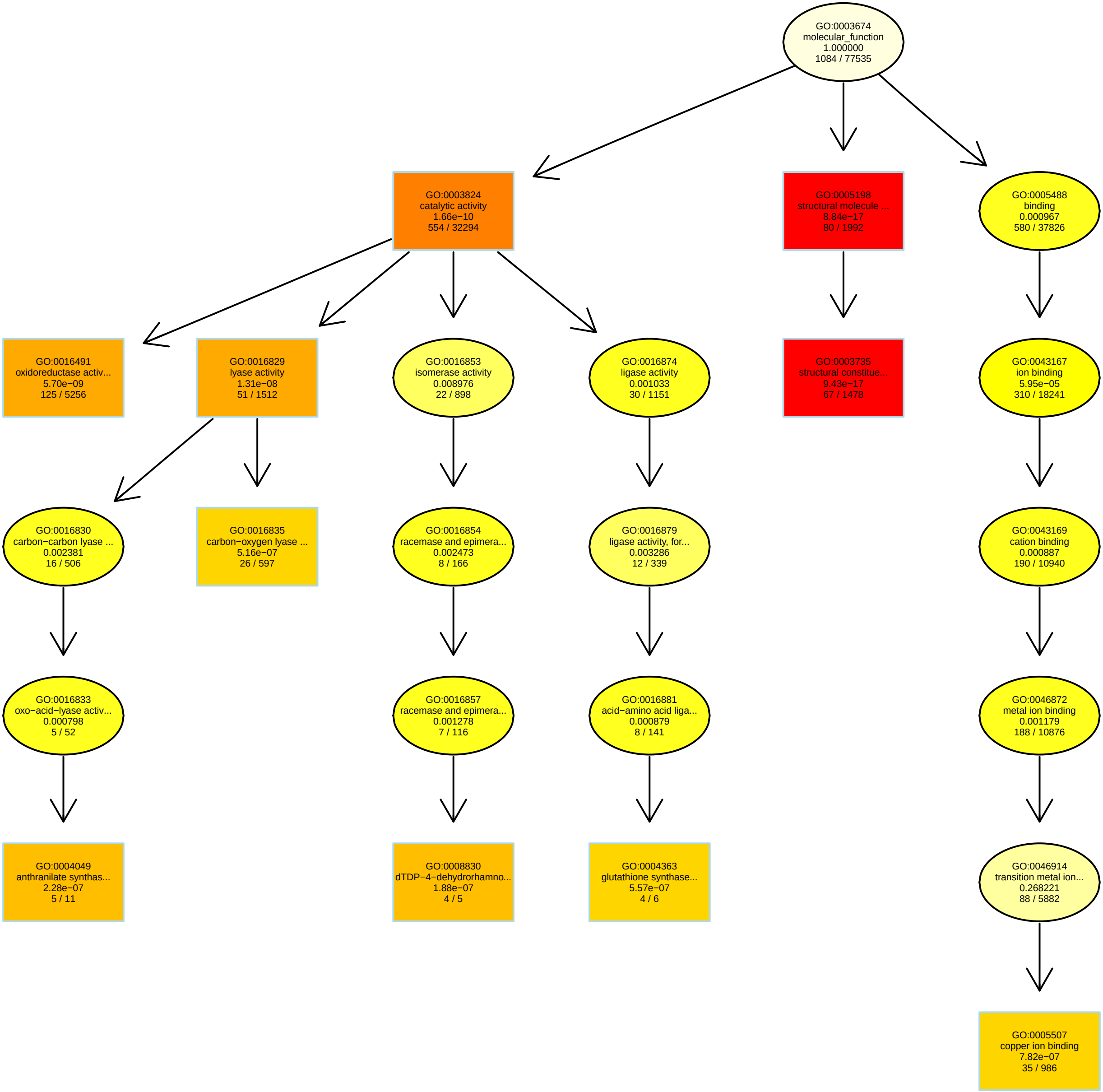
GO:0003674
molecular_function
1.000000
1084 / 77535
GO:0003824
catalytic activity
1.66e−10
554 / 32294
GO:0005198
structural molecule ...
8.84e−17
80 / 1992
GO:0005488
binding
0.000967
580 / 37826
GO:0016491
oxidoreductase activ...
5.70e−09
125 / 5256
GO:0016829
lyase activity
1.31e−08
51 / 1512
GO:0016853
isomerase activity
0.008976
22 / 898
GO:0016874
ligase activity
0.001033
30 / 1151
GO:0003735
structural constitue...
9.43e−17
67 / 1478
GO:0043167
ion binding
5.95e−05
310 / 18241
GO:0016830
carbon−carbon lyase ...
0.002381
16 / 506
GO:0016835
carbon−oxygen lyase ...
5.16e−07
26 / 597
GO:0016854
racemase and epimera...
0.002473
8 / 166
GO:0016879
ligase activity, for...
0.003286
12 / 339
GO:0043169
cation binding
0.000887
190 / 10940
GO:0016833
oxo−acid−lyase activ...
0.000798
5 / 52
GO:0016857
racemase and epimera...
0.001278
7 / 116
GO:0016881
acid−amino acid liga...
0.000879
8 / 141
GO:0046872
metal ion binding
0.001179
188 / 10876
GO:0004049
anthranilate synthas...
2.28e−07
5 / 11
GO:0008830
dTDP−4−dehydrorhamno...
1.88e−07
4 / 5
GO:0004363
glutathione synthase...
5.57e−07
4 / 6
GO:0046914
transition metal ion...
0.268221
88 / 5882
GO:0005507
copper ion binding
7.82e−07
35 / 986

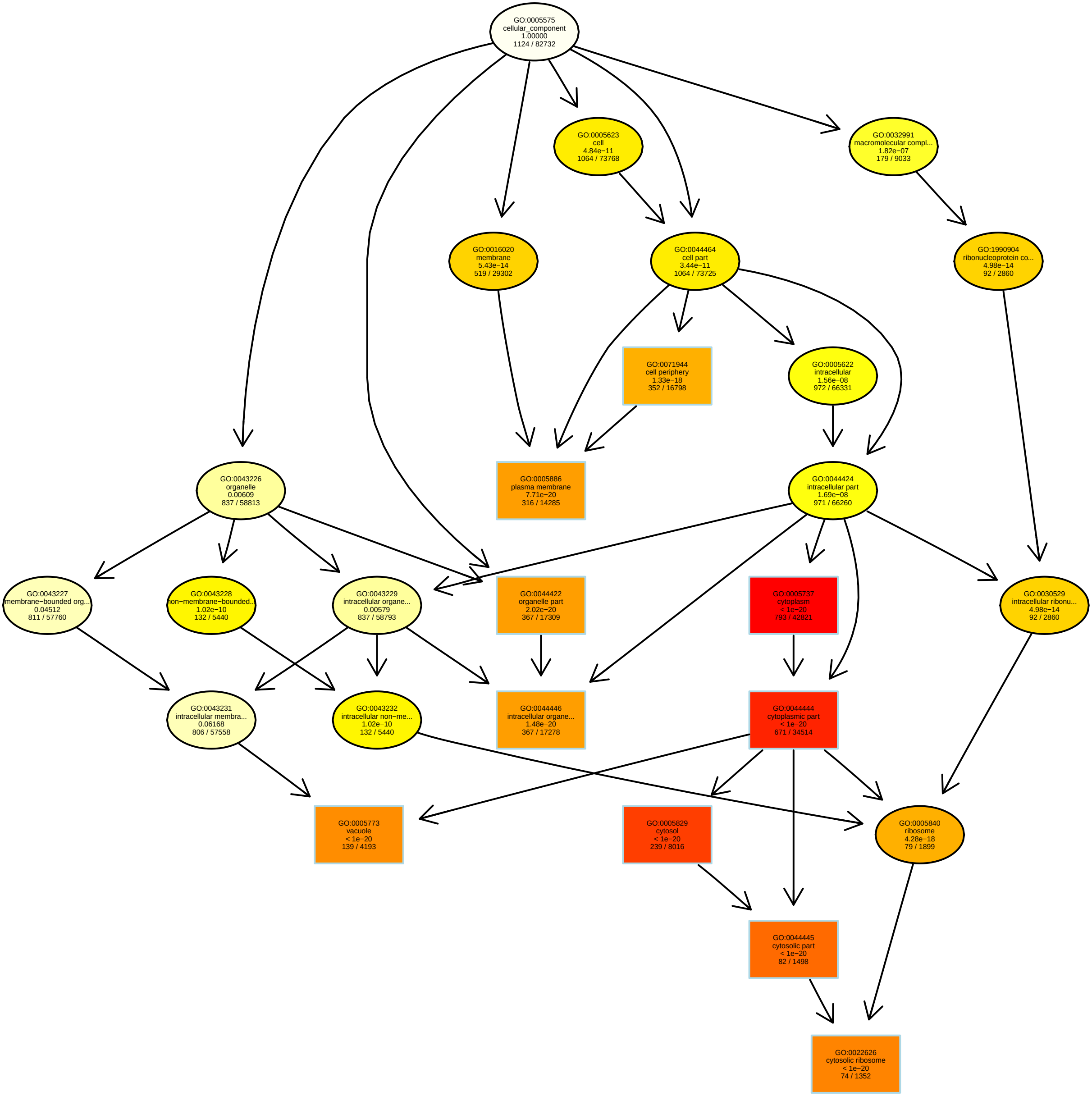
GO:0005575
cellular_component
1.00000
1124 / 82732
GO:0005623
cell
4.84e−11
1064 / 73768
GO:0032991
macromolecular compl...
1.82e−07
179 / 9033
GO:0016020
membrane
5.43e−14
519 / 29302
GO:0044464
cell part
3.44e−11
1064 / 73725
GO:1990904
ribonucleoprotein co...
4.98e−14
92 / 2860
GO:0071944
cell periphery
1.33e−18
352 / 16798
GO:0005622
intracellular
1.56e−08
972 / 66331
GO:0043226
organelle
0.00609
837 / 58813
GO:0005886
plasma membrane
7.71e−20
316 / 14285
GO:0044424
intracellular part
1.69e−08
971 / 66260
GO:0043227
membrane−bounded org...
0.04512
811 / 57760
GO:0043228
non−membrane−bounded...
1.02e−10
132 / 5440
GO:0043229
intracellular organe...
0.00579
837 / 58793
GO:0044422
organelle part
2.02e−20
367 / 17309
GO:0005737
cytoplasm
< 1e−20
793 / 42821
GO:0030529
intracellular ribonu...
4.98e−14
92 / 2860
GO:0043231
intracellular membra...
0.06168
806 / 57558
GO:0043232
intracellular non−me...
1.02e−10
132 / 5440
GO:0044446
intracellular organe...
1.48e−20
367 / 17278
GO:0044444
cytoplasmic part
< 1e−20
671 / 34514
GO:0005773
vacuole
< 1e−20
139 / 4193
GO:0005829
cytosol
< 1e−20
239 / 8016
GO:0005840
ribosome
4.28e−18
79 / 1899
GO:0044445
cytosolic part
< 1e−20
82 / 1498
GO:0022626
cytosolic ribosome
< 1e−20
74 / 1352
