# Supplementary material for: Differential Alternative Splicing Genes and Isoform Regulation Networks of Rapeseed (Brassica napus L.) Infected with Sclerotinia sclerotiorum
Source: Genes (Basel). 2020 Jul 13;11(7):784. doi: 10.3390/genes11070784 (PMC7397149; doi:10.3390/genes11070784)
Supplement: Supplementary file 1 [file genes-11-00784-s001.zip › Fig. S2 Enriched GO terms of genes in the 85 WGCNA modules.zip!/Fig. S2/CytoNodecoral2-go.pdf]

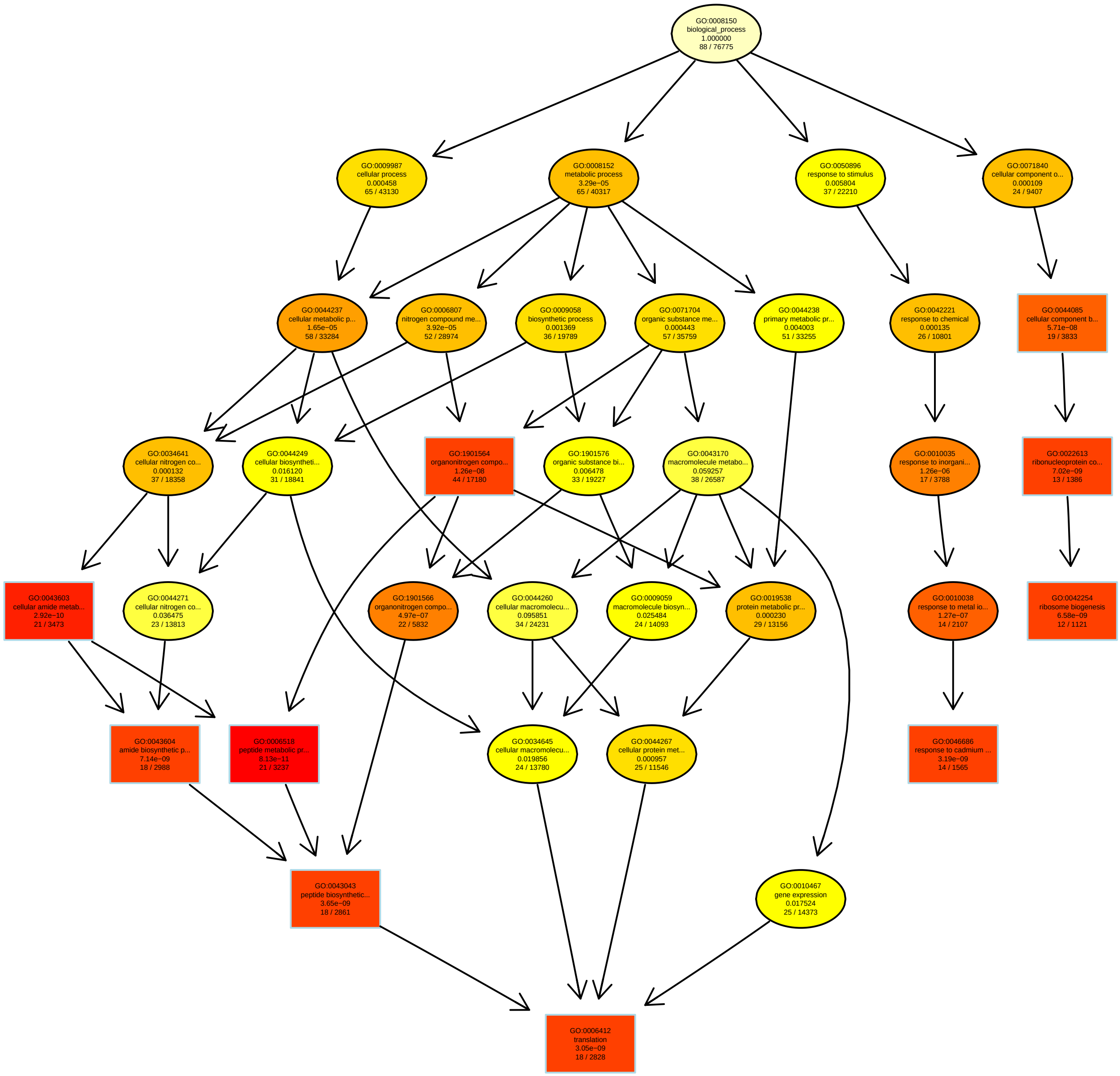

GO:0008150 biological_process 1.000000 88 / 76775
GO:0009987 cellular process 0.000458 65 / 43130
GO:0008152 metabolic process 3.29e−05 65 / 40317
GO:0050896 response to stimulus 0.005804 37 / 22210
GO:0071840 cellular component o... 0.000109 24 / 9407
GO:0044237 cellular metabolic p... 1.65e−05 58 / 33284
GO:0006807 nitrogen compound me... 3.92e−05 52 / 28974
GO:0009058 biosynthetic process 0.001369 36 / 19789
GO:0071704 organic substance me... 0.000443 57 / 35759
GO:0044238 primary metabolic pr... 0.004003 51 / 33255
GO:0042221 response to chemical 0.000135 26 / 10801
GO:0044085 cellular component b... 5.71e−08 19 / 3833
GO:0034641 cellular nitrogen co... 0.000132 37 / 18358
GO:0044249 cellular biosyntheti... 0.016120 31 / 18841
GO:1901564 organonitrogen compo... 1.26e−08 44 / 17180
GO:1901576 organic substance bi... 0.006478 33 / 19227
GO:0043170 macromolecule metabo... 0.059257 38 / 26587
GO:0010035 response to inorgani... 1.26e−06 17 / 3788
GO:0022613 ribonucleoprotein co... 7.02e−09 13 / 1386
GO:0043603 cellular amide metab... 2.92e−10 21 / 3473
GO:0044271 cellular nitrogen co... 0.036475 23 / 13813
GO:1901566 organonitrogen compo... 4.97e−07 22 / 5832
GO:0044260 cellular macromolecu... 0.095851 34 / 24231
GO:0009059 macromolecule biosyn... 0.025484 24 / 14093
GO:0019538 protein metabolic pr... 0.000230 29 / 13156
GO:0010038 response to metal io... 1.27e−07 14 / 2107
GO:0042254 ribosome biogenesis 6.58e−09 12 / 1121
GO:0043604 amide biosynthetic p... 7.14e−09 18 / 2988
GO:0006518 peptide metabolic pr... 8.13e−11 21 / 3237
GO:0034645 cellular macromolecu... 0.019856 24 / 13780
GO:0044267 cellular protein met... 0.000957 25 / 11546
GO:0046686 response to cadmium ... 3.19e−09 14 / 1565
GO:0043043 peptide biosynthetic... 3.65e−09 18 / 2861
GO:0010467 gene expression 0.017524 25 / 14373
GO:0006412 translation 3.05e−09 18 / 2828

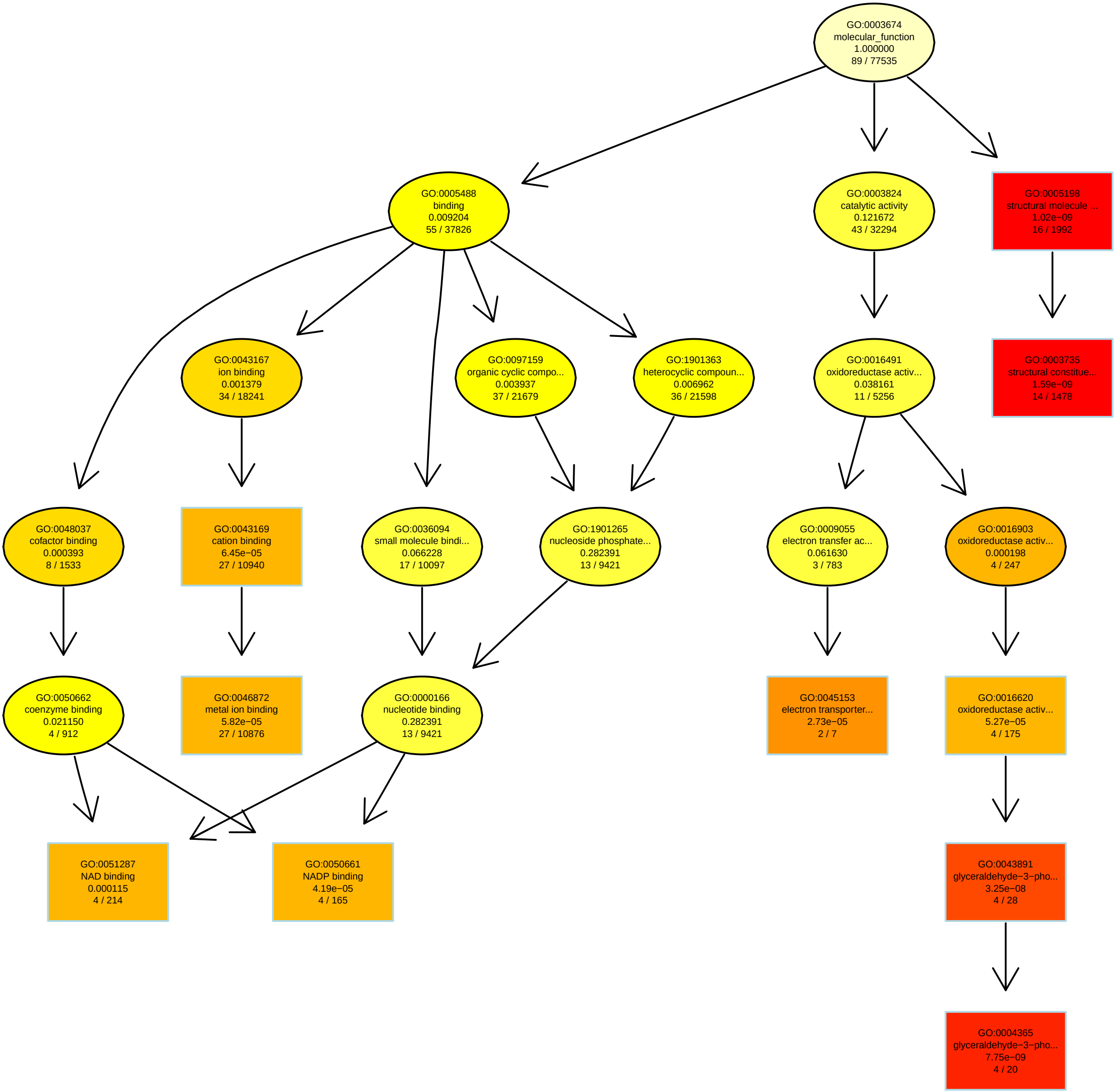

GO:0003674
molecular_function
1.000000
89 / 77535
GO:0005488
binding
0.009204
55 / 37826
GO:0003824
catalytic activity
0.121672
43 / 32294
GO:0005198
structural molecule ...
1.02e−09
16 / 1992
GO:0043167
ion binding
0.001379
34 / 18241
GO:0097159
organic cyclic compo...
0.003937
37 / 21679
GO:1901363
heterocyclic compoun...
0.006962
36 / 21598
GO:0016491
oxidoreductase activ...
0.038161
11 / 5256
GO:0003735
structural constitue...
1.59e−09
14 / 1478
GO:0048037
cofactor binding
0.000393
8 / 1533
GO:0043169
cation binding
6.45e−05
27 / 10940
GO:0036094
small molecule bindi...
0.066228
17 / 10097
GO:1901265
nucleoside phosphate...
0.282391
13 / 9421
GO:0009055
electron transfer ac...
0.061630
3 / 783
GO:0016903
oxidoreductase activ...
0.000198
4 / 247
GO:0050662
coenzyme binding
0.021150
4 / 912
GO:0046872
metal ion binding
5.82e−05
27 / 10876
GO:0000166
nucleotide binding
0.282391
13 / 9421
GO:0045153
electron transporter...
2.73e−05
2 / 7
GO:0016620
oxidoreductase activ...
5.27e−05
4 / 175
GO:0051287
NAD binding
0.000115
4 / 214
GO:0050661
NADP binding
4.19e−05
4 / 165
GO:0043891
glyceraldehyde−3−pho...
3.25e−08
4 / 28
GO:0004365
glyceraldehyde−3−pho...
7.75e−09
4 / 20

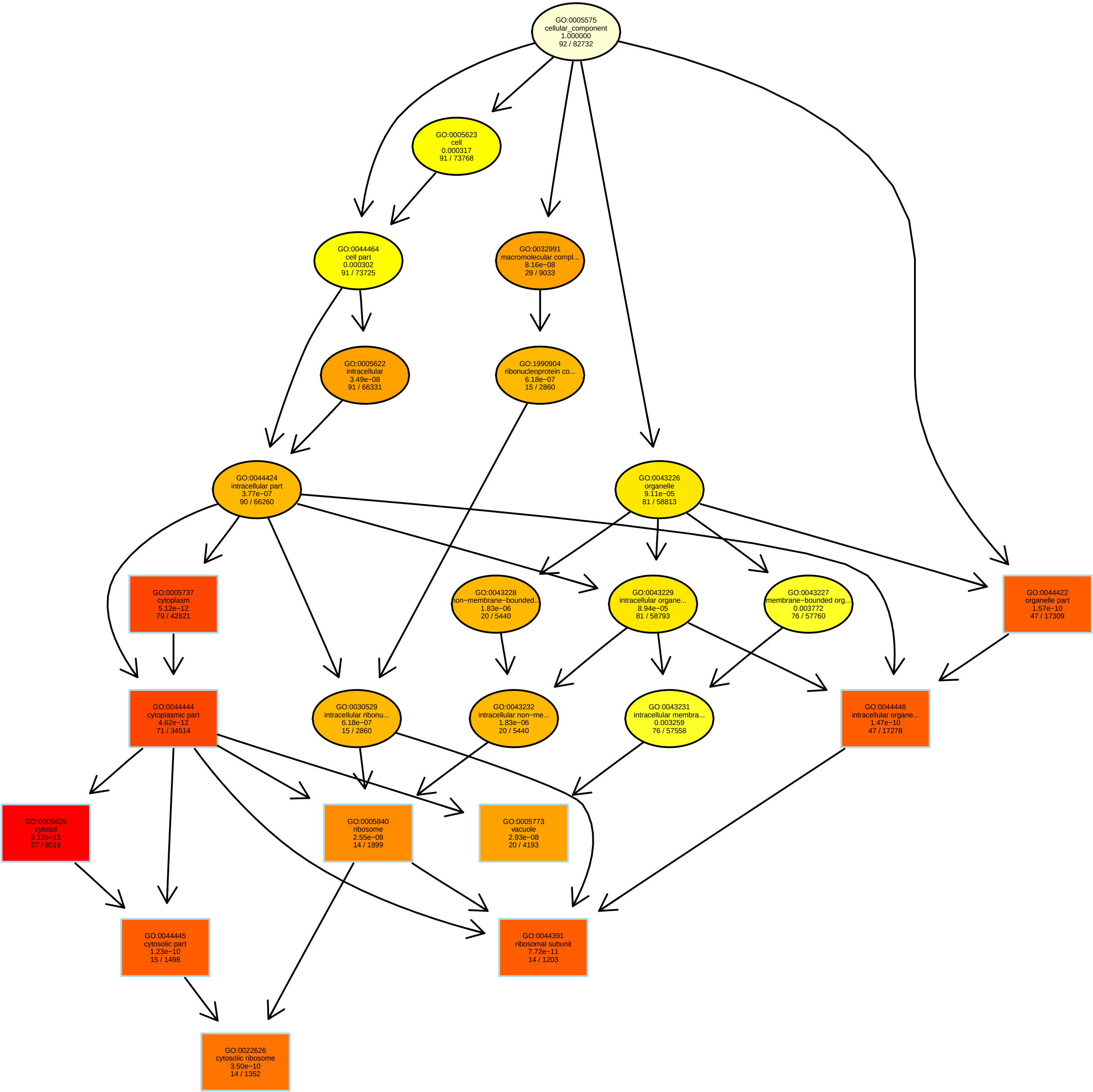
GO:0005575
cellular_component
1.000000
92 / 82732
GO:0005623
cell
0.000317
91 / 73768
GO:0044464
cell part
0.000302
91 / 73725
GO:0032991
macromolecular compl...
8.16e−08
29 / 9033
GO:0005622
intracellular
3.49e−08
91 / 66331
GO:1990904
ribonucleoprotein co...
6.18e−07
15 / 2860
GO:0044424
intracellular part
3.77e−07
90 / 66260
GO:0043226
organelle
9.11e−05
81 / 58813
GO:0005737
cytoplasm
5.12e−12
79 / 42821
GO:0043228
non−membrane−bounded...
1.83e−06
20 / 5440
GO:0043229
intracellular organe...
8.94e−05
81 / 58793
GO:0043227
membrane−bounded org...
0.003772
76 / 57760
GO:0044422
organelle part
1.57e−10
47 / 17309
GO:0044444
cytoplasmic part
4.62e−12
71 / 34514
GO:0030529
intracellular ribonu...
6.18e−07
15 / 2860
GO:0043232
intracellular non−me...
1.83e−06
20 / 5440
GO:0043231
intracellular membra...
0.003259
76 / 57558
GO:0044446
intracellular organe...
1.47e−10
47 / 17278
GO:0005829
cytosol
9.12e−15
37 / 8016
GO:0005840
ribosome
2.55e−08
14 / 1899
GO:0005773
vacuole
2.93e−08
20 / 4193
GO:0044445
cytosolic part
1.23e−10
15 / 1498
GO:0044391
ribosomal subunit
7.72e−11
14 / 1203
GO:0022626
cytosolic ribosome
3.50e−10
14 / 1352
