# Supplementary material for: Differential Alternative Splicing Genes and Isoform Regulation Networks of Rapeseed (Brassica napus L.) Infected with Sclerotinia sclerotiorum
Source: Genes (Basel). 2020 Jul 13;11(7):784. doi: 10.3390/genes11070784 (PMC7397149; doi:10.3390/genes11070784)
Supplement: Supplementary file 1 [file genes-11-00784-s001.zip › Fig. S2 Enriched GO terms of genes in the 85 WGCNA modules.zip!/Fig. S2/CytoNodecyan-go.pdf]

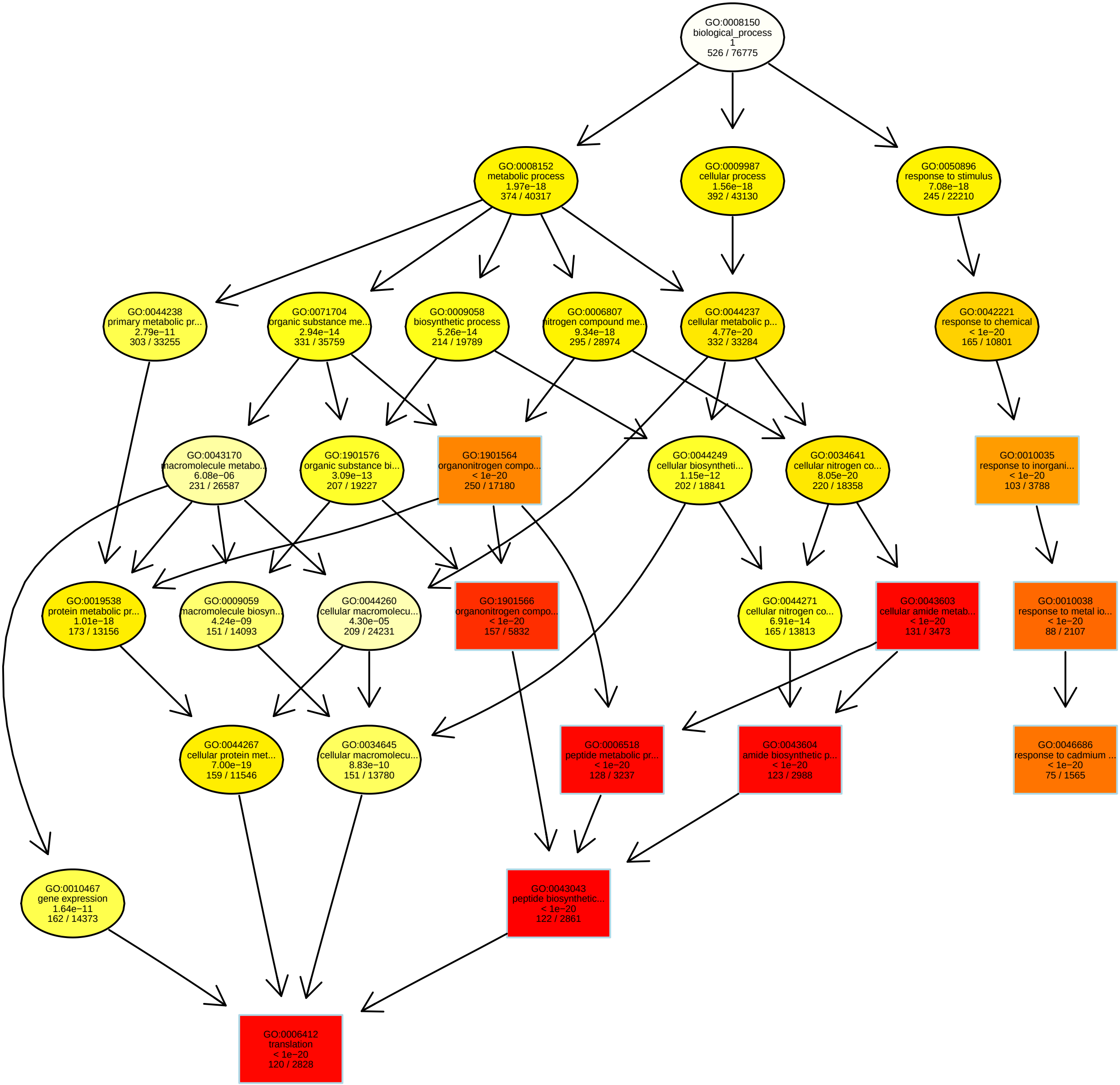

GO:0008150
biological_process
1
526 / 76775
GO:0008152
metabolic process
1.97e−18
374 / 40317
GO:0009987
cellular process
1.56e−18
392 / 43130
GO:0050896
response to stimulus
7.08e−18
245 / 22210
GO:0044238
primary metabolic pr...
2.79e−11
303 / 33255
GO:0071704
organic substance me...
2.94e−14
331 / 35759
GO:0009058
biosynthetic process
5.26e−14
214 / 19789
GO:0006807
nitrogen compound me...
9.34e−18
295 / 28974
GO:0044237
cellular metabolic p...
4.77e−20
332 / 33284
GO:0042221
response to chemical
< 1e−20
165 / 10801
GO:0043170
macromolecule metabo...
6.08e−06
231 / 26587
GO:1901576
organic substance bi...
3.09e−13
207 / 19227
GO:1901564
organonitrogen compo...
< 1e−20
250 / 17180
GO:0044249
cellular biosyntheti...
1.15e−12
202 / 18841
GO:0034641
cellular nitrogen co...
8.05e−20
220 / 18358
GO:0010035
response to inorgani...
< 1e−20
103 / 3788
GO:0019538
protein metabolic pr...
1.01e−18
173 / 13156
GO:0009059
macromolecule biosyn...
4.24e−09
151 / 14093
GO:0044260
cellular macromolecu...
4.30e−05
209 / 24231
GO:1901566
organonitrogen compo...
< 1e−20
157 / 5832
GO:0044271
cellular nitrogen co...
6.91e−14
165 / 13813
GO:0043603
cellular amide metab...
< 1e−20
131 / 3473
GO:0010038
response to metal io...
< 1e−20
88 / 2107
GO:0044267
cellular protein met...
7.00e−19
159 / 11546
GO:0034645
cellular macromolecu...
8.83e−10
151 / 13780
GO:0006518
peptide metabolic pr...
< 1e−20
128 / 3237
GO:0043604
amide biosynthetic p...
< 1e−20
123 / 2988
GO:0046686
response to cadmium ...
< 1e−20
75 / 1565
GO:0010467
gene expression
1.64e−11
162 / 14373
GO:0043043
peptide biosynthetic...
< 1e−20
122 / 2861
GO:0006412
translation
< 1e−20
120 / 2828

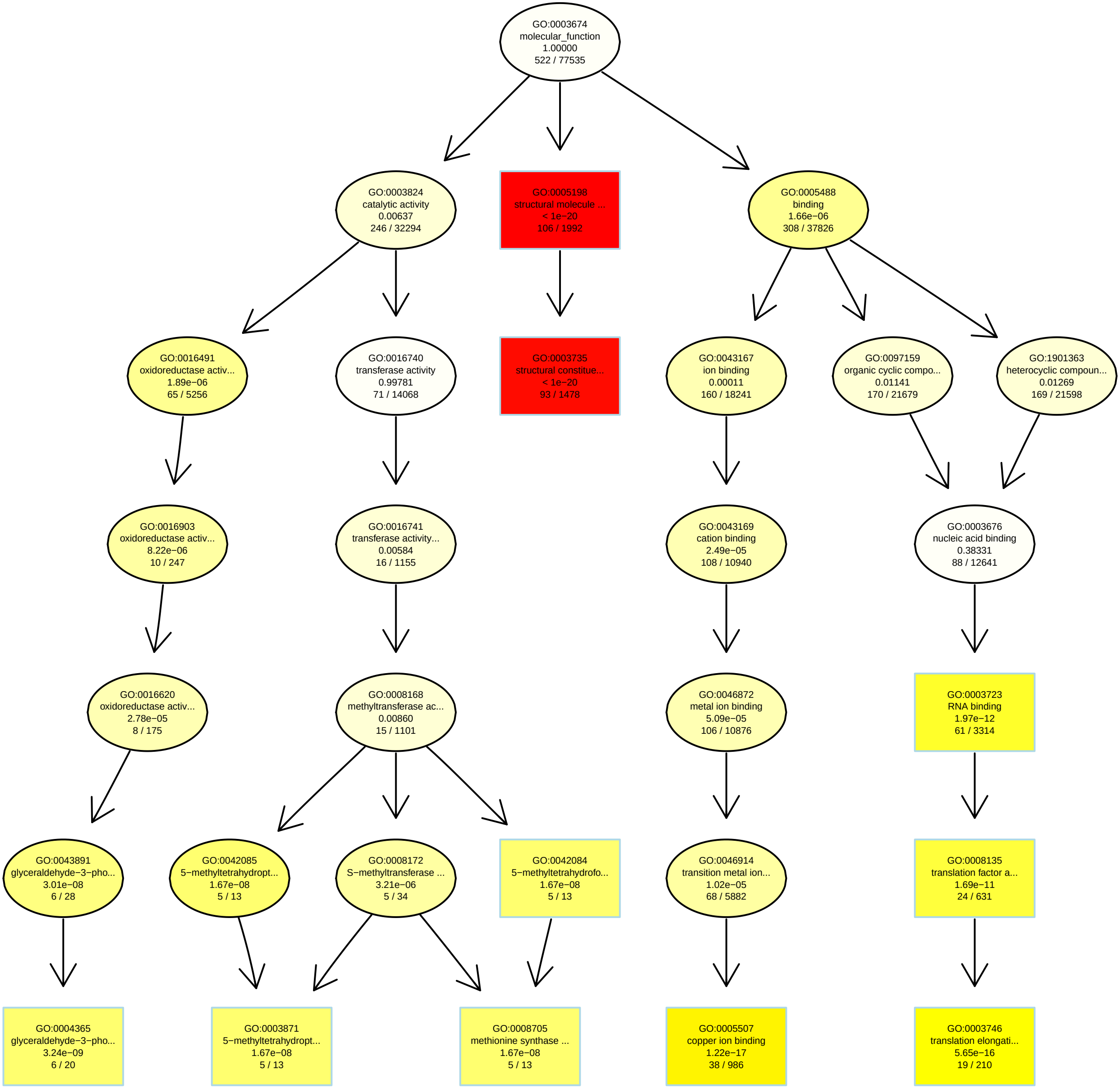
GO:0003674
molecular_function
1.00000
522 / 77535
GO:0003824
catalytic activity
0.00637
246 / 32294
GO:0005198
structural molecule ...
< 1e−20
106 / 1992
GO:0005488
binding
1.66e−06
308 / 37826
GO:0016491
oxidoreductase activ...
1.89e−06
65 / 5256
GO:0016740
transferase activity
0.99781
71 / 14068
GO:0003735
structural constitue...
< 1e−20
93 / 1478
GO:0043167
ion binding
0.00011
160 / 18241
GO:0097159
organic cyclic compo...
0.01141
170 / 21679
GO:1901363
heterocyclic compoun...
0.01269
169 / 21598
GO:0016903
oxidoreductase activ...
8.22e−06
10 / 247
GO:0016741
transferase activity...
0.00584
16 / 1155
GO:0043169
cation binding
2.49e−05
108 / 10940
GO:0003676
nucleic acid binding
0.38331
88 / 12641
GO:0016620
oxidoreductase activ...
2.78e−05
8 / 175
GO:0008168
methyltransferase ac...
0.00860
15 / 1101
GO:0046872
metal ion binding
5.09e−05
106 / 10876
GO:0003723
RNA binding
1.97e−12
61 / 3314
GO:0043891
glyceraldehyde−3−pho...
3.01e−08
6 / 28
GO:0042085
5−methyltetrahydropt...
1.67e−08
5 / 13
GO:0008172
S−methyltransferase ...
3.21e−06
5 / 34
GO:0042084
5−methyltetrahydrofo...
1.67e−08
5 / 13
GO:0046914
transition metal ion...
1.02e−05
68 / 5882
GO:0008135
translation factor a...
1.69e−11
24 / 631
GO:0004365
glyceraldehyde−3−pho...
3.24e−09
6 / 20
GO:0003871
5−methyltetrahydropt...
1.67e−08
5 / 13
GO:0008705
methionine synthase ...
1.67e−08
5 / 13
GO:0005507
copper ion binding
1.22e−17
38 / 986
GO:0003746
translation elongati...
5.65e−16
19 / 210

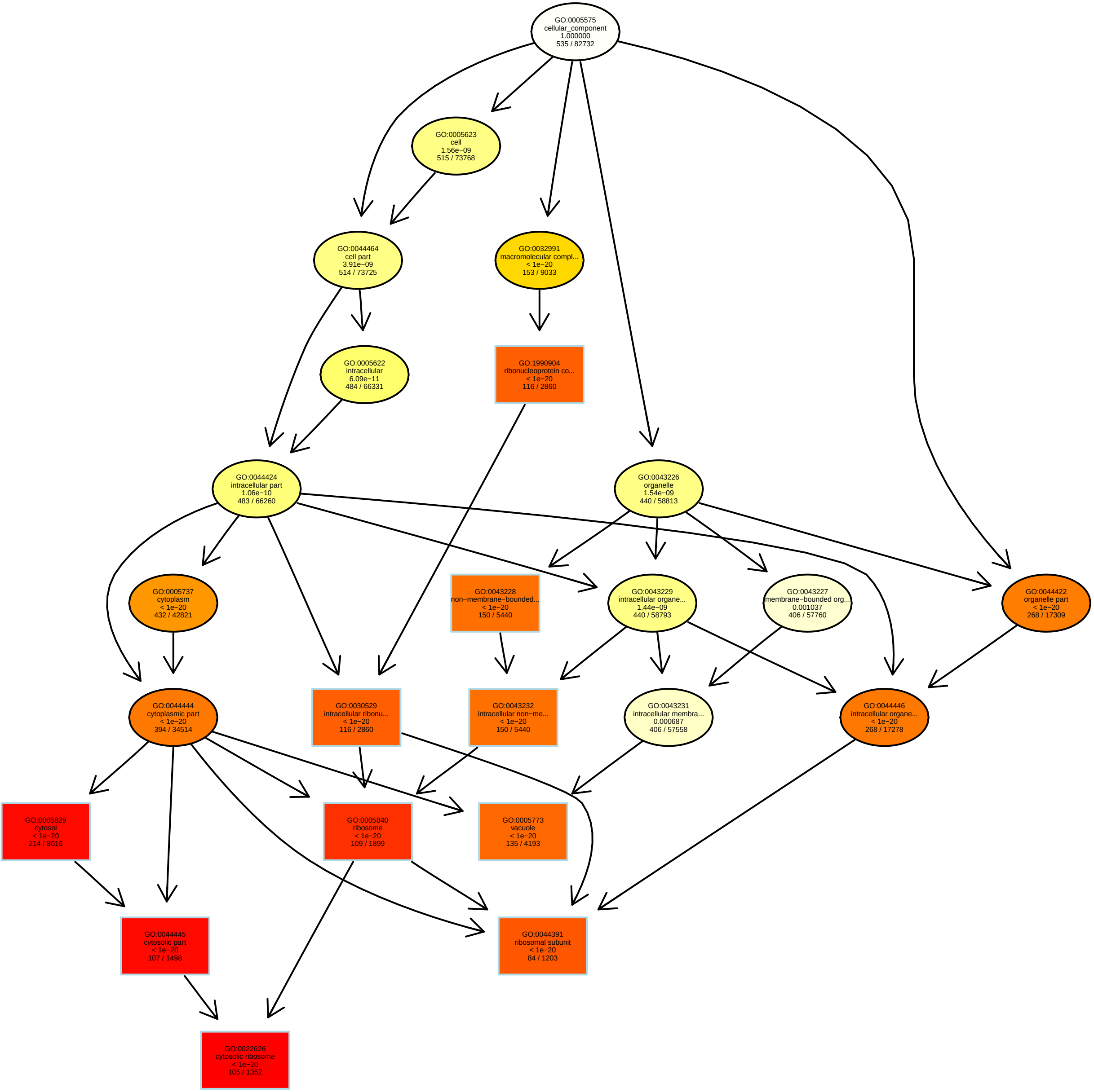
GO:0005575
cellular_component
1.000000
535 / 82732
GO:0005623
cell
1.56e−09
515 / 73768
GO:0044464
cell part
3.91e−09
514 / 73725
GO:0032991
macromolecular compl...
< 1e−20
153 / 9033
GO:0005622
intracellular
6.09e−11
484 / 66331
GO:1990904
ribonucleoprotein co...
< 1e−20
116 / 2860
GO:0044424
intracellular part
1.06e−10
483 / 66260
GO:0043226
organelle
1.54e−09
440 / 58813
GO:0005737
cytoplasm
< 1e−20
432 / 42821
GO:0043228
non−membrane−bounded...
< 1e−20
150 / 5440
GO:0043229
intracellular organe...
1.44e−09
440 / 58793
GO:0043227
membrane−bounded org...
0.001037
406 / 57760
GO:0044422
organelle part
< 1e−20
268 / 17309
GO:0044444
cytoplasmic part
< 1e−20
394 / 34514
GO:0030529
intracellular ribonu...
< 1e−20
116 / 2860
GO:0043232
intracellular non−me...
< 1e−20
150 / 5440
GO:0043231
intracellular membra...
0.000687
406 / 57558
GO:0044446
intracellular organe...
< 1e−20
268 / 17278
GO:0005829
cytosol
< 1e−20
214 / 8016
GO:0005840
ribosome
< 1e−20
109 / 1899
GO:0005773
vacuole
< 1e−20
135 / 4193
GO:0044445
cytosolic part
< 1e−20
107 / 1498
GO:0044391
ribosomal subunit
< 1e−20
84 / 1203
GO:0022626
cytosolic ribosome
< 1e−20
105 / 1352
