# Supplementary material for: Differential Alternative Splicing Genes and Isoform Regulation Networks of Rapeseed (Brassica napus L.) Infected with Sclerotinia sclerotiorum
Source: Genes (Basel). 2020 Jul 13;11(7):784. doi: 10.3390/genes11070784 (PMC7397149; doi:10.3390/genes11070784)
Supplement: Supplementary file 1 [file genes-11-00784-s001.zip › Fig. S2 Enriched GO terms of genes in the 85 WGCNA modules.zip!/Fig. S2/CytoNodedarkgrey-go.pdf]

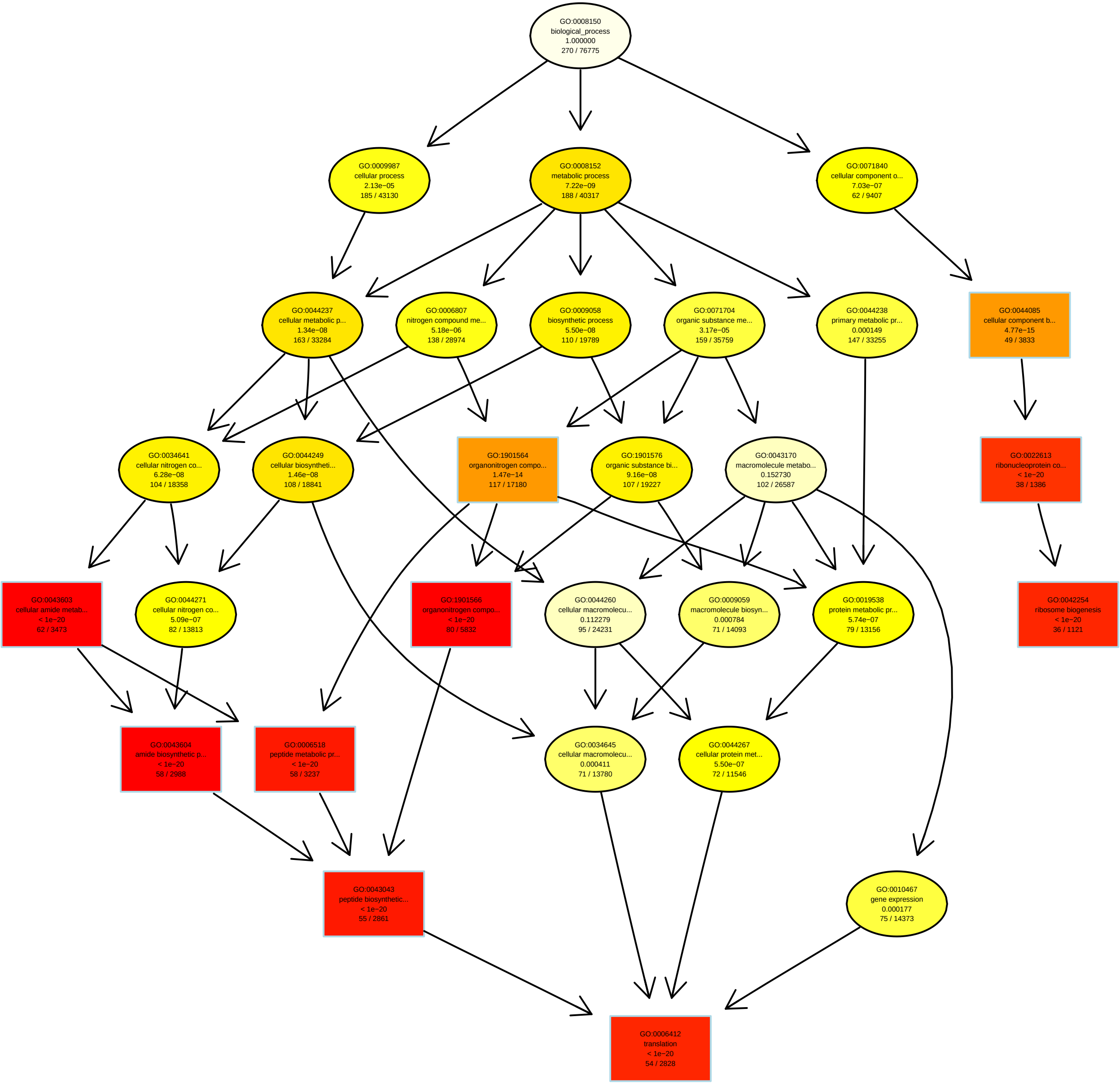

GO:0008150 biological_process 1.000000 270 / 76775
GO:0009987 cellular process 2.13e−05 185 / 43130
GO:0008152 metabolic process 7.22e−09 188 / 40317
GO:0071840 cellular component o... 7.03e−07 62 / 9407
GO:0044237 cellular metabolic p... 1.34e−08 163 / 33284
GO:0006807 nitrogen compound me... 5.18e−06 138 / 28974
GO:0009058 biosynthetic process 5.50e−08 110 / 19789
GO:0071704 organic substance me... 3.17e−05 159 / 35759
GO:0044238 primary metabolic pr... 0.000149 147 / 33255
GO:0044085 cellular component b... 4.77e−15 49 / 3833
GO:0034641 cellular nitrogen co... 6.28e−08 104 / 18358
GO:0044249 cellular biosyntheti... 1.46e−08 108 / 18841
GO:1901564 organonitrogen compo... 1.47e−14 117 / 17180
GO:1901576 organic substance bi... 9.16e−08 107 / 19227
GO:0043170 macromolecule metabo... 0.152730 102 / 26587
GO:0022613 ribonucleoprotein co... < 1e−20 38 / 1386
GO:0043603 cellular amide metab... < 1e−20 62 / 3473
GO:0044271 cellular nitrogen co... 5.09e−07 82 / 13813
GO:1901566 organonitrogen compo... < 1e−20 80 / 5832
GO:0044260 cellular macromolecu... 0.112279 95 / 24231
GO:0009059 macromolecule biosyn... 0.000784 71 / 14093
GO:0019538 protein metabolic pr... 5.74e−07 79 / 13156
GO:0042254 ribosome biogenesis < 1e−20 36 / 1121
GO:0043604 amide biosynthetic p... < 1e−20 58 / 2988
GO:0006518 peptide metabolic pr... < 1e−20 58 / 3237
GO:0034645 cellular macromolecu... 0.000411 71 / 13780
GO:0044267 cellular protein met... 5.50e−07 72 / 11546
GO:0043043 peptide biosynthetic... < 1e−20 55 / 2861
GO:0010467 gene expression 0.000177 75 / 14373
GO:0006412 translation < 1e−20 54 / 2828

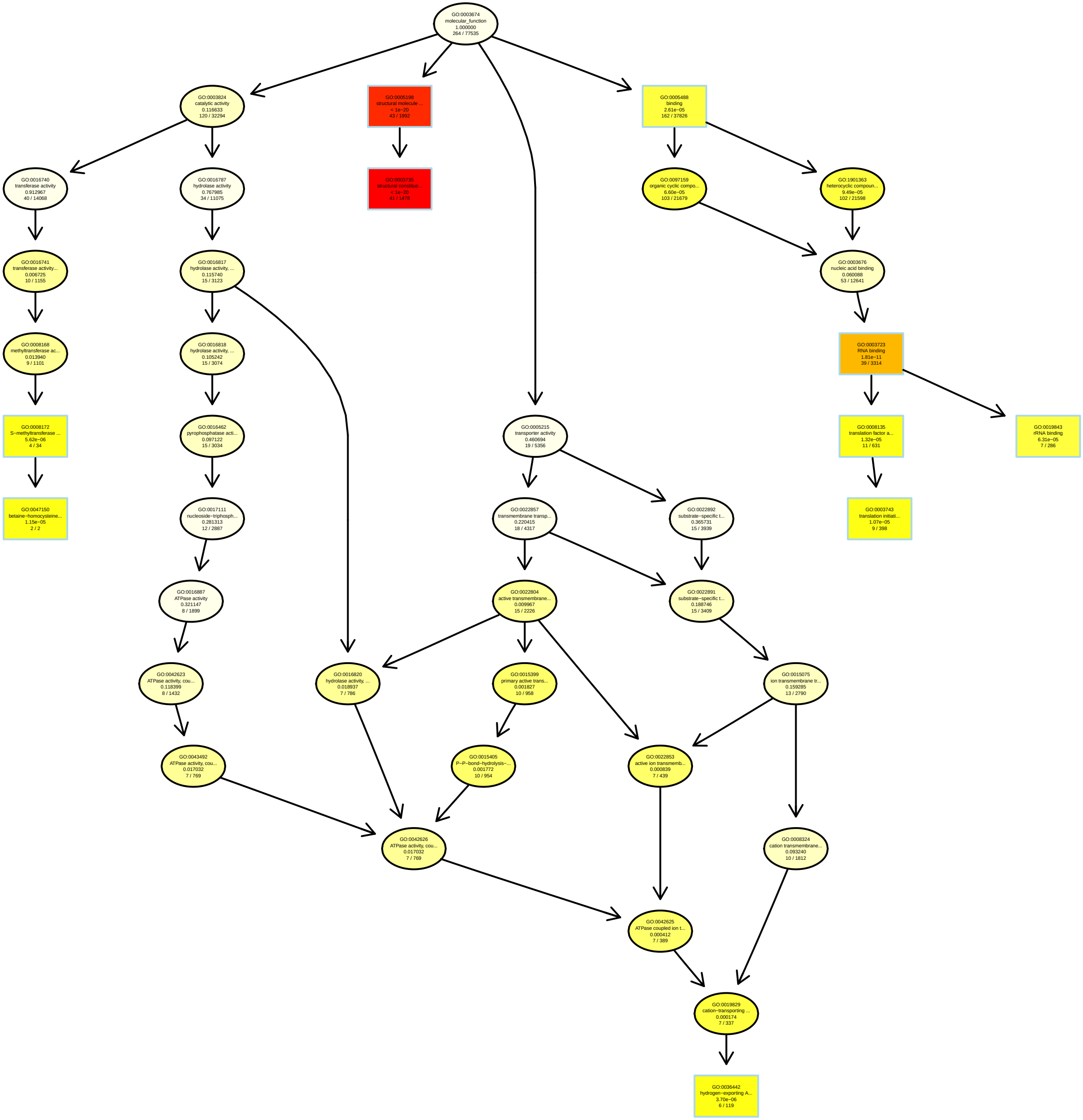
GO:0003674
molecular_function
1.000000
264 / 77535
GO:0003824
catalytic activity
0.116633
120 / 32294
GO:0005198
structural molecule ...
< 1e−20
43 / 1992
GO:0005488
binding
2.61e−05
162 / 37826
GO:0016740
transferase activity
0.912967
40 / 14068
GO:0016787
hydrolase activity
0.767985
34 / 11075
GO:0003735
structural constitue...
< 1e−20
41 / 1478
GO:0097159
organic cyclic compo...
6.60e−05
103 / 21679
GO:1901363
heterocyclic compoun...
9.49e−05
102 / 21598
GO:0016741
transferase activity...
0.006725
10 / 1155
GO:0016817
hydrolase activity, ...
0.115740
15 / 3123
GO:0003676
nucleic acid binding
0.060088
53 / 12641
GO:0008168
methyltransferase ac...
0.013940
9 / 1101
GO:0016818
hydrolase activity, ...
0.105242
15 / 3074
GO:0003723
RNA binding
1.81e−11
39 / 3314
GO:0008172
S−methyltransferase ...
5.62e−06
4 / 34
GO:0016462
pyrophosphatase acti...
0.097122
15 / 3034
GO:0005215
transporter activity
0.460694
19 / 5356
GO:0008135
translation factor a...
1.32e−05
11 / 631
GO:0019843
rRNA binding
6.31e−05
7 / 286
GO:0047150
betaine−homocysteine...
1.15e−05
2 / 2
GO:0017111
nucleoside−triphosph...
0.281313
12 / 2887
GO:0022857
transmembrane transp...
0.220415
18 / 4317
GO:0022892
substrate−specific t...
0.365731
15 / 3939
GO:0003743
translation initiati...
1.07e−05
9 / 398
GO:0016887
ATPase activity
0.321147
8 / 1899
GO:0022804
active transmembrane...
0.009967
15 / 2226
GO:0022891
substrate−specific t...
0.188746
15 / 3409
GO:0042623
ATPase activity, cou...
0.118399
8 / 1432
GO:0016820
hydrolase activity, ...
0.018937
7 / 786
GO:0015399
primary active trans...
0.001827
10 / 958
GO:0015075
ion transmembrane tr...
0.159285
13 / 2790
GO:0043492
ATPase activity, cou...
0.017032
7 / 769
GO:0015405
P−P−bond−hydrolysis−...
0.001772
10 / 954
GO:0022853
active ion transmemb...
0.000839
7 / 439
GO:0042626
ATPase activity, cou...
0.017032
7 / 769
GO:0008324
cation transmembrane...
0.093240
10 / 1812
GO:0042625
ATPase coupled ion t...
0.000412
7 / 389
GO:0019829
cation−transporting ...
0.000174
7 / 337
GO:0036442
hydrogen−exporting A...
3.70e−06
6 / 119

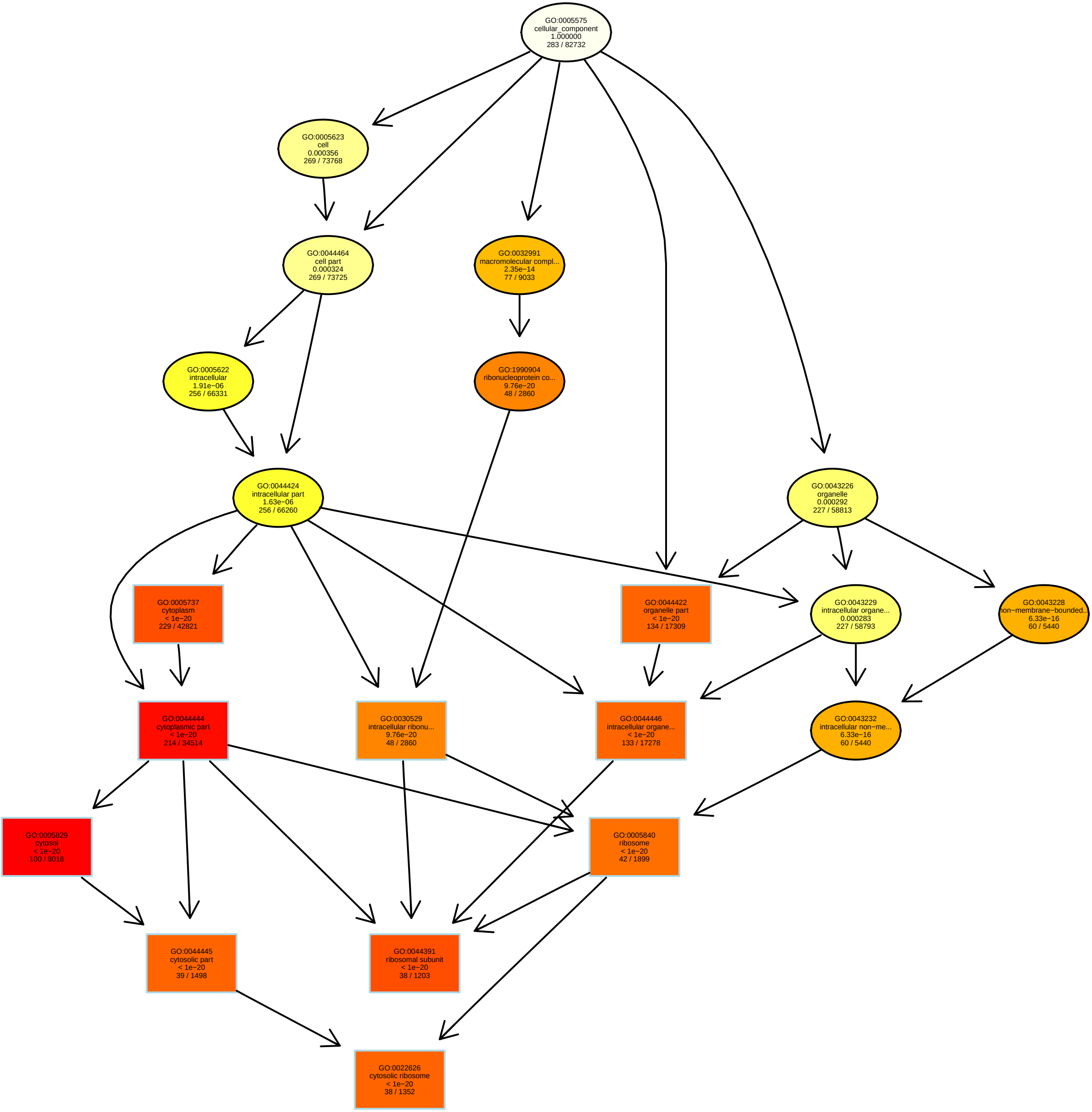

GO:0005575
cellular_component
1.000000
283 / 82732
GO:0005623
cell
0.000356
269 / 73768
GO:0044464
cell part
0.000324
269 / 73725
GO:0032991
macromolecular compl...
2.35e−14
77 / 9033
GO:0005622
intracellular
1.91e−06
256 / 66331
GO:1990904
ribonucleoprotein co...
9.76e−20
48 / 2860
GO:0044424
intracellular part
1.63e−06
256 / 66260
GO:0043226
organelle
0.000292
227 / 58813
GO:0005737
cytoplasm
< 1e−20
229 / 42821
GO:0044422
organelle part
< 1e−20
134 / 17309
GO:0043229
intracellular organe...
0.000283
227 / 58793
GO:0043228
non−membrane−bounded...
6.33e−16
60 / 5440
GO:0044444
cytoplasmic part
< 1e−20
214 / 34514
GO:0030529
intracellular ribonu...
9.76e−20
48 / 2860
GO:0044446
intracellular organe...
< 1e−20
133 / 17278
GO:0043232
intracellular non−me...
6.33e−16
60 / 5440
GO:0005829
cytosol
< 1e−20
100 / 8016
GO:0005840
ribosome
< 1e−20
42 / 1899
GO:0044445
cytosolic part
< 1e−20
39 / 1498
GO:0044391
ribosomal subunit
< 1e−20
38 / 1203
GO:0022626
cytosolic ribosome
< 1e−20
38 / 1352
