# Supplementary material for: Differential Alternative Splicing Genes and Isoform Regulation Networks of Rapeseed (Brassica napus L.) Infected with Sclerotinia sclerotiorum
Source: Genes (Basel). 2020 Jul 13;11(7):784. doi: 10.3390/genes11070784 (PMC7397149; doi:10.3390/genes11070784)
Supplement: Supplementary file 1 [file genes-11-00784-s001.zip › Fig. S2 Enriched GO terms of genes in the 85 WGCNA modules.zip!/Fig. S2/CytoNodedarkolivegreen-go.pdf]

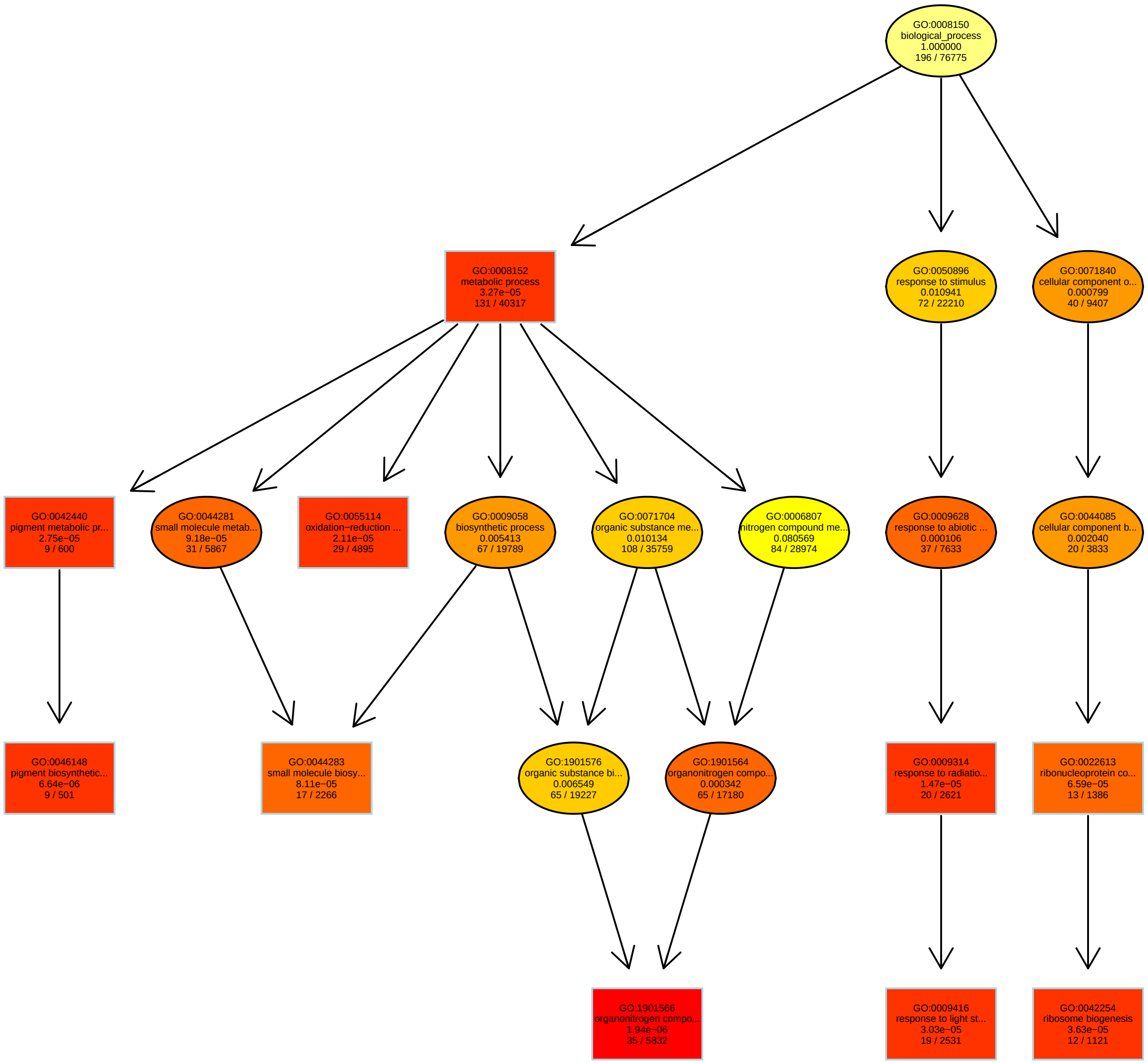
GO:0008150
biological_process
1.000000
196 / 76775
GO:0008152
metabolic process
3.27e−05
131 / 40317
GO:0050896
response to stimulus
0.010941
72 / 22210
GO:0071840
cellular component o...
0.000799
40 / 9407
GO:0042440
pigment metabolic pr...
2.75e−05
9 / 600
GO:0044281
small molecule metab...
9.18e−05
31 / 5867
GO:0055114
oxidation−reduction ...
2.11e−05
29 / 4895
GO:0009058
biosynthetic process
0.005413
67 / 19789
GO:0071704
organic substance me...
0.010134
108 / 35759
GO:0006807
nitrogen compound me...
0.080569
84 / 28974
GO:0009628
response to abiotic ...
0.000106
37 / 7633
GO:0044085
cellular component b...
0.002040
20 / 3833
GO:0046148
pigment biosynthetic...
6.64e−06
9 / 501
GO:0044283
small molecule biosy...
8.11e−05
17 / 2266
GO:1901576
organic substance bi...
0.006549
65 / 19227
GO:1901564
organonitrogen compo...
0.000342
65 / 17180
GO:0009314
response to radiatio...
1.47e−05
20 / 2621
GO:0022613
ribonucleoprotein co...
6.59e−05
13 / 1386
GO:1901566
organonitrogen compo...
1.94e−06
35 / 5832
GO:0009416
response to light st...
3.03e−05
19 / 2531
GO:0042254
ribosome biogenesis
3.63e−05
12 / 1121

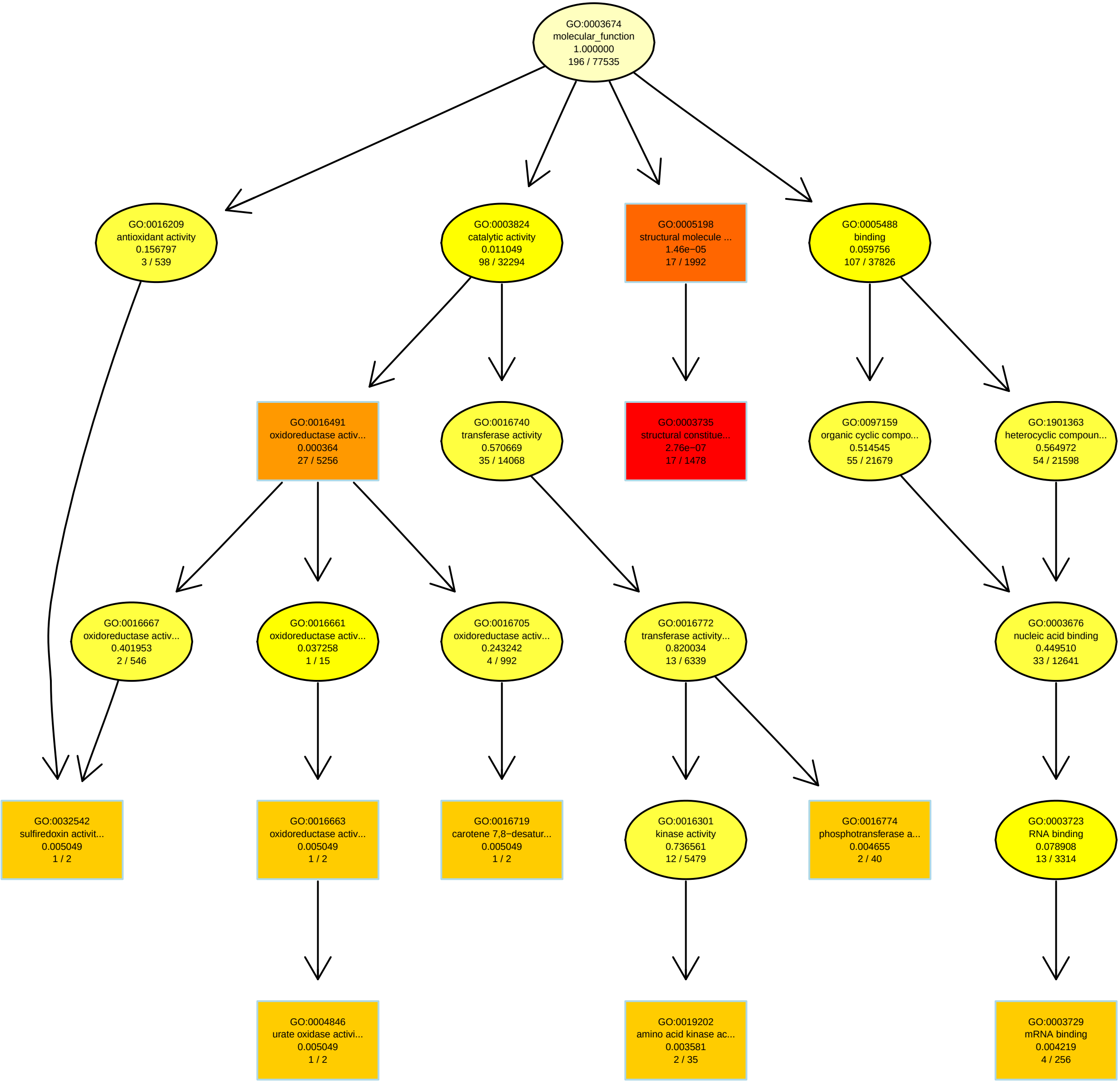
GO:0003674
molecular_function
1.000000
196 / 77535
GO:0016209
antioxidant activity
0.156797
3 / 539
GO:0003824
catalytic activity
0.011049
98 / 32294
GO:0005198
structural molecule ...
1.46e−05
17 / 1992
GO:0005488
binding
0.059756
107 / 37826
GO:0016491
oxidoreductase activ...
0.000364
27 / 5256
GO:0016740
transferase activity
0.570669
35 / 14068
GO:0003735
structural constitue...
2.76e−07
17 / 1478
GO:0097159
organic cyclic compo...
0.514545
55 / 21679
GO:1901363
heterocyclic compoun...
0.564972
54 / 21598
GO:0016667
oxidoreductase activ...
0.401953
2 / 546
GO:0016661
oxidoreductase activ...
0.037258
1 / 15
GO:0016705
oxidoreductase activ...
0.243242
4 / 992
GO:0016772
transferase activity...
0.820034
13 / 6339
GO:0003676
nucleic acid binding
0.449510
33 / 12641
GO:0032542
sulfiredoxin activit...
0.005049
1 / 2
GO:0016663
oxidoreductase activ...
0.005049
1 / 2
GO:0016719
carotene 7,8−desatur...
0.005049
1 / 2
GO:0016301
kinase activity
0.736561
12 / 5479
GO:0016774
phosphotransferase a...
0.004655
2 / 40
GO:0003723
RNA binding
0.078908
13 / 3314
GO:0004846
urate oxidase activi...
0.005049
1 / 2
GO:0019202
amino acid kinase ac...
0.003581
2 / 35
GO:0003729
mRNA binding
0.004219
4 / 256

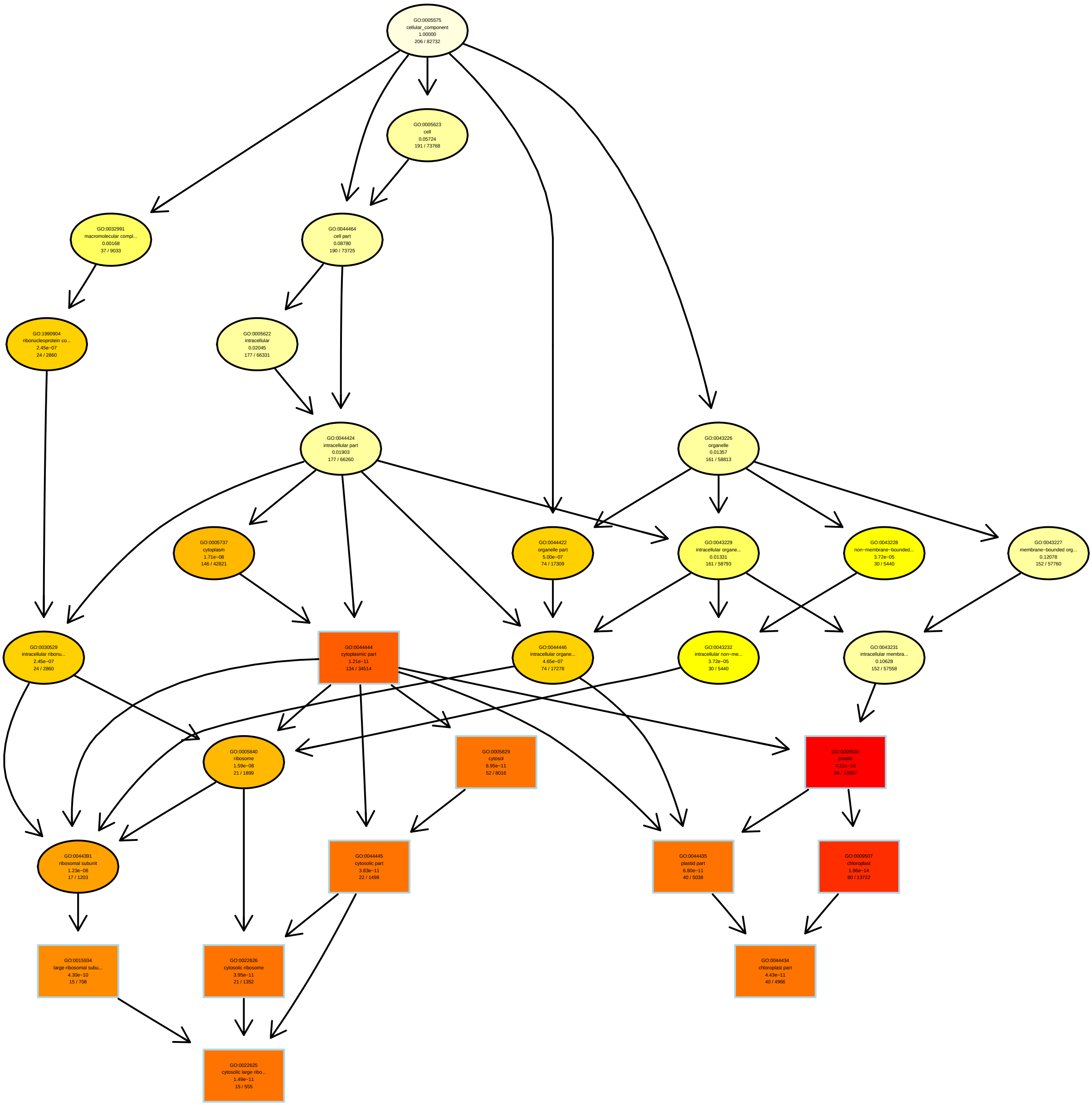

GO:0005575
cellular_component
1.00000
206 / 82732
GO:0005623
cell
0.05724
191 / 73768
GO:0032991
macromolecular compl...
0.00168
37 / 9033
GO:0044464
cell part
0.08780
190 / 73725
GO:1990904
ribonucleoprotein co...
2.45e−07
24 / 2860
GO:0005622
intracellular
0.02045
177 / 66331
GO:0044424
intracellular part
0.01903
177 / 66260
GO:0043226
organelle
0.01357
161 / 58813
GO:0005737
cytoplasm
1.71e−08
146 / 42821
GO:0044422
organelle part
5.00e−07
74 / 17309
GO:0043229
intracellular organe...
0.01331
161 / 58793
GO:0043228
non−membrane−bounded...
3.72e−05
30 / 5440
GO:0043227
membrane−bounded org...
0.12078
152 / 57760
GO:0030529
intracellular ribonu...
2.45e−07
24 / 2860
GO:0044444
cytoplasmic part
1.21e−11
134 / 34514
GO:0044446
intracellular organe...
4.65e−07
74 / 17278
GO:0043232
intracellular non−me...
3.72e−05
30 / 5440
GO:0043231
intracellular membra...
0.10628
152 / 57558
GO:0005840
ribosome
1.59e−08
21 / 1899
GO:0005829
cytosol
8.95e−11
52 / 8016
GO:0009536
plastid
4.21e−16
84 / 13957
GO:0044391
ribosomal subunit
1.23e−08
17 / 1203
GO:0044445
cytosolic part
3.83e−11
22 / 1498
GO:0044435
plastid part
6.80e−11
40 / 5038
GO:0009507
chloroplast
1.96e−14
80 / 13722
GO:0015934
large ribosomal subu...
4.30e−10
15 / 708
GO:0022626
cytosolic ribosome
3.95e−11
21 / 1352
GO:0044434
chloroplast part
4.43e−11
40 / 4966
GO:0022625
cytosolic large ribo...
1.49e−11
15 / 555
