# Supplementary material for: Differential Alternative Splicing Genes and Isoform Regulation Networks of Rapeseed (Brassica napus L.) Infected with Sclerotinia sclerotiorum
Source: Genes (Basel). 2020 Jul 13;11(7):784. doi: 10.3390/genes11070784 (PMC7397149; doi:10.3390/genes11070784)
Supplement: Supplementary file 1 [file genes-11-00784-s001.zip › Fig. S2 Enriched GO terms of genes in the 85 WGCNA modules.zip!/Fig. S2/CytoNodedarkolivegreen4-go.pdf]

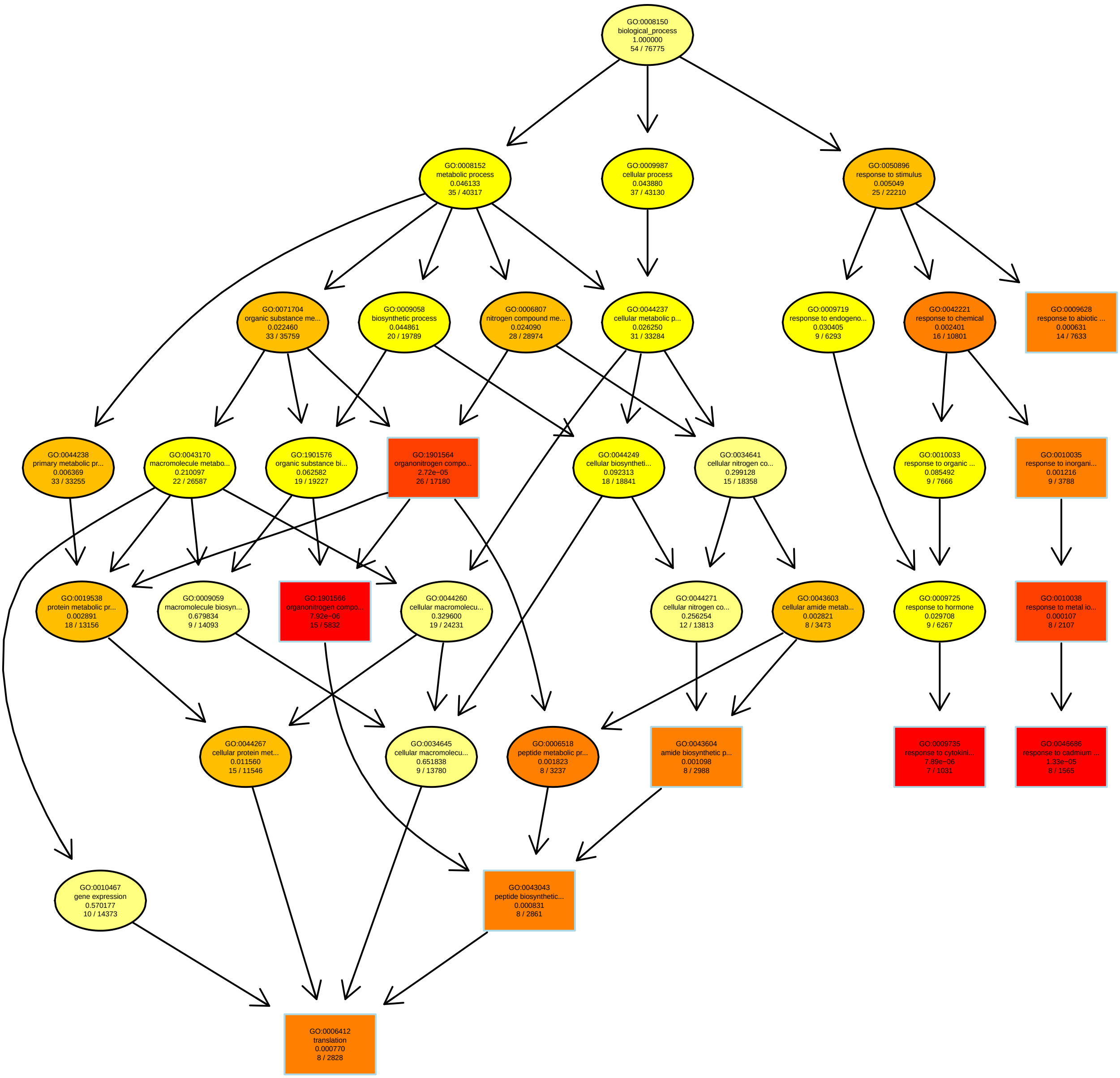

GO:0008150 biological_process 1.000000 54 / 76775
GO:0008152 metabolic process 0.046133 35 / 40317
GO:0009987 cellular process 0.043880 37 / 43130
GO:0050896 response to stimulus 0.005049 25 / 22210
GO:0071704 organic substance me... 0.022460 33 / 35759
GO:0009058 biosynthetic process 0.044861 20 / 19789
GO:0006807 nitrogen compound me... 0.024090 28 / 28974
GO:0044237 cellular metabolic p... 0.026250 31 / 33284
GO:0009719 response to endogeno... 0.030405 9 / 6293
GO:0042221 response to chemical 0.002401 16 / 10801
GO:0009628 response to abiotic ... 0.000631 14 / 7633
GO:0044238 primary metabolic pr... 0.006369 33 / 33255
GO:0043170 macromolecule metabo... 0.210097 22 / 26587
GO:1901576 organic substance bi... 0.062582 19 / 19227
GO:1901564 organonitrogen compo... 2.72e−05 26 / 17180
GO:0044249 cellular biosyntheti... 0.092313 18 / 18841
GO:0034641 cellular nitrogen co... 0.299128 15 / 18358
GO:0010033 response to organic ... 0.085492 9 / 7666
GO:0010035 response to inorgani... 0.001216 9 / 3788
GO:0019538 protein metabolic pr... 0.002891 18 / 13156
GO:0009059 macromolecule biosyn... 0.679834 9 / 14093
GO:1901566 organonitrogen compo... 7.92e−06 15 / 5832
GO:0044260 cellular macromolecu... 0.329600 19 / 24231
GO:0044271 cellular nitrogen co... 0.256254 12 / 13813
GO:0043603 cellular amide metab... 0.002821 8 / 3473
GO:0009725 response to hormone 0.029708 9 / 6267
GO:0010038 response to metal io... 0.000107 8 / 2107
GO:0044267 cellular protein met... 0.011560 15 / 11546
GO:0034645 cellular macromolecu... 0.651838 9 / 13780
GO:0006518 peptide metabolic pr... 0.001823 8 / 3237
GO:0043604 amide biosynthetic p... 0.001098 8 / 2988
GO:0009735 response to cytokini... 7.89e−06 7 / 1031
GO:0046686 response to cadmium ... 1.33e−05 8 / 1565
GO:0010467 gene expression 0.570177 10 / 14373
GO:0043043 peptide biosynthetic... 0.000831 8 / 2861
GO:0006412 translation 0.000770 8 / 2828

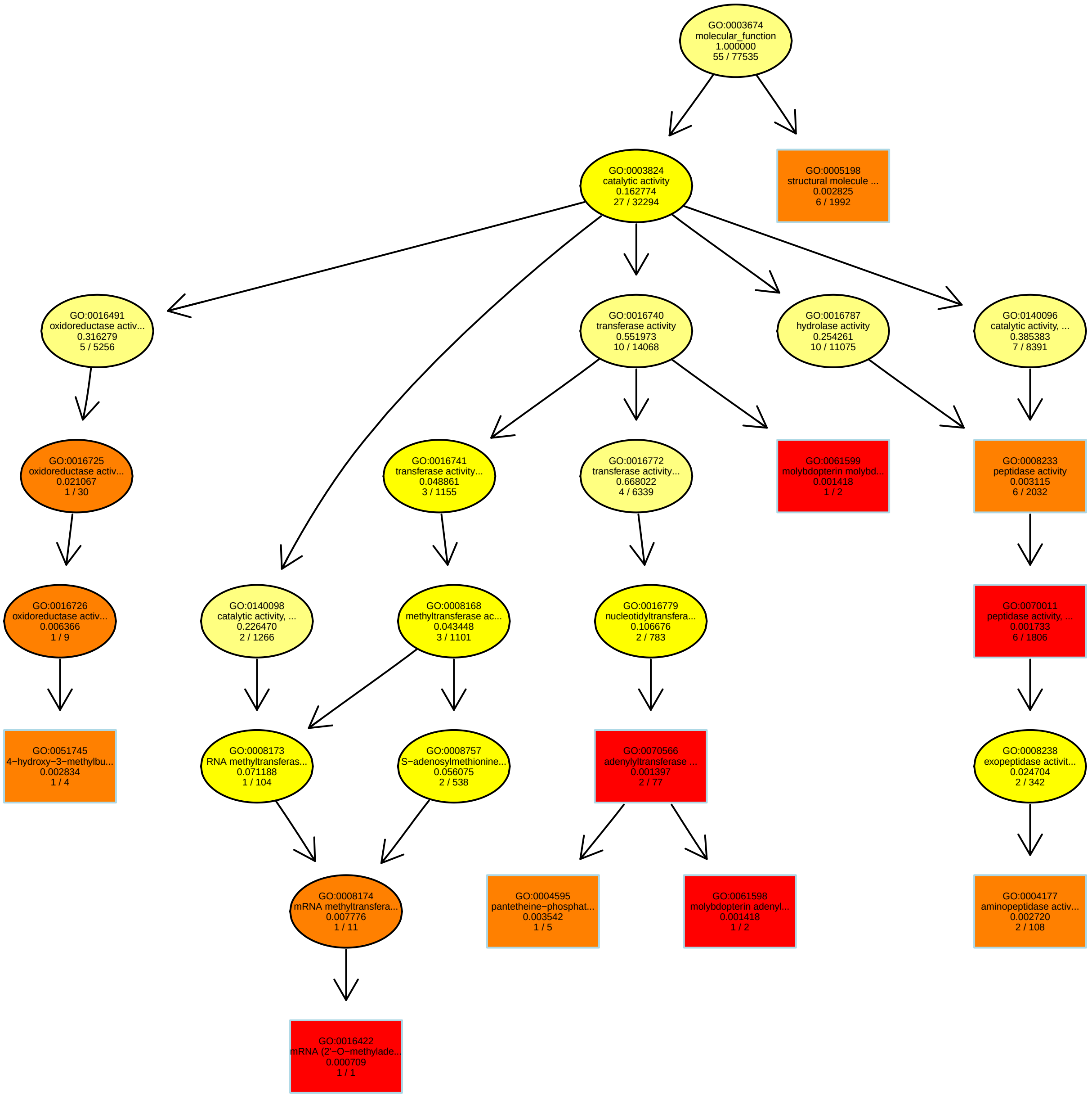

GO:0003674 molecular_function 1.000000 55 / 77535
GO:0003824 catalytic activity 0.162774 27 / 32294
GO:0005198 structural molecule ... 0.002825 6 / 1992
GO:0016491 oxidoreductase activ... 0.316279 5 / 5256
GO:0016740 transferase activity 0.551973 10 / 14068
GO:0016787 hydrolase activity 0.254261 10 / 11075
GO:0140096 catalytic activity, ... 0.385383 7 / 8391
GO:0016725 oxidoreductase activ... 0.021067 1 / 30
GO:0016741 transferase activity... 0.048861 3 / 1155
GO:0016772 transferase activity... 0.668022 4 / 6339
GO:0061599 molybdopterin molybd... 0.001418 1 / 2
GO:0008233 peptidase activity 0.003115 6 / 2032
GO:0016726 oxidoreductase activ... 0.006366 1 / 9
GO:0140098 catalytic activity, ... 0.226470 2 / 1266
GO:0008168 methyltransferase ac... 0.043448 3 / 1101
GO:0016779 nucleotidyltransfera... 0.106676 2 / 783
GO:0070011 peptidase activity, ... 0.001733 6 / 1806
GO:0051745 4−hydroxy−3−methylbu... 0.002834 1 / 4
GO:0008173 RNA methyltransferas... 0.071188 1 / 104
GO:0008757 S−adenosylmethionine... 0.056075 2 / 538
GO:0070566 adenylyltransferase ... 0.001397 2 / 77
GO:0008238 exopeptidase activit... 0.024704 2 / 342
GO:0008174 mRNA methyltransfera... 0.007776 1 / 11
GO:0004595 pantetheine−phosphat... 0.003542 1 / 5
GO:0061598 molybdopterin adenyl... 0.001418 1 / 2
GO:0004177 aminopeptidase activ... 0.002720 2 / 108
GO:0016422 mRNA (2'−O−methylade... 0.000709 1 / 1

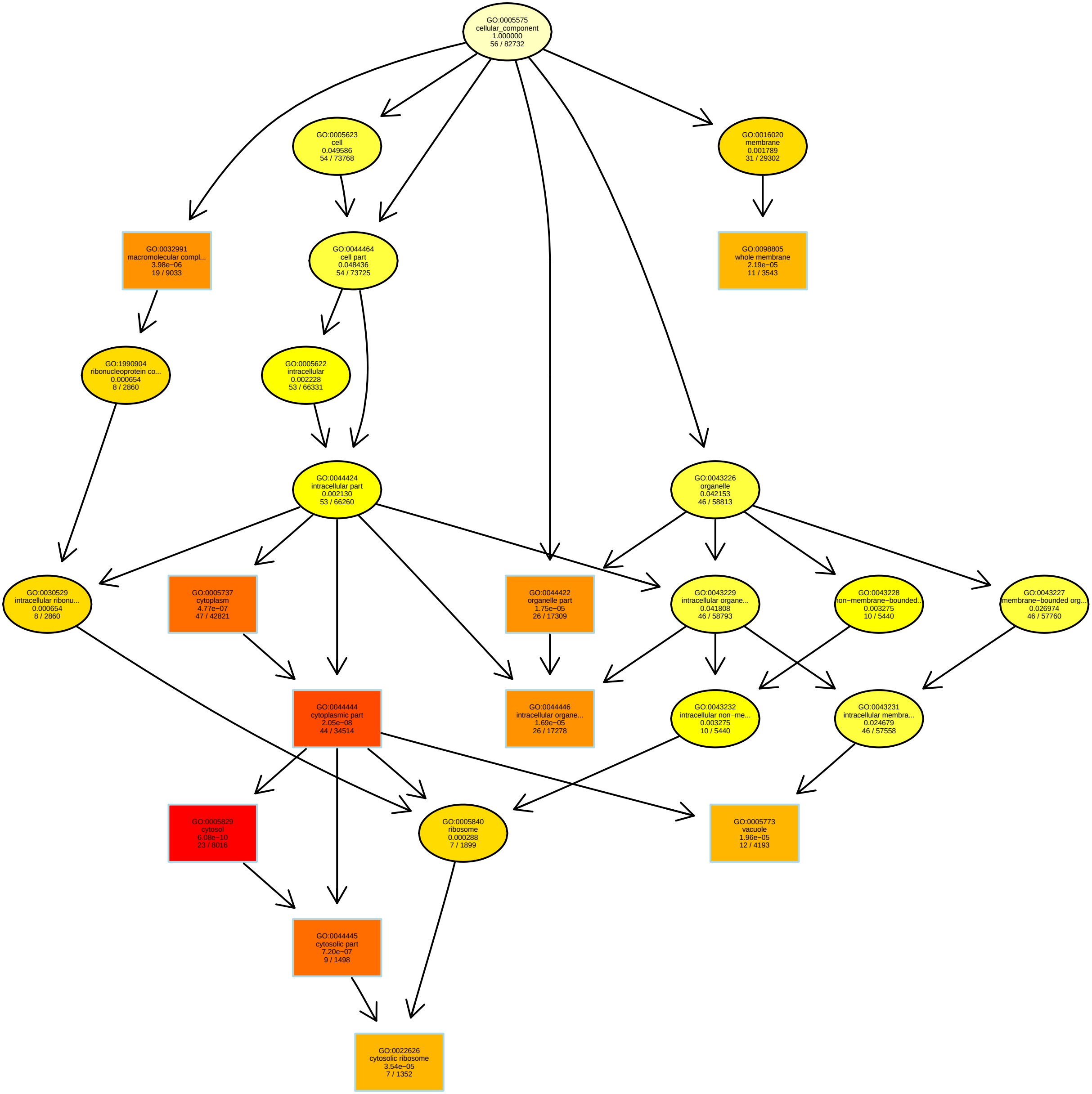
GO:0005575
cellular_component
1.000000
56 / 82732
GO:0005623
cell
0.049586
54 / 73768
GO:0016020
membrane
0.001789
31 / 29302
GO:0032991
macromolecular compl...
3.98e−06
19 / 9033
GO:0044464
cell part
0.048436
54 / 73725
GO:0098805
whole membrane
2.19e−05
11 / 3543
GO:1990904
ribonucleoprotein co...
0.000654
8 / 2860
GO:0005622
intracellular
0.002228
53 / 66331
GO:0044424
intracellular part
0.002130
53 / 66260
GO:0043226
organelle
0.042153
46 / 58813
GO:0030529
intracellular ribonu...
0.000654
8 / 2860
GO:0005737
cytoplasm
4.77e−07
47 / 42821
GO:0044422
organelle part
1.75e−05
26 / 17309
GO:0043229
intracellular organe...
0.041808
46 / 58793
GO:0043228
non−membrane−bounded...
0.003275
10 / 5440
GO:0043227
membrane−bounded org...
0.026974
46 / 57760
GO:0044444
cytoplasmic part
2.05e−08
44 / 34514
GO:0044446
intracellular organe...
1.69e−05
26 / 17278
GO:0043232
intracellular non−me...
0.003275
10 / 5440
GO:0043231
intracellular membra...
0.024679
46 / 57558
GO:0005829
cytosol
6.08e−10
23 / 8016
GO:0005840
ribosome
0.000288
7 / 1899
GO:0005773
vacuole
1.96e−05
12 / 4193
GO:0044445
cytosolic part
7.20e−07
9 / 1498
GO:0022626
cytosolic ribosome
3.54e−05
7 / 1352
