# Supplementary material for: Differential Alternative Splicing Genes and Isoform Regulation Networks of Rapeseed (Brassica napus L.) Infected with Sclerotinia sclerotiorum
Source: Genes (Basel). 2020 Jul 13;11(7):784. doi: 10.3390/genes11070784 (PMC7397149; doi:10.3390/genes11070784)
Supplement: Supplementary file 1 [file genes-11-00784-s001.zip › Fig. S2 Enriched GO terms of genes in the 85 WGCNA modules.zip!/Fig. S2/CytoNodedarkorange-go.pdf]

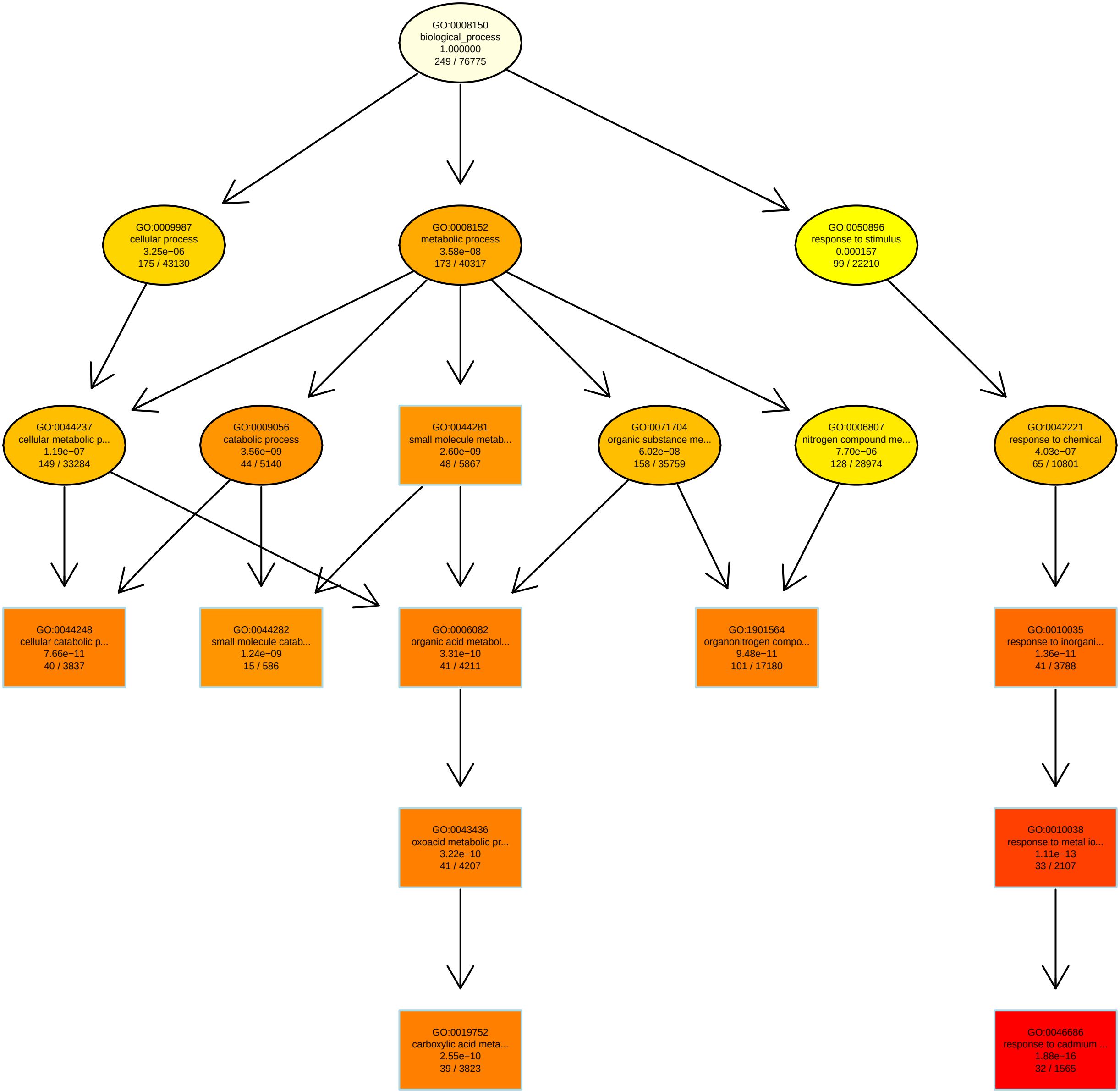
GO:0008150
biological_process
1.000000
249 / 76775
GO:0009987
cellular process
3.25e−06
175 / 43130
GO:0008152
metabolic process
3.58e−08
173 / 40317
GO:0050896
response to stimulus
0.000157
99 / 22210
GO:0044237
cellular metabolic p...
1.19e−07
149 / 33284
GO:0009056
catabolic process
3.56e−09
44 / 5140
GO:0044281
small molecule metab...
2.60e−09
48 / 5867
GO:0071704
organic substance me...
6.02e−08
158 / 35759
GO:0006807
nitrogen compound me...
7.70e−06
128 / 28974
GO:0042221
response to chemical
4.03e−07
65 / 10801
GO:0044248
cellular catabolic p...
7.66e−11
40 / 3837
GO:0044282
small molecule catab...
1.24e−09
15 / 586
GO:0006082
organic acid metabol...
3.31e−10
41 / 4211
GO:1901564
organonitrogen compo...
9.48e−11
101 / 17180
GO:0010035
response to inorgani...
1.36e−11
41 / 3788
GO:0043436
oxoacid metabolic pr...
3.22e−10
41 / 4207
GO:0010038
response to metal io...
1.11e−13
33 / 2107
GO:0019752
carboxylic acid meta...
2.55e−10
39 / 3823
GO:0046686
response to cadmium ...
1.88e−16
32 / 1565

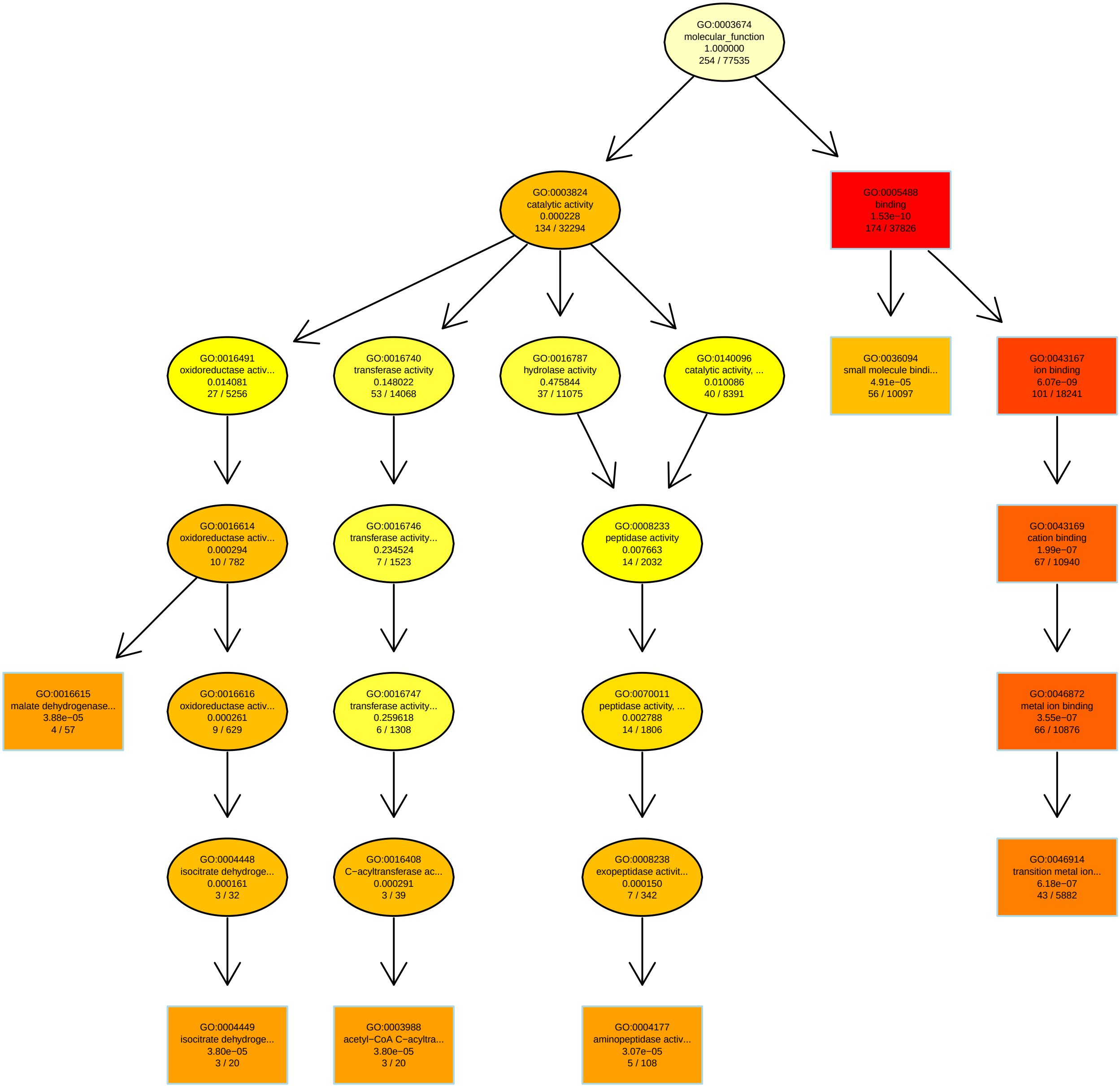
GO:0003674
molecular_function
1.000000
254 / 77535
GO:0003824
catalytic activity
0.000228
134 / 32294
GO:0005488
binding
1.53e−10
174 / 37826
GO:0016491
oxidoreductase activ...
0.014081
27 / 5256
GO:0016740
transferase activity
0.148022
53 / 14068
GO:0016787
hydrolase activity
0.475844
37 / 11075
GO:0140096
catalytic activity, ...
0.010086
40 / 8391
GO:0036094
small molecule bindi...
4.91e−05
56 / 10097
GO:0043167
ion binding
6.07e−09
101 / 18241
GO:0016614
oxidoreductase activ...
0.000294
10 / 782
GO:0016746
transferase activity...
0.234524
7 / 1523
GO:0008233
peptidase activity
0.007663
14 / 2032
GO:0043169
cation binding
1.99e−07
67 / 10940
GO:0016615
malate dehydrogenase...
3.88e−05
4 / 57
GO:0016616
oxidoreductase activ...
0.000261
9 / 629
GO:0016747
transferase activity...
0.259618
6 / 1308
GO:0070011
peptidase activity, ...
0.002788
14 / 1806
GO:0046872
metal ion binding
3.55e−07
66 / 10876
GO:0004448
isocitrate dehydroge...
0.000161
3 / 32
GO:0016408
C−acyltransferase ac...
0.000291
3 / 39
GO:0008238
exopeptidase activit...
0.000150
7 / 342
GO:0046914
transition metal ion...
6.18e−07
43 / 5882
GO:0004449
isocitrate dehydroge...
3.80e−05
3 / 20
GO:0003988
acetyl−CoA C−acyltra...
3.80e−05
3 / 20
GO:0004177
aminopeptidase activ...
3.07e−05
5 / 108

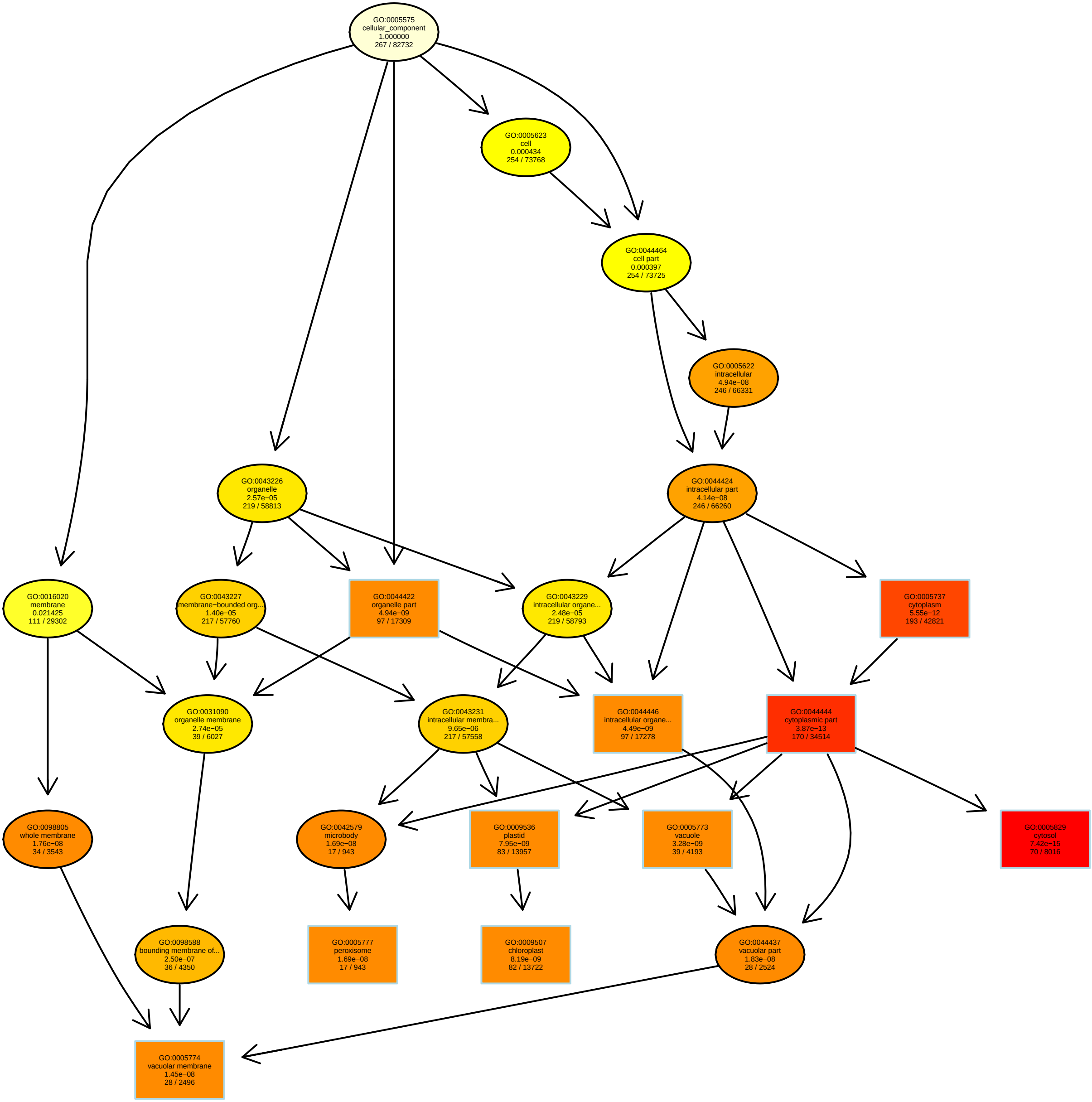

GO:0005575
cellular_component
1.000000
267 / 82732
GO:0005623
cell
0.000434
254 / 73768
GO:0044464
cell part
0.000397
254 / 73725
GO:0005622
intracellular
4.94e−08
246 / 66331
GO:0043226
organelle
2.57e−05
219 / 58813
GO:0044424
intracellular part
4.14e−08
246 / 66260
GO:0016020
membrane
0.021425
111 / 29302
GO:0043227
membrane−bounded org...
1.40e−05
217 / 57760
GO:0044422
organelle part
4.94e−09
97 / 17309
GO:0043229
intracellular organe...
2.48e−05
219 / 58793
GO:0005737
cytoplasm
5.55e−12
193 / 42821
GO:0031090
organelle membrane
2.74e−05
39 / 6027
GO:0043231
intracellular membra...
9.65e−06
217 / 57558
GO:0044446
intracellular organe...
4.49e−09
97 / 17278
GO:0044444
cytoplasmic part
3.87e−13
170 / 34514
GO:0098805
whole membrane
1.76e−08
34 / 3543
GO:0042579
microbody
1.69e−08
17 / 943
GO:0009536
plastid
7.95e−09
83 / 13957
GO:0005773
vacuole
3.28e−09
39 / 4193
GO:0005829
cytosol
7.42e−15
70 / 8016
GO:0098588
bounding membrane of...
2.50e−07
36 / 4350
GO:0005777
peroxisome
1.69e−08
17 / 943
GO:0009507
chloroplast
8.19e−09
82 / 13722
GO:0044437
vacuolar part
1.83e−08
28 / 2524
GO:0005774
vacuolar membrane
1.45e−08
28 / 2496
