# Supplementary material for: Differential Alternative Splicing Genes and Isoform Regulation Networks of Rapeseed (Brassica napus L.) Infected with Sclerotinia sclerotiorum
Source: Genes (Basel). 2020 Jul 13;11(7):784. doi: 10.3390/genes11070784 (PMC7397149; doi:10.3390/genes11070784)
Supplement: Supplementary file 1 [file genes-11-00784-s001.zip › Fig. S2 Enriched GO terms of genes in the 85 WGCNA modules.zip!/Fig. S2/CytoNodedarkorange2-go.pdf]

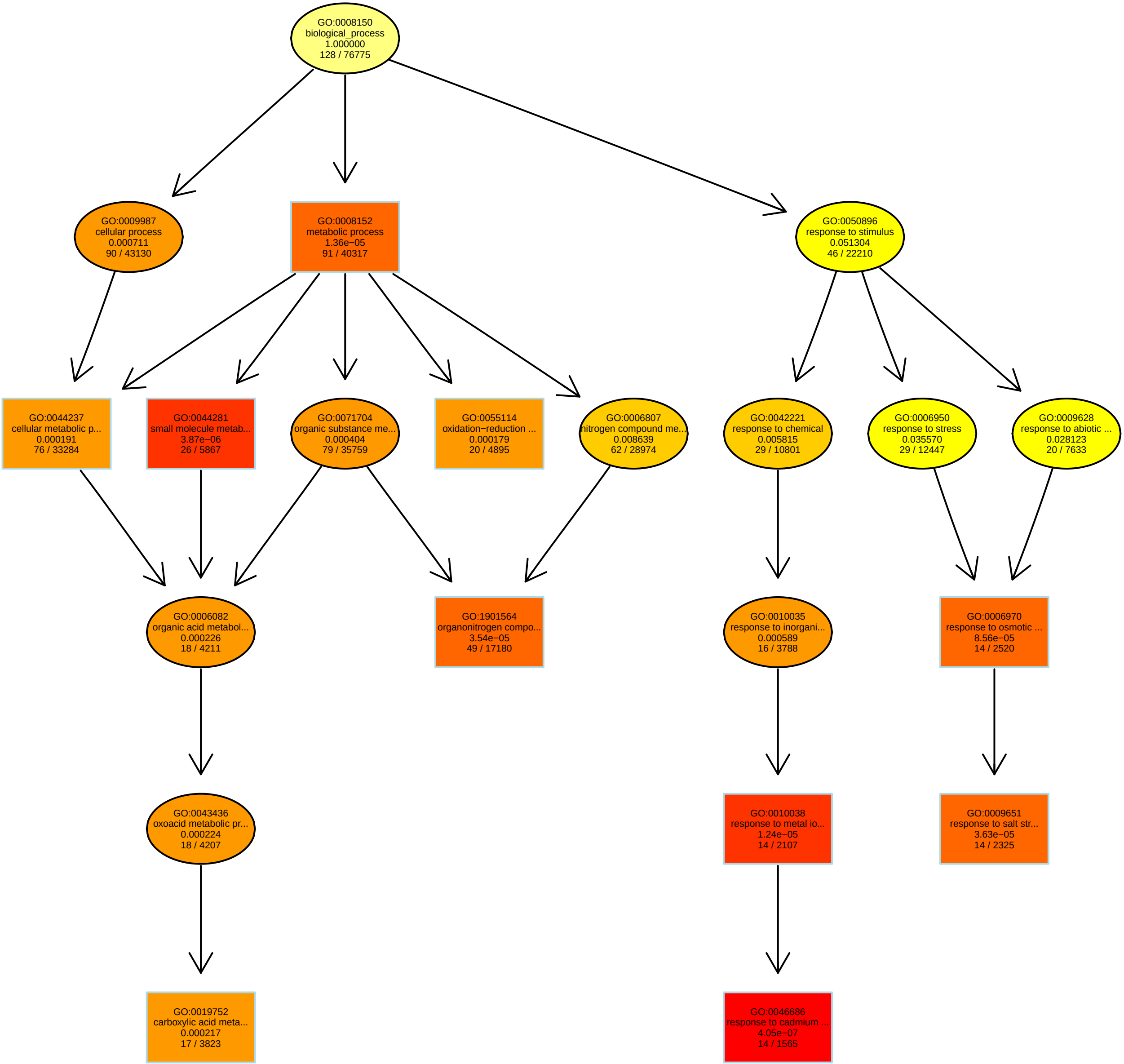
GO:0008150
biological_process
1.000000
128 / 76775
GO:0009987
cellular process
0.000711
90 / 43130
GO:0008152
metabolic process
1.36e−05
91 / 40317
GO:0050896
response to stimulus
0.051304
46 / 22210
GO:0044237
cellular metabolic p...
0.000191
76 / 33284
GO:0044281
small molecule metab...
3.87e−06
26 / 5867
GO:0071704
organic substance me...
0.000404
79 / 35759
GO:0055114
oxidation−reduction ...
0.000179
20 / 4895
GO:0006807
nitrogen compound me...
0.008639
62 / 28974
GO:0042221
response to chemical
0.005815
29 / 10801
GO:0006950
response to stress
0.035570
29 / 12447
GO:0009628
response to abiotic ...
0.028123
20 / 7633
GO:0006082
organic acid metabol...
0.000226
18 / 4211
GO:1901564
organonitrogen compo...
3.54e−05
49 / 17180
GO:0010035
response to inorgani...
0.000589
16 / 3788
GO:0006970
response to osmotic ...
8.56e−05
14 / 2520
GO:0043436
oxoacid metabolic pr...
0.000224
18 / 4207
GO:0010038
response to metal io...
1.24e−05
14 / 2107
GO:0009651
response to salt str...
3.63e−05
14 / 2325
GO:0019752
carboxylic acid meta...
0.000217
17 / 3823
GO:0046686
response to cadmium ...
4.05e−07
14 / 1565

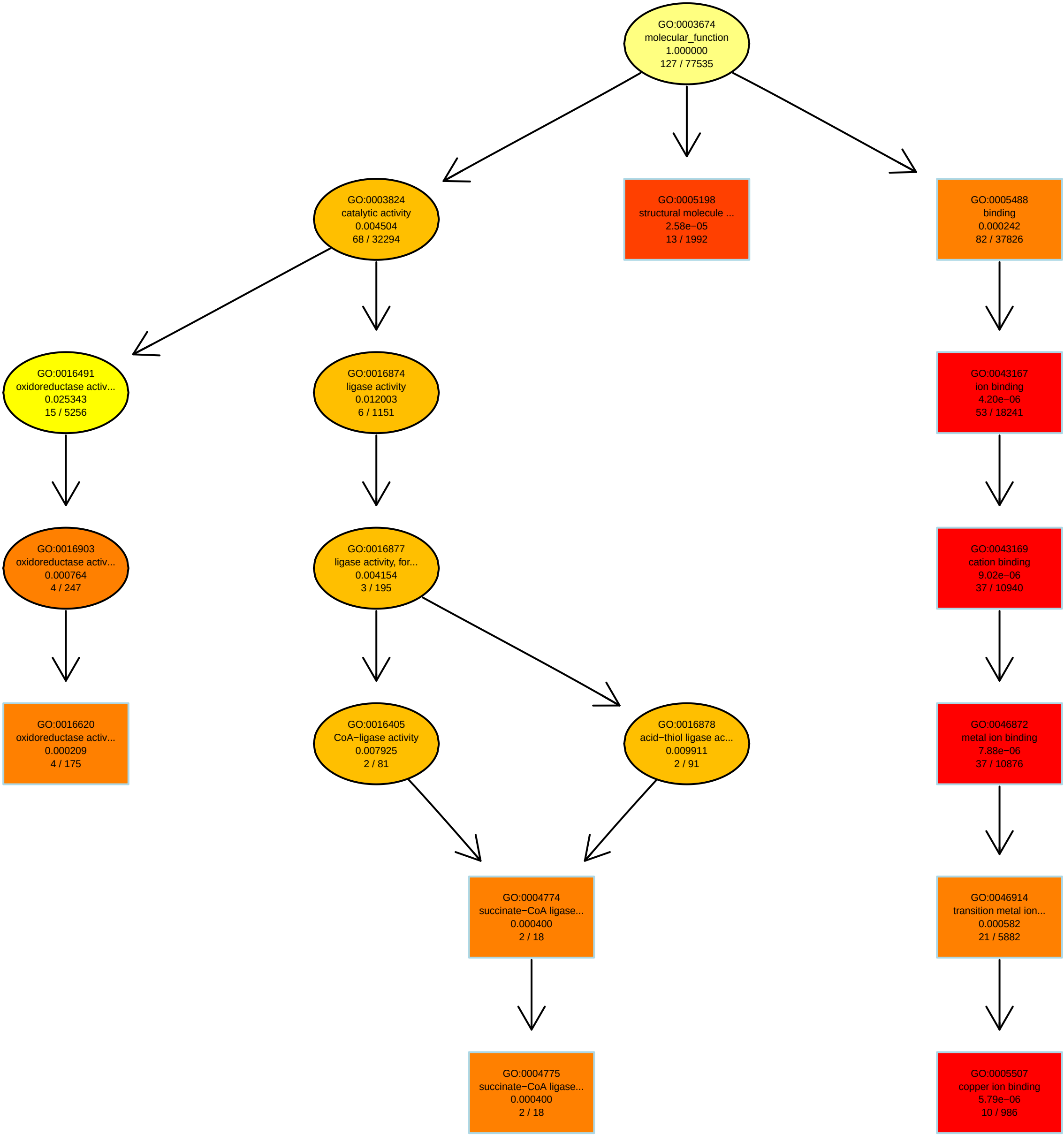
GO:0003674
molecular_function
1.000000
127 / 77535
GO:0003824
catalytic activity
0.004504
68 / 32294
GO:0005198
structural molecule ...
2.58e−05
13 / 1992
GO:0005488
binding
0.000242
82 / 37826
GO:0016491
oxidoreductase activ...
0.025343
15 / 5256
GO:0016874
ligase activity
0.012003
6 / 1151
GO:0043167
ion binding
4.20e−06
53 / 18241
GO:0016903
oxidoreductase activ...
0.000764
4 / 247
GO:0016877
ligase activity, for...
0.004154
3 / 195
GO:0043169
cation binding
9.02e−06
37 / 10940
GO:0016620
oxidoreductase activ...
0.000209
4 / 175
GO:0016405
CoA−ligase activity
0.007925
2 / 81
GO:0016878
acid−thiol ligase ac...
0.009911
2 / 91
GO:0046872
metal ion binding
7.88e−06
37 / 10876
GO:0004774
succinate−CoA ligase...
0.000400
2 / 18
GO:0046914
transition metal ion...
0.000582
21 / 5882
GO:0004775
succinate−CoA ligase...
0.000400
2 / 18
GO:0005507
copper ion binding
5.79e−06
10 / 986

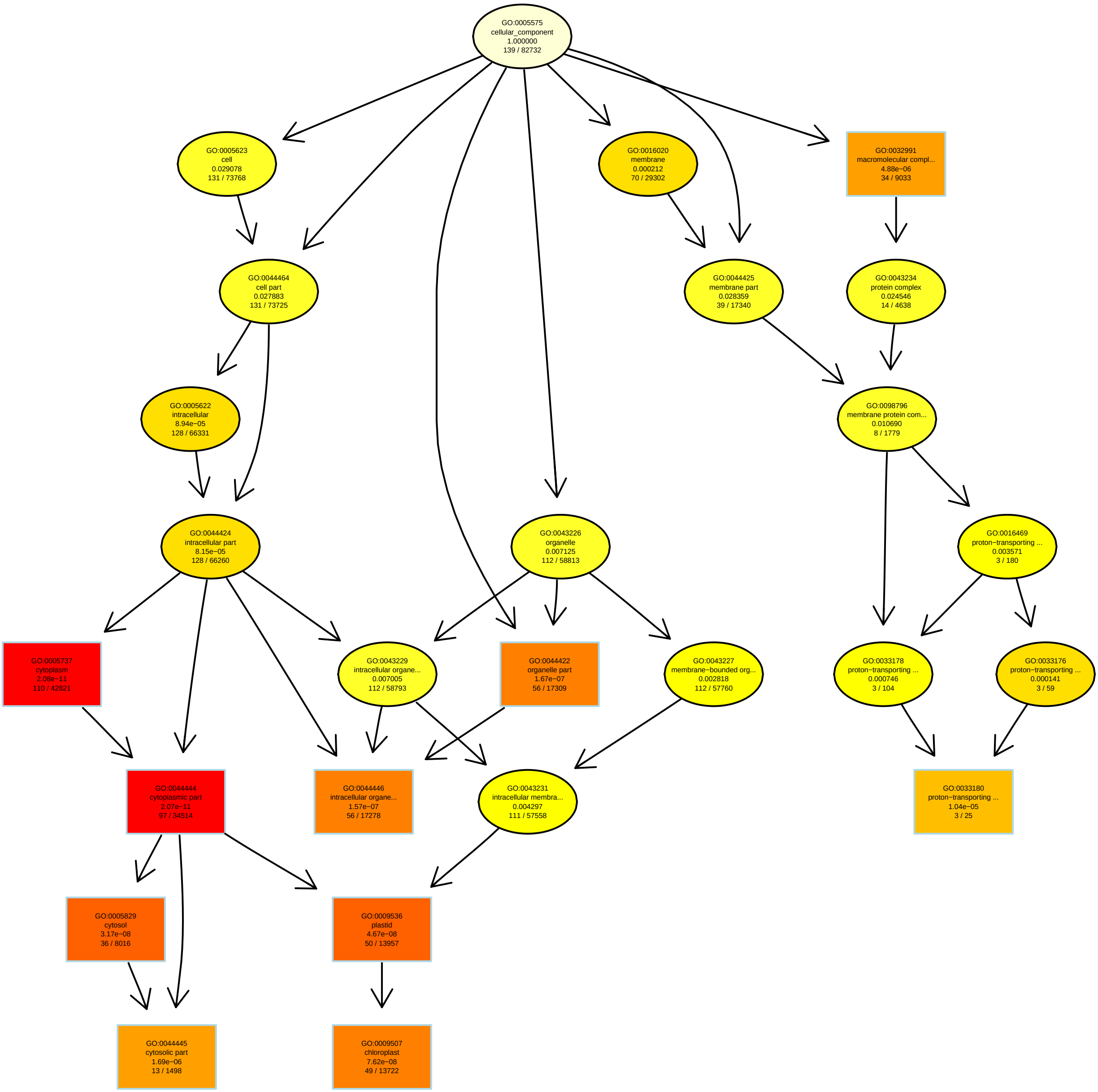
GO:0005575 cellular_component 1.000000 139 / 82732
GO:0005623 cell 0.029078 131 / 73768
GO:0016020 membrane 0.000212 70 / 29302
GO:0032991 macromolecular compl... 4.88e−06 34 / 9033
GO:0044464 cell part 0.027883 131 / 73725
GO:0044425 membrane part 0.028359 39 / 17340
GO:0043234 protein complex 0.024546 14 / 4638
GO:0005622 intracellular 8.94e−05 128 / 66331
GO:0098796 membrane protein com... 0.010690 8 / 1779
GO:0044424 intracellular part 8.15e−05 128 / 66260
GO:0043226 organelle 0.007125 112 / 58813
GO:0016469 proton−transporting ... 0.003571 3 / 180
GO:0005737 cytoplasm 2.08e−11 110 / 42821
GO:0043229 intracellular organe... 0.007005 112 / 58793
GO:0044422 organelle part 1.67e−07 56 / 17309
GO:0043227 membrane−bounded org... 0.002818 112 / 57760
GO:0033178 proton−transporting ... 0.000746 3 / 104
GO:0033176 proton−transporting ... 0.000141 3 / 59
GO:0044444 cytoplasmic part 2.07e−11 97 / 34514
GO:0044446 intracellular organe... 1.57e−07 56 / 17278
GO:0043231 intracellular membra... 0.004297 111 / 57558
GO:0033180 proton−transporting ... 1.04e−05 3 / 25
GO:0005829 cytosol 3.17e−08 36 / 8016
GO:0009536 plastid 4.67e−08 50 / 13957
GO:0044445 cytosolic part 1.69e−06 13 / 1498
GO:0009507 chloroplast 7.62e−08 49 / 13722
