# Supplementary material for: Differential Alternative Splicing Genes and Isoform Regulation Networks of Rapeseed (Brassica napus L.) Infected with Sclerotinia sclerotiorum
Source: Genes (Basel). 2020 Jul 13;11(7):784. doi: 10.3390/genes11070784 (PMC7397149; doi:10.3390/genes11070784)
Supplement: Supplementary file 1 [file genes-11-00784-s001.zip › Fig. S2 Enriched GO terms of genes in the 85 WGCNA modules.zip!/Fig. S2/CytoNodedarkred-go.pdf]

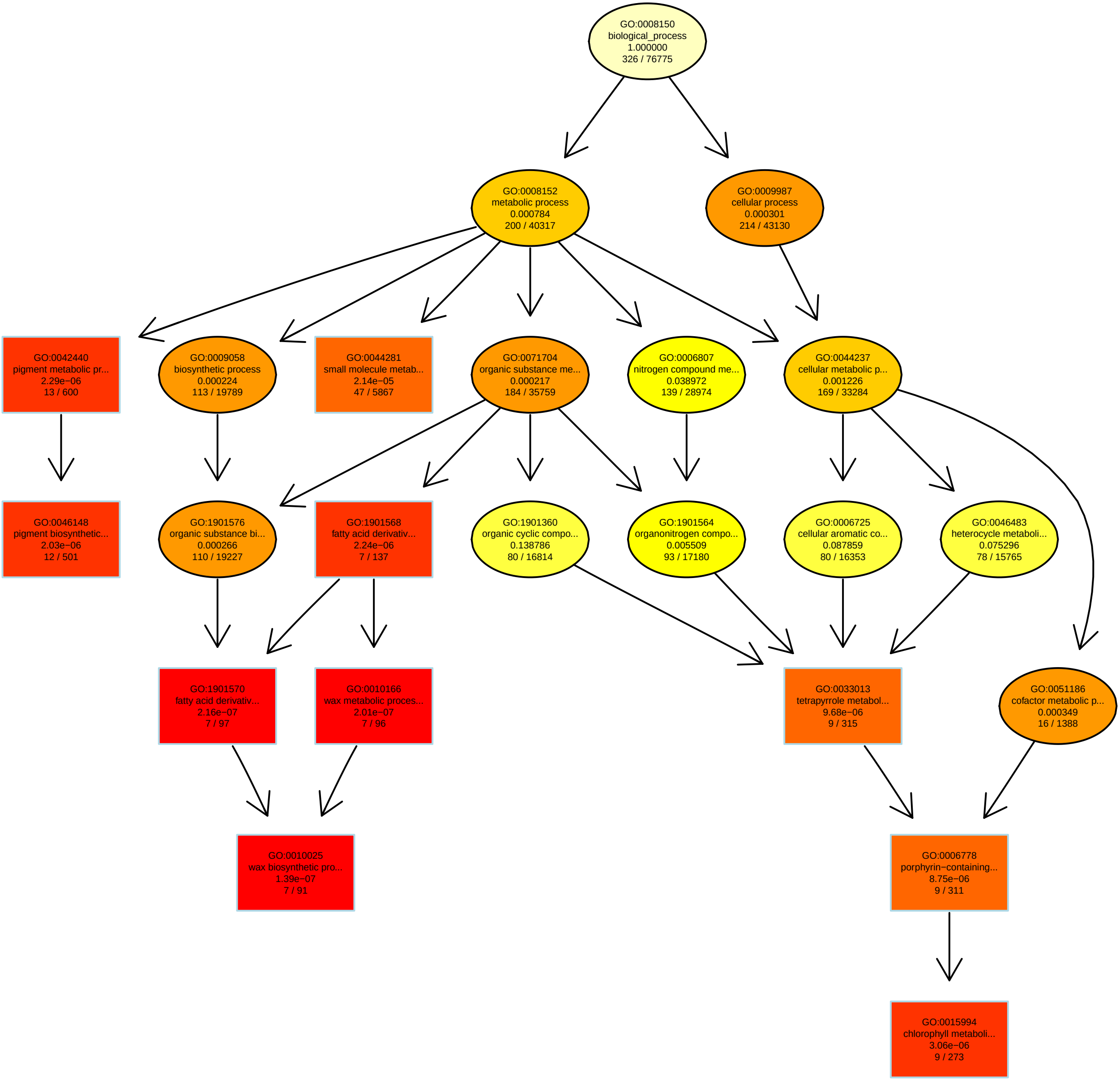
GO:0008150
biological_process
1.000000
326 / 76775
GO:0008152
metabolic process
0.000784
200 / 40317
GO:0009987
cellular process
0.000301
214 / 43130
GO:0042440
pigment metabolic pr...
2.29e−06
13 / 600
GO:0009058
biosynthetic process
0.000224
113 / 19789
GO:0044281
small molecule metab...
2.14e−05
47 / 5867
GO:0071704
organic substance me...
0.000217
184 / 35759
GO:0006807
nitrogen compound me...
0.038972
139 / 28974
GO:0044237
cellular metabolic p...
0.001226
169 / 33284
GO:0046148
pigment biosynthetic...
2.03e−06
12 / 501
GO:1901576
organic substance bi...
0.000266
110 / 19227
GO:1901568
fatty acid derivativ...
2.24e−06
7 / 137
GO:1901360
organic cyclic compo...
0.138786
80 / 16814
GO:1901564
organonitrogen compo...
0.005509
93 / 17180
GO:0006725
cellular aromatic co...
0.087859
80 / 16353
GO:0046483
heterocycle metaboli...
0.075296
78 / 15765
GO:1901570
fatty acid derivativ...
2.16e−07
7 / 97
GO:0010166
wax metabolic proces...
2.01e−07
7 / 96
GO:0033013
tetrapyrrole metabol...
9.68e−06
9 / 315
GO:0051186
cofactor metabolic p...
0.000349
16 / 1388
GO:0010025
wax biosynthetic pro...
1.39e−07
7 / 91
GO:0006778
porphyrin−containing...
8.75e−06
9 / 311
GO:0015994
chlorophyll metaboli...
3.06e−06
9 / 273

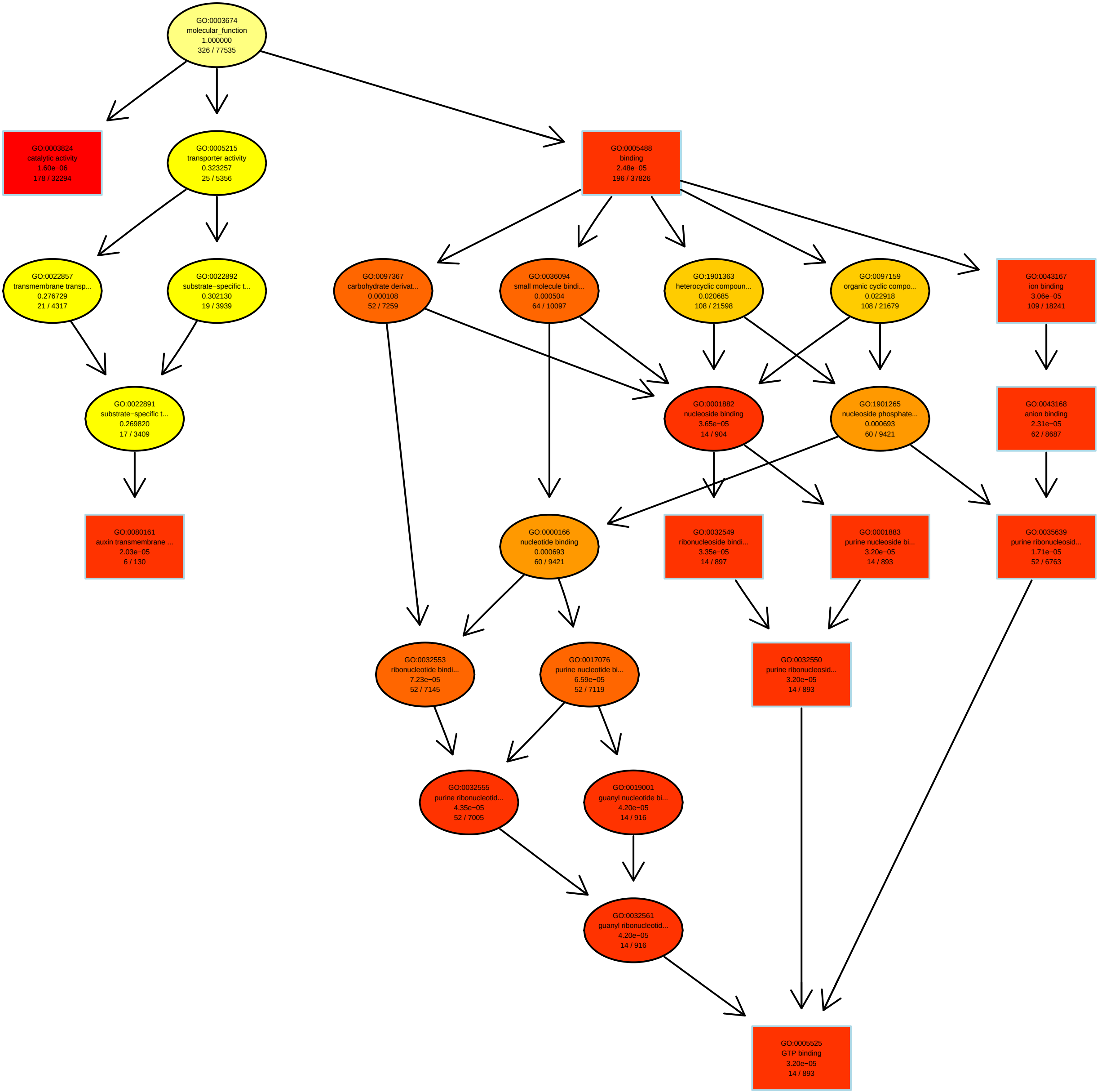
GO:0003674
molecular_function
1.000000
326 / 77535
GO:0003824
catalytic activity
1.60e−06
178 / 32294
GO:0005215
transporter activity
0.323257
25 / 5356
GO:0005488
binding
2.48e−05
196 / 37826
GO:0022857
transmembrane transp...
0.276729
21 / 4317
GO:0022892
substrate−specific t...
0.302130
19 / 3939
GO:0097367
carbohydrate derivat...
0.000108
52 / 7259
GO:0036094
small molecule bindi...
0.000504
64 / 10097
GO:1901363
heterocyclic compoun...
0.020685
108 / 21598
GO:0097159
organic cyclic compo...
0.022918
108 / 21679
GO:0043167
ion binding
3.06e−05
109 / 18241
GO:0022891
substrate−specific t...
0.269820
17 / 3409
GO:0001882
nucleoside binding
3.65e−05
14 / 904
GO:1901265
nucleoside phosphate...
0.000693
60 / 9421
GO:0043168
anion binding
2.31e−05
62 / 8687
GO:0080161
auxin transmembrane ...
2.03e−05
6 / 130
GO:0000166
nucleotide binding
0.000693
60 / 9421
GO:0032549
ribonucleoside bindi...
3.35e−05
14 / 897
GO:0001883
purine nucleoside bi...
3.20e−05
14 / 893
GO:0035639
purine ribonucleosid...
1.71e−05
52 / 6763
GO:0032553
ribonucleotide bindi...
7.23e−05
52 / 7145
GO:0017076
purine nucleotide bi...
6.59e−05
52 / 7119
GO:0032550
purine ribonucleosid...
3.20e−05
14 / 893
GO:0032555
purine ribonucleotid...
4.35e−05
52 / 7005
GO:0019001
guanyl nucleotide bi...
4.20e−05
14 / 916
GO:0032561
guanyl ribonucleotid...
4.20e−05
14 / 916
GO:0005525
GTP binding
3.20e−05
14 / 893

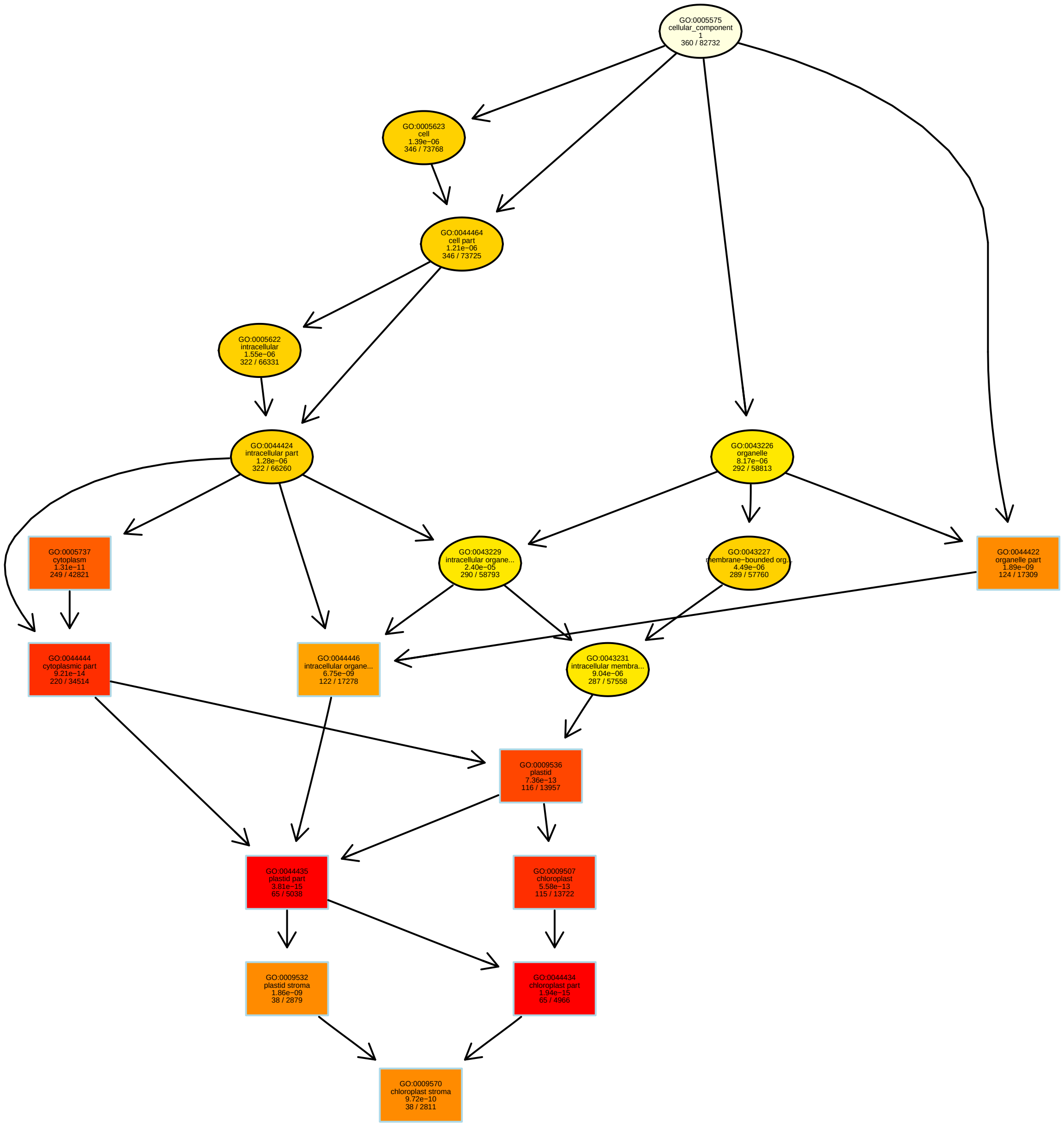

GO:0005575
cellular_component
1
360 / 82732
GO:0005623
cell
1.39e−06
346 / 73768
GO:0044464
cell part
1.21e−06
346 / 73725
GO:0005622
intracellular
1.55e−06
322 / 66331
GO:0044424
intracellular part
1.28e−06
322 / 66260
GO:0043226
organelle
8.17e−06
292 / 58813
GO:0005737
cytoplasm
1.31e−11
249 / 42821
GO:0043229
intracellular organe...
2.40e−05
290 / 58793
GO:0043227
membrane−bounded org...
4.49e−06
289 / 57760
GO:0044422
organelle part
1.89e−09
124 / 17309
GO:0044444
cytoplasmic part
9.21e−14
220 / 34514
GO:0044446
intracellular organe...
6.75e−09
122 / 17278
GO:0043231
intracellular membra...
9.04e−06
287 / 57558
GO:0009536
plastid
7.36e−13
116 / 13957
GO:0044435
plastid part
3.81e−15
65 / 5038
GO:0009507
chloroplast
5.58e−13
115 / 13722
GO:0009532
plastid stroma
1.86e−09
38 / 2879
GO:0044434
chloroplast part
1.94e−15
65 / 4966
GO:0009570
chloroplast stroma
9.72e−10
38 / 2811
