# Supplementary material for: Differential Alternative Splicing Genes and Isoform Regulation Networks of Rapeseed (Brassica napus L.) Infected with Sclerotinia sclerotiorum
Source: Genes (Basel). 2020 Jul 13;11(7):784. doi: 10.3390/genes11070784 (PMC7397149; doi:10.3390/genes11070784)
Supplement: Supplementary file 1 [file genes-11-00784-s001.zip › Fig. S2 Enriched GO terms of genes in the 85 WGCNA modules.zip!/Fig. S2/CytoNodedarkseagreen4-go.pdf]

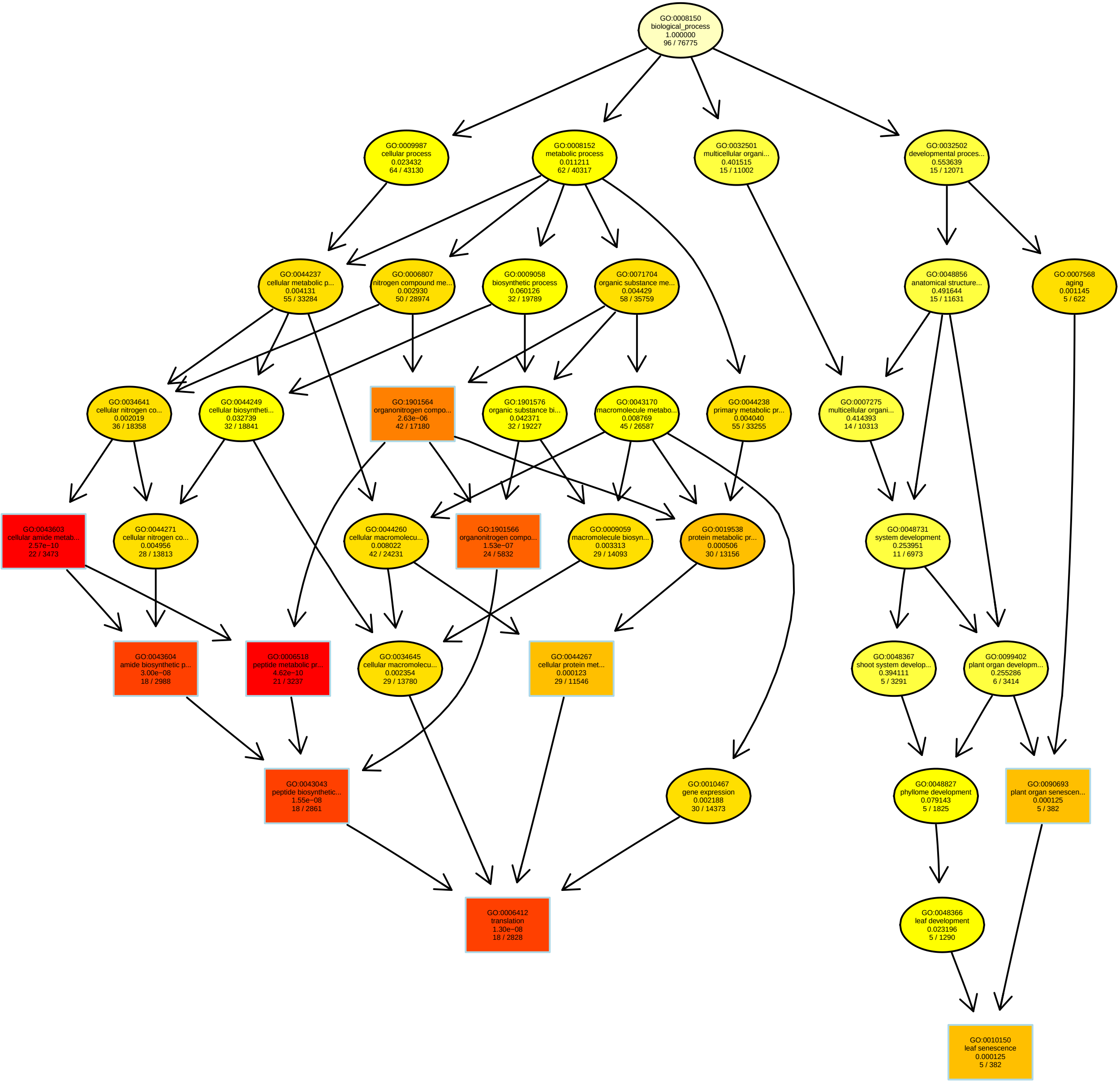

GO:0008150
biological_process
1.000000
96 / 76775
GO:0009987
cellular process
0.023432
64 / 43130
GO:0008152
metabolic process
0.011211
62 / 40317
GO:0032501
multicellular organi...
0.401515
15 / 11002
GO:0032502
developmental proces...
0.553639
15 / 12071
GO:0044237
cellular metabolic p...
0.004131
55 / 33284
GO:0006807
nitrogen compound me...
0.002930
50 / 28974
GO:0009058
biosynthetic process
0.060126
32 / 19789
GO:0071704
organic substance me...
0.004429
58 / 35759
GO:0048856
anatomical structure...
0.491644
15 / 11631
GO:0007568
aging
0.001145
5 / 622
GO:0034641
cellular nitrogen co...
0.002019
36 / 18358
GO:0044249
cellular biosyntheti...
0.032739
32 / 18841
GO:1901564
organonitrogen compo...
2.63e−06
42 / 17180
GO:1901576
organic substance bi...
0.042371
32 / 19227
GO:0043170
macromolecule metabo...
0.008769
45 / 26587
GO:0044238
primary metabolic pr...
0.004040
55 / 33255
GO:0007275
multicellular organi...
0.414393
14 / 10313
GO:0043603
cellular amide metab...
2.57e−10
22 / 3473
GO:0044271
cellular nitrogen co...
0.004956
28 / 13813
GO:0044260
cellular macromolecu...
0.008022
42 / 24231
GO:1901566
organonitrogen compo...
1.53e−07
24 / 5832
GO:0009059
macromolecule biosyn...
0.003313
29 / 14093
GO:0019538
protein metabolic pr...
0.000506
30 / 13156
GO:0048731
system development
0.253951
11 / 6973
GO:0043604
amide biosynthetic p...
3.00e−08
18 / 2988
GO:0006518
peptide metabolic pr...
4.62e−10
21 / 3237
GO:0034645
cellular macromolecu...
0.002354
29 / 13780
GO:0044267
cellular protein met...
0.000123
29 / 11546
GO:0048367
shoot system develop...
0.394111
5 / 3291
GO:0099402
plant organ developm...
0.255286
6 / 3414
GO:0043043
peptide biosynthetic...
1.55e−08
18 / 2861
GO:0010467
gene expression
0.002188
30 / 14373
GO:0048827
phyllome development
0.079143
5 / 1825
GO:0090693
plant organ senescen...
0.000125
5 / 382
GO:0006412
translation
1.30e−08
18 / 2828
GO:0048366
leaf development
0.023196
5 / 1290
GO:0010150
leaf senescence
0.000125
5 / 382

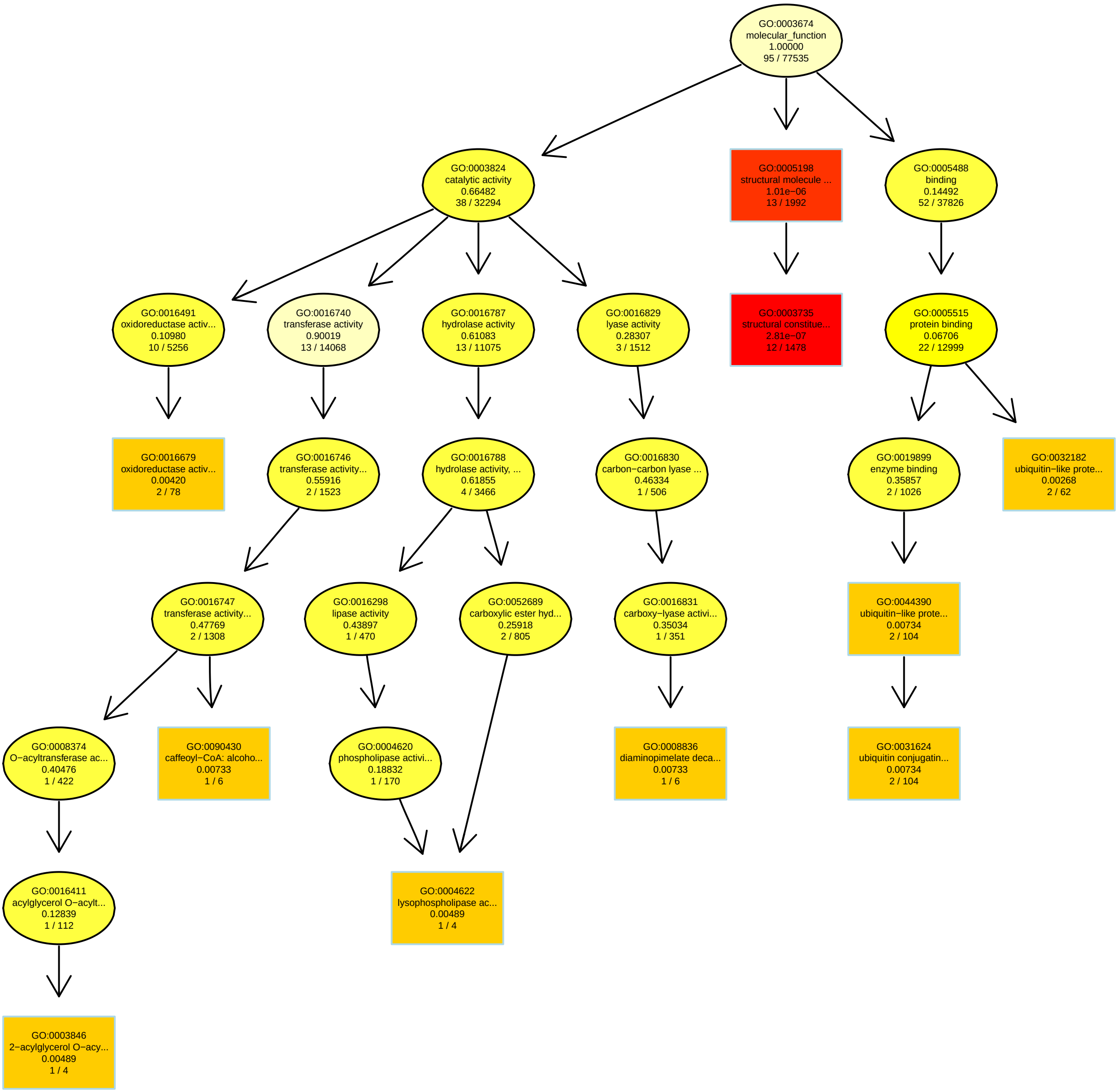

GO:0003674
molecular_function
1.00000
95 / 77535
GO:0003824
catalytic activity
0.66482
38 / 32294
GO:0005198
structural molecule ...
1.01e−06
13 / 1992
GO:0005488
binding
0.14492
52 / 37826
GO:0016491
oxidoreductase activ...
0.10980
10 / 5256
GO:0016740
transferase activity
0.90019
13 / 14068
GO:0016787
hydrolase activity
0.61083
13 / 11075
GO:0016829
lyase activity
0.28307
3 / 1512
GO:0003735
structural constitue...
2.81e−07
12 / 1478
GO:0005515
protein binding
0.06706
22 / 12999
GO:0016679
oxidoreductase activ...
0.00420
2 / 78
GO:0016746
transferase activity...
0.55916
2 / 1523
GO:0016788
hydrolase activity, ...
0.61855
4 / 3466
GO:0016830
carbon−carbon lyase ...
0.46334
1 / 506
GO:0019899
enzyme binding
0.35857
2 / 1026
GO:0032182
ubiquitin−like prote...
0.00268
2 / 62
GO:0016747
transferase activity...
0.47769
2 / 1308
GO:0016298
lipase activity
0.43897
1 / 470
GO:0052689
carboxylic ester hyd...
0.25918
2 / 805
GO:0016831
carboxy−lyase activi...
0.35034
1 / 351
GO:0044390
ubiquitin−like prote...
0.00734
2 / 104
GO:0008374
O−acyltransferase ac...
0.40476
1 / 422
GO:0090430
caffeoyl−CoA: alcoho...
0.00733
1 / 6
GO:0004620
phospholipase activi...
0.18832
1 / 170
GO:0008836
diaminopimelate deca...
0.00733
1 / 6
GO:0031624
ubiquitin conjugatin...
0.00734
2 / 104
GO:0016411
acylglycerol O−acylt...
0.12839
1 / 112
GO:0004622
lysophospholipase ac...
0.00489
1 / 4
GO:0003846
2−acylglycerol O−acy...
0.00489
1 / 4

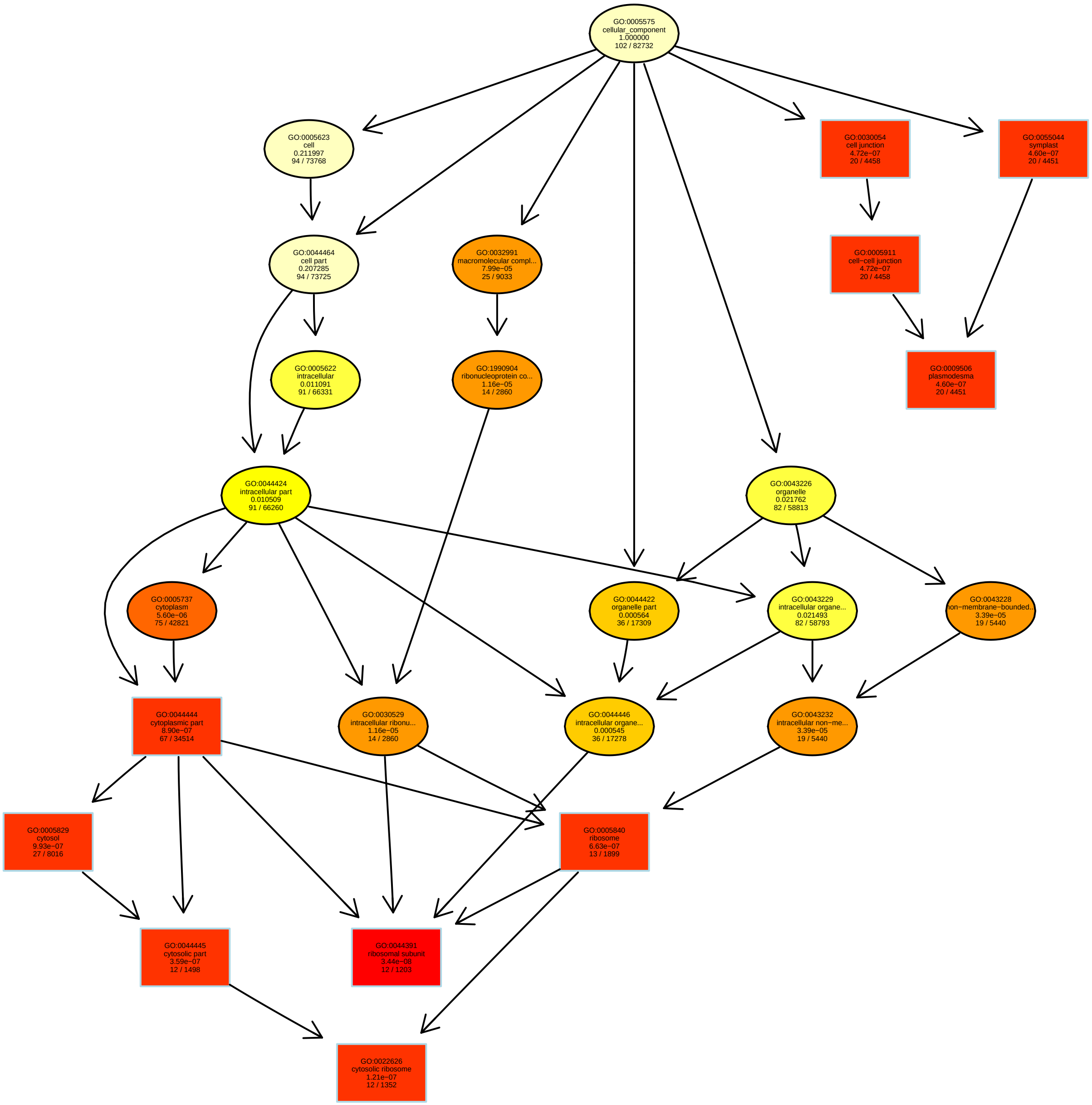

GO:0005575
cellular_component
1.000000
102 / 82732
GO:0005623
cell
0.211997
94 / 73768
GO:0030054
cell junction
4.72e−07
20 / 4458
GO:0055044
symplast
4.60e−07
20 / 4451
GO:0044464
cell part
0.207285
94 / 73725
GO:0032991
macromolecular compl...
7.99e−05
25 / 9033
GO:0005911
cell−cell junction
4.72e−07
20 / 4458
GO:0005622
intracellular
0.011091
91 / 66331
GO:1990904
ribonucleoprotein co...
1.16e−05
14 / 2860
GO:0009506
plasmodesma
4.60e−07
20 / 4451
GO:0044424
intracellular part
0.010509
91 / 66260
GO:0043226
organelle
0.021762
82 / 58813
GO:0005737
cytoplasm
5.60e−06
75 / 42821
GO:0044422
organelle part
0.000564
36 / 17309
GO:0043229
intracellular organe...
0.021493
82 / 58793
GO:0043228
non−membrane−bounded...
3.39e−05
19 / 5440
GO:0044444
cytoplasmic part
8.90e−07
67 / 34514
GO:0030529
intracellular ribonu...
1.16e−05
14 / 2860
GO:0044446
intracellular organe...
0.000545
36 / 17278
GO:0043232
intracellular non−me...
3.39e−05
19 / 5440
GO:0005829
cytosol
9.93e−07
27 / 8016
GO:0005840
ribosome
6.63e−07
13 / 1899
GO:0044445
cytosolic part
3.59e−07
12 / 1498
GO:0044391
ribosomal subunit
3.44e−08
12 / 1203
GO:0022626
cytosolic ribosome
1.21e−07
12 / 1352
