# Supplementary material for: Differential Alternative Splicing Genes and Isoform Regulation Networks of Rapeseed (Brassica napus L.) Infected with Sclerotinia sclerotiorum
Source: Genes (Basel). 2020 Jul 13;11(7):784. doi: 10.3390/genes11070784 (PMC7397149; doi:10.3390/genes11070784)
Supplement: Supplementary file 1 [file genes-11-00784-s001.zip › Fig. S2 Enriched GO terms of genes in the 85 WGCNA modules.zip!/Fig. S2/CytoNodedarkslateblue-go.pdf]

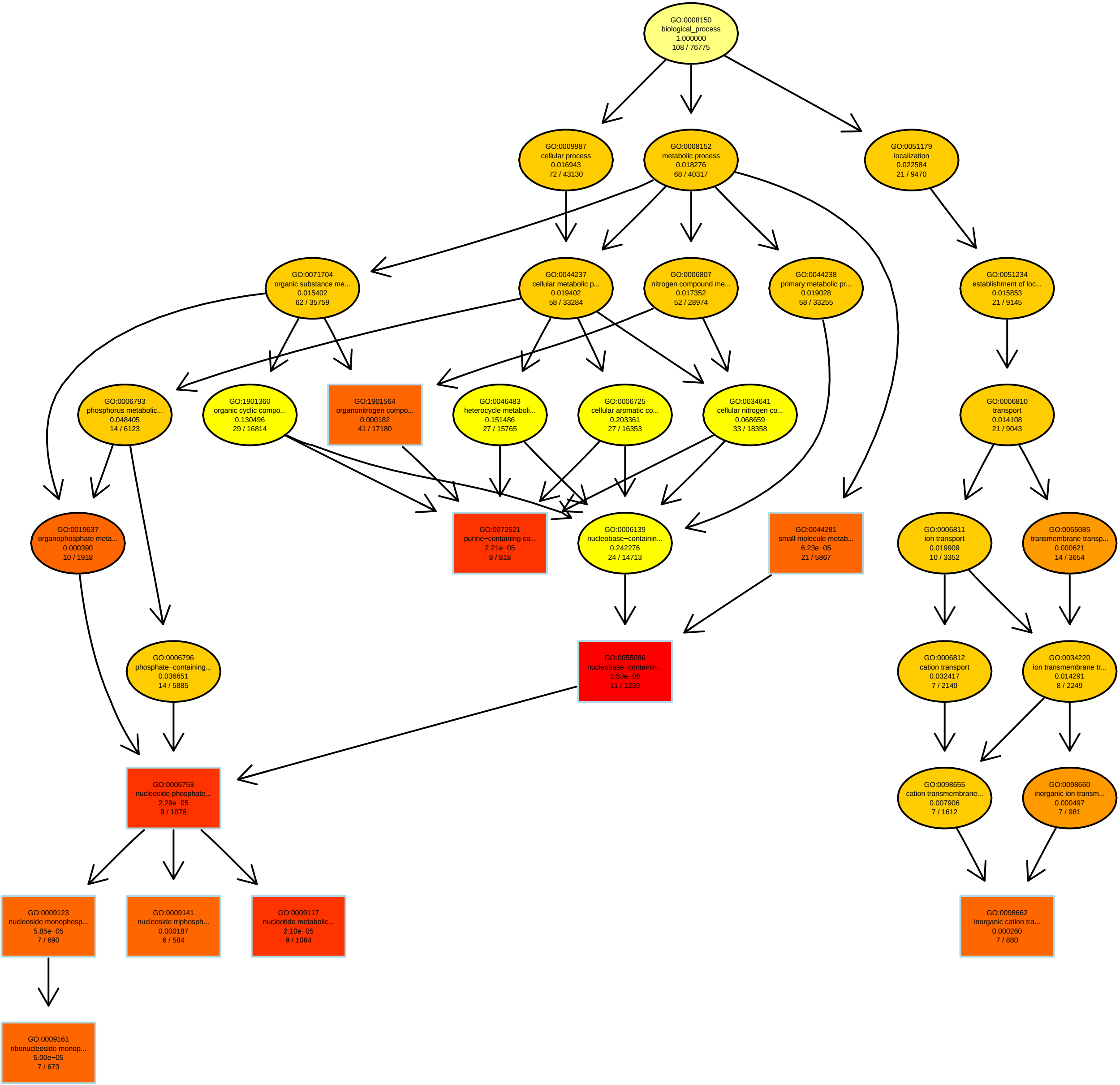

GO:0008150 biological_process 1.000000 108 / 76775
GO:0009987 cellular process 0.016943 72 / 43130
GO:0008152 metabolic process 0.018276 68 / 40317
GO:0051179 localization 0.022584 21 / 9470
GO:0071704 organic substance me... 0.015402 62 / 35759
GO:0044237 cellular metabolic p... 0.019402 58 / 33284
GO:0006807 nitrogen compound me... 0.017352 52 / 28974
GO:0044238 primary metabolic pr... 0.019028 58 / 33255
GO:0051234 establishment of loc... 0.015853 21 / 9145
GO:0006793 phosphorus metabolic... 0.048405 14 / 6123
GO:1901360 organic cyclic compo... 0.130496 29 / 16814
GO:1901564 organonitrogen compo... 0.000182 41 / 17180
GO:0046483 heterocycle metaboli... 0.151486 27 / 15765
GO:0006725 cellular aromatic co... 0.203361 27 / 16353
GO:0034641 cellular nitrogen co... 0.068659 33 / 18358
GO:0006810 transport 0.014108 21 / 9043
GO:0019637 organophosphate meta... 0.000390 10 / 1918
GO:0072521 purine−containing co... 2.21e−05 8 / 818
GO:0006139 nucleobase−containin... 0.242276 24 / 14713
GO:0044281 small molecule metab... 6.23e−05 21 / 5867
GO:0006811 ion transport 0.019909 10 / 3352
GO:0055085 transmembrane transp... 0.000621 14 / 3654
GO:0006796 phosphate−containing... 0.036651 14 / 5885
GO:0055086 nucleobase−containin... 1.53e−06 11 / 1239
GO:0006812 cation transport 0.032417 7 / 2149
GO:0034220 ion transmembrane tr... 0.014291 8 / 2249
GO:0006753 nucleoside phosphate... 2.29e−05 9 / 1076
GO:0098655 cation transmembrane... 0.007906 7 / 1612
GO:0098660 inorganic ion transm... 0.000497 7 / 981
GO:0009123 nucleoside monophosp... 5.85e−05 7 / 690
GO:0009141 nucleoside triphosph... 0.000187 6 / 584
GO:0009117 nucleotide metabolic... 2.10e−05 9 / 1064
GO:0098662 inorganic cation tra... 0.000260 7 / 880
GO:0009161 ribonucleoside monop... 5.00e−05 7 / 673

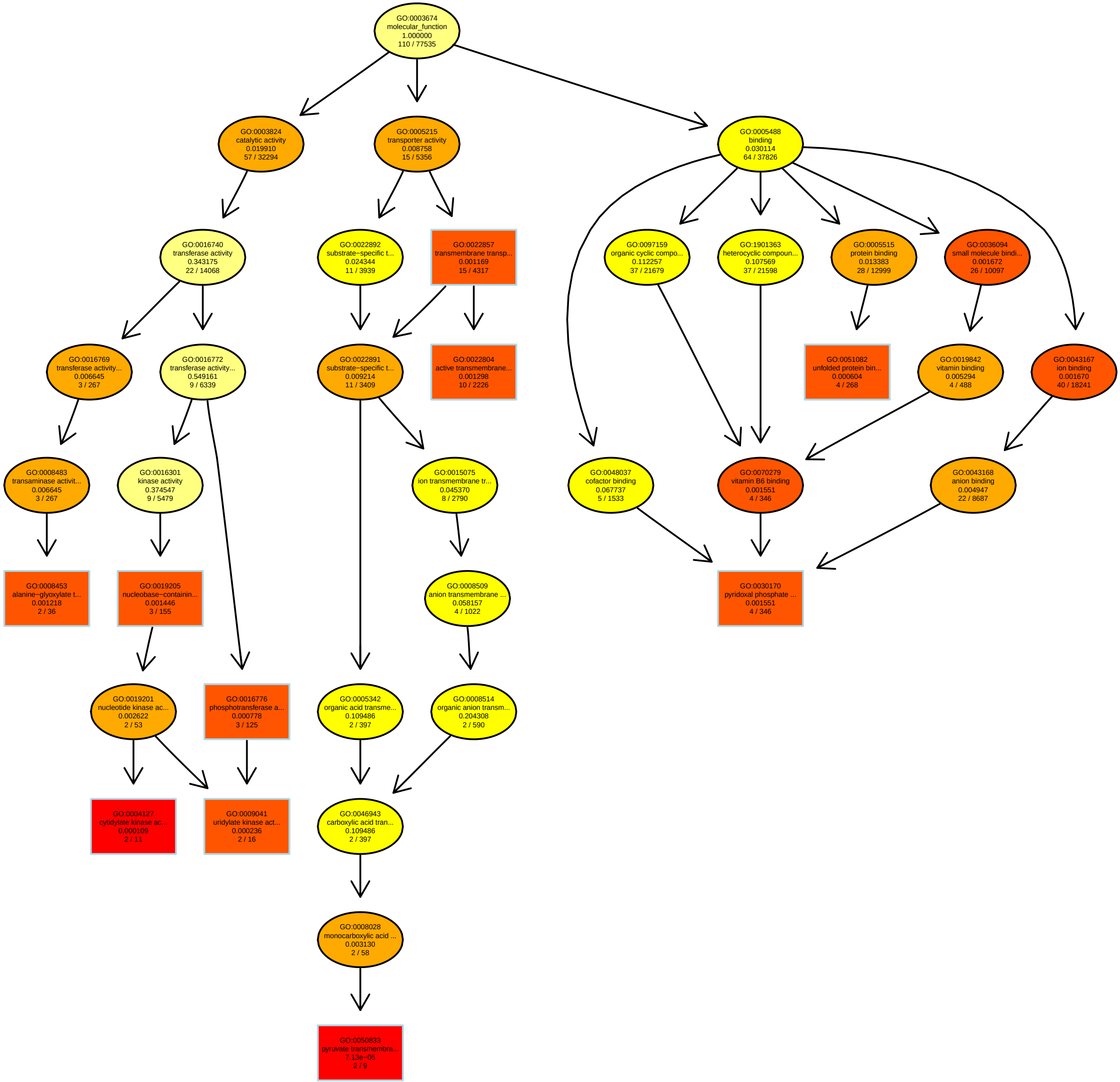

GO:0003674
molecular_function
1.000000
110 / 77535
GO:0003824
catalytic activity
0.019910
57 / 32294
GO:0005215
transporter activity
0.008758
15 / 5356
GO:0005488
binding
0.030114
64 / 37826
GO:0016740
transferase activity
0.343175
22 / 14068
GO:0022892
substrate−specific t...
0.024344
11 / 3939
GO:0022857
transmembrane transp...
0.001169
15 / 4317
GO:0097159
organic cyclic compo...
0.112257
37 / 21679
GO:1901363
heterocyclic compoun...
0.107569
37 / 21598
GO:0005515
protein binding
0.013383
28 / 12999
GO:0036094
small molecule bindi...
0.001672
26 / 10097
GO:0016769
transferase activity...
0.006645
3 / 267
GO:0016772
transferase activity...
0.549161
9 / 6339
GO:0022891
substrate−specific t...
0.009214
11 / 3409
GO:0022804
active transmembrane...
0.001298
10 / 2226
GO:0051082
unfolded protein bin...
0.000604
4 / 268
GO:0019842
vitamin binding
0.005294
4 / 488
GO:0043167
ion binding
0.001670
40 / 18241
GO:0008483
transaminase activit...
0.006645
3 / 267
GO:0016301
kinase activity
0.374547
9 / 5479
GO:0015075
ion transmembrane tr...
0.045370
8 / 2790
GO:0048037
cofactor binding
0.067737
5 / 1533
GO:0070279
vitamin B6 binding
0.001551
4 / 346
GO:0043168
anion binding
0.004947
22 / 8687
GO:0008453
alanine−glyoxylate t...
0.001218
2 / 36
GO:0019205
nucleobase−containin...
0.001446
3 / 155
GO:0008509
anion transmembrane ...
0.058157
4 / 1022
GO:0030170
pyridoxal phosphate ...
0.001551
4 / 346
GO:0019201
nucleotide kinase ac...
0.002622
2 / 53
GO:0016776
phosphotransferase a...
0.000778
3 / 125
GO:0005342
organic acid transme...
0.109486
2 / 397
GO:0008514
organic anion transm...
0.204308
2 / 590
GO:0004127
cytidylate kinase ac...
0.000109
2 / 11
GO:0009041
uridylate kinase act...
0.000236
2 / 16
GO:0046943
carboxylic acid tran...
0.109486
2 / 397
GO:0008028
monocarboxylic acid ...
0.003130
2 / 58
GO:0050833
pyruvate transmembra...
7.13e−05
2 / 9

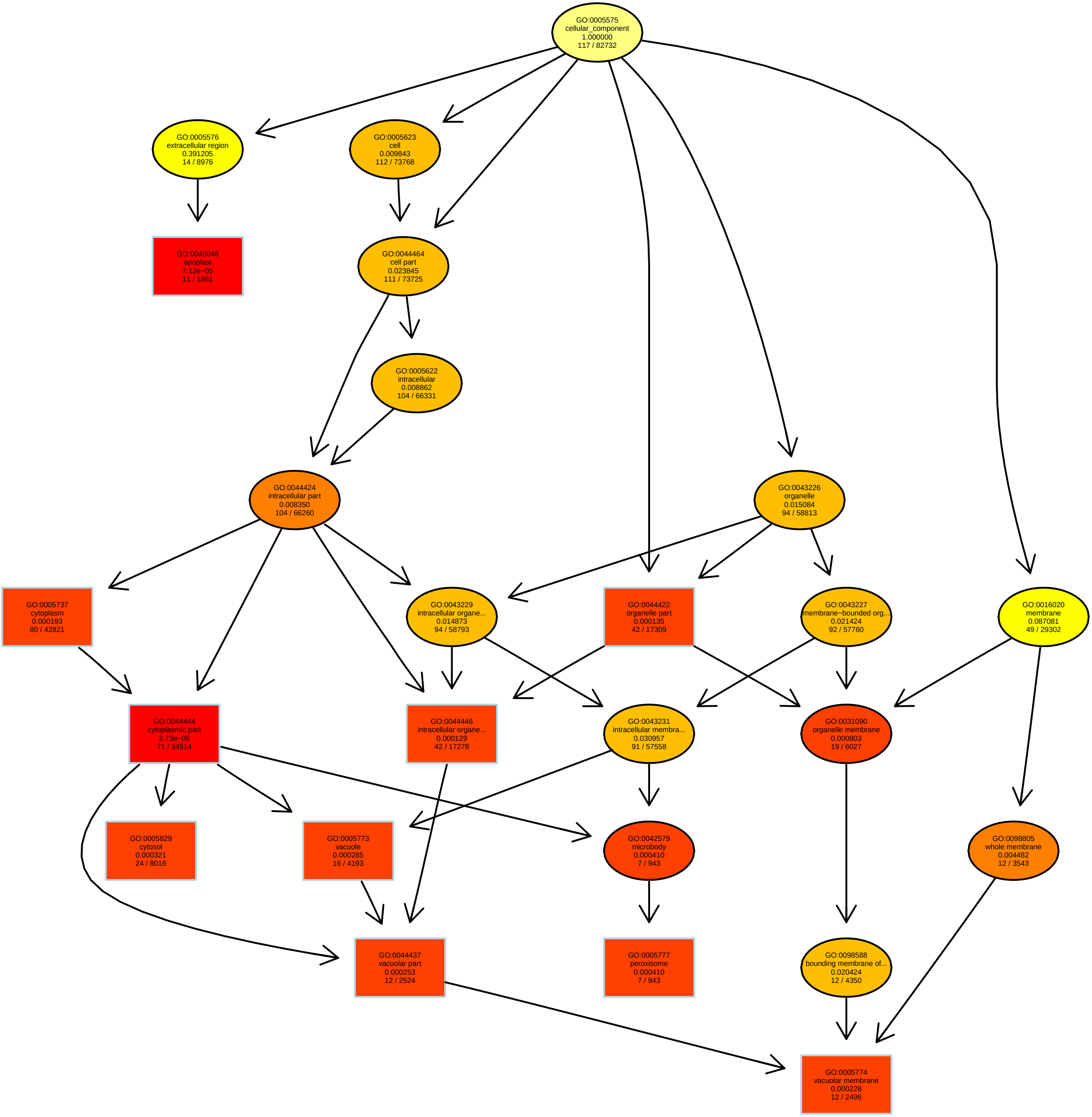
GO:0005575
cellular_component
1.000000
117 / 82732
GO:0005576
extracellular region
0.391205
14 / 8976
GO:0005623
cell
0.009843
112 / 73768
GO:0048046
apoplast
7.12e−05
11 / 1861
GO:0044464
cell part
0.023845
111 / 73725
GO:0005622
intracellular
0.008862
104 / 66331
GO:0044424
intracellular part
0.008350
104 / 66260
GO:0043226
organelle
0.015084
94 / 58813
GO:0005737
cytoplasm
0.000193
80 / 42821
GO:0043229
intracellular organe...
0.014873
94 / 58793
GO:0044422
organelle part
0.000135
42 / 17309
GO:0043227
membrane−bounded org...
0.021424
92 / 57760
GO:0016020
membrane
0.087081
49 / 29302
GO:0044444
cytoplasmic part
2.73e−05
71 / 34514
GO:0044446
intracellular organe...
0.000129
42 / 17278
GO:0043231
intracellular membra...
0.030957
91 / 57558
GO:0031090
organelle membrane
0.000803
19 / 6027
GO:0005829
cytosol
0.000321
24 / 8016
GO:0005773
vacuole
0.000285
16 / 4193
GO:0042579
microbody
0.000410
7 / 943
GO:0098805
whole membrane
0.004482
12 / 3543
GO:0044437
vacuolar part
0.000253
12 / 2524
GO:0005777
peroxisome
0.000410
7 / 943
GO:0098588
bounding membrane of...
0.020424
12 / 4350
GO:0005774
vacuolar membrane
0.000228
12 / 2496
