# Supplementary material for: Differential Alternative Splicing Genes and Isoform Regulation Networks of Rapeseed (Brassica napus L.) Infected with Sclerotinia sclerotiorum
Source: Genes (Basel). 2020 Jul 13;11(7):784. doi: 10.3390/genes11070784 (PMC7397149; doi:10.3390/genes11070784)
Supplement: Supplementary file 1 [file genes-11-00784-s001.zip › Fig. S2 Enriched GO terms of genes in the 85 WGCNA modules.zip!/Fig. S2/CytoNodedarkturquoise-go.pdf]

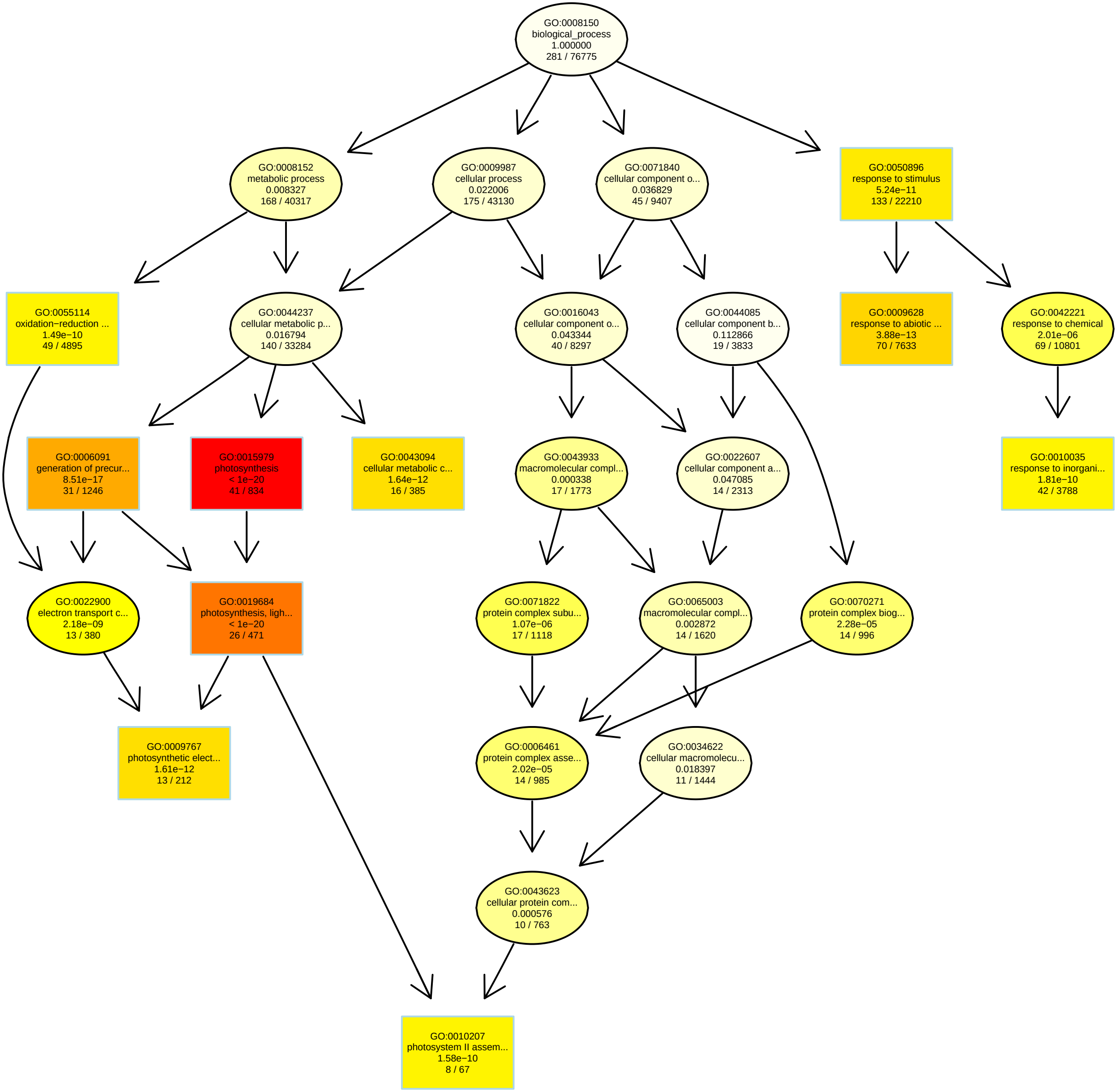

GO:0008150
biological_process
1.000000
281 / 76775
GO:0008152
metabolic process
0.008327
168 / 40317
GO:0009987
cellular process
0.022006
175 / 43130
GO:0071840
cellular component o...
0.036829
45 / 9407
GO:0050896
response to stimulus
5.24e−11
133 / 22210
GO:0055114
oxidation−reduction ...
1.49e−10
49 / 4895
GO:0044237
cellular metabolic p...
0.016794
140 / 33284
GO:0016043
cellular component o...
0.043344
40 / 8297
GO:0044085
cellular component b...
0.112866
19 / 3833
GO:0009628
response to abiotic ...
3.88e−13
70 / 7633
GO:0042221
response to chemical
2.01e−06
69 / 10801
GO:0006091
generation of precur...
8.51e−17
31 / 1246
GO:0015979
photosynthesis
< 1e−20
41 / 834
GO:0043094
cellular metabolic c...
1.64e−12
16 / 385
GO:0043933
macromolecular compl...
0.000338
17 / 1773
GO:0022607
cellular component a...
0.047085
14 / 2313
GO:0010035
response to inorgani...
1.81e−10
42 / 3788
GO:0022900
electron transport c...
2.18e−09
13 / 380
GO:0019684
photosynthesis, ligh...
< 1e−20
26 / 471
GO:0071822
protein complex subu...
1.07e−06
17 / 1118
GO:0065003
macromolecular compl...
0.002872
14 / 1620
GO:0070271
protein complex biog...
2.28e−05
14 / 996
GO:0009767
photosynthetic elect...
1.61e−12
13 / 212
GO:0006461
protein complex asse...
2.02e−05
14 / 985
GO:0034622
cellular macromolecu...
0.018397
11 / 1444
GO:0043623
cellular protein com...
0.000576
10 / 763
GO:0010207
photosystem II assem...
1.58e−10
8 / 67

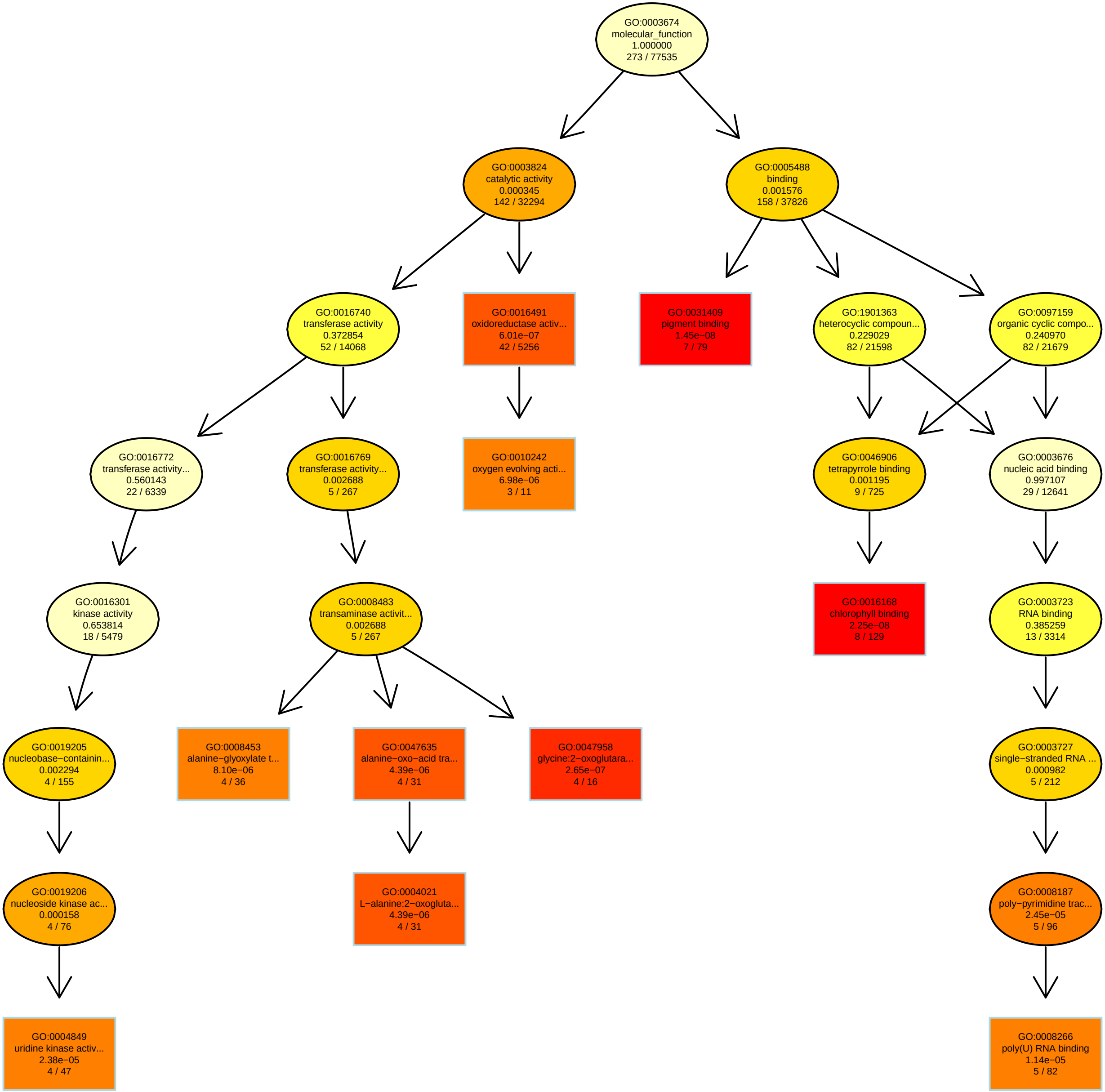
GO:0003674
molecular_function
1.000000
273 / 77535
GO:0003824
catalytic activity
0.000345
142 / 32294
GO:0005488
binding
0.001576
158 / 37826
GO:0016740
transferase activity
0.372854
52 / 14068
GO:0016491
oxidoreductase activ...
6.01e−07
42 / 5256
GO:0031409
pigment binding
1.45e−08
7 / 79
GO:1901363
heterocyclic compoun...
0.229029
82 / 21598
GO:0097159
organic cyclic compo...
0.240970
82 / 21679
GO:0016772
transferase activity...
0.560143
22 / 6339
GO:0016769
transferase activity...
0.002688
5 / 267
GO:0010242
oxygen evolving acti...
6.98e−06
3 / 11
GO:0046906
tetrapyrrole binding
0.001195
9 / 725
GO:0003676
nucleic acid binding
0.997107
29 / 12641
GO:0016301
kinase activity
0.653814
18 / 5479
GO:0008483
transaminase activit...
0.002688
5 / 267
GO:0016168
chlorophyll binding
2.25e−08
8 / 129
GO:0003723
RNA binding
0.385259
13 / 3314
GO:0019205
nucleobase−containin...
0.002294
4 / 155
GO:0008453
alanine−glyoxylate t...
8.10e−06
4 / 36
GO:0047635
alanine−oxo−acid tra...
4.39e−06
4 / 31
GO:0047958
glycine:2−oxoglutara...
2.65e−07
4 / 16
GO:0003727
single−stranded RNA ...
0.000982
5 / 212
GO:0019206
nucleoside kinase ac...
0.000158
4 / 76
GO:0004021
L−alanine:2−oxogluta...
4.39e−06
4 / 31
GO:0008187
poly−pyrimidine trac...
2.45e−05
5 / 96
GO:0004849
uridine kinase activ...
2.38e−05
4 / 47
GO:0008266
poly(U) RNA binding
1.14e−05
5 / 82

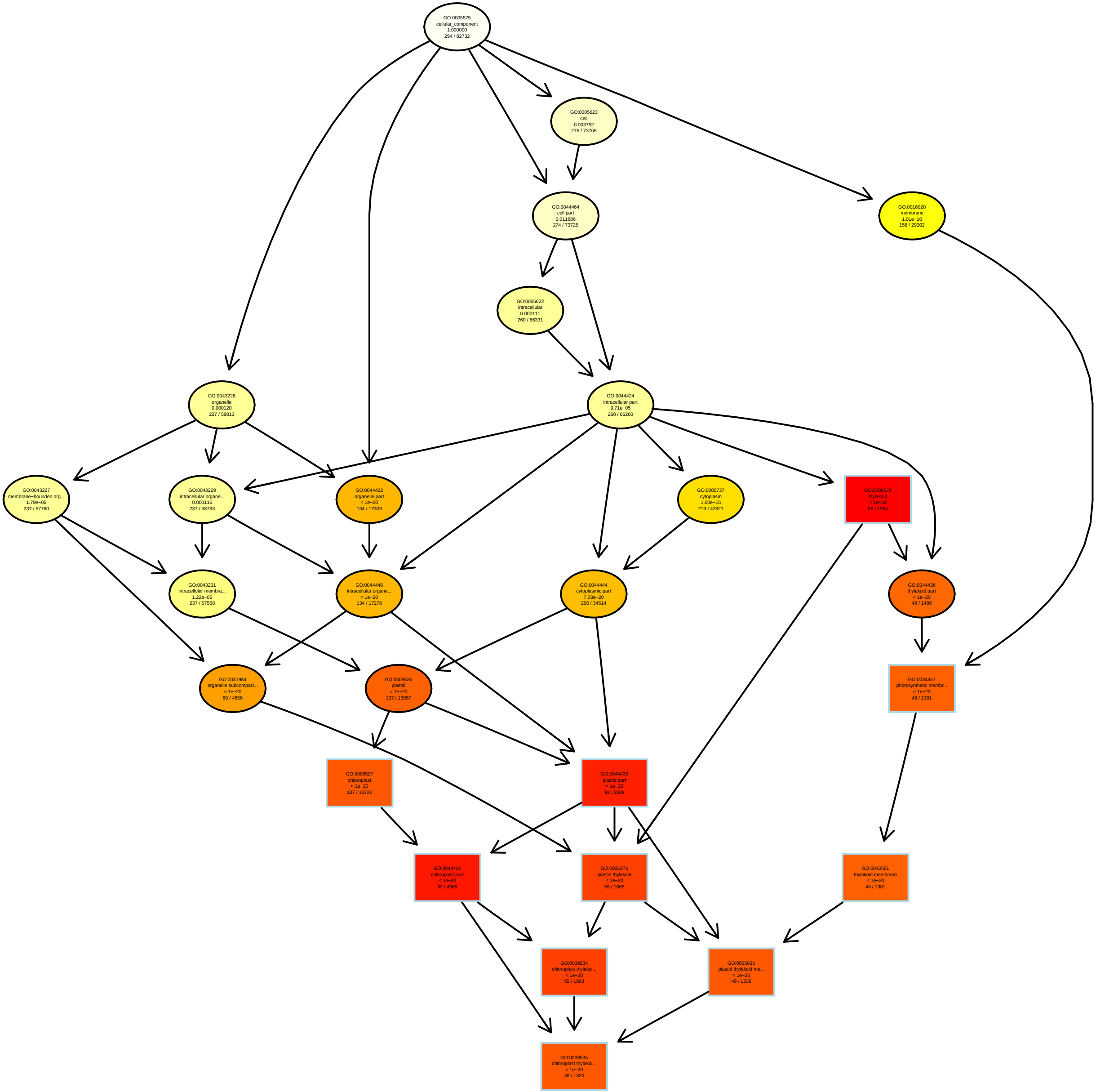
GO:0005575
cellular_component
1.000000
294 / 82732
GO:0005623
cell
0.003752
276 / 73768
GO:0044464
cell part
0.011686
274 / 73725
GO:0016020
membrane
1.01e−10
158 / 29302
GO:0005622
intracellular
0.000111
260 / 66331
GO:0043226
organelle
0.000120
237 / 58813
GO:0044424
intracellular part
9.71e−05
260 / 66260
GO:0043227
membrane−bounded org...
1.79e−05
237 / 57760
GO:0043229
intracellular organe...
0.000116
237 / 58793
GO:0044422
organelle part
< 1e−20
134 / 17309
GO:0005737
cytoplasm
1.00e−15
219 / 42821
GO:0009579
thylakoid
< 1e−20
66 / 1954
GO:0043231
intracellular membra...
1.22e−05
237 / 57558
GO:0044446
intracellular organe...
< 1e−20
134 / 17278
GO:0044444
cytoplasmic part
7.03e−20
200 / 34514
GO:0044436
thylakoid part
< 1e−20
48 / 1489
GO:0031984
organelle subcompart...
< 1e−20
69 / 4656
GO:0009536
plastid
< 1e−20
137 / 13957
GO:0034357
photosynthetic membr...
< 1e−20
48 / 1381
GO:0009507
chloroplast
< 1e−20
137 / 13722
GO:0044435
plastid part
< 1e−20
92 / 5038
GO:0044434
chloroplast part
< 1e−20
92 / 4966
GO:0031976
plastid thylakoid
< 1e−20
55 / 1665
GO:0042651
thylakoid membrane
< 1e−20
48 / 1381
GO:0009534
chloroplast thylakoi...
< 1e−20
55 / 1663
GO:0055035
plastid thylakoid me...
< 1e−20
48 / 1336
GO:0009535
chloroplast thylakoi...
< 1e−20
48 / 1320
