# Supplementary material for: Differential Alternative Splicing Genes and Isoform Regulation Networks of Rapeseed (Brassica napus L.) Infected with Sclerotinia sclerotiorum
Source: Genes (Basel). 2020 Jul 13;11(7):784. doi: 10.3390/genes11070784 (PMC7397149; doi:10.3390/genes11070784)
Supplement: Supplementary file 1 [file genes-11-00784-s001.zip › Fig. S2 Enriched GO terms of genes in the 85 WGCNA modules.zip!/Fig. S2/CytoNodedarkviolet-go.pdf]

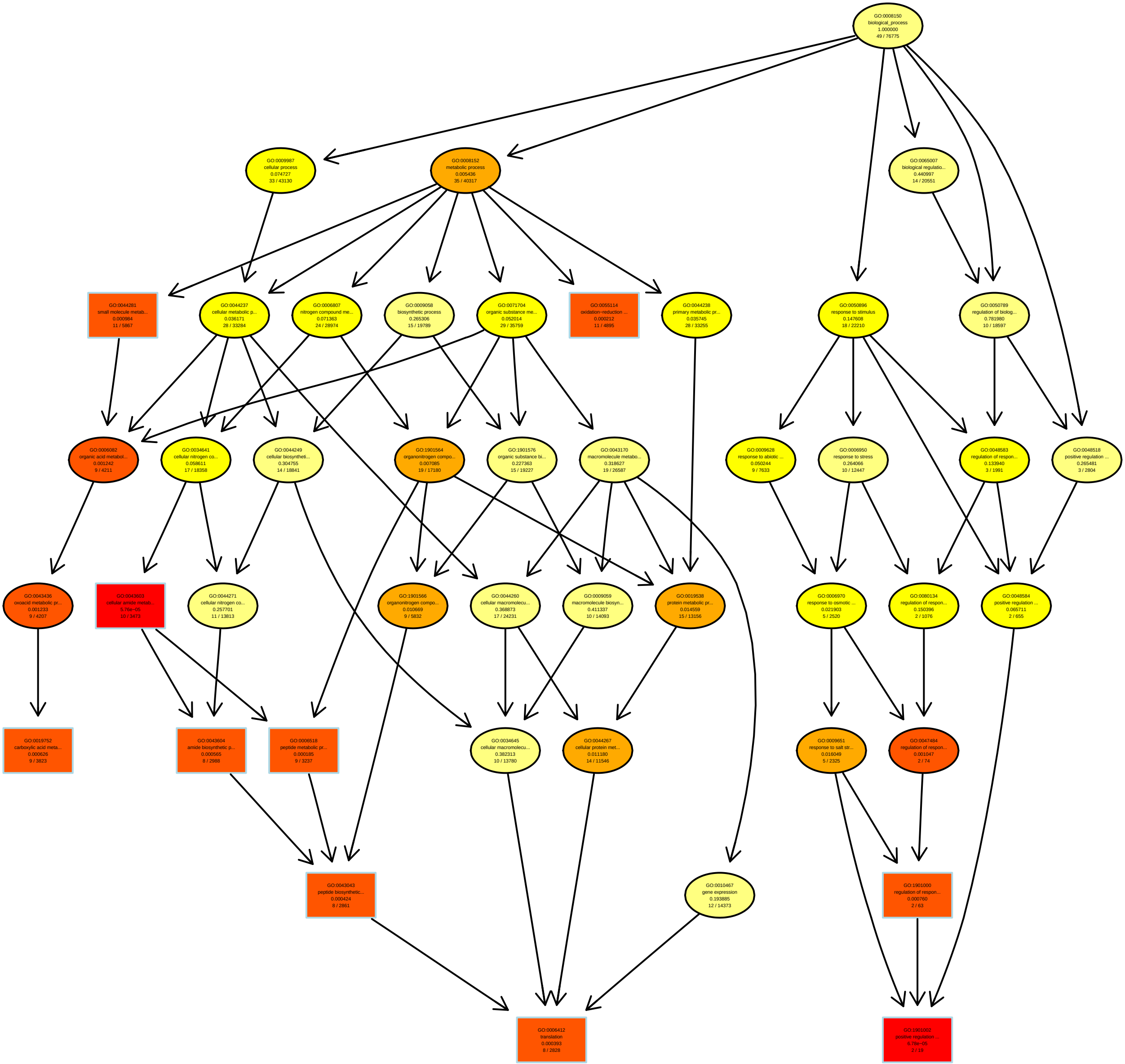

GO:0008150 biological_process 1.000000 49 / 76775
GO:0009987 cellular process 0.074727 33 / 43130
GO:0008152 metabolic process 0.005436 35 / 40317
GO:0065007 biological regulatio... 0.440997 14 / 20551
GO:0044281 small molecule metab... 0.000984 11 / 5867
GO:0044237 cellular metabolic p... 0.036171 28 / 33284
GO:0006807 nitrogen compound me... 0.071363 24 / 28974
GO:0009058 biosynthetic process 0.265306 15 / 19789
GO:0071704 organic substance me... 0.052014 29 / 35759
GO:0055114 oxidation−reduction ... 0.000212 11 / 4895
GO:0044238 primary metabolic pr... 0.035745 28 / 33255
GO:0050896 response to stimulus 0.147608 18 / 22210
GO:0050789 regulation of biolog... 0.781980 10 / 18597
GO:0006082 organic acid metabol... 0.001242 9 / 4211
GO:0034641 cellular nitrogen co... 0.058611 17 / 18358
GO:0044249 cellular biosyntheti... 0.304755 14 / 18841
GO:1901564 organonitrogen compo... 0.007085 19 / 17180
GO:1901576 organic substance bi... 0.227363 15 / 19227
GO:0043170 macromolecule metabo... 0.318627 19 / 26587
GO:0009628 response to abiotic ... 0.050244 9 / 7633
GO:0006950 response to stress 0.264066 10 / 12447
GO:0048583 regulation of respon... 0.133940 3 / 1991
GO:0048518 positive regulation ... 0.265481 3 / 2804
GO:0043436 oxoacid metabolic pr... 0.001233 9 / 4207
GO:0043603 cellular amide metab... 5.78e−05 13 / 3473
GO:0044271 cellular nitrogen co... 0.257701 11 / 13813
GO:1901566 organonitrogen compo... 0.010669 9 / 5832
GO:0044260 cellular macromolecu... 0.368873 17 / 24231
GO:0009059 macromolecule biosyn... 0.411337 10 / 14093
GO:0019538 protein metabolic pr... 0.014559 15 / 13156
GO:0006970 response to osmotic ... 0.021903 5 / 2520
GO:0080134 regulation of respon... 0.150396 2 / 1076
GO:0048584 positive regulation ... 0.065711 2 / 655
GO:0019752 carboxylic acid meta... 0.000626 9 / 3823
GO:0043604 amide biosynthetic p... 0.000565 8 / 2988
GO:0006518 peptide metabolic pr... 0.000185 9 / 3237
GO:0034645 cellular macromolecu... 0.382313 10 / 13780
GO:0044267 cellular protein met... 0.011180 14 / 11546
GO:0009651 response to salt str... 0.016049 5 / 2325
GO:0047484 regulation of respon... 0.001047 2 / 74
GO:0043043 peptide biosynthetic... 0.000424 8 / 2861
GO:0010467 gene expression 0.193885 12 / 14373
GO:1901000 regulation of respon... 0.000760 2 / 63
GO:0006412 translation 0.000393 8 / 2828
GO:1901002 positive regulation... 6.78e−05 2 / 19

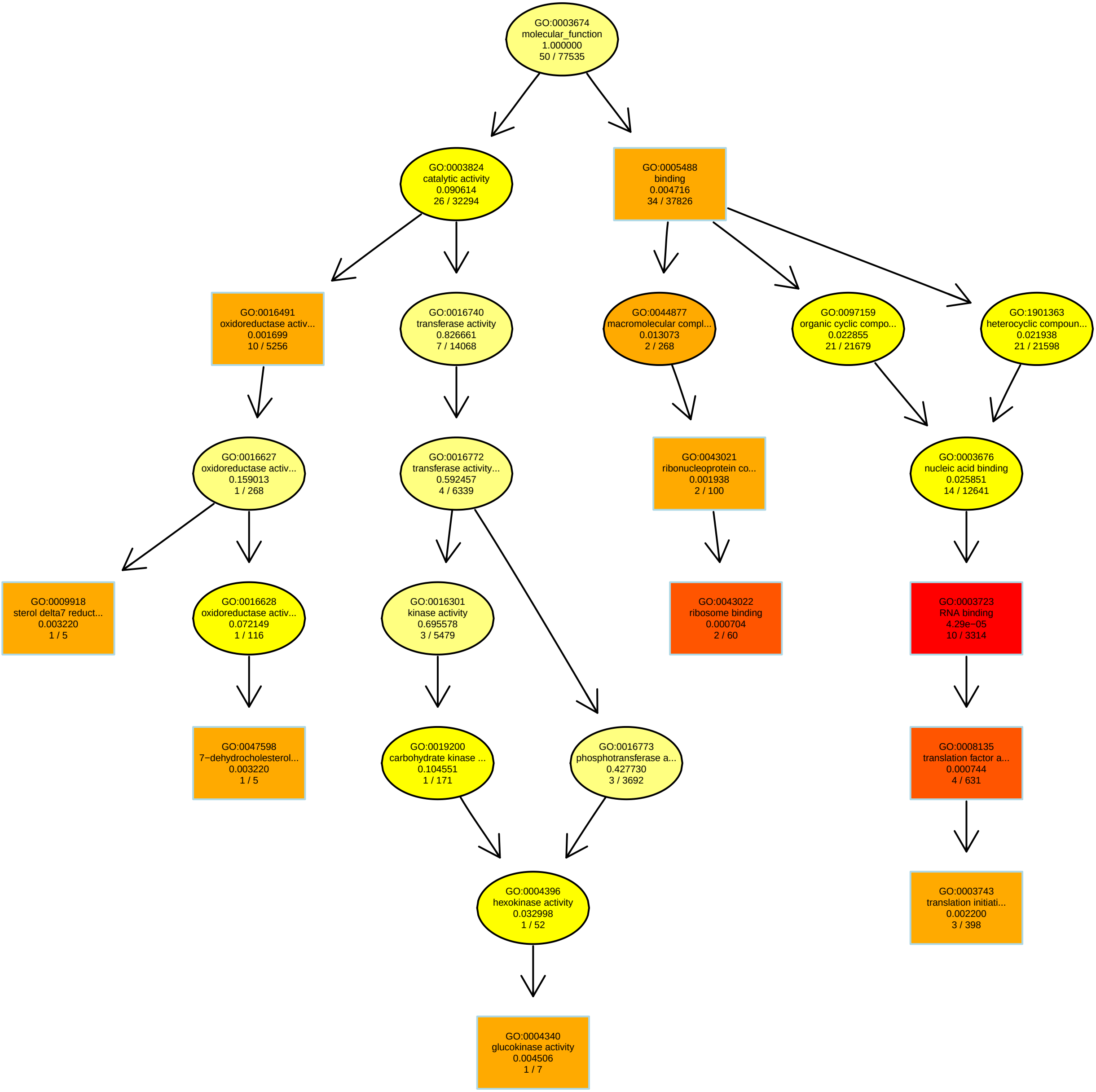

GO:0003674
molecular_function
1.000000
50 / 77535
GO:0003824
catalytic activity
0.090614
26 / 32294
GO:0005488
binding
0.004716
34 / 37826
GO:0016491
oxidoreductase activ...
0.001699
10 / 5256
GO:0016740
transferase activity
0.826661
7 / 14068
GO:0044877
macromolecular compl...
0.013073
2 / 268
GO:0097159
organic cyclic compo...
0.022855
21 / 21679
GO:1901363
heterocyclic compoun...
0.021938
21 / 21598
GO:0016627
oxidoreductase activ...
0.159013
1 / 268
GO:0016772
transferase activity...
0.592457
4 / 6339
GO:0043021
ribonucleoprotein co...
0.001938
2 / 100
GO:0003676
nucleic acid binding
0.025851
14 / 12641
GO:0009918
sterol delta7 reduct...
0.003220
1 / 5
GO:0016628
oxidoreductase activ...
0.072149
1 / 116
GO:0016301
kinase activity
0.695578
3 / 5479
GO:0043022
ribosome binding
0.000704
2 / 60
GO:0003723
RNA binding
4.29e−05
10 / 3314
GO:0047598
7−dehydrocholesterol...
0.003220
1 / 5
GO:0019200
carbohydrate kinase ...
0.104551
1 / 171
GO:0016773
phosphotransferase a...
0.427730
3 / 3692
GO:0008135
translation factor a...
0.000744
4 / 631
GO:0004396
hexokinase activity
0.032998
1 / 52
GO:0003743
translation initiati...
0.002200
3 / 398
GO:0004340
glucokinase activity
0.004506
1 / 7

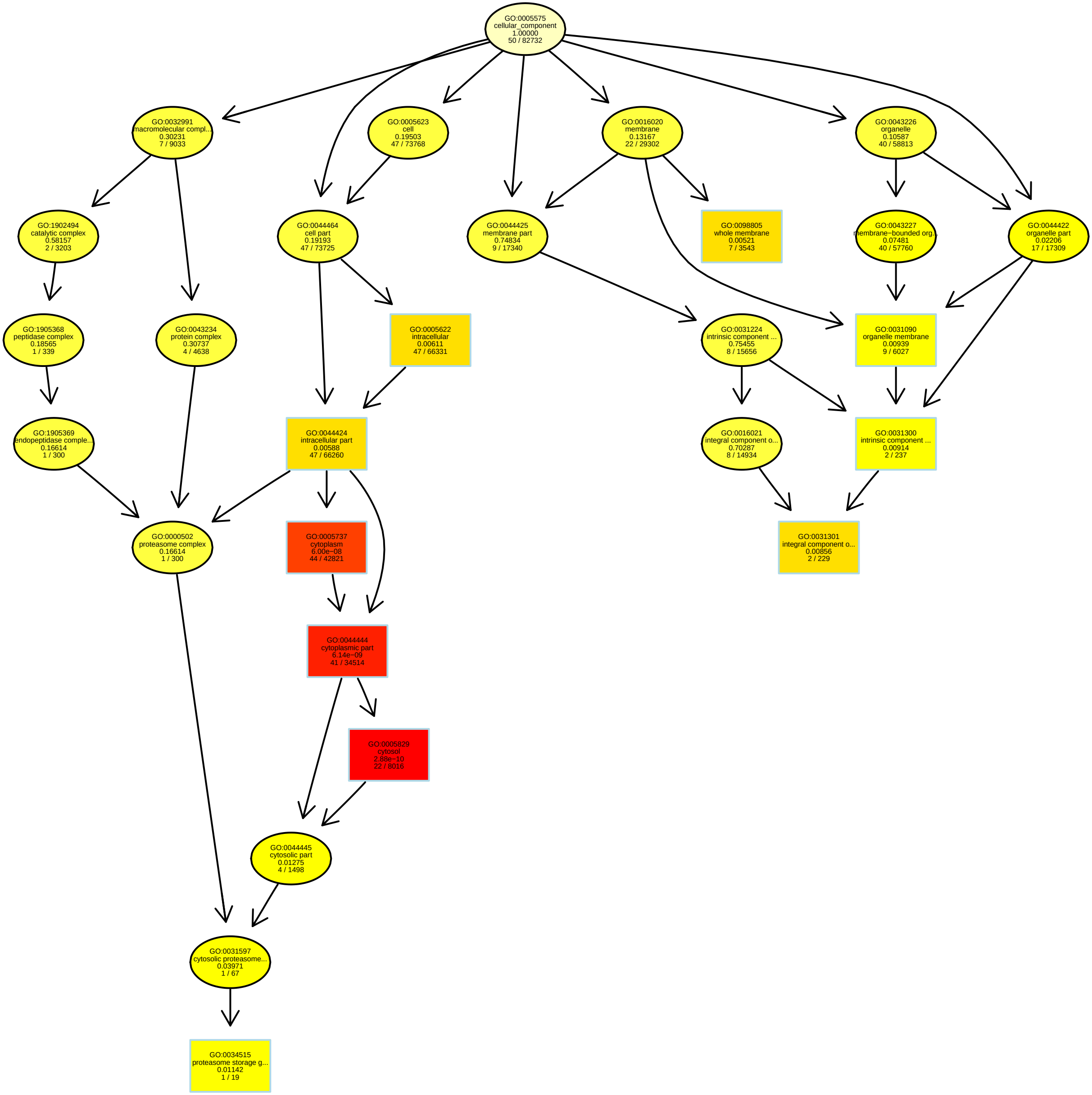
GO:0005575
cellular_component
1.00000
50 / 82732
GO:0032991
macromolecular compl...
0.30231
7 / 9033
GO:0005623
cell
0.19503
47 / 73768
GO:0016020
membrane
0.13167
22 / 29302
GO:0043226
organelle
0.10587
40 / 58813
GO:1902494
catalytic complex
0.58157
2 / 3203
GO:0044464
cell part
0.19193
47 / 73725
GO:0044425
membrane part
0.74834
9 / 17340
GO:0098805
whole membrane
0.00521
7 / 3543
GO:0043227
membrane−bounded org...
0.07481
40 / 57760
GO:0044422
organelle part
0.02206
17 / 17309
GO:1905368
peptidase complex
0.18565
1 / 339
GO:0043234
protein complex
0.30737
4 / 4638
GO:0005622
intracellular
0.00611
47 / 66331
GO:0031224
intrinsic component ...
0.75455
8 / 15656
GO:0031090
organelle membrane
0.00939
9 / 6027
GO:1905369
endopeptidase comple...
0.16614
1 / 300
GO:0044424
intracellular part
0.00588
47 / 66260
GO:0016021
integral component o...
0.70287
8 / 14934
GO:0031300
intrinsic component ...
0.00914
2 / 237
GO:0000502
proteasome complex
0.16614
1 / 300
GO:0005737
cytoplasm
6.00e−08
44 / 42821
GO:0031301
integral component o...
0.00856
2 / 229
GO:0044444
cytoplasmic part
6.14e−09
41 / 34514
GO:0005829
cytosol
2.88e−10
22 / 8016
GO:0044445
cytosolic part
0.01275
4 / 1498
GO:0031597
cytosolic proteasome...
0.03971
1 / 67
GO:0034515
proteasome storage g...
0.01142
1 / 19
