# Supplementary material for: Differential Alternative Splicing Genes and Isoform Regulation Networks of Rapeseed (Brassica napus L.) Infected with Sclerotinia sclerotiorum
Source: Genes (Basel). 2020 Jul 13;11(7):784. doi: 10.3390/genes11070784 (PMC7397149; doi:10.3390/genes11070784)
Supplement: Supplementary file 1 [file genes-11-00784-s001.zip › Fig. S2 Enriched GO terms of genes in the 85 WGCNA modules.zip!/Fig. S2/CytoNodefirebrick4-go.pdf]

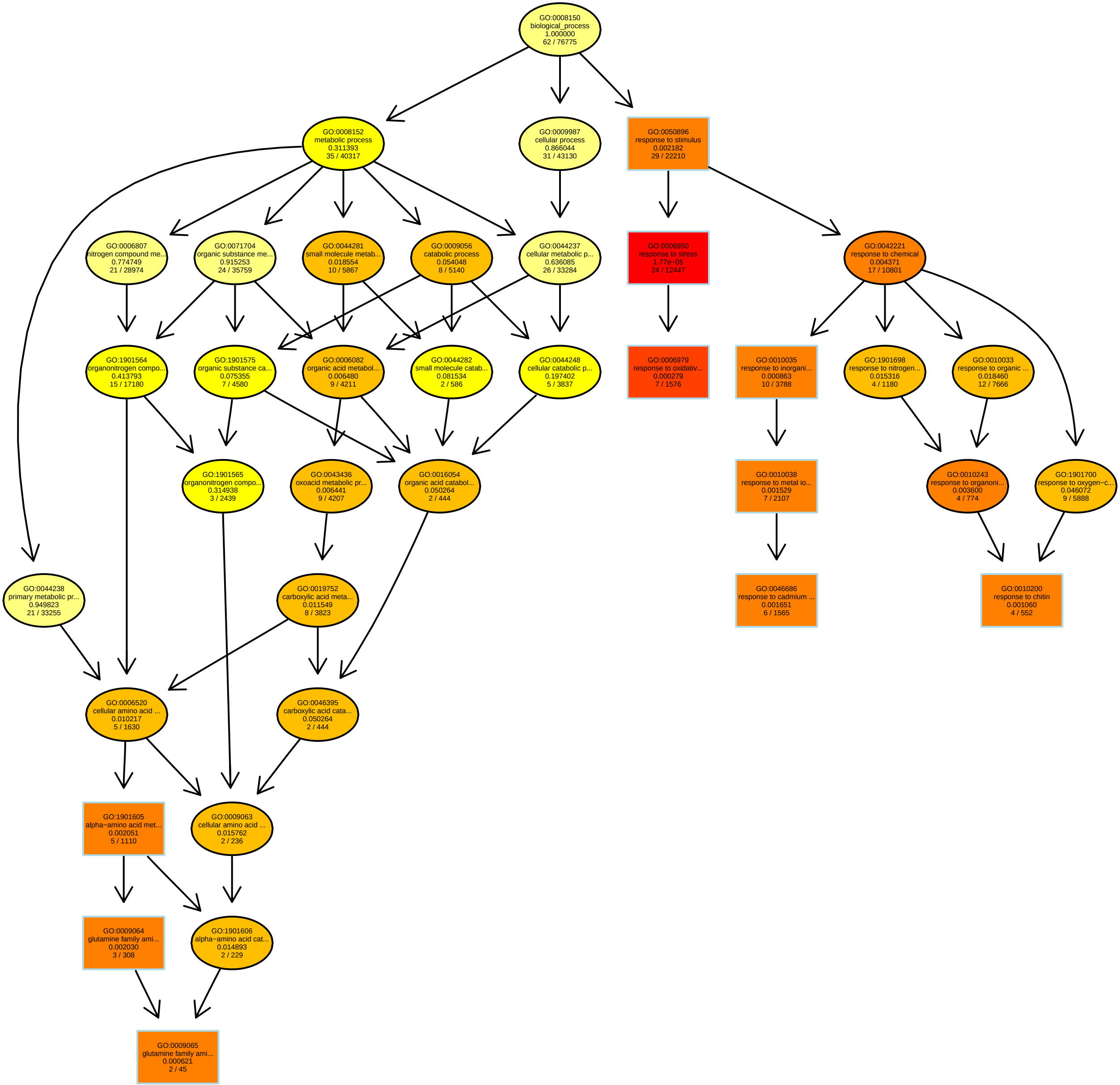
GO:0008150
biological_process
1.000000
62 / 76775
GO:0008152
metabolic process
0.311393
35 / 40317
GO:0009987
cellular process
0.866044
31 / 43130
GO:0050896
response to stimulus
0.002182
29 / 22210
GO:0006807
nitrogen compound me...
0.774749
21 / 28974
GO:0071704
organic substance me...
0.915253
24 / 35759
GO:0044281
small molecule metab...
0.018554
10 / 5867
GO:0009056
catabolic process
0.054048
8 / 5140
GO:0044237
cellular metabolic p...
0.636085
26 / 33284
GO:0006950
response to stress
1.77e−05
24 / 12447
GO:0042221
response to chemical
0.004371
17 / 10801
GO:1901564
organonitrogen compo...
0.413793
15 / 17180
GO:1901575
organic substance ca...
0.075355
7 / 4580
GO:0006082
organic acid metabol...
0.006480
9 / 4211
GO:0044282
small molecule catab...
0.081534
2 / 586
GO:0044248
cellular catabolic p...
0.197402
5 / 3837
GO:0006979
response to oxidativ...
0.000279
7 / 1576
GO:0010035
response to inorgani...
0.000863
10 / 3788
GO:1901698
response to nitrogen...
0.015316
4 / 1180
GO:0010033
response to organic ...
0.018460
12 / 7666
GO:1901565
organonitrogen compo...
0.314938
3 / 2439
GO:0043436
oxoacid metabolic pr...
0.006441
9 / 4207
GO:0016054
organic acid catabol...
0.050264
2 / 444
GO:0010038
response to metal io...
0.001529
7 / 2107
GO:0010243
response to organoni...
0.003600
4 / 774
GO:1901700
response to oxygen−c...
0.046072
9 / 5888
GO:0044238
primary metabolic pr...
0.949823
21 / 33255
GO:0019752
carboxylic acid meta...
0.011549
8 / 3823
GO:0046686
response to cadmium ...
0.001651
6 / 1565
GO:0010200
response to chitin
0.001060
4 / 552
GO:0006520
cellular amino acid ...
0.010217
5 / 1630
GO:0046395
carboxylic acid cata...
0.050264
2 / 444
GO:1901605
alpha−amino acid met...
0.002051
5 / 1110
GO:0009063
cellular amino acid ...
0.015762
2 / 236
GO:0009064
glutamine family ami...
0.002030
3 / 308
GO:1901606
alpha−amino acid cat...
0.014893
2 / 229
GO:0009065
glutamine family ami...
0.000621
2 / 45

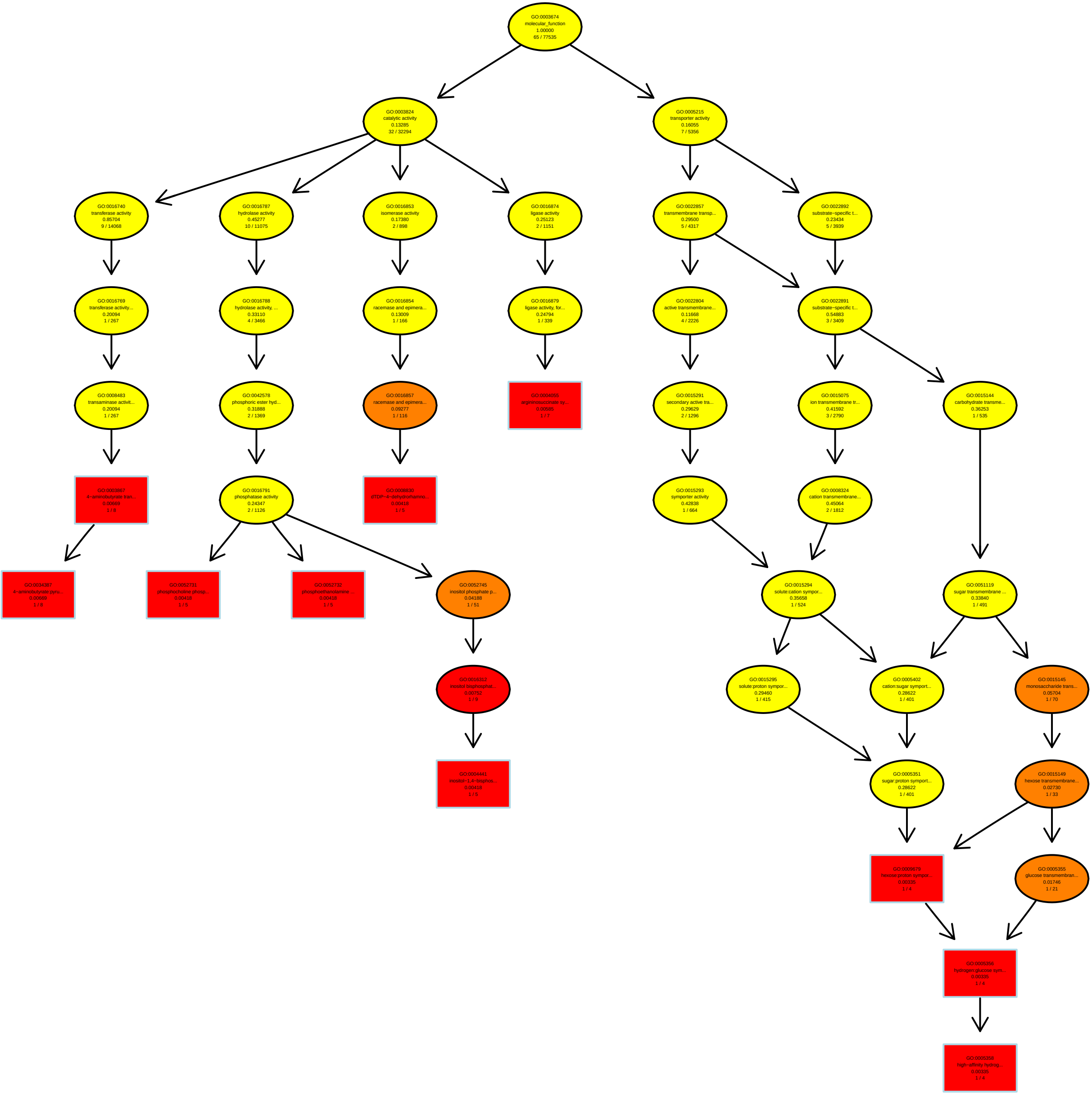
GO:0003674 molecular_function 1.00000 65 / 77535
GO:0003824 catalytic activity 0.13285 32 / 32294
GO:0005215 transporter activity 0.16055 7 / 5356
GO:0016740 transferase activity 0.85704 9 / 14068
GO:0016787 hydrolase activity 0.45277 10 / 11075
GO:0016853 isomerase activity 0.17380 2 / 898
GO:0016874 ligase activity 0.25123 2 / 1151
GO:0022857 transmembrane transp... 0.29500 5 / 4317
GO:0022892 substrate−specific t... 0.23434 5 / 3939
GO:0016769 transferase activity... 0.20094 1 / 267
GO:0016788 hydrolase activity, ... 0.33110 4 / 3466
GO:0016854 racemase and epimera... 0.13009 1 / 166
GO:0016879 ligase activity, for... 0.24794 1 / 339
GO:0022804 active transmembrane... 0.11668 4 / 2226
GO:0022891 substrate−specific t... 0.54883 3 / 3409
GO:0008483 transaminase activit... 0.20094 1 / 267
GO:0042578 phosphoric ester hyd... 0.31888 2 / 1369
GO:0016857 racemase and epimera... 0.09277 1 / 116
GO:0004055 argininosuccinate sy... 0.00585 1 / 7
GO:0015291 secondary active tra... 0.29629 2 / 1296
GO:0015075 ion transmembrane tr... 0.41592 3 / 2790
GO:0015144 carbohydrate transme... 0.36253 1 / 535
GO:0003867 4−aminobutyrate tran... 0.00669 1 / 8
GO:0016791 phosphatase activity 0.24347 2 / 1126
GO:0008830 dTDP−4−dehydrorhamno... 0.00418 1 / 5
GO:0015293 symporter activity 0.42838 1 / 664
GO:0008324 cation transmembrane... 0.45064 2 / 1812
GO:0034387 4−aminobutyrate:pyru... 0.00669 1 / 8
GO:0052731 phosphocholine phosp... 0.00418 1 / 5
GO:0052732 phosphoethanolamine ... 0.00418 1 / 5
GO:0052745 inositol phosphate p... 0.04188 1 / 51
GO:0015294 solute:cation sympor... 0.35658 1 / 524
GO:0051119 sugar transmembrane ... 0.33840 1 / 491
GO:0016312 inositol bisphosphat... 0.00752 1 / 9
GO:0015295 solute:proton sympor... 0.29460 1 / 415
GO:0005402 cation:sugar sympor... 0.28622 1 / 401
GO:0015145 monosaccharide trans... 0.05704 1 / 70
GO:0004441 inositol−1,4−bisphos... 0.00418 1 / 5
GO:0005351 sugar:proton symport... 0.28622 1 / 401
GO:0015149 hexose transmembrane... 0.02730 1 / 33
GO:0009679 hexose:proton sympor... 0.00335 1 / 4
GO:0005355 glucose transmembran... 0.01746 1 / 21
GO:0005356 hydrogen:glucose sym... 0.00335 1 / 4
GO:0005358 high−affinity hydrog... 0.00335 1 / 4

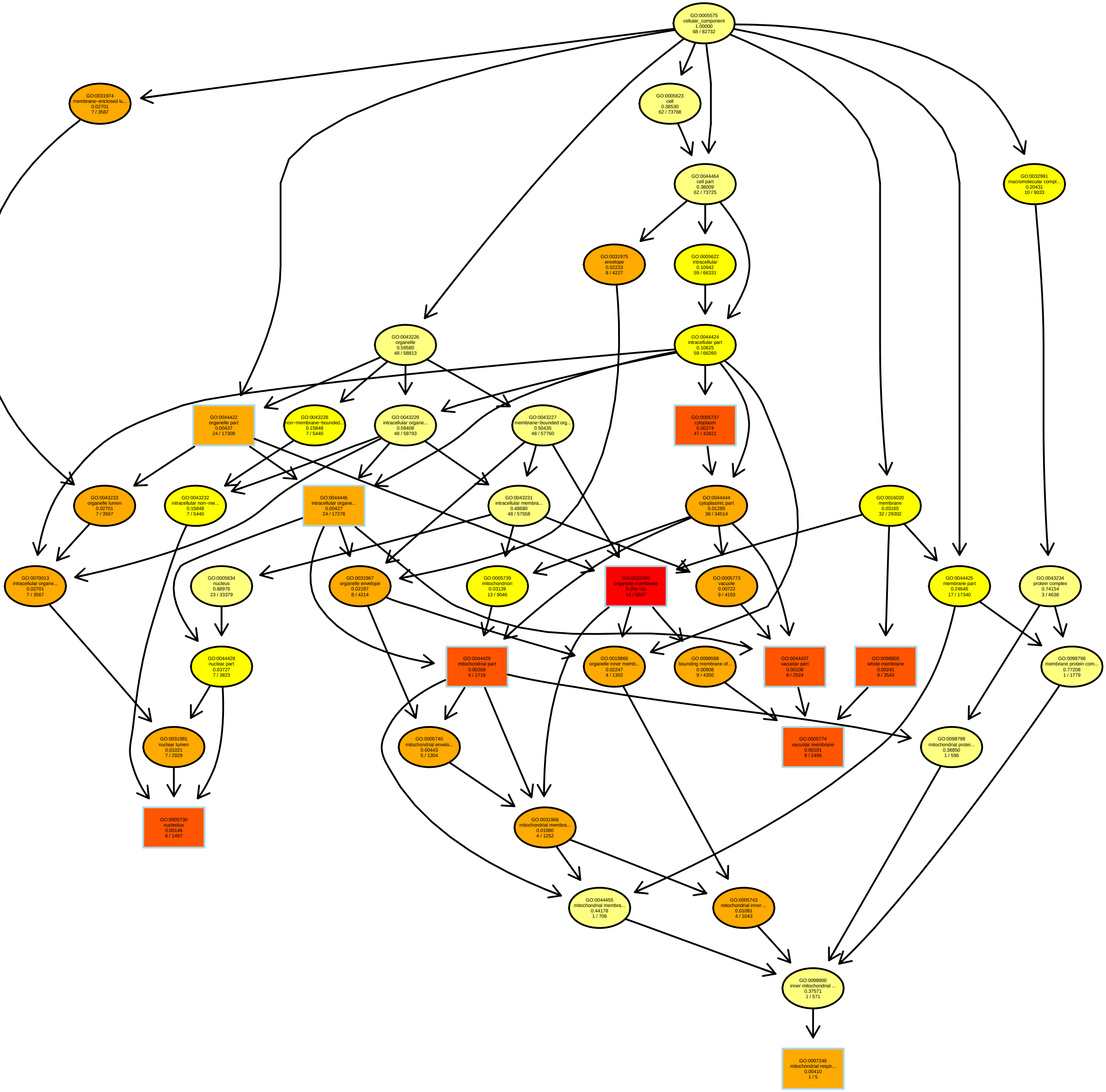
GO:0005575
cellular_component
1.00000
68 / 82732
GO:0031974
membrane−enclosed lu...
0.02701
7 / 3567
GO:0005623
cell
0.38530
62 / 73768
GO:0044464
cell part
0.38009
62 / 73725
GO:0032991
macromolecular compl...
0.20431
10 / 9033
GO:0031975
envelope
0.02233
8 / 4227
GO:0005622
intracellular
0.10942
59 / 66331
GO:0043226
organelle
0.59580
48 / 58813
GO:0044424
intracellular part
0.10625
59 / 66260
GO:0044422
organelle part
0.00437
24 / 17309
GO:0043228
non−membrane−bounded...
0.15848
7 / 5440
GO:0043229
intracellular organe...
0.59408
48 / 58793
GO:0043227
membrane−bounded org...
0.50435
48 / 57760
GO:0005737
cytoplasm
0.00274
47 / 42821
GO:0043233
organelle lumen
0.02701
7 / 3567
GO:0043232
intracellular non−me...
0.15848
7 / 5440
GO:0044446
intracellular organe...
0.00427
24 / 17278
GO:0043231
intracellular membra...
0.48680
48 / 57558
GO:0044444
cytoplasmic part
0.01285
38 / 34514
GO:0016020
membrane
0.03165
32 / 29302
GO:0070013
intracellular organe...
0.02701
7 / 3567
GO:0005634
nucleus
0.88976
23 / 33379
GO:0031967
organelle envelope
0.02197
8 / 4214
GO:0005739
mitochondrion
0.03139
13 / 9046
GO:0031090
organelle membrane
9.25e−05
15 / 6027
GO:0005773
vacuole
0.00722
9 / 4193
GO:0044425
membrane part
0.24646
17 / 17340
GO:0043234
protein complex
0.74154
3 / 4638
GO:0044428
nuclear part
0.03727
7 / 3823
GO:0044429
mitochondrial part
0.00289
6 / 1716
GO:0019866
organelle inner memb...
0.02247
4 / 1302
GO:0098588
bounding membrane of...
0.00908
9 / 4350
GO:0044437
vacuolar part
0.00108
8 / 2524
GO:0098805
whole membrane
0.00241
9 / 3543
GO:0098796
membrane protein com...
0.77208
1 / 1779
GO:0031981
nuclear lumen
0.01021
7 / 2929
GO:0005740
mitochondrial envelo...
0.00443
5 / 1304
GO:0098798
mitochondrial protei...
0.38850
1 / 596
GO:0005774
vacuolar membrane
0.00101
8 / 2496
GO:0005730
nucleolus
0.00146
6 / 1497
GO:0031966
mitochondrial membra...
0.01980
4 / 1252
GO:0044455
mitochondrial membra...
0.44178
1 / 706
GO:0005743
mitochondrial inner ...
0.01081
4 / 1043
GO:0098800
inner mitochondrial ...
0.37571
1 / 571
GO:0097249
mitochondrial respir...
0.00410
1 / 5
