# Supplementary material for: Differential Alternative Splicing Genes and Isoform Regulation Networks of Rapeseed (Brassica napus L.) Infected with Sclerotinia sclerotiorum
Source: Genes (Basel). 2020 Jul 13;11(7):784. doi: 10.3390/genes11070784 (PMC7397149; doi:10.3390/genes11070784)
Supplement: Supplementary file 1 [file genes-11-00784-s001.zip › Fig. S2 Enriched GO terms of genes in the 85 WGCNA modules.zip!/Fig. S2/CytoNodefloralwhite-go.pdf]

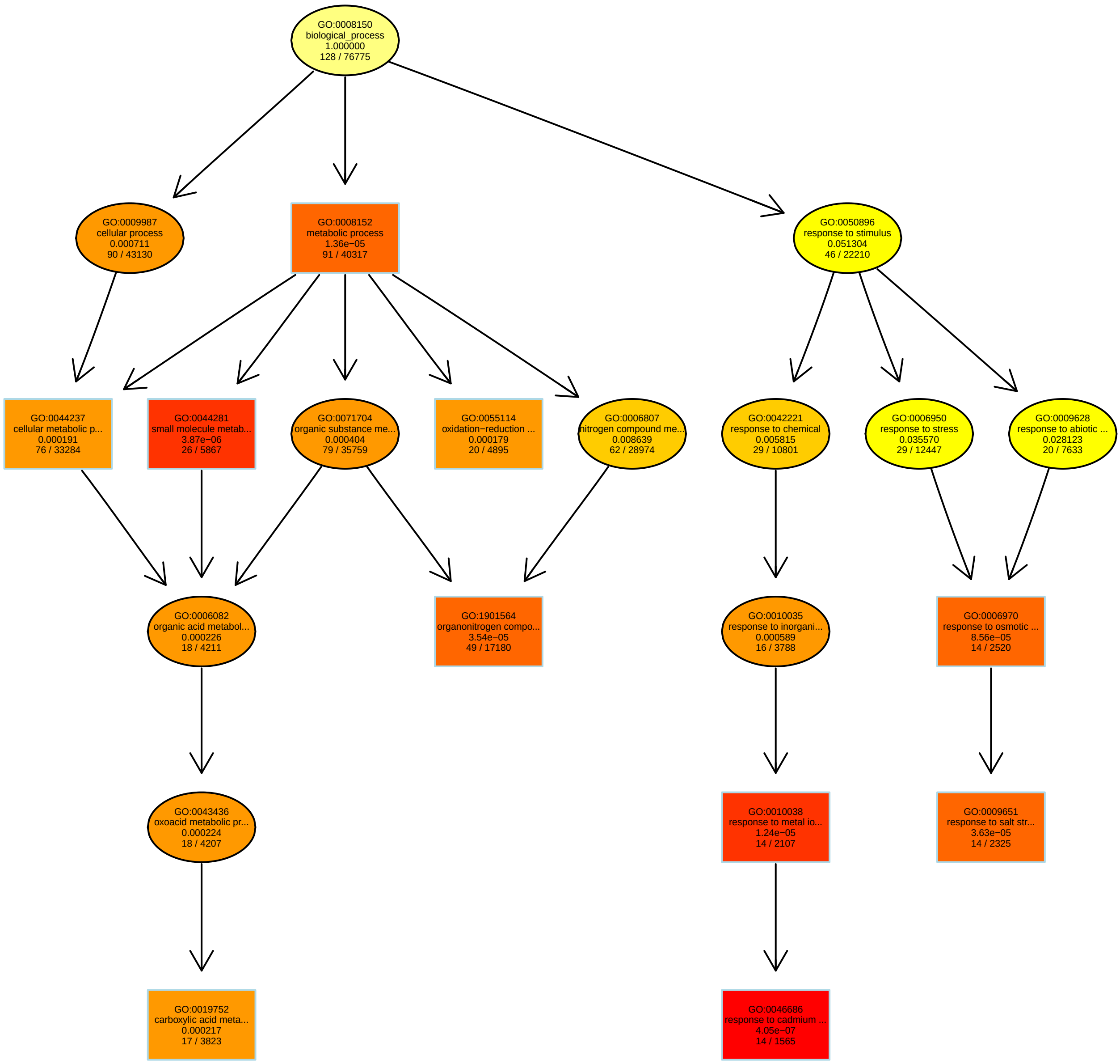
GO:0008150
biological_process
1.000000
128 / 76775
GO:0009987
cellular process
0.000711
90 / 43130
GO:0008152
metabolic process
1.36e−05
91 / 40317
GO:0050896
response to stimulus
0.051304
46 / 22210
GO:0044237
cellular metabolic p...
0.000191
76 / 33284
GO:0044281
small molecule metab...
3.87e−06
26 / 5867
GO:0071704
organic substance me...
0.000404
79 / 35759
GO:0055114
oxidation−reduction ...
0.000179
20 / 4895
GO:0006807
nitrogen compound me...
0.008639
62 / 28974
GO:0042221
response to chemical
0.005815
29 / 10801
GO:0006950
response to stress
0.035570
29 / 12447
GO:0009628
response to abiotic ...
0.028123
20 / 7633
GO:0006082
organic acid metabol...
0.000226
18 / 4211
GO:1901564
organonitrogen compo...
3.54e−05
49 / 17180
GO:0010035
response to inorgani...
0.000589
16 / 3788
GO:0006970
response to osmotic ...
8.56e−05
14 / 2520
GO:0043436
oxoacid metabolic pr...
0.000224
18 / 4207
GO:0010038
response to metal io...
1.24e−05
14 / 2107
GO:0009651
response to salt str...
3.63e−05
14 / 2325
GO:0019752
carboxylic acid meta...
0.000217
17 / 3823
GO:0046686
response to cadmium ...
4.05e−07
14 / 1565

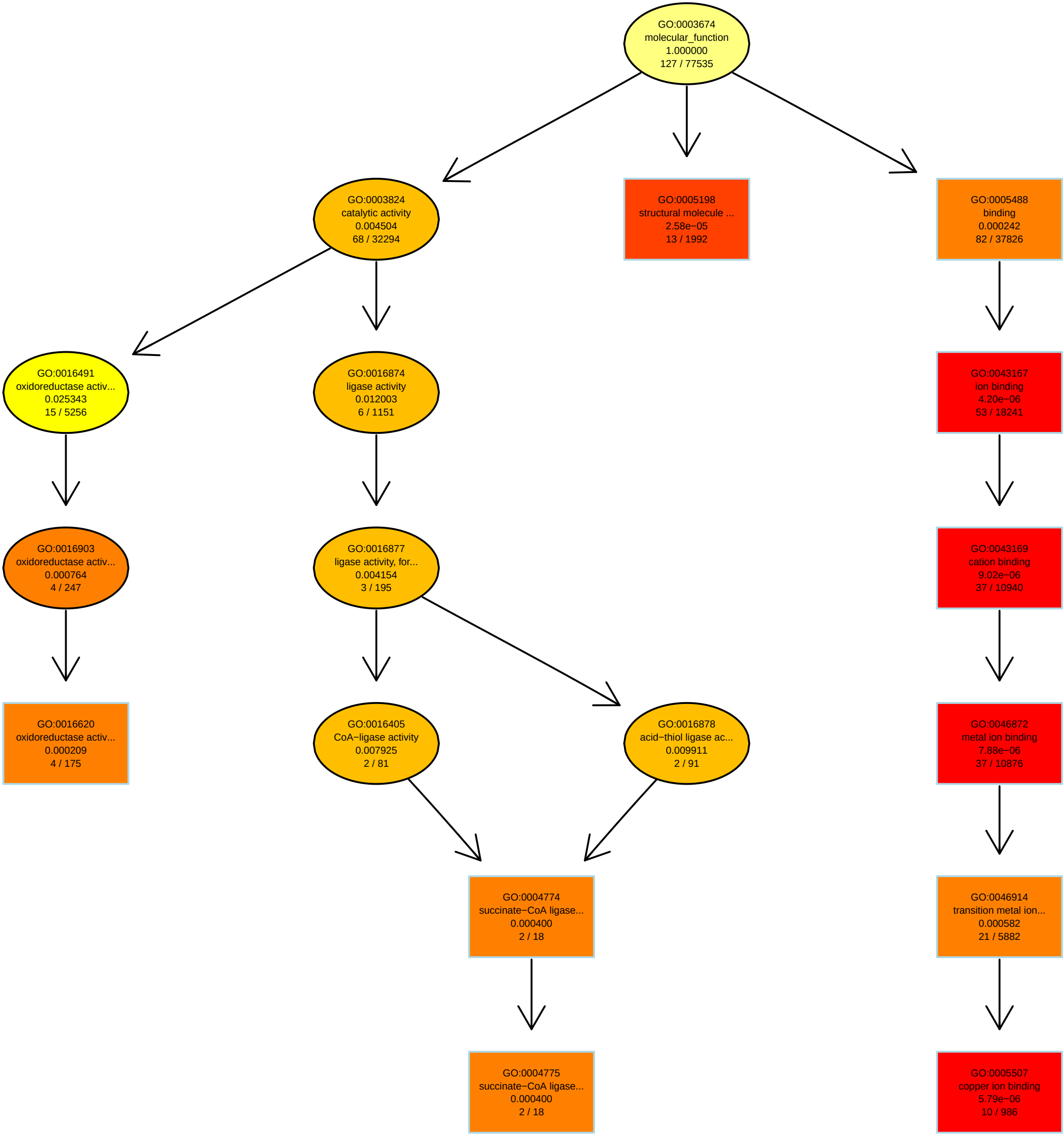

GO:0003674
molecular_function
1.000000
127 / 77535
GO:0003824
catalytic activity
0.004504
68 / 32294
GO:0005198
structural molecule ...
2.58e−05
13 / 1992
GO:0005488
binding
0.000242
82 / 37826
GO:0016491
oxidoreductase activ...
0.025343
15 / 5256
GO:0016874
ligase activity
0.012003
6 / 1151
GO:0043167
ion binding
4.20e−06
53 / 18241
GO:0016903
oxidoreductase activ...
0.000764
4 / 247
GO:0016877
ligase activity, for...
0.004154
3 / 195
GO:0043169
cation binding
9.02e−06
37 / 10940
GO:0016620
oxidoreductase activ...
0.000209
4 / 175
GO:0016405
CoA−ligase activity
0.007925
2 / 81
GO:0016878
acid−thiol ligase ac...
0.009911
2 / 91
GO:0046872
metal ion binding
7.88e−06
37 / 10876
GO:0004774
succinate−CoA ligase...
0.000400
2 / 18
GO:0046914
transition metal ion...
0.000582
21 / 5882
GO:0004775
succinate−CoA ligase...
0.000400
2 / 18
GO:0005507
copper ion binding
5.79e−06
10 / 986

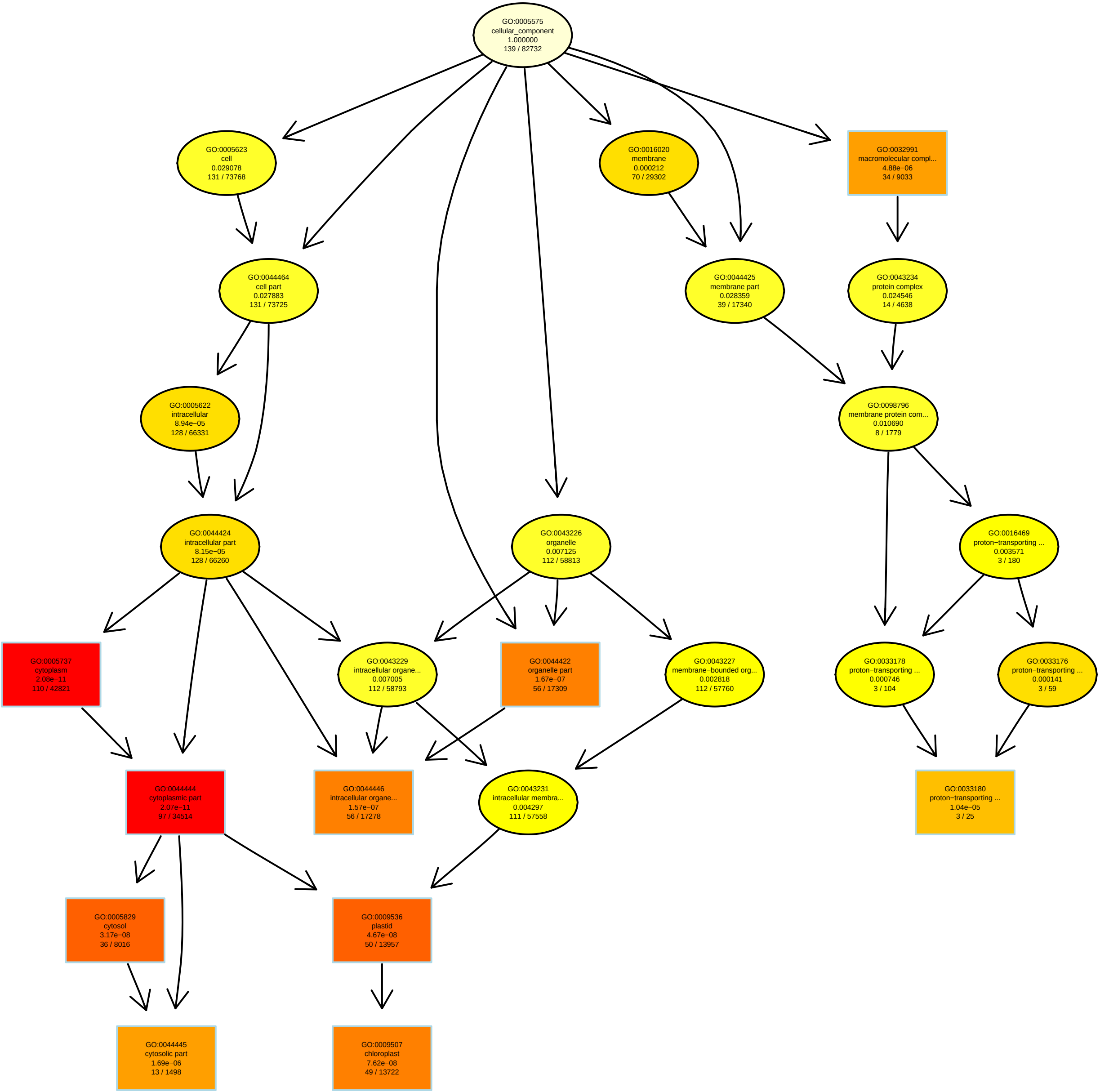
GO:0005575
cellular_component
1.000000
139 / 82732
GO:0005623
cell
0.029078
131 / 73768
GO:0016020
membrane
0.000212
70 / 29302
GO:0032991
macromolecular compl...
4.88e−06
34 / 9033
GO:0044464
cell part
0.027883
131 / 73725
GO:0044425
membrane part
0.028359
39 / 17340
GO:0043234
protein complex
0.024546
14 / 4638
GO:0005622
intracellular
8.94e−05
128 / 66331
GO:0098796
membrane protein com...
0.010690
8 / 1779
GO:0044424
intracellular part
8.15e−05
128 / 66260
GO:0043226
organelle
0.007125
112 / 58813
GO:0016469
proton−transporting ...
0.003571
3 / 180
GO:0005737
cytoplasm
2.08e−11
110 / 42821
GO:0043229
intracellular organe...
0.007005
112 / 58793
GO:0044422
organelle part
1.67e−07
56 / 17309
GO:0043227
membrane−bounded org...
0.002818
112 / 57760
GO:0033178
proton−transporting ...
0.000746
3 / 104
GO:0033176
proton−transporting ...
0.000141
3 / 59
GO:0044444
cytoplasmic part
2.07e−11
97 / 34514
GO:0044446
intracellular organe...
1.57e−07
56 / 17278
GO:0043231
intracellular membra...
0.004297
111 / 57558
GO:0033180
proton−transporting ...
1.04e−05
3 / 25
GO:0005829
cytosol
3.17e−08
36 / 8016
GO:0009536
plastid
4.67e−08
50 / 13957
GO:0044445
cytosolic part
1.69e−06
13 / 1498
GO:0009507
chloroplast
7.62e−08
49 / 13722
