# Supplementary material for: Differential Alternative Splicing Genes and Isoform Regulation Networks of Rapeseed (Brassica napus L.) Infected with Sclerotinia sclerotiorum
Source: Genes (Basel). 2020 Jul 13;11(7):784. doi: 10.3390/genes11070784 (PMC7397149; doi:10.3390/genes11070784)
Supplement: Supplementary file 1 [file genes-11-00784-s001.zip › Fig. S2 Enriched GO terms of genes in the 85 WGCNA modules.zip!/Fig. S2/CytoNodegreen-go.pdf]

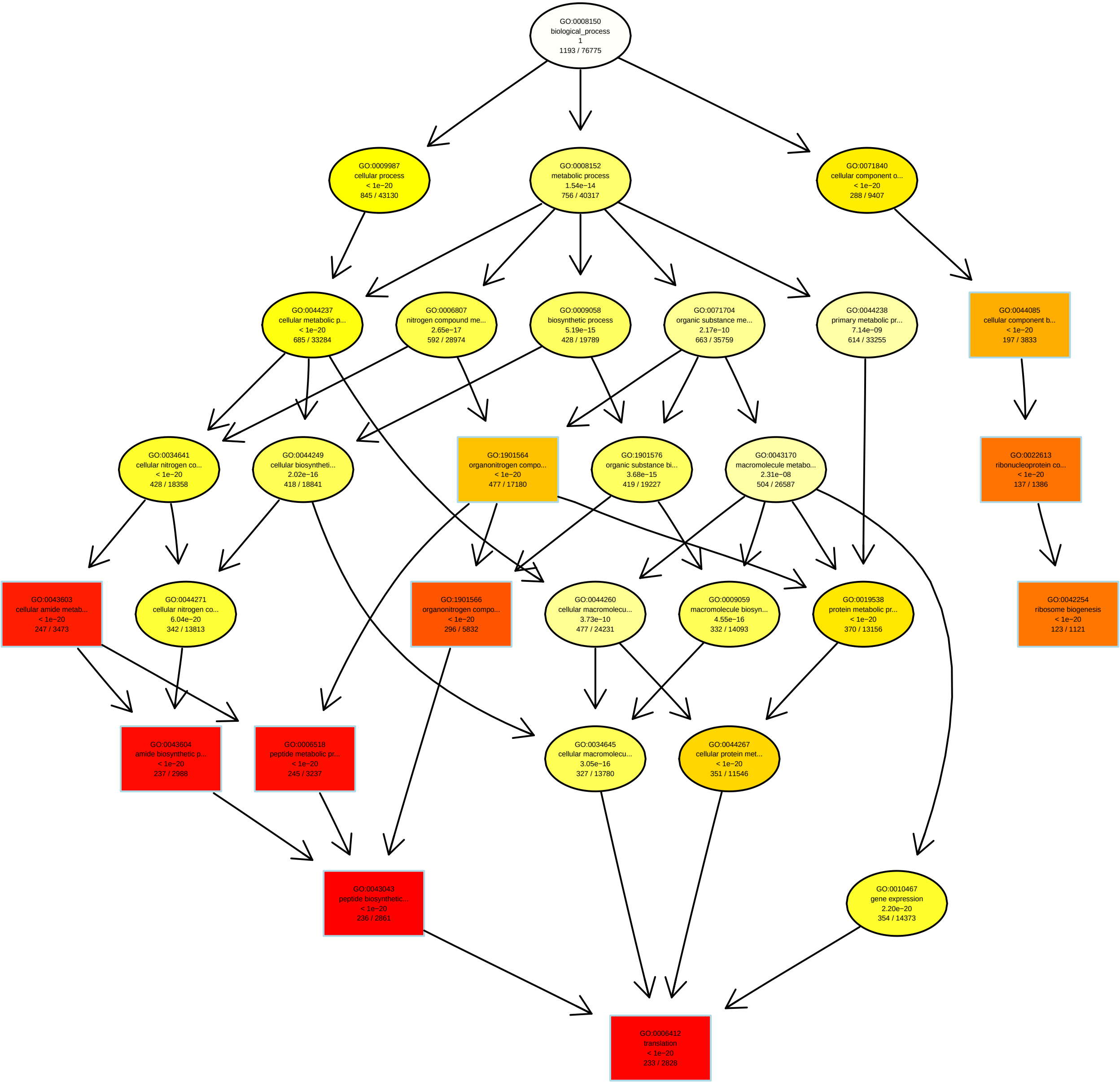
GO:0008150 biological_process 1 1193 / 76775
GO:0009987 cellular process < 1e−20 845 / 43130
GO:0008152 metabolic process 1.54e−14 756 / 40317
GO:0071840 cellular component o... < 1e−20 288 / 9407
GO:0044237 cellular metabolic p... < 1e−20 685 / 33284
GO:0006807 nitrogen compound me... 2.65e−17 592 / 28974
GO:0009058 biosynthetic process 5.19e−15 428 / 19789
GO:0071704 organic substance me... 2.17e−10 663 / 35759
GO:0044238 primary metabolic pr... 7.14e−09 614 / 33255
GO:0044085 cellular component b... < 1e−20 197 / 3833
GO:0034641 cellular nitrogen co... < 1e−20 428 / 18358
GO:0044249 cellular biosyntheti... 2.02e−16 418 / 18841
GO:1901564 organonitrogen compo... < 1e−20 477 / 17180
GO:1901576 organic substance bi... 3.68e−15 419 / 19227
GO:0043170 macromolecule metabo... 2.31e−08 504 / 26587
GO:0022613 ribonucleoprotein co... < 1e−20 137 / 1386
GO:0043603 cellular amide metab... < 1e−20 247 / 3473
GO:0044271 cellular nitrogen co... 6.04e−20 342 / 13813
GO:1901566 organonitrogen compo... < 1e−20 296 / 5832
GO:0044260 cellular macromolecu... 3.73e−10 477 / 24231
GO:0009059 macromolecule biosyn... 4.55e−16 332 / 14093
GO:0019538 protein metabolic pr... < 1e−20 370 / 13156
GO:0042254 ribosome biogenesis < 1e−20 123 / 1121
GO:0043604 amide biosynthetic p... < 1e−20 237 / 2988
GO:0006518 peptide metabolic pr... < 1e−20 245 / 3237
GO:0034645 cellular macromolecu... 3.05e−16 327 / 13780
GO:0044267 cellular protein met... < 1e−20 351 / 11546
GO:0043043 peptide biosynthetic... < 1e−20 236 / 2861
GO:0010467 gene expression 2.20e−20 354 / 14373
GO:0006412 translation < 1e−20 233 / 2828

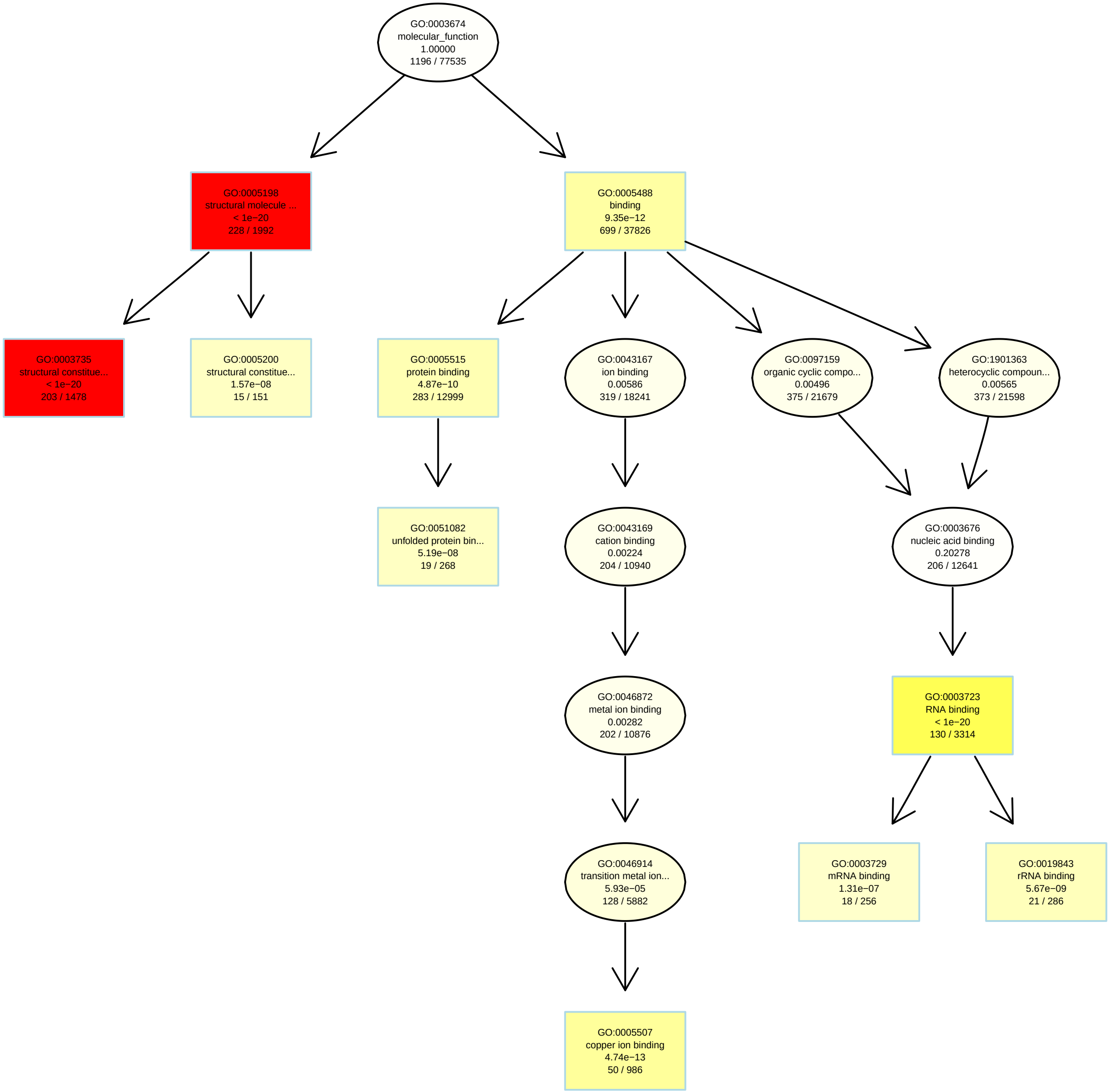
GO:0003674
molecular_function
1.00000
1196 / 77535
GO:0005198
structural molecule ...
< 1e−20
228 / 1992
GO:0005488
binding
9.35e−12
699 / 37826
GO:0003735
structural constitue...
< 1e−20
203 / 1478
GO:0005200
structural constitue...
1.57e−08
15 / 151
GO:0005515
protein binding
4.87e−10
283 / 12999
GO:0043167
ion binding
0.00586
319 / 18241
GO:0097159
organic cyclic compo...
0.00496
375 / 21679
GO:1901363
heterocyclic compoun...
0.00565
373 / 21598
GO:0051082
unfolded protein bin...
5.19e−08
19 / 268
GO:0043169
cation binding
0.00224
204 / 10940
GO:0003676
nucleic acid binding
0.20278
206 / 12641
GO:0046872
metal ion binding
0.00282
202 / 10876
GO:0003723
RNA binding
< 1e−20
130 / 3314
GO:0046914
transition metal ion...
5.93e−05
128 / 5882
GO:0003729
mRNA binding
1.31e−07
18 / 256
GO:0019843
rRNA binding
5.67e−09
21 / 286
GO:0005507
copper ion binding
4.74e−13
50 / 986

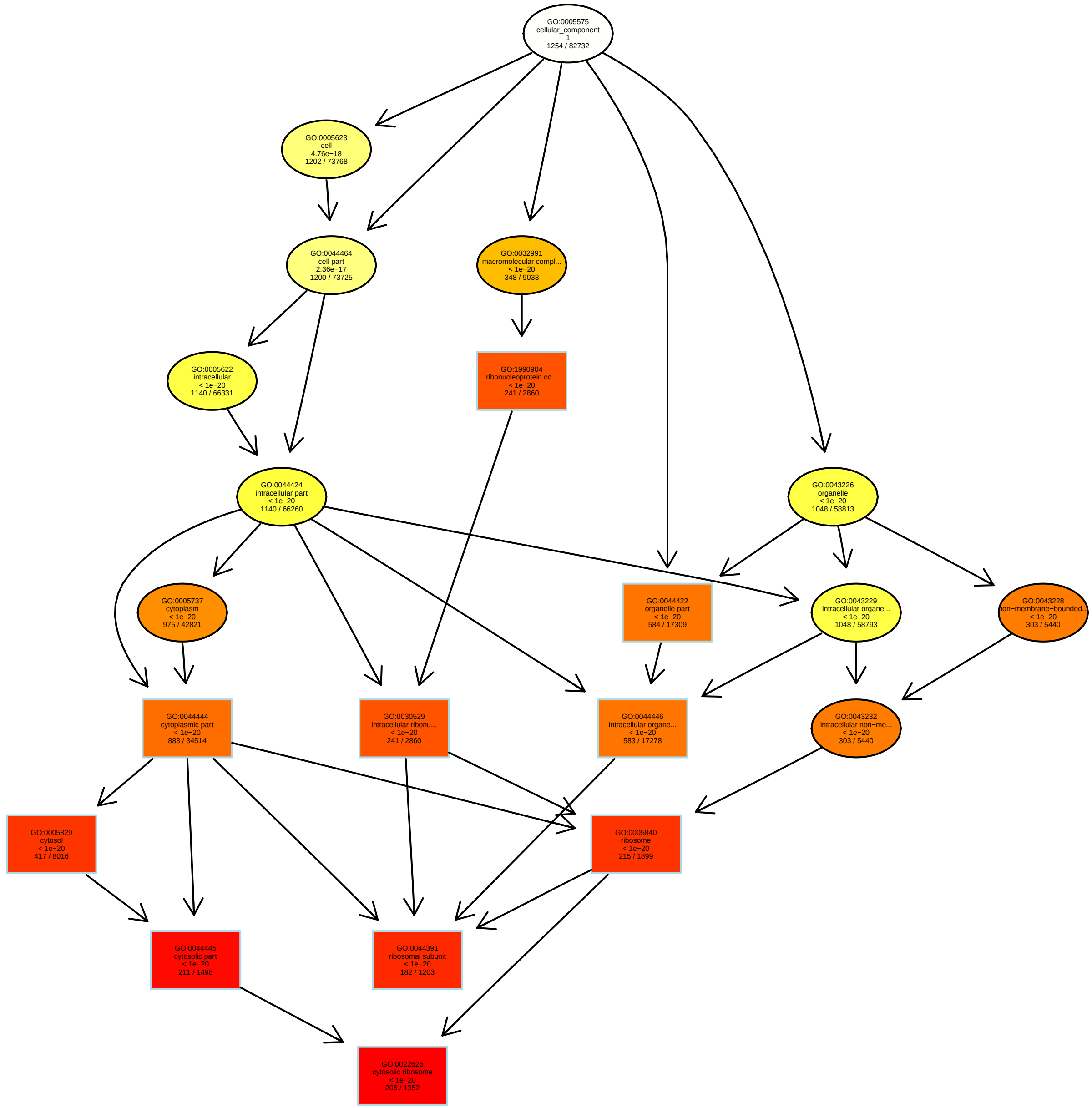
GO:0005575
cellular_component
1
1254 / 82732
GO:0005623
cell
4.76e−18
1202 / 73768
GO:0044464
cell part
2.36e−17
1200 / 73725
GO:0032991
macromolecular compl...
< 1e−20
348 / 9033
GO:0005622
intracellular
< 1e−20
1140 / 66331
GO:1990904
ribonucleoprotein co...
< 1e−20
241 / 2860
GO:0044424
intracellular part
< 1e−20
1140 / 66260
GO:0043226
organelle
< 1e−20
1048 / 58813
GO:0005737
cytoplasm
< 1e−20
975 / 42821
GO:0044422
organelle part
< 1e−20
584 / 17309
GO:0043229
intracellular organe...
< 1e−20
1048 / 58793
GO:0043228
non−membrane−bounded...
< 1e−20
303 / 5440
GO:0044444
cytoplasmic part
< 1e−20
883 / 34514
GO:0030529
intracellular ribonu...
< 1e−20
241 / 2860
GO:0044446
intracellular organe...
< 1e−20
583 / 17278
GO:0043232
intracellular non−me...
< 1e−20
303 / 5440
GO:0005829
cytosol
< 1e−20
417 / 8016
GO:0005840
ribosome
< 1e−20
215 / 1899
GO:0044445
cytosolic part
< 1e−20
211 / 1498
GO:0044391
ribosomal subunit
< 1e−20
182 / 1203
GO:0022626
cytosolic ribosome
< 1e−20
206 / 1352
