# Supplementary material for: Differential Alternative Splicing Genes and Isoform Regulation Networks of Rapeseed (Brassica napus L.) Infected with Sclerotinia sclerotiorum
Source: Genes (Basel). 2020 Jul 13;11(7):784. doi: 10.3390/genes11070784 (PMC7397149; doi:10.3390/genes11070784)
Supplement: Supplementary file 1 [file genes-11-00784-s001.zip › Fig. S2 Enriched GO terms of genes in the 85 WGCNA modules.zip!/Fig. S2/CytoNodegreenyellow-go.pdf]

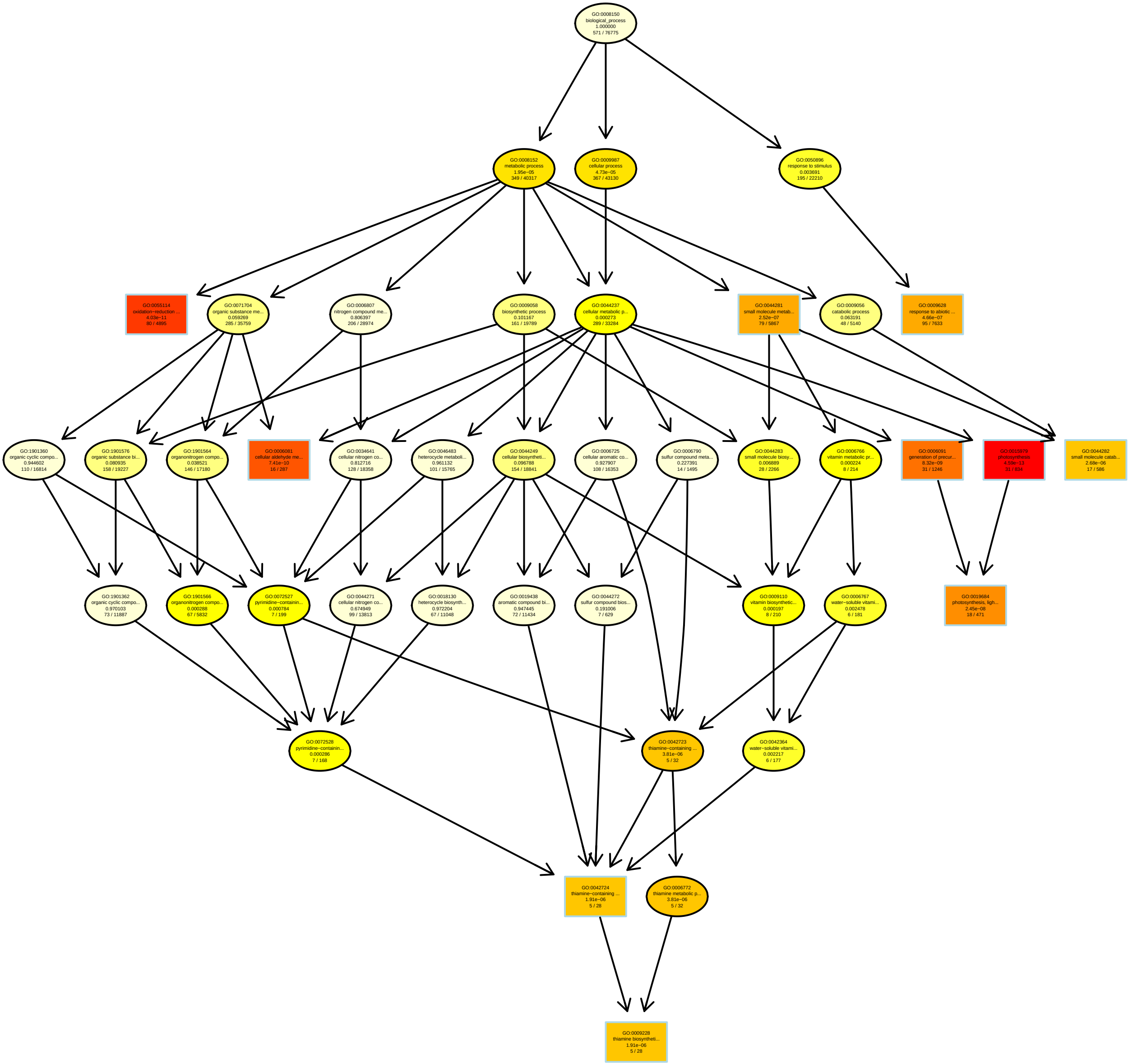
GO:0008150
biological_process
1.000000
571 / 76775
GO:0008152
metabolic process
1.95e−05
349 / 40317
GO:0009987
cellular process
4.73e−05
367 / 43130
GO:0050896
response to stimulus
0.003691
195 / 22210
GO:0055114
oxidation−reduction ...
4.03e−11
80 / 4895
GO:0071704
organic substance me...
0.059269
285 / 35759
GO:0006807
nitrogen compound me...
0.806397
206 / 28974
GO:0009058
biosynthetic process
0.101167
161 / 19789
GO:0044237
cellular metabolic p...
0.000273
289 / 33284
GO:0044281
small molecule metab...
2.52e−07
79 / 5867
GO:0009056
catabolic process
0.063191
48 / 5140
GO:0009628
response to abiotic ...
4.66e−07
95 / 7633
GO:1901360
organic cyclic compo...
0.944602
110 / 16814
GO:1901576
organic substance bi...
0.080935
158 / 19227
GO:1901564
organonitrogen compo...
0.038521
146 / 17180
GO:0006081
cellular aldehyde me...
7.41e−10
16 / 287
GO:0034641
cellular nitrogen co...
0.812716
128 / 18358
GO:0046483
heterocycle metaboli...
0.961132
101 / 15765
GO:0044249
cellular biosyntheti...
0.096788
154 / 18841
GO:0006725
cellular aromatic co...
0.927907
108 / 16353
GO:0006790
sulfur compound meta...
0.227391
14 / 1495
GO:0044283
small molecule biosy...
0.006889
28 / 2266
GO:0006766
vitamin metabolic pr...
0.000224
8 / 214
GO:0006091
generation of precur...
8.32e−09
31 / 1246
GO:0015979
photosynthesis
4.50e−13
31 / 834
GO:0044282
small molecule catab...
2.68e−06
17 / 586
GO:1901362
organic cyclic compo...
0.970103
73 / 11887
GO:1901566
organonitrogen compo...
0.000288
67 / 5832
GO:0072527
pyrimidine−containin...
0.000784
7 / 199
GO:0044271
cellular nitrogen co...
0.674949
99 / 13813
GO:0018130
heterocycle biosynth...
0.972204
67 / 11048
GO:0019438
aromatic compound bi...
0.947445
72 / 11434
GO:0044272
sulfur compound bios...
0.191006
7 / 629
GO:0009110
vitamin biosynthetic...
0.000197
8 / 210
GO:0006767
water−soluble vitami...
0.002478
6 / 181
GO:0019684
photosynthesis, ligh...
2.45e−08
18 / 471
GO:0072528
pyrimidine−containin...
0.000286
7 / 168
GO:0042723
thiamine−containing ...
3.81e−06
5 / 32
GO:0042364
water−soluble vitami...
0.002217
6 / 177
GO:0042724
thiamine−containing ...
1.91e−06
5 / 28
GO:0006772
thiamine metabolic p...
3.81e−06
5 / 32
GO:0009228
thiamine biosyntheti...
1.91e−06
5 / 28

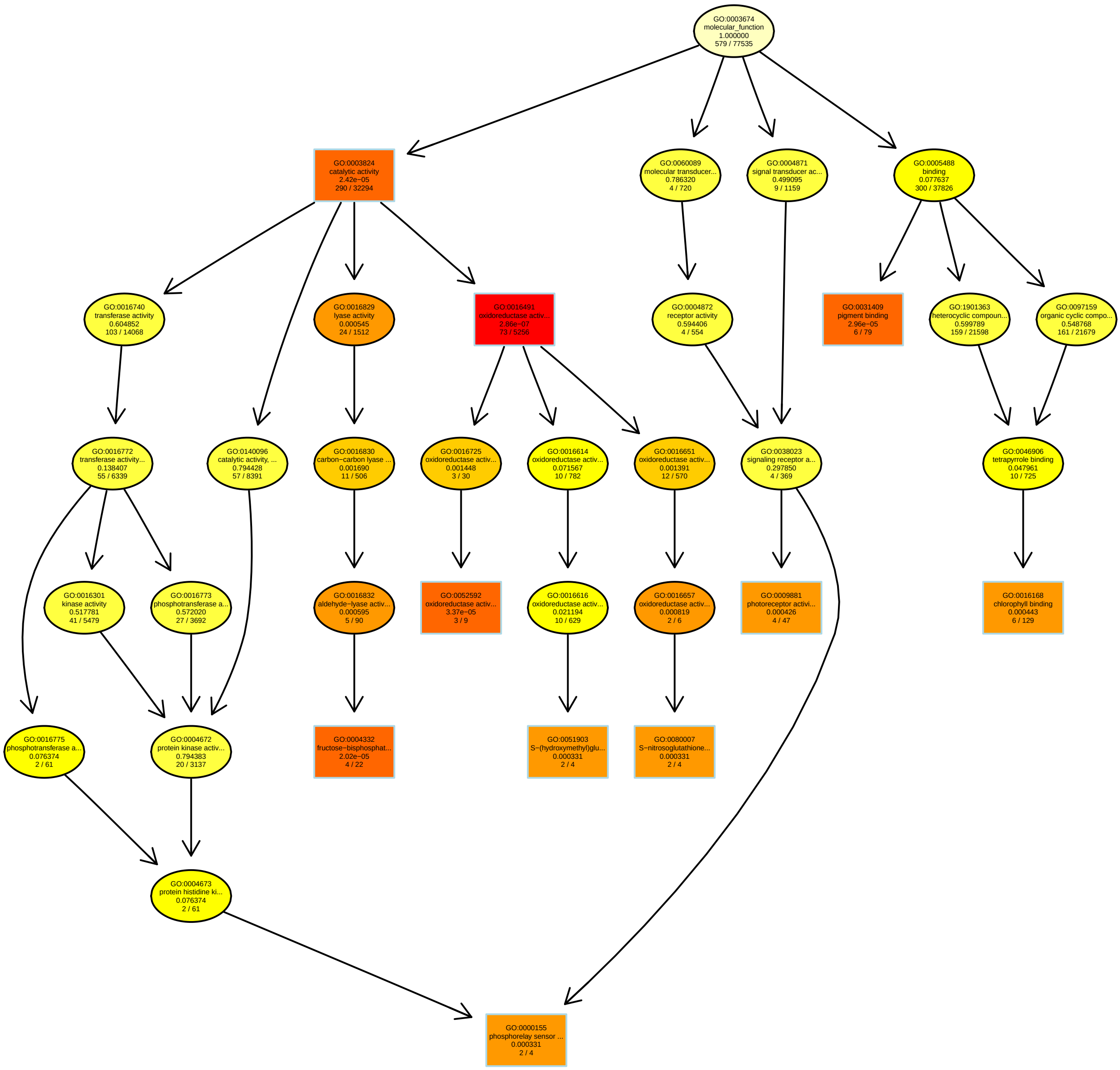
GO:0003674 molecular_function 1.000000 579 / 77535
GO:0003824 catalytic activity 2.42e−05 290 / 32294
GO:0060089 molecular transducer... 0.786320 4 / 720
GO:0004871 signal transducer ac... 0.499095 9 / 1159
GO:0005488 binding 0.077637 300 / 37826
GO:0016740 transferase activity 0.604852 103 / 14068
GO:0016829 lyase activity 0.000545 24 / 1512
GO:0016491 oxidoreductase activ... 2.86e−07 73 / 5256
GO:0004872 receptor activity 0.594406 4 / 554
GO:0031409 pigment binding 2.96e−05 6 / 79
GO:1901363 heterocyclic compoun... 0.599789 159 / 21598
GO:0097159 organic cyclic compo... 0.548768 161 / 21679
GO:0016772 transferase activity... 0.138407 55 / 6339
GO:0140096 catalytic activity, ... 0.794428 57 / 8391
GO:0016830 carbon−carbon lyase ... 0.001690 11 / 506
GO:0016725 oxidoreductase activ... 0.001448 3 / 30
GO:0016614 oxidoreductase activ... 0.071567 10 / 782
GO:0016651 oxidoreductase activ... 0.001391 12 / 570
GO:0038023 signaling receptor a... 0.297850 4 / 369
GO:0046906 tetrapyrrole binding 0.047961 10 / 725
GO:0016301 kinase activity 0.517781 41 / 5479
GO:0016773 phosphotransferase a... 0.572020 27 / 3692
GO:0016832 aldehyde−lyase activ... 0.000595 5 / 90
GO:0052592 oxidoreductase activ... 3.37e−05 3 / 9
GO:0016616 oxidoreductase activ... 0.021194 10 / 629
GO:0016657 oxidoreductase activ... 0.000819 2 / 6
GO:0009881 photoreceptor activi... 0.000426 4 / 47
GO:0016168 chlorophyll binding 0.000443 6 / 129
GO:0016775 phosphotransferase a... 0.076374 2 / 61
GO:0004672 protein kinase activ... 0.794383 20 / 3137
GO:0004332 fructose−bisphosphat... 2.02e−05 4 / 22
GO:0051903 S−(hydroxymethyl)glu... 0.000331 2 / 4
GO:0080007 S−nitrosoglutathione... 0.000331 2 / 4
GO:0004673 protein histidine ki... 0.076374 2 / 61
GO:0000155 phosphorelay sensor ... 0.000331 2 / 4

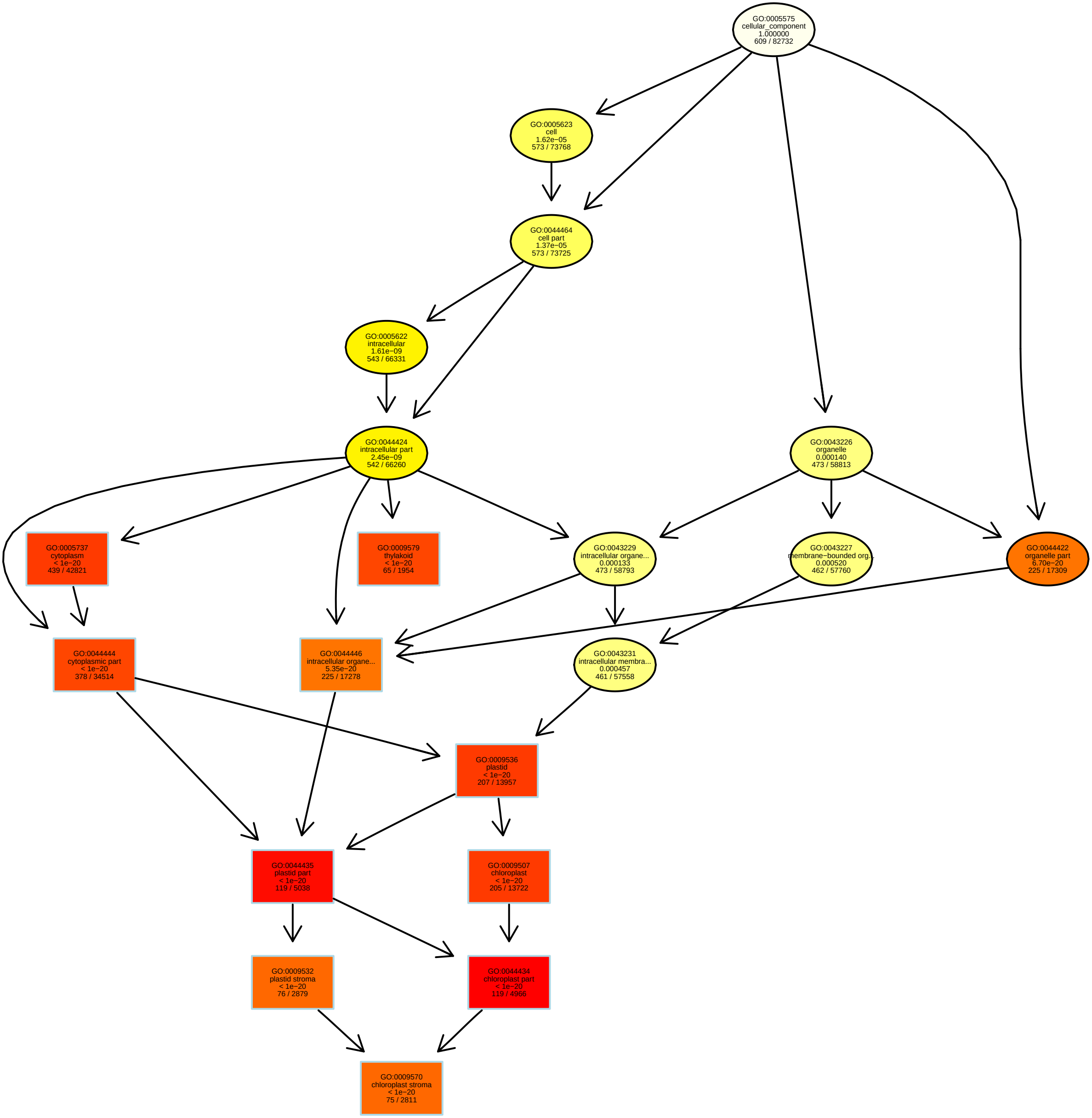
GO:0005575
cellular_component
1.000000
609 / 82732
GO:0005623
cell
1.62e−05
573 / 73768
GO:0044464
cell part
1.37e−05
573 / 73725
GO:0005622
intracellular
1.61e−09
543 / 66331
GO:0044424
intracellular part
2.45e−09
542 / 66260
GO:0043226
organelle
0.000140
473 / 58813
GO:0005737
cytoplasm
< 1e−20
439 / 42821
GO:0009579
thylakoid
< 1e−20
65 / 1954
GO:0043229
intracellular organe...
0.000133
473 / 58793
GO:0043227
membrane−bounded org...
0.000520
462 / 57760
GO:0044422
organelle part
6.70e−20
225 / 17309
GO:0044444
cytoplasmic part
< 1e−20
378 / 34514
GO:0044446
intracellular organe...
5.35e−20
225 / 17278
GO:0043231
intracellular membra...
0.000457
461 / 57558
GO:0009536
plastid
< 1e−20
207 / 13957
GO:0044435
plastid part
< 1e−20
119 / 5038
GO:0009507
chloroplast
< 1e−20
205 / 13722
GO:0009532
plastid stroma
< 1e−20
76 / 2879
GO:0044434
chloroplast part
< 1e−20
119 / 4966
GO:0009570
chloroplast stroma
< 1e−20
75 / 2811
