# Supplementary material for: Differential Alternative Splicing Genes and Isoform Regulation Networks of Rapeseed (Brassica napus L.) Infected with Sclerotinia sclerotiorum
Source: Genes (Basel). 2020 Jul 13;11(7):784. doi: 10.3390/genes11070784 (PMC7397149; doi:10.3390/genes11070784)
Supplement: Supplementary file 1 [file genes-11-00784-s001.zip › Fig. S2 Enriched GO terms of genes in the 85 WGCNA modules.zip!/Fig. S2/CytoNodehoneydew1-go.pdf]

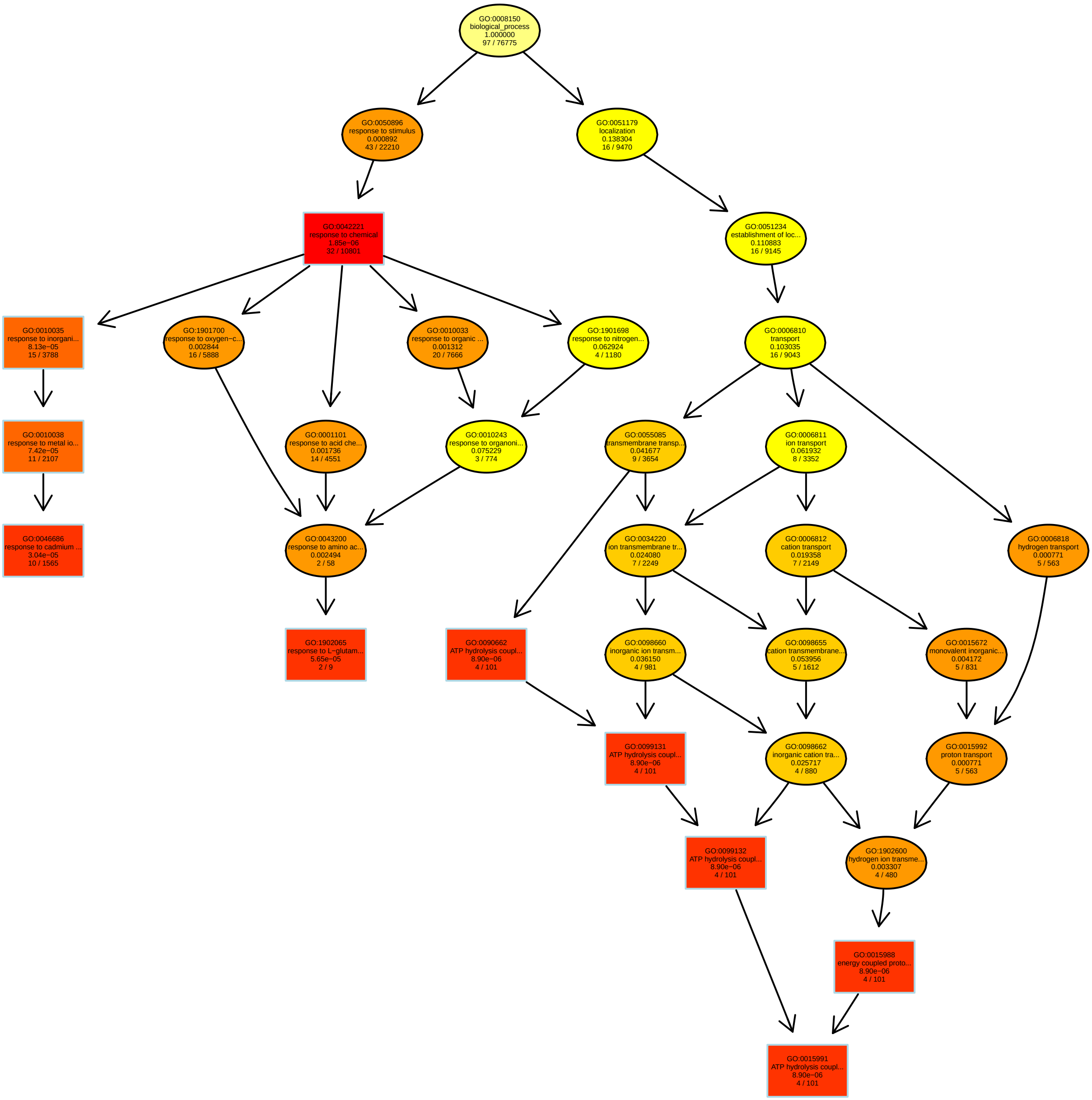
GO:0008150
biological_process
1.000000
97 / 76775
GO:0050896
response to stimulus
0.000892
43 / 22210
GO:0051179
localization
0.138304
16 / 9470
GO:0042221
response to chemical
1.85e−06
32 / 10801
GO:0051234
establishment of loc...
0.110883
16 / 9145
GO:0010035
response to inorgani...
8.13e−05
15 / 3788
GO:1901700
response to oxygen−c...
0.002844
16 / 5888
GO:0010033
response to organic ...
0.001312
20 / 7666
GO:1901698
response to nitrogen...
0.062924
4 / 1180
GO:0006810
transport
0.103035
16 / 9043
GO:0010038
response to metal io...
7.42e−05
11 / 2107
GO:0001101
response to acid che...
0.001736
14 / 4551
GO:0010243
response to organoni...
0.075229
3 / 774
GO:0055085
transmembrane transp...
0.041677
9 / 3654
GO:0006811
ion transport
0.061932
8 / 3352
GO:0046686
response to cadmium ...
3.04e−05
10 / 1565
GO:0043200
response to amino ac...
0.002494
2 / 58
GO:0034220
ion transmembrane tr...
0.024080
7 / 2249
GO:0006812
cation transport
0.019358
7 / 2149
GO:0006818
hydrogen transport
0.000771
5 / 563
GO:1902065
response to L−glutam...
5.65e−05
2 / 9
GO:0090662
ATP hydrolysis coupl...
8.90e−06
4 / 101
GO:0098660
inorganic ion transm...
0.036150
4 / 981
GO:0098655
cation transmembrane...
0.053956
5 / 1612
GO:0015672
monovalent inorganic...
0.004172
5 / 831
GO:0099131
ATP hydrolysis coupl...
8.90e−06
4 / 101
GO:0098662
inorganic cation tra...
0.025717
4 / 880
GO:0015992
proton transport
0.000771
5 / 563
GO:0099132
ATP hydrolysis coupl...
8.90e−06
4 / 101
GO:1902600
hydrogen ion transme...
0.003307
4 / 480
GO:0015988
energy coupled proto...
8.90e−06
4 / 101
GO:0015991
ATP hydrolysis coupl...
8.90e−06
4 / 101

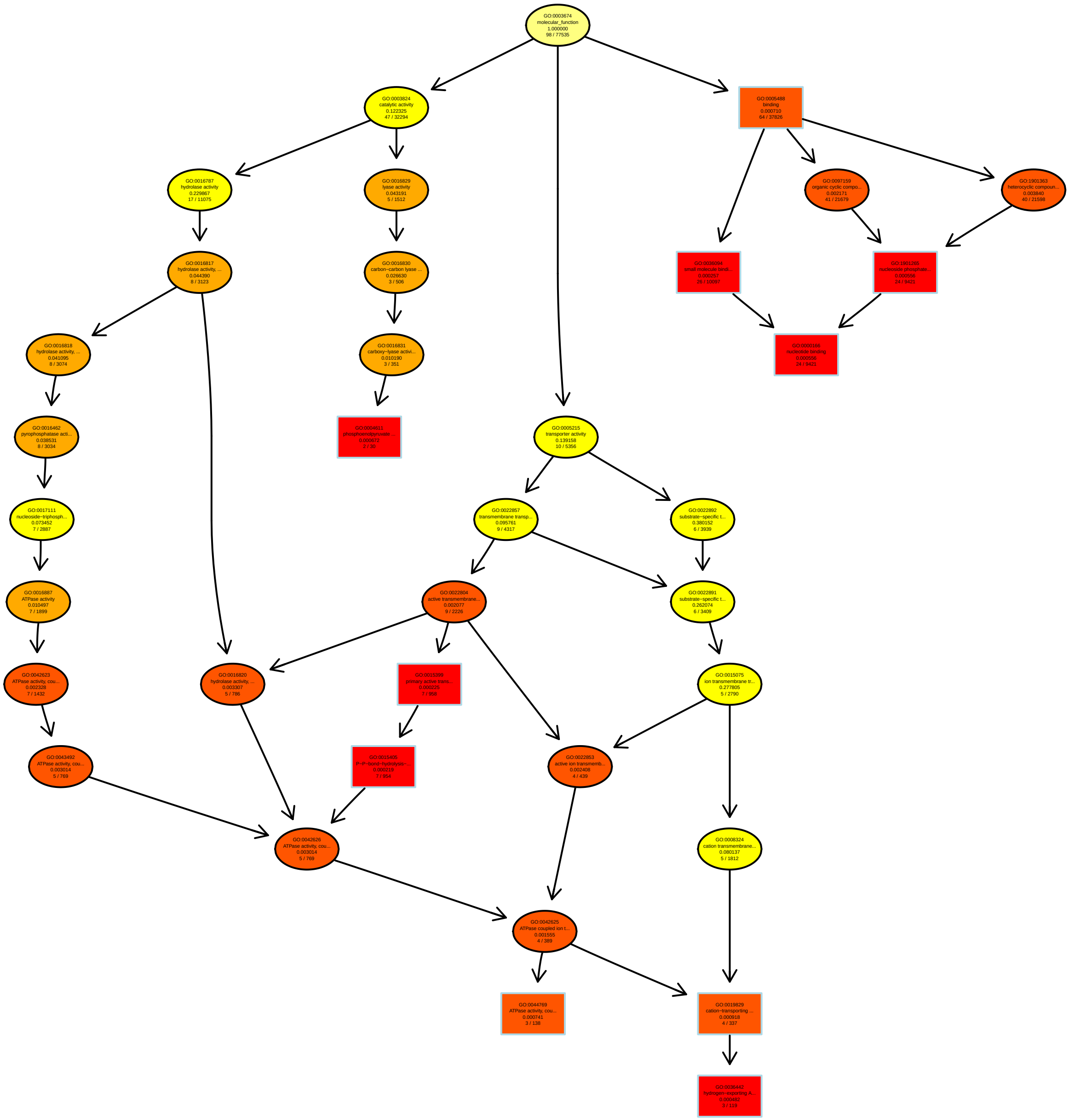
GO:0003674 molecular_function 1.000000 98 / 77535
GO:0003824 catalytic activity 0.122325 47 / 32294
GO:0005488 binding 0.000710 64 / 37826
GO:0016787 hydrolase activity 0.229867 17 / 11075
GO:0016829 lyase activity 0.043191 5 / 1512
GO:0097159 organic cyclic compo... 0.002171 41 / 21679
GO:1901363 heterocyclic compoun... 0.003840 40 / 21598
GO:0016817 hydrolase activity, ... 0.044390 8 / 3123
GO:0016830 carbon−carbon lyase ... 0.026630 3 / 506
GO:0036094 small molecule bindi... 0.000257 26 / 10097
GO:1901265 nucleoside phosphate... 0.000556 24 / 9421
GO:0016818 hydrolase activity, ... 0.041095 8 / 3074
GO:0016831 carboxy−lyase activi... 0.010190 3 / 351
GO:0000166 nucleotide binding 0.000556 24 / 9421
GO:0016462 pyrophosphatase acti... 0.038531 8 / 3034
GO:0004611 phosphoenolpyruvate ... 0.000672 2 / 30
GO:0005215 transporter activity 0.139158 10 / 5356
GO:0017111 nucleoside−triphosph... 0.073452 7 / 2887
GO:0022857 transmembrane transp... 0.095761 9 / 4317
GO:0022892 substrate−specific t... 0.380152 6 / 3939
GO:0016887 ATPase activity 0.010497 7 / 1899
GO:0022804 active transmembrane... 0.002077 9 / 2226
GO:0022891 substrate−specific t... 0.262074 6 / 3409
GO:0042623 ATPase activity, cou... 0.002328 7 / 1432
GO:0016820 hydrolase activity, ... 0.003307 5 / 786
GO:0015399 primary active trans... 0.000225 7 / 958
GO:0015075 ion transmembrane tr... 0.277805 5 / 2790
GO:0043492 ATPase activity, cou... 0.003014 5 / 769
GO:0015405 P−P−bond−hydrolysis−... 0.000219 7 / 954
GO:0022853 active ion transmemb... 0.002408 4 / 439
GO:0042626 ATPase activity, cou... 0.003014 5 / 769
GO:0008324 cation transmembrane... 0.080137 5 / 1812
GO:0042625 ATPase coupled ion t... 0.001555 4 / 389
GO:0044769 ATPase activity, cou... 0.000741 3 / 138
GO:0019829 cation−transporting ... 0.000918 4 / 337
GO:0036442 hydrogen−exporting A... 0.000482 3 / 119

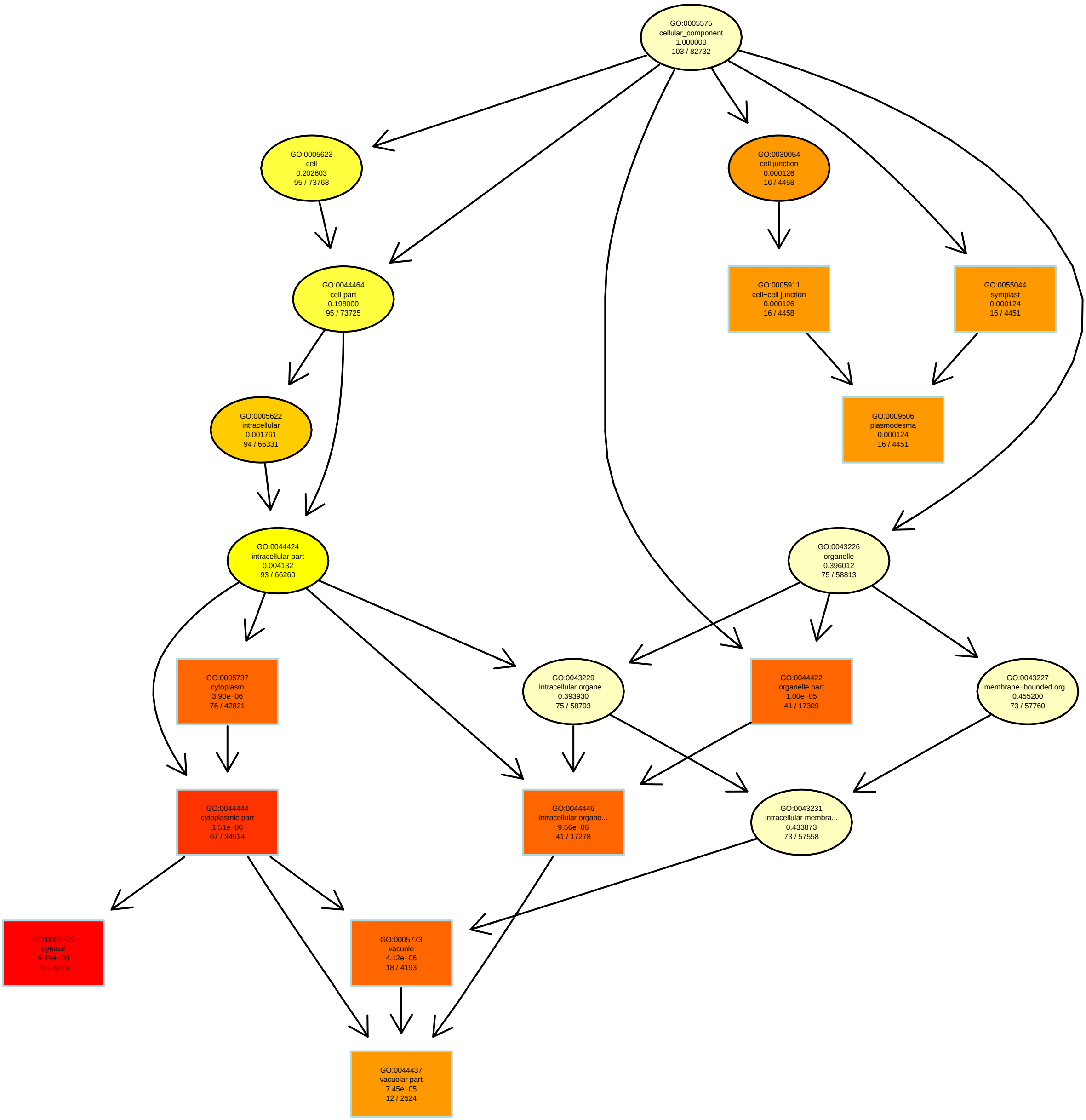
GO:0005575
cellular_component
1.000000
103 / 82732
GO:0005623
cell
0.202603
95 / 73768
GO:0030054
cell junction
0.000126
16 / 4458
GO:0044464
cell part
0.198000
95 / 73725
GO:0005911
cell−cell junction
0.000126
16 / 4458
GO:0055044
symplast
0.000124
16 / 4451
GO:0005622
intracellular
0.001761
94 / 66331
GO:0009506
plasmodesma
0.000124
16 / 4451
GO:0044424
intracellular part
0.004132
93 / 66260
GO:0043226
organelle
0.396012
75 / 58813
GO:0005737
cytoplasm
3.90e−06
76 / 42821
GO:0043229
intracellular organe...
0.393930
75 / 58793
GO:0044422
organelle part
1.00e−05
41 / 17309
GO:0043227
membrane−bounded org...
0.455200
73 / 57760
GO:0044444
cytoplasmic part
1.51e−06
67 / 34514
GO:0044446
intracellular organe...
9.56e−06
41 / 17278
GO:0043231
intracellular membra...
0.433873
73 / 57558
GO:0005829
cytosol
9.49e−08
29 / 8016
GO:0005773
vacuole
4.12e−06
18 / 4193
GO:0044437
vacuolar part
7.45e−05
12 / 2524
