# Supplementary material for: Differential Alternative Splicing Genes and Isoform Regulation Networks of Rapeseed (Brassica napus L.) Infected with Sclerotinia sclerotiorum
Source: Genes (Basel). 2020 Jul 13;11(7):784. doi: 10.3390/genes11070784 (PMC7397149; doi:10.3390/genes11070784)
Supplement: Supplementary file 1 [file genes-11-00784-s001.zip › Fig. S2 Enriched GO terms of genes in the 85 WGCNA modules.zip!/Fig. S2/CytoNodeindianred4-go.pdf]

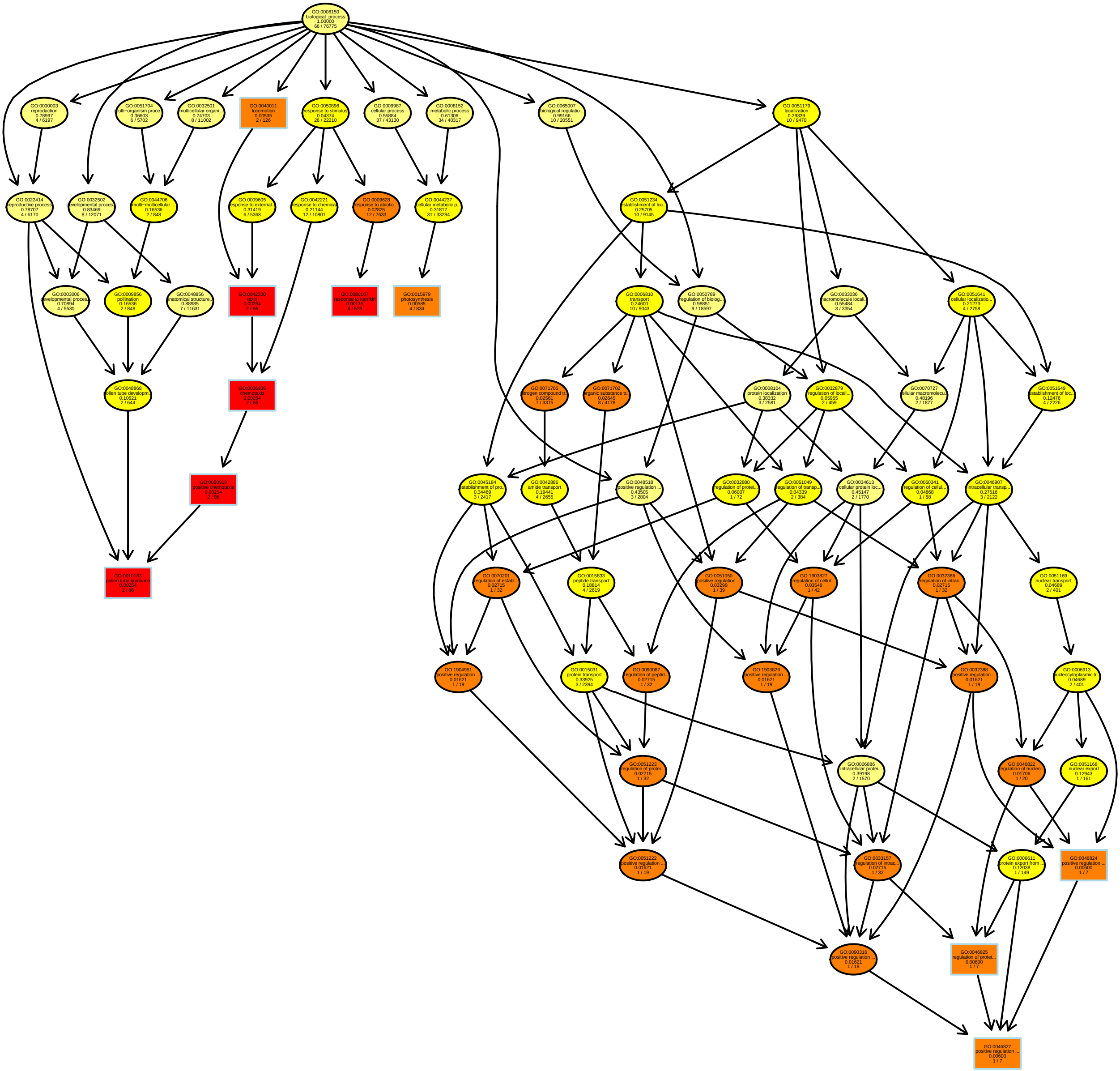

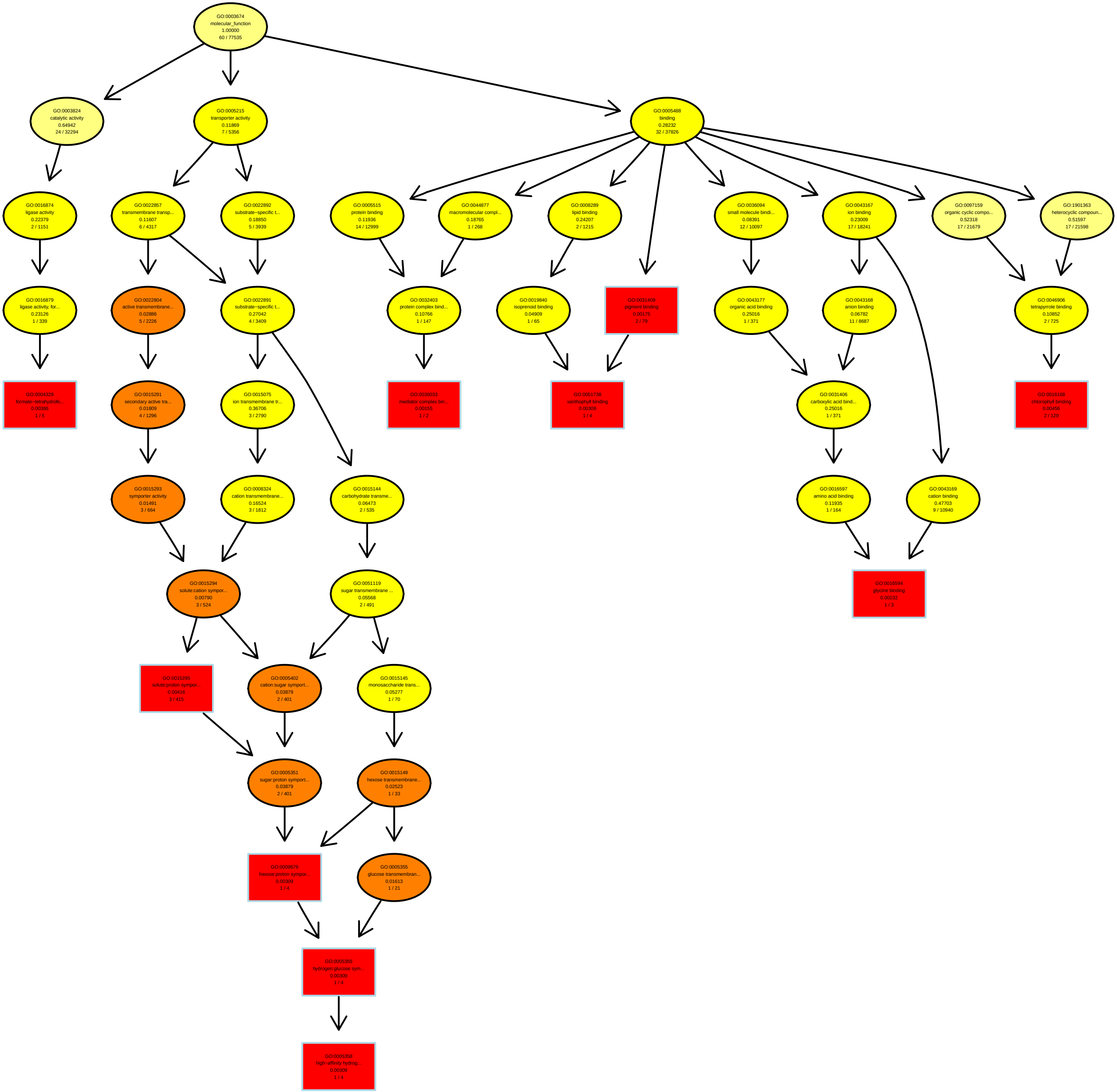

GO:0003674 molecular_function 1.00000 60 / 77535
GO:0003824 catalytic activity 0.64942 24 / 32294
GO:0005215 transporter activity 0.11869 7 / 5356
GO:0005488 binding 0.28232 32 / 37826
GO:0016874 ligase activity 0.22379 2 / 1151
GO:0022857 transmembrane transp... 0.11607 6 / 4317
GO:0022892 substrate−specific t... 0.18850 5 / 3939
GO:0005515 protein binding 0.11936 14 / 12999
GO:0044877 macromolecular compl... 0.18765 1 / 268
GO:0008289 lipid binding 0.24207 2 / 1215
GO:0036094 small molecule bindi... 0.08391 12 / 10097
GO:0043167 ion binding 0.23009 17 / 18241
GO:0097159 organic cyclic compo... 0.52318 17 / 21679
GO:1901363 heterocyclic compoun... 0.51597 17 / 21598
GO:0016879 ligase activity, for... 0.23126 1 / 339
GO:0022804 active transmembrane... 0.02886 5 / 2226
GO:0022891 substrate−specific t... 0.27042 4 / 3409
GO:0032403 protein complex bind... 0.10766 1 / 147
GO:0019840 isoprenoid binding 0.04909 1 / 65
GO:0031409 pigment binding 0.00175 2 / 79
GO:0043177 organic acid binding 0.25016 1 / 371
GO:0043168 anion binding 0.06782 11 / 8687
GO:0046906 tetrapyrrole binding 0.10852 2 / 725
GO:0004329 formate−tetrahydrofo... 0.00386 1 / 5
GO:0015291 secondary active tra... 0.01809 4 / 1296
GO:0015075 ion transmembrane tr... 0.36706 3 / 2790
GO:0036033 mediator complex bin... 0.00155 1 / 2
GO:0051738 xanthophyll binding 0.00309 1 / 4
GO:0031406 carboxylic acid bind... 0.25016 1 / 371
GO:0016168 chlorophyll binding 0.00456 2 / 129
GO:0015293 symporter activity 0.01491 3 / 664
GO:0008324 cation transmembrane... 0.16524 3 / 1812
GO:0015144 carbohydrate transme... 0.06473 2 / 535
GO:0016597 amino acid binding 0.11935 1 / 164
GO:0043169 cation binding 0.47703 9 / 10940
GO:0015294 solute:cation sympor... 0.00790 3 / 524
GO:0051119 sugar transmembrane ... 0.05568 2 / 491
GO:0016594 glycine binding 0.00232 1 / 3
GO:0015295 solute:proton sympor... 0.00416 3 / 415
GO:0005402 cation:sugar symport... 0.03879 2 / 401
GO:0015145 monosaccharide trans... 0.05277 1 / 70
GO:0005351 sugar:proton symport... 0.03879 2 / 401
GO:0015149 hexose transmembrane... 0.02523 1 / 33
GO:0009679 hexose:proton sympor... 0.00309 1 / 4
GO:0005355 glucose transmembran... 0.01613 1 / 21
GO:0005356 hydrogen:glucose sym... 0.00309 1 / 4
GO:0005358 high−affinity hydrog... 0.00309 1 / 4

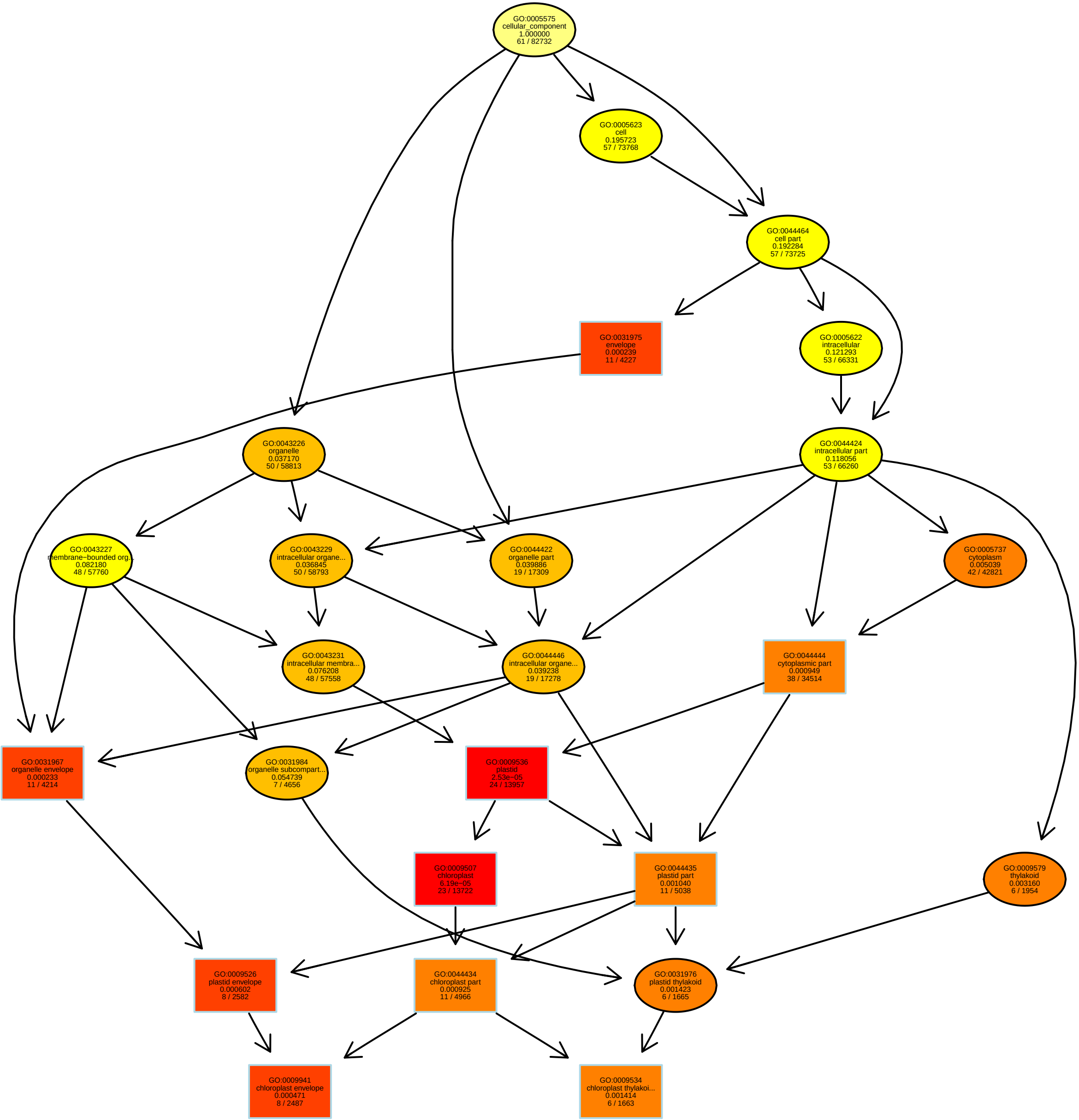
GO:0005575
cellular_component
1.000000
61 / 82732
GO:0005623
cell
0.195723
57 / 73768
GO:0044464
cell part
0.192284
57 / 73725
GO:0031975
envelope
0.000239
11 / 4227
GO:0005622
intracellular
0.121293
53 / 66331
GO:0043226
organelle
0.037170
50 / 58813
GO:0044424
intracellular part
0.118056
53 / 66260
GO:0043227
membrane−bounded org...
0.082180
48 / 57760
GO:0043229
intracellular organe...
0.036845
50 / 58793
GO:0044422
organelle part
0.039886
19 / 17309
GO:0005737
cytoplasm
0.005039
42 / 42821
GO:0043231
intracellular membra...
0.076208
48 / 57558
GO:0044446
intracellular organe...
0.039238
19 / 17278
GO:0044444
cytoplasmic part
0.000949
38 / 34514
GO:0031967
organelle envelope
0.000233
11 / 4214
GO:0031984
organelle subcompart...
0.054739
7 / 4656
GO:0009536
plastid
2.53e−05
24 / 13957
GO:0009507
chloroplast
6.19e−05
23 / 13722
GO:0044435
plastid part
0.001040
11 / 5038
GO:0009579
thylakoid
0.003160
6 / 1954
GO:0009526
plastid envelope
0.000602
8 / 2582
GO:0044434
chloroplast part
0.000925
11 / 4966
GO:0031976
plastid thylakoid
0.001423
6 / 1665
GO:0009941
chloroplast envelope
0.000471
8 / 2487
GO:0009534
chloroplast thylakoi...
0.001414
6 / 1663
