# Supplementary material for: Differential Alternative Splicing Genes and Isoform Regulation Networks of Rapeseed (Brassica napus L.) Infected with Sclerotinia sclerotiorum
Source: Genes (Basel). 2020 Jul 13;11(7):784. doi: 10.3390/genes11070784 (PMC7397149; doi:10.3390/genes11070784)
Supplement: Supplementary file 1 [file genes-11-00784-s001.zip › Fig. S2 Enriched GO terms of genes in the 85 WGCNA modules.zip!/Fig. S2/CytoNodeivory-go.pdf]

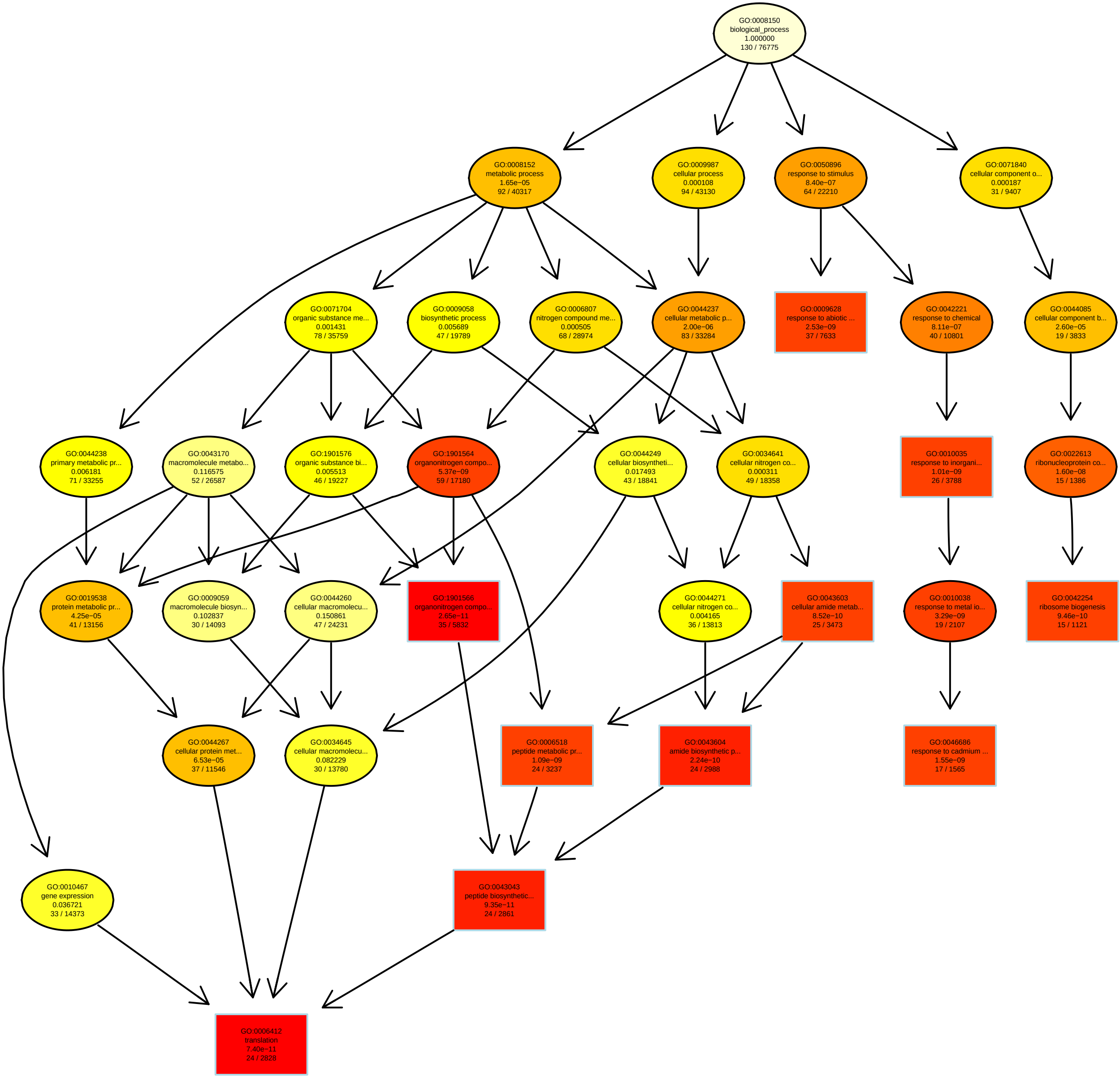
GO:0008150
biological_process
1.000000
130 / 76775
GO:0008152
metabolic process
1.65e−05
92 / 40317
GO:0009987
cellular process
0.000108
94 / 43130
GO:0050896
response to stimulus
8.40e−07
64 / 22210
GO:0071840
cellular component o...
0.000187
31 / 9407
GO:0071704
organic substance me...
0.001431
78 / 35759
GO:0009058
biosynthetic process
0.005689
47 / 19789
GO:0006807
nitrogen compound me...
0.000505
68 / 28974
GO:0044237
cellular metabolic p...
2.00e−06
83 / 33284
GO:0009628
response to abiotic ...
2.53e−09
37 / 7633
GO:0042221
response to chemical
8.11e−07
40 / 10801
GO:0044085
cellular component b...
2.60e−05
19 / 3833
GO:0044238
primary metabolic pr...
0.006181
71 / 33255
GO:0043170
macromolecule metabo...
0.116575
52 / 26587
GO:1901576
organic substance bi...
0.005513
46 / 19227
GO:1901564
organonitrogen compo...
5.37e−09
59 / 17180
GO:0044249
cellular biosyntheti...
0.017493
43 / 18841
GO:0034641
cellular nitrogen co...
0.000311
49 / 18358
GO:0010035
response to inorgani...
1.01e−09
26 / 3788
GO:0022613
ribonucleoprotein co...
1.60e−08
15 / 1386
GO:0019538
protein metabolic pr...
4.25e−05
41 / 13156
GO:0009059
macromolecule biosyn...
0.102837
30 / 14093
GO:0044260
cellular macromolecu...
0.150861
47 / 24231
GO:1901566
organonitrogen compo...
2.65e−11
35 / 5832
GO:0044271
cellular nitrogen co...
0.004165
36 / 13813
GO:0043603
cellular amide metab...
8.52e−10
25 / 3473
GO:0010038
response to metal io...
3.29e−09
19 / 2107
GO:0042254
ribosome biogenesis
9.46e−10
15 / 1121
GO:0044267
cellular protein met...
6.53e−05
37 / 11546
GO:0034645
cellular macromolecu...
0.082229
30 / 13780
GO:0006518
peptide metabolic pr...
1.09e−09
24 / 3237
GO:0043604
amide biosynthetic p...
2.24e−10
24 / 2988
GO:0046686
response to cadmium ...
1.55e−09
17 / 1565
GO:0010467
gene expression
0.036721
33 / 14373
GO:0043043
peptide biosynthetic...
9.35e−11
24 / 2861
GO:0006412
translation
7.40e−11
24 / 2828

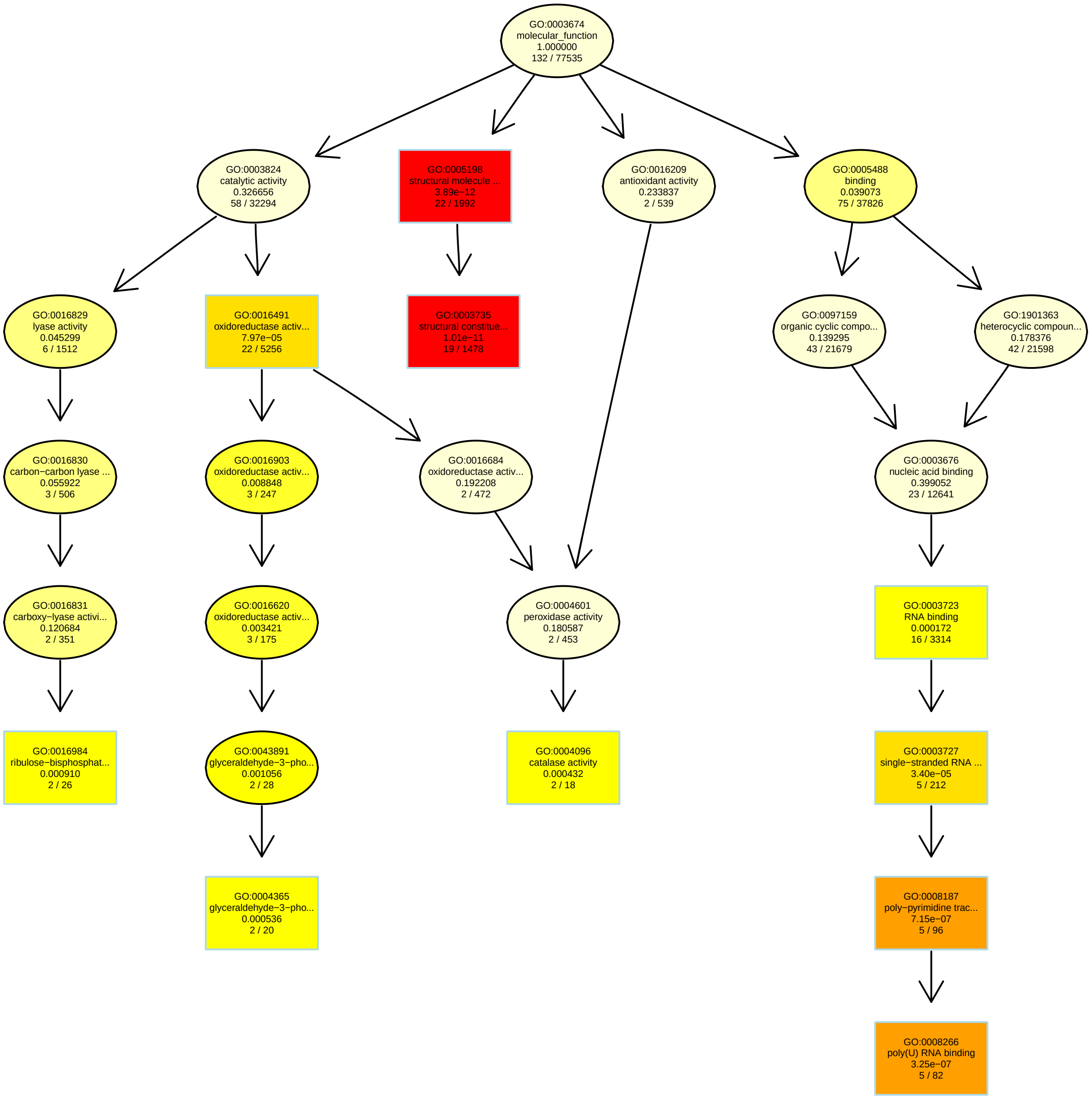
GO:0003674
molecular_function
1.000000
132 / 77535
GO:0003824
catalytic activity
0.326656
58 / 32294
GO:0005198
structural molecule ...
3.89e−12
22 / 1992
GO:0016209
antioxidant activity
0.233837
2 / 539
GO:0005488
binding
0.039073
75 / 37826
GO:0016829
lyase activity
0.045299
6 / 1512
GO:0016491
oxidoreductase activ...
7.97e−05
22 / 5256
GO:0003735
structural constitue...
1.01e−11
19 / 1478
GO:0097159
organic cyclic compo...
0.139295
43 / 21679
GO:1901363
heterocyclic compoun...
0.178376
42 / 21598
GO:0016830
carbon−carbon lyase ...
0.055922
3 / 506
GO:0016903
oxidoreductase activ...
0.008848
3 / 247
GO:0016684
oxidoreductase activ...
0.192208
2 / 472
GO:0003676
nucleic acid binding
0.399052
23 / 12641
GO:0016831
carboxy−lyase activi...
0.120684
2 / 351
GO:0016620
oxidoreductase activ...
0.003421
3 / 175
GO:0004601
peroxidase activity
0.180587
2 / 453
GO:0003723
RNA binding
0.000172
16 / 3314
GO:0016984
ribulose−bisphosphat...
0.000910
2 / 26
GO:0043891
glyceraldehyde−3−pho...
0.001056
2 / 28
GO:0004096
catalase activity
0.000432
2 / 18
GO:0003727
single−stranded RNA ...
3.40e−05
5 / 212
GO:0004365
glyceraldehyde−3−pho...
0.000536
2 / 20
GO:0008187
poly−pyrimidine trac...
7.15e−07
5 / 96
GO:0008266
poly(U) RNA binding
3.25e−07
5 / 82

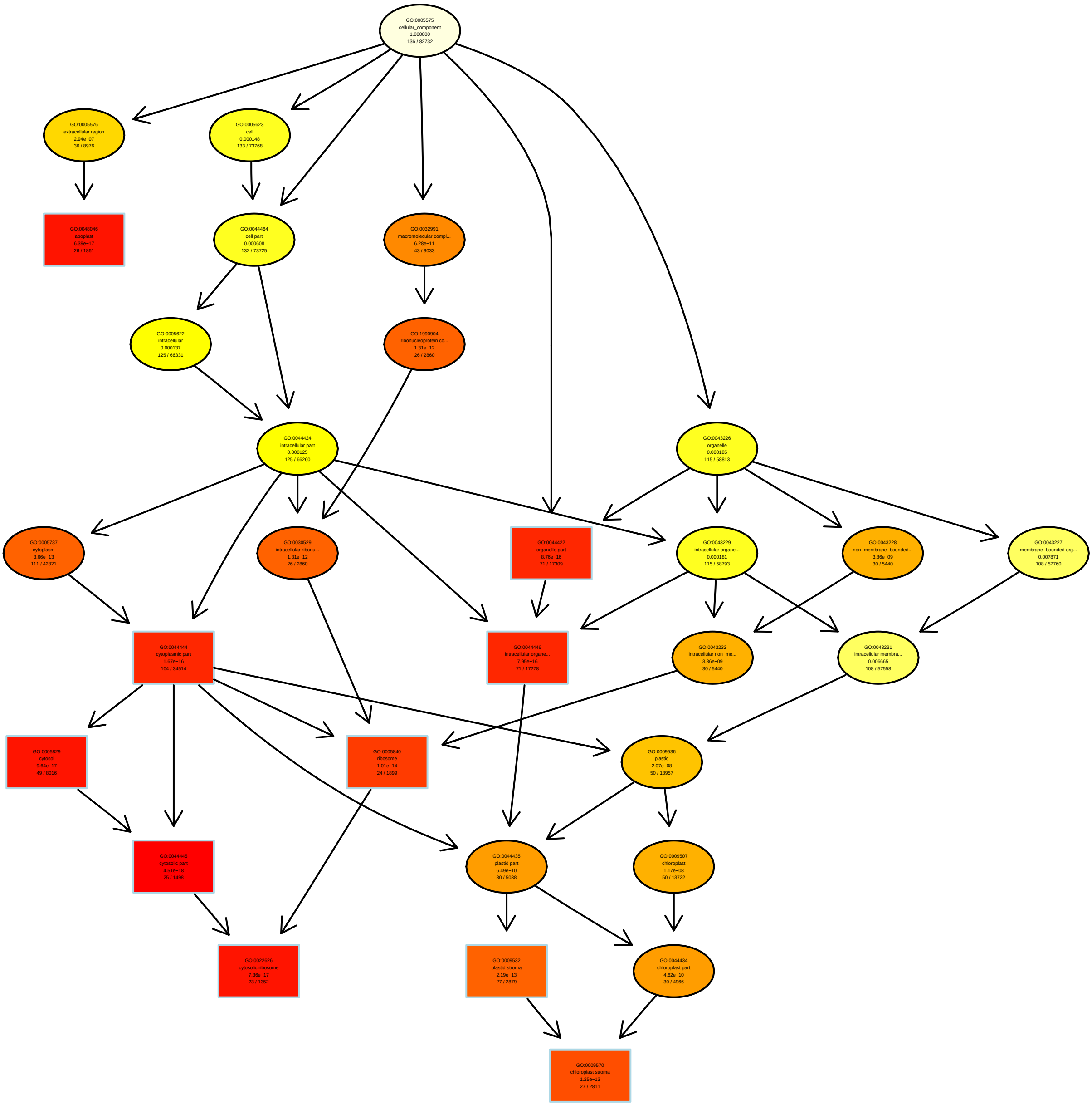
GO:0005575 cellular_component 1.000000 136 / 82732
GO:0005576 extracellular region 2.94e−07 36 / 8976
GO:0005623 cell 0.000148 133 / 73768
GO:0048046 apoplast 6.39e−17 26 / 1861
GO:0044464 cell part 0.000608 132 / 73725
GO:0032991 macromolecular compl... 6.28e−11 43 / 9033
GO:0005622 intracellular 0.000137 125 / 66331
GO:1990904 ribonucleoprotein co... 1.31e−12 26 / 2860
GO:0044424 intracellular part 0.000125 125 / 66260
GO:0043226 organelle 0.000185 115 / 58813
GO:0005737 cytoplasm 3.66e−13 111 / 42821
GO:0030529 intracellular ribonu... 1.31e−12 26 / 2860
GO:0044422 organelle part 8.76e−16 71 / 17309
GO:0043229 intracellular organe... 0.000181 115 / 58793
GO:0043228 non−membrane−bounded... 3.86e−09 30 / 5440
GO:0043227 membrane−bounded org... 0.007871 108 / 57760
GO:0044444 cytoplasmic part 1.67e−16 104 / 34514
GO:0044446 intracellular organe... 7.95e−16 71 / 17278
GO:0043232 intracellular non−me... 3.86e−09 30 / 5440
GO:0043231 intracellular membra... 0.006665 108 / 57558
GO:0005829 cytosol 9.64e−17 49 / 8016
GO:0005840 ribosome 1.01e−14 24 / 1899
GO:0009536 plastid 2.07e−08 50 / 13957
GO:0044445 cytosolic part 4.51e−18 25 / 1498
GO:0044435 plastid part 6.49e−10 30 / 5038
GO:0009507 chloroplast 1.17e−08 50 / 13722
GO:0022626 cytosolic ribosome 7.36e−17 23 / 1352
GO:0009532 plastid stroma 2.19e−13 27 / 2879
GO:0044434 chloroplast part 4.62e−10 30 / 4966
GO:0009570 chloroplast stroma 1.25e−13 27 / 2811
