# Supplementary material for: Differential Alternative Splicing Genes and Isoform Regulation Networks of Rapeseed (Brassica napus L.) Infected with Sclerotinia sclerotiorum
Source: Genes (Basel). 2020 Jul 13;11(7):784. doi: 10.3390/genes11070784 (PMC7397149; doi:10.3390/genes11070784)
Supplement: Supplementary file 1 [file genes-11-00784-s001.zip › Fig. S2 Enriched GO terms of genes in the 85 WGCNA modules.zip!/Fig. S2/CytoNodelavenderblush2-go.pdf]

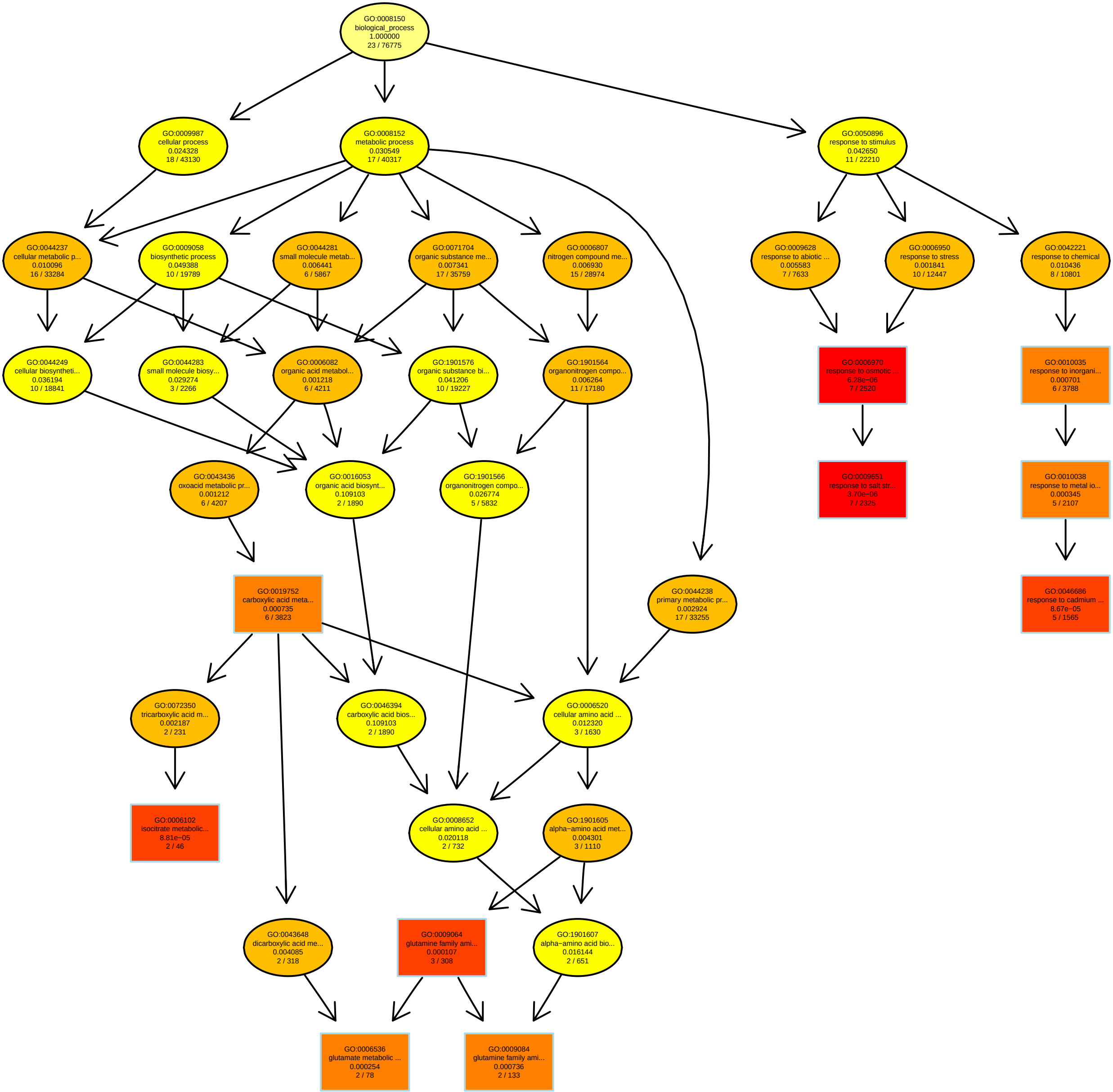

GO:0008150 biological_process 1.000000 23 / 76775
GO:0009987 cellular process 0.024328 18 / 43130
GO:0008152 metabolic process 0.030549 17 / 40317
GO:0050896 response to stimulus 0.042650 11 / 22210
GO:0044237 cellular metabolic p... 0.010096 16 / 33284
GO:0009058 biosynthetic process 0.049388 10 / 19789
GO:0044281 small molecule metab... 0.006441 6 / 5867
GO:0071704 organic substance me... 0.007341 17 / 35759
GO:0006807 nitrogen compound me... 0.006930 15 / 28974
GO:0009628 response to abiotic ... 0.005583 7 / 7633
GO:0006950 response to stress 0.001841 10 / 12447
GO:0042221 response to chemical 0.010436 8 / 10801
GO:0044249 cellular biosyntheti... 0.036194 10 / 18841
GO:0044283 small molecule biosy... 0.029274 3 / 2266
GO:0006082 organic acid metabol... 0.001218 6 / 4211
GO:1901576 organic substance bi... 0.041206 10 / 19227
GO:1901564 organonitrogen compo... 0.006264 11 / 17180
GO:0006970 response to osmotic ... 6.28e−06 7 / 2520
GO:0010035 response to inorgani... 0.000701 6 / 3788
GO:0043436 oxoacid metabolic pr... 0.001212 6 / 4207
GO:0016053 organic acid biosynt... 0.109103 2 / 1890
GO:1901566 organonitrogen compo... 0.026774 5 / 5832
GO:0009651 response to salt str... 3.70e−06 7 / 2325
GO:0010038 response to metal io... 0.000345 5 / 2107
GO:0019752 carboxylic acid meta... 0.000735 6 / 3823
GO:0044238 primary metabolic pr... 0.002924 17 / 33255
GO:0046686 response to cadmium ... 8.67e−05 5 / 1565
GO:0072350 tricarboxylic acid m... 0.002187 2 / 231
GO:0046394 carboxylic acid bios... 0.109103 2 / 1890
GO:0006520 cellular amino acid ... 0.012320 3 / 1630
GO:0006102 isocitrate metabolic... 8.81e−05 2 / 46
GO:0008652 cellular amino acid ... 0.020118 2 / 732
GO:1901605 alpha−amino acid met... 0.004301 3 / 1110
GO:0043648 dicarboxylic acid me... 0.004085 2 / 318
GO:0009064 glutamine family ami... 0.000107 3 / 308
GO:1901607 alpha−amino acid bio... 0.016144 2 / 651
GO:0006536 glutamate metabolic ... 0.000254 2 / 78
GO:0009084 glutamine family ami... 0.000736 2 / 133

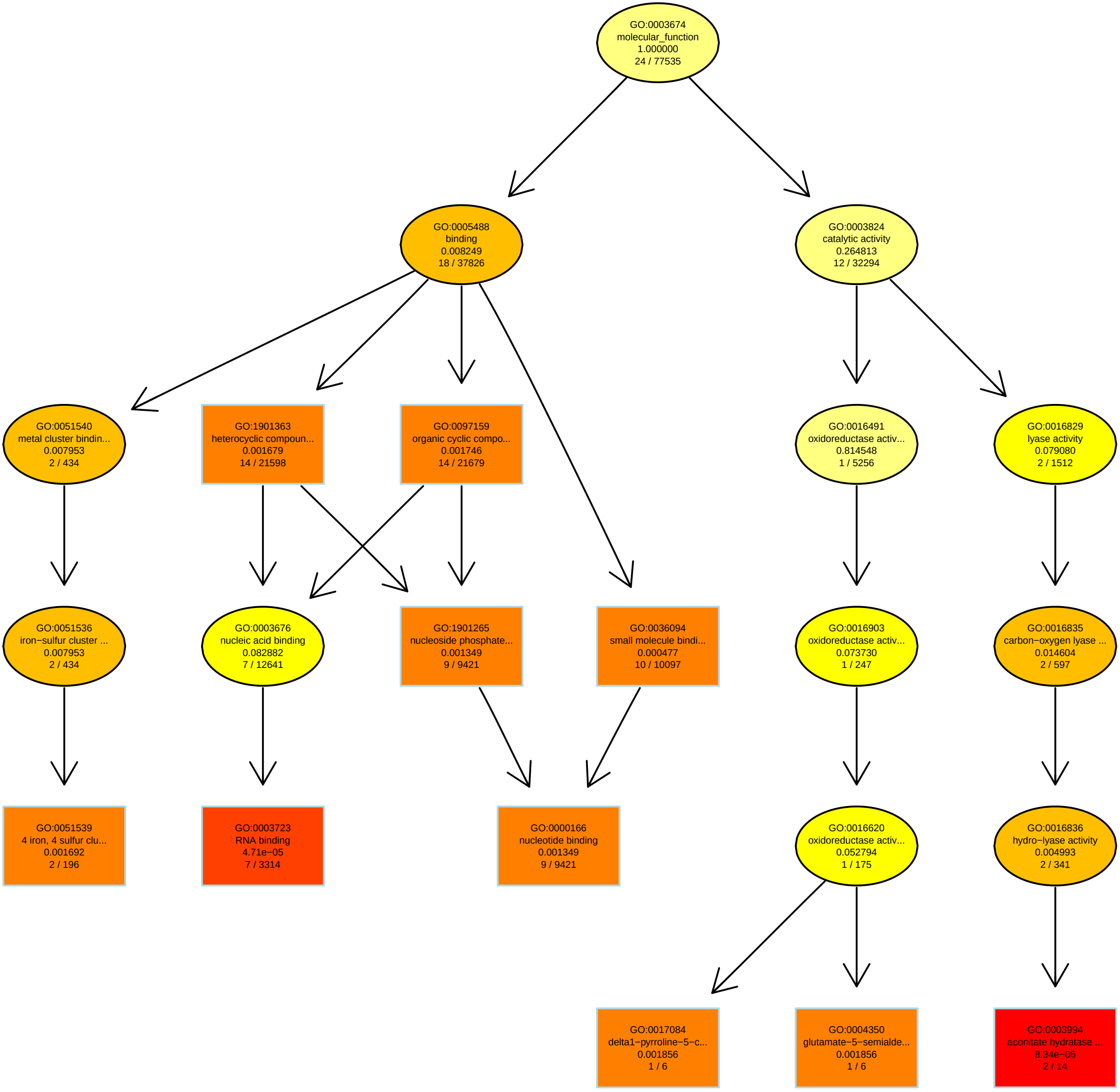
GO:0003674
molecular_function
1.000000
24 / 77535
GO:0005488
binding
0.008249
18 / 37826
GO:0003824
catalytic activity
0.264813
12 / 32294
GO:0051540
metal cluster bindin...
0.007953
2 / 434
GO:1901363
heterocyclic compoun...
0.001679
14 / 21598
GO:0097159
organic cyclic compo...
0.001746
14 / 21679
GO:0016491
oxidoreductase activ...
0.814548
1 / 5256
GO:0016829
lyase activity
0.079080
2 / 1512
GO:0051536
iron−sulfur cluster ...
0.007953
2 / 434
GO:0003676
nucleic acid binding
0.082882
7 / 12641
GO:1901265
nucleoside phosphate...
0.001349
9 / 9421
GO:0036094
small molecule bindi...
0.000477
10 / 10097
GO:0016903
oxidoreductase activ...
0.073730
1 / 247
GO:0016835
carbon−oxygen lyase ...
0.014604
2 / 597
GO:0051539
4 iron, 4 sulfur clu...
0.001692
2 / 196
GO:0003723
RNA binding
4.71e−05
7 / 3314
GO:0000166
nucleotide binding
0.001349
9 / 9421
GO:0016620
oxidoreductase activ...
0.052794
1 / 175
GO:0016836
hydro−lyase activity
0.004993
2 / 341
GO:0017084
delta1−pyrroline−5−c...
0.001856
1 / 6
GO:0004350
glutamate−5−semialde...
0.001856
1 / 6
GO:0003994
aconitate hydratase ...
8.34e−06
2 / 14

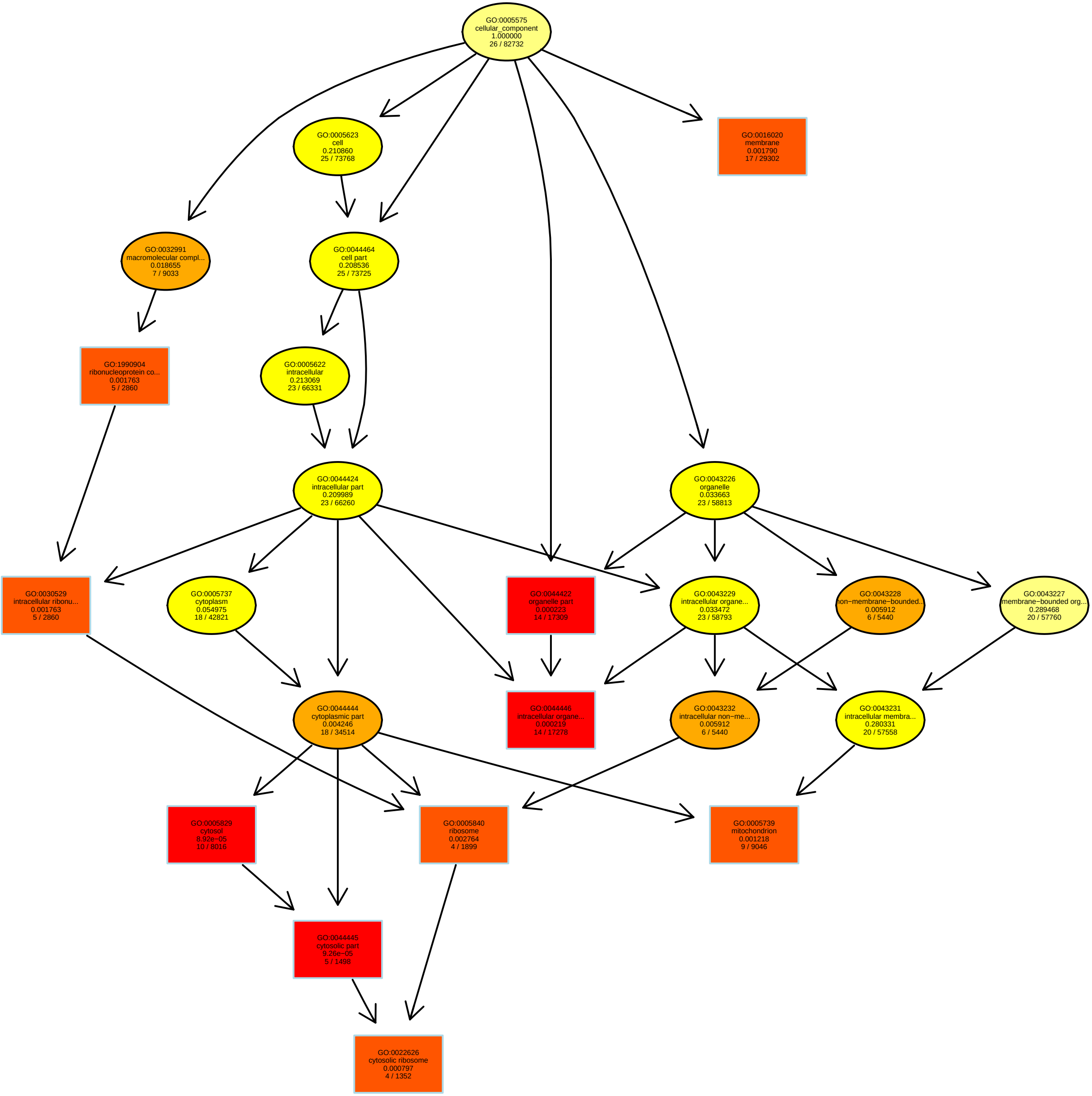
GO:0005575
cellular_component
1.000000
26 / 82732
GO:0005623
cell
0.210860
25 / 73768
GO:0016020
membrane
0.001790
17 / 29302
GO:0032991
macromolecular compl...
0.018655
7 / 9033
GO:0044464
cell part
0.208536
25 / 73725
GO:1990904
ribonucleoprotein co...
0.001763
5 / 2860
GO:0005622
intracellular
0.213069
23 / 66331
GO:0044424
intracellular part
0.209989
23 / 66260
GO:0043226
organelle
0.033663
23 / 58813
GO:0030529
intracellular ribonu...
0.001763
5 / 2860
GO:0005737
cytoplasm
0.054975
18 / 42821
GO:0044422
organelle part
0.000223
14 / 17309
GO:0043229
intracellular organe...
0.033472
23 / 58793
GO:0043228
non−membrane−bounded...
0.005912
6 / 5440
GO:0043227
membrane−bounded org...
0.289468
20 / 57760
GO:0044444
cytoplasmic part
0.004246
18 / 34514
GO:0044446
intracellular organe...
0.000219
14 / 17278
GO:0043232
intracellular non−me...
0.005912
6 / 5440
GO:0043231
intracellular membra...
0.280331
20 / 57558
GO:0005829
cytosol
8.92e−05
10 / 8016
GO:0005840
ribosome
0.002764
4 / 1899
GO:0005739
mitochondrion
0.001218
9 / 9046
GO:0044445
cytosolic part
9.26e−05
5 / 1498
GO:0022626
cytosolic ribosome
0.000797
4 / 1352
