# Supplementary material for: Differential Alternative Splicing Genes and Isoform Regulation Networks of Rapeseed (Brassica napus L.) Infected with Sclerotinia sclerotiorum
Source: Genes (Basel). 2020 Jul 13;11(7):784. doi: 10.3390/genes11070784 (PMC7397149; doi:10.3390/genes11070784)
Supplement: Supplementary file 1 [file genes-11-00784-s001.zip › Fig. S2 Enriched GO terms of genes in the 85 WGCNA modules.zip!/Fig. S2/CytoNodelavenderblush3-go.pdf]

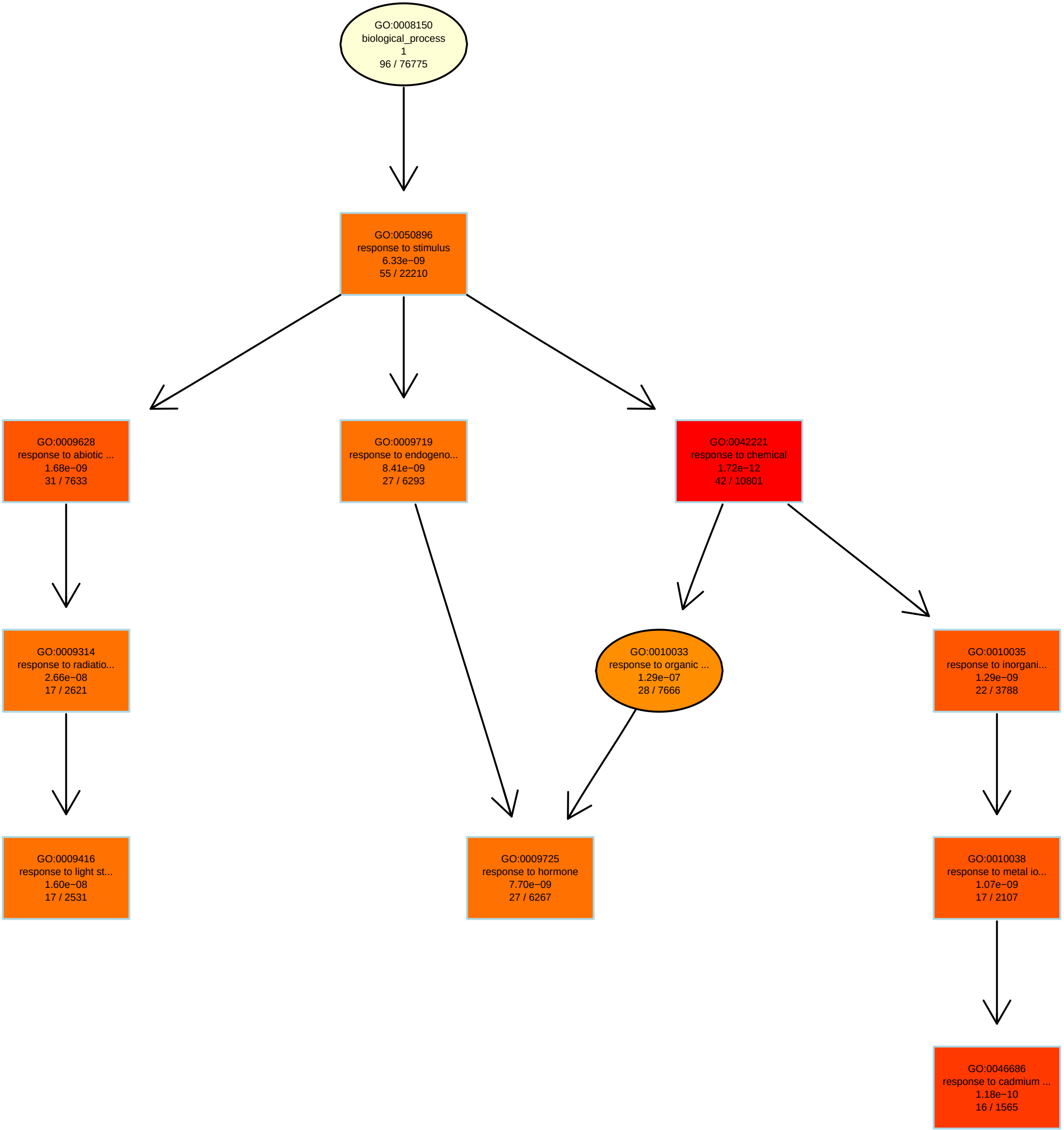
GO:0008150
biological_process
1
96 / 76775
GO:0050896
response to stimulus
6.33e−09
55 / 22210
GO:0009628
response to abiotic ...
1.68e−09
31 / 7633
GO:0009719
response to endogeno...
8.41e−09
27 / 6293
GO:0042221
response to chemical
1.72e−12
42 / 10801
GO:0009314
response to radiatio...
2.66e−08
17 / 2621
GO:0010033
response to organic ...
1.29e−07
28 / 7666
GO:0010035
response to inorgani...
1.29e−09
22 / 3788
GO:0009416
response to light st...
1.60e−08
17 / 2531
GO:0009725
response to hormone
7.70e−09
27 / 6267
GO:0010038
response to metal io...
1.07e−09
17 / 2107
GO:0046686
response to cadmium ...
1.18e−10
16 / 1565

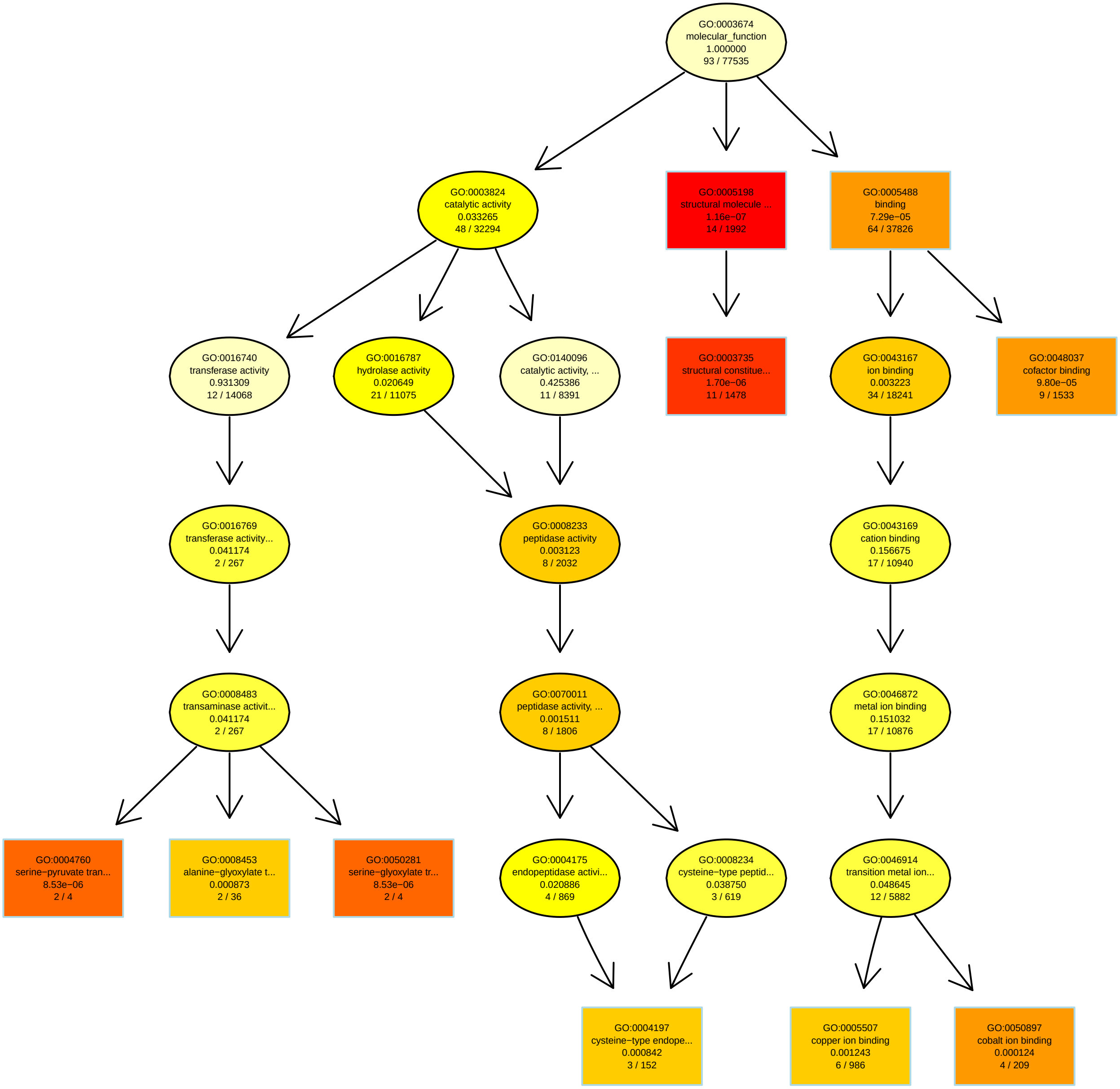
GO:0003674
molecular_function
1.000000
93 / 77535
GO:0003824
catalytic activity
0.033265
48 / 32294
GO:0005198
structural molecule ...
1.16e−07
14 / 1992
GO:0005488
binding
7.29e−05
64 / 37826
GO:0016740
transferase activity
0.931309
12 / 14068
GO:0016787
hydrolase activity
0.020649
21 / 11075
GO:0140096
catalytic activity, ...
0.425386
11 / 8391
GO:0003735
structural constitue...
1.70e−06
11 / 1478
GO:0043167
ion binding
0.003223
34 / 18241
GO:0048037
cofactor binding
9.80e−05
9 / 1533
GO:0016769
transferase activity...
0.041174
2 / 267
GO:0008233
peptidase activity
0.003123
8 / 2032
GO:0043169
cation binding
0.156675
17 / 10940
GO:0008483
transaminase activit...
0.041174
2 / 267
GO:0070011
peptidase activity, ...
0.001511
8 / 1806
GO:0046872
metal ion binding
0.151032
17 / 10876
GO:0004760
serine−pyruvate tran...
8.53e−06
2 / 4
GO:0008453
alanine−glyoxylate t...
0.000873
2 / 36
GO:0050281
serine−glyoxylate tr...
8.53e−06
2 / 4
GO:0004175
endopeptidase activi...
0.020886
4 / 869
GO:0008234
cysteine−type peptid...
0.038750
3 / 619
GO:0046914
transition metal ion...
0.048645
12 / 5882
GO:0004197
cysteine−type endope...
0.000842
3 / 152
GO:0005507
copper ion binding
0.001243
6 / 986
GO:0050897
cobalt ion binding
0.000124
4 / 209

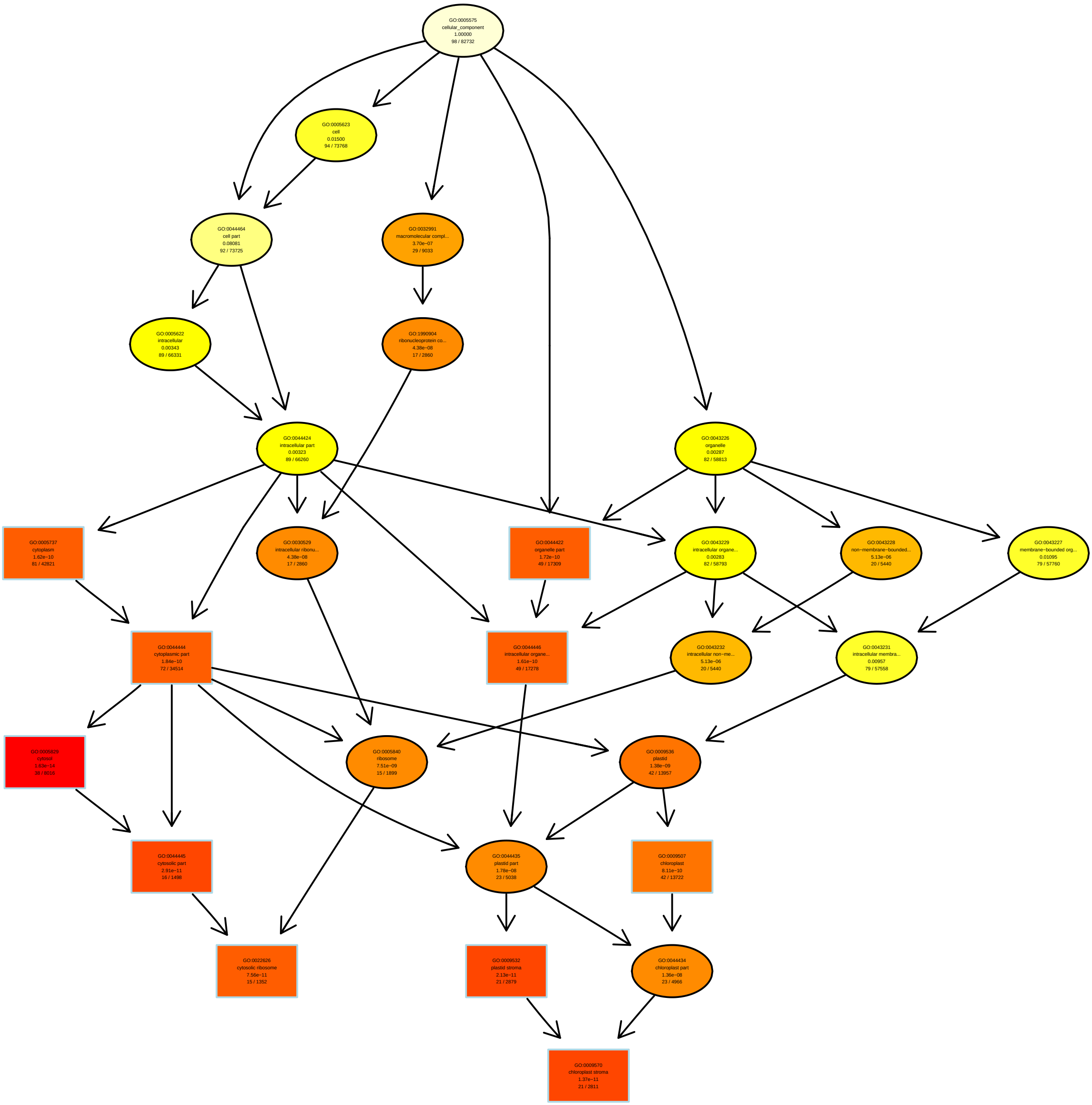
GO:0005575
cellular_component
1.00000
98 / 82732
GO:0005623
cell
0.01500
94 / 73768
GO:0044464
cell part
0.08081
92 / 73725
GO:0032991
macromolecular compl...
3.70e−07
29 / 9033
GO:0005622
intracellular
0.00343
89 / 66331
GO:1990904
ribonucleoprotein co...
4.38e−08
17 / 2860
GO:0044424
intracellular part
0.00323
89 / 66260
GO:0043226
organelle
0.00287
82 / 58813
GO:0005737
cytoplasm
1.62e−10
81 / 42821
GO:0030529
intracellular ribonu...
4.38e−08
17 / 2860
GO:0044422
organelle part
1.72e−10
49 / 17309
GO:0043229
intracellular organe...
0.00283
82 / 58793
GO:0043228
non−membrane−bounded...
5.13e−06
20 / 5440
GO:0043227
membrane−bounded org...
0.01095
79 / 57760
GO:0044444
cytoplasmic part
1.84e−10
72 / 34514
GO:0044446
intracellular organe...
1.61e−10
49 / 17278
GO:0043232
intracellular non−me...
5.13e−06
20 / 5440
GO:0043231
intracellular membra...
0.00957
79 / 57558
GO:0005829
cytosol
1.63e−14
38 / 8016
GO:0005840
ribosome
7.51e−09
15 / 1899
GO:0009536
plastid
1.38e−09
42 / 13957
GO:0044445
cytosolic part
2.91e−11
16 / 1498
GO:0044435
plastid part
1.78e−08
23 / 5038
GO:0009507
chloroplast
8.11e−10
42 / 13722
GO:0022626
cytosolic ribosome
7.56e−11
15 / 1352
GO:0009532
plastid stroma
2.13e−11
21 / 2879
GO:0044434
chloroplast part
1.36e−08
23 / 4966
GO:0009570
chloroplast stroma
1.37e−11
21 / 2811
