# Supplementary material for: Differential Alternative Splicing Genes and Isoform Regulation Networks of Rapeseed (Brassica napus L.) Infected with Sclerotinia sclerotiorum
Source: Genes (Basel). 2020 Jul 13;11(7):784. doi: 10.3390/genes11070784 (PMC7397149; doi:10.3390/genes11070784)
Supplement: Supplementary file 1 [file genes-11-00784-s001.zip › Fig. S2 Enriched GO terms of genes in the 85 WGCNA modules.zip!/Fig. S2/CytoNodelightcoral-go.pdf]

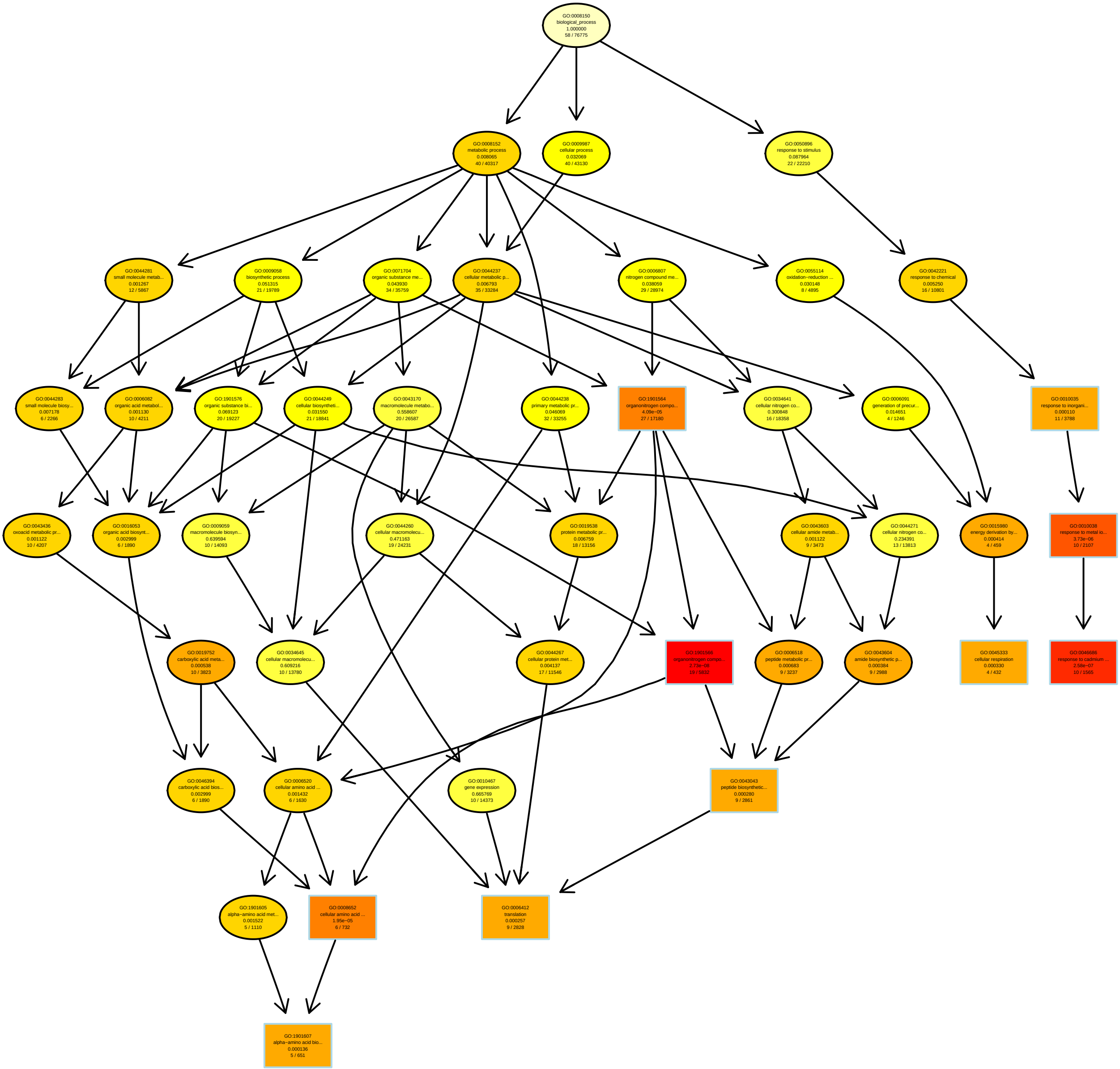
GO:0008150 biological_process 1.000000 58 / 76775
GO:0008152 metabolic process 0.008065 40 / 40317
GO:0009987 cellular process 0.032069 40 / 43130
GO:0050896 response to stimulus 0.087964 22 / 22210
GO:0044281 small molecule metab... 0.001267 12 / 5867
GO:0009058 biosynthetic process 0.051315 21 / 19789
GO:0071704 organic substance me... 0.043930 34 / 35759
GO:0044237 cellular metabolic p... 0.006793 35 / 33284
GO:0006807 nitrogen compound me... 0.038059 29 / 28974
GO:0055114 oxidation−reduction ... 0.030148 8 / 4895
GO:0042221 response to chemical 0.005250 16 / 10801
GO:0044283 small molecule biosy... 0.007178 6 / 2266
GO:0006082 organic acid metabol... 0.001130 10 / 4211
GO:1901576 organic substance bi... 0.069123 20 / 19227
GO:0044249 cellular biosyntheti... 0.031550 21 / 18841
GO:0043170 macromolecule metabo... 0.558607 20 / 26587
GO:0044238 primary metabolic pr... 0.046069 32 / 33255
GO:1901564 organonitrogen compo... 4.09e−05 27 / 17180
GO:0034641 cellular nitrogen co... 0.300848 16 / 18358
GO:0006091 generation of precur... 0.014651 4 / 1246
GO:0010035 response to inorgani... 0.000110 11 / 3788
GO:0043436 oxoacid metabolic pr... 0.001122 10 / 4207
GO:0016053 organic acid biosynt... 0.002999 6 / 1890
GO:0009059 macromolecule biosyn... 0.639594 10 / 14093
GO:0044260 cellular macromolecu... 0.471163 19 / 24231
GO:0019538 protein metabolic pr... 0.006759 18 / 13156
GO:0043603 cellular amide metab... 0.001122 9 / 3473
GO:0044271 cellular nitrogen co... 0.234391 13 / 13813
GO:0015980 energy derivation by... 0.000414 4 / 459
GO:0010038 response to metal io... 3.73e−06 10 / 2107
GO:0019752 carboxylic acid meta... 0.000538 10 / 3823
GO:0034645 cellular macromolecu... 0.609216 10 / 13780
GO:0044267 cellular protein met... 0.004137 17 / 11546
GO:1901566 organonitrogen compo... 2.73e−06 18 / 5832
GO:0006518 peptide metabolic pr... 0.000683 9 / 3237
GO:0043604 amide biosynthetic p... 0.000384 9 / 2988
GO:0045333 cellular respiration 0.000330 4 / 432
GO:0046686 response to cadmium ... 2.58e−07 10 / 1565
GO:0046394 carboxylic acid bios... 0.002999 6 / 1890
GO:0006520 cellular amino acid ... 0.001432 6 / 1630
GO:0010467 gene expression 0.665769 10 / 14373
GO:0043043 peptide biosynthetic... 0.000280 9 / 2861
GO:1901605 alpha−amino acid met... 0.001522 5 / 1110
GO:0008652 cellular amino acid ... 1.95e−05 6 / 732
GO:0006412 translation 0.000257 9 / 2828
GO:1901607 alpha−amino acid bio... 0.000136 5 / 651

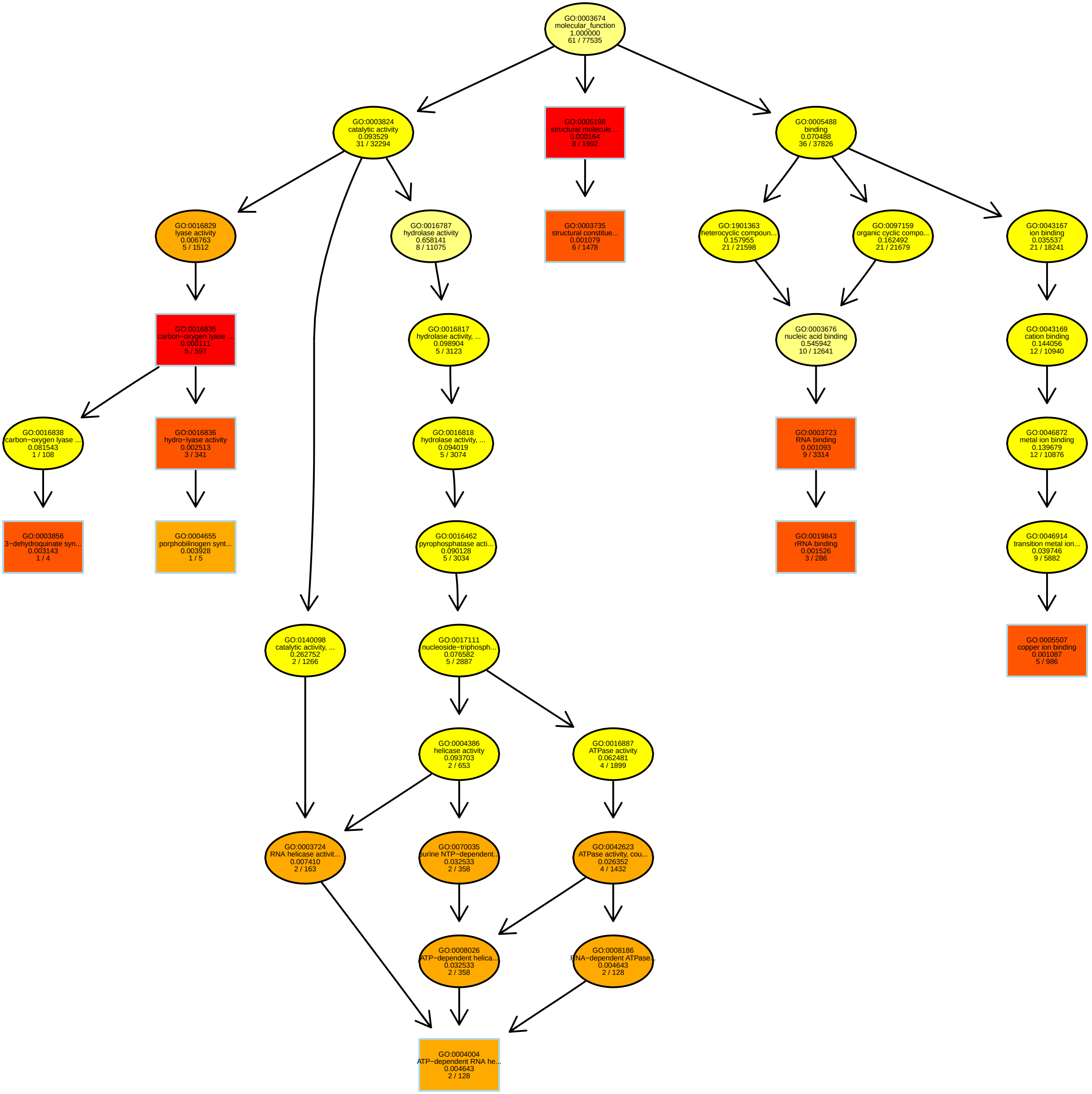
GO:0003674 molecular_function 1.000000 61 / 77535
GO:0003824 catalytic activity 0.093529 31 / 32294
GO:0005198 structural molecule ... 0.000164 8 / 1992
GO:0005488 binding 0.070488 36 / 37826
GO:0016829 lyase activity 0.006763 5 / 1512
GO:0016787 hydrolase activity 0.658141 8 / 11075
GO:0003735 structural constitue... 0.001079 6 / 1478
GO:1901363 heterocyclic compoun... 0.157955 21 / 21598
GO:0097159 organic cyclic compo... 0.162492 21 / 21679
GO:0043167 ion binding 0.035537 21 / 18241
GO:0016835 carbon−oxygen lyase ... 0.000111 5 / 597
GO:0016817 hydrolase activity, ... 0.098904 5 / 3123
GO:0003676 nucleic acid binding 0.545942 10 / 12641
GO:0043169 cation binding 0.144056 12 / 10940
GO:0016838 carbon−oxygen lyase ... 0.081543 1 / 108
GO:0016836 hydro−lyase activity 0.002513 3 / 341
GO:0016818 hydrolase activity, ... 0.094019 5 / 3074
GO:0003723 RNA binding 0.001093 9 / 3314
GO:0046872 metal ion binding 0.139679 12 / 10876
GO:0003856 3−dehydroquinate syn... 0.003143 1 / 4
GO:0004655 porphobilinogen synt... 0.003928 1 / 5
GO:0016462 pyrophosphatase acti... 0.090128 5 / 3034
GO:0019843 rRNA binding 0.001526 3 / 286
GO:0046914 transition metal ion... 0.039746 9 / 5882
GO:0140098 catalytic activity, ... 0.262752 2 / 1266
GO:0017111 nucleoside−triphosph... 0.076582 5 / 2887
GO:0005507 copper ion binding 0.001087 5 / 986
GO:0004386 helicase activity 0.093703 2 / 653
GO:0016887 ATPase activity 0.062481 4 / 1899
GO:0003724 RNA helicase activit... 0.007410 2 / 163
GO:0070035 purine NTP−dependent... 0.032533 2 / 358
GO:0042623 ATPase activity, cou... 0.026352 4 / 1432
GO:0008026 ATP−dependent helica... 0.032533 2 / 358
GO:0008186 RNA−dependent ATPase... 0.004643 2 / 128
GO:0004004 ATP−dependent RNA he... 0.004643 2 / 128

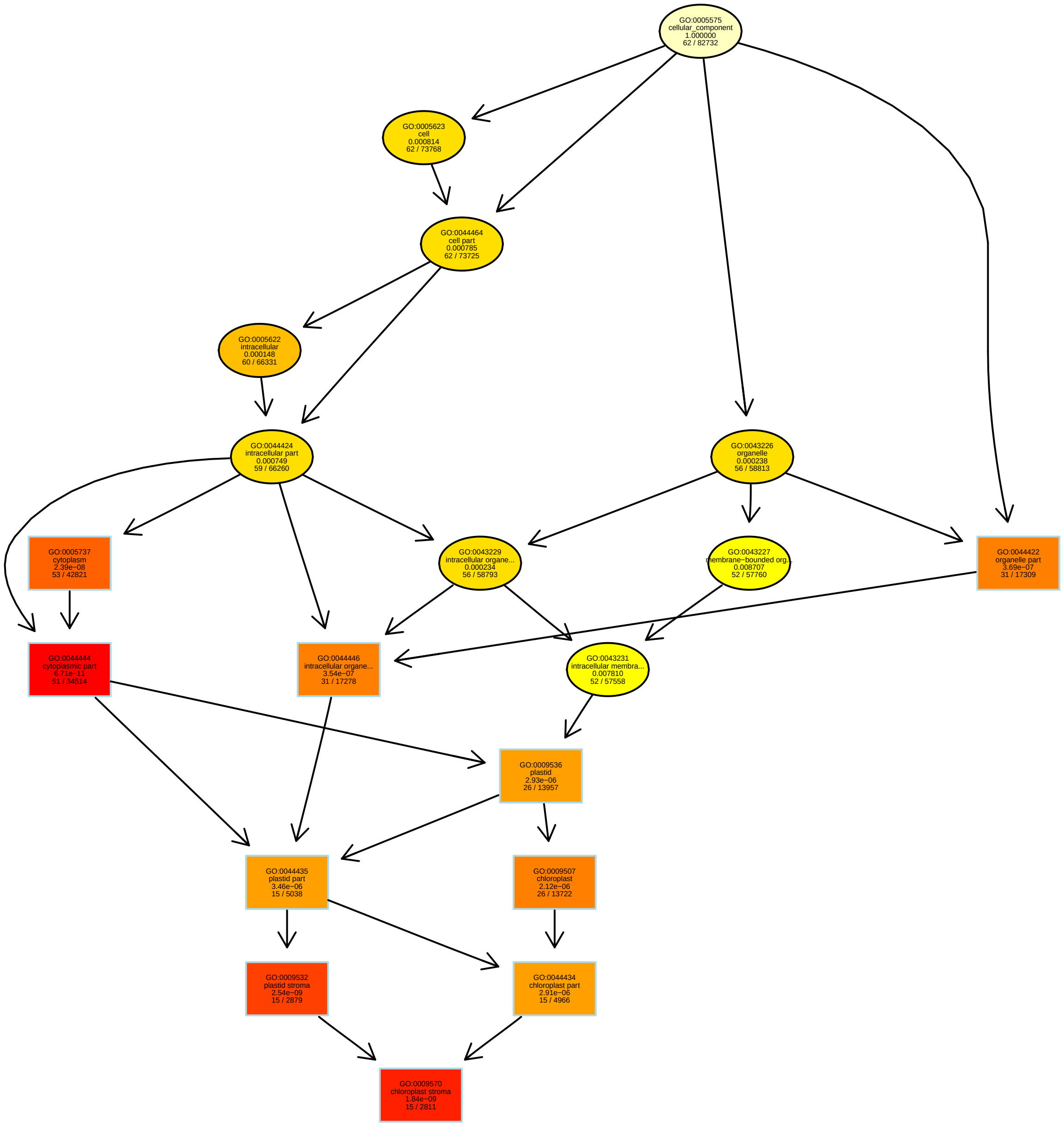
GO:0005575
cellular_component
1.000000
62 / 82732
GO:0005623
cell
0.000814
62 / 73768
GO:0044464
cell part
0.000785
62 / 73725
GO:0005622
intracellular
0.000148
60 / 66331
GO:0044424
intracellular part
0.000749
59 / 66260
GO:0043226
organelle
0.000238
56 / 58813
GO:0005737
cytoplasm
2.39e−08
53 / 42821
GO:0043229
intracellular organe...
0.000234
56 / 58793
GO:0043227
membrane−bounded org...
0.008707
52 / 57760
GO:0044422
organelle part
3.69e−07
31 / 17309
GO:0044444
cytoplasmic part
6.71e−11
51 / 34514
GO:0044446
intracellular organe...
3.54e−07
31 / 17278
GO:0043231
intracellular membra...
0.007810
52 / 57558
GO:0009536
plastid
2.93e−06
26 / 13957
GO:0044435
plastid part
3.46e−06
15 / 5038
GO:0009507
chloroplast
2.12e−06
26 / 13722
GO:0009532
plastid stroma
2.54e−09
15 / 2879
GO:0044434
chloroplast part
2.91e−06
15 / 4966
GO:0009570
chloroplast stroma
1.84e−09
15 / 2811
