# Supplementary material for: Differential Alternative Splicing Genes and Isoform Regulation Networks of Rapeseed (Brassica napus L.) Infected with Sclerotinia sclerotiorum
Source: Genes (Basel). 2020 Jul 13;11(7):784. doi: 10.3390/genes11070784 (PMC7397149; doi:10.3390/genes11070784)
Supplement: Supplementary file 1 [file genes-11-00784-s001.zip › Fig. S2 Enriched GO terms of genes in the 85 WGCNA modules.zip!/Fig. S2/CytoNodelightcyan-go.pdf]

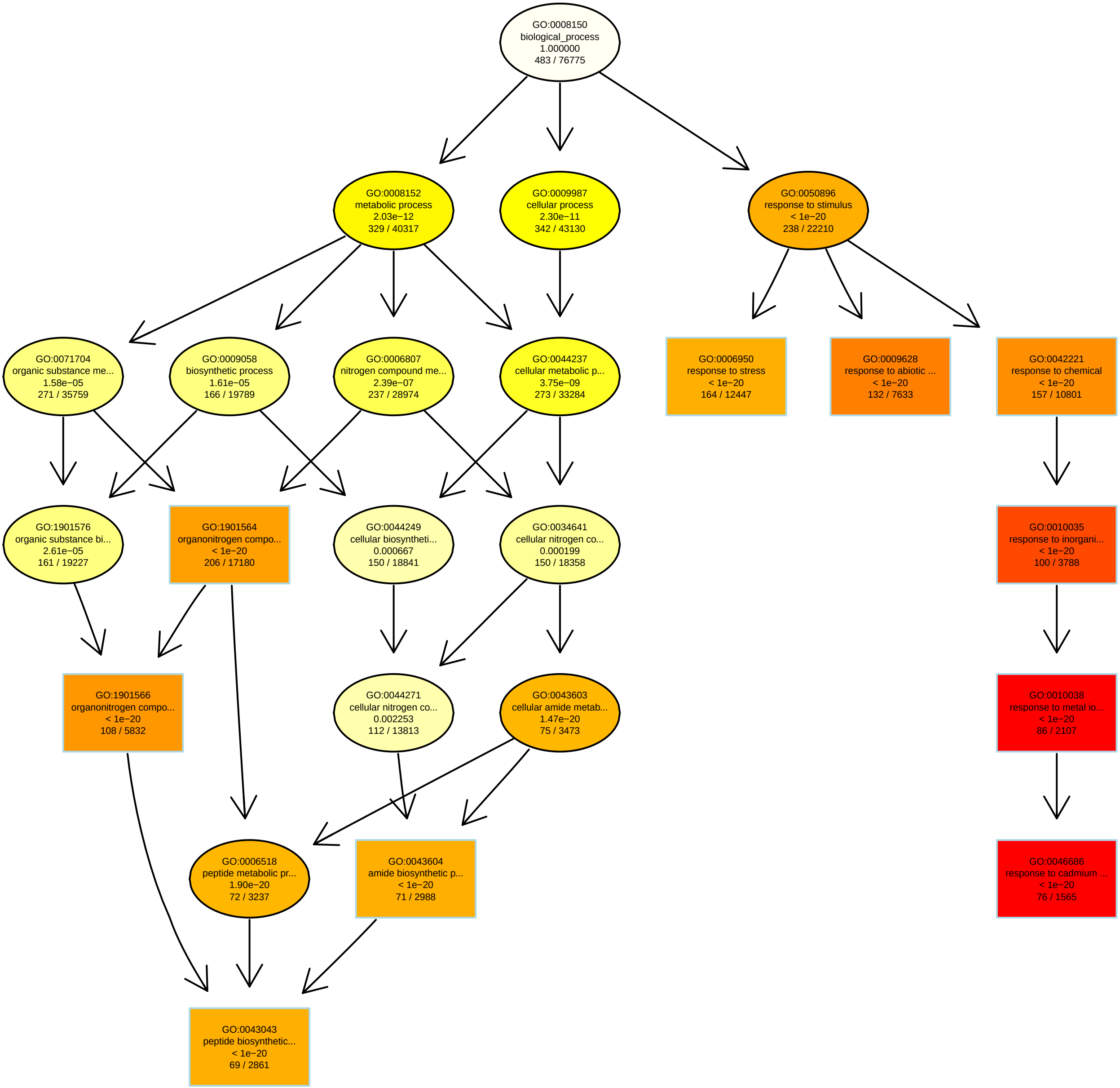

GO:0008150
biological_process
1.000000
483 / 76775
GO:0008152
metabolic process
2.03e−12
329 / 40317
GO:0009987
cellular process
2.30e−11
342 / 43130
GO:0050896
response to stimulus
< 1e−20
238 / 22210
GO:0071704
organic substance me...
1.58e−05
271 / 35759
GO:0009058
biosynthetic process
1.61e−05
166 / 19789
GO:0006807
nitrogen compound me...
2.39e−07
237 / 28974
GO:0044237
cellular metabolic p...
3.75e−09
273 / 33284
GO:0006950
response to stress
< 1e−20
164 / 12447
GO:0009628
response to abiotic ...
< 1e−20
132 / 7633
GO:0042221
response to chemical
< 1e−20
157 / 10801
GO:1901576
organic substance bi...
2.61e−05
161 / 19227
GO:1901564
organonitrogen compo...
< 1e−20
206 / 17180
GO:0044249
cellular biosyntheti...
0.000667
150 / 18841
GO:0034641
cellular nitrogen co...
0.000199
150 / 18358
GO:0010035
response to inorgani...
< 1e−20
100 / 3788
GO:1901566
organonitrogen compo...
< 1e−20
108 / 5832
GO:0044271
cellular nitrogen co...
0.002253
112 / 13813
GO:0043603
cellular amide metab...
1.47e−20
75 / 3473
GO:0010038
response to metal io...
< 1e−20
86 / 2107
GO:0006518
peptide metabolic pr...
1.90e−20
72 / 3237
GO:0043604
amide biosynthetic p...
< 1e−20
71 / 2988
GO:0046686
response to cadmium ...
< 1e−20
76 / 1565
GO:0043043
peptide biosynthetic...
< 1e−20
69 / 2861

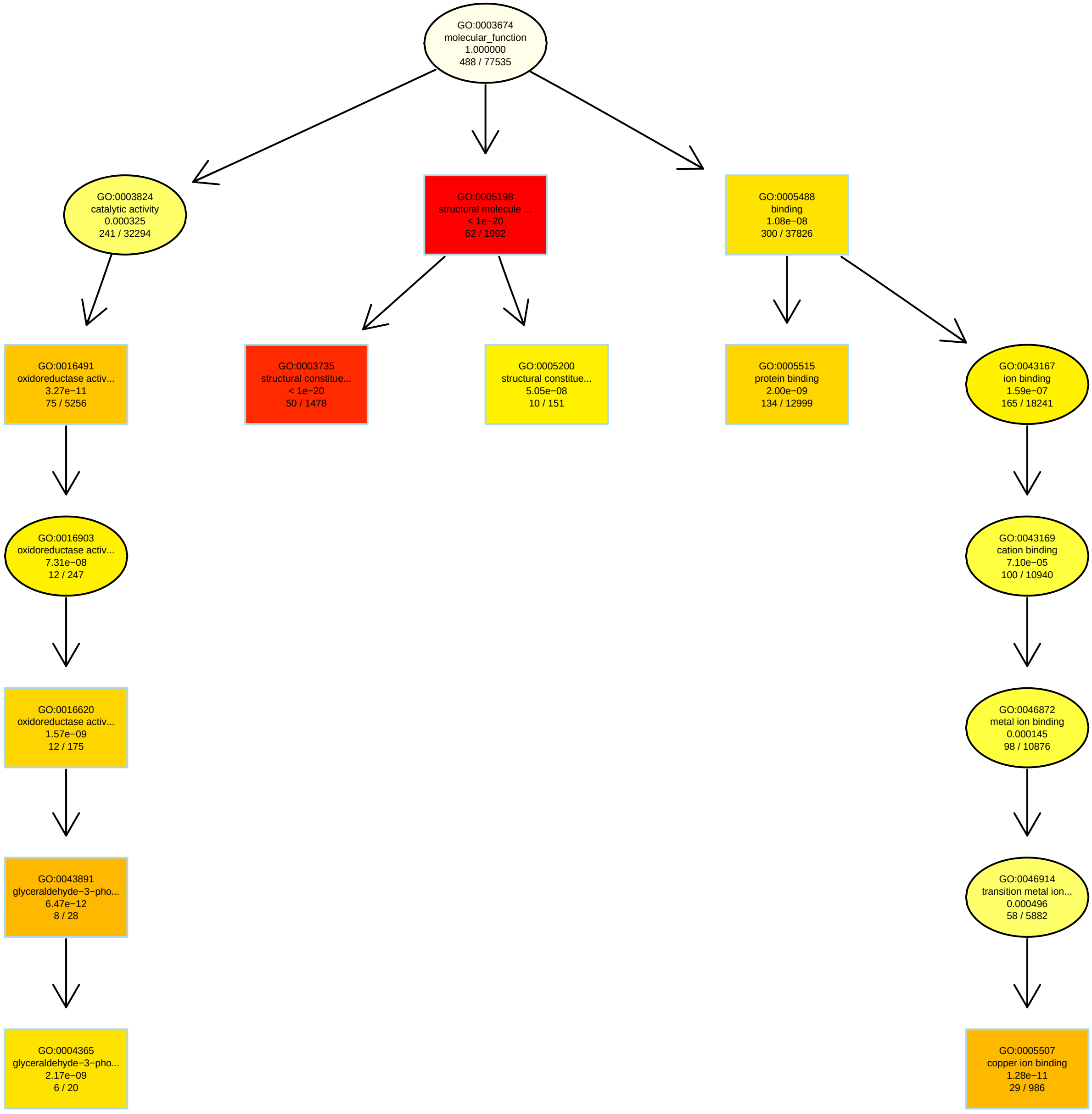
GO:0003674
molecular_function
1.000000
488 / 77535
GO:0003824
catalytic activity
0.000325
241 / 32294
GO:0005198
structural molecule ...
< 1e−20
62 / 1992
GO:0005488
binding
1.08e−08
300 / 37826
GO:0016491
oxidoreductase activ...
3.27e−11
75 / 5256
GO:0003735
structural constitue...
< 1e−20
50 / 1478
GO:0005200
structural constitue...
5.05e−08
10 / 151
GO:0005515
protein binding
2.00e−09
134 / 12999
GO:0043167
ion binding
1.59e−07
165 / 18241
GO:0016903
oxidoreductase activ...
7.31e−08
12 / 247
GO:0043169
cation binding
7.10e−05
100 / 10940
GO:0016620
oxidoreductase activ...
1.57e−09
12 / 175
GO:0046872
metal ion binding
0.000145
98 / 10876
GO:0043891
glyceraldehyde−3−pho...
6.47e−12
8 / 28
GO:0046914
transition metal ion...
0.000496
58 / 5882
GO:0004365
glyceraldehyde−3−pho...
2.17e−09
6 / 20
GO:0005507
copper ion binding
1.28e−11
29 / 986

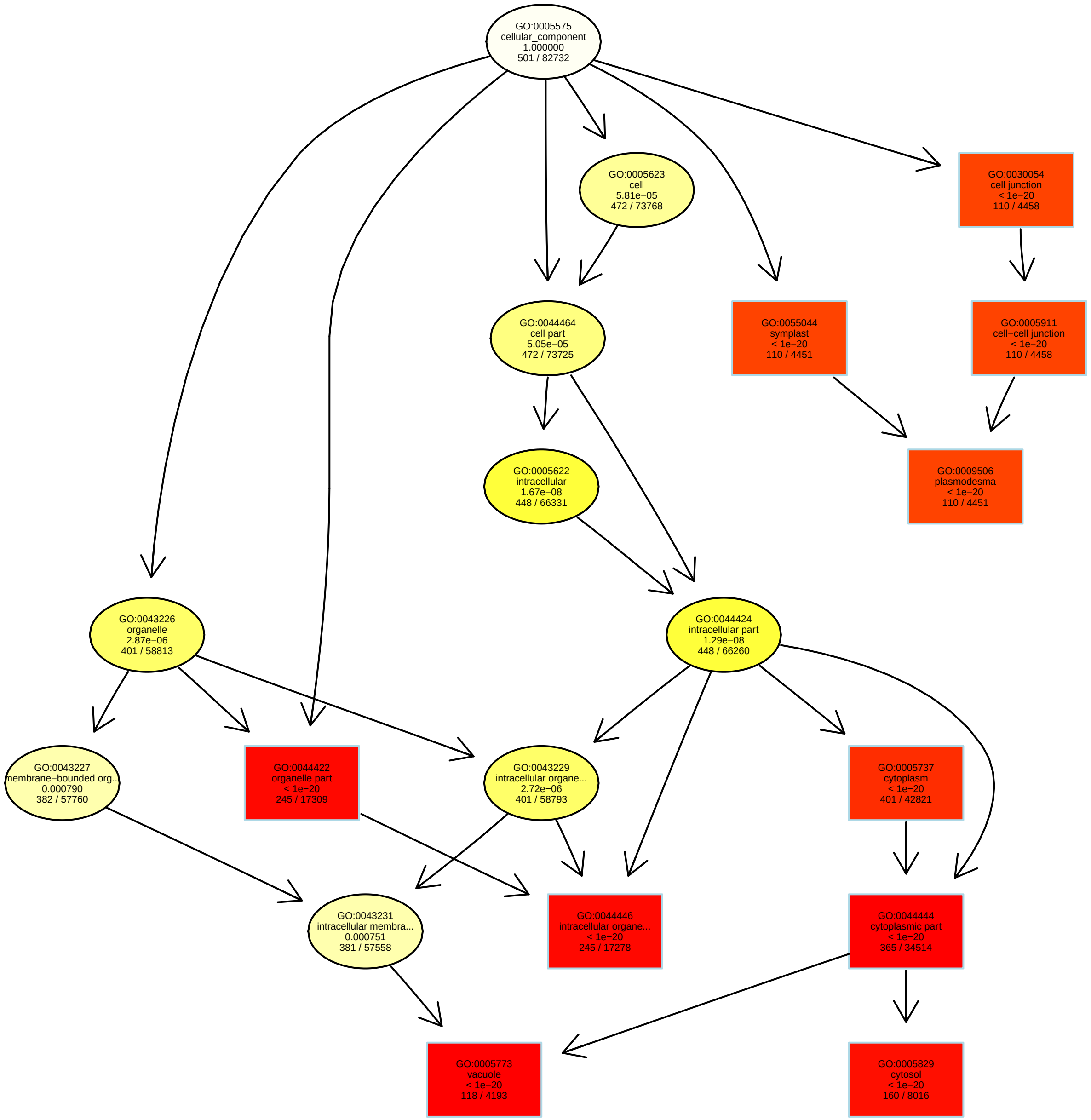
GO:0005575
cellular_component
1.000000
501 / 82732
GO:0005623
cell
5.81e−05
472 / 73768
GO:0030054
cell junction
< 1e−20
110 / 4458
GO:0044464
cell part
5.05e−05
472 / 73725
GO:0055044
symplast
< 1e−20
110 / 4451
GO:0005911
cell−cell junction
< 1e−20
110 / 4458
GO:0005622
intracellular
1.67e−08
448 / 66331
GO:0009506
plasmodesma
< 1e−20
110 / 4451
GO:0043226
organelle
2.87e−06
401 / 58813
GO:0044424
intracellular part
1.29e−08
448 / 66260
GO:0043227
membrane−bounded org...
0.000790
382 / 57760
GO:0044422
organelle part
< 1e−20
245 / 17309
GO:0043229
intracellular organe...
2.72e−06
401 / 58793
GO:0005737
cytoplasm
< 1e−20
401 / 42821
GO:0043231
intracellular membra...
0.000751
381 / 57558
GO:0044446
intracellular organe...
< 1e−20
245 / 17278
GO:0044444
cytoplasmic part
< 1e−20
365 / 34514
GO:0005773
vacuole
< 1e−20
118 / 4193
GO:0005829
cytosol
< 1e−20
160 / 8016
