# Supplementary material for: Differential Alternative Splicing Genes and Isoform Regulation Networks of Rapeseed (Brassica napus L.) Infected with Sclerotinia sclerotiorum
Source: Genes (Basel). 2020 Jul 13;11(7):784. doi: 10.3390/genes11070784 (PMC7397149; doi:10.3390/genes11070784)
Supplement: Supplementary file 1 [file genes-11-00784-s001.zip › Fig. S2 Enriched GO terms of genes in the 85 WGCNA modules.zip!/Fig. S2/CytoNodelightcyan1-go.pdf]

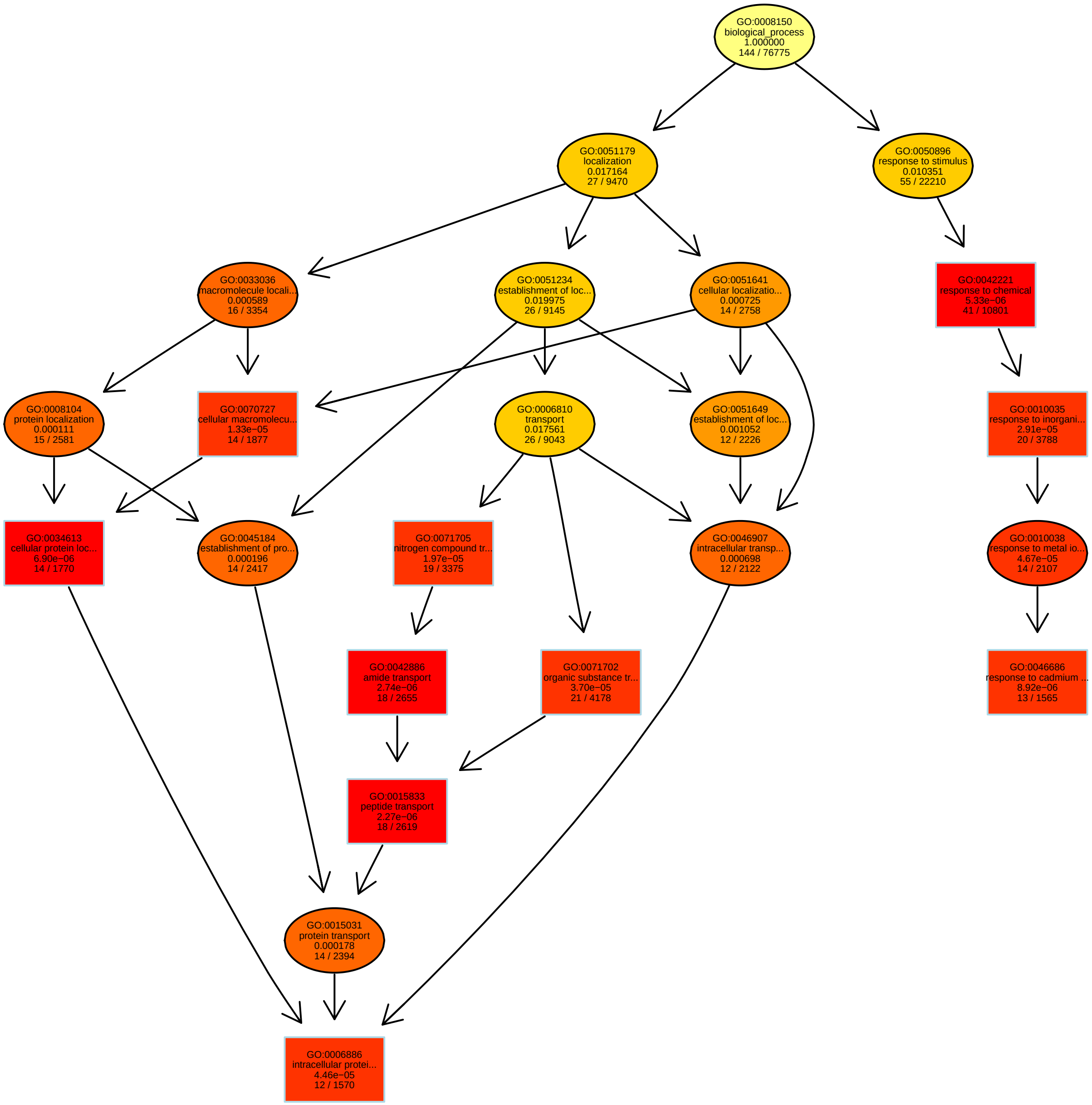
GO:0008150
biological_process
1.000000
144 / 76775
GO:0051179
localization
0.017164
27 / 9470
GO:0050896
response to stimulus
0.010351
55 / 22210
GO:0033036
macromolecule locali...
0.000589
16 / 3354
GO:0051234
establishment of loc...
0.019975
26 / 9145
GO:0051641
cellular localizatio...
0.000725
14 / 2758
GO:0042221
response to chemical
5.33e−06
41 / 10801
GO:0008104
protein localization
0.000111
15 / 2581
GO:0070727
cellular macromolecu...
1.33e−05
14 / 1877
GO:0006810
transport
0.017561
26 / 9043
GO:0051649
establishment of loc...
0.001052
12 / 2226
GO:0010035
response to inorgani...
2.91e−05
20 / 3788
GO:0034613
cellular protein loc...
6.90e−06
14 / 1770
GO:0045184
establishment of pro...
0.000196
14 / 2417
GO:0071705
nitrogen compound tr...
1.97e−05
19 / 3375
GO:0046907
intracellular transp...
0.000698
12 / 2122
GO:0010038
response to metal io...
4.67e−05
14 / 2107
GO:0042886
amide transport
2.74e−06
18 / 2655
GO:0071702
organic substance tr...
3.70e−05
21 / 4178
GO:0046686
response to cadmium ...
8.92e−06
13 / 1565
GO:0015833
peptide transport
2.27e−06
18 / 2619
GO:0015031
protein transport
0.000178
14 / 2394
GO:0006886
intracellular protei...
4.46e−05
12 / 1570

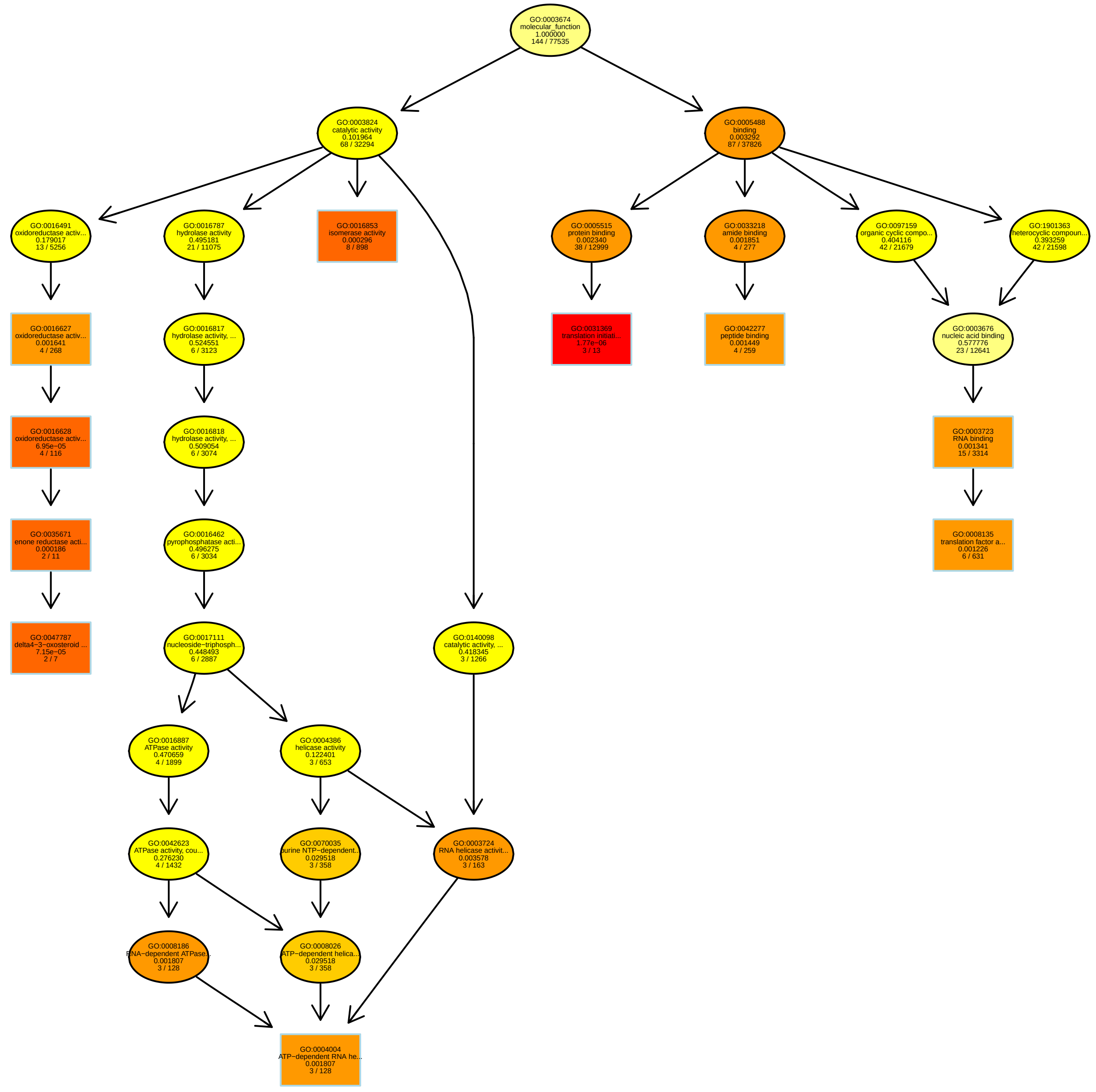
GO:0003674 molecular_function 1.000000 144 / 77535
GO:0003824 catalytic activity 0.101964 68 / 32294
GO:0005488 binding 0.003292 87 / 37826
GO:0016491 oxidoreductase activ... 0.179017 13 / 5256
GO:0016787 hydrolase activity 0.495181 21 / 11075
GO:0016853 isomerase activity 0.000296 8 / 898
GO:0005515 protein binding 0.002340 38 / 12999
GO:0033218 amide binding 0.001851 4 / 277
GO:0097159 organic cyclic compo... 0.404116 42 / 21679
GO:1901363 heterocyclic compoun... 0.393259 42 / 21598
GO:0016627 oxidoreductase activ... 0.001641 4 / 268
GO:0016817 hydrolase activity, ... 0.524551 6 / 3123
GO:0031369 translation initiati... 1.77e−06 3 / 13
GO:0042277 peptide binding 0.001449 4 / 259
GO:0003676 nucleic acid binding 0.577776 23 / 12641
GO:0016628 oxidoreductase activ... 6.95e−05 4 / 116
GO:0016818 hydrolase activity, ... 0.509054 6 / 3074
GO:0003723 RNA binding 0.001341 15 / 3314
GO:0035671 enone reductase acti... 0.000186 2 / 11
GO:0016462 pyrophosphatase acti... 0.496275 6 / 3034
GO:0008135 translation factor a... 0.001226 6 / 631
GO:0047787 delta4−3−oxosteroid ... 7.15e−05 2 / 7
GO:0017111 nucleoside−triphosph... 0.448493 6 / 2887
GO:0140098 catalytic activity, ... 0.418345 3 / 1266
GO:0016887 ATPase activity 0.470659 4 / 1899
GO:0004386 helicase activity 0.122401 3 / 653
GO:0042623 ATPase activity, cou... 0.276230 4 / 1432
GO:0070035 purine NTP−dependent... 0.029518 3 / 358
GO:0003724 RNA helicase activit... 0.003578 3 / 163
GO:0008186 RNA−dependent ATPase... 0.001807 3 / 128
GO:0008026 ATP−dependent helica... 0.029518 3 / 358
GO:0004004 ATP−dependent RNA he... 0.001807 3 / 128

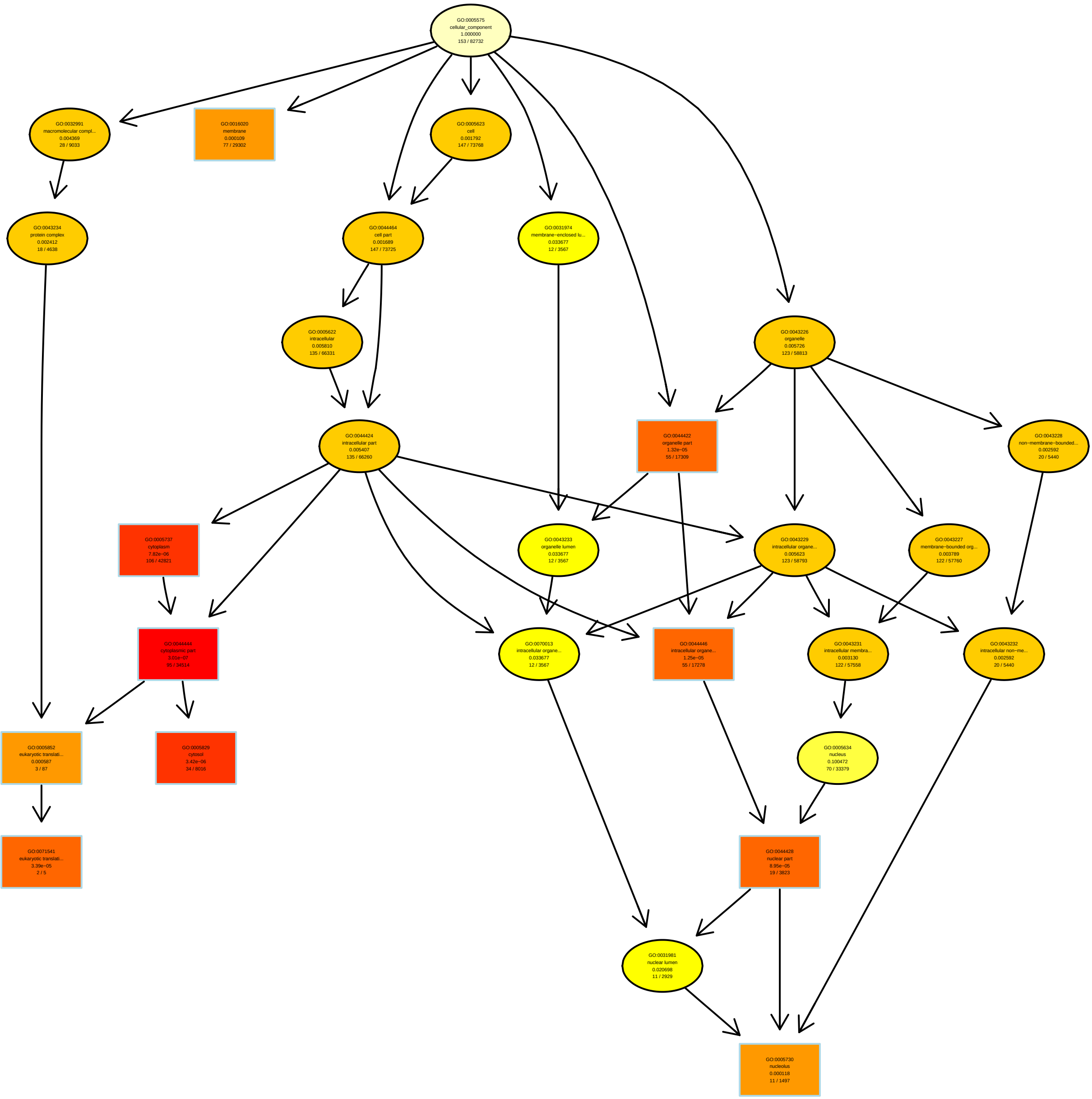
GO:0005575
cellular_component
1.000000
153 / 82732
GO:0032991
macromolecular compl...
0.004369
28 / 9033
GO:0016020
membrane
0.000109
77 / 29302
GO:0005623
cell
0.001792
147 / 73768
GO:0043234
protein complex
0.002412
18 / 4638
GO:0044464
cell part
0.001689
147 / 73725
GO:0031974
membrane−enclosed lu...
0.033677
12 / 3567
GO:0005622
intracellular
0.005810
135 / 66331
GO:0043226
organelle
0.005726
123 / 58813
GO:0044424
intracellular part
0.005407
135 / 66260
GO:0044422
organelle part
1.32e−05
55 / 17309
GO:0043228
non−membrane−bounded...
0.002592
20 / 5440
GO:0005737
cytoplasm
7.82e−06
106 / 42821
GO:0043233
organelle lumen
0.033677
12 / 3567
GO:0043229
intracellular organe...
0.005623
123 / 58793
GO:0043227
membrane−bounded org...
0.003789
122 / 57760
GO:0044444
cytoplasmic part
3.01e−07
95 / 34514
GO:0070013
intracellular organe...
0.033677
12 / 3567
GO:0044446
intracellular organe...
1.25e−05
55 / 17278
GO:0043231
intracellular membra...
0.003130
122 / 57558
GO:0043232
intracellular non−me...
0.002592
20 / 5440
GO:0005852
eukaryotic translati...
0.000587
3 / 87
GO:0005829
cytosol
3.42e−06
34 / 8016
GO:0005634
nucleus
0.100472
70 / 33379
GO:0071541
eukaryotic translati...
3.39e−05
2 / 5
GO:0044428
nuclear part
8.95e−05
19 / 3823
GO:0031981
nuclear lumen
0.020698
11 / 2929
GO:0005730
nucleolus
0.000118
11 / 1497
