# Supplementary material for: Differential Alternative Splicing Genes and Isoform Regulation Networks of Rapeseed (Brassica napus L.) Infected with Sclerotinia sclerotiorum
Source: Genes (Basel). 2020 Jul 13;11(7):784. doi: 10.3390/genes11070784 (PMC7397149; doi:10.3390/genes11070784)
Supplement: Supplementary file 1 [file genes-11-00784-s001.zip › Fig. S2 Enriched GO terms of genes in the 85 WGCNA modules.zip!/Fig. S2/CytoNodelightgreen-go.pdf]

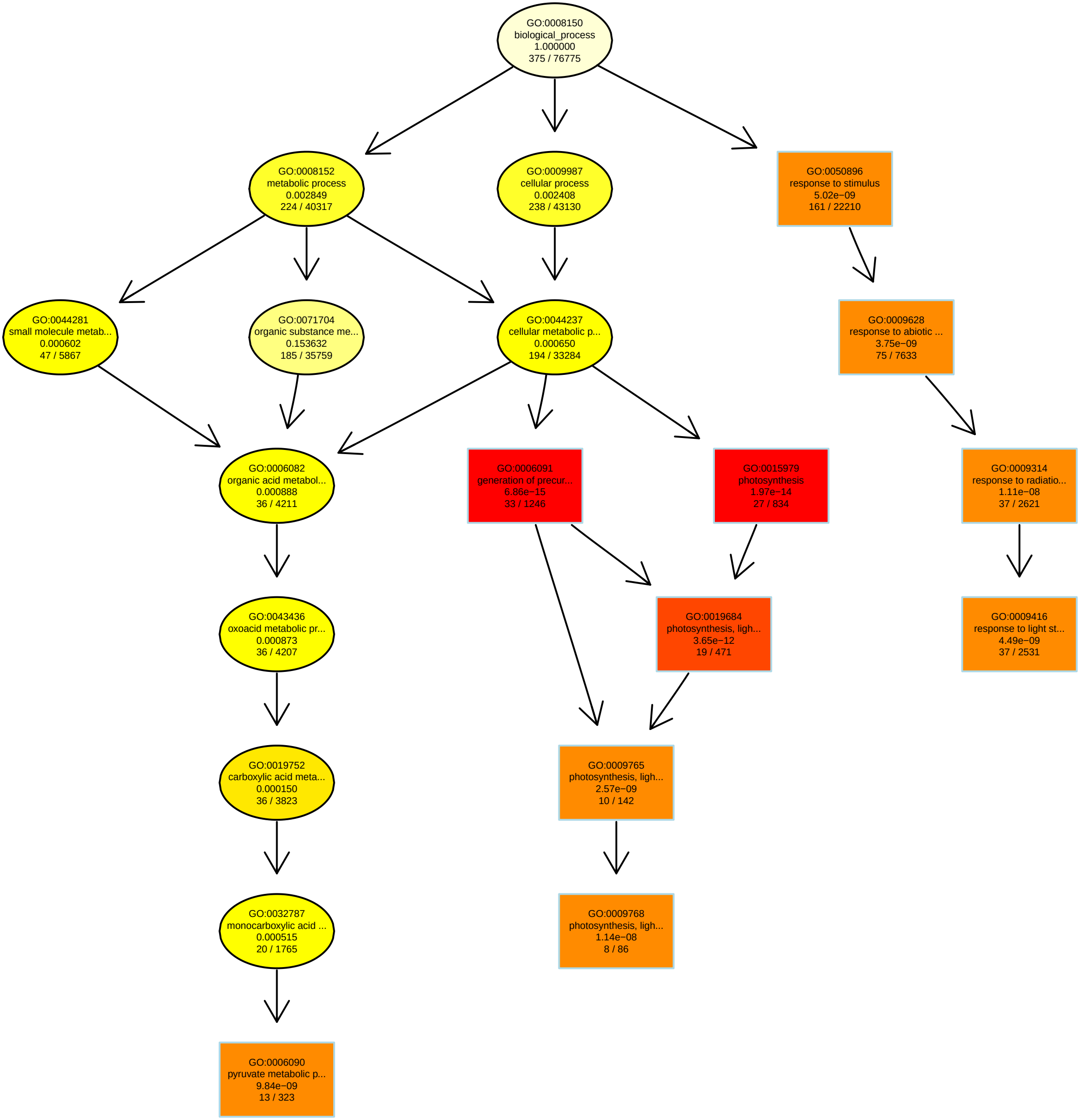
GO:0008150
biological_process
1.000000
375 / 76775
GO:0008152
metabolic process
0.002849
224 / 40317
GO:0009987
cellular process
0.002408
238 / 43130
GO:0050896
response to stimulus
5.02e−09
161 / 22210
GO:0044281
small molecule metab...
0.000602
47 / 5867
GO:0071704
organic substance me...
0.153632
185 / 35759
GO:0044237
cellular metabolic p...
0.000650
194 / 33284
GO:0009628
response to abiotic ...
3.75e−09
75 / 7633
GO:0006082
organic acid metabol...
0.000888
36 / 4211
GO:0006091
generation of precur...
6.86e−15
33 / 1246
GO:0015979
photosynthesis
1.97e−14
27 / 834
GO:0009314
response to radiatio...
1.11e−08
37 / 2621
GO:0043436
oxoacid metabolic pr...
0.000873
36 / 4207
GO:0019684
photosynthesis, ligh...
3.65e−12
19 / 471
GO:0009416
response to light st...
4.49e−09
37 / 2531
GO:0019752
carboxylic acid meta...
0.000150
36 / 3823
GO:0009765
photosynthesis, ligh...
2.57e−09
10 / 142
GO:0032787
monocarboxylic acid ...
0.000515
20 / 1765
GO:0009768
photosynthesis, ligh...
1.14e−08
8 / 86
GO:0006090
pyruvate metabolic p...
9.84e−09
13 / 323

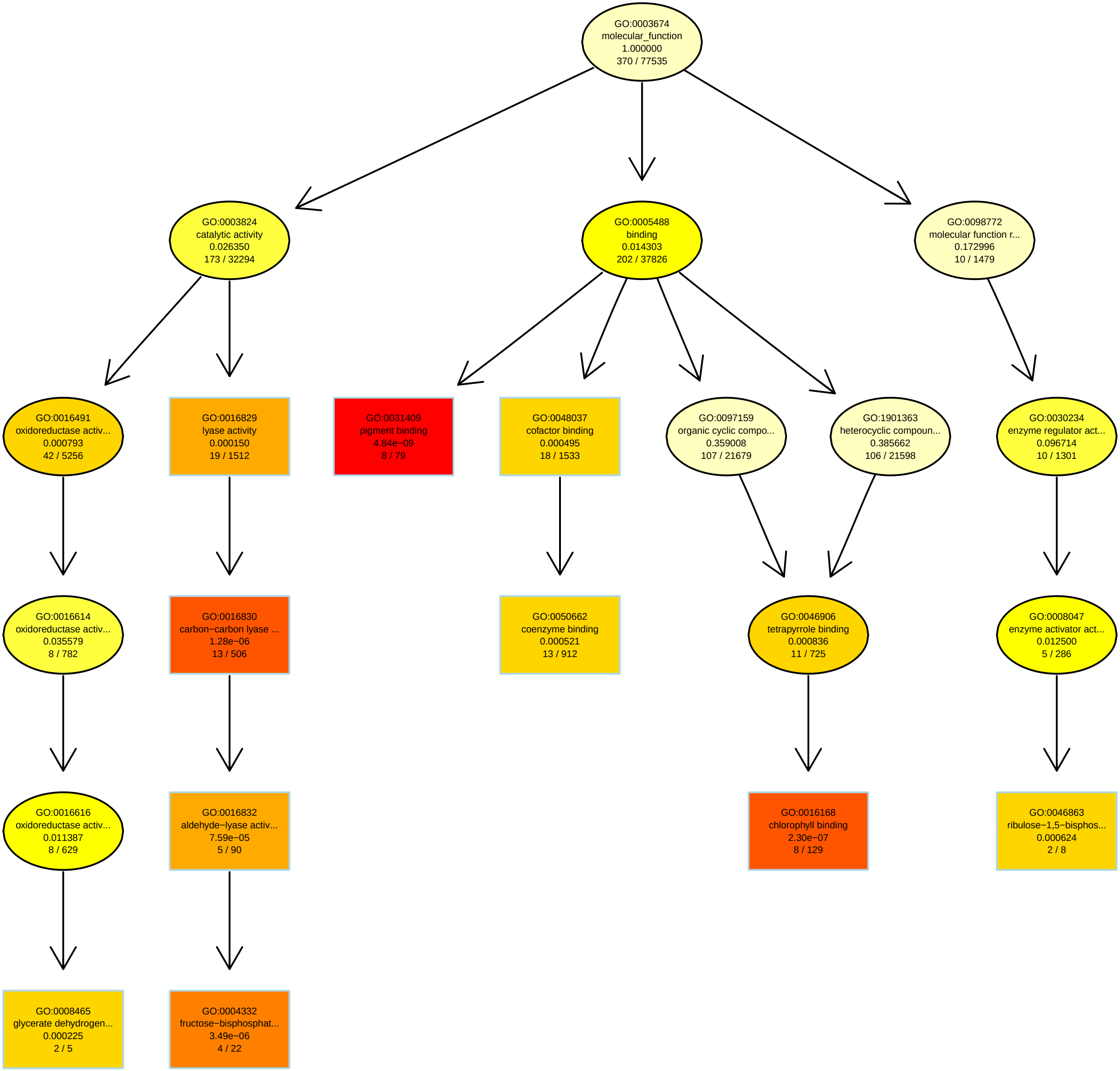
GO:0003674
molecular_function
1.000000
370 / 77535
GO:0003824
catalytic activity
0.026350
173 / 32294
GO:0005488
binding
0.014303
202 / 37826
GO:0098772
molecular function r...
0.172996
10 / 1479
GO:0016491
oxidoreductase activ...
0.000793
42 / 5256
GO:0016829
lyase activity
0.000150
19 / 1512
GO:0031409
pigment binding
4.84e−09
8 / 79
GO:0048037
cofactor binding
0.000495
18 / 1533
GO:0097159
organic cyclic compo...
0.359008
107 / 21679
GO:1901363
heterocyclic compoun...
0.385662
106 / 21598
GO:0030234
enzyme regulator act...
0.096714
10 / 1301
GO:0016614
oxidoreductase activ...
0.035579
8 / 782
GO:0016830
carbon−carbon lyase ...
1.28e−06
13 / 506
GO:0050662
coenzyme binding
0.000521
13 / 912
GO:0046906
tetrapyrrole binding
0.000836
11 / 725
GO:0008047
enzyme activator act...
0.012500
5 / 286
GO:0016616
oxidoreductase activ...
0.011387
8 / 629
GO:0016832
aldehyde−lyase activ...
7.59e−05
5 / 90
GO:0016168
chlorophyll binding
2.30e−07
8 / 129
GO:0046863
ribulose−1,5−bisphos...
0.000624
2 / 8
GO:0008465
glycerate dehydrogen...
0.000225
2 / 5
GO:0004332
fructose−bisphosphat...
3.49e−06
4 / 22

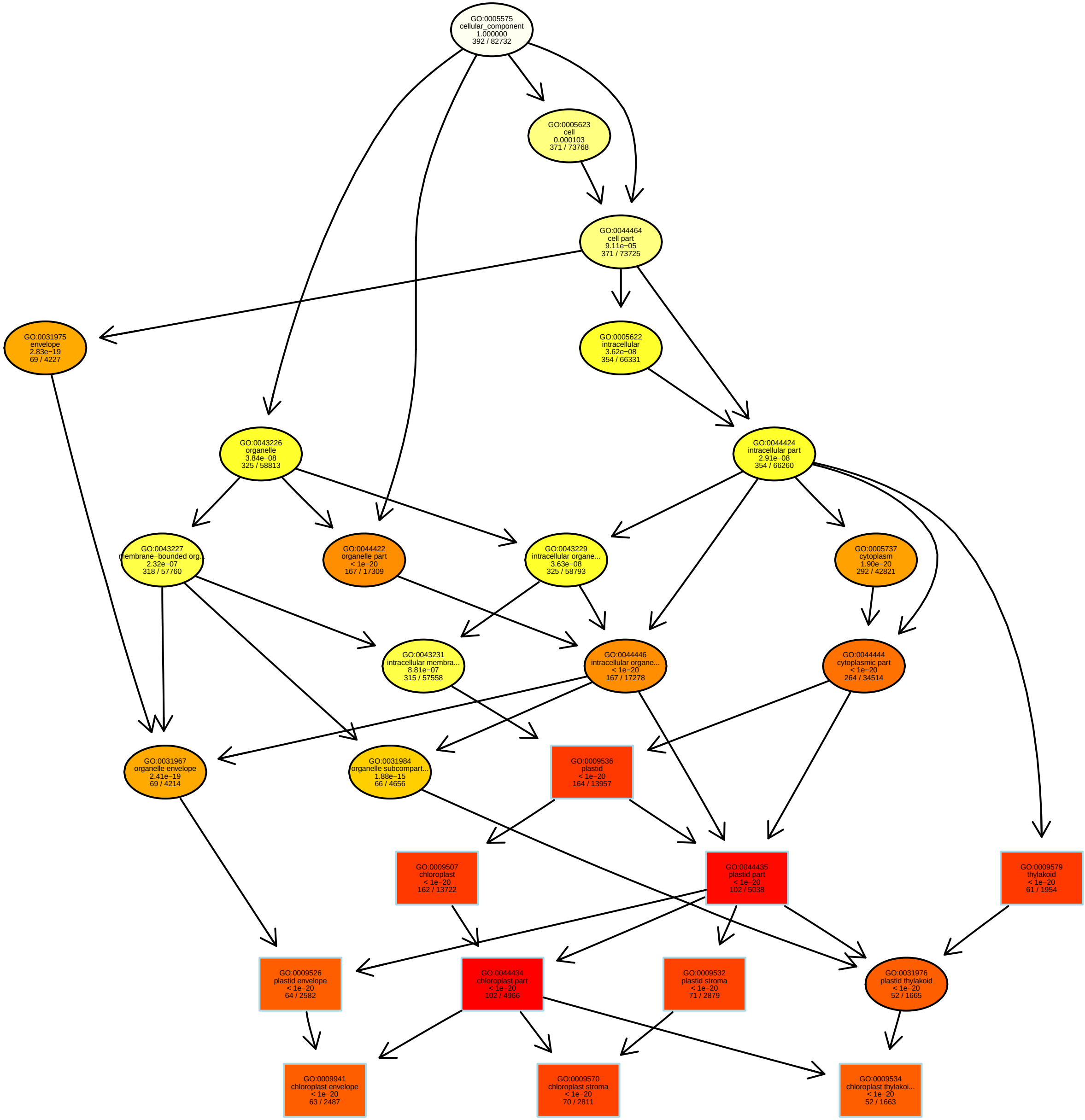
GO:0005575 cellular_component 1.000000 392 / 82732
GO:0005623 cell 0.000103 371 / 73768
GO:0044464 cell part 9.11e−05 371 / 73725
GO:0031975 envelope 2.83e−19 69 / 4227
GO:0005622 intracellular 3.62e−08 354 / 66331
GO:0043226 organelle 3.84e−08 325 / 58813
GO:0044424 intracellular part 2.91e−08 354 / 66260
GO:0043227 membrane−bounded org... 2.32e−07 318 / 57760
GO:0044422 organelle part < 1e−20 167 / 17309
GO:0043229 intracellular organe... 3.63e−08 325 / 58793
GO:0005737 cytoplasm 1.90e−20 292 / 42821
GO:0043231 intracellular membra... 8.81e−07 315 / 57558
GO:0044446 intracellular organe... < 1e−20 167 / 17278
GO:0044444 cytoplasmic part < 1e−20 264 / 34514
GO:0031967 organelle envelope 2.41e−19 69 / 4214
GO:0031984 organelle subcompart... 1.88e−15 66 / 4656
GO:0009536 plastid < 1e−20 164 / 13957
GO:0009507 chloroplast < 1e−20 162 / 13722
GO:0044435 plastid part < 1e−20 102 / 5038
GO:0009579 thylakoid < 1e−20 61 / 1954
GO:0009526 plastid envelope < 1e−20 64 / 2582
GO:0044434 chloroplast part < 1e−20 102 / 4966
GO:0009532 plastid stroma < 1e−20 71 / 2879
GO:0031976 plastid thylakoid < 1e−20 52 / 1665
GO:0009941 chloroplast envelope < 1e−20 63 / 2487
GO:0009570 chloroplast stroma < 1e−20 70 / 2811
GO:0009534 chloroplast thylakoi... < 1e−20 52 / 1663
