# Supplementary material for: Differential Alternative Splicing Genes and Isoform Regulation Networks of Rapeseed (Brassica napus L.) Infected with Sclerotinia sclerotiorum
Source: Genes (Basel). 2020 Jul 13;11(7):784. doi: 10.3390/genes11070784 (PMC7397149; doi:10.3390/genes11070784)
Supplement: Supplementary file 1 [file genes-11-00784-s001.zip › Fig. S2 Enriched GO terms of genes in the 85 WGCNA modules.zip!/Fig. S2/CytoNodelightpink3-go.pdf]

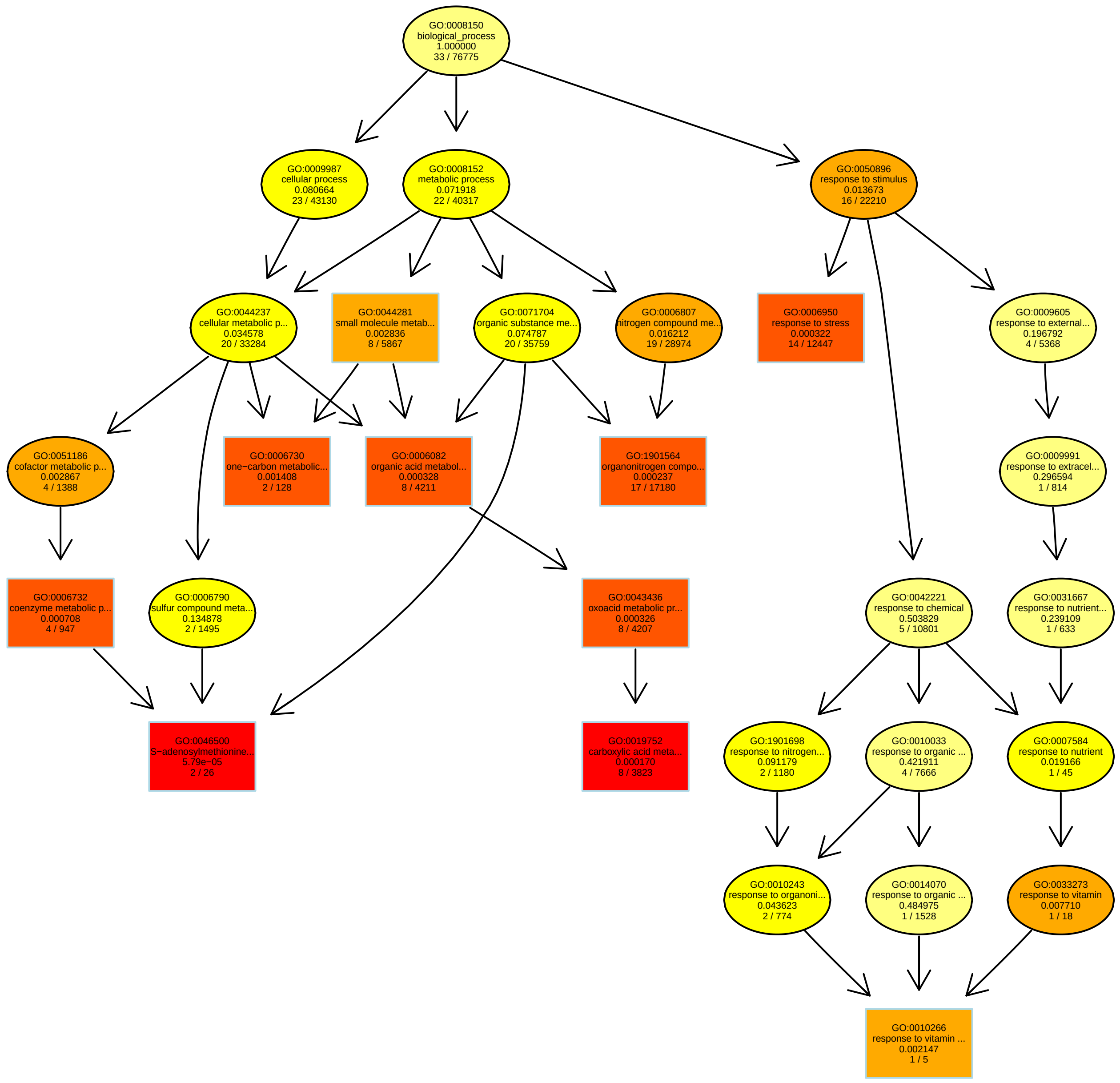

GO:0008150
biological_process
1.000000
33 / 76775
GO:0009987
cellular process
0.080664
23 / 43130
GO:0008152
metabolic process
0.071918
22 / 40317
GO:0050896
response to stimulus
0.013673
16 / 22210
GO:0044237
cellular metabolic p...
0.034578
20 / 33284
GO:0044281
small molecule metab...
0.002836
8 / 5867
GO:0071704
organic substance me...
0.074787
20 / 35759
GO:0006807
nitrogen compound me...
0.016212
19 / 28974
GO:0006950
response to stress
0.000322
14 / 12447
GO:0009605
response to external...
0.196792
4 / 5368
GO:0051186
cofactor metabolic p...
0.002867
4 / 1388
GO:0006730
one−carbon metabolic...
0.001408
2 / 128
GO:0006082
organic acid metabol...
0.000328
8 / 4211
GO:1901564
organonitrogen compo...
0.000237
17 / 17180
GO:0009991
response to extracel...
0.296594
1 / 814
GO:0006732
coenzyme metabolic p...
0.000708
4 / 947
GO:0006790
sulfur compound meta...
0.134878
2 / 1495
GO:0043436
oxoacid metabolic pr...
0.000326
8 / 4207
GO:0042221
response to chemical
0.503829
5 / 10801
GO:0031667
response to nutrient...
0.239109
1 / 633
GO:0046500
S−adenosylmethionine...
5.79e−05
2 / 26
GO:0019752
carboxylic acid meta...
0.000170
8 / 3823
GO:1901698
response to nitrogen...
0.091179
2 / 1180
GO:0010033
response to organic ...
0.421911
4 / 7666
GO:0007584
response to nutrient
0.019166
1 / 45
GO:0010243
response to organoni...
0.043623
2 / 774
GO:0014070
response to organic ...
0.484975
1 / 1528
GO:0033273
response to vitamin
0.007710
1 / 18
GO:0010266
response to vitamin ...
0.002147
1 / 5

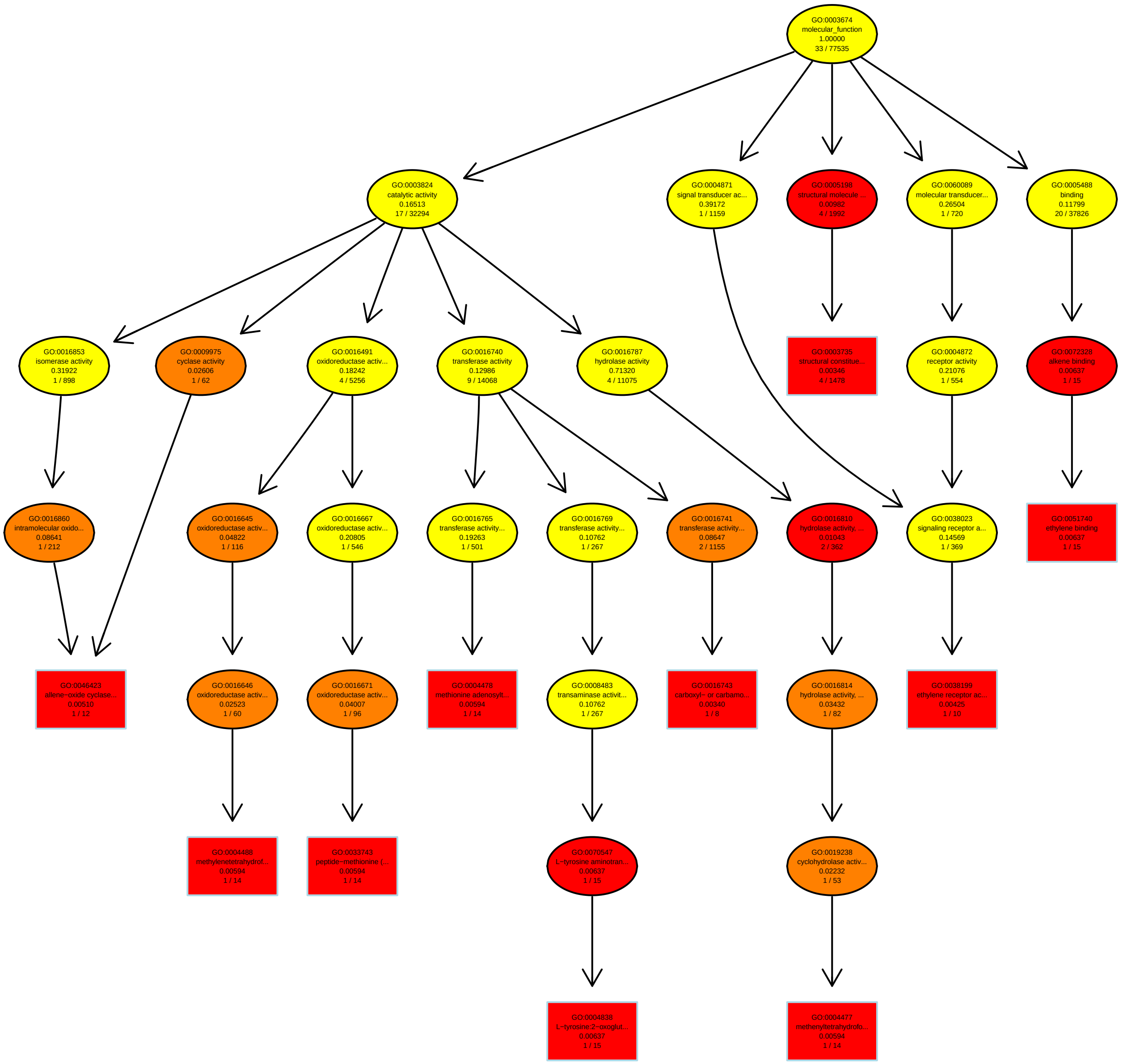

GO:0003674 molecular_function 1.00000 33 / 77535
GO:0003824 catalytic activity 0.16513 17 / 32294
GO:0004871 signal transducer ac... 0.39172 1 / 1159
GO:0005198 structural molecule ... 0.00982 4 / 1992
GO:0060089 molecular transducer... 0.26504 1 / 720
GO:0005488 binding 0.11799 20 / 37826
GO:0016853 isomerase activity 0.31922 1 / 898
GO:0009975 cyclase activity 0.02606 1 / 62
GO:0016491 oxidoreductase activ... 0.18242 4 / 5256
GO:0016740 transferase activity 0.12986 9 / 14068
GO:0016787 hydrolase activity 0.71320 4 / 11075
GO:0003735 structural constitue... 0.00346 4 / 1478
GO:0004872 receptor activity 0.21076 1 / 554
GO:0072328 alkene binding 0.00637 1 / 15
GO:0016860 intramolecular oxido... 0.08641 1 / 212
GO:0016645 oxidoreductase activ... 0.04822 1 / 116
GO:0016667 oxidoreductase activ... 0.20805 1 / 546
GO:0016765 transferase activity... 0.19263 1 / 501
GO:0016769 transferase activity... 0.10762 1 / 267
GO:0016741 transferase activity... 0.08647 2 / 1155
GO:0016810 hydrolase activity, ... 0.01043 2 / 362
GO:0038023 signaling receptor a... 0.14569 1 / 369
GO:0051740 ethylene binding 0.00637 1 / 15
GO:0046423 allene−oxide cyclase... 0.00510 1 / 12
GO:0016646 oxidoreductase activ... 0.02523 1 / 60
GO:0016671 oxidoreductase activ... 0.04007 1 / 96
GO:0004478 methionine adenosylt... 0.00594 1 / 14
GO:0008483 transaminase activit... 0.10762 1 / 267
GO:0016743 carboxyl− or carbamo... 0.00340 1 / 8
GO:0016814 hydrolase activity, ... 0.03432 1 / 82
GO:0038199 ethylene receptor ac... 0.00425 1 / 10
GO:0004488 methylenetetrahydrof... 0.00594 1 / 14
GO:0033743 peptide−methionine (... 0.00594 1 / 14
GO:0070547 L−tyrosine aminotran... 0.00637 1 / 15
GO:0019238 cyclohydrolase activ... 0.02232 1 / 53
GO:0004838 L−tyrosine:2−oxoglut... 0.00637 1 / 15
GO:0004477 methenyltetrahydrofo... 0.00594 1 / 14

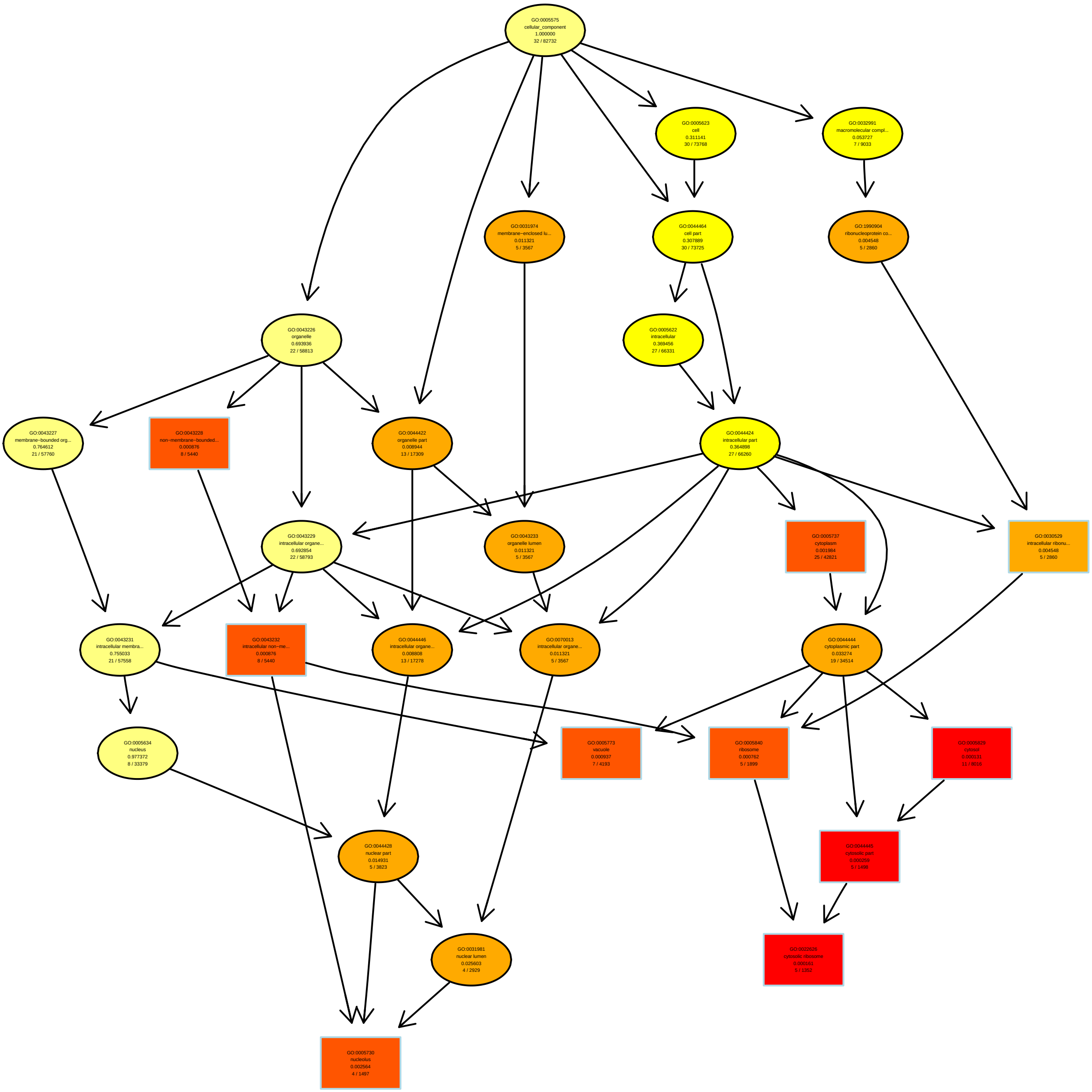
GO:0005575
cellular_component
1.000000
32 / 82732
GO:0005623
cell
0.311141
30 / 73768
GO:0032991
macromolecular compl...
0.053727
7 / 9033
GO:0031974
membrane−enclosed lu...
0.011321
5 / 3567
GO:0044464
cell part
0.307889
30 / 73725
GO:1990904
ribonucleoprotein co...
0.004548
5 / 2860
GO:0043226
organelle
0.693936
22 / 58813
GO:0005622
intracellular
0.369456
27 / 66331
GO:0043227
membrane−bounded org...
0.764612
21 / 57760
GO:0043228
non−membrane−bounded...
0.000876
8 / 5440
GO:0044422
organelle part
0.008944
13 / 17309
GO:0044424
intracellular part
0.364898
27 / 66260
GO:0043229
intracellular organe...
0.692854
22 / 58793
GO:0043233
organelle lumen
0.011321
5 / 3567
GO:0005737
cytoplasm
0.001984
25 / 42821
GO:0030529
intracellular ribonu...
0.004548
5 / 2860
GO:0043231
intracellular membra...
0.755033
21 / 57558
GO:0043232
intracellular non−me...
0.000876
8 / 5440
GO:0044446
intracellular organe...
0.008808
13 / 17278
GO:0070013
intracellular organe...
0.011321
5 / 3567
GO:0044444
cytoplasmic part
0.033274
19 / 34514
GO:0005634
nucleus
0.977372
8 / 33379
GO:0005773
vacuole
0.000937
7 / 4193
GO:0005840
ribosome
0.000762
5 / 1899
GO:0005829
cytosol
0.000131
11 / 8016
GO:0044428
nuclear part
0.014931
5 / 3823
GO:0044445
cytosolic part
0.000259
5 / 1498
GO:0031981
nuclear lumen
0.025603
4 / 2929
GO:0022626
cytosolic ribosome
0.000161
5 / 1352
GO:0005730
nucleolus
0.002564
4 / 1497
