# Supplementary material for: Differential Alternative Splicing Genes and Isoform Regulation Networks of Rapeseed (Brassica napus L.) Infected with Sclerotinia sclerotiorum
Source: Genes (Basel). 2020 Jul 13;11(7):784. doi: 10.3390/genes11070784 (PMC7397149; doi:10.3390/genes11070784)
Supplement: Supplementary file 1 [file genes-11-00784-s001.zip › Fig. S2 Enriched GO terms of genes in the 85 WGCNA modules.zip!/Fig. S2/CytoNodelightpink4-go.pdf]

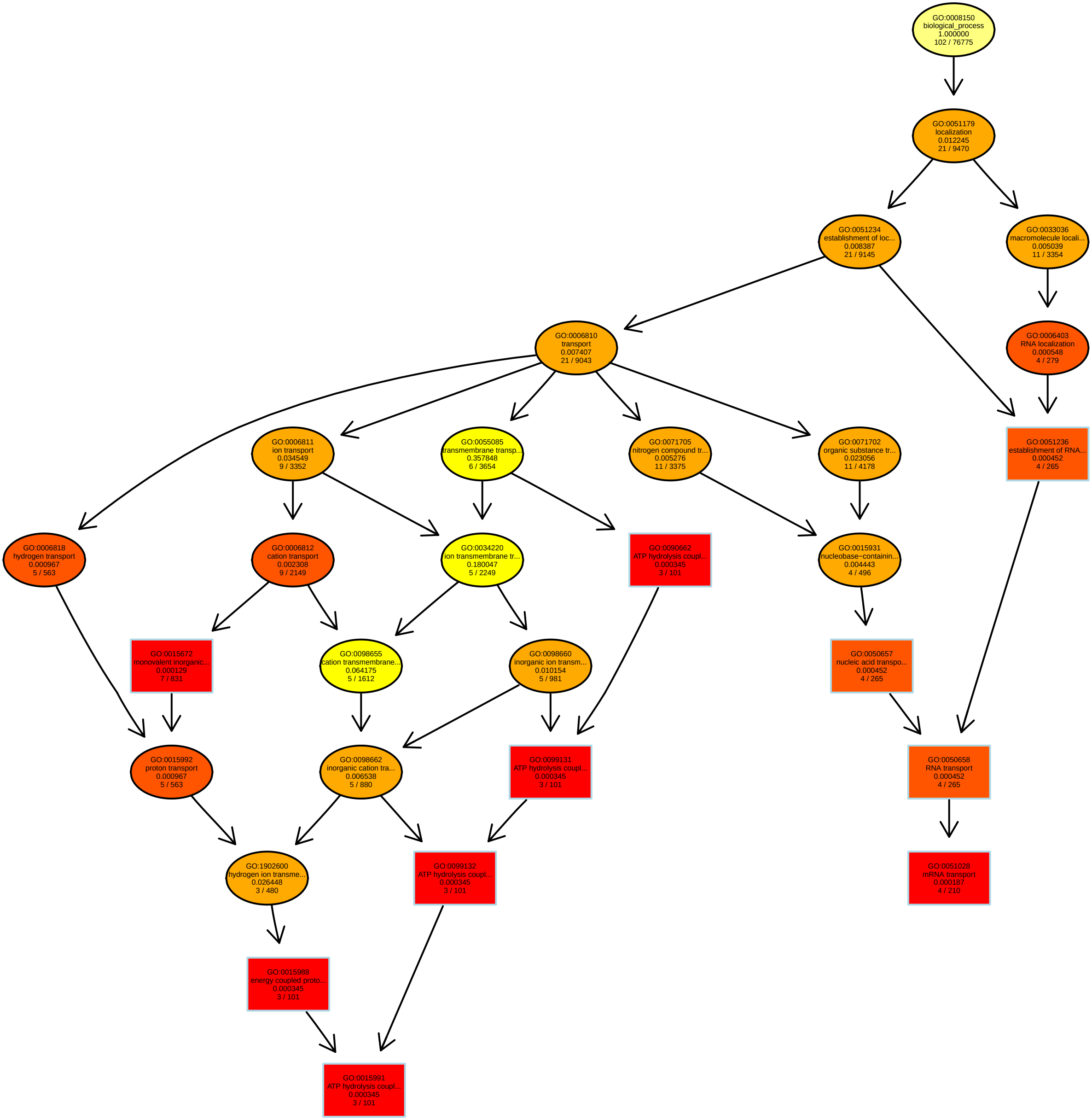
GO:0008150 biological_process 1.000000 102 / 76775
GO:0051179 localization 0.012245 21 / 9470
GO:0051234 establishment of loc... 0.008387 21 / 9145
GO:0033036 macromolecule locali... 0.005039 11 / 3354
GO:0006810 transport 0.007407 21 / 9043
GO:0006403 RNA localization 0.000548 4 / 279
GO:0006811 ion transport 0.034549 9 / 3352
GO:0055085 transmembrane transp... 0.357848 6 / 3654
GO:0071705 nitrogen compound tr... 0.005276 11 / 3375
GO:0071702 organic substance tr... 0.023056 11 / 4178
GO:0051236 establishment of RNA... 0.000452 4 / 265
GO:0006818 hydrogen transport 0.000967 5 / 563
GO:0006812 cation transport 0.002308 9 / 2149
GO:0034220 ion transmembrane tr... 0.180047 5 / 2249
GO:0090662 ATP hydrolysis coupl... 0.000345 3 / 101
GO:0015931 nucleobase−containin... 0.004443 4 / 496
GO:0015672 monovalent inorganic... 0.000129 7 / 831
GO:0098655 cation transmembrane... 0.064175 5 / 1612
GO:0098660 inorganic ion transm... 0.010154 5 / 981
GO:0050657 nucleic acid transpo... 0.000452 4 / 265
GO:0015992 proton transport 0.000967 5 / 563
GO:0098662 inorganic cation tra... 0.006538 5 / 880
GO:0099131 ATP hydrolysis coupl... 0.000345 3 / 101
GO:0050658 RNA transport 0.000452 4 / 265
GO:1902600 hydrogen ion transme... 0.026448 3 / 480
GO:0099132 ATP hydrolysis coupl... 0.000345 3 / 101
GO:0051028 mRNA transport 0.000187 4 / 210
GO:0015988 energy coupled proto... 0.000345 3 / 101
GO:0015991 ATP hydrolysis coupl... 0.000345 3 / 101

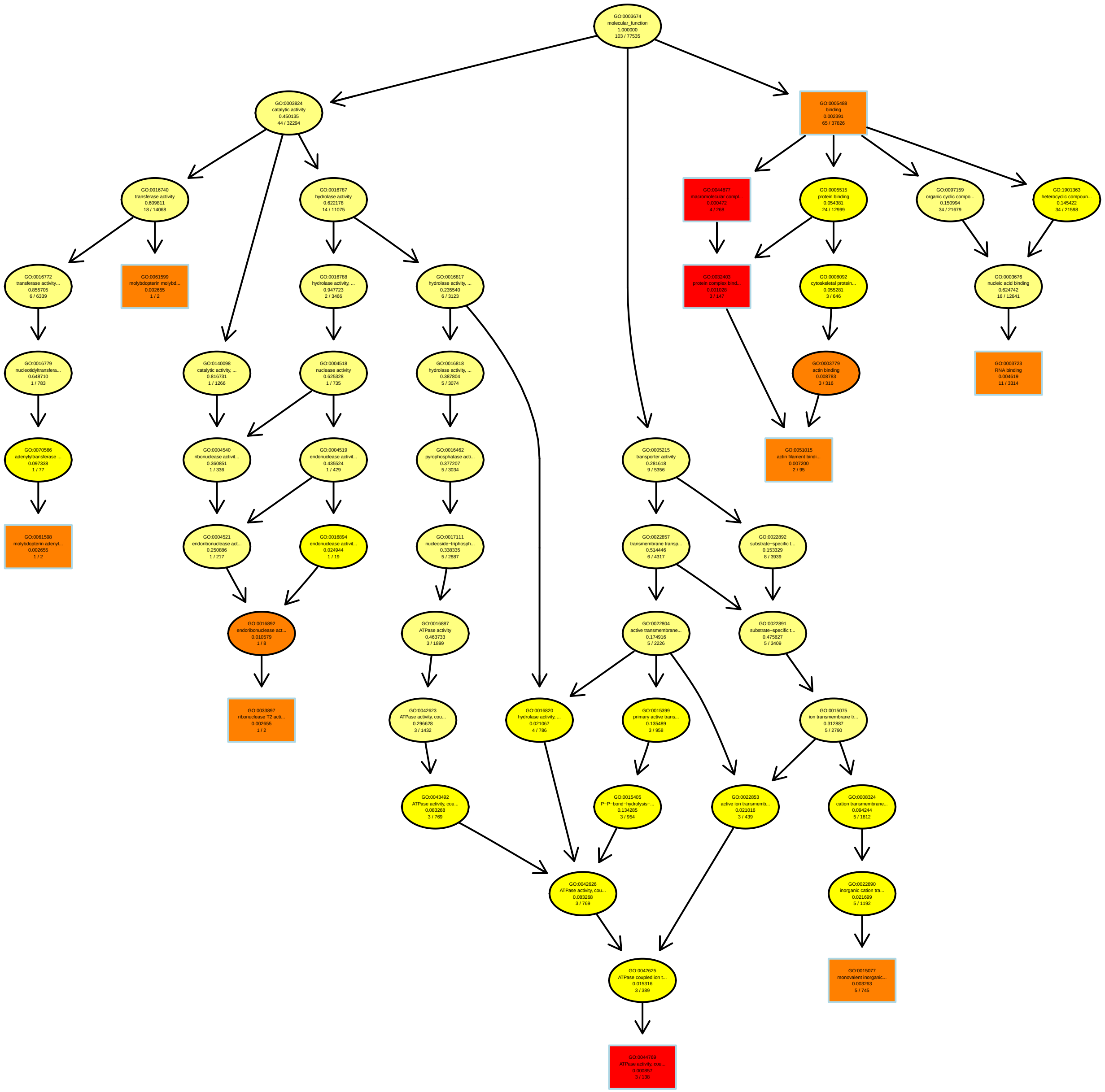
GO:0003674 molecular_function 1.000000 103 / 77535
GO:0003824 catalytic activity 0.450135 44 / 32294
GO:0005488 binding 0.002391 65 / 37826
GO:0016740 transferase activity 0.609811 18 / 14068
GO:0016787 hydrolase activity 0.622178 14 / 11075
GO:0044877 macromolecular compl... 0.000472 4 / 268
GO:0005515 protein binding 0.054381 24 / 12999
GO:0097159 organic cyclic compo... 0.150994 34 / 21679
GO:1901363 heterocyclic compoun... 0.145422 34 / 21598
GO:0016772 transferase activity... 0.855705 6 / 6339
GO:0061599 molybdopterin molybd... 0.002655 1 / 2
GO:0016788 hydrolase activity, ... 0.947723 2 / 3466
GO:0016817 hydrolase activity, ... 0.235540 6 / 3123
GO:0032403 protein complex bind... 0.001029 3 / 147
GO:0008092 cytoskeletal protein... 0.055281 3 / 646
GO:0003676 nucleic acid binding 0.624742 16 / 12641
GO:0016779 nucleotidyltransfera... 0.648710 1 / 783
GO:0140098 catalytic activity, ... 0.816731 1 / 1266
GO:0004518 nuclease activity 0.625328 1 / 735
GO:0016818 hydrolase activity, ... 0.387804 5 / 3074
GO:0003779 actin binding 0.008783 3 / 316
GO:0003723 RNA binding 0.004619 11 / 3314
GO:0070566 adenylyltransferase ... 0.097338 1 / 77
GO:0004540 ribonuclease activit... 0.360851 1 / 336
GO:0004519 endonuclease activit... 0.435524 1 / 429
GO:0016462 pyrophosphatase acti... 0.377207 5 / 3034
GO:0005215 transporter activity 0.281618 9 / 5356
GO:0051015 actin filament bindi... 0.007200 2 / 95
GO:0061598 molybdopterin adenyl... 0.002655 1 / 2
GO:0004521 endoribonuclease act... 0.250886 1 / 217
GO:0016894 endonuclease activit... 0.024944 1 / 19
GO:0017111 nucleoside-triphosph... 0.338335 5 / 2887
GO:0022857 transmembrane transp... 0.514446 6 / 4317
GO:0022892 substrate-specific t... 0.153329 8 / 3939
GO:0016892 endoribonuclease act... 0.010579 1 / 8
GO:0016887 ATPase activity 0.463733 3 / 1899
GO:0022804 active transmembrane... 0.174916 5 / 2226
GO:0022891 substrate-specific t... 0.475627 5 / 3409
GO:0033897 ribonuclease T2 acti... 0.002655 1 / 2
GO:0042623 ATPase activity, cou... 0.296628 3 / 1432
GO:0016820 hydrolase activity, ... 0.021067 4 / 786
GO:0015399 primary active trans... 0.135489 3 / 958
GO:0015075 ion transmembrane tr... 0.312887 5 / 2790
GO:0043492 ATPase activity, cou... 0.083268 3 / 769
GO:0015405 P-P-bond-hydrolysis-... 0.134285 3 / 954
GO:0022853 active ion transmemb... 0.021016 3 / 439
GO:0008324 cation transmembrane... 0.094244 5 / 1812
GO:0042626 ATPase activity, cou... 0.083268 3 / 769
GO:0022890 inorganic cation tra... 0.021699 5 / 1192
GO:0042625 ATPase coupled ion t... 0.015316 3 / 389
GO:0015077 monovalent inorganic... 0.003263 5 / 745
GO:0044769 ATPase activity, cou... 0.000057 3 / 136

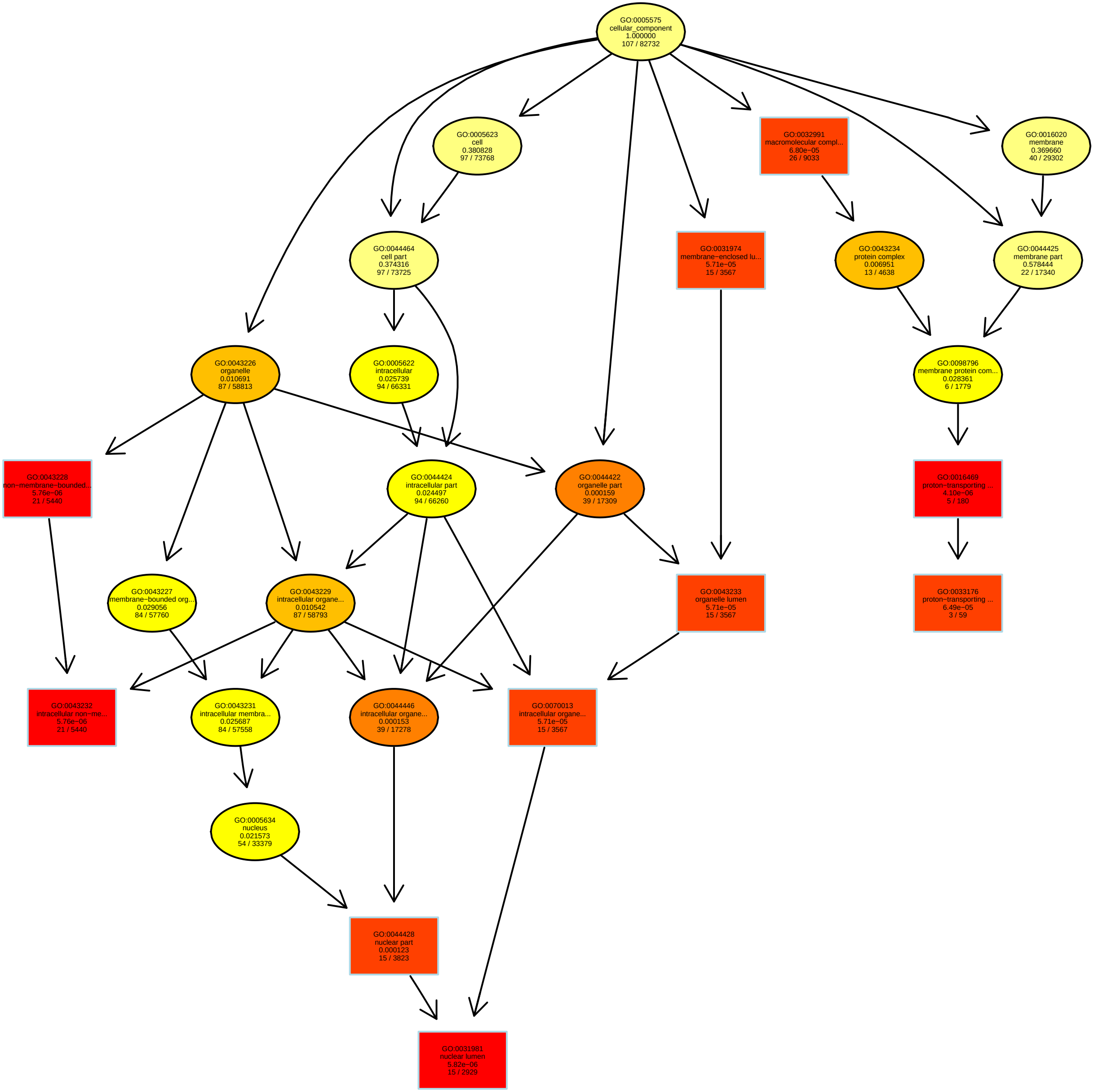
GO:0005575
cellular_component
1.000000
107 / 82732
GO:0005623
cell
0.380828
97 / 73768
GO:0032991
macromolecular compl...
6.80e−05
26 / 9033
GO:0016020
membrane
0.369660
40 / 29302
GO:0044464
cell part
0.374316
97 / 73725
GO:0031974
membrane−enclosed lu...
5.71e−05
15 / 3567
GO:0043234
protein complex
0.006951
13 / 4638
GO:0044425
membrane part
0.578444
22 / 17340
GO:0043226
organelle
0.010691
87 / 58813
GO:0005622
intracellular
0.025739
94 / 66331
GO:0098796
membrane protein com...
0.028361
6 / 1779
GO:0043228
non−membrane−bounded...
5.76e−06
21 / 5440
GO:0044424
intracellular part
0.024497
94 / 66260
GO:0044422
organelle part
0.000159
39 / 17309
GO:0016469
proton−transporting ...
4.10e−06
5 / 160
GO:0043227
membrane−bounded org...
0.029056
84 / 57760
GO:0043229
intracellular organe...
0.010542
87 / 58793
GO:0043233
organelle lumen
5.71e−05
15 / 3567
GO:0033176
proton−transporting ...
6.49e−05
3 / 59
GO:0043232
intracellular non−me...
5.76e−06
21 / 5440
GO:0043231
intracellular membra...
0.025687
84 / 57558
GO:0044446
intracellular organe...
0.000153
39 / 17278
GO:0070013
intracellular organe...
5.71e−05
15 / 3567
GO:0005634
nucleus
0.021573
54 / 33379
GO:0044428
nuclear part
0.000123
15 / 3823
GO:0031981
nuclear lumen
5.82e−06
15 / 2929
