# Supplementary material for: Differential Alternative Splicing Genes and Isoform Regulation Networks of Rapeseed (Brassica napus L.) Infected with Sclerotinia sclerotiorum
Source: Genes (Basel). 2020 Jul 13;11(7):784. doi: 10.3390/genes11070784 (PMC7397149; doi:10.3390/genes11070784)
Supplement: Supplementary file 1 [file genes-11-00784-s001.zip › Fig. S2 Enriched GO terms of genes in the 85 WGCNA modules.zip!/Fig. S2/CytoNodelightsteelblue-go.pdf]

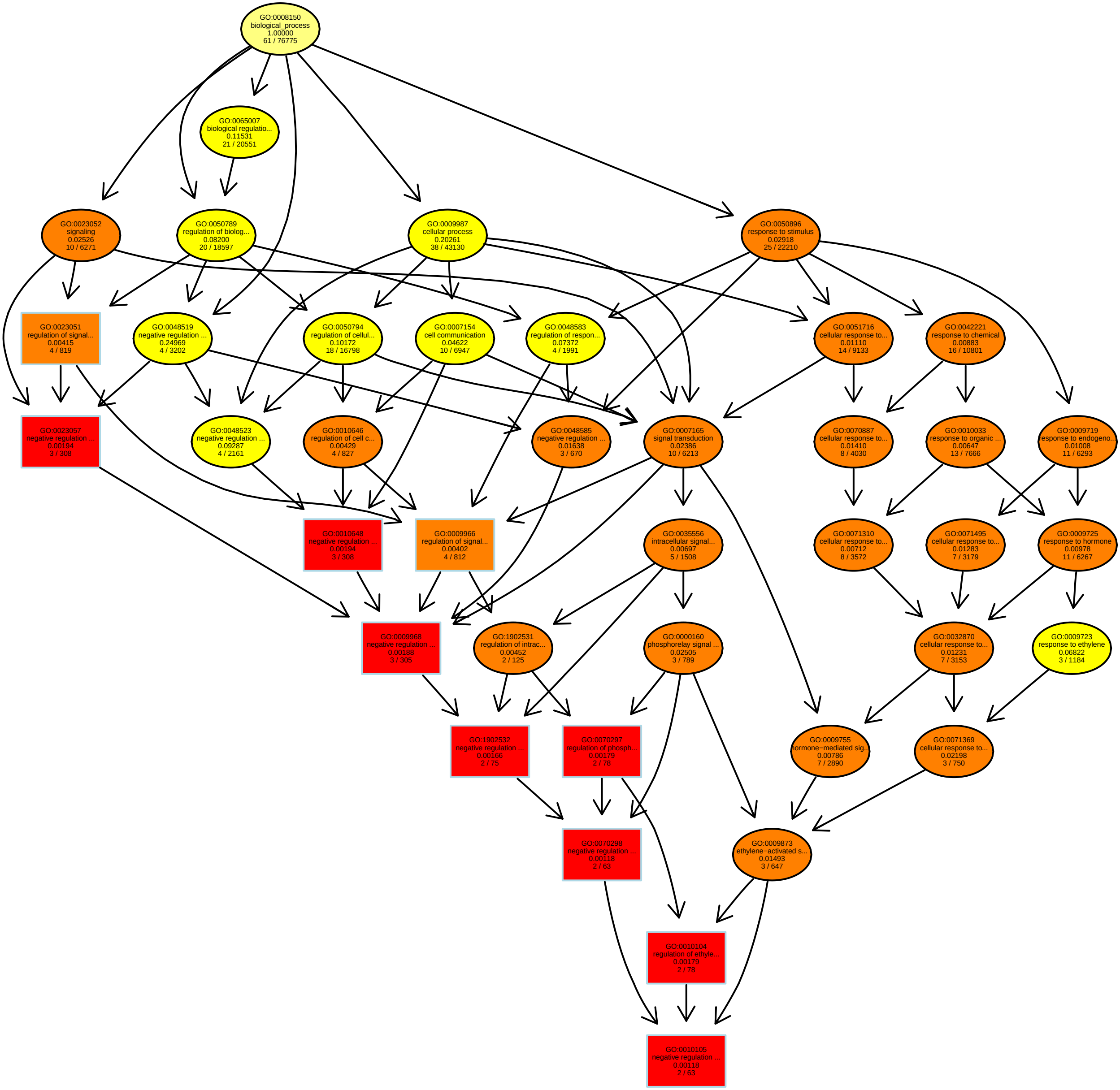
GO:0008150 biological_process 1.00000 61 / 76775
GO:0065007 biological regulatio... 0.11531 21 / 20551
GO:0023052 signaling 0.02526 10 / 6271
GO:0050789 regulation of biolog... 0.08200 20 / 18597
GO:0009987 cellular process 0.20261 38 / 43130
GO:0050896 response to stimulus 0.02918 25 / 22210
GO:0023051 regulation of signal... 0.00415 4 / 819
GO:0048519 negative regulation ... 0.24969 4 / 3202
GO:0050794 regulation of cellul... 0.10172 18 / 16798
GO:0007154 cell communication 0.04622 10 / 6947
GO:0048583 regulation of respon... 0.07372 4 / 1991
GO:0051716 cellular response to... 0.01110 14 / 9133
GO:0042221 response to chemical 0.00883 16 / 10801
GO:0023057 negative regulation ... 0.00194 3 / 308
GO:0048523 negative regulation ... 0.09287 4 / 2161
GO:0010646 regulation of cell c... 0.00429 4 / 827
GO:0048585 negative regulation ... 0.01638 3 / 670
GO:0007165 signal transduction 0.02386 10 / 6213
GO:0070887 cellular response to... 0.01410 8 / 4030
GO:0010033 response to organic ... 0.00647 13 / 7666
GO:0009719 response to endogeno... 0.01008 11 / 6293
GO:0010648 negative regulation ... 0.00194 3 / 308
GO:0009966 regulation of signal... 0.00402 4 / 812
GO:0035556 intracellular signal... 0.00697 5 / 1508
GO:0071310 cellular response to... 0.00712 8 / 3572
GO:0071495 cellular response to... 0.01283 7 / 3179
GO:0009725 response to hormone 0.00978 11 / 6267
GO:0009968 negative regulation ... 0.00188 3 / 305
GO:1902531 regulation of intrac... 0.00452 2 / 125
GO:0000160 phosphorelay signal ... 0.02505 3 / 789
GO:0032870 cellular response to... 0.01231 7 / 3153
GO:0009723 response to ethylene 0.06822 3 / 1184
GO:1902532 negative regulation ... 0.00166 2 / 75
GO:0070297 regulation of phosph... 0.00179 2 / 78
GO:0009755 hormone−mediated sig... 0.00786 7 / 2890
GO:0071369 cellular response to... 0.02198 3 / 750
GO:0070298 negative regulation ... 0.00118 2 / 63
GO:0009873 ethylene−activated s... 0.01493 3 / 647
GO:0010104 regulation of ethyle... 0.00179 2 / 78
GO:0010105 negative regulation ... 0.00118 2 / 63

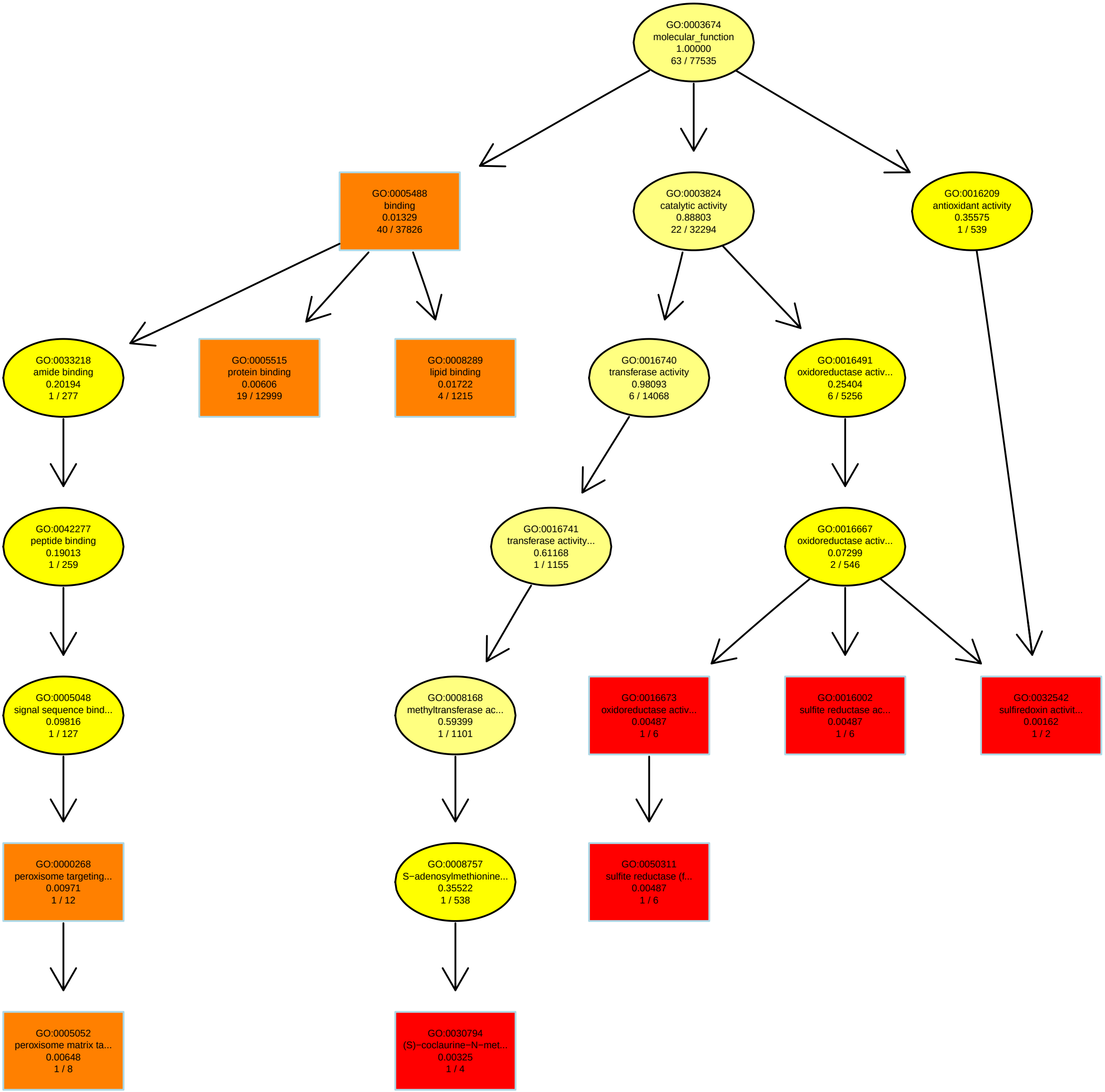
GO:0003674
molecular_function
1.00000
63 / 77535
GO:0005488
binding
0.01329
40 / 37826
GO:0003824
catalytic activity
0.88803
22 / 32294
GO:0016209
antioxidant activity
0.35575
1 / 539
GO:0033218
amide binding
0.20194
1 / 277
GO:0005515
protein binding
0.00606
19 / 12999
GO:0008289
lipid binding
0.01722
4 / 1215
GO:0016740
transferase activity
0.98093
6 / 14068
GO:0016491
oxidoreductase activ...
0.25404
6 / 5256
GO:0042277
peptide binding
0.19013
1 / 259
GO:0016741
transferase activity...
0.61168
1 / 1155
GO:0016667
oxidoreductase activ...
0.07299
2 / 546
GO:0005048
signal sequence bind...
0.09816
1 / 127
GO:0008168
methyltransferase ac...
0.59399
1 / 1101
GO:0016673
oxidoreductase activ...
0.00487
1 / 6
GO:0016002
sulfite reductase ac...
0.00487
1 / 6
GO:0032542
sulfiredoxin activit...
0.00162
1 / 2
GO:0000268
peroxisome targeting...
0.00971
1 / 12
GO:0008757
S−adenosylmethionine...
0.35522
1 / 538
GO:0050311
sulfite reductase (f...
0.00487
1 / 6
GO:0005052
peroxisome matrix ta...
0.00648
1 / 8
GO:0030794
(S)−coclaurine−N−met...
0.00325
1 / 4

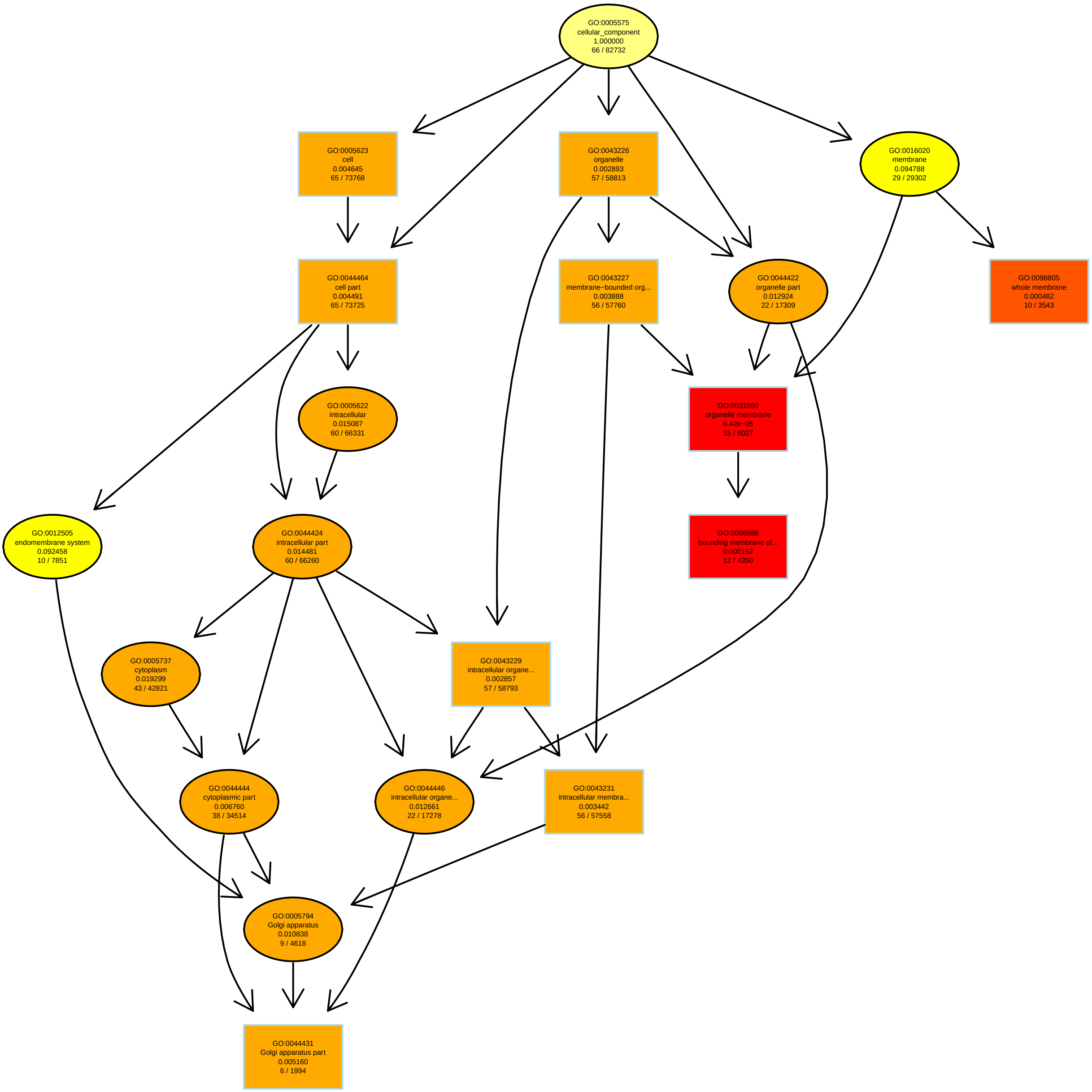
GO:0005575
cellular_component
1.000000
66 / 82732
GO:0005623
cell
0.004645
65 / 73768
GO:0043226
organelle
0.002893
57 / 58813
GO:0016020
membrane
0.094788
29 / 29302
GO:0044464
cell part
0.004491
65 / 73725
GO:0043227
membrane−bounded org...
0.003888
56 / 57760
GO:0044422
organelle part
0.012924
22 / 17309
GO:0098805
whole membrane
0.000482
10 / 3543
GO:0005622
intracellular
0.015087
60 / 66331
GO:0031090
organelle membrane
6.42e−05
15 / 6027
GO:0012505
endomembrane system
0.092458
10 / 7851
GO:0044424
intracellular part
0.014481
60 / 66260
GO:0098588
bounding membrane of...
0.000152
12 / 4350
GO:0005737
cytoplasm
0.019299
43 / 42821
GO:0043229
intracellular organe...
0.002857
57 / 58793
GO:0044444
cytoplasmic part
0.006760
38 / 34514
GO:0044446
intracellular organe...
0.012661
22 / 17278
GO:0043231
intracellular membra...
0.003442
56 / 57558
GO:0005794
Golgi apparatus
0.010838
9 / 4618
GO:0044431
Golgi apparatus part
0.005160
6 / 1994
