# Supplementary material for: Differential Alternative Splicing Genes and Isoform Regulation Networks of Rapeseed (Brassica napus L.) Infected with Sclerotinia sclerotiorum
Source: Genes (Basel). 2020 Jul 13;11(7):784. doi: 10.3390/genes11070784 (PMC7397149; doi:10.3390/genes11070784)
Supplement: Supplementary file 1 [file genes-11-00784-s001.zip › Fig. S2 Enriched GO terms of genes in the 85 WGCNA modules.zip!/Fig. S2/CytoNodelightsteelblue1-go.pdf]

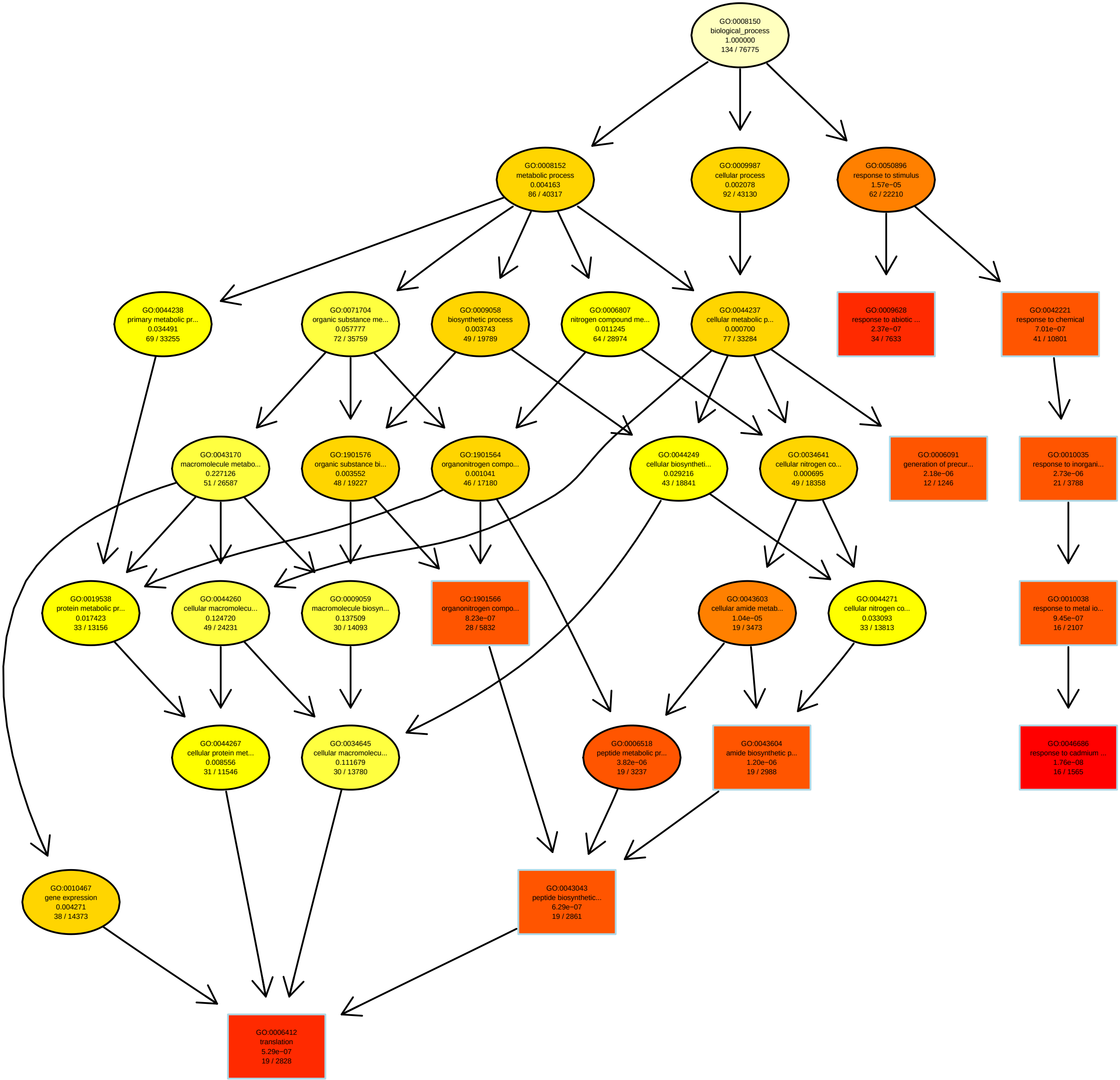
GO:0008150
biological_process
1.000000
134 / 76775
GO:0008152
metabolic process
0.004163
86 / 40317
GO:0009987
cellular process
0.002078
92 / 43130
GO:0050896
response to stimulus
1.57e−05
62 / 22210
GO:0044238
primary metabolic pr...
0.034491
69 / 33255
GO:0071704
organic substance me...
0.057777
72 / 35759
GO:0009058
biosynthetic process
0.003743
49 / 19789
GO:0006807
nitrogen compound me...
0.011245
64 / 28974
GO:0044237
cellular metabolic p...
0.000700
77 / 33284
GO:0009628
response to abiotic ...
2.37e−07
34 / 7633
GO:0042221
response to chemical
7.01e−07
41 / 10801
GO:0043170
macromolecule metabo...
0.227126
51 / 26587
GO:1901576
organic substance bi...
0.003552
48 / 19227
GO:1901564
organonitrogen compo...
0.001041
46 / 17180
GO:0044249
cellular biosyntheti...
0.029216
43 / 18841
GO:0034641
cellular nitrogen co...
0.000695
49 / 18358
GO:0006091
generation of precur...
2.18e−06
12 / 1246
GO:0010035
response to inorgani...
2.73e−06
21 / 3788
GO:0019538
protein metabolic pr...
0.017423
33 / 13156
GO:0044260
cellular macromolecu...
0.124720
49 / 24231
GO:0009059
macromolecule biosyn...
0.137509
30 / 14093
GO:1901566
organonitrogen compo...
8.23e−07
28 / 5832
GO:0043603
cellular amide metab...
1.04e−05
19 / 3473
GO:0044271
cellular nitrogen co...
0.033093
33 / 13813
GO:0010038
response to metal io...
9.45e−07
16 / 2107
GO:0044267
cellular protein met...
0.008556
31 / 11546
GO:0034645
cellular macromolecu...
0.111679
30 / 13780
GO:0006518
peptide metabolic pr...
3.82e−06
19 / 3237
GO:0043604
amide biosynthetic p...
1.20e−06
19 / 2988
GO:0046686
response to cadmium ...
1.76e−08
16 / 1565
GO:0010467
gene expression
0.004271
38 / 14373
GO:0043043
peptide biosynthetic...
6.29e−07
19 / 2861
GO:0006412
translation
5.29e−07
19 / 2828

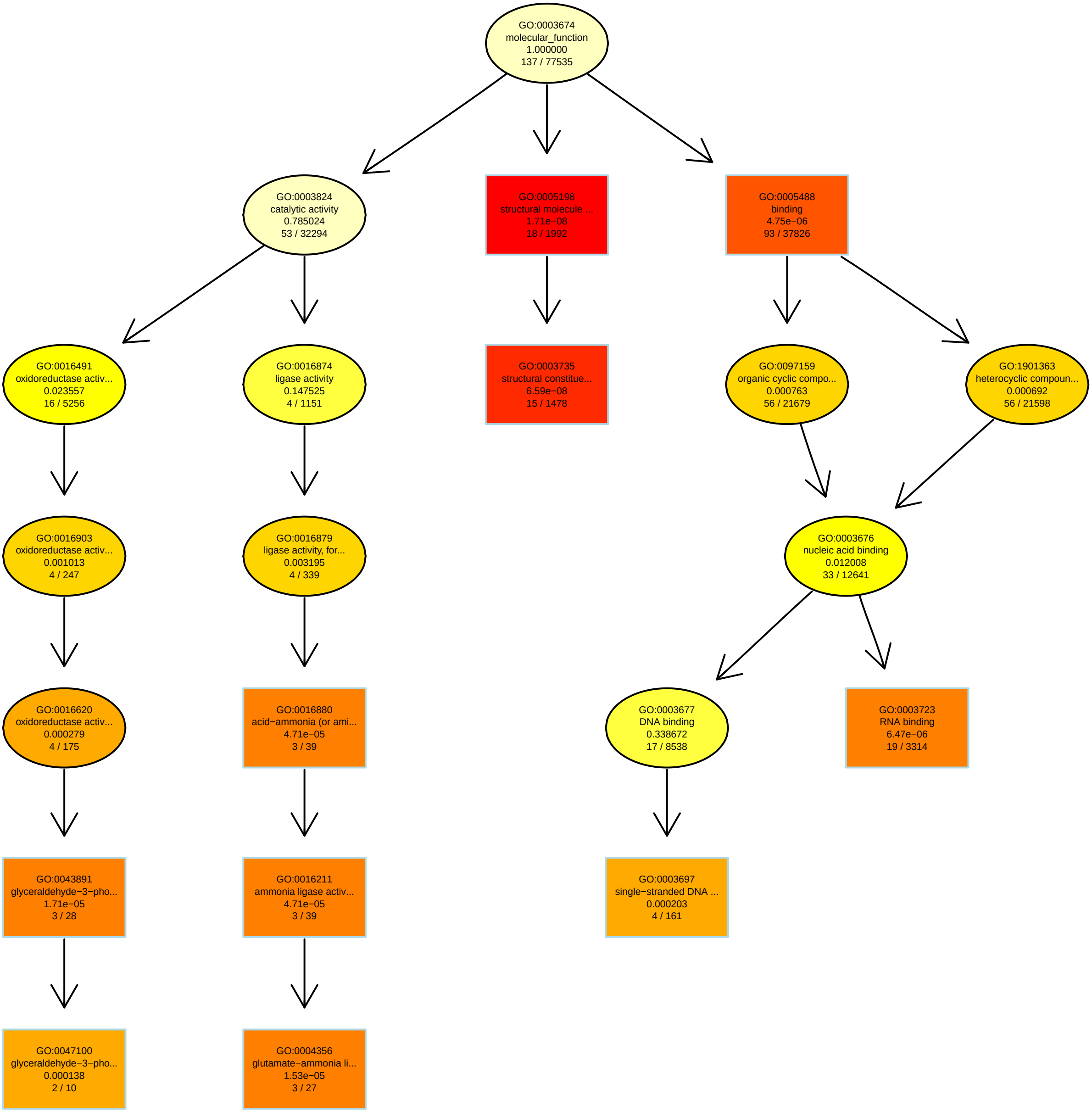
GO:0003674
molecular_function
1.000000
137 / 77535
GO:0003824
catalytic activity
0.785024
53 / 32294
GO:0005198
structural molecule ...
1.71e−08
18 / 1992
GO:0005488
binding
4.75e−06
93 / 37826
GO:0016491
oxidoreductase activ...
0.023557
16 / 5256
GO:0016874
ligase activity
0.147525
4 / 1151
GO:0003735
structural constitue...
6.59e−08
15 / 1478
GO:0097159
organic cyclic compo...
0.000763
56 / 21679
GO:1901363
heterocyclic compoun...
0.000692
56 / 21598
GO:0016903
oxidoreductase activ...
0.001013
4 / 247
GO:0016879
ligase activity, for...
0.003195
4 / 339
GO:0003676
nucleic acid binding
0.012008
33 / 12641
GO:0016620
oxidoreductase activ...
0.000279
4 / 175
GO:0016880
acid−ammonia (or ami...
4.71e−05
3 / 39
GO:0003677
DNA binding
0.338672
17 / 8538
GO:0003723
RNA binding
6.47e−06
19 / 3314
GO:0043891
glyceraldehyde−3−pho...
1.71e−05
3 / 28
GO:0016211
ammonia ligase activ...
4.71e−05
3 / 39
GO:0003697
single−stranded DNA ...
0.000203
4 / 161
GO:0047100
glyceraldehyde−3−pho...
0.000138
2 / 10
GO:0004356
glutamate−ammonia li...
1.53e−05
3 / 27

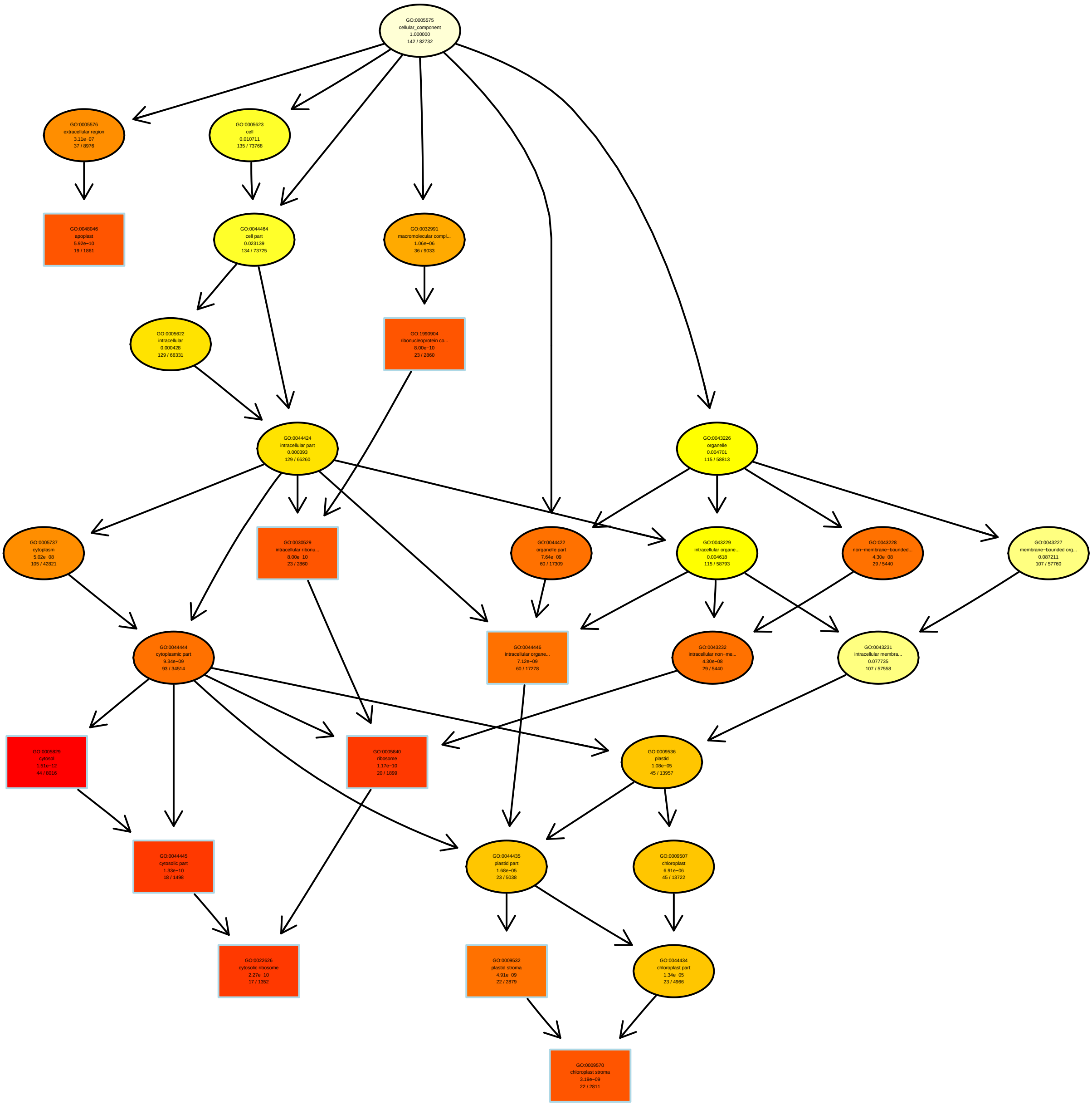
GO:0005575 cellular_component 1.000000 142 / 82732
GO:0005576 extracellular region 3.11e−07 37 / 8976
GO:0005623 cell 0.010711 135 / 73768
GO:0048046 apoplast 5.92e−10 19 / 1861
GO:0044464 cell part 0.023139 134 / 73725
GO:0032991 macromolecular compl... 1.06e−06 36 / 9033
GO:0005622 intracellular 0.000428 129 / 66331
GO:1990904 ribonucleoprotein co... 8.00e−10 23 / 2860
GO:0044424 intracellular part 0.000393 129 / 66260
GO:0043226 organelle 0.004701 115 / 58813
GO:0005737 cytoplasm 5.02e−08 105 / 42821
GO:0030529 intracellular ribonu... 8.00e−10 23 / 2860
GO:0044422 organelle part 7.64e−09 60 / 17309
GO:0043229 intracellular organe... 0.004618 115 / 58793
GO:0043228 non−membrane−bounded... 4.30e−08 29 / 5440
GO:0043227 membrane−bounded org... 0.087211 107 / 57760
GO:0044444 cytoplasmic part 9.34e−09 93 / 34514
GO:0044446 intracellular organe... 7.12e−09 60 / 17278
GO:0043232 intracellular non−me... 4.30e−08 29 / 5440
GO:0043231 intracellular membra... 0.077735 107 / 57558
GO:0005829 cytosol 1.51e−12 44 / 8016
GO:0005840 ribosome 1.17e−10 20 / 1899
GO:0009536 plastid 1.08e−05 45 / 13957
GO:0044445 cytosolic part 1.33e−10 18 / 1498
GO:0044435 plastid part 1.68e−05 23 / 5038
GO:0009507 chloroplast 6.91e−06 45 / 13722
GO:0022626 cytosolic ribosome 2.27e−10 17 / 1352
GO:0009532 plastid stroma 4.91e−09 22 / 2879
GO:0044434 chloroplast part 1.34e−05 23 / 4966
GO:0009570 chloroplast stroma 3.19e−09 22 / 2811
