# Supplementary material for: Differential Alternative Splicing Genes and Isoform Regulation Networks of Rapeseed (Brassica napus L.) Infected with Sclerotinia sclerotiorum
Source: Genes (Basel). 2020 Jul 13;11(7):784. doi: 10.3390/genes11070784 (PMC7397149; doi:10.3390/genes11070784)
Supplement: Supplementary file 1 [file genes-11-00784-s001.zip › Fig. S2 Enriched GO terms of genes in the 85 WGCNA modules.zip!/Fig. S2/CytoNodelightyellow-go.pdf]

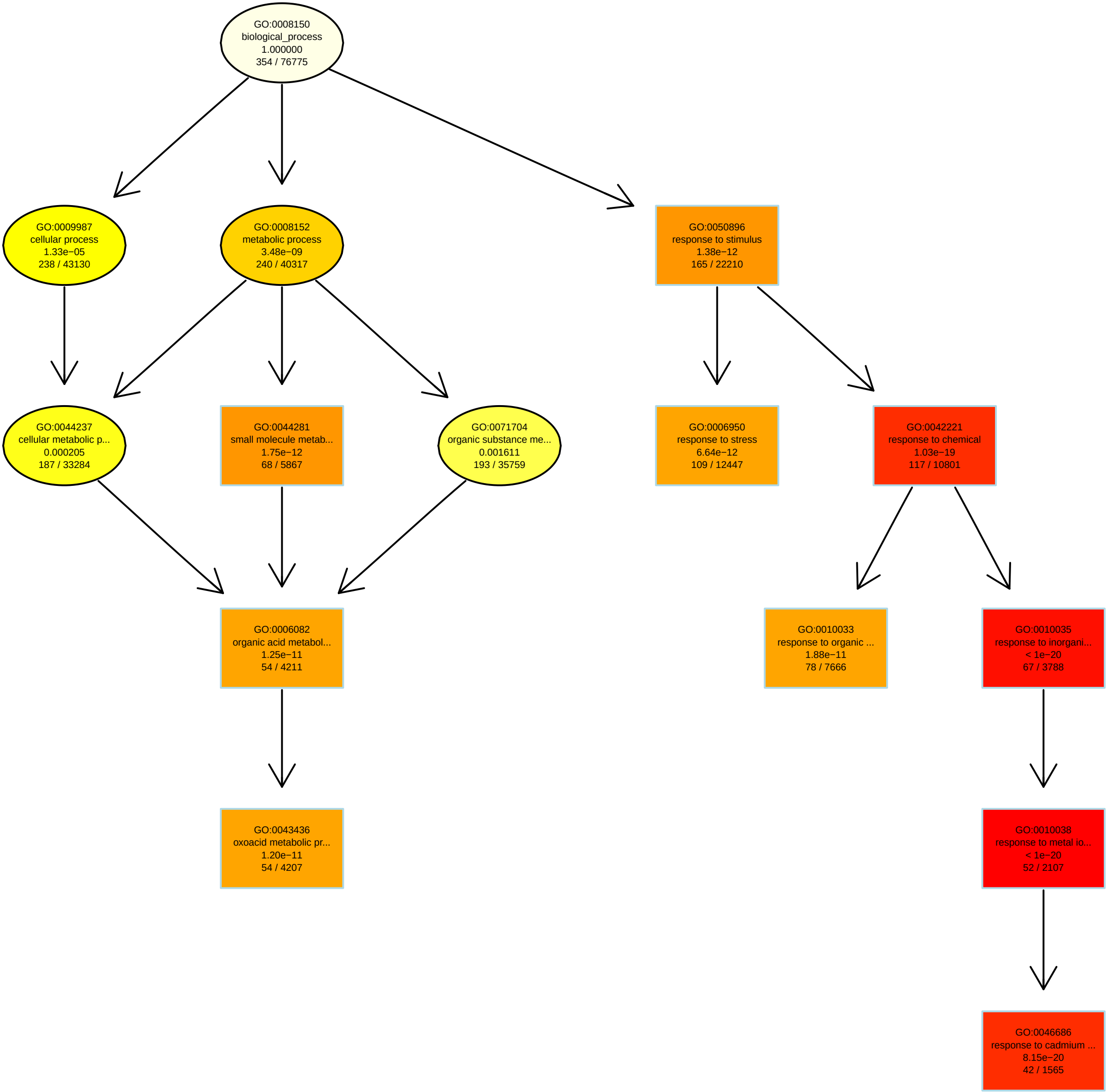
GO:0008150
biological_process
1.000000
354 / 76775
GO:0009987
cellular process
1.33e−05
238 / 43130
GO:0008152
metabolic process
3.48e−09
240 / 40317
GO:0050896
response to stimulus
1.38e−12
165 / 22210
GO:0044237
cellular metabolic p...
0.000205
187 / 33284
GO:0044281
small molecule metab...
1.75e−12
68 / 5867
GO:0071704
organic substance me...
0.001611
193 / 35759
GO:0006950
response to stress
6.64e−12
109 / 12447
GO:0042221
response to chemical
1.03e−19
117 / 10801
GO:0006082
organic acid metabol...
1.25e−11
54 / 4211
GO:0010033
response to organic ...
1.88e−11
78 / 7666
GO:0010035
response to inorgani...
< 1e−20
67 / 3788
GO:0043436
oxoacid metabolic pr...
1.20e−11
54 / 4207
GO:0010038
response to metal io...
< 1e−20
52 / 2107
GO:0046686
response to cadmium ...
8.15e−20
42 / 1565

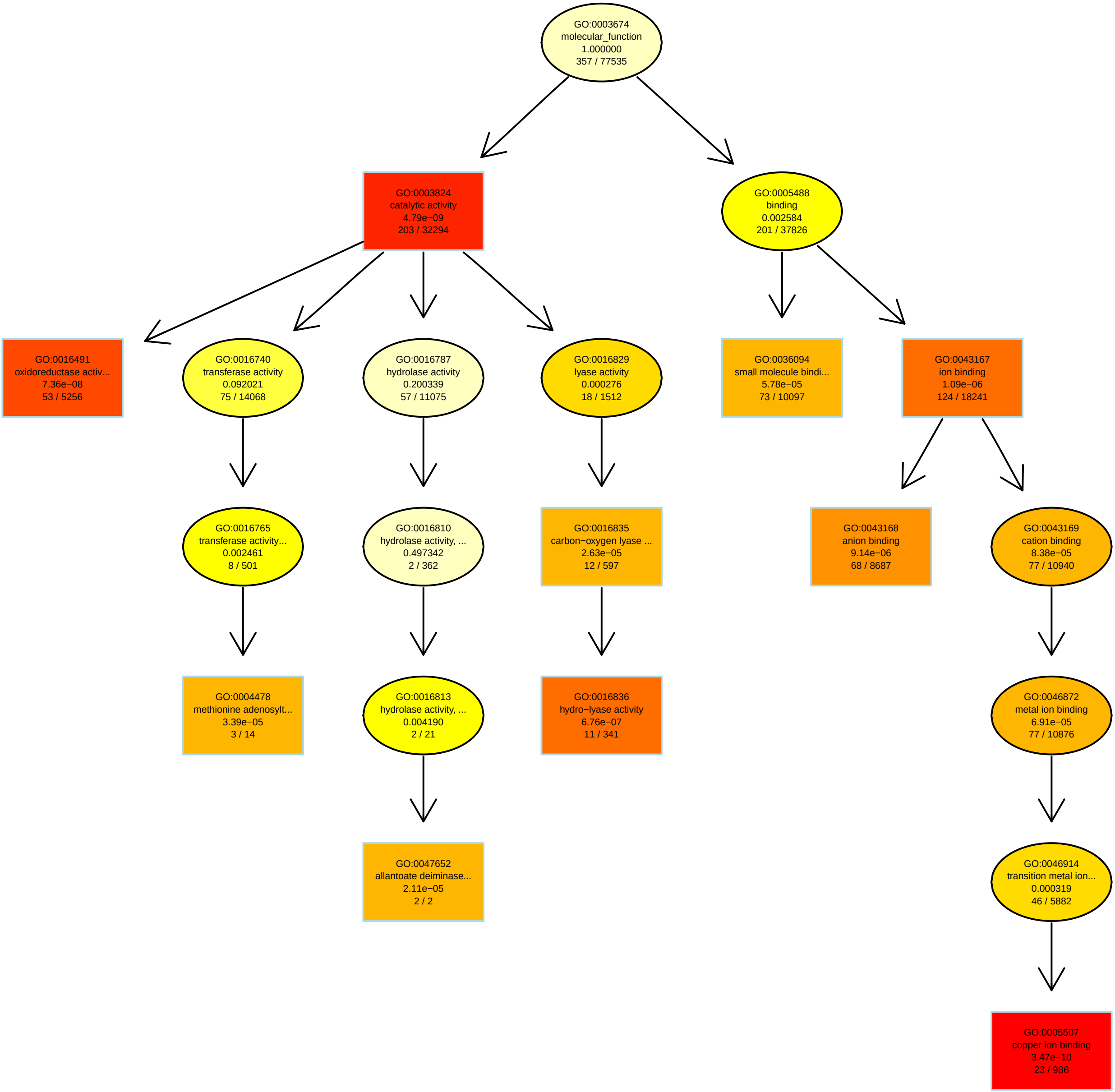
GO:0003674
molecular_function
1.000000
357 / 77535
GO:0003824
catalytic activity
4.79e−09
203 / 32294
GO:0005488
binding
0.002584
201 / 37826
GO:0016491
oxidoreductase activ...
7.36e−08
53 / 5256
GO:0016740
transferase activity
0.092021
75 / 14068
GO:0016787
hydrolase activity
0.200339
57 / 11075
GO:0016829
lyase activity
0.000276
18 / 1512
GO:0036094
small molecule bindi...
5.78e−05
73 / 10097
GO:0043167
ion binding
1.09e−06
124 / 18241
GO:0016765
transferase activity...
0.002461
8 / 501
GO:0016810
hydrolase activity, ...
0.497342
2 / 362
GO:0016835
carbon−oxygen lyase ...
2.63e−05
12 / 597
GO:0043168
anion binding
9.14e−06
68 / 8687
GO:0043169
cation binding
8.38e−05
77 / 10940
GO:0004478
methionine adenosylt...
3.39e−05
3 / 14
GO:0016813
hydrolase activity, ...
0.004190
2 / 21
GO:0016836
hydro−lyase activity
6.76e−07
11 / 341
GO:0046872
metal ion binding
6.91e−05
77 / 10876
GO:0047652
allantoate deiminase...
2.11e−05
2 / 2
GO:0046914
transition metal ion...
0.000319
46 / 5882
GO:0005507
copper ion binding
3.47e−10
23 / 986

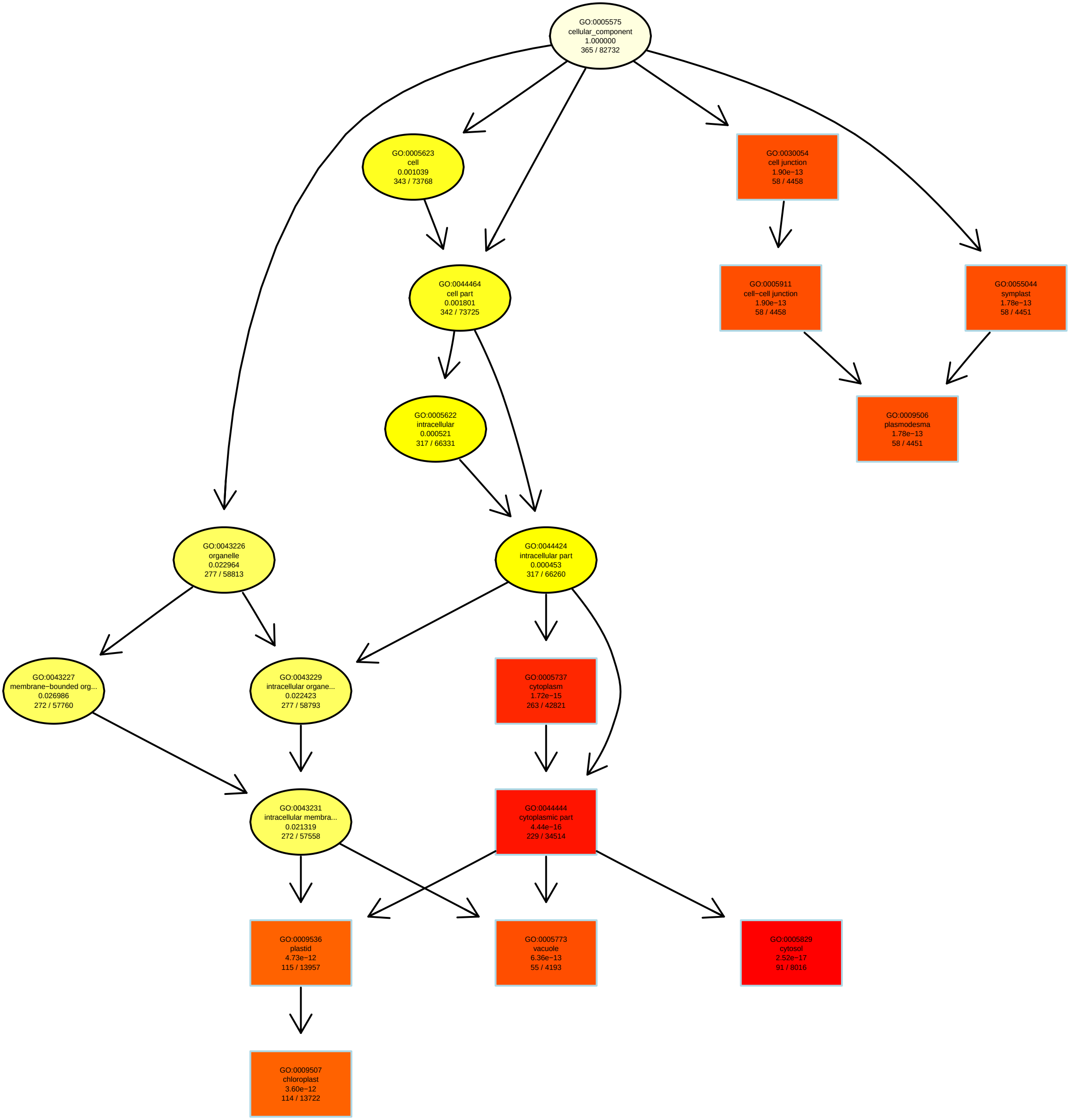
GO:0005575
cellular_component
1.000000
365 / 82732
GO:0005623
cell
0.001039
343 / 73768
GO:0030054
cell junction
1.90e−13
58 / 4458
GO:0044464
cell part
0.001801
342 / 73725
GO:0005911
cell−cell junction
1.90e−13
58 / 4458
GO:0055044
symplast
1.78e−13
58 / 4451
GO:0005622
intracellular
0.000521
317 / 66331
GO:0009506
plasmodesma
1.78e−13
58 / 4451
GO:0043226
organelle
0.022964
277 / 58813
GO:0044424
intracellular part
0.000453
317 / 66260
GO:0043227
membrane−bounded org...
0.026986
272 / 57760
GO:0043229
intracellular organe...
0.022423
277 / 58793
GO:0005737
cytoplasm
1.72e−15
263 / 42821
GO:0043231
intracellular membra...
0.021319
272 / 57558
GO:0044444
cytoplasmic part
4.44e−16
229 / 34514
GO:0009536
plastid
4.73e−12
115 / 13957
GO:0005773
vacuole
6.36e−13
55 / 4193
GO:0005829
cytosol
2.52e−17
91 / 8016
GO:0009507
chloroplast
3.60e−12
114 / 13722
