# Supplementary material for: Differential Alternative Splicing Genes and Isoform Regulation Networks of Rapeseed (Brassica napus L.) Infected with Sclerotinia sclerotiorum
Source: Genes (Basel). 2020 Jul 13;11(7):784. doi: 10.3390/genes11070784 (PMC7397149; doi:10.3390/genes11070784)
Supplement: Supplementary file 1 [file genes-11-00784-s001.zip › Fig. S2 Enriched GO terms of genes in the 85 WGCNA modules.zip!/Fig. S2/CytoNodemagenta-go.pdf]

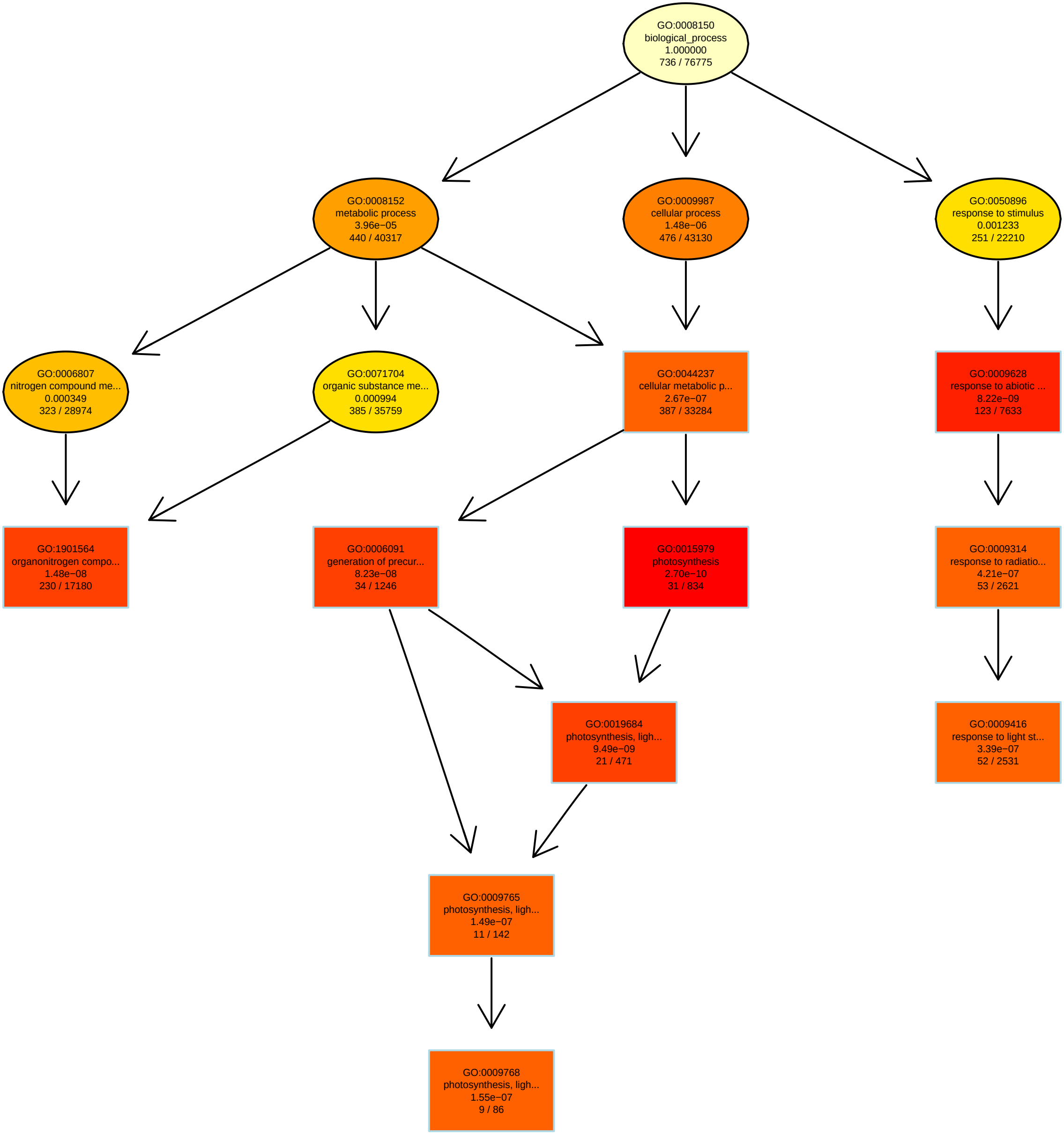
GO:0008150
biological_process
1.000000
736 / 76775
GO:0008152
metabolic process
3.96e−05
440 / 40317
GO:0009987
cellular process
1.48e−06
476 / 43130
GO:0050896
response to stimulus
0.001233
251 / 22210
GO:0006807
nitrogen compound me...
0.000349
323 / 28974
GO:0071704
organic substance me...
0.000994
385 / 35759
GO:0044237
cellular metabolic p...
2.67e−07
387 / 33284
GO:0009628
response to abiotic ...
8.22e−09
123 / 7633
GO:1901564
organonitrogen compo...
1.48e−08
230 / 17180
GO:0006091
generation of precur...
8.23e−08
34 / 1246
GO:0015979
photosynthesis
2.70e−10
31 / 834
GO:0009314
response to radiatio...
4.21e−07
53 / 2621
GO:0019684
photosynthesis, ligh...
9.49e−09
21 / 471
GO:0009416
response to light st...
3.39e−07
52 / 2531
GO:0009765
photosynthesis, ligh...
1.49e−07
11 / 142
GO:0009768
photosynthesis, ligh...
1.55e−07
9 / 86

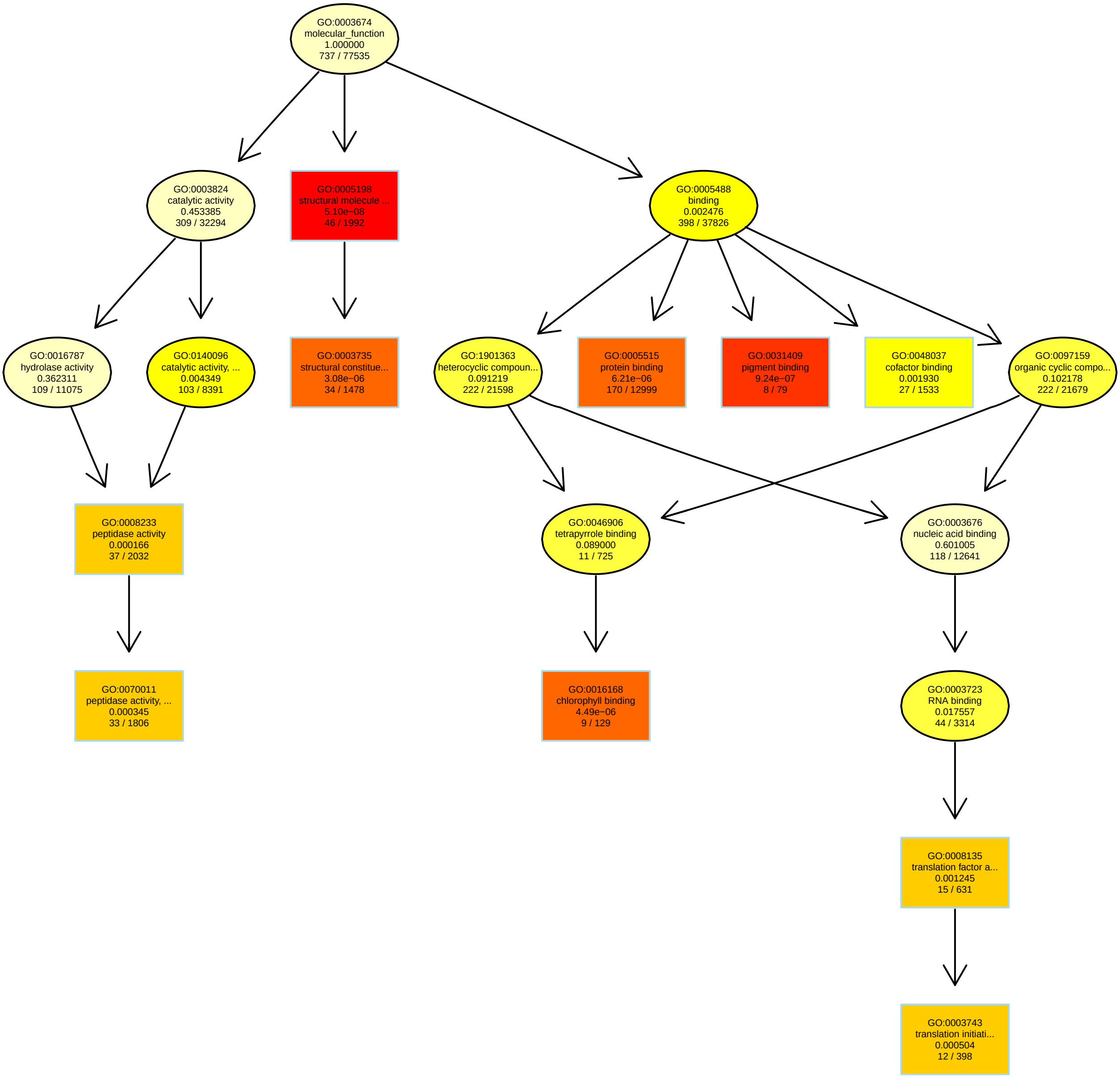
GO:0003674
molecular_function
1.000000
737 / 77535
GO:0003824
catalytic activity
0.453385
309 / 32294
GO:0005198
structural molecule ...
5.10e−08
46 / 1992
GO:0005488
binding
0.002476
398 / 37826
GO:0016787
hydrolase activity
0.362311
109 / 11075
GO:0140096
catalytic activity, ...
0.004349
103 / 8391
GO:0003735
structural constitue...
3.08e−06
34 / 1478
GO:1901363
heterocyclic compoun...
0.091219
222 / 21598
GO:0005515
protein binding
6.21e−06
170 / 12999
GO:0031409
pigment binding
9.24e−07
8 / 79
GO:0048037
cofactor binding
0.001930
27 / 1533
GO:0097159
organic cyclic compo...
0.102178
222 / 21679
GO:0008233
peptidase activity
0.000166
37 / 2032
GO:0046906
tetrapyrrole binding
0.089000
11 / 725
GO:0003676
nucleic acid binding
0.601005
118 / 12641
GO:0070011
peptidase activity, ...
0.000345
33 / 1806
GO:0016168
chlorophyll binding
4.49e−06
9 / 129
GO:0003723
RNA binding
0.017557
44 / 3314
GO:0008135
translation factor a...
0.001245
15 / 631
GO:0003743
translation initiati...
0.000504
12 / 398

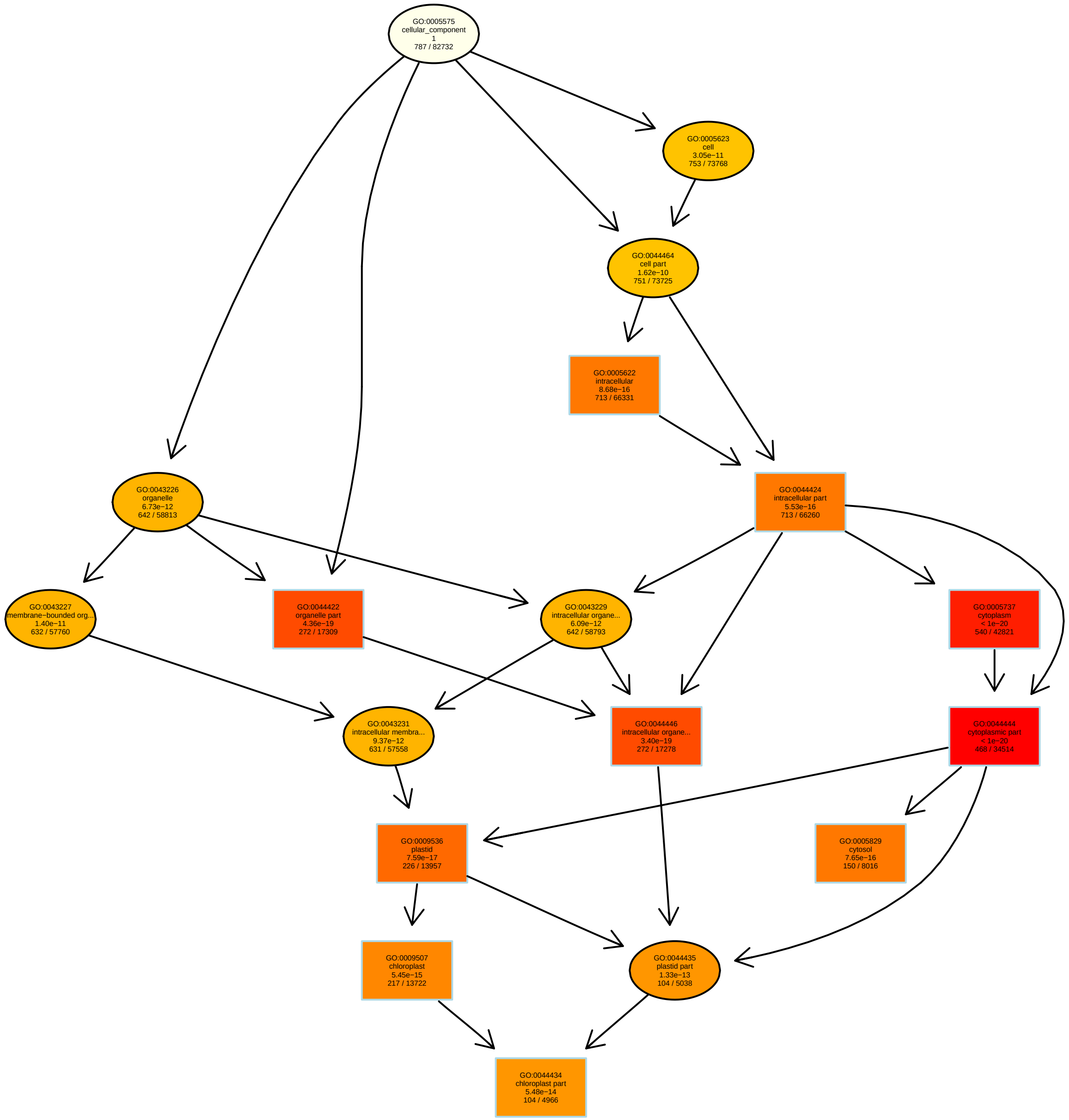

GO:0005575
cellular_component
1
787 / 82732
GO:0005623
cell
3.05e−11
753 / 73768
GO:0044464
cell part
1.62e−10
751 / 73725
GO:0005622
intracellular
8.68e−16
713 / 66331
GO:0043226
organelle
6.73e−12
642 / 58813
GO:0044424
intracellular part
5.53e−16
713 / 66260
GO:0043227
membrane−bounded org...
1.40e−11
632 / 57760
GO:0044422
organelle part
4.36e−19
272 / 17309
GO:0043229
intracellular organe...
6.09e−12
642 / 58793
GO:0005737
cytoplasm
< 1e−20
540 / 42821
GO:0043231
intracellular membra...
9.37e−12
631 / 57558
GO:0044446
intracellular organe...
3.40e−19
272 / 17278
GO:0044444
cytoplasmic part
< 1e−20
468 / 34514
GO:0009536
plastid
7.59e−17
226 / 13957
GO:0005829
cytosol
7.65e−16
150 / 8016
GO:0009507
chloroplast
5.45e−15
217 / 13722
GO:0044435
plastid part
1.33e−13
104 / 5038
GO:0044434
chloroplast part
5.48e−14
104 / 4966
