# Supplementary material for: Differential Alternative Splicing Genes and Isoform Regulation Networks of Rapeseed (Brassica napus L.) Infected with Sclerotinia sclerotiorum
Source: Genes (Basel). 2020 Jul 13;11(7):784. doi: 10.3390/genes11070784 (PMC7397149; doi:10.3390/genes11070784)
Supplement: Supplementary file 1 [file genes-11-00784-s001.zip › Fig. S2 Enriched GO terms of genes in the 85 WGCNA modules.zip!/Fig. S2/CytoNodemagenta4-go.pdf]

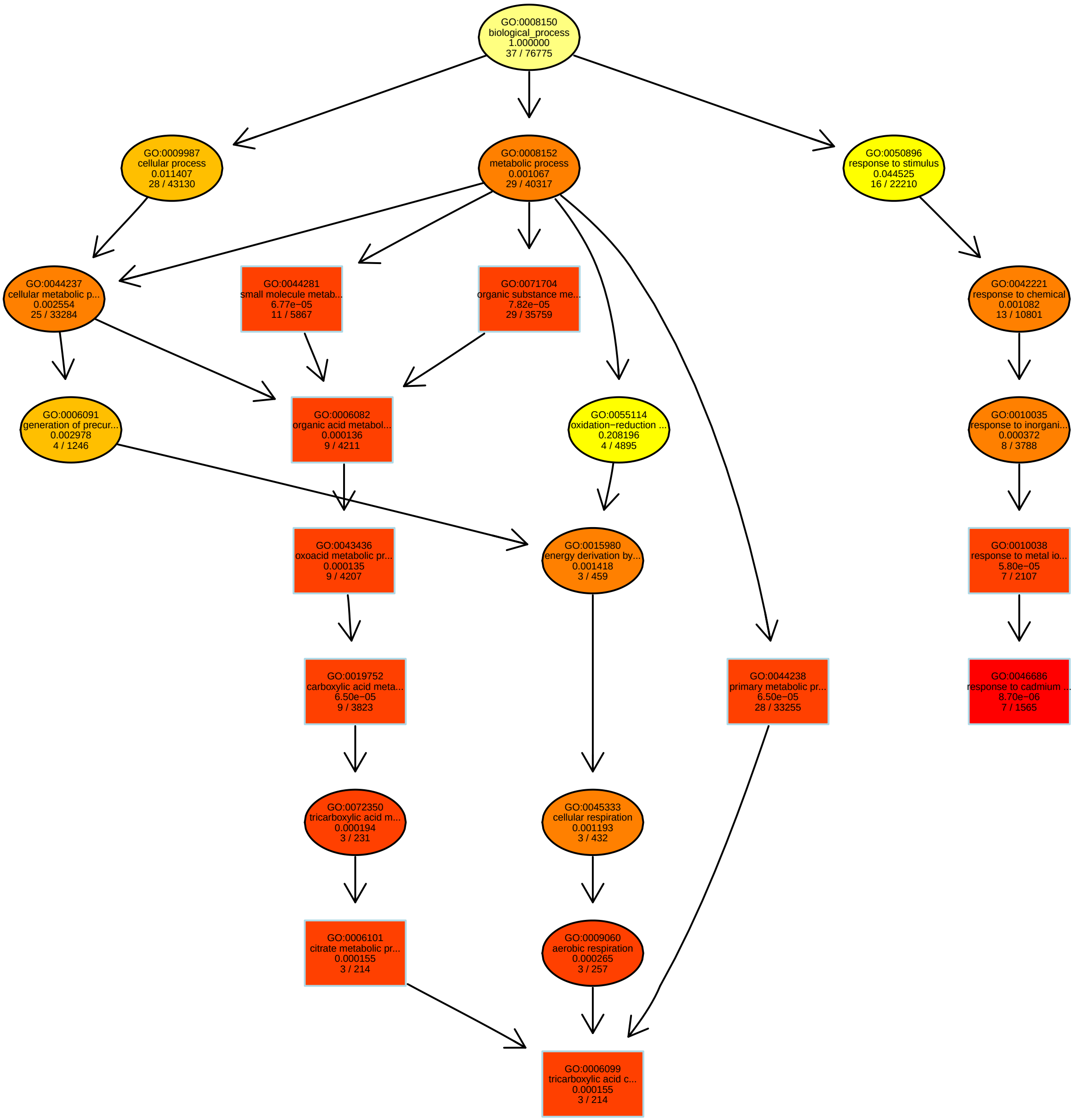

GO:0008150
biological_process
1.000000
37 / 76775
GO:0009987
cellular process
0.011407
28 / 43130
GO:0008152
metabolic process
0.001067
29 / 40317
GO:0050896
response to stimulus
0.044525
16 / 22210
GO:0044237
cellular metabolic p...
0.002554
25 / 33284
GO:0044281
small molecule metab...
6.77e−05
11 / 5867
GO:0071704
organic substance me...
7.82e−05
29 / 35759
GO:0042221
response to chemical
0.001082
13 / 10801
GO:0006091
generation of precur...
0.002978
4 / 1246
GO:0006082
organic acid metabol...
0.000136
9 / 4211
GO:0055114
oxidation−reduction ...
0.208196
4 / 4895
GO:0010035
response to inorgani...
0.000372
8 / 3788
GO:0043436
oxoacid metabolic pr...
0.000135
9 / 4207
GO:0015980
energy derivation by...
0.001418
3 / 459
GO:0010038
response to metal io...
5.80e−05
7 / 2107
GO:0019752
carboxylic acid meta...
6.50e−05
9 / 3823
GO:0044238
primary metabolic pr...
6.50e−05
28 / 33255
GO:0046686
response to cadmium ...
8.70e−06
7 / 1565
GO:0072350
tricarboxylic acid m...
0.000194
3 / 231
GO:0045333
cellular respiration
0.001193
3 / 432
GO:0006101
citrate metabolic pr...
0.000155
3 / 214
GO:0009060
aerobic respiration
0.000265
3 / 257
GO:0006099
tricarboxylic acid c...
0.000155
3 / 214

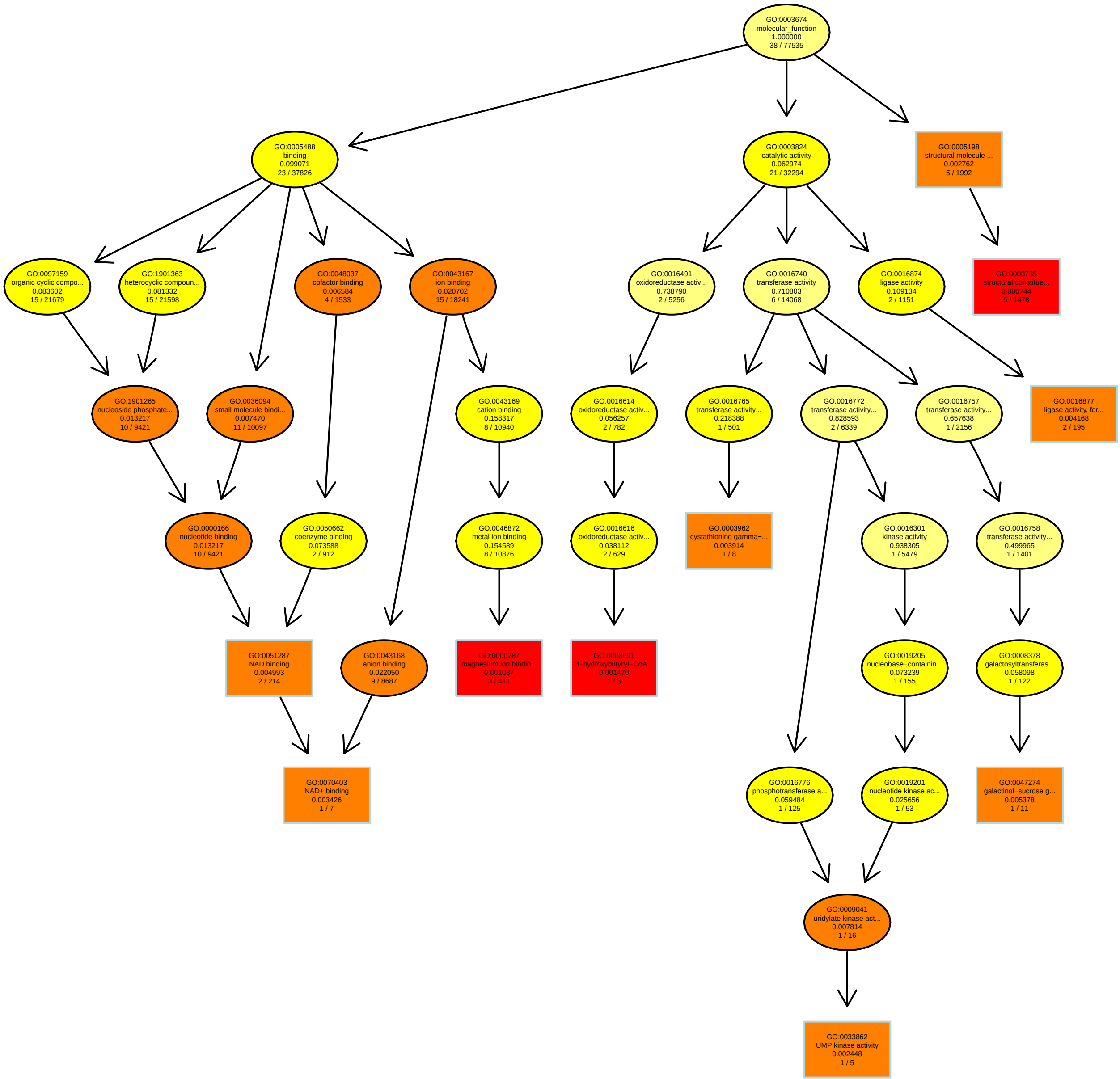
GO:0003674 molecular_function 1.000000 38 / 77535
GO:0005488 binding 0.099071 23 / 37826
GO:0003824 catalytic activity 0.062974 21 / 32294
GO:0005198 structural molecule ... 0.002762 5 / 1992
GO:0097159 organic cyclic compo... 0.083602 15 / 21679
GO:1901363 heterocyclic compoun... 0.081332 15 / 21598
GO:0048037 cofactor binding 0.006584 4 / 1533
GO:0043167 ion binding 0.020702 15 / 18241
GO:0016491 oxidoreductase activ... 0.738790 2 / 5256
GO:0016740 transferase activity 0.710803 6 / 14068
GO:0016874 ligase activity 0.109134 2 / 1151
GO:0003735 structural constitue... 0.000744 5 / 1478
GO:1901265 nucleoside phosphate... 0.013217 10 / 9421
GO:0036094 small molecule bindi... 0.007470 11 / 10097
GO:0043169 cation binding 0.158317 8 / 10940
GO:0016614 oxidoreductase activ... 0.056257 2 / 782
GO:0016765 transferase activity... 0.218388 1 / 501
GO:0016772 transferase activity... 0.828593 2 / 6339
GO:0016757 transferase activity... 0.657638 1 / 2156
GO:0016877 ligase activity, for... 0.004168 2 / 195
GO:0000166 nucleotide binding 0.013217 10 / 9421
GO:0050662 coenzyme binding 0.073588 2 / 912
GO:0046872 metal ion binding 0.154589 8 / 10876
GO:0016616 oxidoreductase activ... 0.038112 2 / 629
GO:0003962 cystathionine gamma−... 0.003914 1 / 8
GO:0016301 kinase activity 0.938305 1 / 5479
GO:0016758 transferase activity... 0.499965 1 / 1401
GO:0051287 NAD binding 0.004993 2 / 214
GO:0043168 anion binding 0.022050 9 / 8687
GO:0000287 magnesium ion bindin... 0.001087 3 / 411
GO:0008691 3−hydroxybutyryl−CoA... 0.001470 1 / 3
GO:0019205 nucleobase−containin... 0.073239 1 / 155
GO:0008378 galactosyltransferas... 0.058098 1 / 122
GO:0070403 NAD+ binding 0.003426 1 / 7
GO:0016776 phosphotransferase a... 0.059484 1 / 125
GO:0019201 nucleotide kinase ac... 0.025656 1 / 53
GO:0047274 galactinol−sucrose g... 0.005378 1 / 11
GO:0009041 uridylate kinase act... 0.007814 1 / 16
GO:0033862 UMP kinase activity 0.002448 1 / 5

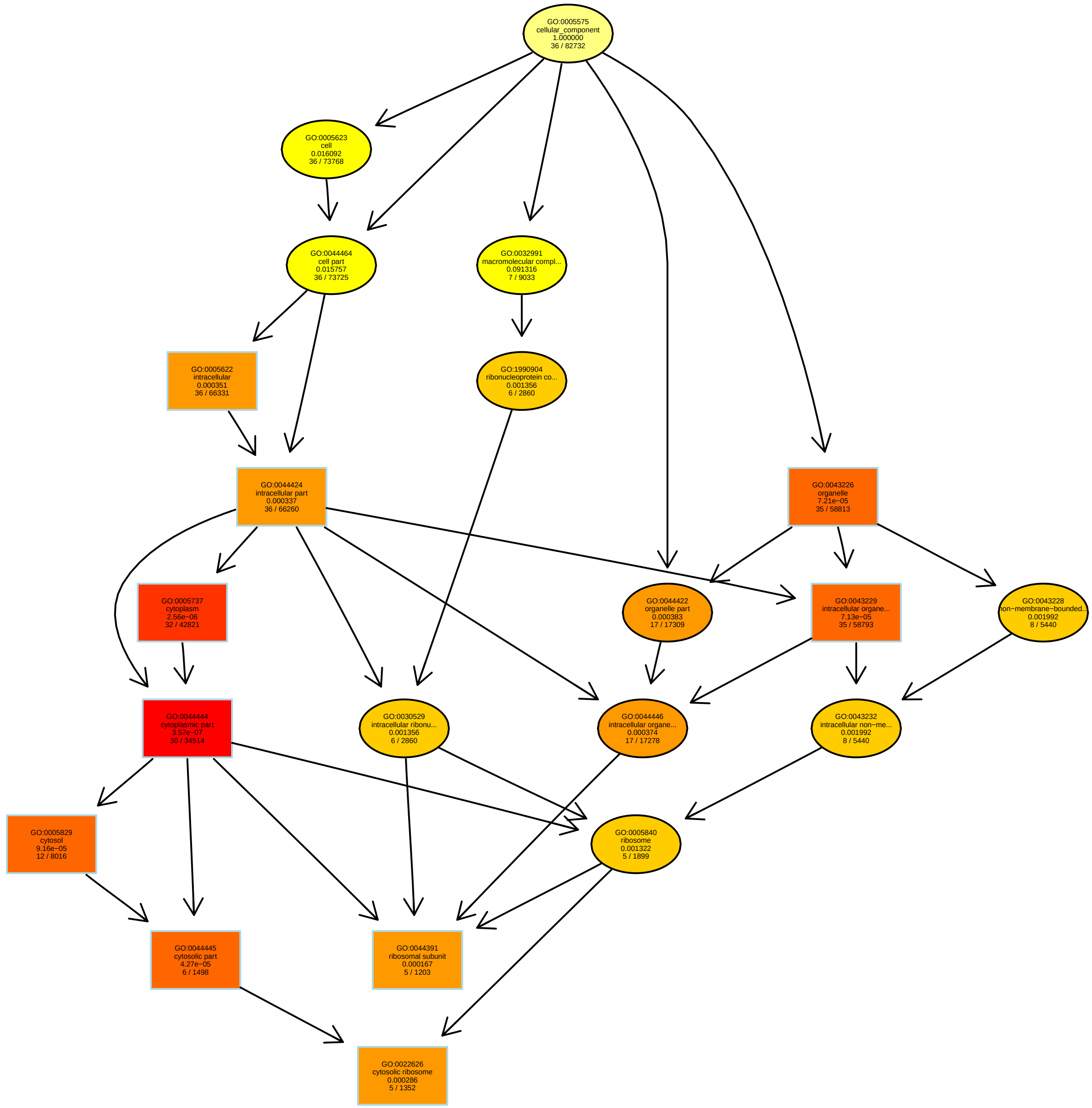
GO:0005575
cellular_component
1.000000
36 / 82732
GO:0005623
cell
0.016092
36 / 73768
GO:0044464
cell part
0.015757
36 / 73725
GO:0032991
macromolecular compl...
0.091316
7 / 9033
GO:0005622
intracellular
0.000351
36 / 66331
GO:1990904
ribonucleoprotein co...
0.001356
6 / 2860
GO:0044424
intracellular part
0.000337
36 / 66260
GO:0043226
organelle
7.21e−05
35 / 58813
GO:0005737
cytoplasm
2.56e−06
32 / 42821
GO:0044422
organelle part
0.000383
17 / 17309
GO:0043229
intracellular organe...
7.13e−05
35 / 58793
GO:0043228
non−membrane−bounded...
0.001992
8 / 5440
GO:0044444
cytoplasmic part
3.57e−07
30 / 34514
GO:0030529
intracellular ribonu...
0.001356
6 / 2860
GO:0044446
intracellular organe...
0.000374
17 / 17278
GO:0043232
intracellular non−me...
0.001992
8 / 5440
GO:0005829
cytosol
9.16e−05
12 / 8016
GO:0005840
ribosome
0.001322
5 / 1899
GO:0044445
cytosolic part
4.27e−05
6 / 1498
GO:0044391
ribosomal subunit
0.000167
5 / 1203
GO:0022626
cytosolic ribosome
0.000286
5 / 1352
