# Supplementary material for: Differential Alternative Splicing Genes and Isoform Regulation Networks of Rapeseed (Brassica napus L.) Infected with Sclerotinia sclerotiorum
Source: Genes (Basel). 2020 Jul 13;11(7):784. doi: 10.3390/genes11070784 (PMC7397149; doi:10.3390/genes11070784)
Supplement: Supplementary file 1 [file genes-11-00784-s001.zip › Fig. S2 Enriched GO terms of genes in the 85 WGCNA modules.zip!/Fig. S2/CytoNodemaroon-go.pdf]

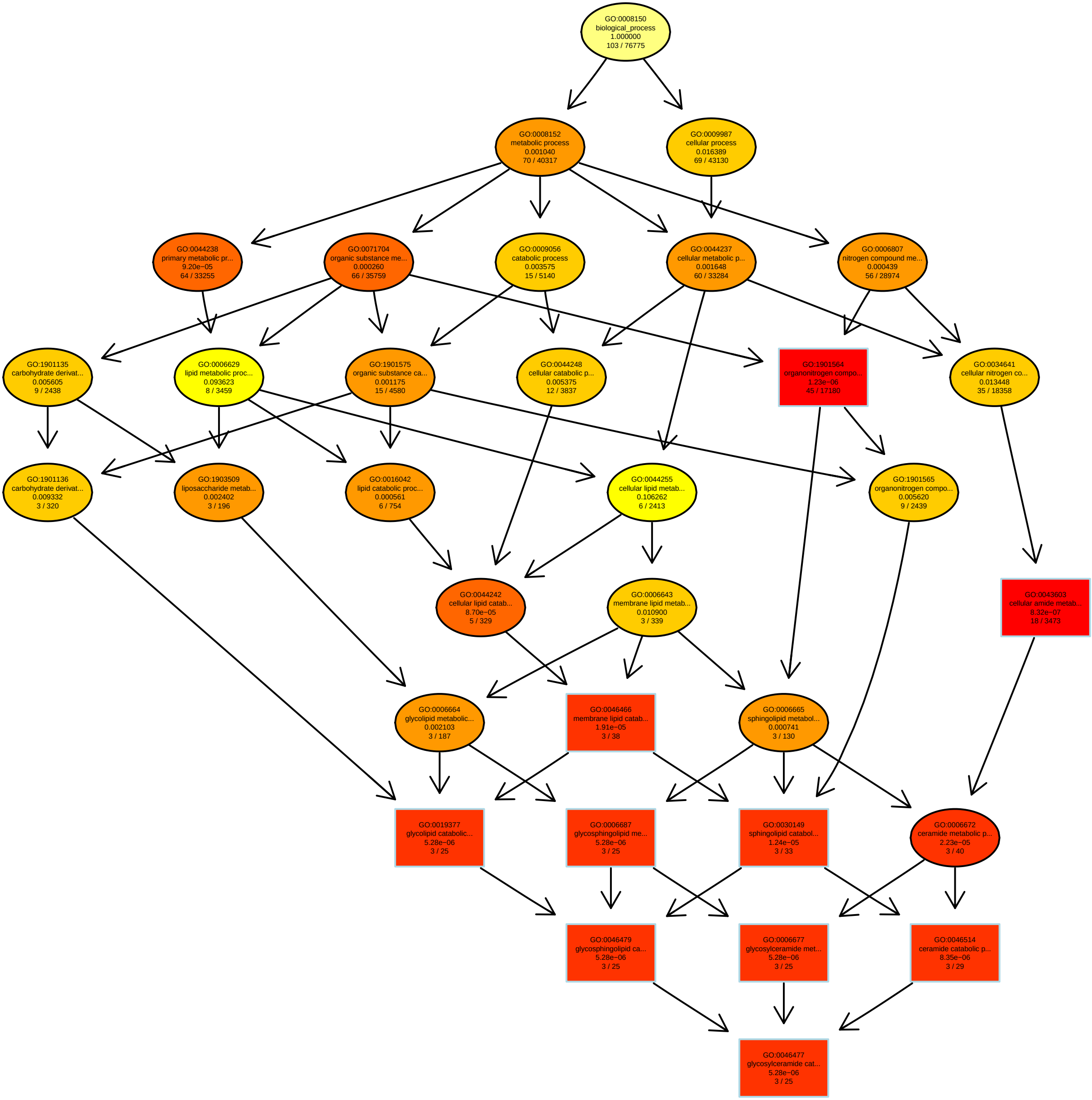
GO:0008150
biological_process
1.000000
103 / 76775
GO:0008152
metabolic process
0.001040
70 / 40317
GO:0009987
cellular process
0.016389
69 / 43130
GO:0044238
primary metabolic pr...
9.20e−05
64 / 33255
GO:0071704
organic substance me...
0.000260
66 / 35759
GO:0009056
catabolic process
0.003575
15 / 5140
GO:0044237
cellular metabolic p...
0.001648
60 / 33284
GO:0006807
nitrogen compound me...
0.000439
56 / 28974
GO:1901135
carbohydrate derivat...
0.005605
9 / 2438
GO:0006629
lipid metabolic proc...
0.093623
8 / 3459
GO:1901575
organic substance ca...
0.001175
15 / 4580
GO:0044248
cellular catabolic p...
0.005375
12 / 3837
GO:1901564
organonitrogen compo...
1.23e−06
45 / 17180
GO:0034641
cellular nitrogen co...
0.013448
35 / 18358
GO:1901136
carbohydrate derivat...
0.009332
3 / 320
GO:1903509
liposaccharide metab...
0.002402
3 / 196
GO:0016042
lipid catabolic proc...
0.000561
6 / 754
GO:0044255
cellular lipid metab...
0.106262
6 / 2413
GO:1901565
organonitrogen compo...
0.005620
9 / 2439
GO:0044242
cellular lipid catab...
8.70e−05
5 / 329
GO:0006643
membrane lipid metab...
0.010900
3 / 339
GO:0043603
cellular amide metab...
8.32e−07
18 / 3473
GO:0006664
glycolipid metabolic...
0.002103
3 / 187
GO:0046466
membrane lipid catab...
1.91e−05
3 / 38
GO:0006665
sphingolipid metabol...
0.000741
3 / 130
GO:0019377
glycolipid catabolic...
5.28e−06
3 / 25
GO:0006687
glycosphingolipid me...
5.28e−06
3 / 25
GO:0030149
sphingolipid catabol...
1.24e−05
3 / 33
GO:0006672
ceramide metabolic p...
2.23e−05
3 / 40
GO:0046479
glycosphingolipid ca...
5.28e−06
3 / 25
GO:0006677
glycosylceramide met...
5.28e−06
3 / 25
GO:0046514
ceramide catabolic p...
8.35e−06
3 / 29
GO:0046477
glycosylceramide cat...
5.28e−06
3 / 25

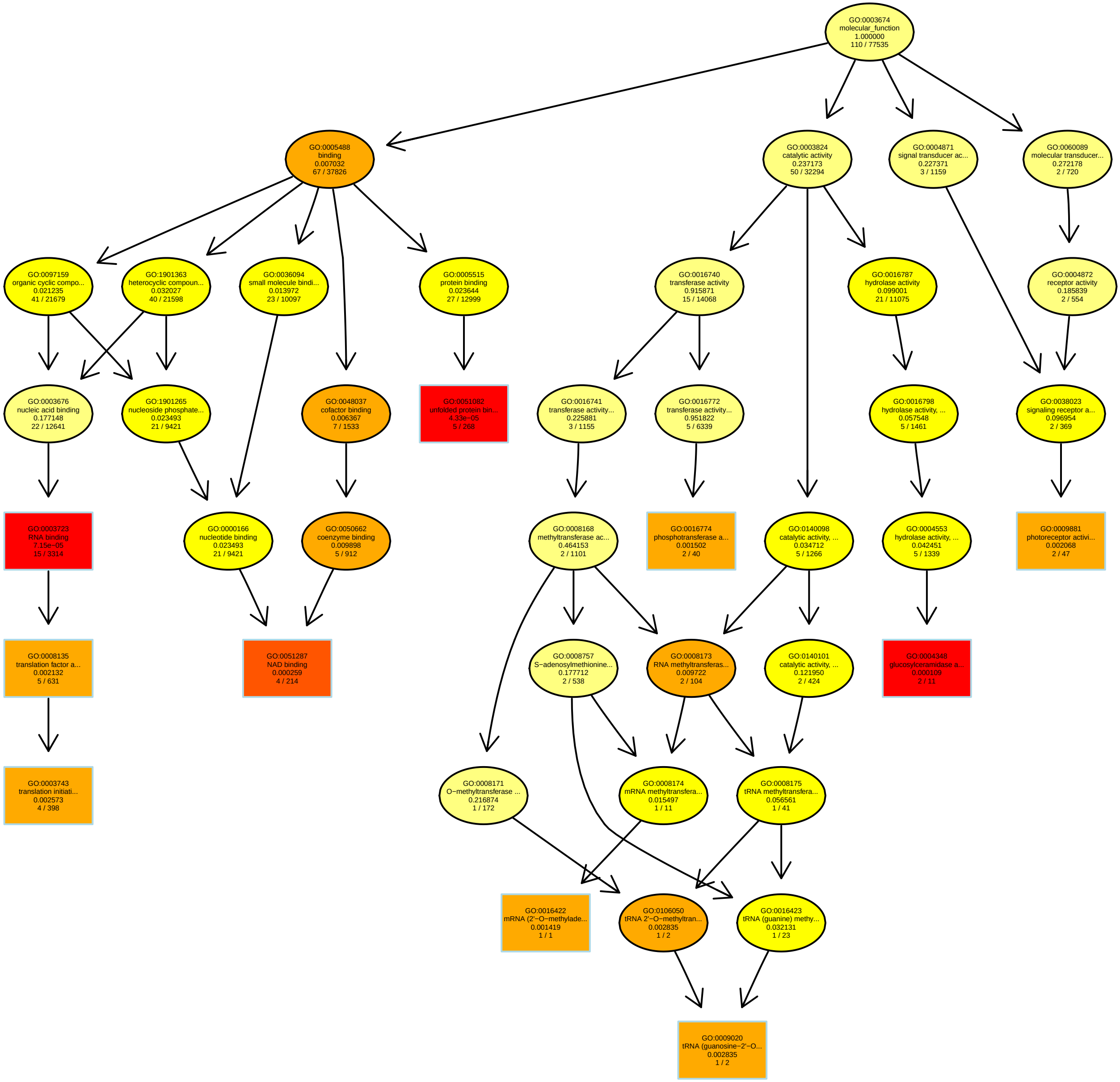
GO:0003674 molecular_function 1.000000 110 / 77535
GO:0005488 binding 0.007032 67 / 37826
GO:0003824 catalytic activity 0.237173 50 / 32294
GO:0004871 signal transducer ac... 0.227371 3 / 1159
GO:0060089 molecular transducer... 0.272178 2 / 720
GO:0097159 organic cyclic compo... 0.021235 41 / 21679
GO:1901363 heterocyclic compoun... 0.032027 40 / 21598
GO:0036094 small molecule bindi... 0.013972 23 / 10097
GO:0005515 protein binding 0.023644 27 / 12999
GO:0016740 transferase activity 0.915871 15 / 14068
GO:0016787 hydrolase activity 0.099001 21 / 11075
GO:0004872 receptor activity 0.185839 2 / 554
GO:0003676 nucleic acid binding 0.177148 22 / 12641
GO:1901265 nucleoside phosphate... 0.023493 21 / 9421
GO:0048037 cofactor binding 0.006367 7 / 1533
GO:0051082 unfolded protein bin... 4.33e−05 5 / 268
GO:0016741 transferase activity... 0.225881 3 / 1155
GO:0016772 transferase activity... 0.951822 5 / 6339
GO:0016798 hydrolase activity, ... 0.057548 5 / 1461
GO:0038023 signaling receptor a... 0.096954 2 / 369
GO:0003723 RNA binding 7.15e−05 15 / 3314
GO:0000166 nucleotide binding 0.023493 21 / 9421
GO:0050662 coenzyme binding 0.009898 5 / 912
GO:0008168 methyltransferase ac... 0.464153 2 / 1101
GO:0016774 phosphotransferase a... 0.001502 2 / 40
GO:0140098 catalytic activity, ... 0.034712 5 / 1266
GO:0004553 hydrolase activity, ... 0.042451 5 / 1339
GO:0009881 photoreceptor activi... 0.002068 2 / 47
GO:0008135 translation factor a... 0.002132 5 / 631
GO:0051287 NAD binding 0.000259 4 / 214
GO:0008757 S−adenosylmethionine... 0.177712 2 / 538
GO:0008173 RNA methyltransferas... 0.009722 2 / 104
GO:0140101 catalytic activity, ... 0.121950 2 / 424
GO:0004348 glucosylceramidase a... 0.000109 2 / 11
GO:0003743 translation initiati... 0.002573 4 / 398
GO:0008171 O−methyltransferase ... 0.216874 1 / 172
GO:0008174 mRNA methyltransfera... 0.015497 1 / 11
GO:0008175 tRNA methyltransfera... 0.056561 1 / 41
GO:0016422 mRNA (2'−O−methylade... 0.001419 1 / 1
GO:0106050 tRNA 2'−O−methyltran... 0.002835 1 / 2
GO:0016423 tRNA (guanine) methy... 0.032131 1 / 23
GO:0009020 tRNA (guanosine−2'−O... 0.002835 1 / 2

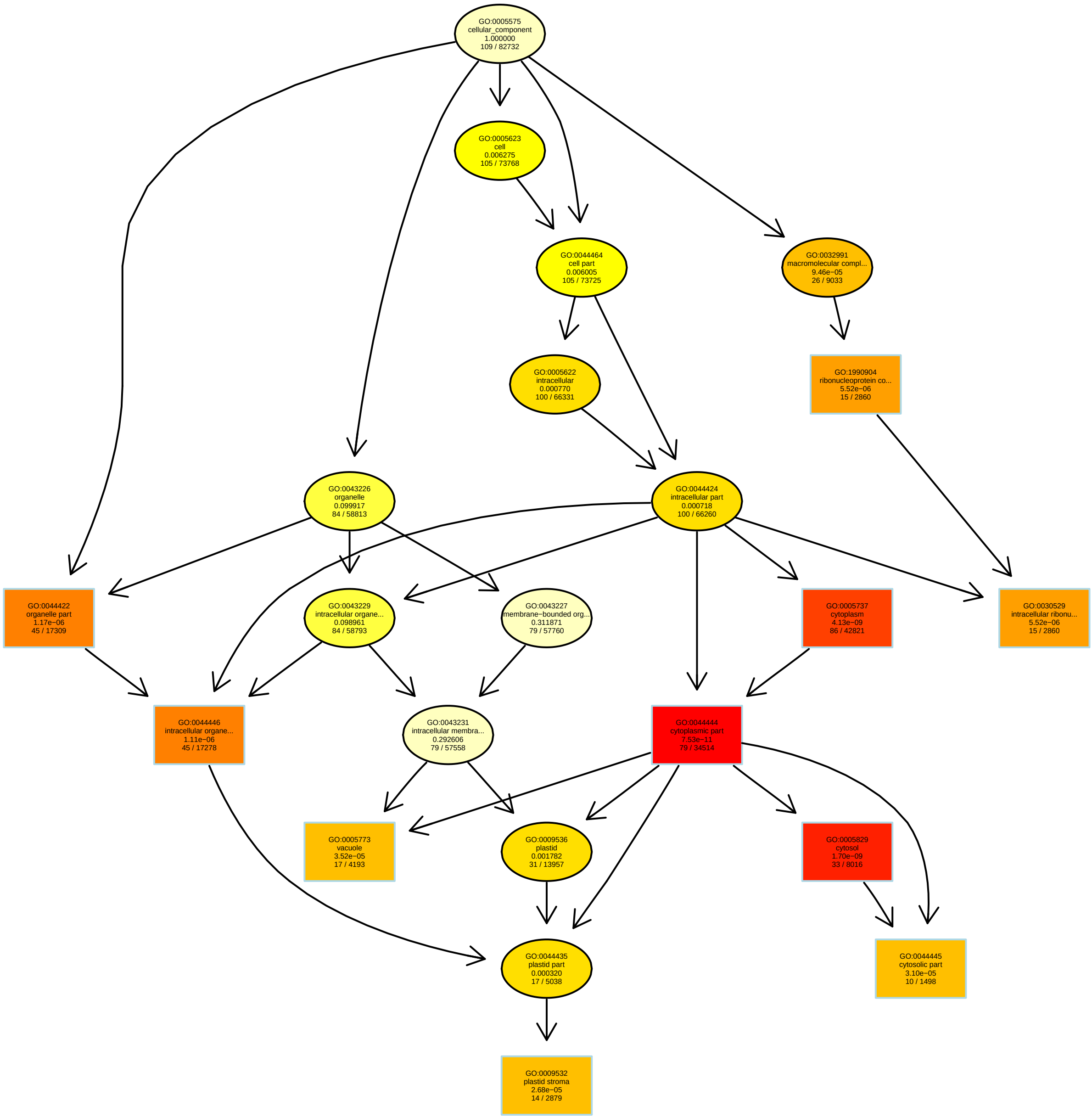
GO:0005575
cellular_component
1.000000
109 / 82732
GO:0005623
cell
0.006275
105 / 73768
GO:0044464
cell part
0.006005
105 / 73725
GO:0032991
macromolecular compl...
9.46e−05
26 / 9033
GO:0005622
intracellular
0.000770
100 / 66331
GO:1990904
ribonucleoprotein co...
5.52e−06
15 / 2860
GO:0043226
organelle
0.099917
84 / 58813
GO:0044424
intracellular part
0.000718
100 / 66260
GO:0044422
organelle part
1.17e−06
45 / 17309
GO:0043229
intracellular organe...
0.098961
84 / 58793
GO:0043227
membrane−bounded org...
0.311871
79 / 57760
GO:0005737
cytoplasm
4.13e−09
86 / 42821
GO:0030529
intracellular ribonu...
5.52e−06
15 / 2860
GO:0044446
intracellular organe...
1.11e−06
45 / 17278
GO:0043231
intracellular membra...
0.292606
79 / 57558
GO:0044444
cytoplasmic part
7.53e−11
79 / 34514
GO:0005773
vacuole
3.52e−05
17 / 4193
GO:0009536
plastid
0.001782
31 / 13957
GO:0005829
cytosol
1.70e−09
33 / 8016
GO:0044435
plastid part
0.000320
17 / 5038
GO:0044445
cytosolic part
3.10e−05
10 / 1498
GO:0009532
plastid stroma
2.68e−05
14 / 2879
