# Supplementary material for: Differential Alternative Splicing Genes and Isoform Regulation Networks of Rapeseed (Brassica napus L.) Infected with Sclerotinia sclerotiorum
Source: Genes (Basel). 2020 Jul 13;11(7):784. doi: 10.3390/genes11070784 (PMC7397149; doi:10.3390/genes11070784)
Supplement: Supplementary file 1 [file genes-11-00784-s001.zip › Fig. S2 Enriched GO terms of genes in the 85 WGCNA modules.zip!/Fig. S2/CytoNodemediumorchid-go.pdf]

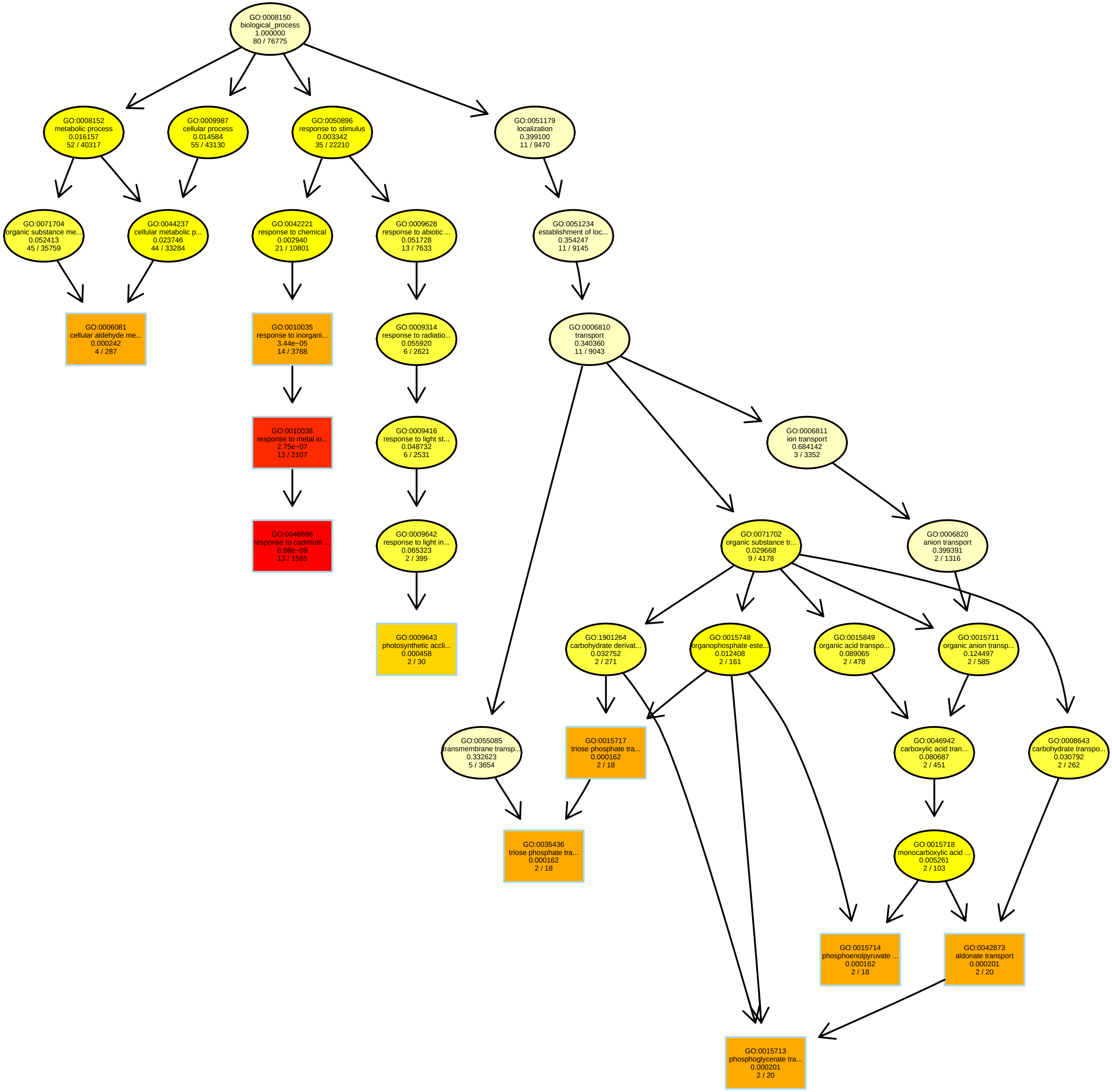
GO:0008150 biological_process 1.000000 80 / 76775
GO:0008152 metabolic process 0.016157 52 / 40317
GO:0009987 cellular process 0.014584 55 / 43130
GO:0050896 response to stimulus 0.003342 35 / 22210
GO:0051179 localization 0.399100 11 / 9470
GO:0071704 organic substance me... 0.052413 45 / 35759
GO:0044237 cellular metabolic p... 0.023746 44 / 33284
GO:0042221 response to chemical 0.002940 21 / 10801
GO:0009628 response to abiotic ... 0.051728 13 / 7633
GO:0051234 establishment of loc... 0.354247 11 / 9145
GO:0006081 cellular aldehyde me... 0.000242 4 / 287
GO:0010035 response to inorgani... 3.44e−05 14 / 3788
GO:0009314 response to radiatio... 0.055920 6 / 2621
GO:0006810 transport 0.340360 11 / 9043
GO:0010038 response to metal io... 2.75e−07 13 / 2107
GO:0009416 response to light st... 0.048732 6 / 2531
GO:0006811 ion transport 0.684142 3 / 3352
GO:0046686 response to cadmium ... 8.88e−09 13 / 1565
GO:0009642 response to light in... 0.065323 2 / 399
GO:0071702 organic substance tr... 0.029668 9 / 4178
GO:0006820 anion transport 0.399391 2 / 1316
GO:0009643 photosynthetic accli... 0.000458 2 / 30
GO:1901264 carbohydrate derivat... 0.032752 2 / 271
GO:0015748 organophosphate este... 0.012408 2 / 161
GO:0015849 organic acid transpo... 0.089065 2 / 478
GO:0015711 organic anion transp... 0.124497 2 / 585
GO:0055085 transmembrane transp... 0.332623 5 / 3654
GO:0015717 triose phosphate tra... 0.000162 2 / 18
GO:0046942 carboxylic acid tran... 0.080687 2 / 451
GO:0008643 carbohydrate transpo... 0.030792 2 / 262
GO:0035436 triose phosphate tra... 0.000162 2 / 18
GO:0015718 monocarboxylic acid ... 0.005261 2 / 103
GO:0015714 phosphoenolpyruvate ... 0.000162 2 / 18
GO:0042873 aldonate transport 0.000201 2 / 20
GO:0015713 phosphoglycerate tra... 0.000201 2 / 20

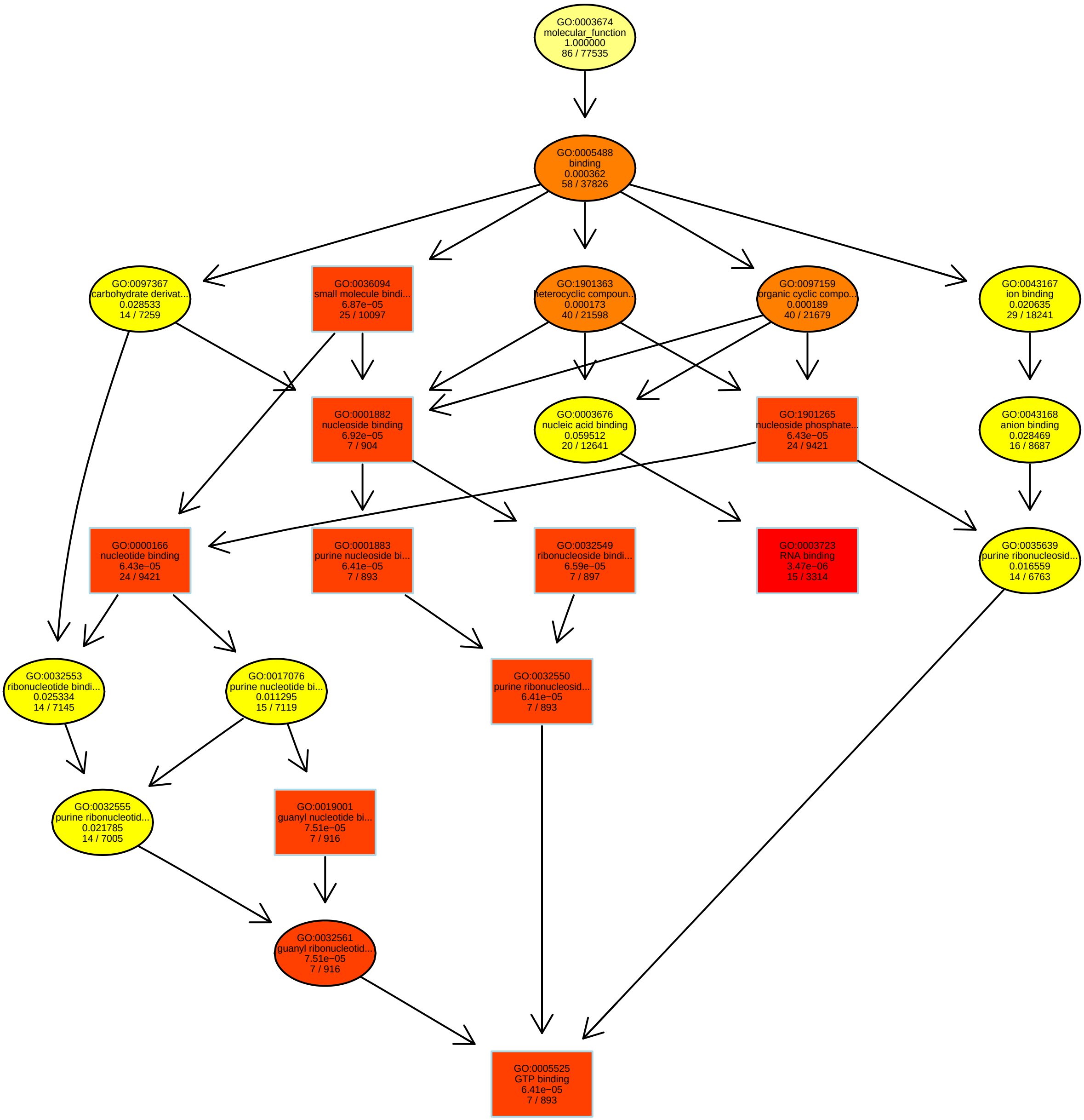

GO:0003674 molecular_function 1.000000 86 / 77535
GO:0005488 binding 0.000362 58 / 37826
GO:0097367 carbohydrate derivat... 0.028533 14 / 7259
GO:0036094 small molecule bindi... 6.87e−05 25 / 10097
GO:1901363 heterocyclic compoun... 0.000173 40 / 21598
GO:0097159 organic cyclic compo... 0.000189 40 / 21679
GO:0043167 ion binding 0.020635 29 / 18241
GO:0001882 nucleoside binding 6.92e−05 7 / 904
GO:0003676 nucleic acid binding 0.059512 20 / 12641
GO:1901265 nucleoside phosphate... 6.43e−05 24 / 9421
GO:0043168 anion binding 0.028469 16 / 8687
GO:0000166 nucleotide binding 6.43e−05 24 / 9421
GO:0001883 purine nucleoside bi... 6.41e−05 7 / 893
GO:0032549 ribonucleoside bindi... 6.59e−05 7 / 897
GO:0003723 RNA binding 3.47e−06 15 / 3314
GO:0035639 purine ribonucleosid... 0.016559 14 / 6763
GO:0032553 ribonucleotide bindi... 0.025334 14 / 7145
GO:0017076 purine nucleotide bi... 0.011295 15 / 7119
GO:0032550 purine ribonucleosid... 6.41e−05 7 / 893
GO:0032555 purine ribonucleotid... 0.021785 14 / 7005
GO:0019001 guanyl nucleotide bi... 7.51e−05 7 / 916
GO:0032561 guanyl ribonucleotid... 7.51e−05 7 / 916
GO:0005525 GTP binding 6.41e−05 7 / 893

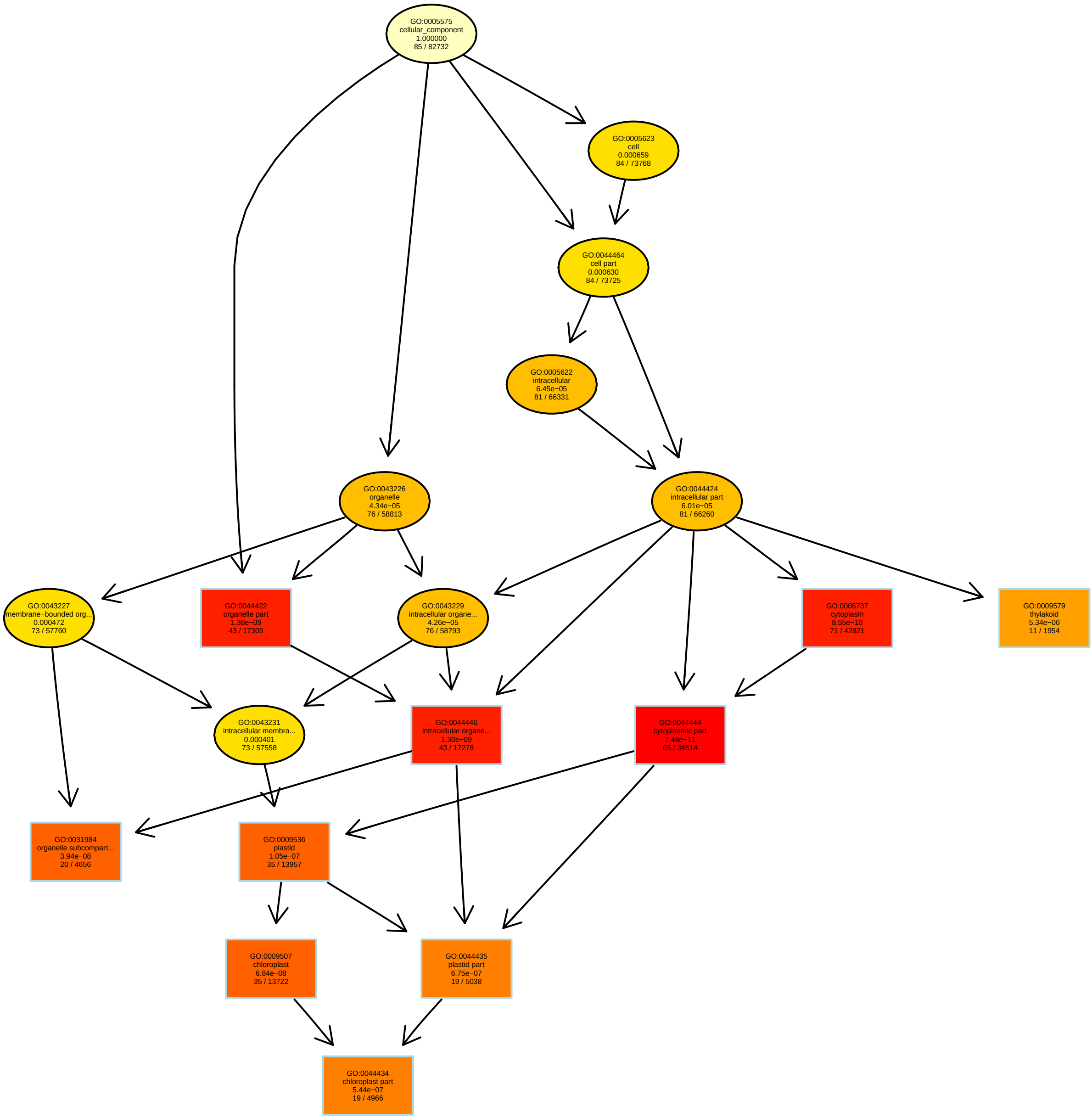
GO:0005575
cellular_component
1.000000
85 / 82732
GO:0005623
cell
0.000659
84 / 73768
GO:0044464
cell part
0.000630
84 / 73725
GO:0005622
intracellular
6.45e−05
81 / 66331
GO:0043226
organelle
4.34e−05
76 / 58813
GO:0044424
intracellular part
6.01e−05
81 / 66260
GO:0043227
membrane−bounded org...
0.000472
73 / 57760
GO:0044422
organelle part
1.38e−09
43 / 17309
GO:0043229
intracellular organe...
4.26e−05
76 / 58793
GO:0005737
cytoplasm
8.55e−10
71 / 42821
GO:0009579
thylakoid
5.34e−06
11 / 1954
GO:0043231
intracellular membra...
0.000401
73 / 57558
GO:0044446
intracellular organe...
1.30e−09
43 / 17278
GO:0044444
cytoplasmic part
7.48e−11
65 / 34514
GO:0031984
organelle subcompart...
3.94e−08
20 / 4656
GO:0009536
plastid
1.05e−07
35 / 13957
GO:0009507
chloroplast
6.84e−08
35 / 13722
GO:0044435
plastid part
6.75e−07
19 / 5038
GO:0044434
chloroplast part
5.44e−07
19 / 4966
