# Supplementary material for: Differential Alternative Splicing Genes and Isoform Regulation Networks of Rapeseed (Brassica napus L.) Infected with Sclerotinia sclerotiorum
Source: Genes (Basel). 2020 Jul 13;11(7):784. doi: 10.3390/genes11070784 (PMC7397149; doi:10.3390/genes11070784)
Supplement: Supplementary file 1 [file genes-11-00784-s001.zip › Fig. S2 Enriched GO terms of genes in the 85 WGCNA modules.zip!/Fig. S2/CytoNodemediumpurple2-go.pdf]

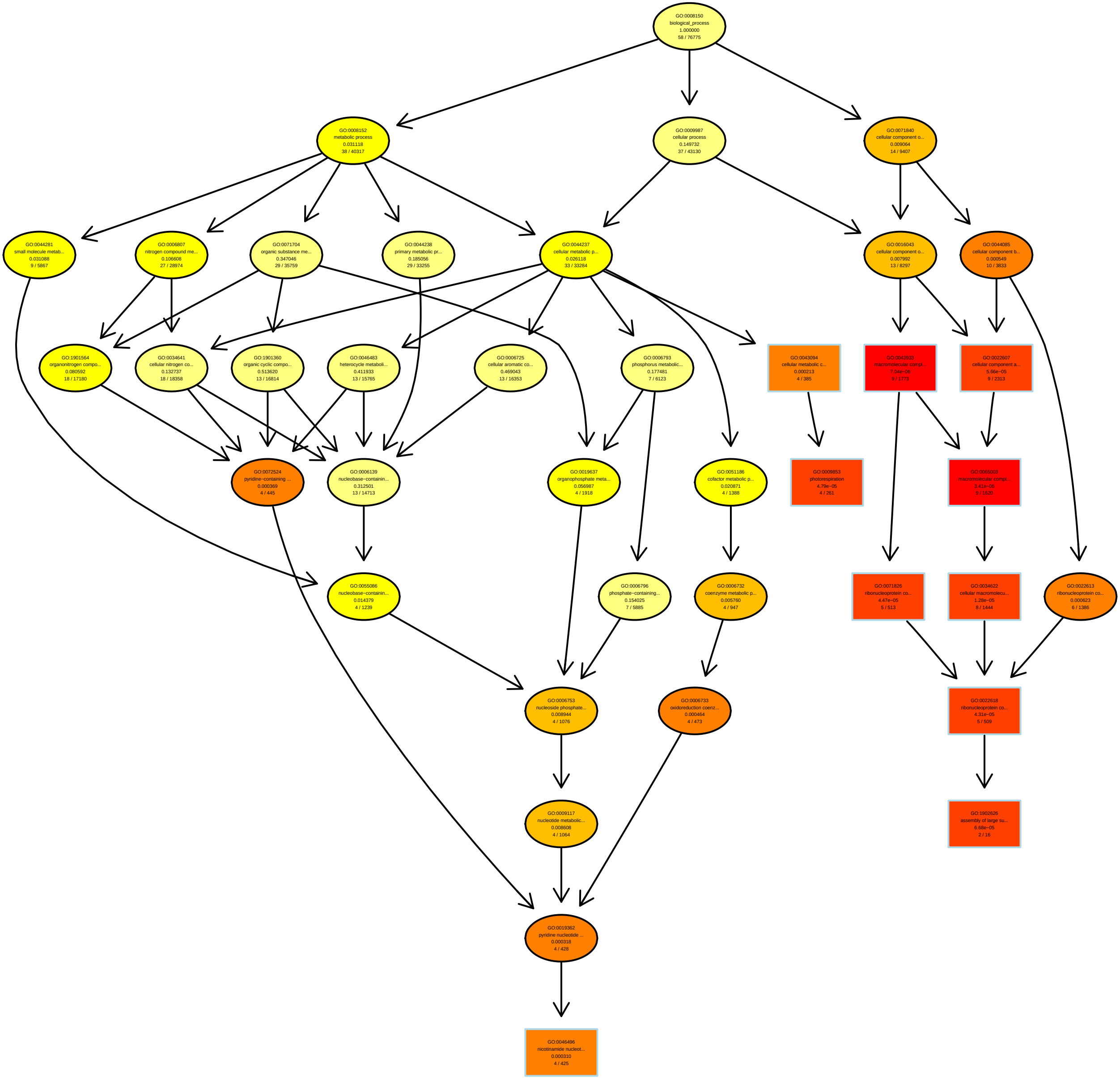

GO:0008150
biological_process
1.000000
58 / 76775
GO:0008152
metabolic process
0.031118
38 / 40317
GO:0009987
cellular process
0.149732
37 / 43130
GO:0071840
cellular component o...
0.009064
14 / 9407
GO:0044281
small molecule metab...
0.031088
9 / 5867
GO:0006807
nitrogen compound me...
0.106608
27 / 28974
GO:0071704
organic substance me...
0.347046
29 / 35759
GO:0044238
primary metabolic pr...
0.185056
29 / 33255
GO:0044237
cellular metabolic p...
0.026118
33 / 33284
GO:0016043
cellular component o...
0.007992
13 / 8297
GO:0044085
cellular component b...
0.000549
10 / 3833
GO:1901564
organonitrogen compo...
0.080592
18 / 17180
GO:0034641
cellular nitrogen co...
0.132737
18 / 18358
GO:1901360
organic cyclic compo...
0.513620
13 / 16814
GO:0046483
heterocycle metaboli...
0.411933
13 / 15765
GO:0006725
cellular aromatic co...
0.469043
13 / 16353
GO:0006793
phosphorus metabolic...
0.177481
7 / 6123
GO:0043094
cellular metabolic c...
0.000213
4 / 385
GO:0043933
macromolecular compl...
7.04e−06
9 / 1773
GO:0022607
cellular component a...
5.66e−05
9 / 2313
GO:0072524
pyridine−containing ...
0.000369
4 / 445
GO:0006139
nucleobase−containin...
0.312501
13 / 14713
GO:0019637
organophosphate meta...
0.056987
4 / 1918
GO:0051186
cofactor metabolic p...
0.020871
4 / 1388
GO:0009853
photorespiration
4.79e−05
4 / 261
GO:0065003
macromolecular compl...
3.41e−06
9 / 1520
GO:0055086
nucleobase−containin...
0.014379
4 / 1239
GO:0006796
phosphate−containing...
0.154025
7 / 5885
GO:0006732
coenzyme metabolic p...
0.005760
4 / 947
GO:0071826
ribonucleoprotein co...
4.47e−05
5 / 513
GO:0034622
cellular macromolecu...
1.28e−05
8 / 1444
GO:0022613
ribonucleoprotein co...
0.000623
6 / 1386
GO:0006753
nucleoside phosphate...
0.008944
4 / 1076
GO:0006733
oxidoreduction coenz...
0.000464
4 / 473
GO:0022618
ribonucleoprotein co...
4.31e−05
5 / 509
GO:0009117
nucleotide metabolic...
0.008608
4 / 1064
GO:1902626
assembly of large su...
6.68e−05
2 / 16
GO:0019362
pyridine nucleotide ...
0.000318
4 / 428
GO:0046496
nicotinamide nucleot...
0.000310
4 / 425

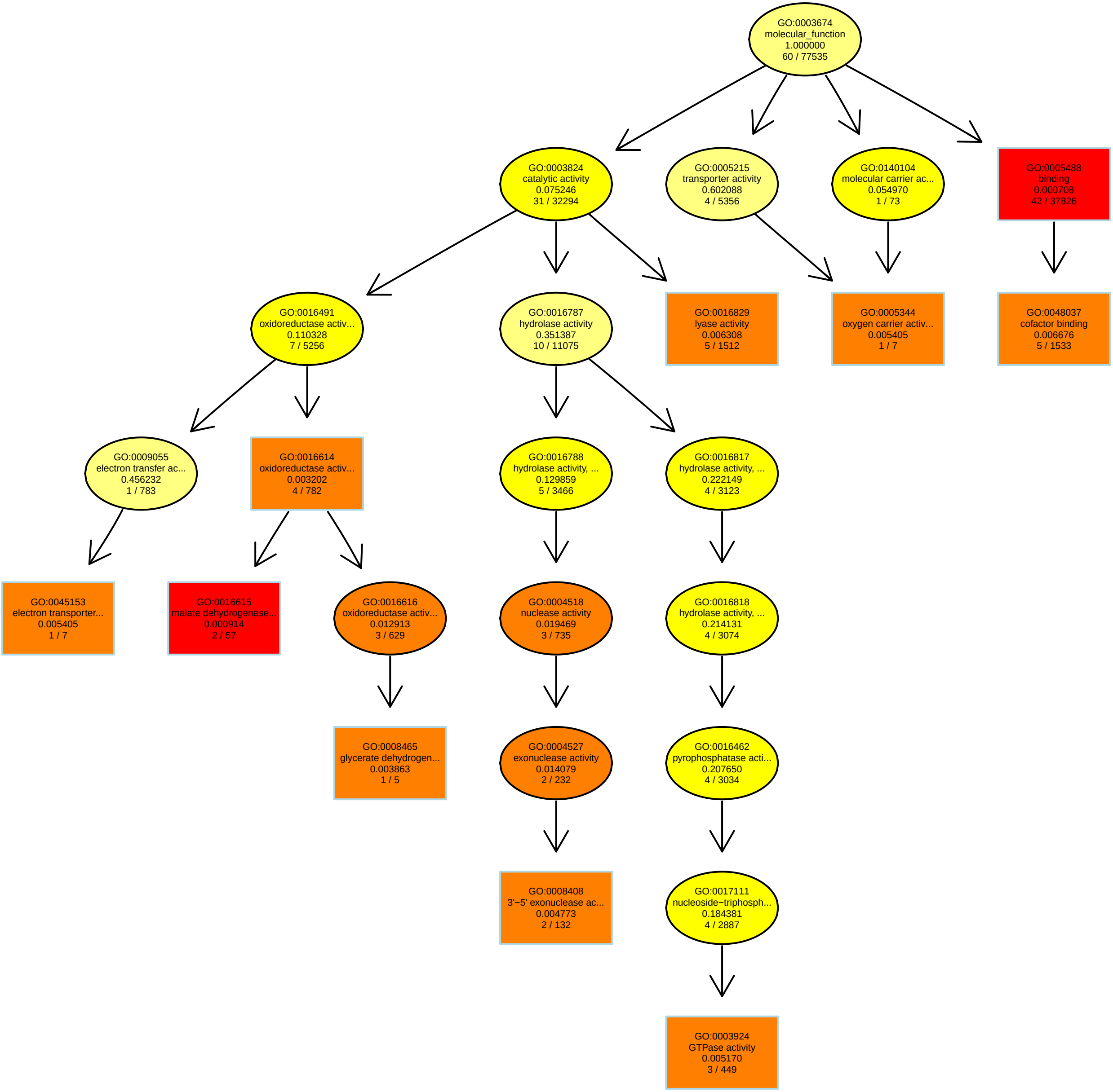
GO:0003674
molecular_function
1.000000
60 / 77535
GO:0003824
catalytic activity
0.075246
31 / 32294
GO:0005215
transporter activity
0.602088
4 / 5356
GO:0140104
molecular carrier ac...
0.054970
1 / 73
GO:0005488
binding
0.000708
42 / 37826
GO:0016491
oxidoreductase activ...
0.110328
7 / 5256
GO:0016787
hydrolase activity
0.351387
10 / 11075
GO:0016829
lyase activity
0.006308
5 / 1512
GO:0005344
oxygen carrier activ...
0.005405
1 / 7
GO:0048037
cofactor binding
0.006676
5 / 1533
GO:0009055
electron transfer ac...
0.456232
1 / 783
GO:0016614
oxidoreductase activ...
0.003202
4 / 782
GO:0016788
hydrolase activity, ...
0.129859
5 / 3466
GO:0016817
hydrolase activity, ...
0.222149
4 / 3123
GO:0045153
electron transporter...
0.005405
1 / 7
GO:0016615
malate dehydrogenase...
0.000914
2 / 57
GO:0016616
oxidoreductase activ...
0.012913
3 / 629
GO:0004518
nuclease activity
0.019469
3 / 735
GO:0016818
hydrolase activity, ...
0.214131
4 / 3074
GO:0008465
glycerate dehydrogen...
0.003863
1 / 5
GO:0004527
exonuclease activity
0.014079
2 / 232
GO:0016462
pyrophosphatase acti...
0.207650
4 / 3034
GO:0008408
3'−5' exonuclease ac...
0.004773
2 / 132
GO:0017111
nucleoside−triphosph...
0.184381
4 / 2887
GO:0003924
GTPase activity
0.005170
3 / 449

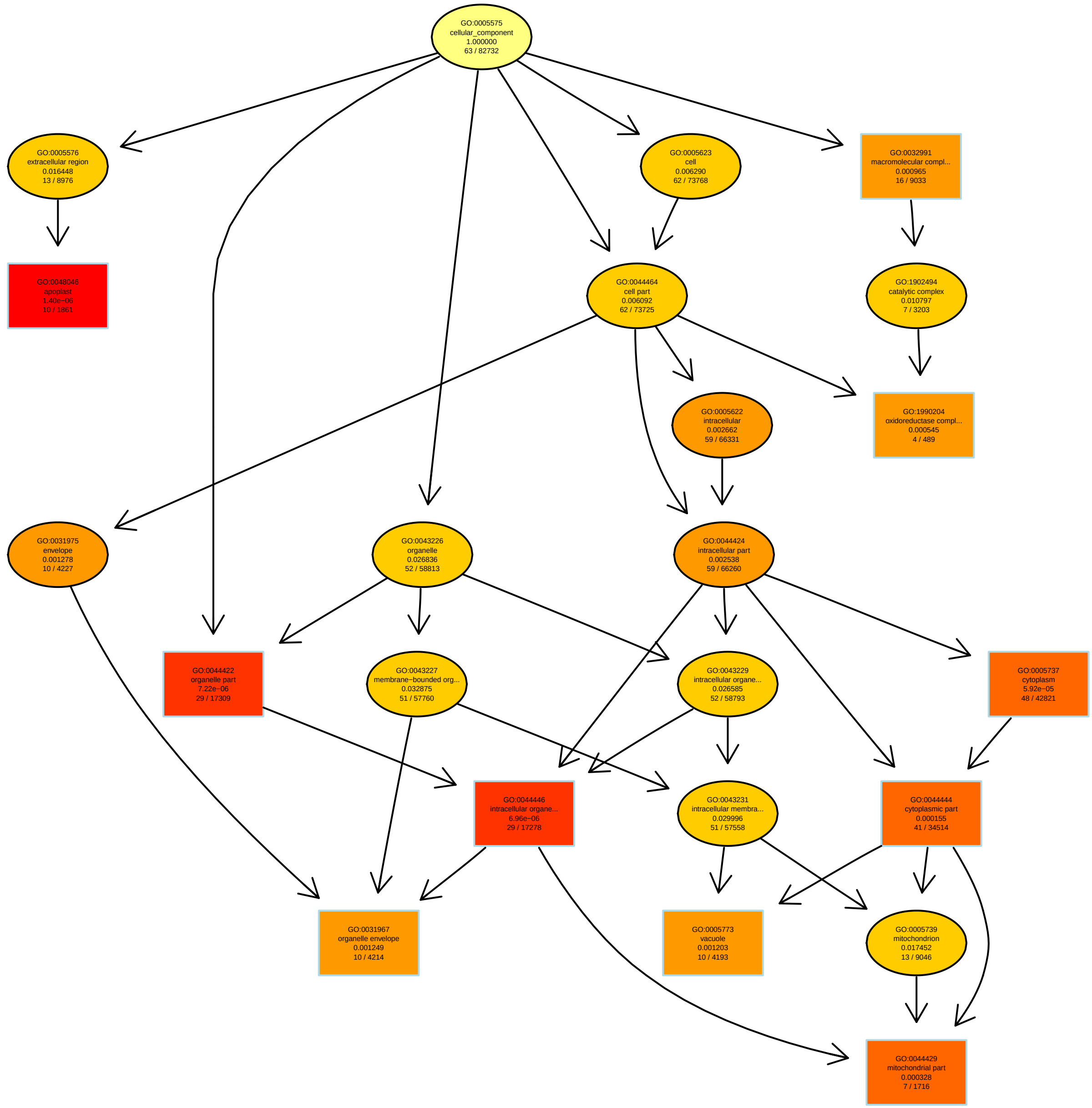
GO:0005575 cellular_component 1.000000 63 / 82732
GO:0005576 extracellular region 0.016448 13 / 8976
GO:0005623 cell 0.006290 62 / 73768
GO:0032991 macromolecular compl... 0.000965 16 / 9033
GO:0048046 apoplast 1.40e−06 10 / 1861
GO:0044464 cell part 0.006092 62 / 73725
GO:1902494 catalytic complex 0.010797 7 / 3203
GO:0005622 intracellular 0.002662 59 / 66331
GO:1990204 oxidoreductase compl... 0.000545 4 / 489
GO:0031975 envelope 0.001278 10 / 4227
GO:0043226 organelle 0.026836 52 / 58813
GO:0044424 intracellular part 0.002538 59 / 66260
GO:0044422 organelle part 7.22e−06 29 / 17309
GO:0043227 membrane−bounded org... 0.032875 51 / 57760
GO:0043229 intracellular organe... 0.026585 52 / 58793
GO:0005737 cytoplasm 5.92e−05 48 / 42821
GO:0044446 intracellular organe... 6.96e−06 29 / 17278
GO:0043231 intracellular membra... 0.029996 51 / 57558
GO:0044444 cytoplasmic part 0.000155 41 / 34514
GO:0031967 organelle envelope 0.001249 10 / 4214
GO:0005773 vacuole 0.001203 10 / 4193
GO:0005739 mitochondrion 0.017452 13 / 9046
GO:0044429 mitochondrial part 0.000328 7 / 1716
