# Supplementary material for: Differential Alternative Splicing Genes and Isoform Regulation Networks of Rapeseed (Brassica napus L.) Infected with Sclerotinia sclerotiorum
Source: Genes (Basel). 2020 Jul 13;11(7):784. doi: 10.3390/genes11070784 (PMC7397149; doi:10.3390/genes11070784)
Supplement: Supplementary file 1 [file genes-11-00784-s001.zip › Fig. S2 Enriched GO terms of genes in the 85 WGCNA modules.zip!/Fig. S2/CytoNodemediumpurple3-go.pdf]

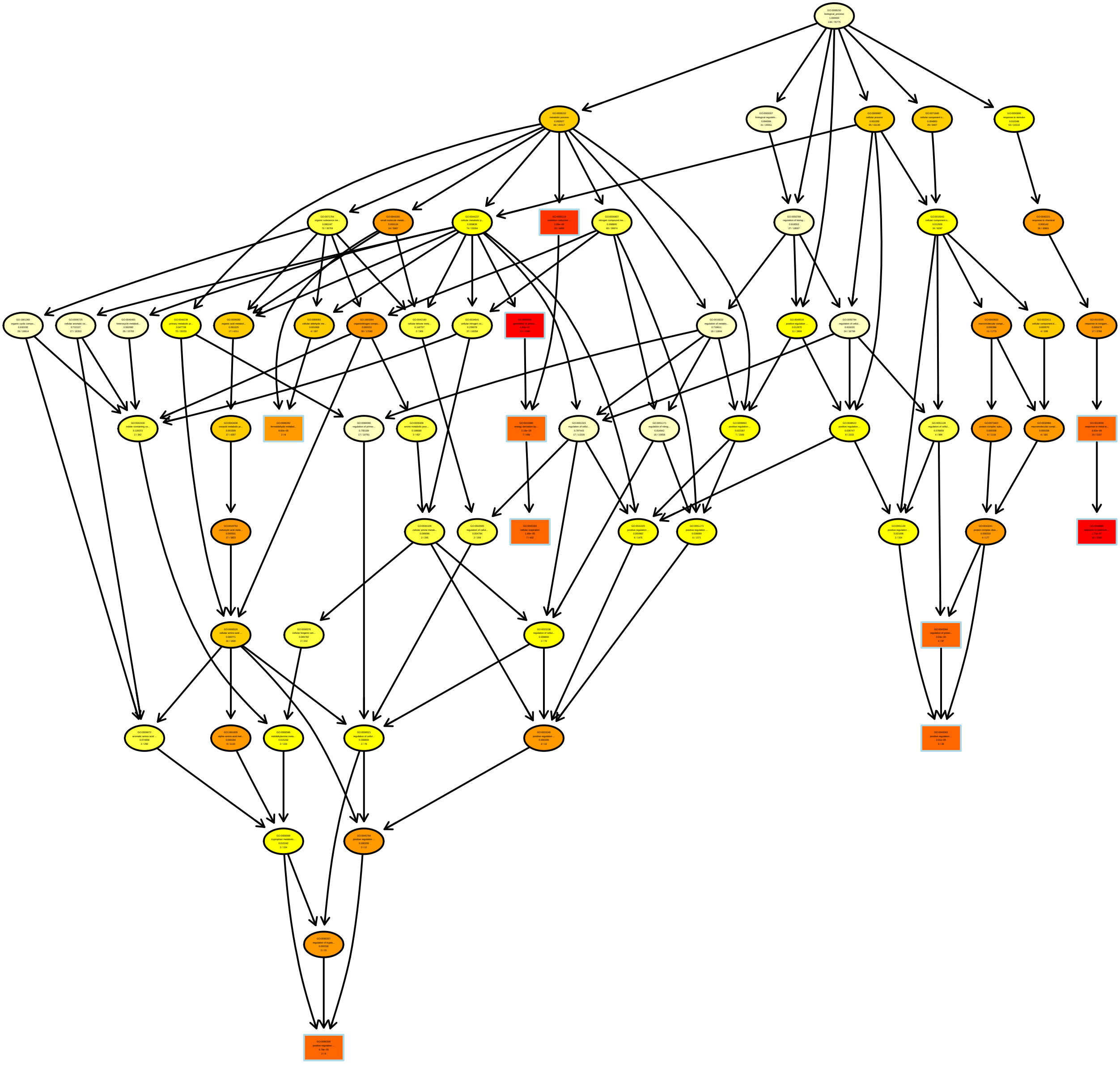

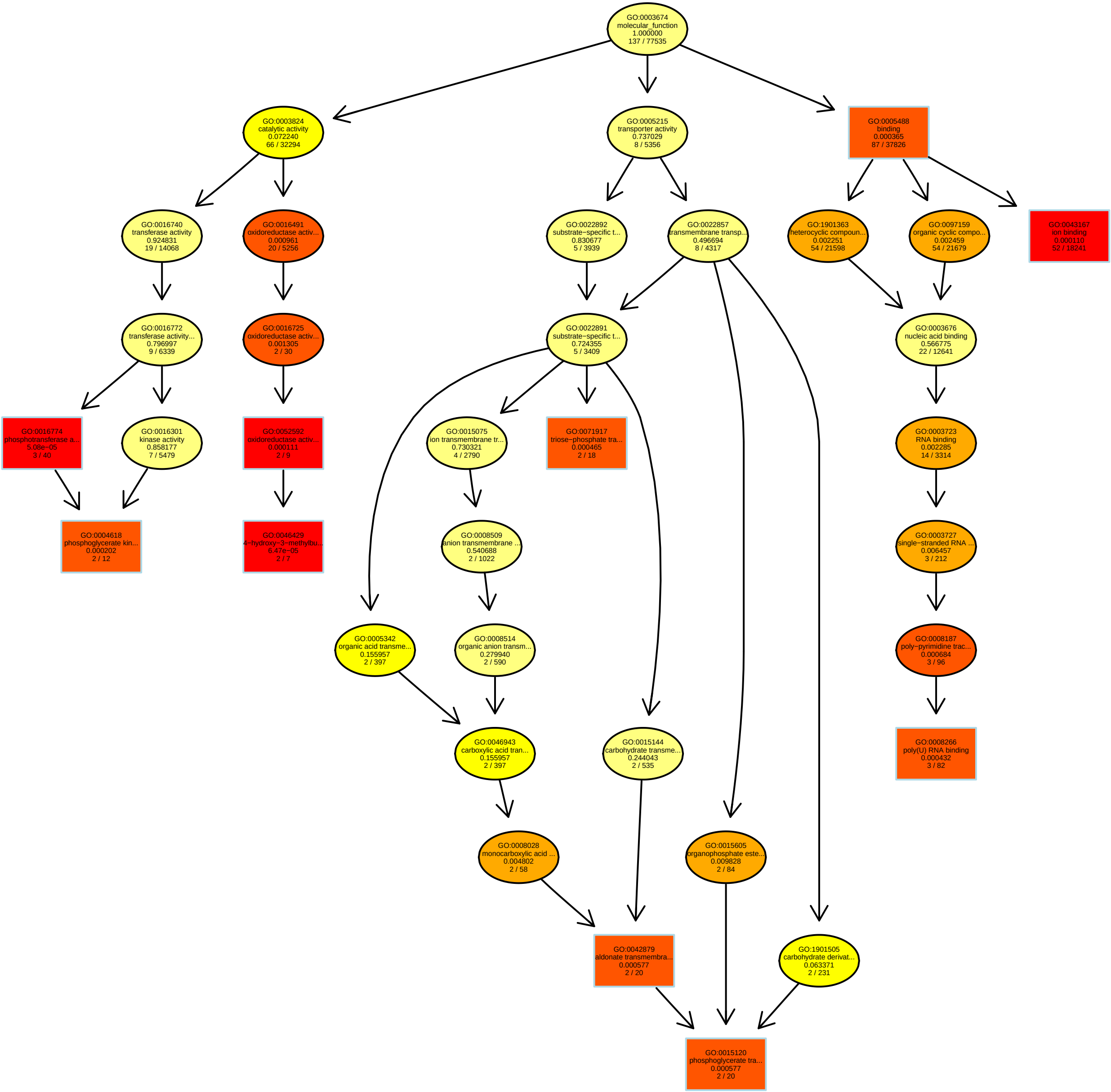

GO:0003674 molecular_function 1.000000 137 / 77535
GO:0003824 catalytic activity 0.072240 66 / 32294
GO:0005215 transporter activity 0.737029 8 / 5356
GO:0005488 binding 0.000365 87 / 37826
GO:0016740 transferase activity 0.924831 19 / 14068
GO:0016491 oxidoreductase activ... 0.000961 20 / 5256
GO:0022892 substrate−specific t... 0.830677 5 / 3939
GO:0022857 transmembrane transp... 0.496694 8 / 4317
GO:1901363 heterocyclic compoun... 0.002251 54 / 21598
GO:0097159 organic cyclic compo... 0.002459 54 / 21679
GO:0043167 ion binding 0.000110 52 / 18241
GO:0016772 transferase activity... 0.796997 9 / 6339
GO:0016725 oxidoreductase activ... 0.001305 2 / 30
GO:0022891 substrate−specific t... 0.724355 5 / 3409
GO:0003676 nucleic acid binding 0.566775 22 / 12641
GO:0016774 phosphotransferase a... 5.08e−05 3 / 40
GO:0016301 kinase activity 0.858177 7 / 5479
GO:0052592 oxidoreductase activ... 0.000111 2 / 9
GO:0015075 ion transmembrane tr... 0.730321 4 / 2790
GO:0071917 triose−phosphate tra... 0.000465 2 / 18
GO:0003723 RNA binding 0.002285 14 / 3314
GO:0004618 phosphoglycerate kin... 0.000202 2 / 12
GO:0046429 4−hydroxy−3−methylbu... 6.47e−05 2 / 7
GO:0008509 anion transmembrane ... 0.540688 2 / 1022
GO:0003727 single−stranded RNA ... 0.006457 3 / 212
GO:0005342 organic acid transme... 0.155957 2 / 397
GO:0008514 organic anion transm... 0.279940 2 / 590
GO:0008187 poly−pyrimidine trac... 0.000684 3 / 96
GO:0046943 carboxylic acid tran... 0.155957 2 / 397
GO:0015144 carbohydrate transme... 0.244043 2 / 535
GO:0008266 poly(U) RNA binding 0.000432 3 / 82
GO:0008028 monocarboxylic acid ... 0.004802 2 / 58
GO:0015605 organophosphate este... 0.009828 2 / 84
GO:0042879 aldonate transmembra... 0.000577 2 / 20
GO:1901505 carbohydrate derivat... 0.063371 2 / 231
GO:0015120 phosphoglycerate tra... 0.000577 2 / 20

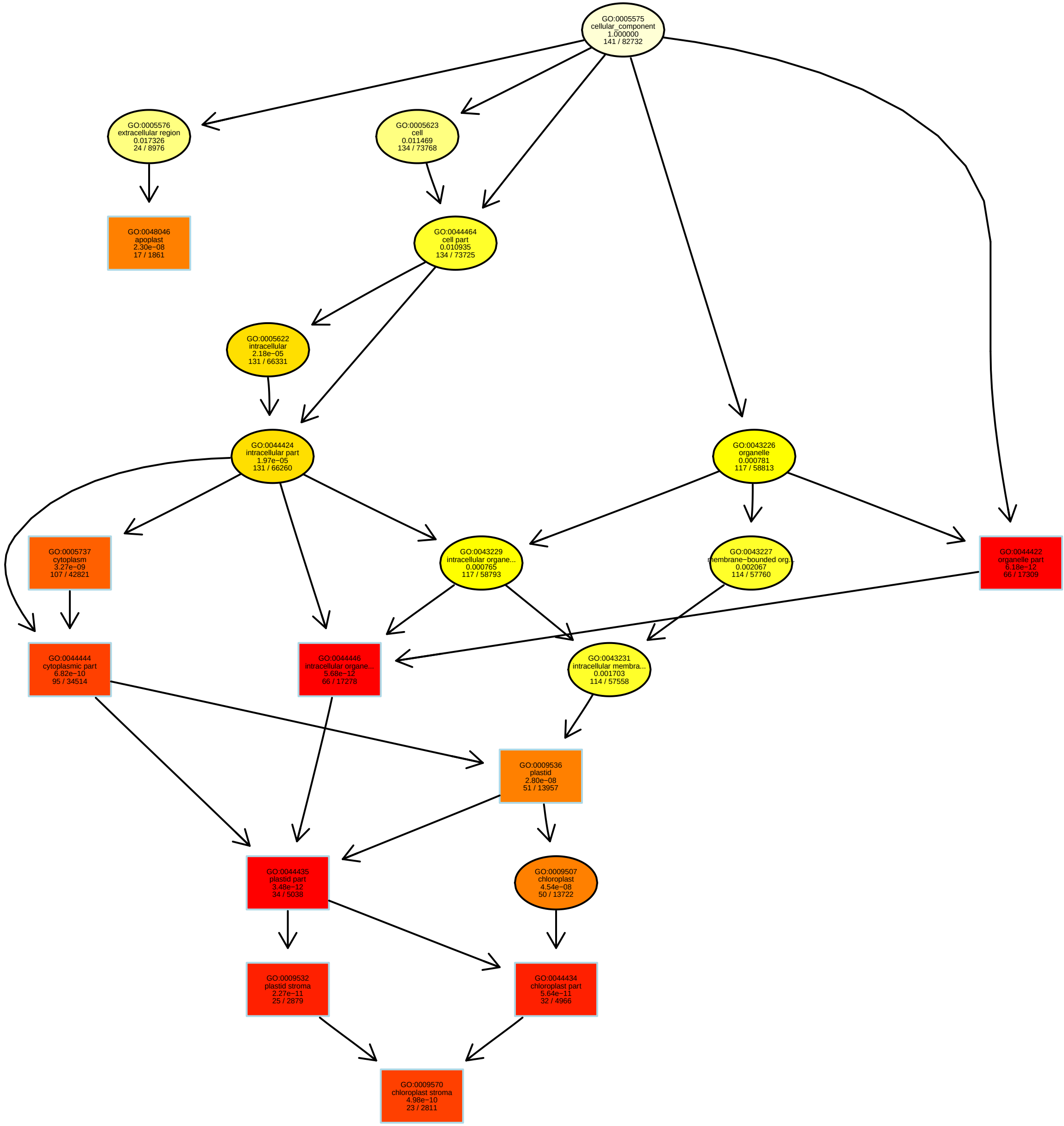
GO:0005575
cellular_component
1.000000
141 / 82732
GO:0005576
extracellular region
0.017326
24 / 8976
GO:0005623
cell
0.011469
134 / 73768
GO:0048046
apoplast
2.30e−08
17 / 1861
GO:0044464
cell part
0.010935
134 / 73725
GO:0005622
intracellular
2.18e−05
131 / 66331
GO:0044424
intracellular part
1.97e−05
131 / 66260
GO:0043226
organelle
0.000781
117 / 58813
GO:0005737
cytoplasm
3.27e−09
107 / 42821
GO:0043229
intracellular organe...
0.000765
117 / 58793
GO:0043227
membrane−bounded org...
0.002067
114 / 57760
GO:0044422
organelle part
6.18e−12
66 / 17309
GO:0044444
cytoplasmic part
6.82e−10
95 / 34514
GO:0044446
intracellular organe...
5.68e−12
66 / 17278
GO:0043231
intracellular membra...
0.001703
114 / 57558
GO:0009536
plastid
2.80e−08
51 / 13957
GO:0044435
plastid part
3.48e−12
34 / 5038
GO:0009507
chloroplast
4.54e−08
50 / 13722
GO:0009532
plastid stroma
2.27e−11
25 / 2879
GO:0044434
chloroplast part
5.64e−11
32 / 4966
GO:0009570
chloroplast stroma
4.98e−10
23 / 2811
