# Supplementary material for: Differential Alternative Splicing Genes and Isoform Regulation Networks of Rapeseed (Brassica napus L.) Infected with Sclerotinia sclerotiorum
Source: Genes (Basel). 2020 Jul 13;11(7):784. doi: 10.3390/genes11070784 (PMC7397149; doi:10.3390/genes11070784)
Supplement: Supplementary file 1 [file genes-11-00784-s001.zip › Fig. S2 Enriched GO terms of genes in the 85 WGCNA modules.zip!/Fig. S2/CytoNodemidnightblue-go.pdf]

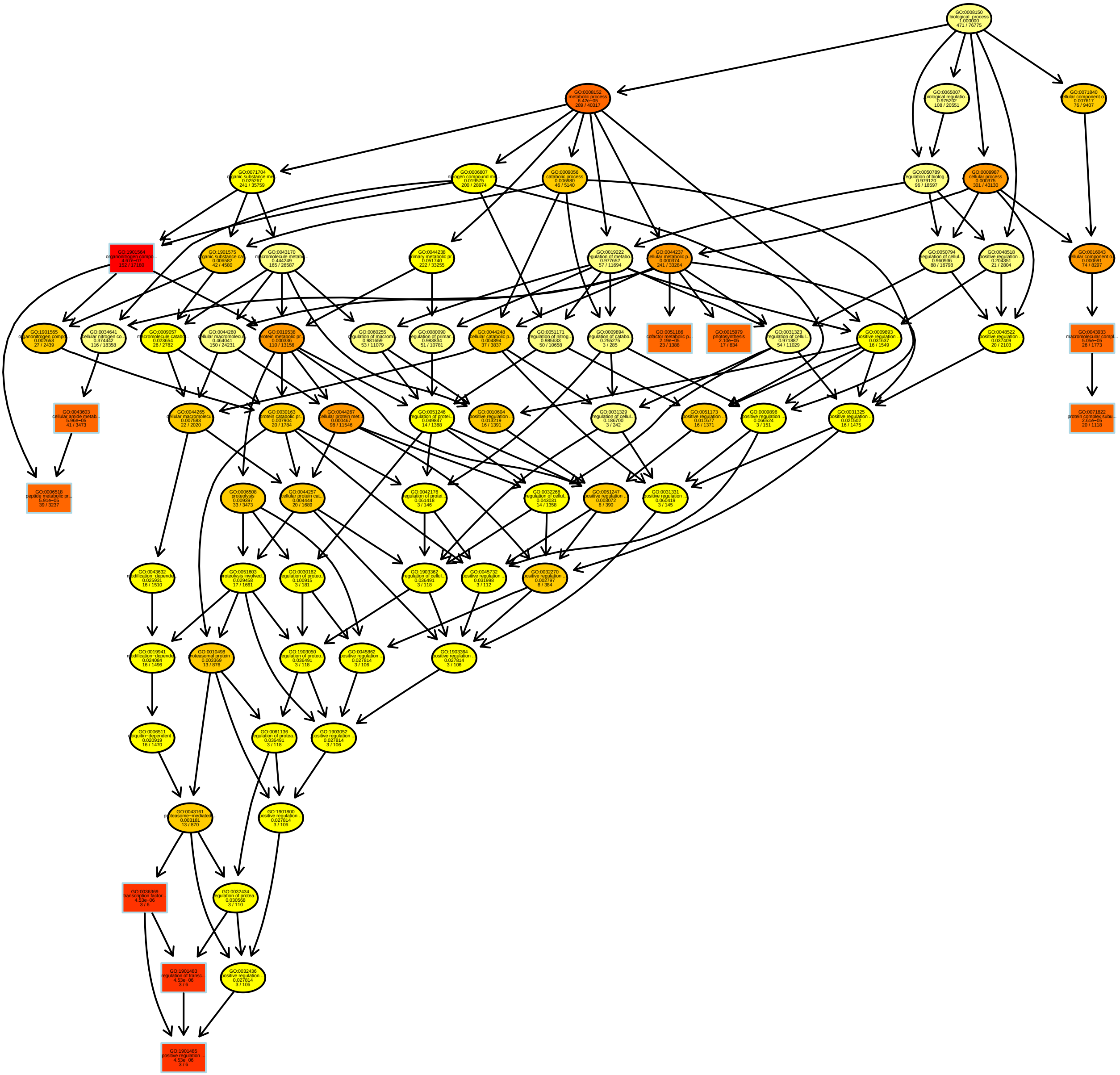

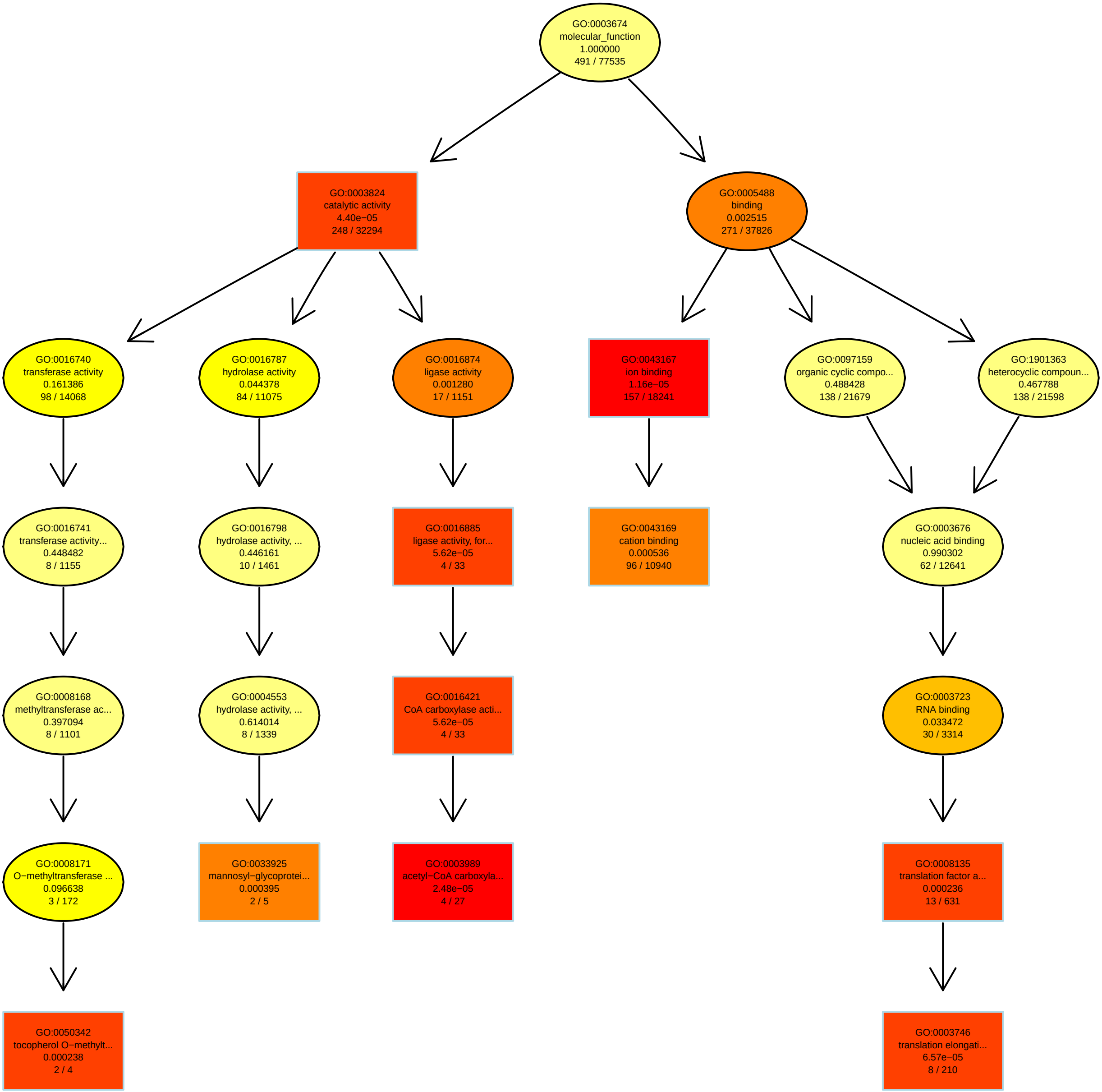
GO:0003674
molecular_function
1.000000
491 / 77535
GO:0003824
catalytic activity
4.40e−05
248 / 32294
GO:0005488
binding
0.002515
271 / 37826
GO:0016740
transferase activity
0.161386
98 / 14068
GO:0016787
hydrolase activity
0.044378
84 / 11075
GO:0016874
ligase activity
0.001280
17 / 1151
GO:0043167
ion binding
1.16e−05
157 / 18241
GO:0097159
organic cyclic compo...
0.488428
138 / 21679
GO:1901363
heterocyclic compoun...
0.467788
138 / 21598
GO:0016741
transferase activity...
0.448482
8 / 1155
GO:0016798
hydrolase activity, ...
0.446161
10 / 1461
GO:0016885
ligase activity, for...
5.62e−05
4 / 33
GO:0043169
cation binding
0.000536
96 / 10940
GO:0003676
nucleic acid binding
0.990302
62 / 12641
GO:0008168
methyltransferase ac...
0.397094
8 / 1101
GO:0004553
hydrolase activity, ...
0.614014
8 / 1339
GO:0016421
CoA carboxylase acti...
5.62e−05
4 / 33
GO:0003723
RNA binding
0.033472
30 / 3314
GO:0008171
O−methyltransferase ...
0.096638
3 / 172
GO:0033925
mannosyl−glycoprotei...
0.000395
2 / 5
GO:0003989
acetyl−CoA carboxyla...
2.48e−05
4 / 27
GO:0008135
translation factor a...
0.000236
13 / 631
GO:0050342
tocopherol O−methylt...
0.000238
2 / 4
GO:0003746
translation elongati...
6.57e−05
8 / 210

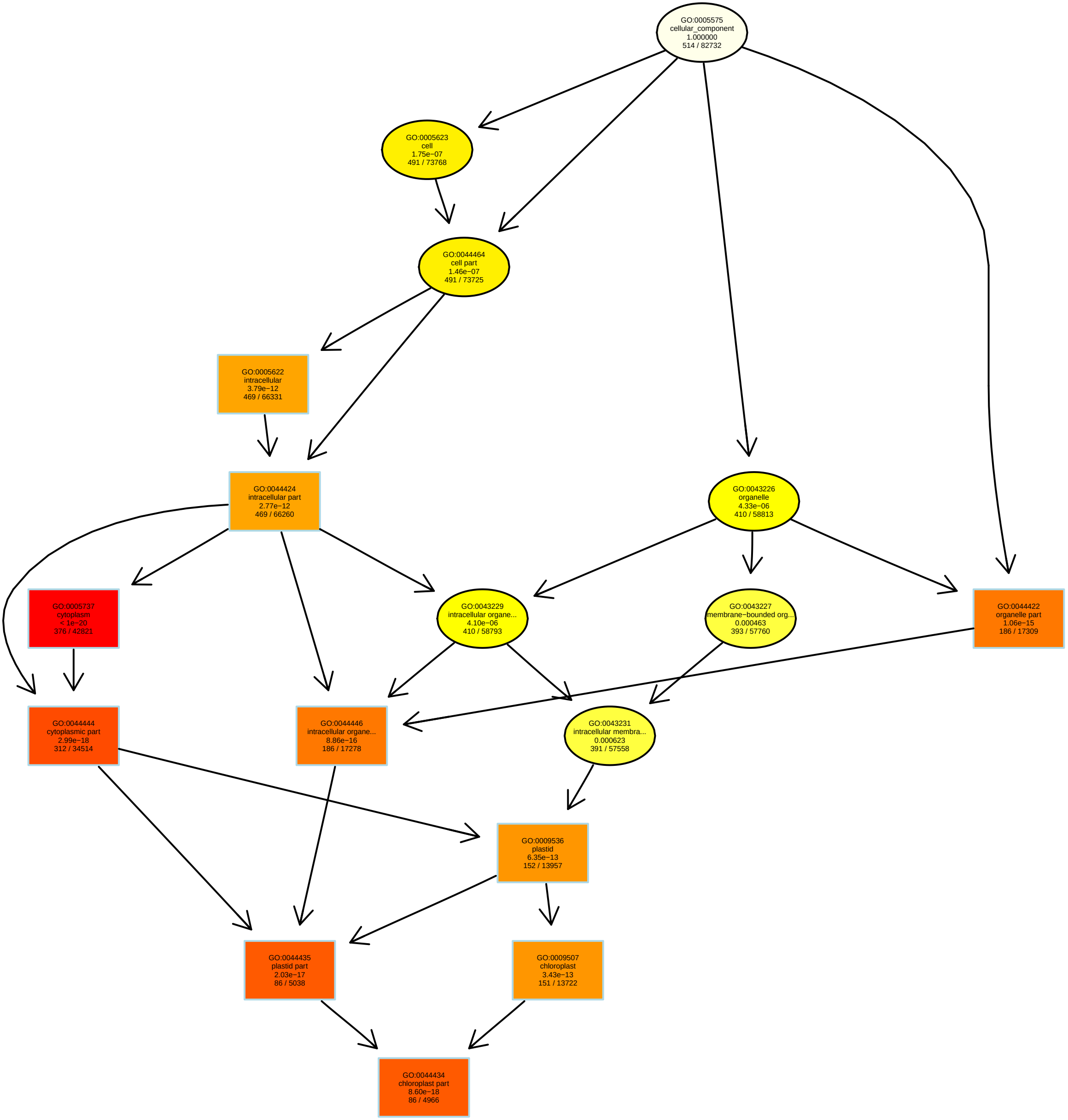
GO:0005575
cellular_component
1.000000
514 / 82732
GO:0005623
cell
1.75e−07
491 / 73768
GO:0044464
cell part
1.46e−07
491 / 73725
GO:0005622
intracellular
3.79e−12
469 / 66331
GO:0044424
intracellular part
2.77e−12
469 / 66260
GO:0043226
organelle
4.33e−06
410 / 58813
GO:0005737
cytoplasm
< 1e−20
376 / 42821
GO:0043229
intracellular organe...
4.10e−06
410 / 58793
GO:0043227
membrane−bounded org...
0.000463
393 / 57760
GO:0044422
organelle part
1.06e−15
186 / 17309
GO:0044444
cytoplasmic part
2.99e−18
312 / 34514
GO:0044446
intracellular organe...
8.86e−16
186 / 17278
GO:0043231
intracellular membra...
0.000623
391 / 57558
GO:0009536
plastid
6.35e−13
152 / 13957
GO:0044435
plastid part
2.03e−17
86 / 5038
GO:0009507
chloroplast
3.43e−13
151 / 13722
GO:0044434
chloroplast part
8.60e−18
86 / 4966
