# Supplementary material for: Differential Alternative Splicing Genes and Isoform Regulation Networks of Rapeseed (Brassica napus L.) Infected with Sclerotinia sclerotiorum
Source: Genes (Basel). 2020 Jul 13;11(7):784. doi: 10.3390/genes11070784 (PMC7397149; doi:10.3390/genes11070784)
Supplement: Supplementary file 1 [file genes-11-00784-s001.zip › Fig. S2 Enriched GO terms of genes in the 85 WGCNA modules.zip!/Fig. S2/CytoNodenavajowhite1-go.pdf]

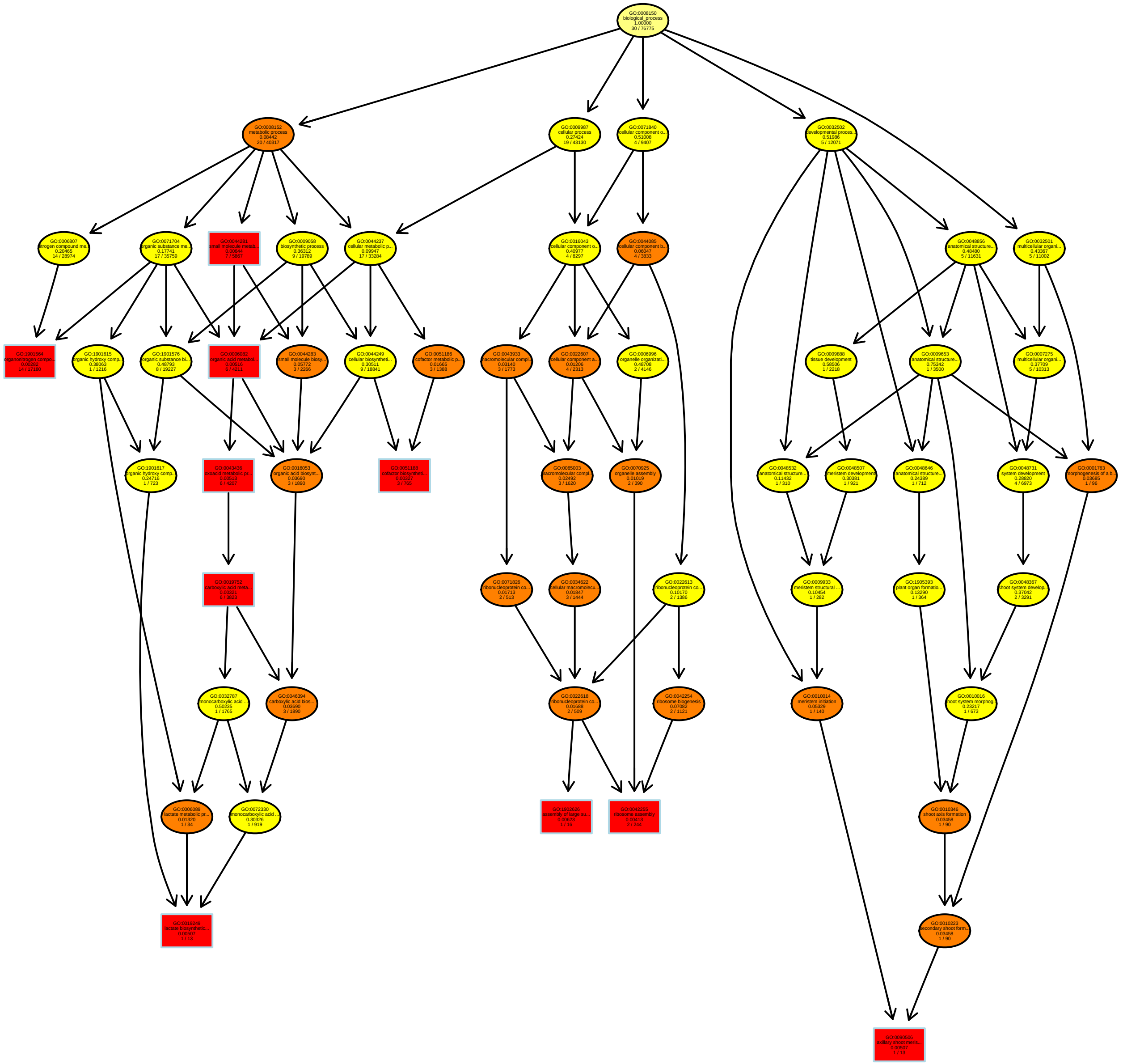

GO:0008150 biological_process 1.00000 30 / 76775
GO:0008152 metabolic process 0.08442 20 / 40317
GO:0009987 cellular process 0.27424 19 / 43130
GO:0071840 cellular component o... 0.51008 4 / 9407
GO:0032502 developmental proces... 0.51986 5 / 12071
GO:0006807 itrogen compound me... 0.20465 14 / 28974
GO:0071704 organic substance me... 0.17741 17 / 35759
GO:0044281 small molecule metab... 0.00644 7 / 5867
GO:0009058 biosynthetic process 0.36312 9 / 19789
GO:0044237 cellular metabolic p... 0.09947 17 / 33284
GO:0016043 cellular component o... 0.40977 4 / 8297
GO:0044085 cellular component b... 0.06047 4 / 3833
GO:0048856 anatomical structure... 0.48480 5 / 11631
GO:0032501 multicellular organi... 0.43367 5 / 11002
GO:1901564 organonitrogen compo... 0.00282 14 / 17180
GO:1901615 organic hydroxy comp... 0.38063 1 / 1216
GO:1901576 organic substance bi... 0.48793 8 / 19227
GO:0006082 organic acid metabol... 0.00516 6 / 4211
GO:0044283 small molecule biosy... 0.05772 3 / 2266
GO:0044249 cellular biosyntheti... 0.30511 9 / 18841
GO:0051186 cofactor metabolic p... 0.01665 3 / 1388
GO:0043933 macromolecular compl... 0.03140 3 / 1773
GO:0022607 cellular component a... 0.01206 4 / 2313
GO:0006996 organelle organizati... 0.48708 2 / 4146
GO:0009888 tissue development 0.58506 1 / 2218
GO:0009653 anatomical structure... 0.75342 1 / 3500
GO:0007275 multicellular organi... 0.37709 5 / 10313
GO:1901617 organic hydroxy comp... 0.24716 1 / 723
GO:0043436 oxoacid metabolic pr... 0.00513 6 / 4207
GO:0016053 organic acid biosynt... 0.03690 3 / 1890
GO:0051188 cofactor biosyntheti... 0.00327 3 / 765
GO:0065003 macromolecular compl... 0.02492 3 / 1620
GO:0070925 organelle assembly 0.01019 2 / 390
GO:0048532 anatomical structure... 0.11432 1 / 310
GO:0048507 meristem development 0.30381 1 / 921
GO:0048646 anatomical structure... 0.24389 1 / 712
GO:0048731 system development 0.28820 4 / 6973
GO:0001763 morphogenesis of a b... 0.03685 1 / 96
GO:0019752 carboxylic acid meta... 0.00321 6 / 3823
GO:0071826 ribonucleoprotein co... 0.01713 2 / 513
GO:0034622 cellular macromolecu... 0.01847 3 / 1444
GO:0022613 ribonucleoprotein co... 0.10170 2 / 1386
GO:0009933 meristem structural ... 0.10454 1 / 282
GO:1905393 plant organ formatio... 0.13290 1 / 364
GO:0048367 shoot system develop... 0.37042 2 / 3291
GO:0032787 monocarboxylic acid ... 0.50235 1 / 1765
GO:0046394 carboxylic acid bios... 0.03690 3 / 1890
GO:0022618 ribonucleoprotein co... 0.01688 2 / 509
GO:0042254 ribosome biogenesis 0.07082 2 / 1121
GO:0010014 meristem initiation 0.05329 1 / 140
GO:0010016 shoot system morphog... 0.23217 1 / 673
GO:0006089 lactate metabolic pr... 0.01320 1 / 34
GO:0072330 monocarboxylic acid ... 0.30326 1 / 919
GO:1902626 assembly of large su... 0.00623 1 / 16
GO:0042255 ribosome assembly 0.00413 2 / 244
GO:0010346 shoot axis formation 0.03458 1 / 90
GO:0019249 lactate biosynthetic... 0.00507 1 / 13
GO:0010223 secondary shoot form... 0.03458 1 / 90
GO:0090506 axillary shoot meris... 0.00507 1 / 13

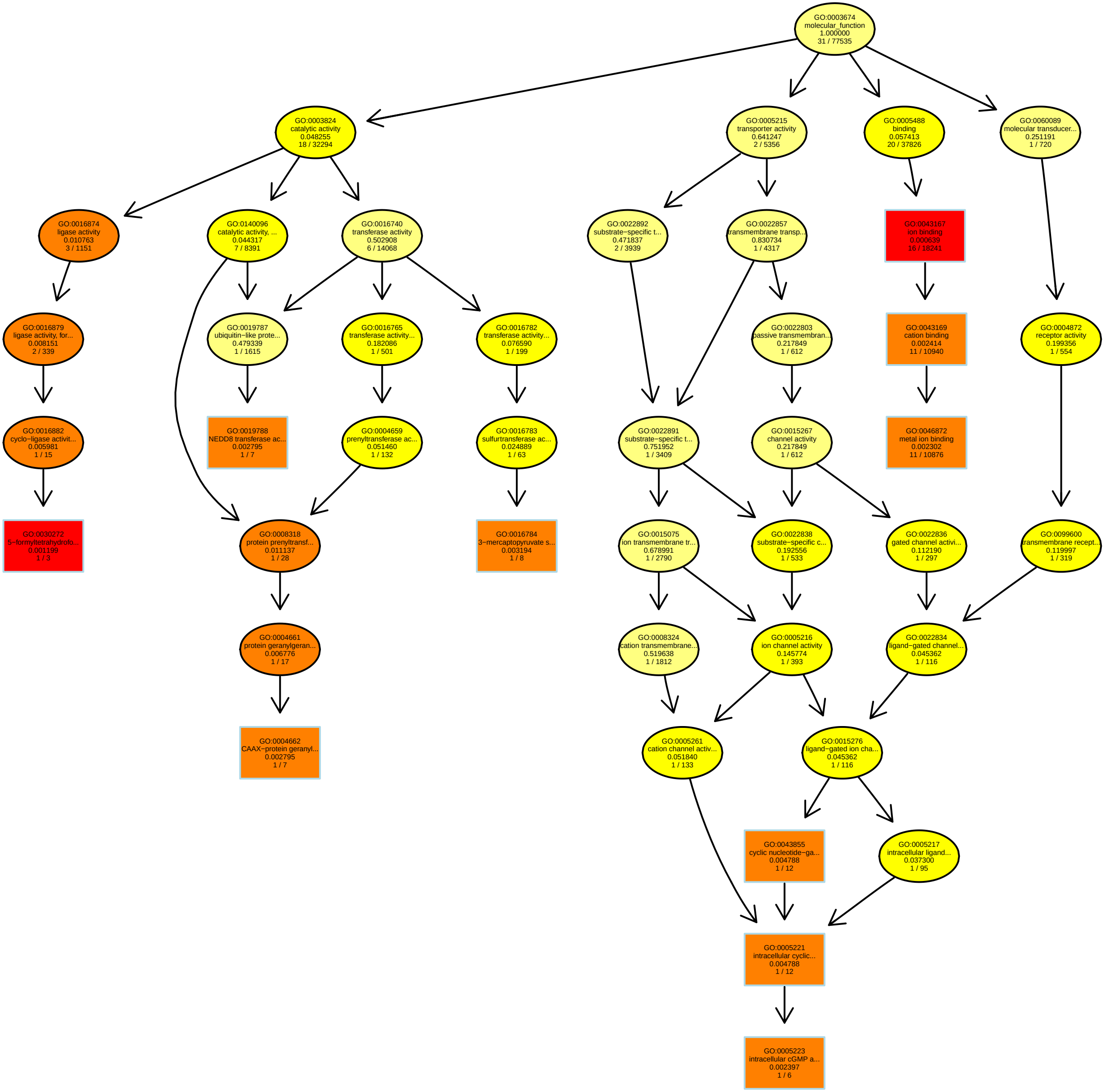
GO:0003674
molecular_function
1.000000
31 / 77535
GO:0003824
catalytic activity
0.048255
18 / 32294
GO:0005215
transporter activity
0.641247
2 / 5356
GO:0005488
binding
0.057413
20 / 37826
GO:0060089
molecular transducer...
0.251191
1 / 720
GO:0016874
ligase activity
0.010763
3 / 1151
GO:0140096
catalytic activity, ...
0.044317
7 / 8391
GO:0016740
transferase activity
0.502908
6 / 14068
GO:0022892
substrate−specific t...
0.471837
2 / 3939
GO:0022857
transmembrane transp...
0.830734
1 / 4317
GO:0043167
ion binding
0.000639
16 / 18241
GO:0016879
ligase activity, for...
0.008151
2 / 339
GO:0019787
ubiquitin−like prote...
0.479339
1 / 1615
GO:0016765
transferase activity...
0.182086
1 / 501
GO:0016782
transferase activity...
0.076590
1 / 199
GO:0022803
passive transmembran...
0.217849
1 / 612
GO:0043169
cation binding
0.002414
11 / 10940
GO:0004872
receptor activity
0.199356
1 / 554
GO:0016882
cyclo−ligase activit...
0.005981
1 / 15
GO:0019788
NEDD8 transferase ac...
0.002795
1 / 7
GO:0004659
prenyltransferase ac...
0.051460
1 / 132
GO:0016783
sulfurtransferase ac...
0.024889
1 / 63
GO:0022891
substrate−specific t...
0.751952
1 / 3409
GO:0015267
channel activity
0.217849
1 / 612
GO:0046872
metal ion binding
0.002302
11 / 10876
GO:0030272
5−formyltetrahydrofo...
0.001199
1 / 3
GO:0008318
protein prenyltransf...
0.011137
1 / 28
GO:0016784
3−mercaptopyruvate s...
0.003194
1 / 8
GO:0015075
ion transmembrane tr...
0.678991
1 / 2790
GO:0022838
substrate−specific c...
0.192556
1 / 533
GO:0022836
gated channel activi...
0.112190
1 / 297
GO:0099600
transmembrane recept...
0.119997
1 / 319
GO:0004661
protein geranylgeran...
0.006776
1 / 17
GO:0008324
cation transmembrane...
0.519638
1 / 1812
GO:0005216
ion channel activity
0.145774
1 / 393
GO:0022834
ligand−gated channel...
0.045362
1 / 116
GO:0004662
CAAX−protein geranyl...
0.002795
1 / 7
GO:0005261
cation channel activ...
0.051840
1 / 133
GO:0015276
ligand−gated ion cha...
0.045362
1 / 116
GO:0043855
cyclic nucleotide−ga...
0.004788
1 / 12
GO:0005217
intracellular ligand...
0.037300
1 / 95
GO:0005221
intracellular cyclic...
0.004788
1 / 12
GO:0005223
intracellular cGMP a...
0.002397
1 / 6

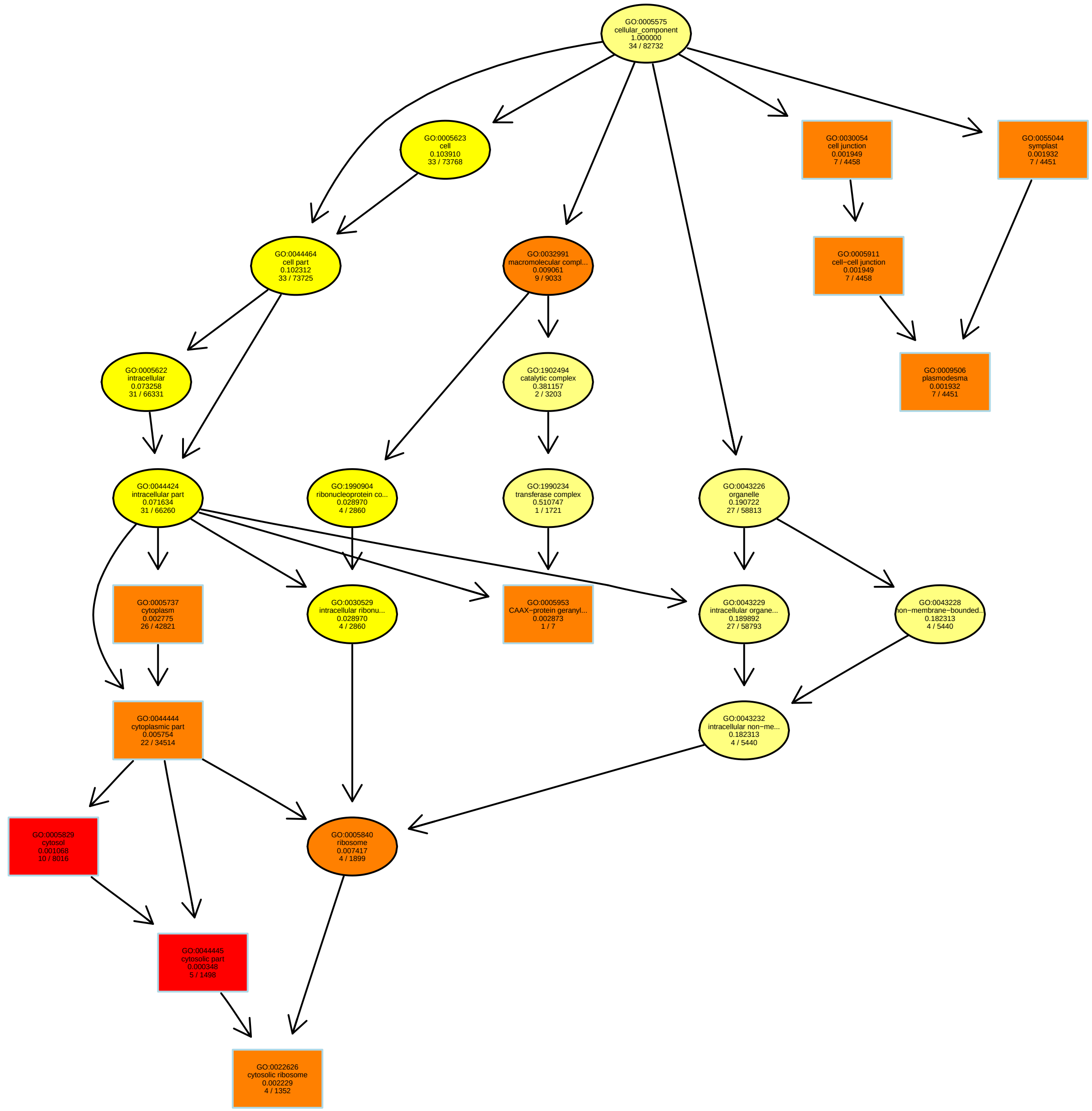
GO:0005575
cellular_component
1.000000
34 / 82732
GO:0005623
cell
0.103910
33 / 73768
GO:0030054
cell junction
0.001949
7 / 4458
GO:0055044
symplast
0.001932
7 / 4451
GO:0044464
cell part
0.102312
33 / 73725
GO:0032991
macromolecular compl...
0.009061
9 / 9033
GO:0005911
cell−cell junction
0.001949
7 / 4458
GO:0005622
intracellular
0.073258
31 / 66331
GO:1902494
catalytic complex
0.381157
2 / 3203
GO:0009506
plasmodesma
0.001932
7 / 4451
GO:0044424
intracellular part
0.071634
31 / 66260
GO:1990904
ribonucleoprotein co...
0.028970
4 / 2860
GO:1990234
transferase complex
0.510747
1 / 1721
GO:0043226
organelle
0.190722
27 / 58813
GO:0005737
cytoplasm
0.002775
26 / 42821
GO:0030529
intracellular ribonu...
0.028970
4 / 2860
GO:0005953
CAAX−protein geranyl...
0.002873
1 / 7
GO:0043229
intracellular organe...
0.189892
27 / 58793
GO:0043228
non−membrane−bounded...
0.182313
4 / 5440
GO:0044444
cytoplasmic part
0.005754
22 / 34514
GO:0043232
intracellular non−me...
0.182313
4 / 5440
GO:0005829
cytosol
0.001068
10 / 8016
GO:0005840
ribosome
0.007417
4 / 1899
GO:0044445
cytosolic part
0.000348
5 / 1498
GO:0022626
cytosolic ribosome
0.002229
4 / 1352
