# Supplementary material for: Differential Alternative Splicing Genes and Isoform Regulation Networks of Rapeseed (Brassica napus L.) Infected with Sclerotinia sclerotiorum
Source: Genes (Basel). 2020 Jul 13;11(7):784. doi: 10.3390/genes11070784 (PMC7397149; doi:10.3390/genes11070784)
Supplement: Supplementary file 1 [file genes-11-00784-s001.zip › Fig. S2 Enriched GO terms of genes in the 85 WGCNA modules.zip!/Fig. S2/CytoNodenavajowhite2-go.pdf]

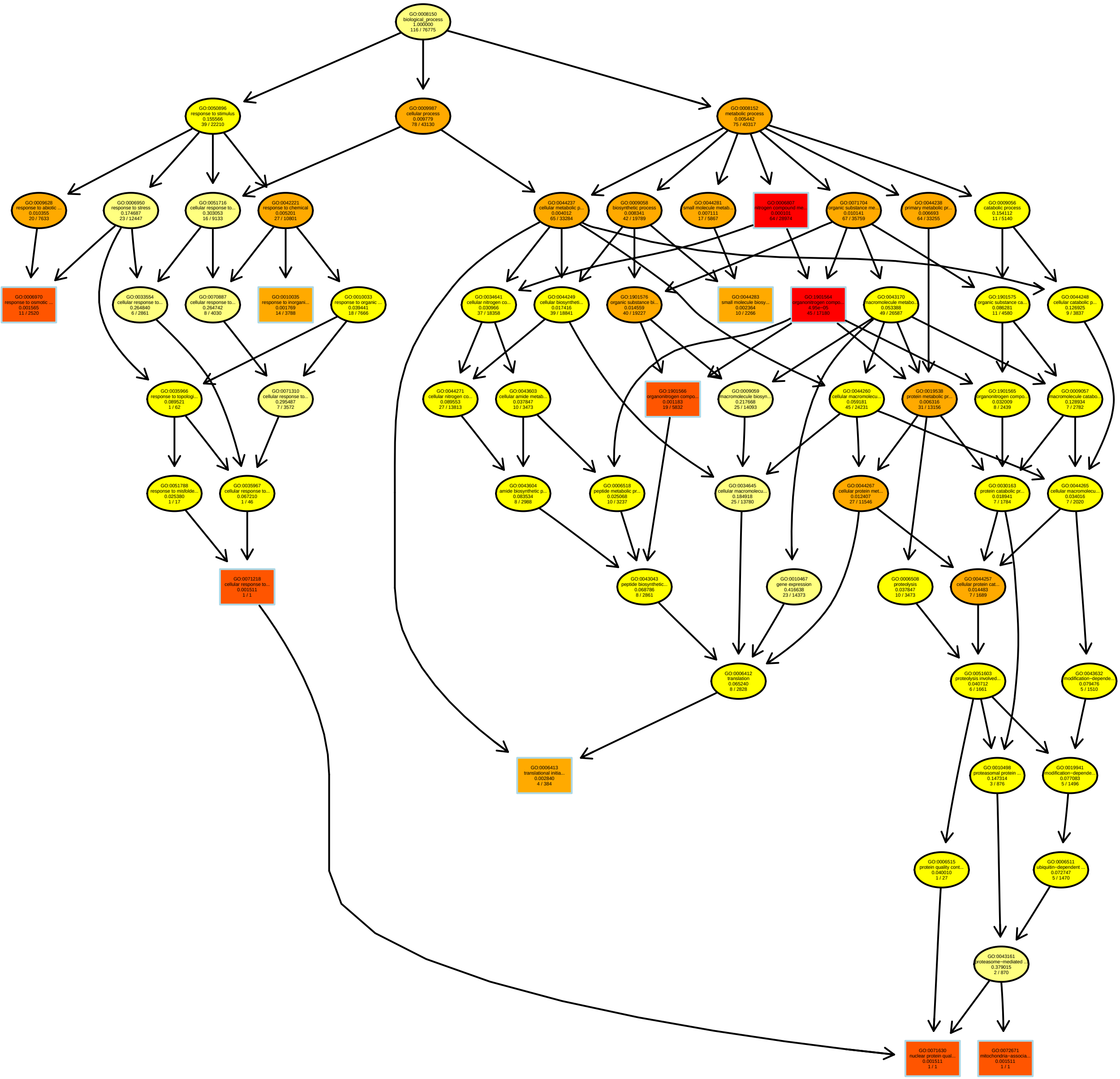
GO:0008150 biological_process 1.000000 116 / 76775
GO:0050896 response to stimulus 0.155566 39 / 22210
GO:0009987 cellular process 0.009779 78 / 43130
GO:0008152 metabolic process 0.005442 75 / 40317
GO:0009628 response to abiotic ... 0.010355 20 / 7633
GO:0006950 response to stress 0.174687 23 / 12447
GO:0051716 cellular response to... 0.303053 16 / 9133
GO:0042221 response to chemical 0.005201 27 / 10801
GO:0044237 cellular metabolic p... 0.004012 65 / 33284
GO:0009058 biosynthetic process 0.008341 42 / 19789
GO:0044281 small molecule metab... 0.007111 17 / 5867
GO:0006807 nitrogen compound me... 0.000101 64 / 28974
GO:0071704 organic substance me... 0.010141 67 / 35759
GO:0044238 primary metabolic pr... 0.006693 64 / 33255
GO:0009056 catabolic process 0.154112 11 / 5140
GO:0006970 response to osmotic ... 0.001565 11 / 2520
GO:0033554 cellular response to... 0.264840 6 / 2861
GO:0070887 cellular response to... 0.264742 8 / 4030
GO:0010035 response to inorgani... 0.001769 14 / 3788
GO:0010033 response to organic ... 0.039441 18 / 7666
GO:0034641 cellular nitrogen co... 0.030966 37 / 18358
GO:0044249 cellular biosyntheti... 0.017416 39 / 18841
GO:1901576 organic substance bi... 0.014559 40 / 19227
GO:0044283 small molecule biosy... 0.002364 10 / 2266
GO:1901564 organonitrogen compo... 4.65e-05 45 / 17180
GO:0043170 macromolecule metabo... 0.053388 49 / 26587
GO:1901575 organic substance ca... 0.086281 11 / 4580
GO:0044248 cellular catabolic p... 0.126925 9 / 3837
GO:0035966 response to topologi... 0.089521 1 / 62
GO:0071310 cellular response to... 0.295487 7 / 3572
GO:0044271 cellular nitrogen co... 0.089553 27 / 13813
GO:0043603 cellular amide metab... 0.037847 10 / 3473
GO:1901566 organonitrogen compo... 0.001183 19 / 5832
GO:0009059 macromolecule biosyn... 0.217668 25 / 14093
GO:0044260 cellular macromolecu... 0.059181 45 / 24231
GO:0019538 protein metabolic pr... 0.006316 31 / 13156
GO:1901565 organonitrogen compo... 0.032009 8 / 2439
GO:0009057 macromolecule catabo... 0.128934 7 / 2782
GO:0051788 response to misfolde... 0.025380 1 / 17
GO:0035967 cellular response to... 0.067210 1 / 46
GO:0043604 amide biosynthetic p... 0.083534 8 / 2988
GO:0006518 peptide metabolic pr... 0.025068 10 / 3237
GO:0034645 cellular macromolecu... 0.184918 25 / 13780
GO:0044267 cellular protein met... 0.012407 27 / 11546
GO:0030163 protein catabolic pr... 0.018941 7 / 1784
GO:0044265 cellular macromolecu... 0.034016 7 / 2020
GO:0071218 cellular response to... 0.001511 1 / 1
GO:0043043 peptide biosynthetic... 0.068786 8 / 2861
GO:0010467 gene expression 0.416638 23 / 14373
GO:0006508 proteolysis 0.037847 10 / 3473
GO:0044257 cellular protein cat... 0.014483 7 / 1689
GO:0006412 translation 0.065240 8 / 2828
GO:0051603 proteolysis involved... 0.040712 6 / 1661
GO:0043632 modification-depende... 0.079476 5 / 1510
GO:0006413 translational initia... 0.002840 4 / 384
GO:0010498 proteasomal protein ... 0.147314 3 / 876
GO:0019941 modification-depende... 0.077083 5 / 1496
GO:0006515 protein quality cont... 0.040010 1 / 27
GO:0006511 ubiquitin-dependent ... 0.072747 5 / 1470
GO:0043161 proteasome-mediated ... 0.379015 2 / 870
GO:0071630 nuclear protein qual... 0.001511 1 / 1
GO:0072671 mitochondria-associa... 0.001511 1 / 1

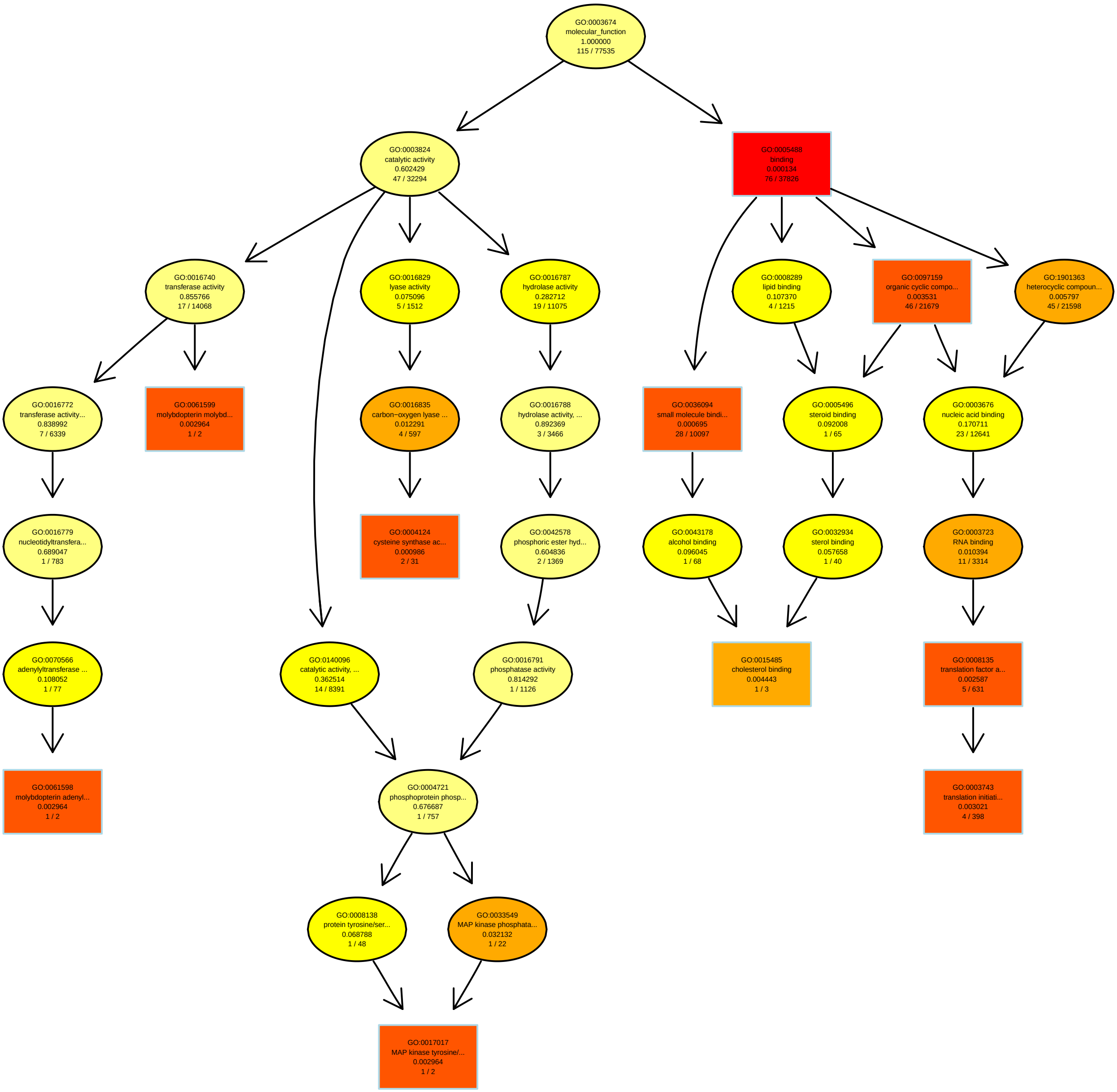
GO:0003674 molecular_function 1.000000 115 / 77535
GO:0003824 catalytic activity 0.602429 47 / 32294
GO:0005488 binding 0.000134 76 / 37826
GO:0016740 transferase activity 0.855766 17 / 14068
GO:0016829 lyase activity 0.075096 5 / 1512
GO:0016787 hydrolase activity 0.282712 19 / 11075
GO:0008289 lipid binding 0.107370 4 / 1215
GO:0097159 organic cyclic compo... 0.003531 46 / 21679
GO:1901363 heterocyclic compoun... 0.005797 45 / 21598
GO:0016772 transferase activity... 0.838992 7 / 6339
GO:0061599 molybdopterin molybd... 0.002964 1 / 2
GO:0016835 carbon−oxygen lyase ... 0.012291 4 / 597
GO:0016788 hydrolase activity, ... 0.892369 3 / 3466
GO:0036094 small molecule bindi... 0.000695 28 / 10097
GO:0005496 steroid binding 0.092008 1 / 65
GO:0003676 nucleic acid binding 0.170711 23 / 12641
GO:0016779 nucleotidyltransfera... 0.689047 1 / 783
GO:0004124 cysteine synthase ac... 0.000986 2 / 31
GO:0042578 phosphoric ester hyd... 0.604836 2 / 1369
GO:0043178 alcohol binding 0.096045 1 / 68
GO:0032934 sterol binding 0.057658 1 / 40
GO:0003723 RNA binding 0.010394 11 / 3314
GO:0070566 adenylyltransferase ... 0.108052 1 / 77
GO:0140096 catalytic activity, ... 0.362514 14 / 8391
GO:0016791 phosphatase activity 0.814292 1 / 1126
GO:0015485 cholesterol binding 0.004443 1 / 3
GO:0008135 translation factor a... 0.002587 5 / 631
GO:0061598 molybdopterin adenyl... 0.002964 1 / 2
GO:0004721 phosphoprotein phosp... 0.676687 1 / 757
GO:0003743 translation initiati... 0.003021 4 / 398
GO:0008138 protein tyrosine/ser... 0.068788 1 / 48
GO:0033549 MAP kinase phosphata... 0.032132 1 / 22
GO:0017017 MAP kinase tyrosine/... 0.002964 1 / 2

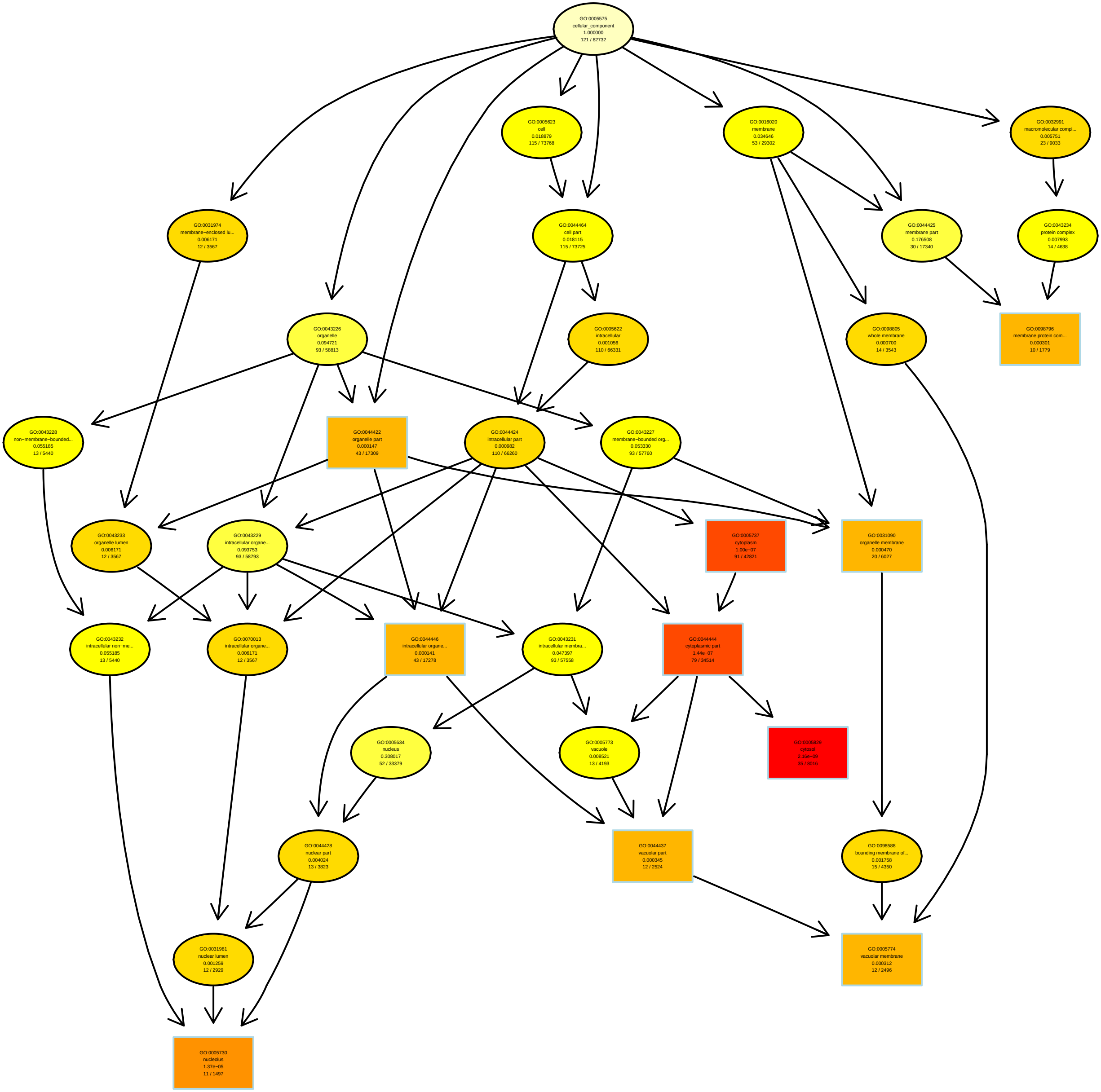
GO:0005575 cellular_component 1.000000 121 / 82732
GO:0005623 cell 0.018879 115 / 73768
GO:0016020 membrane 0.034646 53 / 29302
GO:0032991 macromolecular compl... 0.005751 23 / 9033
GO:0031974 membrane−enclosed lu... 0.006171 12 / 3567
GO:0044464 cell part 0.018115 115 / 73725
GO:0044425 membrane part 0.176508 30 / 17340
GO:0043234 protein complex 0.007993 14 / 4638
GO:0043226 organelle 0.094721 93 / 58813
GO:0005622 intracellular 0.001056 110 / 66331
GO:0098805 whole membrane 0.000700 14 / 3543
GO:0098796 membrane protein com... 0.000301 10 / 1779
GO:0043228 non−membrane−bounded... 0.055185 13 / 5440
GO:0044422 organelle part 0.000147 43 / 17309
GO:0044424 intracellular part 0.000982 110 / 66260
GO:0043227 membrane−bounded org... 0.053330 93 / 57760
GO:0043233 organelle lumen 0.006171 12 / 3567
GO:0043229 intracellular organe... 0.093753 93 / 58793
GO:0005737 cytoplasm 1.00e−07 91 / 42821
GO:0031090 organelle membrane 0.000470 20 / 6027
GO:0043232 intracellular non−me... 0.055185 13 / 5440
GO:0070013 intracellular organe... 0.006171 12 / 3567
GO:0044446 intracellular organe... 0.000141 43 / 17278
GO:0043231 intracellular membra... 0.047397 93 / 57558
GO:0044444 cytoplasmic part 1.44e−07 79 / 34514
GO:0005634 nucleus 0.308017 52 / 33379
GO:0005773 vacuole 0.008521 13 / 4193
GO:0005829 cytosol 2.16e−09 35 / 8016
GO:0044428 nuclear part 0.004024 13 / 3823
GO:0044437 vacuolar part 0.000345 12 / 2524
GO:0098588 bounding membrane of... 0.001758 15 / 4350
GO:0031981 nuclear lumen 0.001259 12 / 2929
GO:0005774 vacuolar membrane 0.000312 12 / 2496
GO:0005730 nucleolus 1.37e−05 11 / 1497
