# Supplementary material for: Differential Alternative Splicing Genes and Isoform Regulation Networks of Rapeseed (Brassica napus L.) Infected with Sclerotinia sclerotiorum
Source: Genes (Basel). 2020 Jul 13;11(7):784. doi: 10.3390/genes11070784 (PMC7397149; doi:10.3390/genes11070784)
Supplement: Supplementary file 1 [file genes-11-00784-s001.zip › Fig. S2 Enriched GO terms of genes in the 85 WGCNA modules.zip!/Fig. S2/CytoNodeorange-go.pdf]

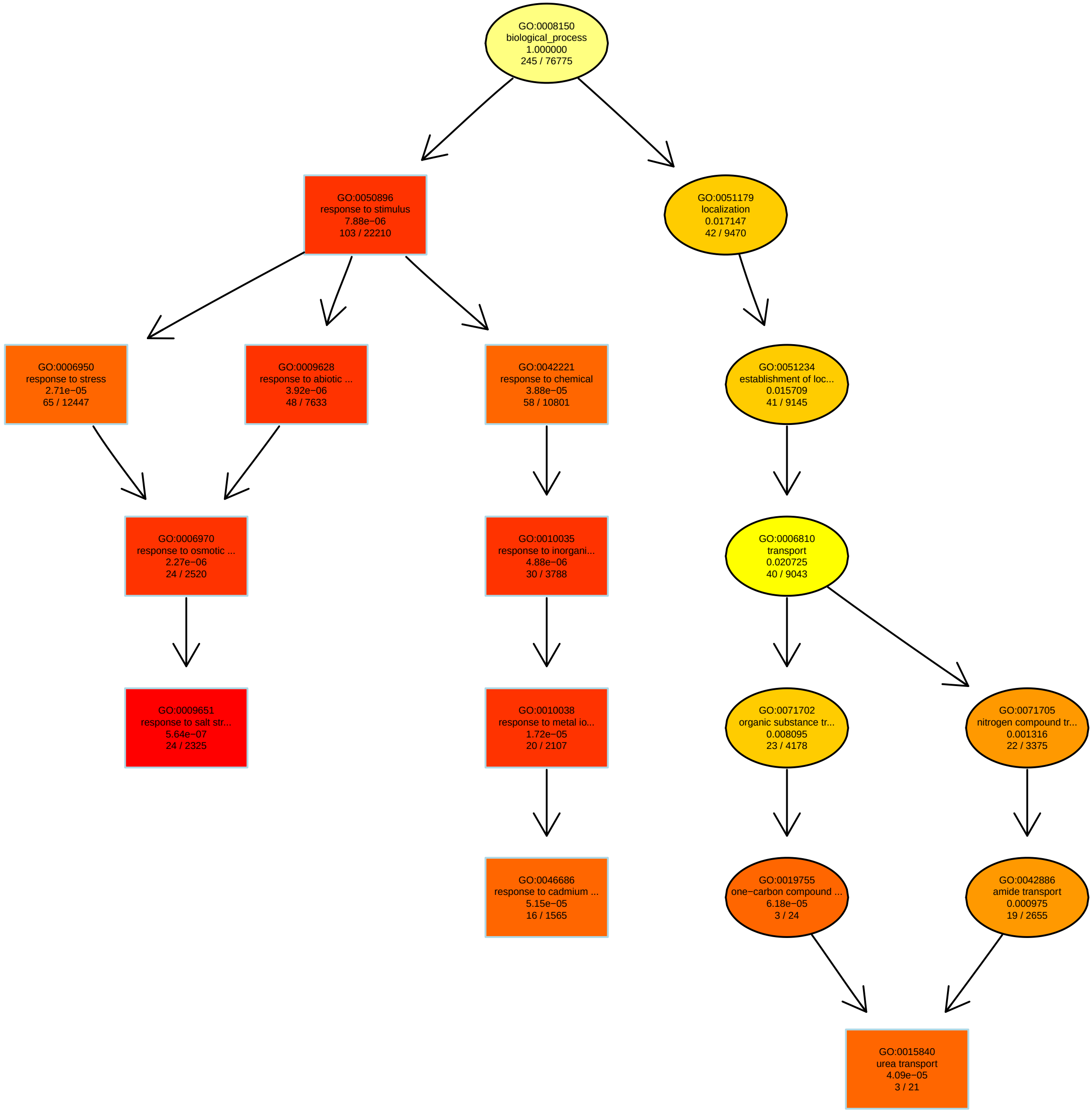
GO:0008150
biological_process
1.000000
245 / 76775
GO:0050896
response to stimulus
7.88e−06
103 / 22210
GO:0051179
localization
0.017147
42 / 9470
GO:0006950
response to stress
2.71e−05
65 / 12447
GO:0009628
response to abiotic ...
3.92e−06
48 / 7633
GO:0042221
response to chemical
3.88e−05
58 / 10801
GO:0051234
establishment of loc...
0.015709
41 / 9145
GO:0006970
response to osmotic ...
2.27e−06
24 / 2520
GO:0010035
response to inorgani...
4.88e−06
30 / 3788
GO:0006810
transport
0.020725
40 / 9043
GO:0009651
response to salt str...
5.64e−07
24 / 2325
GO:0010038
response to metal io...
1.72e−05
20 / 2107
GO:0071702
organic substance tr...
0.008095
23 / 4178
GO:0071705
nitrogen compound tr...
0.001316
22 / 3375
GO:0046686
response to cadmium ...
5.15e−05
16 / 1565
GO:0019755
one−carbon compound ...
6.18e−05
3 / 24
GO:0042886
amide transport
0.000975
19 / 2655
GO:0015840
urea transport
4.09e−05
3 / 21

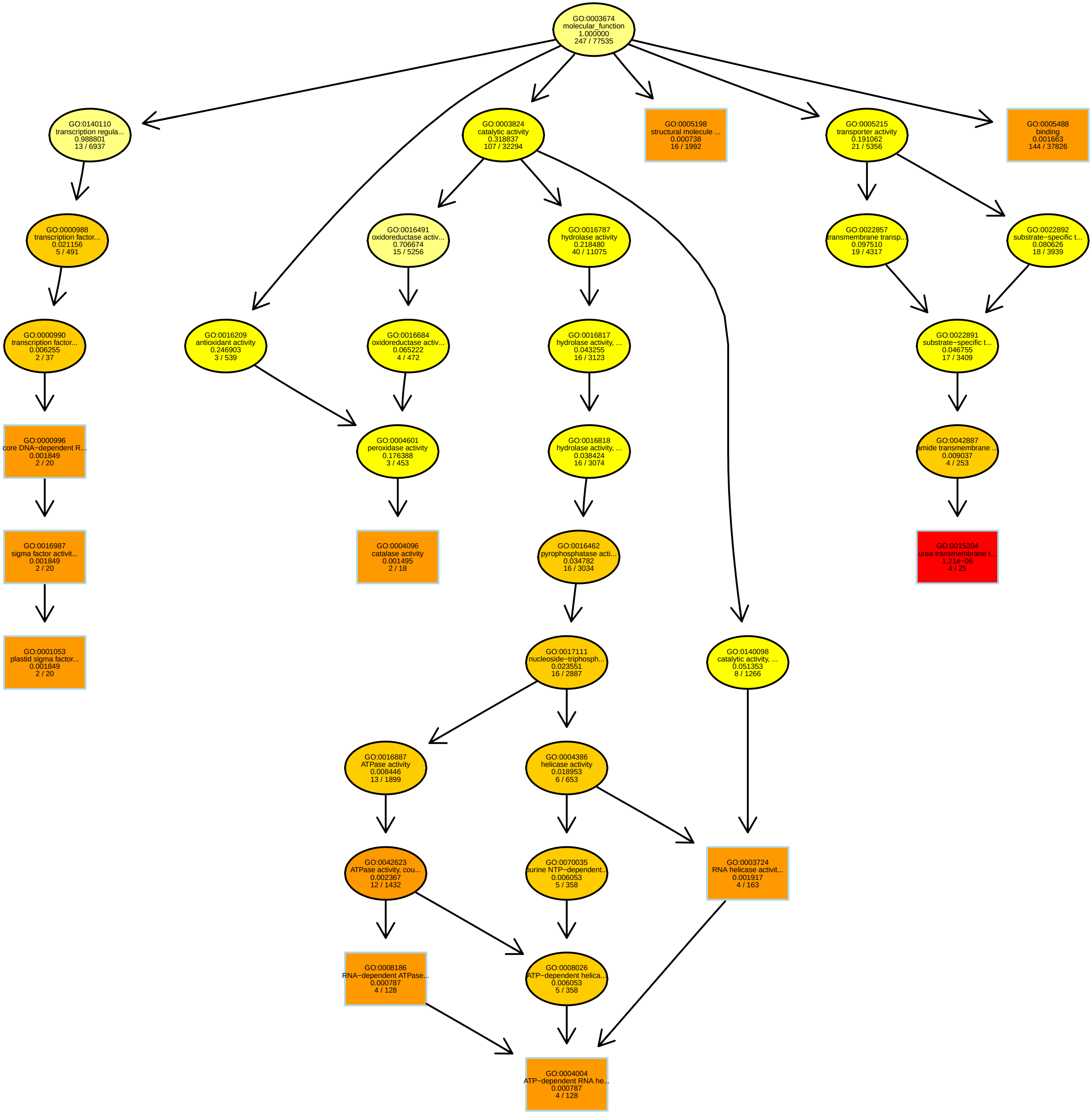
GO:0003674
molecular_function
1.000000
247 / 77535
GO:0140110
transcription regula...
0.988801
13 / 6937
GO:0003824
catalytic activity
0.318837
107 / 32294
GO:0005198
structural molecule ...
0.000738
16 / 1992
GO:0005215
transporter activity
0.191062
21 / 5356
GO:0005488
binding
0.001663
144 / 37826
GO:0000988
transcription factor...
0.021156
5 / 491
GO:0016491
oxidoreductase activ...
0.706674
15 / 5256
GO:0016787
hydrolase activity
0.218480
40 / 11075
GO:0022857
transmembrane transp...
0.097510
19 / 4317
GO:0022892
substrate−specific t...
0.080626
18 / 3939
GO:0000990
transcription factor...
0.006255
2 / 37
GO:0016209
antioxidant activity
0.246903
3 / 539
GO:0016684
oxidoreductase activ...
0.065222
4 / 472
GO:0016817
hydrolase activity, ...
0.043255
16 / 3123
GO:0022891
substrate−specific t...
0.046755
17 / 3409
GO:0000996
core DNA−dependent R...
0.001849
2 / 20
GO:0004601
peroxidase activity
0.176388
3 / 453
GO:0016818
hydrolase activity, ...
0.038424
16 / 3074
GO:0042887
amide transmembrane ...
0.009037
4 / 253
GO:0016987
sigma factor activit...
0.001849
2 / 20
GO:0004096
catalase activity
0.001495
2 / 18
GO:0016462
pyrophosphatase acti...
0.034782
16 / 3034
GO:0015204
urea transmembrane t...
1.21e−06
4 / 25
GO:0001053
plastid sigma factor...
0.001849
2 / 20
GO:0017111
nucleoside−triphosph...
0.023551
16 / 2887
GO:0140098
catalytic activity, ...
0.051353
8 / 1266
GO:0016887
ATPase activity
0.008446
13 / 1899
GO:0004386
helicase activity
0.018953
6 / 653
GO:0042623
ATPase activity, cou...
0.002367
12 / 1432
GO:0070035
purine NTP−dependent...
0.006053
5 / 358
GO:0003724
RNA helicase activit...
0.001917
4 / 163
GO:0008186
RNA−dependent ATPase...
0.000787
4 / 128
GO:0008026
ATP−dependent helica...
0.006053
5 / 358
GO:0004004
ATP−dependent RNA he...
0.000787
4 / 128

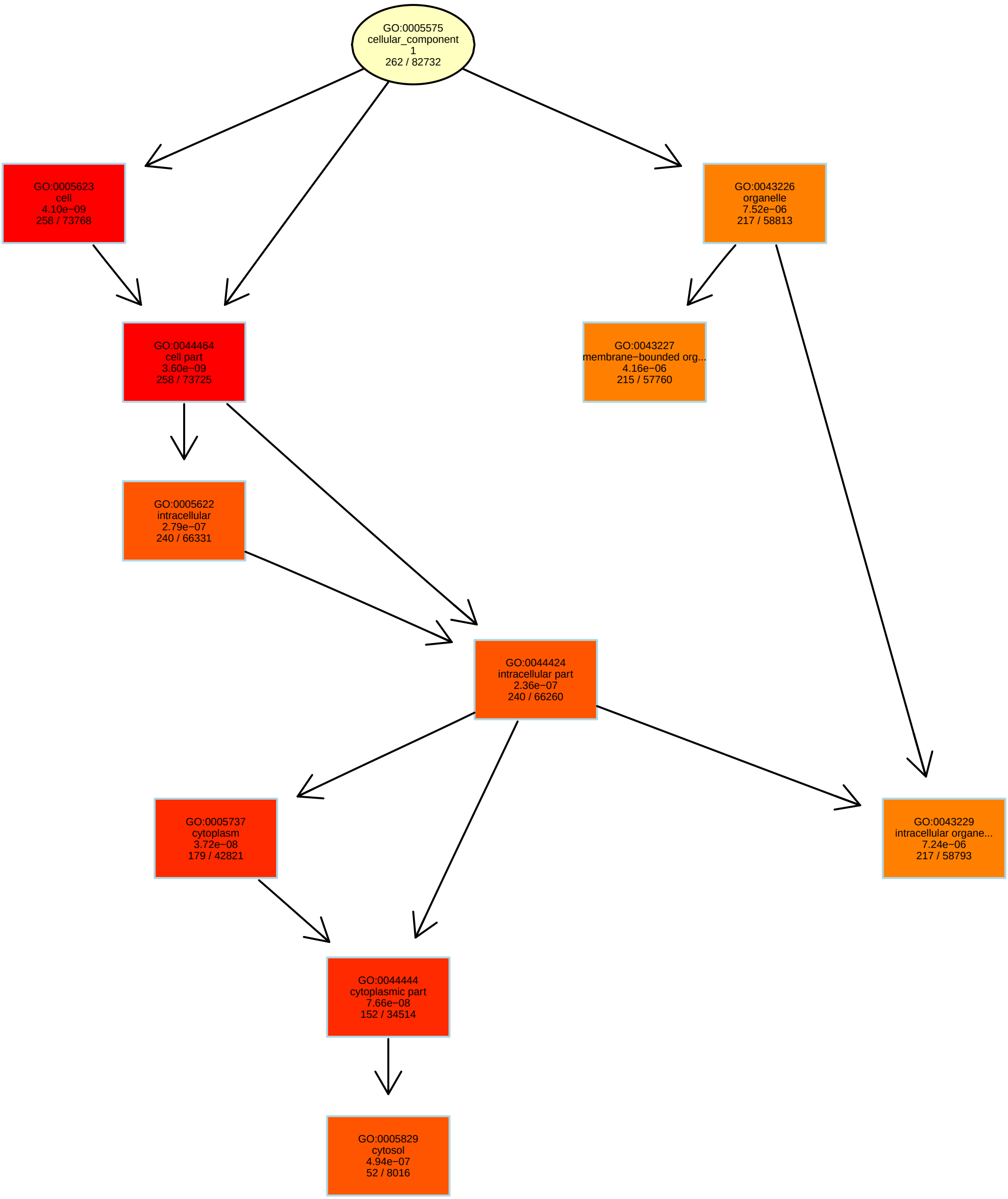
GO:0005575
cellular_component
1
262 / 82732
GO:0005623
cell
4.10e−09
258 / 73768
GO:0043226
organelle
7.52e−06
217 / 58813
GO:0044464
cell part
3.60e−09
258 / 73725
GO:0043227
membrane−bounded org...
4.16e−06
215 / 57760
GO:0005622
intracellular
2.79e−07
240 / 66331
GO:0044424
intracellular part
2.36e−07
240 / 66260
GO:0005737
cytoplasm
3.72e−08
179 / 42821
GO:0043229
intracellular organe...
7.24e−06
217 / 58793
GO:0044444
cytoplasmic part
7.66e−08
152 / 34514
GO:0005829
cytosol
4.94e−07
52 / 8016
