# Supplementary material for: Differential Alternative Splicing Genes and Isoform Regulation Networks of Rapeseed (Brassica napus L.) Infected with Sclerotinia sclerotiorum
Source: Genes (Basel). 2020 Jul 13;11(7):784. doi: 10.3390/genes11070784 (PMC7397149; doi:10.3390/genes11070784)
Supplement: Supplementary file 1 [file genes-11-00784-s001.zip › Fig. S2 Enriched GO terms of genes in the 85 WGCNA modules.zip!/Fig. S2/CytoNodeorangered3-go.pdf]

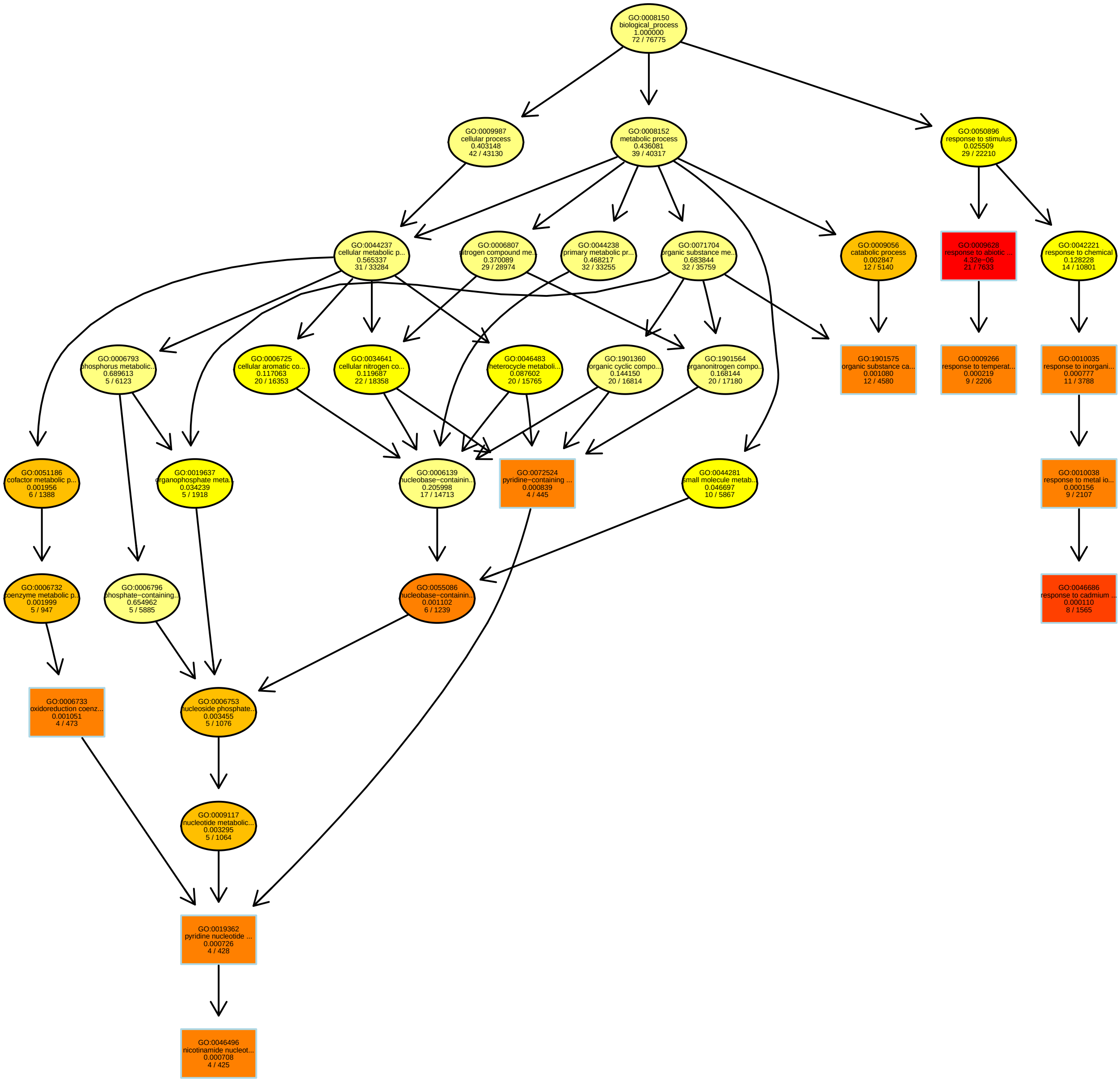

GO:0008150 biological_process 1.000000 72 / 76775
GO:0009987 cellular process 0.403148 42 / 43130
GO:0008152 metabolic process 0.436081 39 / 40317
GO:0050896 response to stimulus 0.025509 29 / 22210
GO:0044237 cellular metabolic p... 0.565337 31 / 33284
GO:0006807 nitrogen compound me... 0.370089 29 / 28974
GO:0044238 primary metabolic pr... 0.468217 32 / 33255
GO:0071704 organic substance me... 0.683844 32 / 35759
GO:0009056 catabolic process 0.002847 12 / 5140
GO:0009628 response to abiotic ... 4.32e−06 21 / 7633
GO:0042221 response to chemical 0.128228 14 / 10801
GO:0006793 phosphorus metabolic... 0.689613 5 / 6123
GO:0006725 cellular aromatic co... 0.117063 20 / 16353
GO:0034641 cellular nitrogen co... 0.119687 22 / 18358
GO:0046483 heterocycle metaboli... 0.087602 20 / 15765
GO:1901360 organic cyclic compo... 0.144150 20 / 16814
GO:1901564 organonitrogen compo... 0.168144 20 / 17180
GO:1901575 organic substance ca... 0.001080 12 / 4580
GO:0009266 response to temperat... 0.000219 9 / 2206
GO:0010035 response to inorgani... 0.000777 11 / 3788
GO:0051186 cofactor metabolic p... 0.001956 6 / 1388
GO:0019637 organophosphate meta... 0.034239 5 / 1918
GO:0006139 nucleobase−containin... 0.205998 17 / 14713
GO:0072524 pyridine−containing ... 0.000839 4 / 445
GO:0044281 small molecule metab... 0.046697 10 / 5867
GO:0010038 response to metal io... 0.000156 9 / 2107
GO:0006732 coenzyme metabolic p... 0.001999 5 / 947
GO:0006796 phosphate−containing... 0.654962 5 / 5885
GO:0055086 nucleobase−containin... 0.001102 6 / 1239
GO:0046686 response to cadmium ... 0.000110 8 / 1565
GO:0006733 oxidoreduction coenz... 0.001051 4 / 473
GO:0006753 nucleoside phosphate... 0.003455 5 / 1076
GO:0009117 nucleotide metabolic... 0.003295 5 / 1064
GO:0019362 pyridine nucleotide ... 0.000726 4 / 428
GO:0046496 nicotinamide nucleot... 0.000708 4 / 425

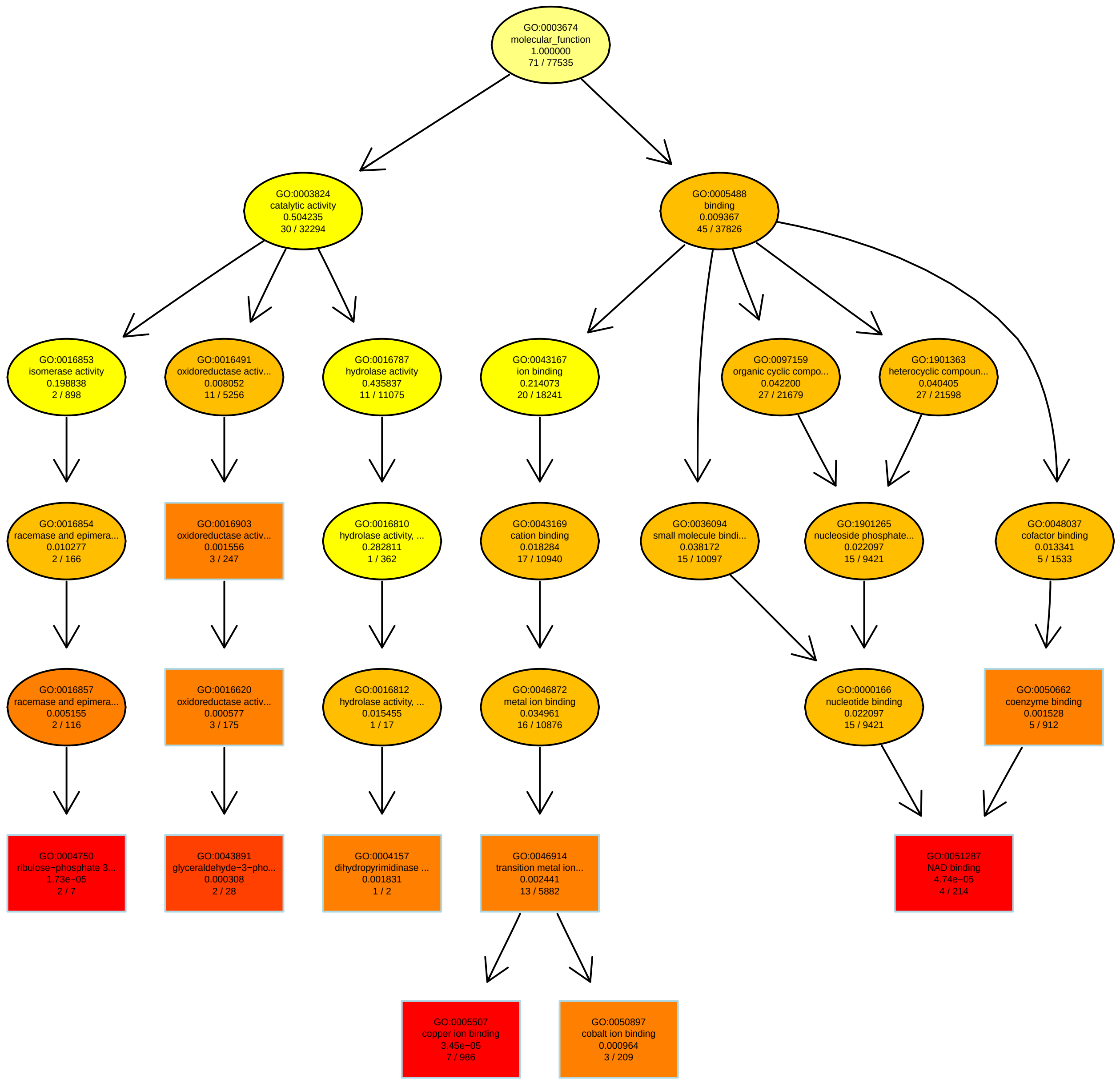
GO:0003674
molecular_function
1.000000
71 / 77535
GO:0003824
catalytic activity
0.504235
30 / 32294
GO:0005488
binding
0.009367
45 / 37826
GO:0016853
isomerase activity
0.198838
2 / 898
GO:0016491
oxidoreductase activ...
0.008052
11 / 5256
GO:0016787
hydrolase activity
0.435837
11 / 11075
GO:0043167
ion binding
0.214073
20 / 18241
GO:0097159
organic cyclic compo...
0.042200
27 / 21679
GO:1901363
heterocyclic compoun...
0.040405
27 / 21598
GO:0016854
racemase and epimera...
0.010277
2 / 166
GO:0016903
oxidoreductase activ...
0.001556
3 / 247
GO:0016810
hydrolase activity, ...
0.282811
1 / 362
GO:0043169
cation binding
0.018284
17 / 10940
GO:0036094
small molecule bindi...
0.038172
15 / 10097
GO:1901265
nucleoside phosphate...
0.022097
15 / 9421
GO:0048037
cofactor binding
0.013341
5 / 1533
GO:0016857
racemase and epimera...
0.005155
2 / 116
GO:0016620
oxidoreductase activ...
0.000577
3 / 175
GO:0016812
hydrolase activity, ...
0.015455
1 / 17
GO:0046872
metal ion binding
0.034961
16 / 10876
GO:0000166
nucleotide binding
0.022097
15 / 9421
GO:0050662
coenzyme binding
0.001528
5 / 912
GO:0004750
ribulose−phosphate 3...
1.73e−05
2 / 7
GO:0043891
glyceraldehyde−3−pho...
0.000308
2 / 28
GO:0004157
dihydropyrimidinase ...
0.001831
1 / 2
GO:0046914
transition metal ion...
0.002441
13 / 5882
GO:0051287
NAD binding
4.74e−05
4 / 214
GO:0005507
copper ion binding
3.45e−05
7 / 986
GO:0050897
cobalt ion binding
0.000964
3 / 209

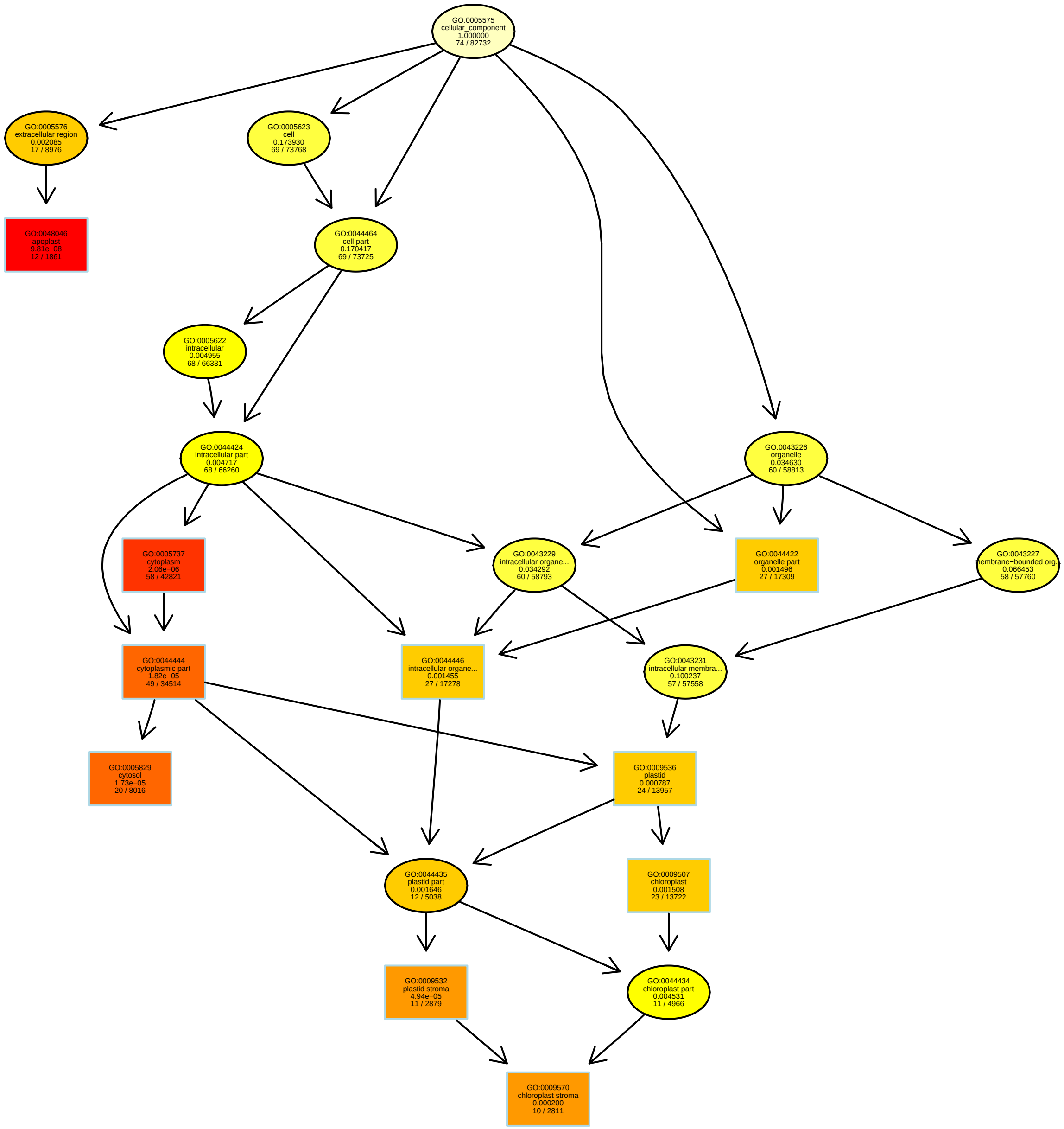
GO:0005575
cellular_component
1.000000
74 / 82732
GO:0005576
extracellular region
0.002085
17 / 8976
GO:0005623
cell
0.173930
69 / 73768
GO:0048046
apoplast
9.81e−08
12 / 1861
GO:0044464
cell part
0.170417
69 / 73725
GO:0005622
intracellular
0.004955
68 / 66331
GO:0044424
intracellular part
0.004717
68 / 66260
GO:0043226
organelle
0.034630
60 / 58813
GO:0005737
cytoplasm
2.06e−06
58 / 42821
GO:0043229
intracellular organe...
0.034292
60 / 58793
GO:0044422
organelle part
0.001496
27 / 17309
GO:0043227
membrane−bounded org...
0.066453
58 / 57760
GO:0044444
cytoplasmic part
1.82e−05
49 / 34514
GO:0044446
intracellular organe...
0.001455
27 / 17278
GO:0043231
intracellular membra...
0.100237
57 / 57558
GO:0005829
cytosol
1.73e−05
20 / 8016
GO:0009536
plastid
0.000787
24 / 13957
GO:0044435
plastid part
0.001646
12 / 5038
GO:0009507
chloroplast
0.001508
23 / 13722
GO:0009532
plastid stroma
4.94e−05
11 / 2879
GO:0044434
chloroplast part
0.004531
11 / 4966
GO:0009570
chloroplast stroma
0.000200
10 / 2811
