# Supplementary material for: Differential Alternative Splicing Genes and Isoform Regulation Networks of Rapeseed (Brassica napus L.) Infected with Sclerotinia sclerotiorum
Source: Genes (Basel). 2020 Jul 13;11(7):784. doi: 10.3390/genes11070784 (PMC7397149; doi:10.3390/genes11070784)
Supplement: Supplementary file 1 [file genes-11-00784-s001.zip › Fig. S2 Enriched GO terms of genes in the 85 WGCNA modules.zip!/Fig. S2/CytoNodeorangered4-go.pdf]

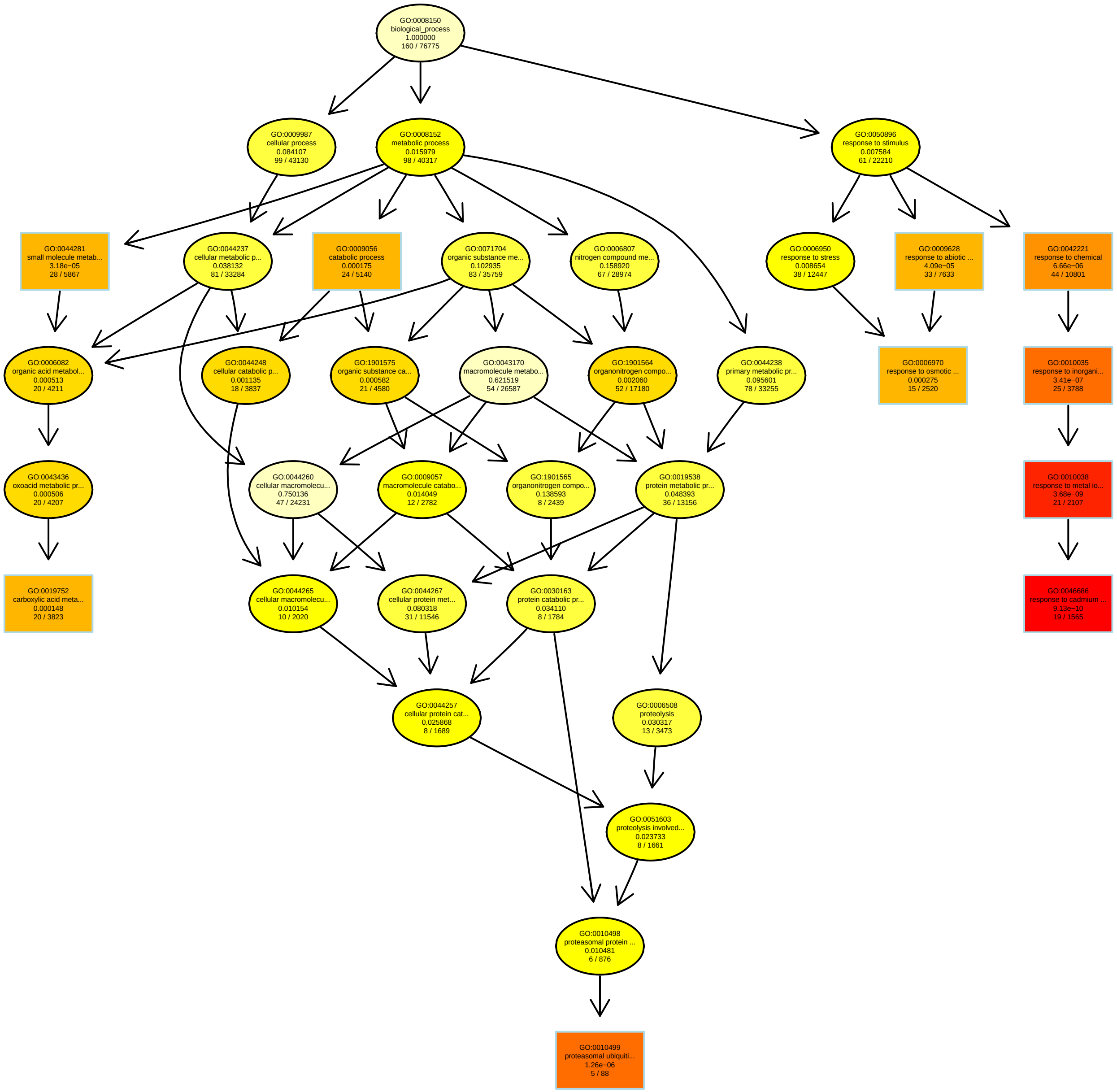

GO:0008150 biological_process 1.000000 160 / 76775
GO:0009987 cellular process 0.084107 99 / 43130
GO:0008152 metabolic process 0.015979 98 / 40317
GO:0050896 response to stimulus 0.007584 61 / 22210
GO:0044281 small molecule metab... 3.18e−05 28 / 5867
GO:0044237 cellular metabolic p... 0.038132 81 / 33284
GO:0009056 catabolic process 0.000175 24 / 5140
GO:0071704 organic substance me... 0.102935 83 / 35759
GO:0006807 nitrogen compound me... 0.158920 67 / 28974
GO:0006950 response to stress 0.008654 38 / 12447
GO:0009628 response to abiotic ... 4.09e−05 33 / 7633
GO:0042221 response to chemical 6.66e−06 44 / 10801
GO:0006082 organic acid metabol... 0.000513 20 / 4211
GO:0044248 cellular catabolic p... 0.001135 18 / 3837
GO:1901575 organic substance ca... 0.000582 21 / 4580
GO:0043170 macromolecule metabo... 0.621519 54 / 26587
GO:1901564 organonitrogen compo... 0.002060 52 / 17180
GO:0044238 primary metabolic pr... 0.095601 78 / 33255
GO:0006970 response to osmotic ... 0.000275 15 / 2520
GO:0010035 response to inorgani... 3.41e−07 25 / 3788
GO:0043436 oxoacid metabolic pr... 0.000506 20 / 4207
GO:0044260 cellular macromolecu... 0.750136 47 / 24231
GO:0009057 macromolecule catabo... 0.014049 12 / 2782
GO:1901565 organonitrogen compo... 0.138593 8 / 2439
GO:0019538 protein metabolic pr... 0.048393 36 / 13156
GO:0010038 response to metal io... 3.68e−09 21 / 2107
GO:0019752 carboxylic acid meta... 0.000148 20 / 3823
GO:0044265 cellular macromolecu... 0.010154 10 / 2020
GO:0044267 cellular protein met... 0.080318 31 / 11546
GO:0030163 protein catabolic pr... 0.034110 8 / 1784
GO:0046686 response to cadmium ... 9.13e−10 19 / 1565
GO:0044257 cellular protein cat... 0.025868 8 / 1689
GO:0006508 proteolysis 0.030317 13 / 3473
GO:0051603 proteolysis involved... 0.023733 8 / 1661
GO:0010498 proteasomal protein ... 0.010481 6 / 876
GO:0010499 proteasomal ubiquiti... 1.26e−06 5 / 88

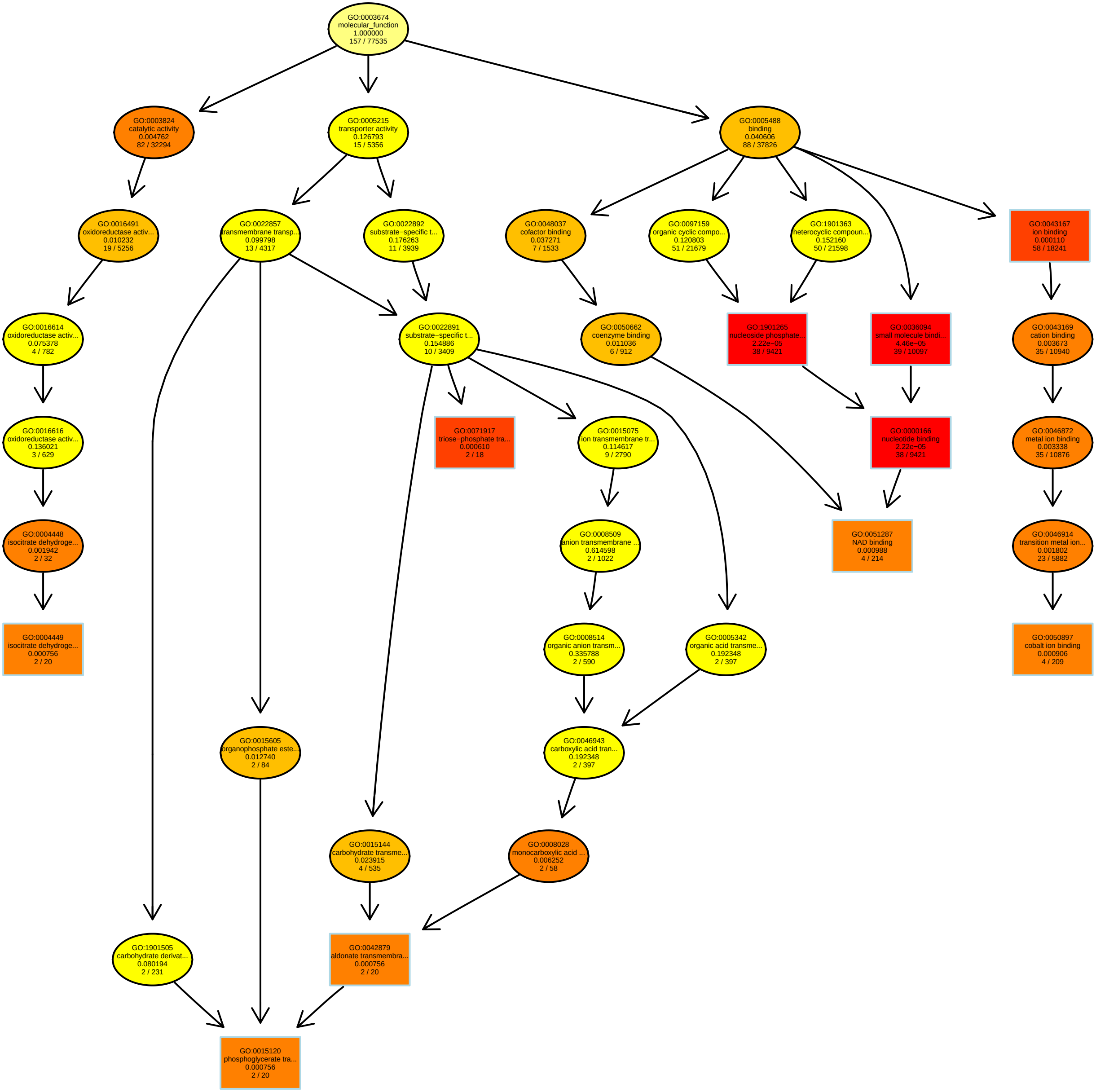

GO:0003674 molecular_function 1.000000 157 / 77535
GO:0003824 catalytic activity 0.004762 82 / 32294
GO:0005215 transporter activity 0.126793 15 / 5356
GO:0005488 binding 0.040606 88 / 37826
GO:0016491 oxidoreductase activ... 0.010232 19 / 5256
GO:0022857 transmembrane transp... 0.099798 13 / 4317
GO:0022892 substrate−specific t... 0.176263 11 / 3939
GO:0048037 cofactor binding 0.037271 7 / 1533
GO:0097159 organic cyclic compo... 0.120803 51 / 21679
GO:1901363 heterocyclic compoun... 0.152160 50 / 21598
GO:0043167 ion binding 0.000110 58 / 18241
GO:0016614 oxidoreductase activ... 0.075378 4 / 782
GO:0022891 substrate−specific t... 0.154886 10 / 3409
GO:0050662 coenzyme binding 0.011036 6 / 912
GO:1901265 nucleoside phosphate... 2.22e−05 38 / 9421
GO:0036094 small molecule bindi... 4.46e−05 39 / 10097
GO:0043169 cation binding 0.003673 35 / 10940
GO:0016616 oxidoreductase activ... 0.136021 3 / 629
GO:0071917 triose−phosphate tra... 0.000610 2 / 18
GO:0015075 ion transmembrane tr... 0.114617 9 / 2790
GO:0000166 nucleotide binding 2.22e−05 38 / 9421
GO:0046872 metal ion binding 0.003338 35 / 10876
GO:0004448 isocitrate dehydroge... 0.001942 2 / 32
GO:0008509 anion transmembrane ... 0.614598 2 / 1022
GO:0051287 NAD binding 0.000988 4 / 214
GO:0046914 transition metal ion... 0.001802 23 / 5882
GO:0004449 isocitrate dehydroge... 0.000756 2 / 20
GO:0008514 organic anion transm... 0.335788 2 / 590
GO:0005342 organic acid transme... 0.192348 2 / 397
GO:0050897 cobalt ion binding 0.000906 4 / 209
GO:0015605 organophosphate este... 0.012740 2 / 84
GO:0046943 carboxylic acid tran... 0.192348 2 / 397
GO:0015144 carbohydrate transme... 0.023915 4 / 535
GO:0008028 monocarboxylic acid ... 0.006252 2 / 58
GO:1901505 carbohydrate derivat... 0.080194 2 / 231
GO:0042879 aldonate transmembra... 0.000756 2 / 20
GO:0015120 phosphoglycerate tra... 0.000756 2 / 20

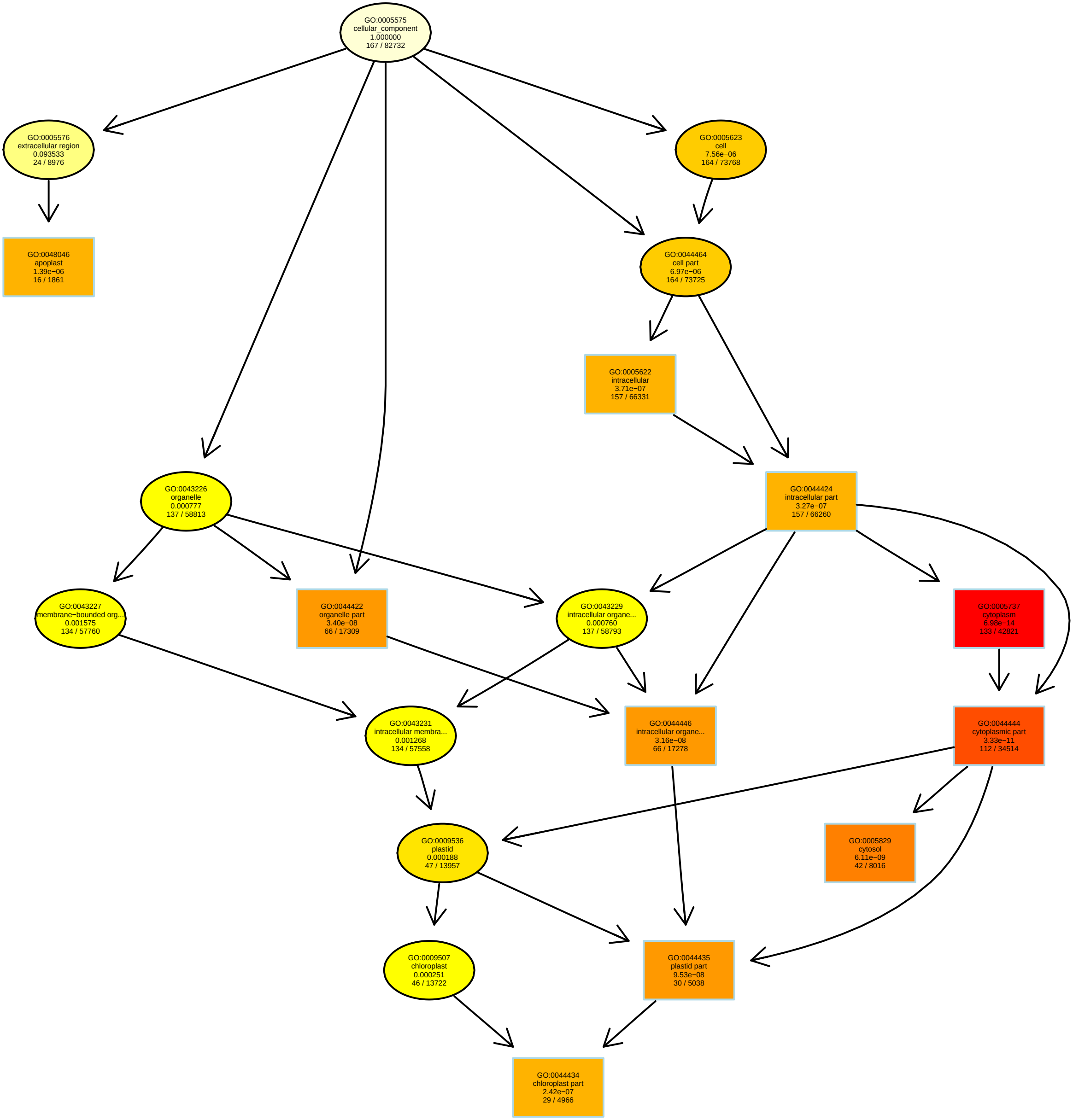
GO:0005575
cellular_component
1.000000
167 / 82732
GO:0005576
extracellular region
0.093533
24 / 8976
GO:0005623
cell
7.56e−06
164 / 73768
GO:0048046
apoplast
1.39e−06
16 / 1861
GO:0044464
cell part
6.97e−06
164 / 73725
GO:0005622
intracellular
3.71e−07
157 / 66331
GO:0043226
organelle
0.000777
137 / 58813
GO:0044424
intracellular part
3.27e−07
157 / 66260
GO:0043227
membrane−bounded org...
0.001575
134 / 57760
GO:0044422
organelle part
3.40e−08
66 / 17309
GO:0043229
intracellular organe...
0.000760
137 / 58793
GO:0005737
cytoplasm
6.98e−14
133 / 42821
GO:0043231
intracellular membra...
0.001268
134 / 57558
GO:0044446
intracellular organe...
3.16e−08
66 / 17278
GO:0044444
cytoplasmic part
3.33e−11
112 / 34514
GO:0009536
plastid
0.000188
47 / 13957
GO:0005829
cytosol
6.11e−09
42 / 8016
GO:0009507
chloroplast
0.000251
46 / 13722
GO:0044435
plastid part
9.53e−08
30 / 5038
GO:0044434
chloroplast part
2.42e−07
29 / 4966
