# Supplementary material for: Differential Alternative Splicing Genes and Isoform Regulation Networks of Rapeseed (Brassica napus L.) Infected with Sclerotinia sclerotiorum
Source: Genes (Basel). 2020 Jul 13;11(7):784. doi: 10.3390/genes11070784 (PMC7397149; doi:10.3390/genes11070784)
Supplement: Supplementary file 1 [file genes-11-00784-s001.zip › Fig. S2 Enriched GO terms of genes in the 85 WGCNA modules.zip!/Fig. S2/CytoNodepaleturquoise-go.pdf]

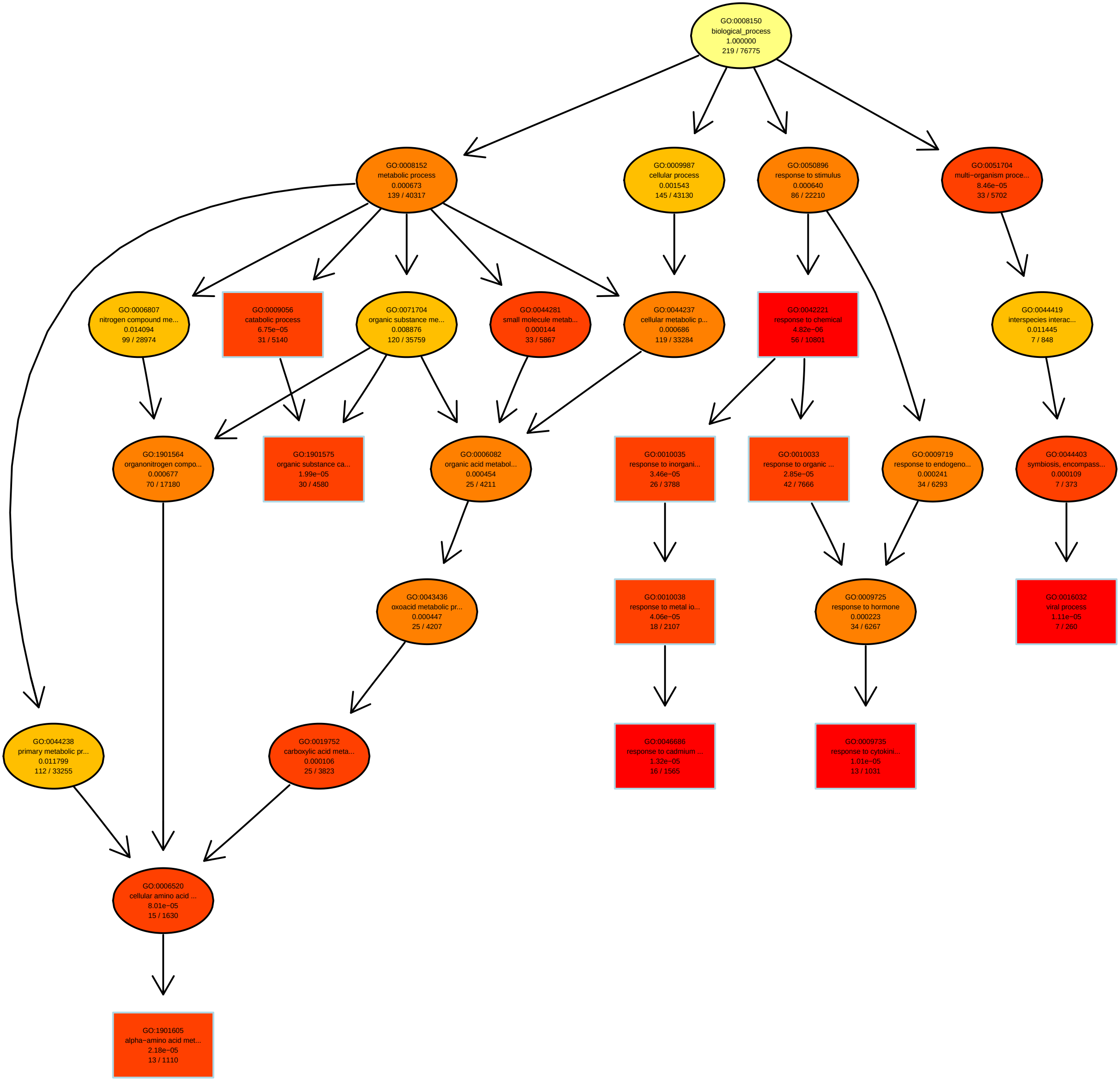
GO:0008150
biological_process
1.000000
219 / 76775
GO:0008152
metabolic process
0.000673
139 / 40317
GO:0009987
cellular process
0.001543
145 / 43130
GO:0050896
response to stimulus
0.000640
86 / 22210
GO:0051704
multi−organism proce...
8.46e−05
33 / 5702
GO:0006807
nitrogen compound me...
0.014094
99 / 28974
GO:0009056
catabolic process
6.75e−05
31 / 5140
GO:0071704
organic substance me...
0.008876
120 / 35759
GO:0044281
small molecule metab...
0.000144
33 / 5867
GO:0044237
cellular metabolic p...
0.000686
119 / 33284
GO:0042221
response to chemical
4.82e−06
56 / 10801
GO:0044419
interspecies interac...
0.011445
7 / 848
GO:1901564
organonitrogen compo...
0.000677
70 / 17180
GO:1901575
organic substance ca...
1.99e−05
30 / 4580
GO:0006082
organic acid metabol...
0.000454
25 / 4211
GO:0010035
response to inorgani...
3.46e−05
26 / 3788
GO:0010033
response to organic ...
2.85e−05
42 / 7666
GO:0009719
response to endogeno...
0.000241
34 / 6293
GO:0044403
symbiosis, encompass...
0.000109
7 / 373
GO:0043436
oxoacid metabolic pr...
0.000447
25 / 4207
GO:0010038
response to metal io...
4.06e−05
18 / 2107
GO:0009725
response to hormone
0.000223
34 / 6267
GO:0016032
viral process
1.11e−05
7 / 260
GO:0044238
primary metabolic pr...
0.011799
112 / 33255
GO:0019752
carboxylic acid meta...
0.000106
25 / 3823
GO:0046686
response to cadmium ...
1.32e−05
16 / 1565
GO:0009735
response to cytokini...
1.01e−05
13 / 1031
GO:0006520
cellular amino acid ...
8.01e−05
15 / 1630
GO:1901605
alpha−amino acid met...
2.18e−05
13 / 1110

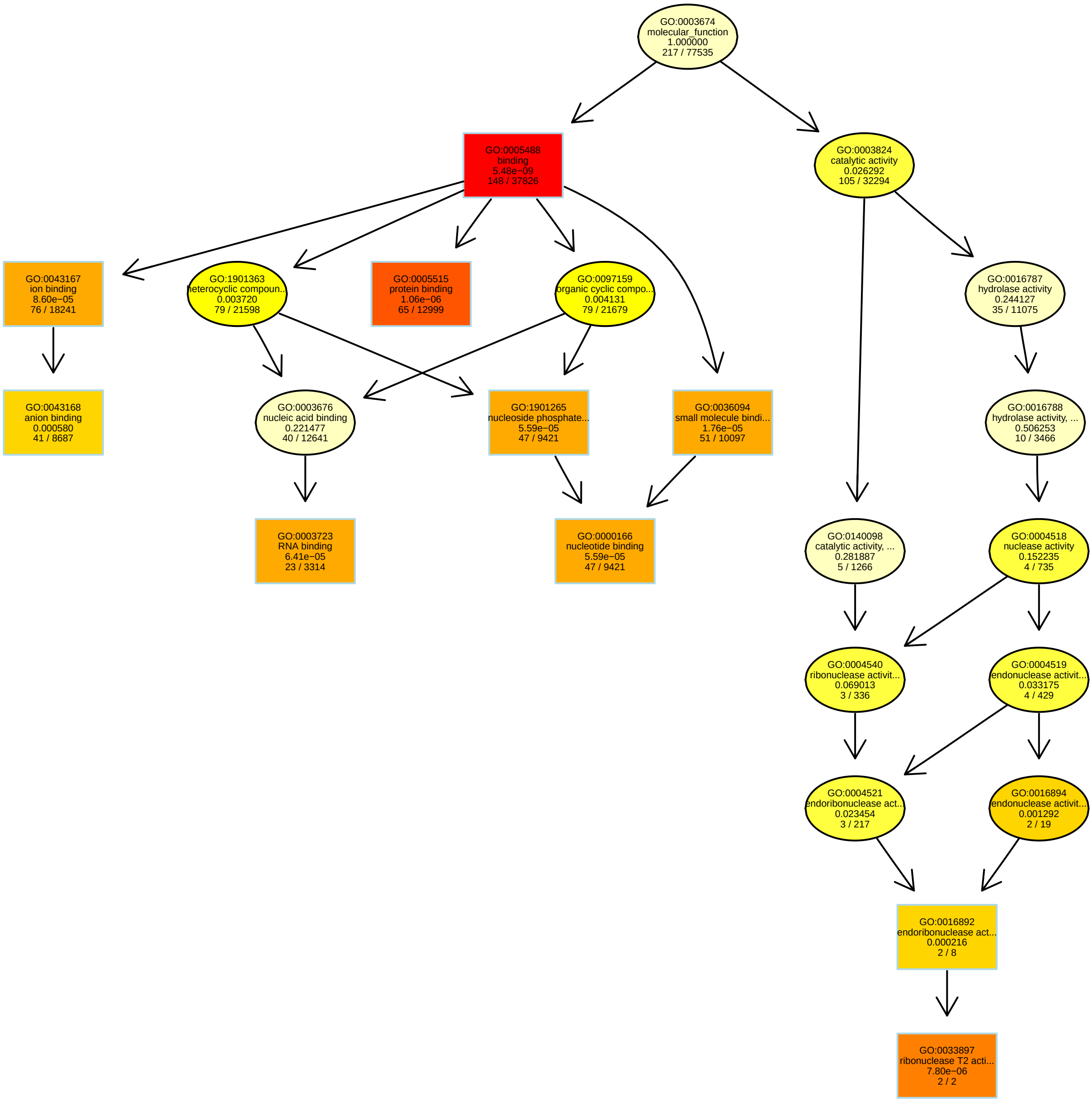
GO:0003674
molecular_function
1.000000
217 / 77535
GO:0005488
binding
5.48e−09
148 / 37826
GO:0003824
catalytic activity
0.026292
105 / 32294
GO:0043167
ion binding
8.60e−05
76 / 18241
GO:1901363
heterocyclic compoun...
0.003720
79 / 21598
GO:0005515
protein binding
1.06e−06
65 / 12999
GO:0097159
organic cyclic compo...
0.004131
79 / 21679
GO:0016787
hydrolase activity
0.244127
35 / 11075
GO:0043168
anion binding
0.000580
41 / 8687
GO:0003676
nucleic acid binding
0.221477
40 / 12641
GO:1901265
nucleoside phosphate...
5.59e−05
47 / 9421
GO:0036094
small molecule bindi...
1.76e−05
51 / 10097
GO:0016788
hydrolase activity, ...
0.506253
10 / 3466
GO:0003723
RNA binding
6.41e−05
23 / 3314
GO:0000166
nucleotide binding
5.59e−05
47 / 9421
GO:0140098
catalytic activity, ...
0.281887
5 / 1266
GO:0004518
nuclease activity
0.152235
4 / 735
GO:0004540
ribonuclease activit...
0.069013
3 / 336
GO:0004519
endonuclease activit...
0.033175
4 / 429
GO:0004521
endoribonuclease act...
0.023454
3 / 217
GO:0016894
endonuclease activit...
0.001292
2 / 19
GO:0016892
endoribonuclease act...
0.000216
2 / 8
GO:0033897
ribonuclease T2 acti...
7.80e−06
2 / 2

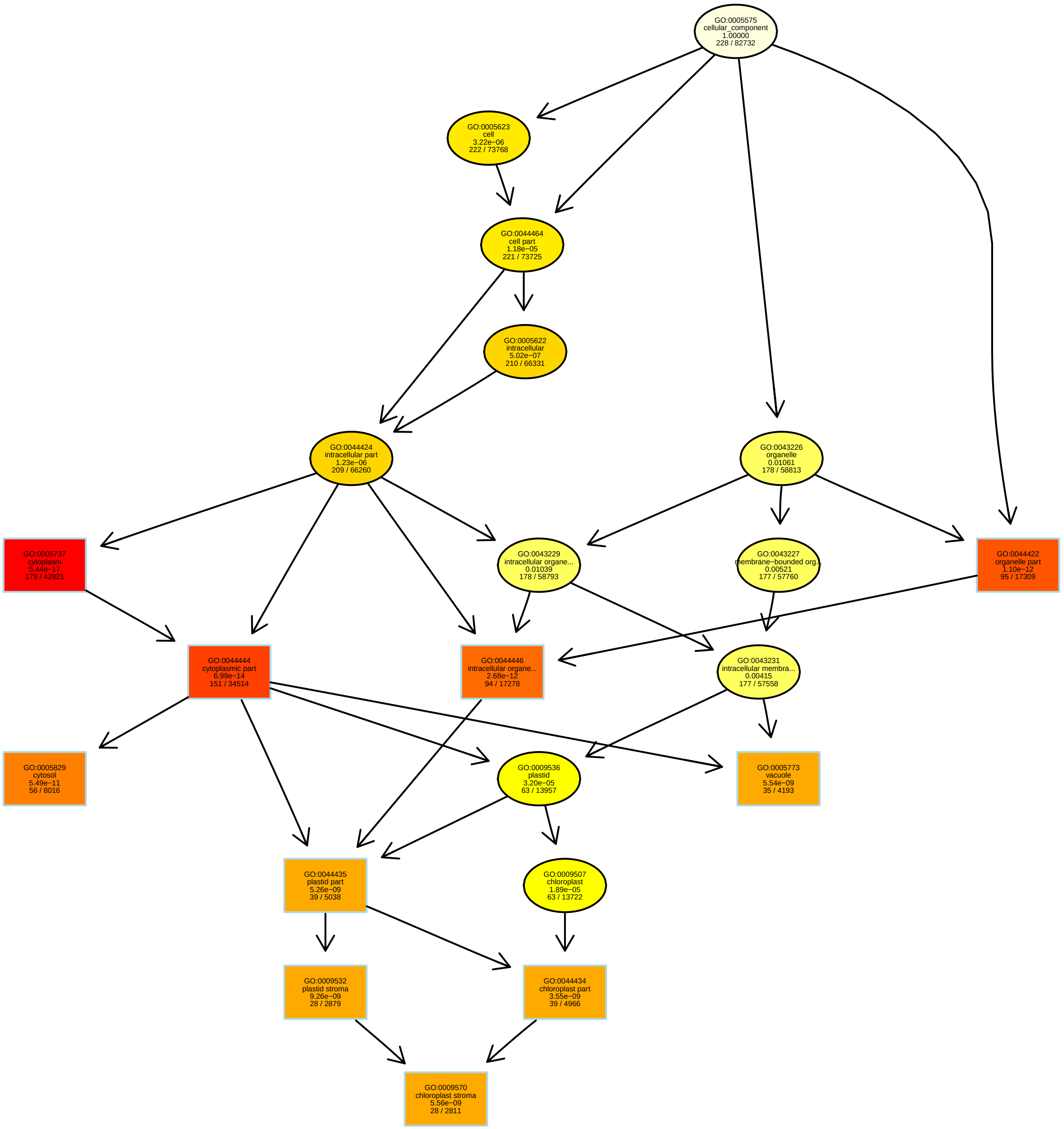
GO:0005575
cellular_component
1.00000
228 / 82732
GO:0005623
cell
3.22e−06
222 / 73768
GO:0044464
cell part
1.18e−05
221 / 73725
GO:0005622
intracellular
5.02e−07
210 / 66331
GO:0044424
intracellular part
1.23e−06
209 / 66260
GO:0043226
organelle
0.01061
178 / 58813
GO:0005737
cytoplasm
5.44e−17
179 / 42821
GO:0043229
intracellular organe...
0.01039
178 / 58793
GO:0043227
membrane−bounded org...
0.00521
177 / 57760
GO:0044422
organelle part
1.10e−12
95 / 17309
GO:0044444
cytoplasmic part
6.99e−14
151 / 34514
GO:0044446
intracellular organe...
2.68e−12
94 / 17278
GO:0043231
intracellular membra...
0.00415
177 / 57558
GO:0005829
cytosol
5.49e−11
56 / 8016
GO:0009536
plastid
3.20e−05
63 / 13957
GO:0005773
vacuole
5.54e−09
35 / 4193
GO:0044435
plastid part
5.26e−09
39 / 5038
GO:0009507
chloroplast
1.89e−05
63 / 13722
GO:0009532
plastid stroma
9.26e−09
28 / 2879
GO:0044434
chloroplast part
3.55e−09
39 / 4966
GO:0009570
chloroplast stroma
5.56e−09
28 / 2811
