# Supplementary material for: Differential Alternative Splicing Genes and Isoform Regulation Networks of Rapeseed (Brassica napus L.) Infected with Sclerotinia sclerotiorum
Source: Genes (Basel). 2020 Jul 13;11(7):784. doi: 10.3390/genes11070784 (PMC7397149; doi:10.3390/genes11070784)
Supplement: Supplementary file 1 [file genes-11-00784-s001.zip › Fig. S2 Enriched GO terms of genes in the 85 WGCNA modules.zip!/Fig. S2/CytoNodepalevioletred2-go.pdf]

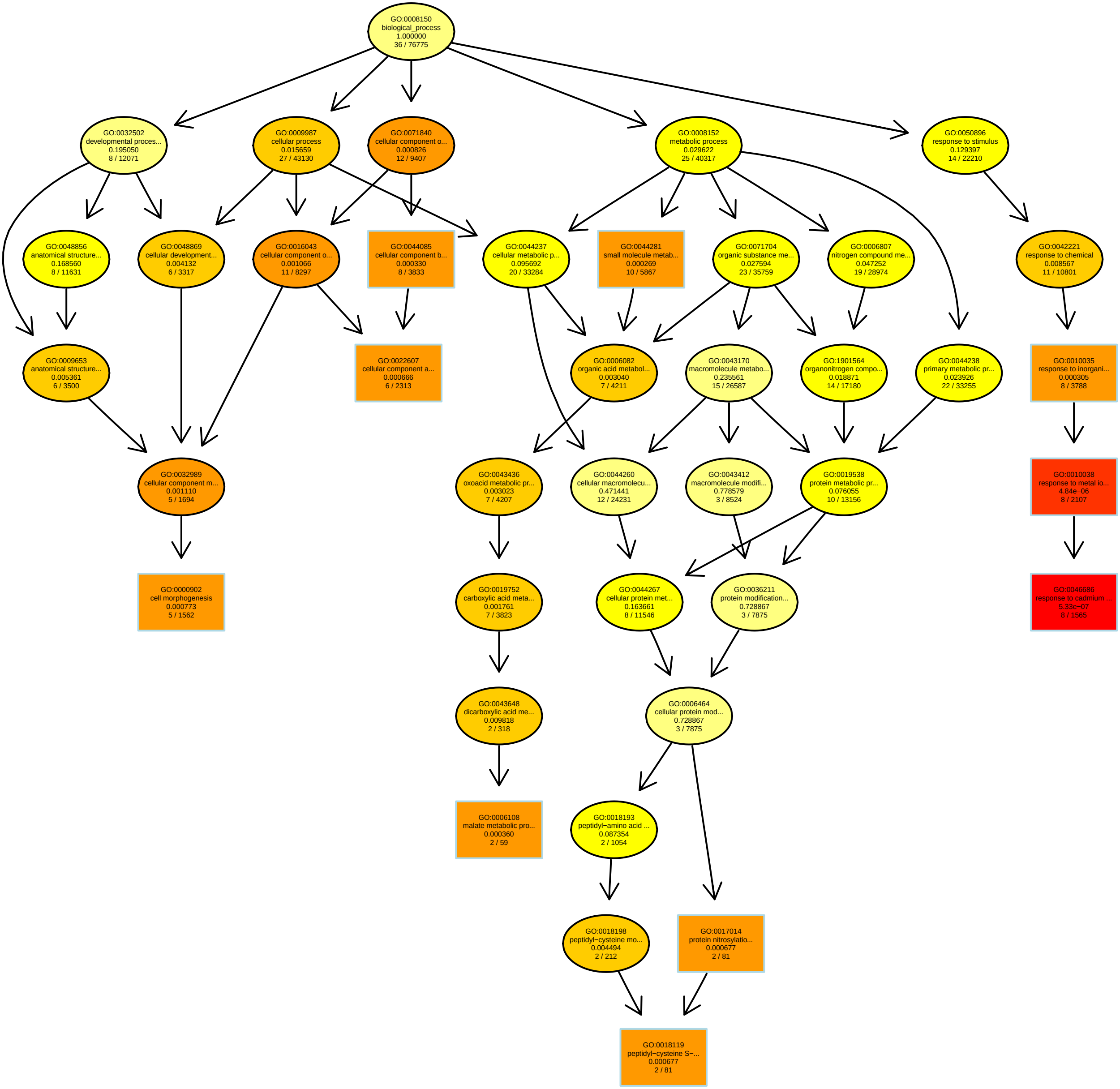
GO:0008150
biological_process
1.000000
36 / 76775
GO:0032502
developmental proces...
0.195050
8 / 12071
GO:0009987
cellular process
0.015659
27 / 43130
GO:0071840
cellular component o...
0.000826
12 / 9407
GO:0008152
metabolic process
0.029622
25 / 40317
GO:0050896
response to stimulus
0.129397
14 / 22210
GO:0048856
anatomical structure...
0.168560
8 / 11631
GO:0048869
cellular development...
0.004132
6 / 3317
GO:0016043
cellular component o...
0.001066
11 / 8297
GO:0044085
cellular component b...
0.000330
8 / 3833
GO:0044237
cellular metabolic p...
0.095692
20 / 33284
GO:0044281
small molecule metab...
0.000269
10 / 5867
GO:0071704
organic substance me...
0.027594
23 / 35759
GO:0006807
nitrogen compound me...
0.047252
19 / 28974
GO:0042221
response to chemical
0.008567
11 / 10801
GO:0009653
anatomical structure...
0.005361
6 / 3500
GO:0022607
cellular component a...
0.000666
6 / 2313
GO:0006082
organic acid metabol...
0.003040
7 / 4211
GO:0043170
macromolecule metabo...
0.235561
15 / 26587
GO:1901564
organonitrogen compo...
0.018871
14 / 17180
GO:0044238
primary metabolic pr...
0.023926
22 / 33255
GO:0010035
response to inorgani...
0.000305
8 / 3788
GO:0032989
cellular component m...
0.001110
5 / 1694
GO:0043436
oxoacid metabolic pr...
0.003023
7 / 4207
GO:0044260
cellular macromolecu...
0.471441
12 / 24231
GO:0043412
macromolecule modifi...
0.778579
3 / 8524
GO:0019538
protein metabolic pr...
0.076055
10 / 13156
GO:0010038
response to metal io...
4.84e−06
8 / 2107
GO:0000902
cell morphogenesis
0.000773
5 / 1562
GO:0019752
carboxylic acid meta...
0.001761
7 / 3823
GO:0044267
cellular protein met...
0.163661
8 / 11546
GO:0036211
protein modification...
0.728867
3 / 7875
GO:0046686
response to cadmium ...
5.33e−07
8 / 1565
GO:0043648
dicarboxylic acid me...
0.009818
2 / 318
GO:0006464
cellular protein mod...
0.728867
3 / 7875
GO:0006108
malate metabolic pro...
0.000360
2 / 59
GO:0018193
peptidyl−amino acid ...
0.087354
2 / 1054
GO:0018198
peptidyl−cysteine mo...
0.004494
2 / 212
GO:0017014
protein nitrosylatio...
0.000677
2 / 81
GO:0018119
peptidyl−cysteine S−...
0.000677
2 / 81

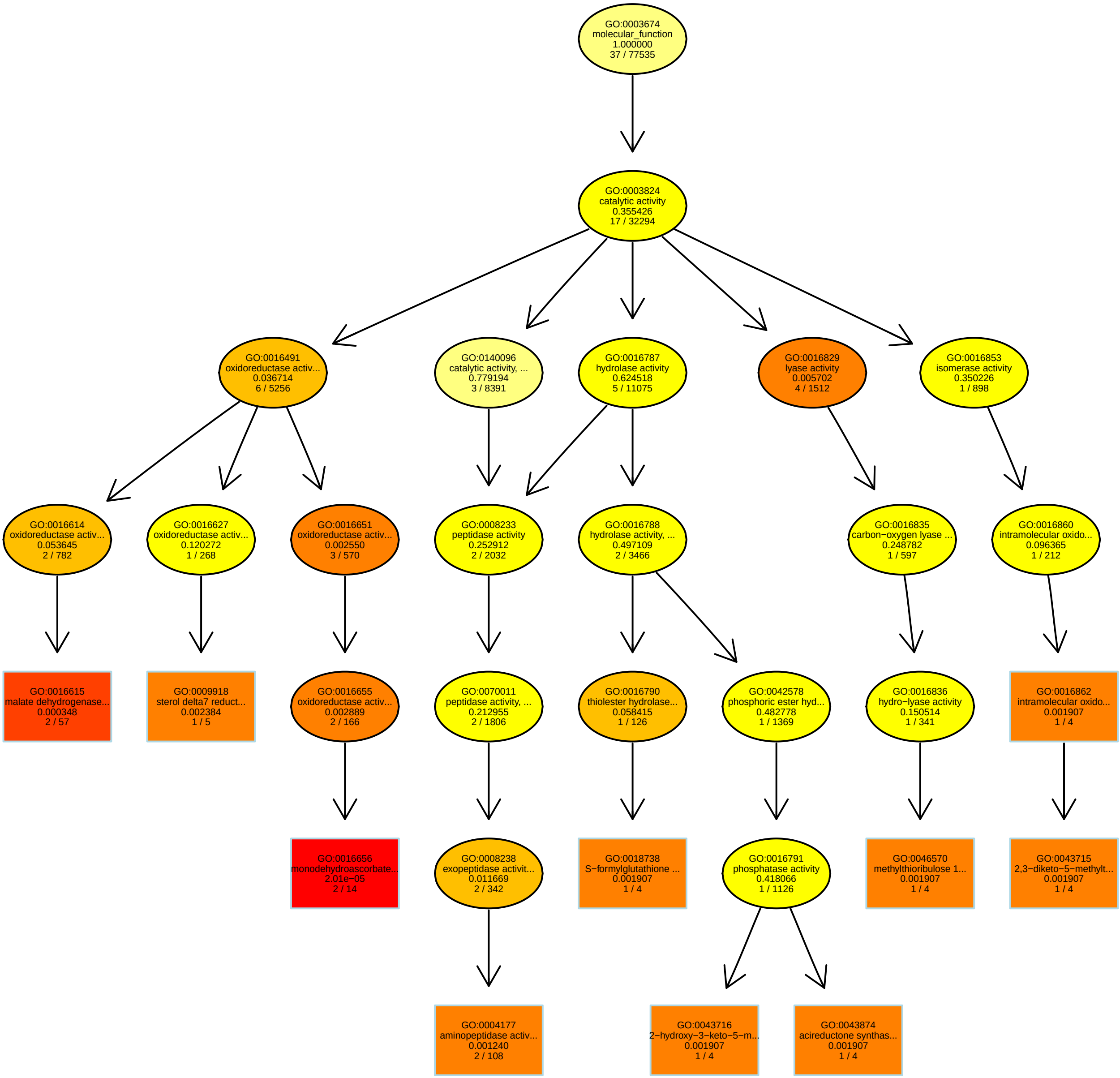
GO:0003674
molecular_function
1.000000
37 / 77535
GO:0003824
catalytic activity
0.355426
17 / 32294
GO:0016491
oxidoreductase activ...
0.036714
6 / 5256
GO:0140096
catalytic activity, ...
0.779194
3 / 8391
GO:0016787
hydrolase activity
0.624518
5 / 11075
GO:0016829
lyase activity
0.005702
4 / 1512
GO:0016853
isomerase activity
0.350226
1 / 898
GO:0016614
oxidoreductase activ...
0.053645
2 / 782
GO:0016627
oxidoreductase activ...
0.120272
1 / 268
GO:0016651
oxidoreductase activ...
0.002550
3 / 570
GO:0008233
peptidase activity
0.252912
2 / 2032
GO:0016788
hydrolase activity, ...
0.497109
2 / 3466
GO:0016835
carbon−oxygen lyase ...
0.248782
1 / 597
GO:0016860
intramolecular oxido...
0.096365
1 / 212
GO:0016615
malate dehydrogenase...
0.000348
2 / 57
GO:0009918
sterol delta7 reduct...
0.002384
1 / 5
GO:0016655
oxidoreductase activ...
0.002889
2 / 166
GO:0070011
peptidase activity, ...
0.212955
2 / 1806
GO:0016790
thiolester hydrolase...
0.058415
1 / 126
GO:0042578
phosphoric ester hyd...
0.482778
1 / 1369
GO:0016836
hydro−lyase activity
0.150514
1 / 341
GO:0016862
intramolecular oxido...
0.001907
1 / 4
GO:0016656
monodehydroascorbate...
2.01e−05
2 / 14
GO:0008238
exopeptidase activit...
0.011669
2 / 342
GO:0018738
S−formylglutathione ...
0.001907
1 / 4
GO:0016791
phosphatase activity
0.418066
1 / 1126
GO:0046570
methylthioribulose 1...
0.001907
1 / 4
GO:0043715
2,3−diketo−5−methylt...
0.001907
1 / 4
GO:0004177
aminopeptidase activ...
0.001240
2 / 108
GO:0043716
2−hydroxy−3−keto−5−m...
0.001907
1 / 4
GO:0043874
acireductone synthas...
0.001907
1 / 4

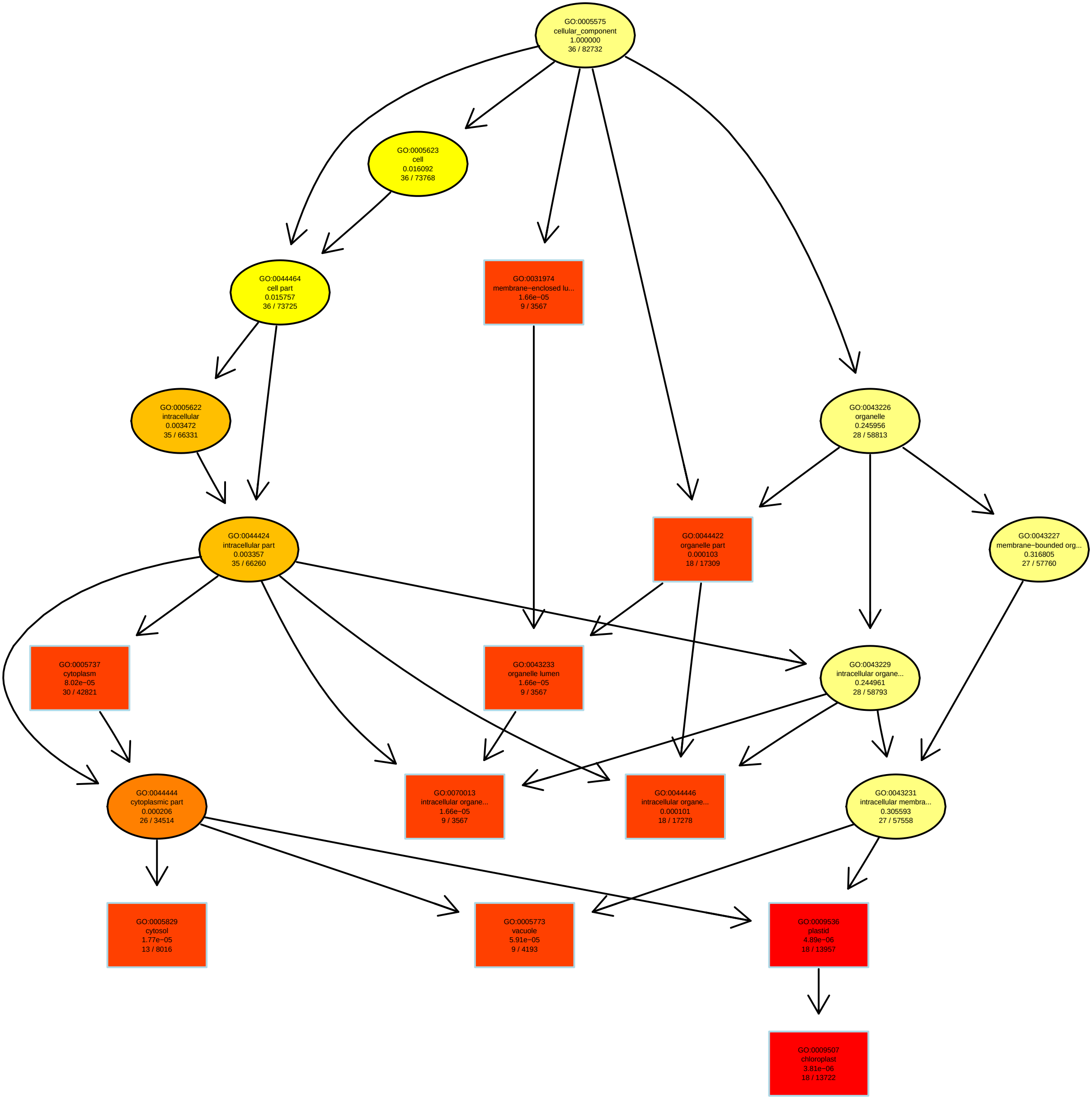
GO:0005575
cellular_component
1.000000
36 / 82732
GO:0005623
cell
0.016092
36 / 73768
GO:0044464
cell part
0.015757
36 / 73725
GO:0031974
membrane−enclosed lu...
1.66e−05
9 / 3567
GO:0005622
intracellular
0.003472
35 / 66331
GO:0043226
organelle
0.245956
28 / 58813
GO:0044424
intracellular part
0.003357
35 / 66260
GO:0044422
organelle part
0.000103
18 / 17309
GO:0043227
membrane−bounded org...
0.316805
27 / 57760
GO:0005737
cytoplasm
8.02e−05
30 / 42821
GO:0043233
organelle lumen
1.66e−05
9 / 3567
GO:0043229
intracellular organe...
0.244961
28 / 58793
GO:0044444
cytoplasmic part
0.000206
26 / 34514
GO:0070013
intracellular organe...
1.66e−05
9 / 3567
GO:0044446
intracellular organe...
0.000101
18 / 17278
GO:0043231
intracellular membra...
0.305593
27 / 57558
GO:0005829
cytosol
1.77e−05
13 / 8016
GO:0005773
vacuole
5.91e−05
9 / 4193
GO:0009536
plastid
4.89e−06
18 / 13957
GO:0009507
chloroplast
3.81e−06
18 / 13722
