# Supplementary material for: Differential Alternative Splicing Genes and Isoform Regulation Networks of Rapeseed (Brassica napus L.) Infected with Sclerotinia sclerotiorum
Source: Genes (Basel). 2020 Jul 13;11(7):784. doi: 10.3390/genes11070784 (PMC7397149; doi:10.3390/genes11070784)
Supplement: Supplementary file 1 [file genes-11-00784-s001.zip › Fig. S2 Enriched GO terms of genes in the 85 WGCNA modules.zip!/Fig. S2/CytoNodepalevioletred3-go.pdf]

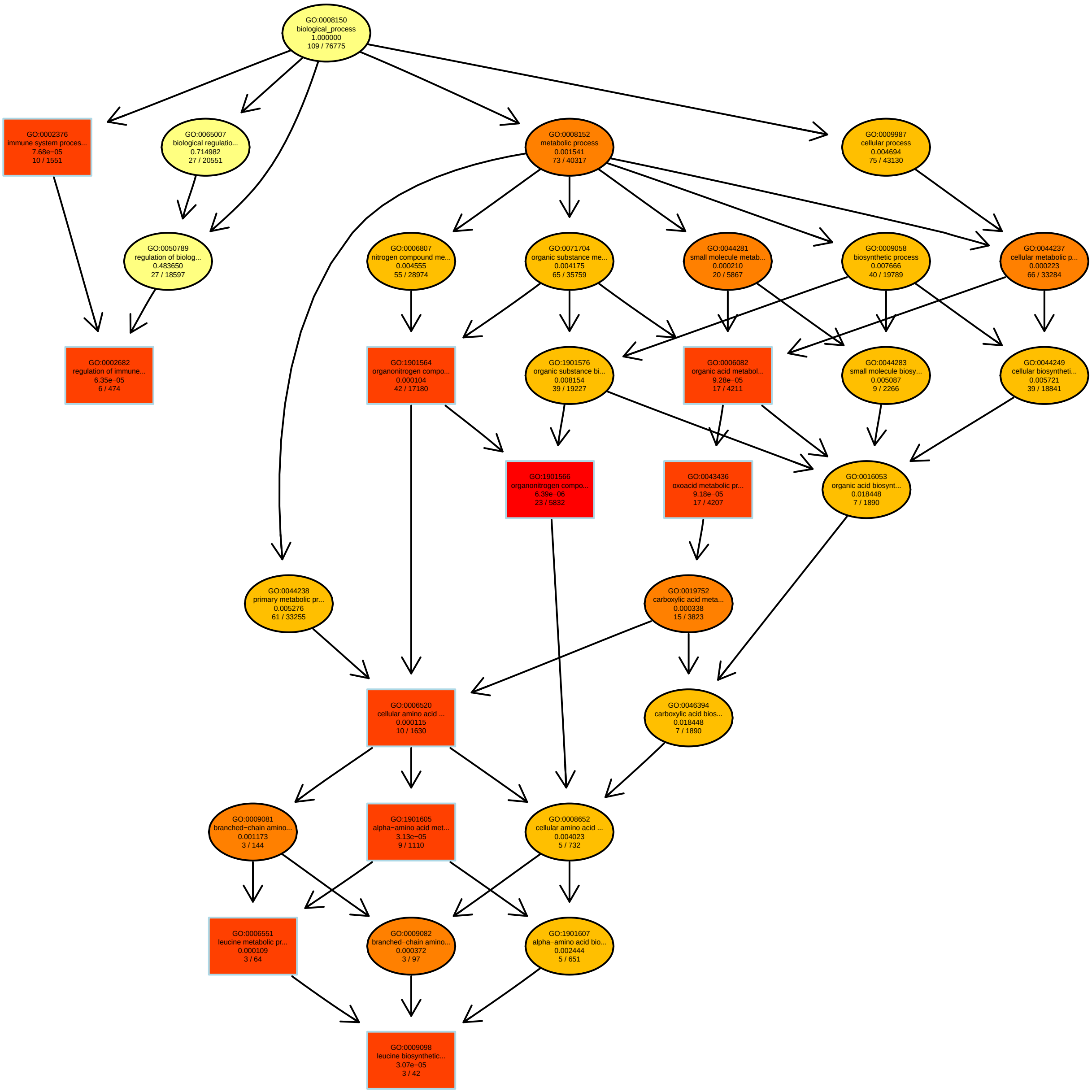
GO:0008150
biological_process
1.000000
109 / 76775
GO:0002376
immune system proces...
7.68e−05
10 / 1551
GO:0065007
biological regulatio...
0.714982
27 / 20551
GO:0008152
metabolic process
0.001541
73 / 40317
GO:0009987
cellular process
0.004694
75 / 43130
GO:0050789
regulation of biolog...
0.483650
27 / 18597
GO:0006807
nitrogen compound me...
0.004555
55 / 28974
GO:0071704
organic substance me...
0.004175
65 / 35759
GO:0044281
small molecule metab...
0.000210
20 / 5867
GO:0009058
biosynthetic process
0.007666
40 / 19789
GO:0044237
cellular metabolic p...
0.000223
66 / 33284
GO:0002682
regulation of immune...
6.35e−05
6 / 474
GO:1901564
organonitrogen compo...
0.000104
42 / 17180
GO:1901576
organic substance bi...
0.008154
39 / 19227
GO:0006082
organic acid metabol...
9.28e−05
17 / 4211
GO:0044283
small molecule biosy...
0.005087
9 / 2266
GO:0044249
cellular biosyntheti...
0.005721
39 / 18841
GO:1901566
organonitrogen compo...
6.39e−06
23 / 5832
GO:0043436
oxoacid metabolic pr...
9.18e−05
17 / 4207
GO:0016053
organic acid biosynt...
0.018448
7 / 1890
GO:0044238
primary metabolic pr...
0.005276
61 / 33255
GO:0019752
carboxylic acid meta...
0.000338
15 / 3823
GO:0006520
cellular amino acid ...
0.000115
10 / 1630
GO:0046394
carboxylic acid bios...
0.018448
7 / 1890
GO:0009081
branched−chain amino...
0.001173
3 / 144
GO:1901605
alpha−amino acid met...
3.13e−05
9 / 1110
GO:0008652
cellular amino acid ...
0.004023
5 / 732
GO:0006551
leucine metabolic pr...
0.000109
3 / 64
GO:0009082
branched−chain amino...
0.000372
3 / 97
GO:1901607
alpha−amino acid bio...
0.002444
5 / 651
GO:0009098
leucine biosynthetic...
3.07e−05
3 / 42

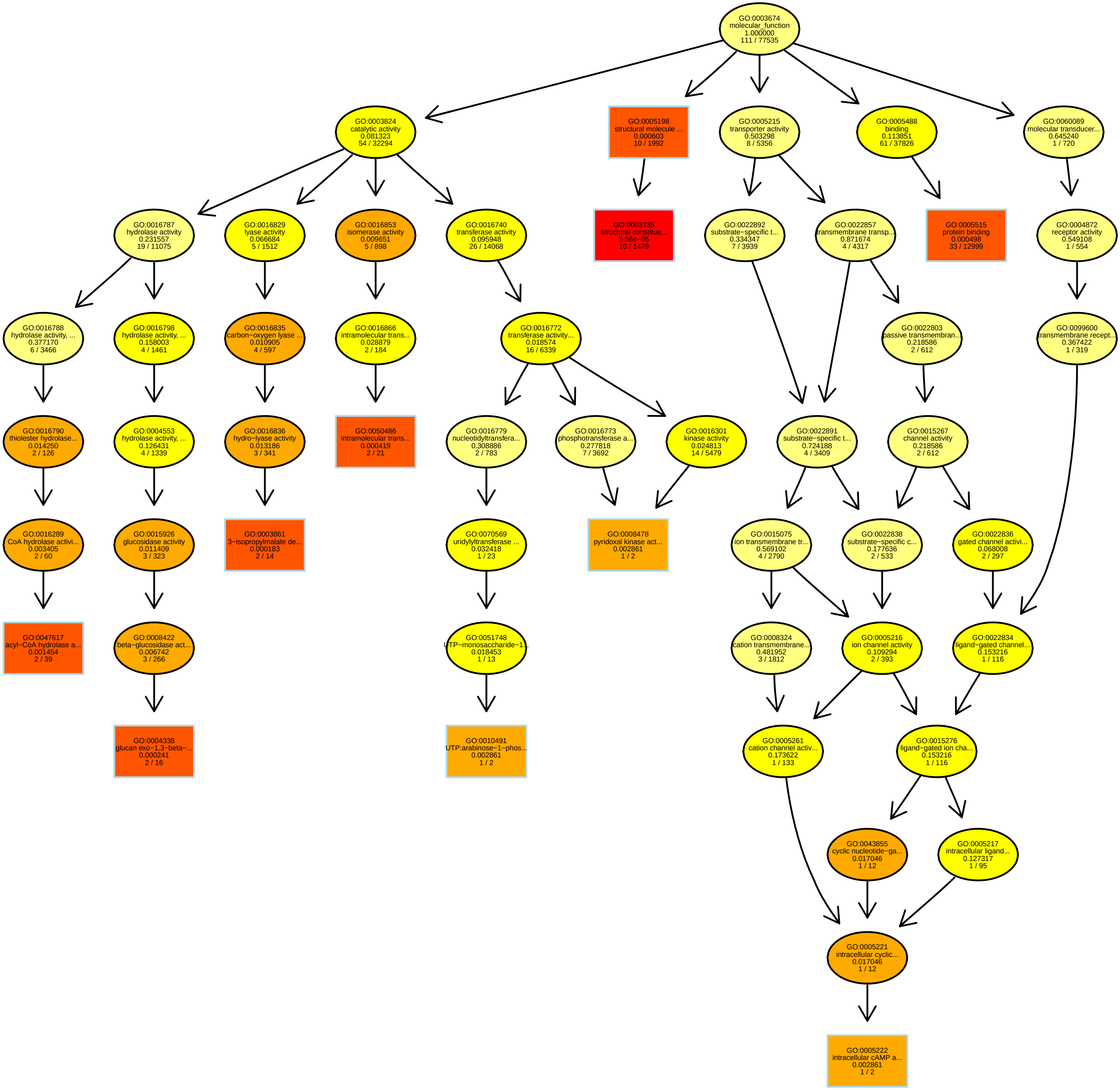
GO:0003674 molecular_function 1.000000 111 / 77535
GO:0003824 catalytic activity 0.081323 54 / 32294
GO:0005198 structural molecule ... 0.000603 10 / 1992
GO:0005215 transporter activity 0.503298 8 / 5356
GO:0005488 binding 0.113851 61 / 37826
GO:0060089 molecular transducer... 0.645240 1 / 720
GO:0016787 hydrolase activity 0.231557 19 / 11075
GO:0016829 lyase activity 0.066684 5 / 1512
GO:0016853 isomerase activity 0.009651 5 / 898
GO:0016740 transferase activity 0.095948 26 / 14068
GO:0003735 structural constitue... 5.56e−05 10 / 1478
GO:0022892 substrate−specific t... 0.334347 7 / 3939
GO:0022857 transmembrane transp... 0.871674 4 / 4317
GO:0005515 protein binding 0.000498 33 / 12999
GO:0004872 receptor activity 0.549108 1 / 554
GO:0016788 hydrolase activity, ... 0.377170 6 / 3466
GO:0016798 hydrolase activity, ... 0.158003 4 / 1461
GO:0016835 carbon−oxygen lyase ... 0.010905 4 / 597
GO:0016866 intramolecular trans... 0.028879 2 / 184
GO:0016772 transferase activity... 0.018574 16 / 6339
GO:0022803 passive transmembran... 0.218586 2 / 612
GO:0099600 transmembrane recept... 0.367422 1 / 319
GO:0016790 thiolester hydrolase... 0.014250 2 / 126
GO:0004553 hydrolase activity, ... 0.126431 4 / 1339
GO:0016836 hydro−lyase activity 0.013186 3 / 341
GO:0050486 intramolecular trans... 0.000419 2 / 21
GO:0016779 nucleotidyltransfera... 0.308886 2 / 783
GO:0016773 phosphotransferase a... 0.277818 7 / 3692
GO:0016301 kinase activity 0.024813 14 / 5479
GO:0022891 substrate−specific t... 0.724188 4 / 3409
GO:0015267 channel activity 0.218586 2 / 612
GO:0016289 CoA hydrolase activi... 0.003405 2 / 60
GO:0015926 glucosidase activity 0.011409 3 / 323
GO:0003861 3−isopropylmalate de... 0.000183 2 / 14
GO:0070569 uridylyltransferase ... 0.032418 1 / 23
GO:0008478 pyridoxal kinase act... 0.002861 1 / 2
GO:0015075 ion transmembrane tr... 0.569102 4 / 2790
GO:0022838 substrate−specific c... 0.177636 2 / 533
GO:0022836 gated channel activi... 0.068008 2 / 297
GO:0047617 acyl−CoA hydrolase a... 0.001454 2 / 39
GO:0008422 beta−glucosidase act... 0.006742 3 / 266
GO:0051748 UTP−monosaccharide−1... 0.018453 1 / 13
GO:0008324 cation transmembrane... 0.481952 3 / 1812
GO:0005216 ion channel activity 0.109294 2 / 393
GO:0022834 ligand−gated channel... 0.153216 1 / 116
GO:0004338 glucan exo−1,3−beta−... 0.000241 2 / 16
GO:0010491 UTP:arabinose−1−phos... 0.002861 1 / 2
GO:0005261 cation channel activ... 0.173622 1 / 133
GO:0015276 ligand−gated ion cha... 0.153216 1 / 116
GO:0043855 cyclic nucleotide−ga... 0.017046 1 / 12
GO:0005217 intracellular ligand... 0.127317 1 / 95
GO:0005221 intracellular cyclic... 0.017046 1 / 12
GO:0005222 intracellular cAMP a... 0.002861 1 / 2

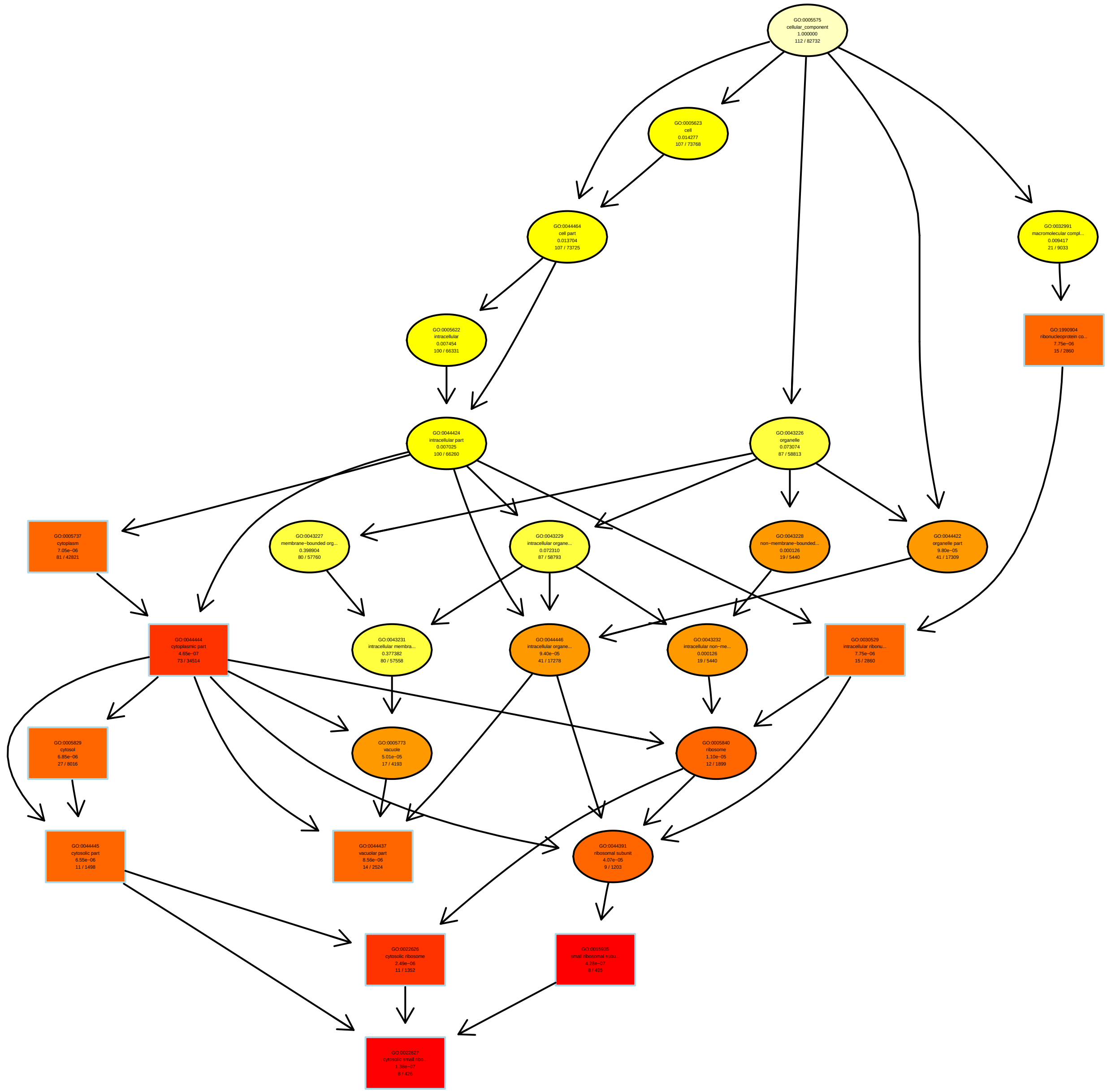

GO:0005575
cellular_component
1.000000
112 / 82732
GO:0005623
cell
0.014277
107 / 73768
GO:0044464
cell part
0.013704
107 / 73725
GO:0032991
macromolecular compl...
0.009417
21 / 9033
GO:0005622
intracellular
0.007454
100 / 66331
GO:1990904
ribonucleoprotein co...
7.75e−06
15 / 2860
GO:0044424
intracellular part
0.007025
100 / 66260
GO:0043226
organelle
0.073074
87 / 58813
GO:0005737
cytoplasm
7.05e−06
81 / 42821
GO:0043227
membrane−bounded org...
0.398904
80 / 57760
GO:0043229
intracellular organe...
0.072310
87 / 58793
GO:0043228
non−membrane−bounded...
0.000126
19 / 5440
GO:0044422
organelle part
9.80e−05
41 / 17309
GO:0044444
cytoplasmic part
4.65e−07
73 / 34514
GO:0043231
intracellular membra...
0.377382
80 / 57558
GO:0044446
intracellular organe...
9.40e−05
41 / 17278
GO:0043232
intracellular non−me...
0.000126
19 / 5440
GO:0030529
intracellular ribonu...
7.75e−06
15 / 2860
GO:0005829
cytosol
6.85e−06
27 / 8016
GO:0005773
vacuole
5.01e−05
17 / 4193
GO:0005840
ribosome
1.10e−05
12 / 1899
GO:0044445
cytosolic part
6.55e−06
11 / 1498
GO:0044437
vacuolar part
8.56e−06
14 / 2524
GO:0044391
ribosomal subunit
4.07e−05
9 / 1203
GO:0022626
cytosolic ribosome
2.49e−06
11 / 1352
GO:0015935
small ribosomal subu...
4.28e−07
8 / 495
GO:0022627
cytosolic small ribo...
1.38e−07
8 / 426
