# Supplementary material for: Differential Alternative Splicing Genes and Isoform Regulation Networks of Rapeseed (Brassica napus L.) Infected with Sclerotinia sclerotiorum
Source: Genes (Basel). 2020 Jul 13;11(7):784. doi: 10.3390/genes11070784 (PMC7397149; doi:10.3390/genes11070784)
Supplement: Supplementary file 1 [file genes-11-00784-s001.zip › Fig. S2 Enriched GO terms of genes in the 85 WGCNA modules.zip!/Fig. S2/CytoNodepink-go.pdf]

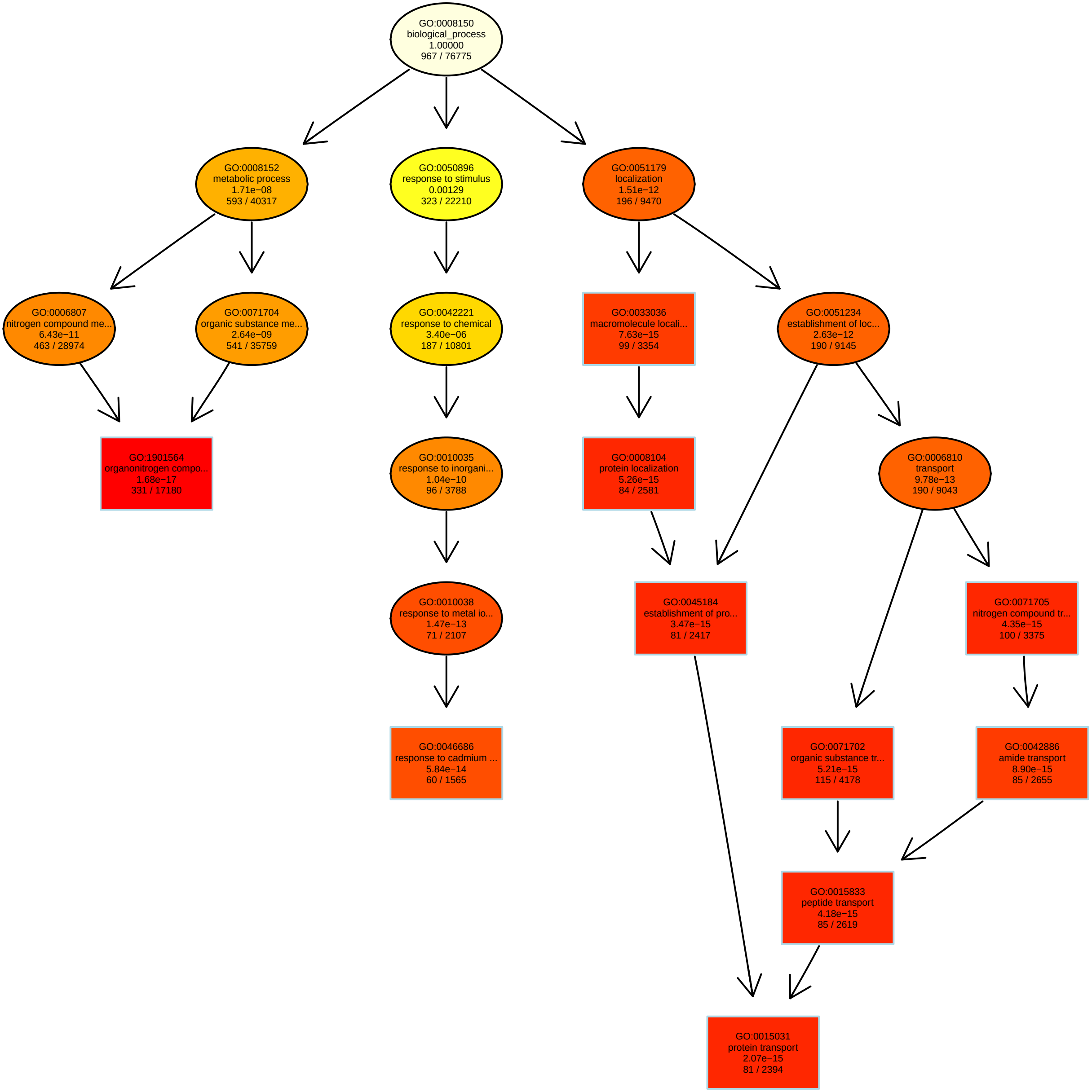
GO:0008150
biological_process
1.00000
967 / 76775
GO:0008152
metabolic process
1.71e−08
593 / 40317
GO:0050896
response to stimulus
0.00129
323 / 22210
GO:0051179
localization
1.51e−12
196 / 9470
GO:0006807
nitrogen compound me...
6.43e−11
463 / 28974
GO:0071704
organic substance me...
2.64e−09
541 / 35759
GO:0042221
response to chemical
3.40e−06
187 / 10801
GO:0033036
macromolecule locali...
7.63e−15
99 / 3354
GO:0051234
establishment of loc...
2.63e−12
190 / 9145
GO:1901564
organonitrogen compo...
1.68e−17
331 / 17180
GO:0010035
response to inorgani...
1.04e−10
96 / 3788
GO:0008104
protein localization
5.26e−15
84 / 2581
GO:0006810
transport
9.78e−13
190 / 9043
GO:0010038
response to metal io...
1.47e−13
71 / 2107
GO:0045184
establishment of pro...
3.47e−15
81 / 2417
GO:0071705
nitrogen compound tr...
4.35e−15
100 / 3375
GO:0046686
response to cadmium ...
5.84e−14
60 / 1565
GO:0071702
organic substance tr...
5.21e−15
115 / 4178
GO:0042886
amide transport
8.90e−15
85 / 2655
GO:0015833
peptide transport
4.18e−15
85 / 2619
GO:0015031
protein transport
2.07e−15
81 / 2394

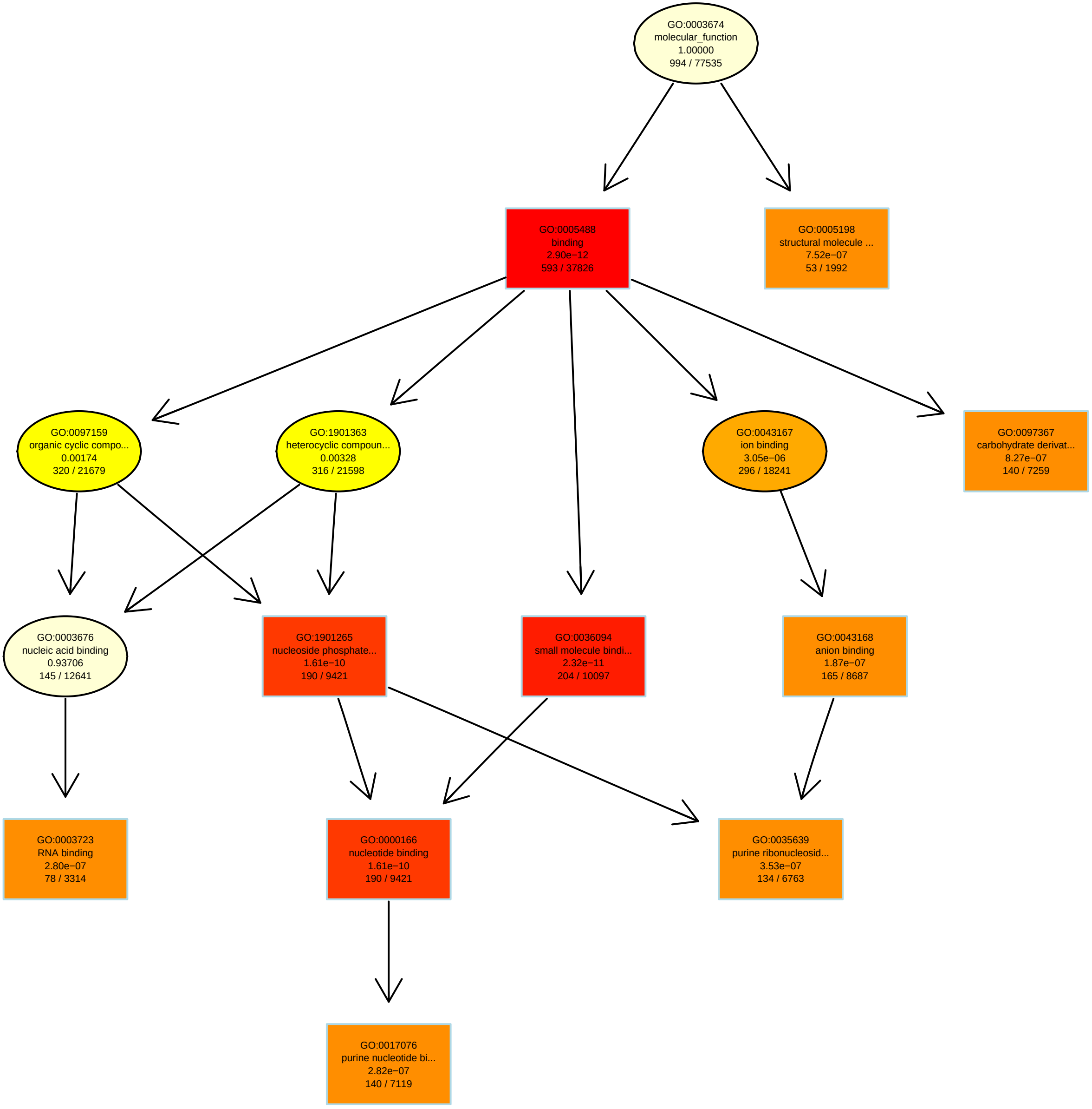

GO:0003674
molecular_function
1.00000
994 / 77535
GO:0005488
binding
2.90e−12
593 / 37826
GO:0005198
structural molecule ...
7.52e−07
53 / 1992
GO:0097159
organic cyclic compo...
0.00174
320 / 21679
GO:1901363
heterocyclic compoun...
0.00328
316 / 21598
GO:0043167
ion binding
3.05e−06
296 / 18241
GO:0097367
carbohydrate derivat...
8.27e−07
140 / 7259
GO:0003676
nucleic acid binding
0.93706
145 / 12641
GO:1901265
nucleoside phosphate...
1.61e−10
190 / 9421
GO:0036094
small molecule bindi...
2.32e−11
204 / 10097
GO:0043168
anion binding
1.87e−07
165 / 8687
GO:0003723
RNA binding
2.80e−07
78 / 3314
GO:0000166
nucleotide binding
1.61e−10
190 / 9421
GO:0035639
purine ribonucleosid...
3.53e−07
134 / 6763
GO:0017076
purine nucleotide bi...
2.82e−07
140 / 7119

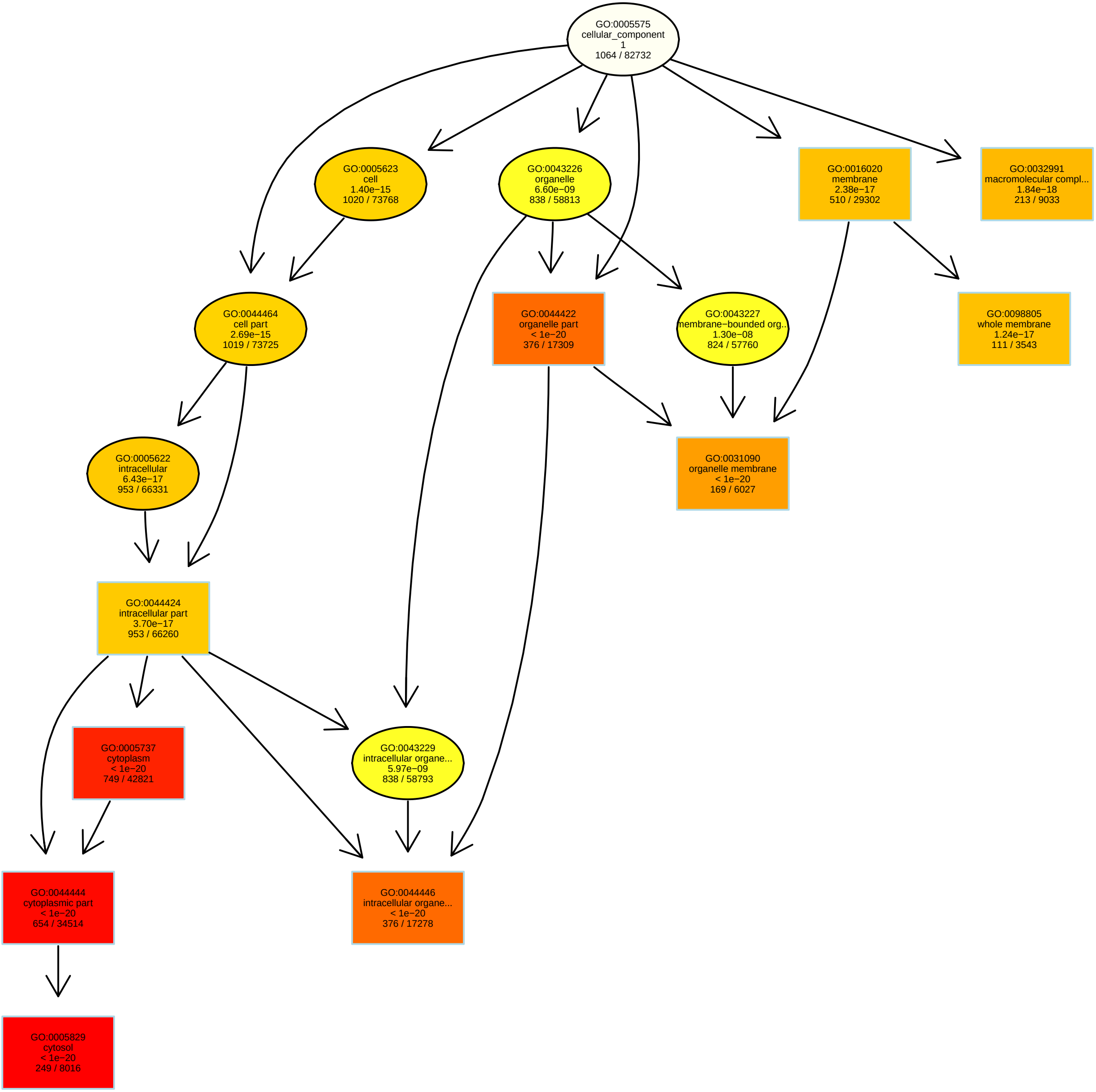
GO:0005575
cellular_component
1
1064 / 82732
GO:0005623
cell
1.40e−15
1020 / 73768
GO:0043226
organelle
6.60e−09
838 / 58813
GO:0016020
membrane
2.38e−17
510 / 29302
GO:0032991
macromolecular compl...
1.84e−18
213 / 9033
GO:0044464
cell part
2.69e−15
1019 / 73725
GO:0044422
organelle part
< 1e−20
376 / 17309
GO:0043227
membrane−bounded org...
1.30e−08
824 / 57760
GO:0098805
whole membrane
1.24e−17
111 / 3543
GO:0005622
intracellular
6.43e−17
953 / 66331
GO:0031090
organelle membrane
< 1e−20
169 / 6027
GO:0044424
intracellular part
3.70e−17
953 / 66260
GO:0005737
cytoplasm
< 1e−20
749 / 42821
GO:0043229
intracellular organe...
5.97e−09
838 / 58793
GO:0044444
cytoplasmic part
< 1e−20
654 / 34514
GO:0044446
intracellular organe...
< 1e−20
376 / 17278
GO:0005829
cytosol
< 1e−20
249 / 8016
