# Supplementary material for: Differential Alternative Splicing Genes and Isoform Regulation Networks of Rapeseed (Brassica napus L.) Infected with Sclerotinia sclerotiorum
Source: Genes (Basel). 2020 Jul 13;11(7):784. doi: 10.3390/genes11070784 (PMC7397149; doi:10.3390/genes11070784)
Supplement: Supplementary file 1 [file genes-11-00784-s001.zip › Fig. S2 Enriched GO terms of genes in the 85 WGCNA modules.zip!/Fig. S2/CytoNodeplum-go.pdf]

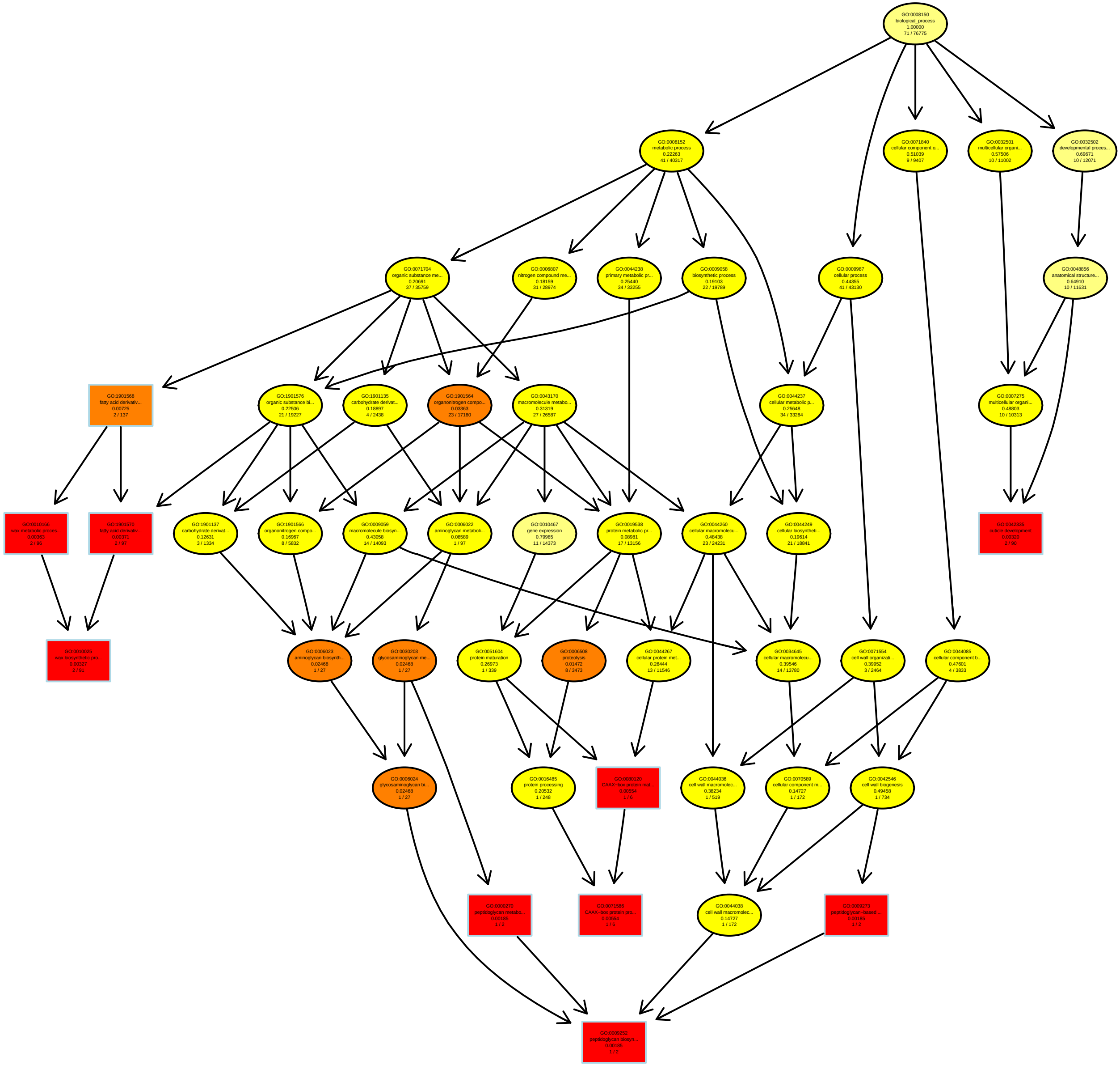

GO:0008150
biological_process
1.00000
71 / 76775
GO:0008152
metabolic process
0.22263
41 / 40317
GO:0071840
cellular component o...
0.51039
9 / 9407
GO:0032501
multicellular organi...
0.57506
10 / 11002
GO:0032502
developmental proces...
0.69671
10 / 12071
GO:0071704
organic substance me...
0.20691
37 / 35759
GO:0006807
nitrogen compound me...
0.18159
31 / 28974
GO:0044238
primary metabolic pr...
0.25440
34 / 33255
GO:0009058
biosynthetic process
0.19103
22 / 19789
GO:0009987
cellular process
0.44355
41 / 43130
GO:0048856
anatomical structure...
0.64910
10 / 11631
GO:1901568
fatty acid derivativ...
0.00725
2 / 137
GO:1901576
organic substance bi...
0.22506
21 / 19227
GO:1901135
carbohydrate derivat...
0.18897
4 / 2438
GO:1901564
organonitrogen compo...
0.03363
23 / 17180
GO:0043170
macromolecule metabo...
0.31319
27 / 26587
GO:0044237
cellular metabolic p...
0.25648
34 / 33284
GO:0007275
multicellular organi...
0.48803
10 / 10313
GO:0010166
wax metabolic proces...
0.00363
2 / 96
GO:1901570
fatty acid derivativ...
0.00371
2 / 97
GO:1901137
carbohydrate derivat...
0.12631
3 / 1334
GO:1901566
organonitrogen compo...
0.16967
8 / 5832
GO:0009059
macromolecule biosyn...
0.43058
14 / 14093
GO:0006022
aminoglycan metaboli...
0.08589
1 / 97
GO:0010467
gene expression
0.79985
11 / 14373
GO:0019538
protein metabolic pr...
0.08981
17 / 13156
GO:0044260
cellular macromolecu...
0.48438
23 / 24231
GO:0044249
cellular biosyntheti...
0.19614
21 / 18841
GO:0042335
cuticle development
0.00320
2 / 90
GO:0010025
wax biosynthetic pro...
0.00327
2 / 91
GO:0006023
aminoglycan biosynth...
0.02468
1 / 27
GO:0030203
glycosaminoglycan me...
0.02468
1 / 27
GO:0051604
protein maturation
0.26973
1 / 339
GO:0006508
proteolysis
0.01472
8 / 3473
GO:0044267
cellular protein met...
0.26444
13 / 11546
GO:0034645
cellular macromolecu...
0.39546
14 / 13780
GO:0071554
cell wall organizati...
0.39952
3 / 2464
GO:0044085
cellular component b...
0.47601
4 / 3833
GO:0006024
glycosaminoglycan bi...
0.02468
1 / 27
GO:0016485
protein processing
0.20532
1 / 248
GO:0080120
CAAX−box protein mat...
0.00554
1 / 6
GO:0044036
cell wall macromolec...
0.38234
1 / 519
GO:0070589
cellular component m...
0.14727
1 / 172
GO:0042546
cell wall biogenesis
0.49458
1 / 734
GO:0000270
peptidoglycan metabo...
0.00185
1 / 2
GO:0071586
CAAX−box protein pro...
0.00554
1 / 6
GO:0044038
cell wall macromolec...
0.14727
1 / 172
GO:0009273
peptidoglycan−based ...
0.00185
1 / 2
GO:0009252
peptidoglycan biosyn...
0.00185
1 / 2

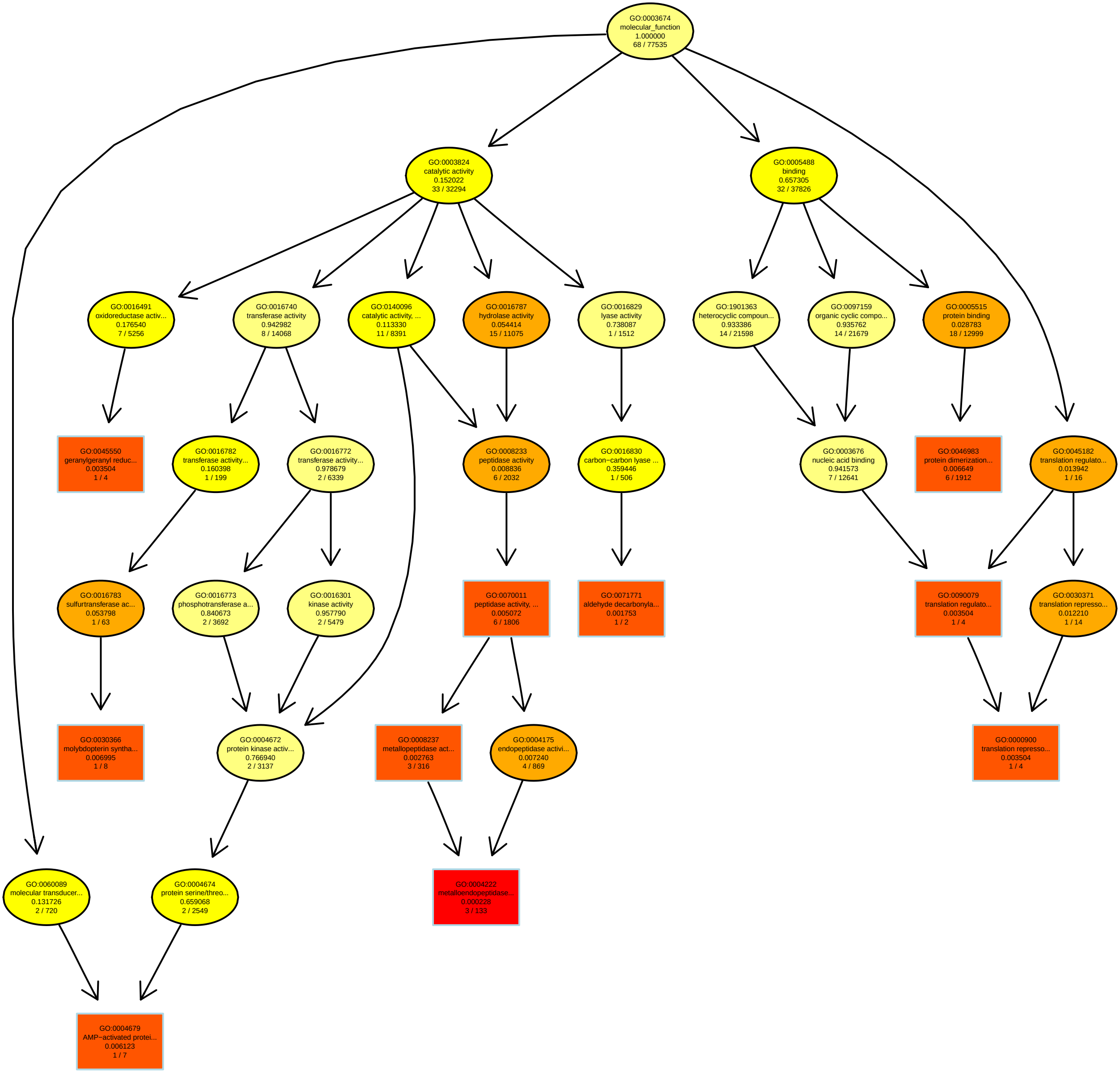
GO:0003674
molecular_function
1.000000
68 / 77535
GO:0003824
catalytic activity
0.152022
33 / 32294
GO:0005488
binding
0.657305
32 / 37826
GO:0016491
oxidoreductase activ...
0.176540
7 / 5256
GO:0016740
transferase activity
0.942982
8 / 14068
GO:0140096
catalytic activity, ...
0.113330
11 / 8391
GO:0016787
hydrolase activity
0.054414
15 / 11075
GO:0016829
lyase activity
0.738087
1 / 1512
GO:1901363
heterocyclic compoun...
0.933386
14 / 21598
GO:0097159
organic cyclic compo...
0.935762
14 / 21679
GO:0005515
protein binding
0.028783
18 / 12999
GO:0045550
geranylgeranyl reduc...
0.003504
1 / 4
GO:0016782
transferase activity...
0.160398
1 / 199
GO:0016772
transferase activity...
0.978679
2 / 6339
GO:0008233
peptidase activity
0.008836
6 / 2032
GO:0016830
carbon−carbon lyase ...
0.359446
1 / 506
GO:0003676
nucleic acid binding
0.941573
7 / 12641
GO:0046983
protein dimerization...
0.006649
6 / 1912
GO:0045182
translation regulato...
0.013942
1 / 16
GO:0016783
sulfurtransferase ac...
0.053798
1 / 63
GO:0016773
phosphotransferase a...
0.840673
2 / 3692
GO:0016301
kinase activity
0.957790
2 / 5479
GO:0070011
peptidase activity, ...
0.005072
6 / 1806
GO:0071771
aldehyde decarbonyla...
0.001753
1 / 2
GO:0090079
translation regulato...
0.003504
1 / 4
GO:0030371
translation represso...
0.012210
1 / 14
GO:0030366
molybdopterin syntha...
0.006995
1 / 8
GO:0004672
protein kinase activ...
0.766940
2 / 3137
GO:0008237
metallopeptidase act...
0.002763
3 / 316
GO:0004175
endopeptidase activi...
0.007240
4 / 869
GO:0000900
translation represso...
0.003504
1 / 4
GO:0060089
molecular transducer...
0.131726
2 / 720
GO:0004674
protein serine/threo...
0.659068
2 / 2549
GO:0004222
metalloendopeptidase...
0.000228
3 / 133
GO:0004679
AMP−activated protei...
0.006123
1 / 7

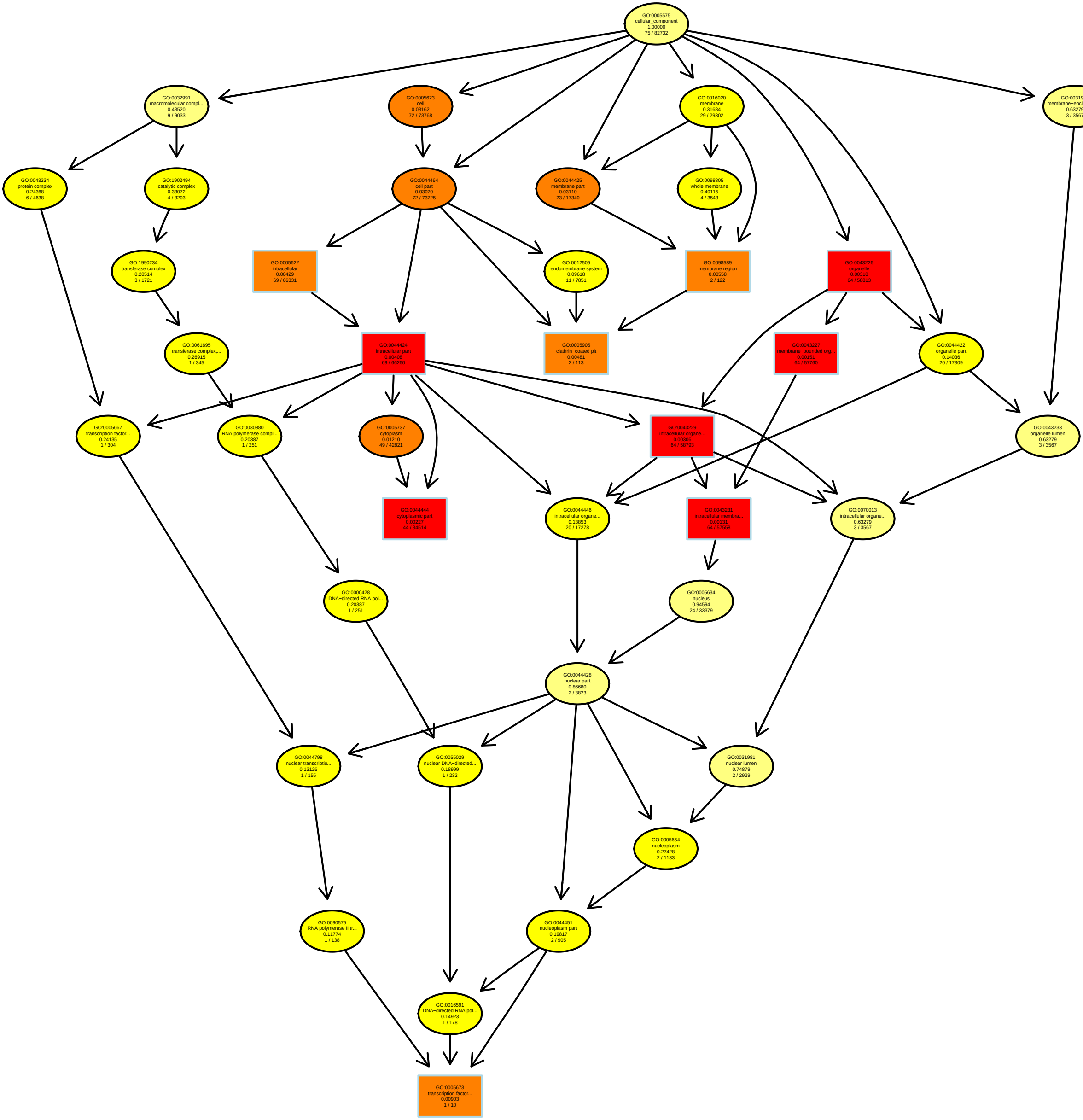

GO:0005575
cellular_component
1.00000
75 / 82732
GO:0032991
macromolecular compl...
0.43520
9 / 9033
GO:0005623
cell
0.03162
72 / 73768
GO:0016020
membrane
0.31684
29 / 29302
GO:0031974
membrane−enclosed lu...
0.63279
3 / 3567
GO:0043234
protein complex
0.24368
6 / 4638
GO:1902494
catalytic complex
0.33072
4 / 3203
GO:0044464
cell part
0.03070
72 / 73725
GO:0044425
membrane part
0.03110
23 / 17340
GO:0098805
whole membrane
0.40115
4 / 3543
GO:1990234
transferase complex
0.20514
3 / 1721
GO:0005622
intracellular
0.00429
69 / 66331
GO:0012505
endomembrane system
0.09618
11 / 7851
GO:0098589
membrane region
0.00558
2 / 122
GO:0043226
organelle
0.00310
64 / 58813
GO:0061695
transferase complex,...
0.26915
1 / 345
GO:0044424
intracellular part
0.00408
69 / 66260
GO:0005905
clathrin−coated pit
0.00481
2 / 113
GO:0043227
membrane−bounded org...
0.00151
64 / 57760
GO:0044422
organelle part
0.14036
20 / 17309
GO:0005667
transcription factor...
0.24135
1 / 304
GO:0030880
RNA polymerase compl...
0.20387
1 / 251
GO:0005737
cytoplasm
0.01210
49 / 42821
GO:0043229
intracellular organe...
0.00306
64 / 58793
GO:0043233
organelle lumen
0.63279
3 / 3567
GO:0044444
cytoplasmic part
0.00227
44 / 34514
GO:0044446
intracellular organe...
0.13853
20 / 17278
GO:0043231
intracellular membra...
0.00131
64 / 57558
GO:0070013
intracellular organe...
0.63279
3 / 3567
GO:0000428
DNA−directed RNA pol...
0.20387
1 / 251
GO:0005634
nucleus
0.94594
24 / 33379
GO:0044428
nuclear part
0.86680
2 / 3823
GO:0044798
nuclear transcriptio...
0.13126
1 / 155
GO:0055029
nuclear DNA−directed...
0.18999
1 / 232
GO:0031981
nuclear lumen
0.74879
2 / 2929
GO:0005654
nucleoplasm
0.27428
2 / 1133
GO:0090575
RNA polymerase II tr...
0.11774
1 / 138
GO:0044451
nucleoplasm part
0.19817
2 / 905
GO:0016591
DNA−directed RNA pol...
0.14923
1 / 178
GO:0005673
transcription factor...
0.00903
1 / 10
