# Supplementary material for: Differential Alternative Splicing Genes and Isoform Regulation Networks of Rapeseed (Brassica napus L.) Infected with Sclerotinia sclerotiorum
Source: Genes (Basel). 2020 Jul 13;11(7):784. doi: 10.3390/genes11070784 (PMC7397149; doi:10.3390/genes11070784)
Supplement: Supplementary file 1 [file genes-11-00784-s001.zip › Fig. S2 Enriched GO terms of genes in the 85 WGCNA modules.zip!/Fig. S2/CytoNodeplum1-go.pdf]

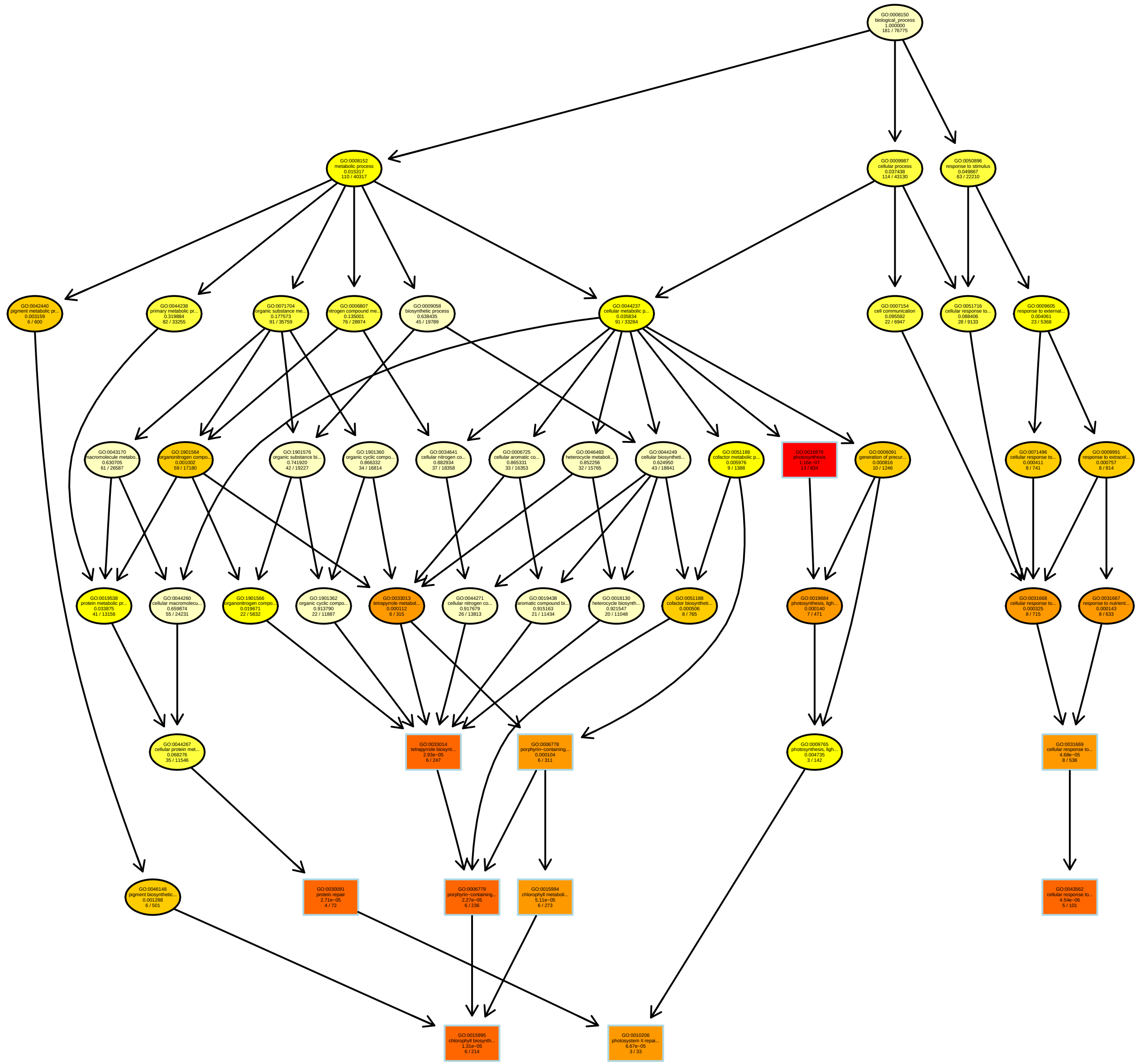

GO:0008150 biological_process 1.000000 181 / 76775
GO:0008152 metabolic process 0.015317 110 / 40317
GO:0009987 cellular process 0.037438 114 / 43130
GO:0050896 response to stimulus 0.049867 63 / 22210
GO:0042440 pigment metabolic pr... 0.003159 6 / 600
GO:0044238 primary metabolic pr... 0.319884 82 / 33255
GO:0071704 organic substance me... 0.177573 91 / 35759
GO:0006807 nitrogen compound me... 0.135001 76 / 28974
GO:0009058 biosynthetic process 0.638435 45 / 19789
GO:0044237 cellular metabolic p... 0.035834 91 / 33284
GO:0007154 cell communication 0.095592 22 / 6947
GO:0051716 cellular response to... 0.088406 28 / 9133
GO:0009605 response to external... 0.004061 23 / 5368
GO:0043170 macromolecule metabo... 0.630705 61 / 26587
GO:1901564 organonitrogen compo... 0.001002 59 / 17180
GO:1901576 organic substance bi... 0.741920 42 / 19227
GO:1901360 organic cyclic compo... 0.866332 34 / 16814
GO:0034641 cellular nitrogen co... 0.882934 37 / 18358
GO:0006725 cellular aromatic co... 0.865331 33 / 16353
GO:0046483 heterocycle metaboli... 0.852256 32 / 15765
GO:0044249 cellular biosyntheti... 0.624950 43 / 18841
GO:0051186 cofactor metabolic p... 0.005976 9 / 1388
GO:0015979 photosynthesis 1.16e−07 13 / 834
GO:0006091 generation of precur... 0.000816 10 / 1246
GO:0071496 cellular response to... 0.000411 8 / 741
GO:0009991 response to extracel... 0.000757 8 / 814
GO:0019538 protein metabolic pr... 0.033875 41 / 13156
GO:0044260 cellular macromolecu... 0.659874 55 / 24231
GO:1901566 organonitrogen compo... 0.019671 22 / 5832
GO:1901362 organic cyclic compo... 0.913790 22 / 11887
GO:0033013 tetrapyrrole metabol... 0.000112 6 / 315
GO:0044271 cellular nitrogen co... 0.917679 26 / 13813
GO:0019438 aromatic compound bi... 0.915163 21 / 11434
GO:0018130 heterocycle biosynth... 0.921547 20 / 11048
GO:0051188 cofactor biosyntheti... 0.000506 8 / 765
GO:0019684 photosynthesis, ligh... 0.000140 7 / 471
GO:0031668 cellular response to... 0.000325 8 / 715
GO:0031667 response to nutrient... 0.000143 8 / 633
GO:0044267 cellular protein met... 0.068276 35 / 11546
GO:0033014 tetrapyrrole biosynt... 2.93e−05 6 / 247
GO:0006778 porphyrin−containing... 0.000104 6 / 311
GO:0009765 photosynthesis, ligh... 0.004735 3 / 142
GO:0031669 cellular response to... 4.68e−05 8 / 538
GO:0046148 pigment biosynthetic... 0.001288 6 / 501
GO:0030091 protein repair 2.71e−05 4 / 72
GO:0006779 porphyrin−containing... 2.27e−05 6 / 236
GO:0015994 chlorophyll metaboli... 5.11e−05 6 / 273
GO:0043562 cellular response to... 4.54e−06 5 / 101
GO:0015995 chlorophyll biosynth... 1.31e−05 6 / 214
GO:0010206 photosystem II repai... 6.67e−05 3 / 33

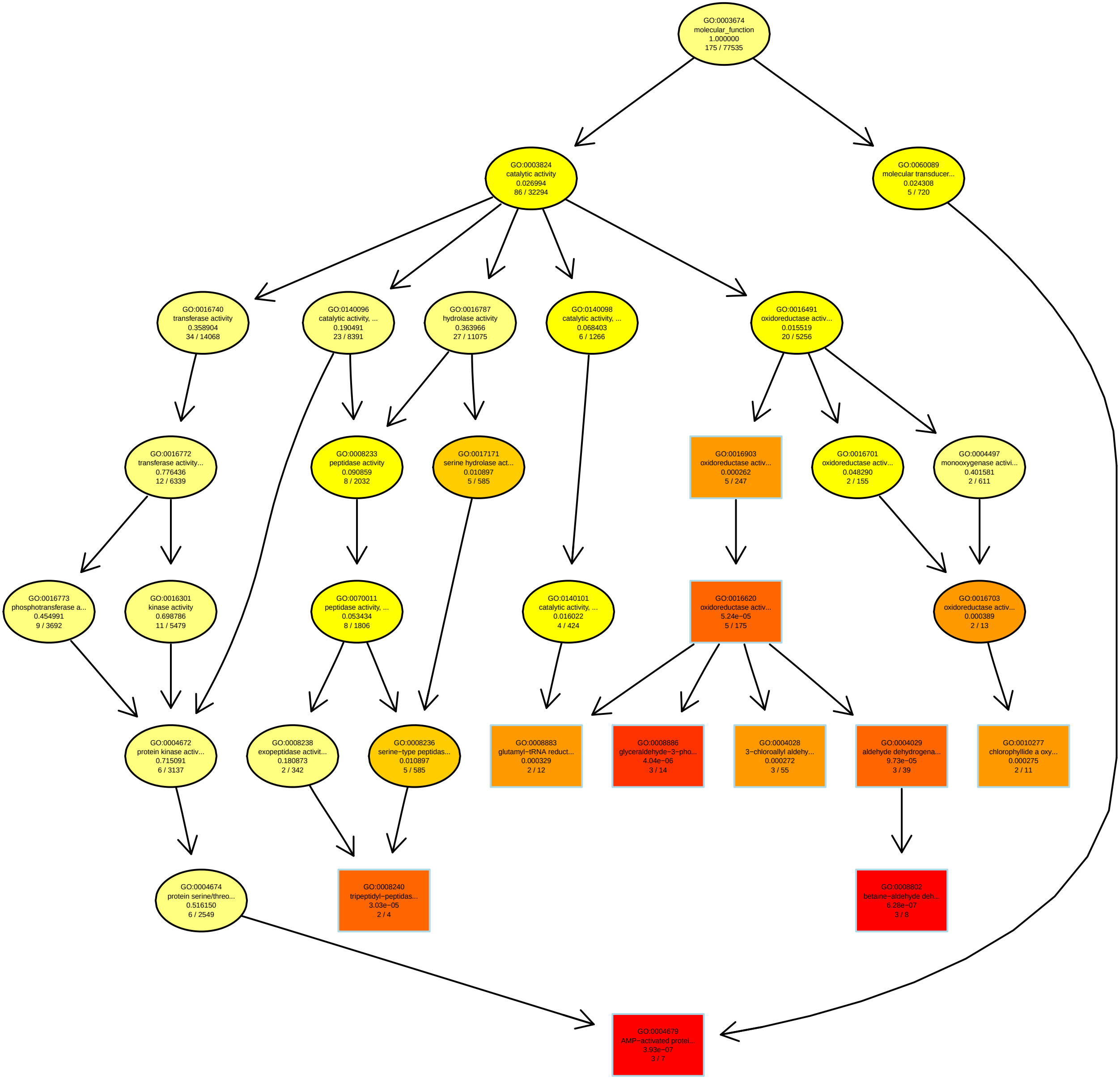
GO:0003674
molecular_function
1.000000
175 / 77535
GO:0003824
catalytic activity
0.026994
86 / 32294
GO:0060089
molecular transducer...
0.024308
5 / 720
GO:0016740
transferase activity
0.358904
34 / 14068
GO:0140096
catalytic activity, ...
0.190491
23 / 8391
GO:0016787
hydrolase activity
0.363966
27 / 11075
GO:0140098
catalytic activity, ...
0.068403
6 / 1266
GO:0016491
oxidoreductase activ...
0.015519
20 / 5256
GO:0016772
transferase activity...
0.776436
12 / 6339
GO:0008233
peptidase activity
0.090859
8 / 2032
GO:0017171
serine hydrolase act...
0.010897
5 / 585
GO:0016903
oxidoreductase activ...
0.000262
5 / 247
GO:0016701
oxidoreductase activ...
0.048290
2 / 155
GO:0004497
monooxygenase activi...
0.401581
2 / 611
GO:0016773
phosphotransferase a...
0.454991
9 / 3692
GO:0016301
kinase activity
0.698786
11 / 5479
GO:0070011
peptidase activity, ...
0.053434
8 / 1806
GO:0140101
catalytic activity, ...
0.016022
4 / 424
GO:0016620
oxidoreductase activ...
5.24e−05
5 / 175
GO:0016703
oxidoreductase activ...
0.000389
2 / 13
GO:0004672
protein kinase activ...
0.715091
6 / 3137
GO:0008238
exopeptidase activit...
0.180873
2 / 342
GO:0008236
serine−type peptidas...
0.010897
5 / 585
GO:0008883
glutamyl−tRNA reduct...
0.000329
2 / 12
GO:0008886
glyceraldehyde−3−pho...
4.04e−06
3 / 14
GO:0004028
3−chloroallyl aldehy...
0.000272
3 / 55
GO:0004029
aldehyde dehydrogena...
9.73e−05
3 / 39
GO:0010277
chlorophyllide a oxy...
0.000275
2 / 11
GO:0004674
protein serine/threo...
0.516150
6 / 2549
GO:0008240
tripeptidyl−peptidas...
3.03e−05
2 / 4
GO:0008802
betaine−aldehyde deh...
6.28e−07
3 / 8
GO:0004679
AMP−activated protei...
3.93e−07
3 / 7

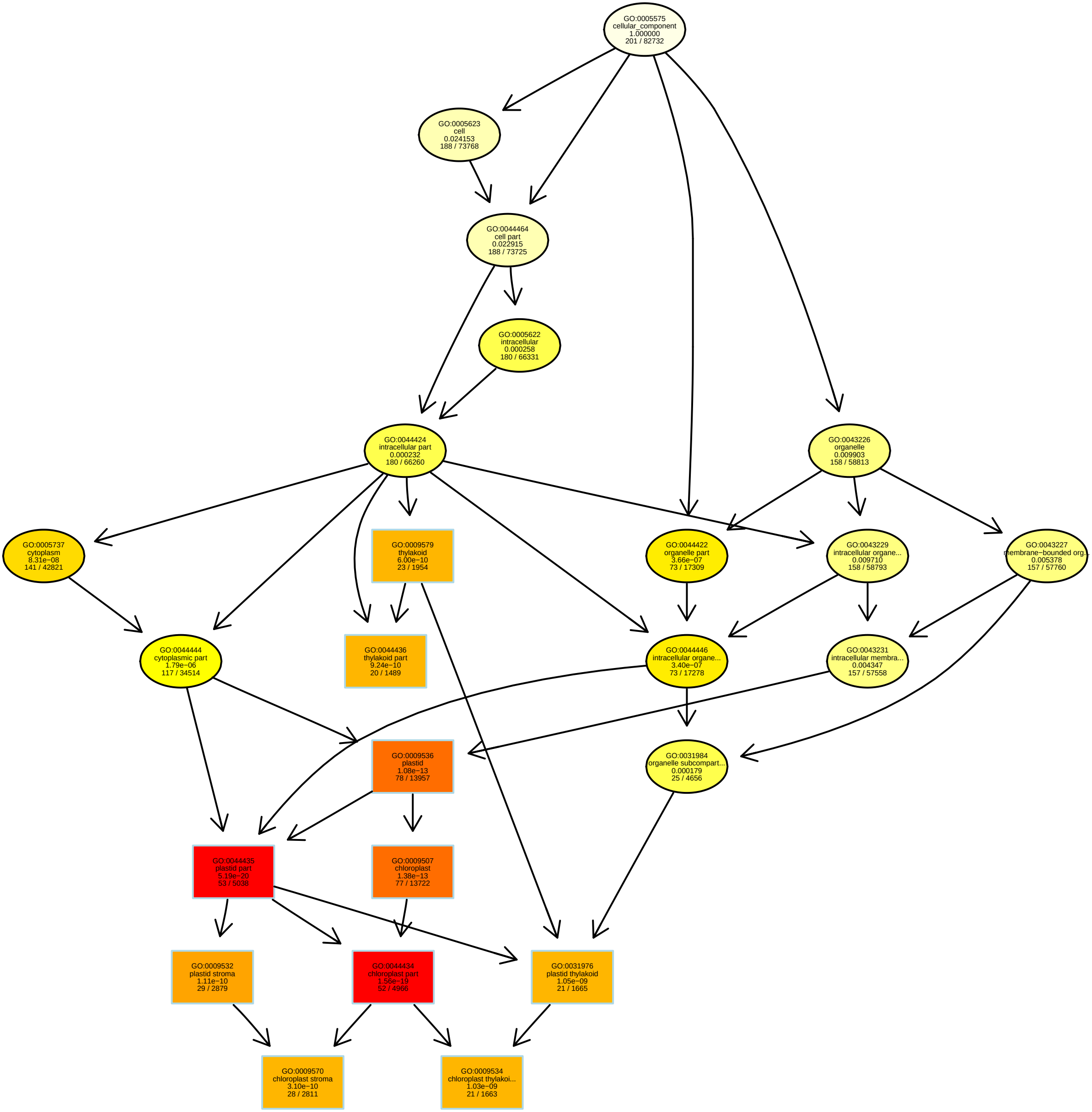

GO:0005575
cellular_component
1.000000
201 / 82732
GO:0005623
cell
0.024153
188 / 73768
GO:0044464
cell part
0.022915
188 / 73725
GO:0005622
intracellular
0.000258
180 / 66331
GO:0044424
intracellular part
0.000232
180 / 66260
GO:0043226
organelle
0.009903
158 / 58813
GO:0005737
cytoplasm
8.31e−08
141 / 42821
GO:0009579
thylakoid
6.00e−10
23 / 1954
GO:0044422
organelle part
3.66e−07
73 / 17309
GO:0043229
intracellular organe...
0.009710
158 / 58793
GO:0043227
membrane−bounded org...
0.005378
157 / 57760
GO:0044444
cytoplasmic part
1.79e−06
117 / 34514
GO:0044436
thylakoid part
9.24e−10
20 / 1489
GO:0044446
intracellular organe...
3.40e−07
73 / 17278
GO:0043231
intracellular membra...
0.004347
157 / 57558
GO:0009536
plastid
1.08e−13
78 / 13957
GO:0031984
organelle subcompart...
0.000179
25 / 4656
GO:0044435
plastid part
5.19e−20
53 / 5038
GO:0009507
chloroplast
1.38e−13
77 / 13722
GO:0009532
plastid stroma
1.11e−10
29 / 2879
GO:0044434
chloroplast part
1.56e−19
52 / 4966
GO:0031976
plastid thylakoid
1.05e−09
21 / 1665
GO:0009570
chloroplast stroma
3.10e−10
28 / 2811
GO:0009534
chloroplast thylakoi...
1.03e−09
21 / 1663
