# Supplementary material for: Differential Alternative Splicing Genes and Isoform Regulation Networks of Rapeseed (Brassica napus L.) Infected with Sclerotinia sclerotiorum
Source: Genes (Basel). 2020 Jul 13;11(7):784. doi: 10.3390/genes11070784 (PMC7397149; doi:10.3390/genes11070784)
Supplement: Supplementary file 1 [file genes-11-00784-s001.zip › Fig. S2 Enriched GO terms of genes in the 85 WGCNA modules.zip!/Fig. S2/CytoNodeplum2-go.pdf]

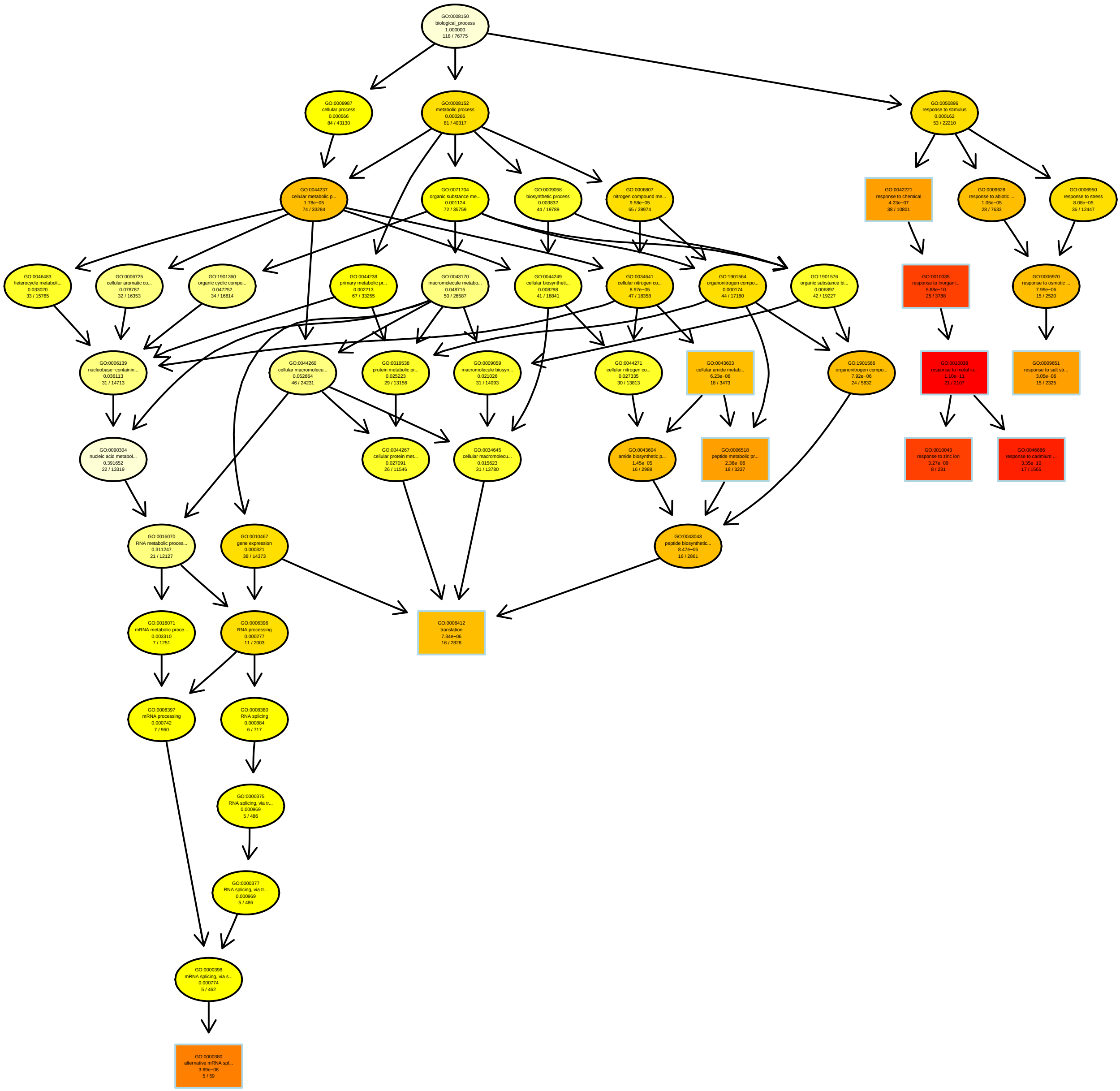
GO:0008150 biological_process 1.000000 118 / 76775
GO:0009987 cellular process 0.000566 84 / 43130
GO:0008152 metabolic process 0.000266 81 / 40317
GO:0050896 response to stimulus 0.000162 53 / 22210
GO:0044237 cellular metabolic p... 1.78e−05 74 / 33284
GO:0071704 organic substance me... 0.001124 72 / 35759
GO:0009058 biosynthetic process 0.003832 44 / 19789
GO:0006807 nitrogen compound me... 9.58e−05 65 / 28974
GO:0042221 response to chemical 4.23e−07 38 / 10801
GO:0009628 response to abiotic ... 1.05e−05 28 / 7633
GO:0006950 response to stress 8.08e−05 36 / 12447
GO:0046483 heterocycle metaboli... 0.033020 33 / 15765
GO:0006725 cellular aromatic co... 0.078787 32 / 16353
GO:1901360 organic cyclic compo... 0.047252 34 / 16814
GO:0044238 primary metabolic pr... 0.002213 67 / 33255
GO:0043170 macromolecule metabo... 0.048715 50 / 26587
GO:0044249 cellular biosyntheti... 0.008298 41 / 18841
GO:0034641 cellular nitrogen co... 8.97e−05 47 / 18358
GO:1901564 organonitrogen compo... 0.000174 44 / 17180
GO:1901576 organic substance bi... 0.006897 42 / 19227
GO:0010035 response to inorgani... 5.88e−10 25 / 3788
GO:0006970 response to osmotic ... 7.99e−06 15 / 2520
GO:0006139 nucleobase−containin... 0.036113 31 / 14713
GO:0044260 cellular macromolecu... 0.052664 46 / 24231
GO:0019538 protein metabolic pr... 0.025223 29 / 13156
GO:0009059 macromolecule biosyn... 0.021026 31 / 14093
GO:0044271 cellular nitrogen co... 0.027335 30 / 13813
GO:0043603 cellular amide metab... 6.23e−06 18 / 3473
GO:1901566 organonitrogen compo... 7.92e−06 24 / 5832
GO:0010038 response to metal io... 1.10e−11 21 / 2107
GO:0009651 response to salt str... 3.05e−06 15 / 2325
GO:0090304 nucleic acid metabol... 0.391652 22 / 13319
GO:0044267 cellular protein met... 0.027091 26 / 11546
GO:0034645 cellular macromolecu... 0.015623 31 / 13780
GO:0043604 amide biosynthetic p... 1.45e−05 16 / 2988
GO:0006518 peptide metabolic pr... 2.36e−06 18 / 3237
GO:0010043 response to zinc ion 3.27e−09 8 / 231
GO:0046686 response to cadmium ... 3.35e−10 17 / 1565
GO:0016070 RNA metabolic proces... 0.311247 21 / 12127
GO:0010467 gene expression 0.000321 38 / 14373
GO:0043043 peptide biosynthetic... 8.47e−06 16 / 2861
GO:0016071 mRNA metabolic proce... 0.003310 7 / 1251
GO:0006396 RNA processing 0.000277 11 / 2003
GO:0006412 translation 7.34e−06 16 / 2828
GO:0006397 mRNA processing 0.000742 7 / 960
GO:0008380 RNA splicing 0.000884 6 / 717
GO:0000375 RNA splicing, via tr... 0.000969 5 / 486
GO:0000377 RNA splicing, via tr... 0.000969 5 / 486
GO:0000398 mRNA splicing, via s... 0.000774 5 / 462
GO:0000380 alternative mRNA spl... 3.69e−08 5 / 59

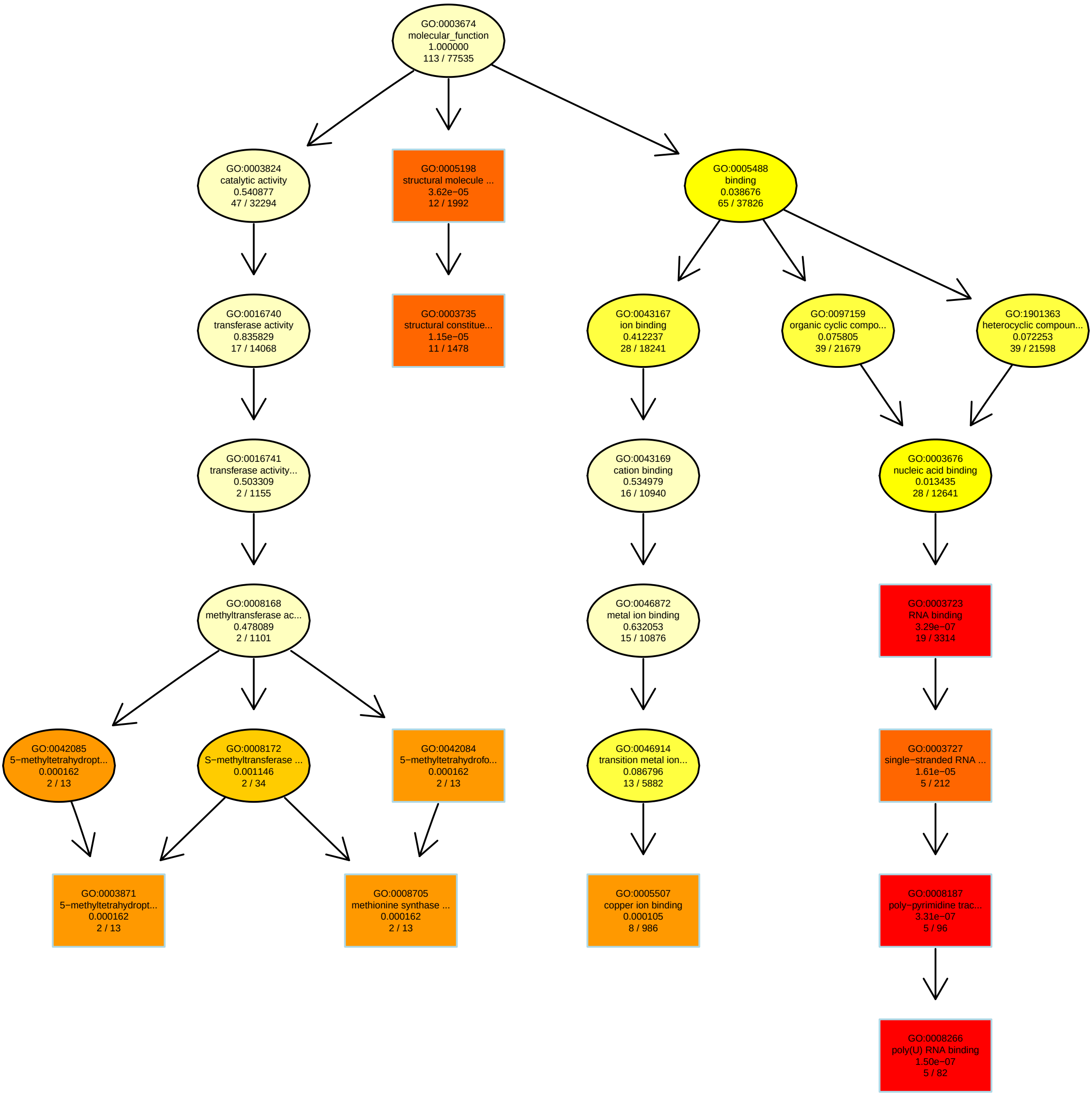
GO:0003674
molecular_function
1.000000
113 / 77535
GO:0003824
catalytic activity
0.540877
47 / 32294
GO:0005198
structural molecule ...
3.62e−05
12 / 1992
GO:0005488
binding
0.038676
65 / 37826
GO:0016740
transferase activity
0.835829
17 / 14068
GO:0003735
structural constitue...
1.15e−05
11 / 1478
GO:0043167
ion binding
0.412237
28 / 18241
GO:0097159
organic cyclic compo...
0.075805
39 / 21679
GO:1901363
heterocyclic compoun...
0.072253
39 / 21598
GO:0016741
transferase activity...
0.503309
2 / 1155
GO:0043169
cation binding
0.534979
16 / 10940
GO:0003676
nucleic acid binding
0.013435
28 / 12641
GO:0008168
methyltransferase ac...
0.478089
2 / 1101
GO:0046872
metal ion binding
0.632053
15 / 10876
GO:0003723
RNA binding
3.29e−07
19 / 3314
GO:0042085
5−methyltetrahydropt...
0.000162
2 / 13
GO:0008172
S−methyltransferase ...
0.001146
2 / 34
GO:0042084
5−methyltetrahydrofo...
0.000162
2 / 13
GO:0046914
transition metal ion...
0.086796
13 / 5882
GO:0003727
single−stranded RNA ...
1.61e−05
5 / 212
GO:0003871
5−methyltetrahydropt...
0.000162
2 / 13
GO:0008705
methionine synthase ...
0.000162
2 / 13
GO:0005507
copper ion binding
0.000105
8 / 986
GO:0008187
poly−pyrimidine trac...
3.31e−07
5 / 96
GO:0008266
poly(U) RNA binding
1.50e−07
5 / 82

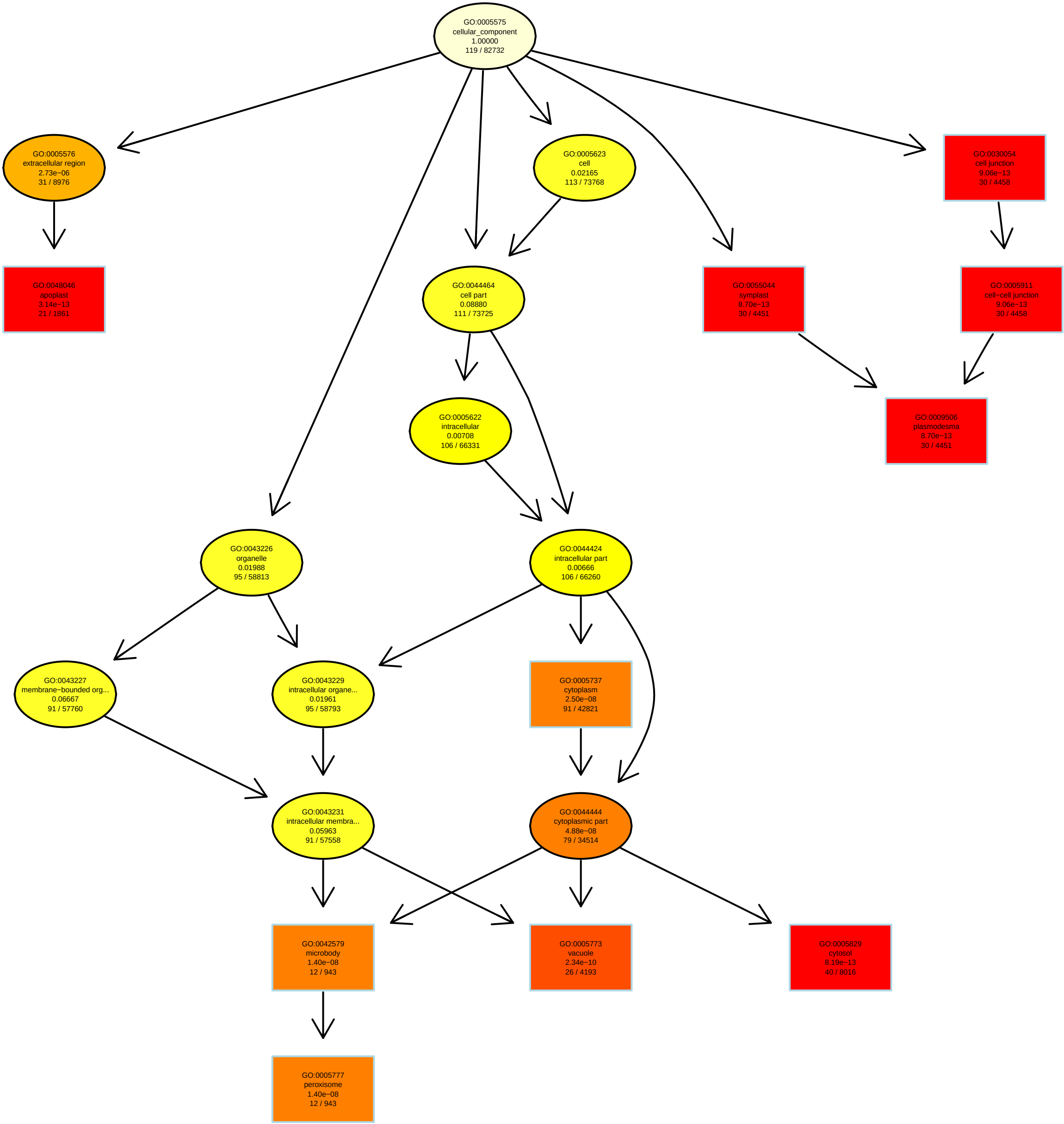
GO:0005575
cellular_component
1.00000
119 / 82732
GO:0005576
extracellular region
2.73e−06
31 / 8976
GO:0005623
cell
0.02165
113 / 73768
GO:0030054
cell junction
9.06e−13
30 / 4458
GO:0048046
apoplast
3.14e−13
21 / 1861
GO:0044464
cell part
0.08880
111 / 73725
GO:0055044
symplast
8.70e−13
30 / 4451
GO:0005911
cell−cell junction
9.06e−13
30 / 4458
GO:0005622
intracellular
0.00708
106 / 66331
GO:0009506
plasmodesma
8.70e−13
30 / 4451
GO:0043226
organelle
0.01988
95 / 58813
GO:0044424
intracellular part
0.00666
106 / 66260
GO:0043227
membrane−bounded org...
0.06667
91 / 57760
GO:0043229
intracellular organe...
0.01961
95 / 58793
GO:0005737
cytoplasm
2.50e−08
91 / 42821
GO:0043231
intracellular membra...
0.05963
91 / 57558
GO:0044444
cytoplasmic part
4.88e−08
79 / 34514
GO:0042579
microbody
1.40e−08
12 / 943
GO:0005773
vacuole
2.34e−10
26 / 4193
GO:0005829
cytosol
8.19e−13
40 / 8016
GO:0005777
peroxisome
1.40e−08
12 / 943
