# Supplementary material for: Differential Alternative Splicing Genes and Isoform Regulation Networks of Rapeseed (Brassica napus L.) Infected with Sclerotinia sclerotiorum
Source: Genes (Basel). 2020 Jul 13;11(7):784. doi: 10.3390/genes11070784 (PMC7397149; doi:10.3390/genes11070784)
Supplement: Supplementary file 1 [file genes-11-00784-s001.zip › Fig. S2 Enriched GO terms of genes in the 85 WGCNA modules.zip!/Fig. S2/CytoNodeplum3-go.pdf]

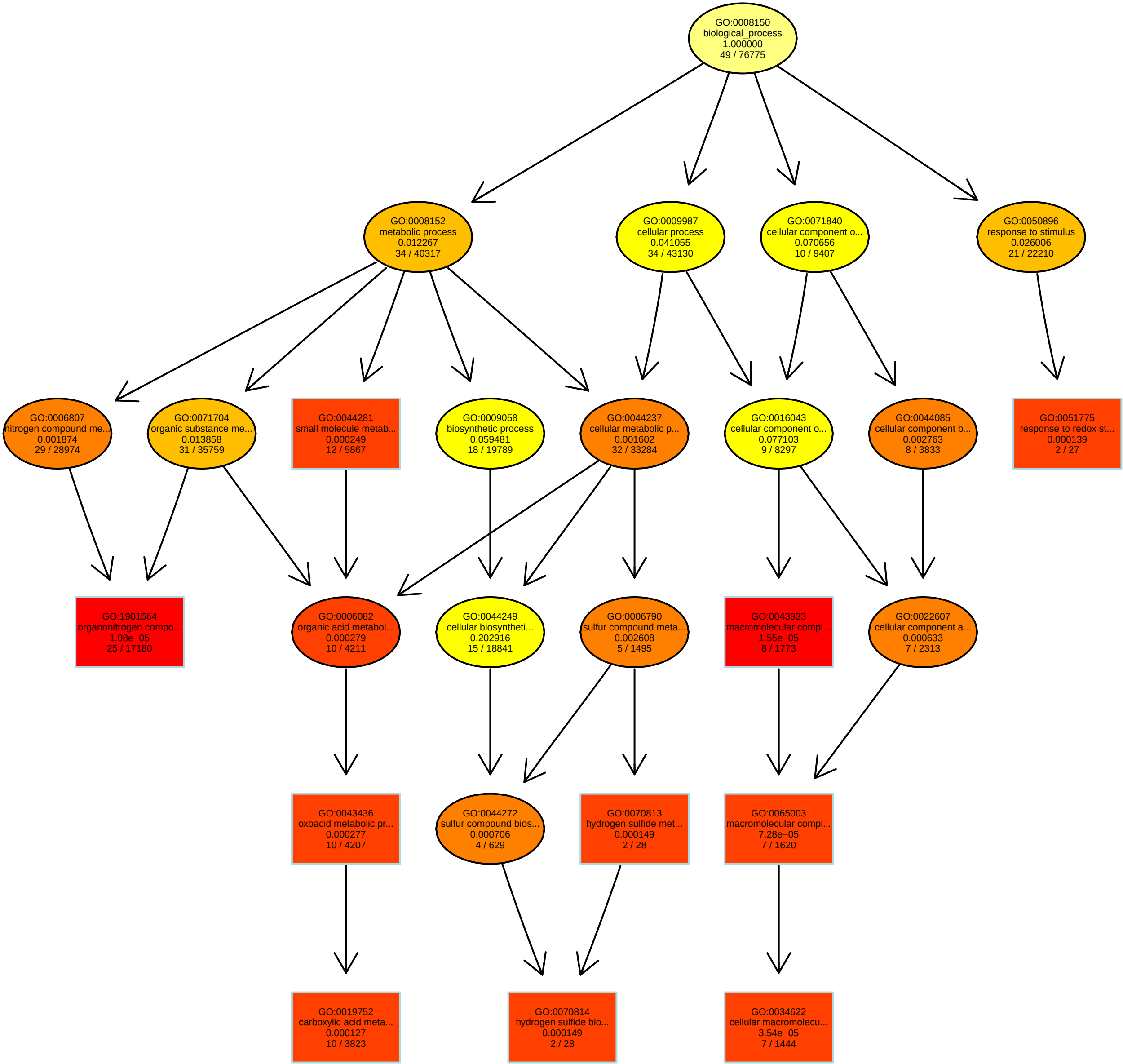
GO:0008150
biological_process
1.000000
49 / 76775
GO:0008152
metabolic process
0.012267
34 / 40317
GO:0009987
cellular process
0.041055
34 / 43130
GO:0071840
cellular component o...
0.070656
10 / 9407
GO:0050896
response to stimulus
0.026006
21 / 22210
GO:0006807
nitrogen compound me...
0.001874
29 / 28974
GO:0071704
organic substance me...
0.013858
31 / 35759
GO:0044281
small molecule metab...
0.000249
12 / 5867
GO:0009058
biosynthetic process
0.059481
18 / 19789
GO:0044237
cellular metabolic p...
0.001602
32 / 33284
GO:0016043
cellular component o...
0.077103
9 / 8297
GO:0044085
cellular component b...
0.002763
8 / 3833
GO:0051775
response to redox st...
0.000139
2 / 27
GO:1901564
organonitrogen compo...
1.08e−05
25 / 17180
GO:0006082
organic acid metabol...
0.000279
10 / 4211
GO:0044249
cellular biosyntheti...
0.202916
15 / 18841
GO:0006790
sulfur compound meta...
0.002608
5 / 1495
GO:0043933
macromolecular compl...
1.55e−05
8 / 1773
GO:0022607
cellular component a...
0.000633
7 / 2313
GO:0043436
oxoacid metabolic pr...
0.000277
10 / 4207
GO:0044272
sulfur compound bios...
0.000706
4 / 629
GO:0070813
hydrogen sulfide met...
0.000149
2 / 28
GO:0065003
macromolecular compl...
7.28e−05
7 / 1620
GO:0019752
carboxylic acid meta...
0.000127
10 / 3823
GO:0070814
hydrogen sulfide bio...
0.000149
2 / 28
GO:0034622
cellular macromolecu...
3.54e−05
7 / 1444

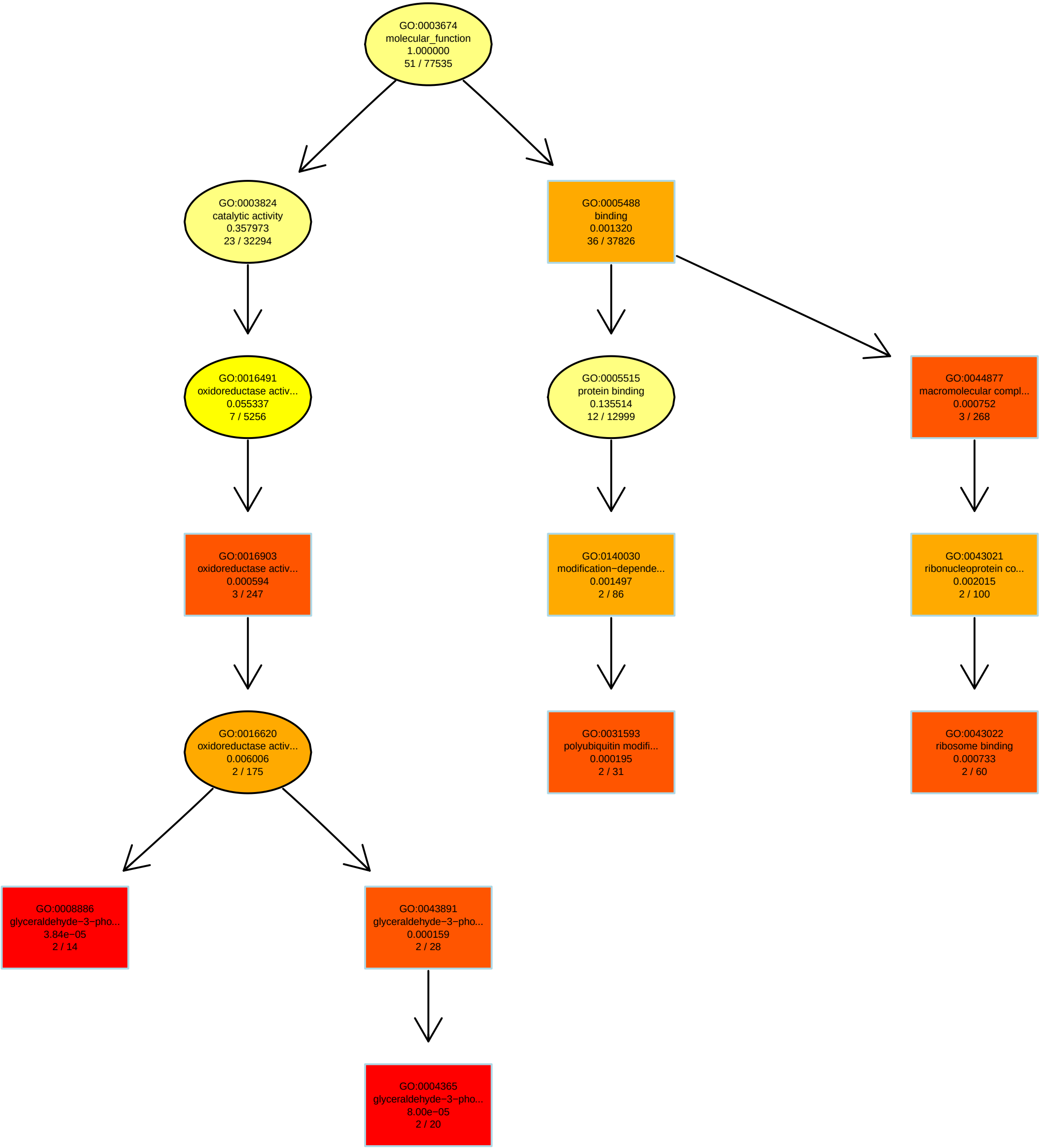
GO:0003674
molecular_function
1.000000
51 / 77535
GO:0003824
catalytic activity
0.357973
23 / 32294
GO:0005488
binding
0.001320
36 / 37826
GO:0016491
oxidoreductase activ...
0.055337
7 / 5256
GO:0005515
protein binding
0.135514
12 / 12999
GO:0044877
macromolecular compl...
0.000752
3 / 268
GO:0016903
oxidoreductase activ...
0.000594
3 / 247
GO:0140030
modification−depende...
0.001497
2 / 86
GO:0043021
ribonucleoprotein co...
0.002015
2 / 100
GO:0016620
oxidoreductase activ...
0.006006
2 / 175
GO:0031593
polyubiquitin modifi...
0.000195
2 / 31
GO:0043022
ribosome binding
0.000733
2 / 60
GO:0008886
glyceraldehyde−3−pho...
3.84e−05
2 / 14
GO:0043891
glyceraldehyde−3−pho...
0.000159
2 / 28
GO:0004365
glyceraldehyde−3−pho...
8.00e−05
2 / 20

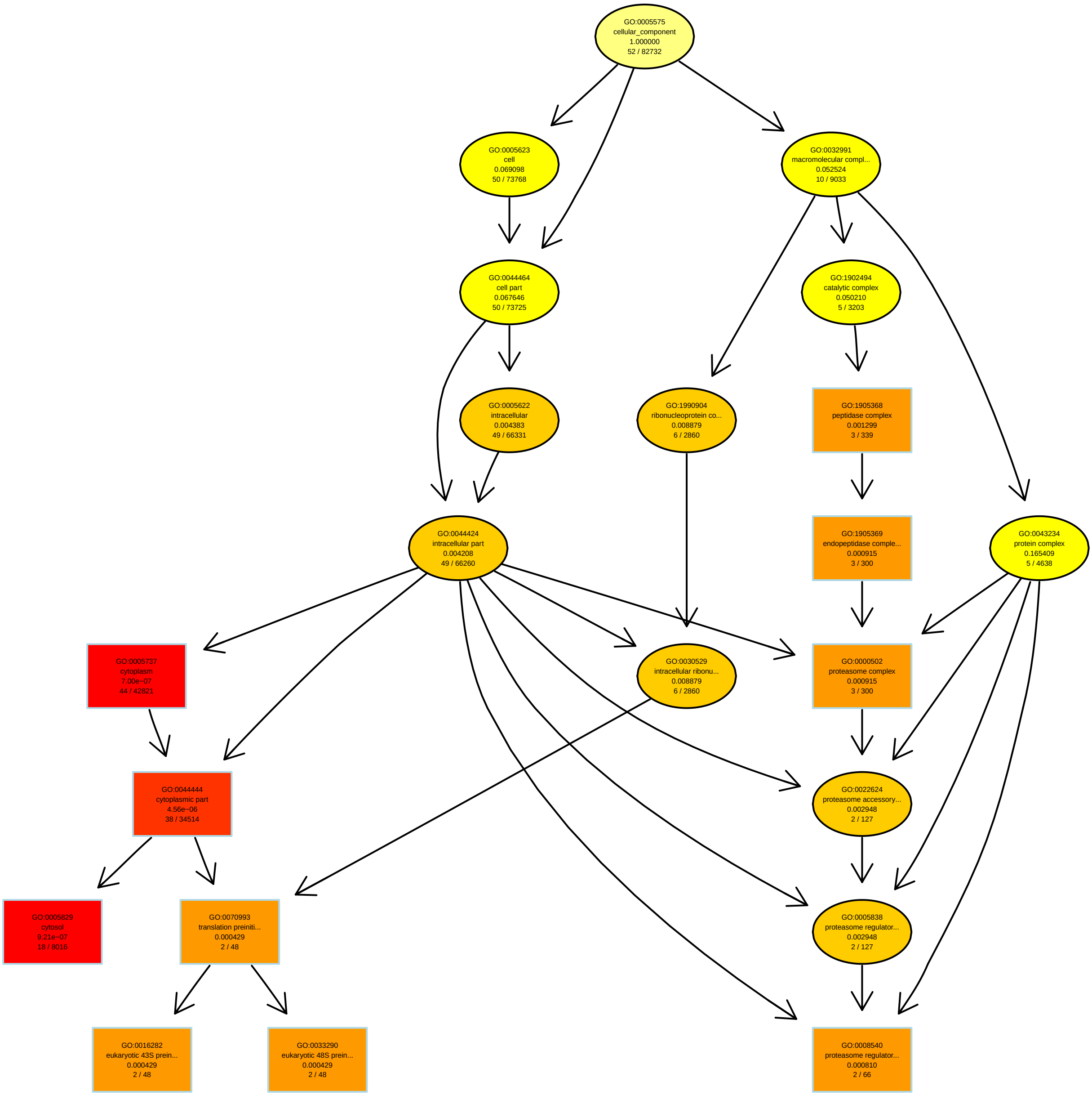

GO:0005575
cellular_component
1.000000
52 / 82732
GO:0005623
cell
0.069098
50 / 73768
GO:0032991
macromolecular compl...
0.052524
10 / 9033
GO:0044464
cell part
0.067646
50 / 73725
GO:1902494
catalytic complex
0.050210
5 / 3203
GO:0005622
intracellular
0.004383
49 / 66331
GO:1990904
ribonucleoprotein co...
0.008879
6 / 2860
GO:1905368
peptidase complex
0.001299
3 / 339
GO:0044424
intracellular part
0.004208
49 / 66260
GO:1905369
endopeptidase comple...
0.000915
3 / 300
GO:0043234
protein complex
0.165409
5 / 4638
GO:0005737
cytoplasm
7.00e−07
44 / 42821
GO:0030529
intracellular ribonu...
0.008879
6 / 2860
GO:0000502
proteasome complex
0.000915
3 / 300
GO:0044444
cytoplasmic part
4.56e−06
38 / 34514
GO:0022624
proteasome accessory...
0.002948
2 / 127
GO:0005829
cytosol
9.21e−07
18 / 8016
GO:0070993
translation preiniti...
0.000429
2 / 48
GO:0005838
proteasome regulator...
0.002948
2 / 127
GO:0016282
eukaryotic 43S prein...
0.000429
2 / 48
GO:0033290
eukaryotic 48S prein...
0.000429
2 / 48
GO:0008540
proteasome regulator...
0.000810
2 / 66
