# Supplementary material for: Differential Alternative Splicing Genes and Isoform Regulation Networks of Rapeseed (Brassica napus L.) Infected with Sclerotinia sclerotiorum
Source: Genes (Basel). 2020 Jul 13;11(7):784. doi: 10.3390/genes11070784 (PMC7397149; doi:10.3390/genes11070784)
Supplement: Supplementary file 1 [file genes-11-00784-s001.zip › Fig. S2 Enriched GO terms of genes in the 85 WGCNA modules.zip!/Fig. S2/CytoNodepurple-go.pdf]

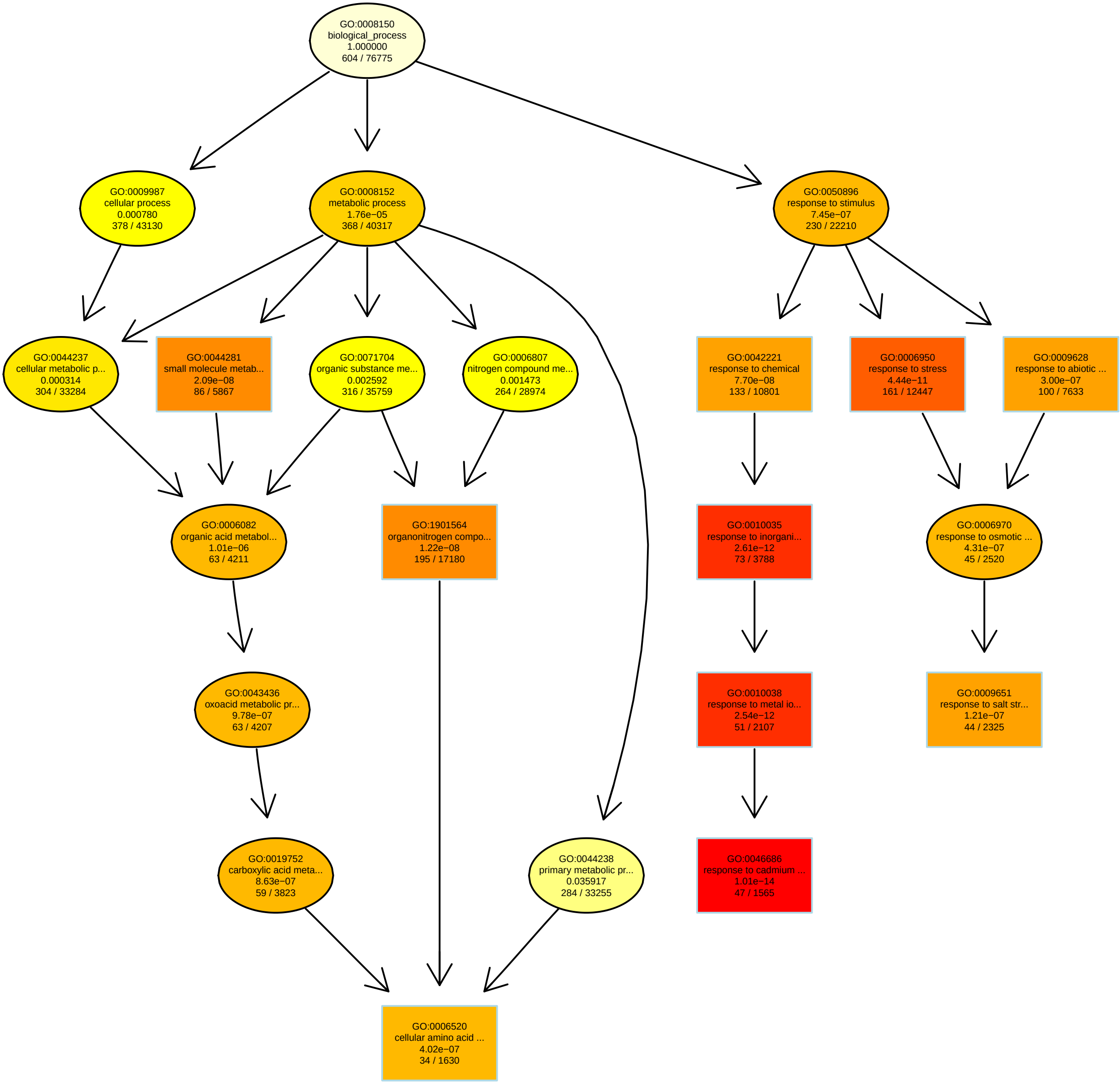
GO:0008150
biological_process
1.000000
604 / 76775
GO:0009987
cellular process
0.000780
378 / 43130
GO:0008152
metabolic process
1.76e−05
368 / 40317
GO:0050896
response to stimulus
7.45e−07
230 / 22210
GO:0044237
cellular metabolic p...
0.000314
304 / 33284
GO:0044281
small molecule metab...
2.09e−08
86 / 5867
GO:0071704
organic substance me...
0.002592
316 / 35759
GO:0006807
nitrogen compound me...
0.001473
264 / 28974
GO:0042221
response to chemical
7.70e−08
133 / 10801
GO:0006950
response to stress
4.44e−11
161 / 12447
GO:0009628
response to abiotic ...
3.00e−07
100 / 7633
GO:0006082
organic acid metabol...
1.01e−06
63 / 4211
GO:1901564
organonitrogen compo...
1.22e−08
195 / 17180
GO:0010035
response to inorgani...
2.61e−12
73 / 3788
GO:0006970
response to osmotic ...
4.31e−07
45 / 2520
GO:0043436
oxoacid metabolic pr...
9.78e−07
63 / 4207
GO:0010038
response to metal io...
2.54e−12
51 / 2107
GO:0009651
response to salt str...
1.21e−07
44 / 2325
GO:0019752
carboxylic acid meta...
8.63e−07
59 / 3823
GO:0044238
primary metabolic pr...
0.035917
284 / 33255
GO:0046686
response to cadmium ...
1.01e−14
47 / 1565
GO:0006520
cellular amino acid ...
4.02e−07
34 / 1630

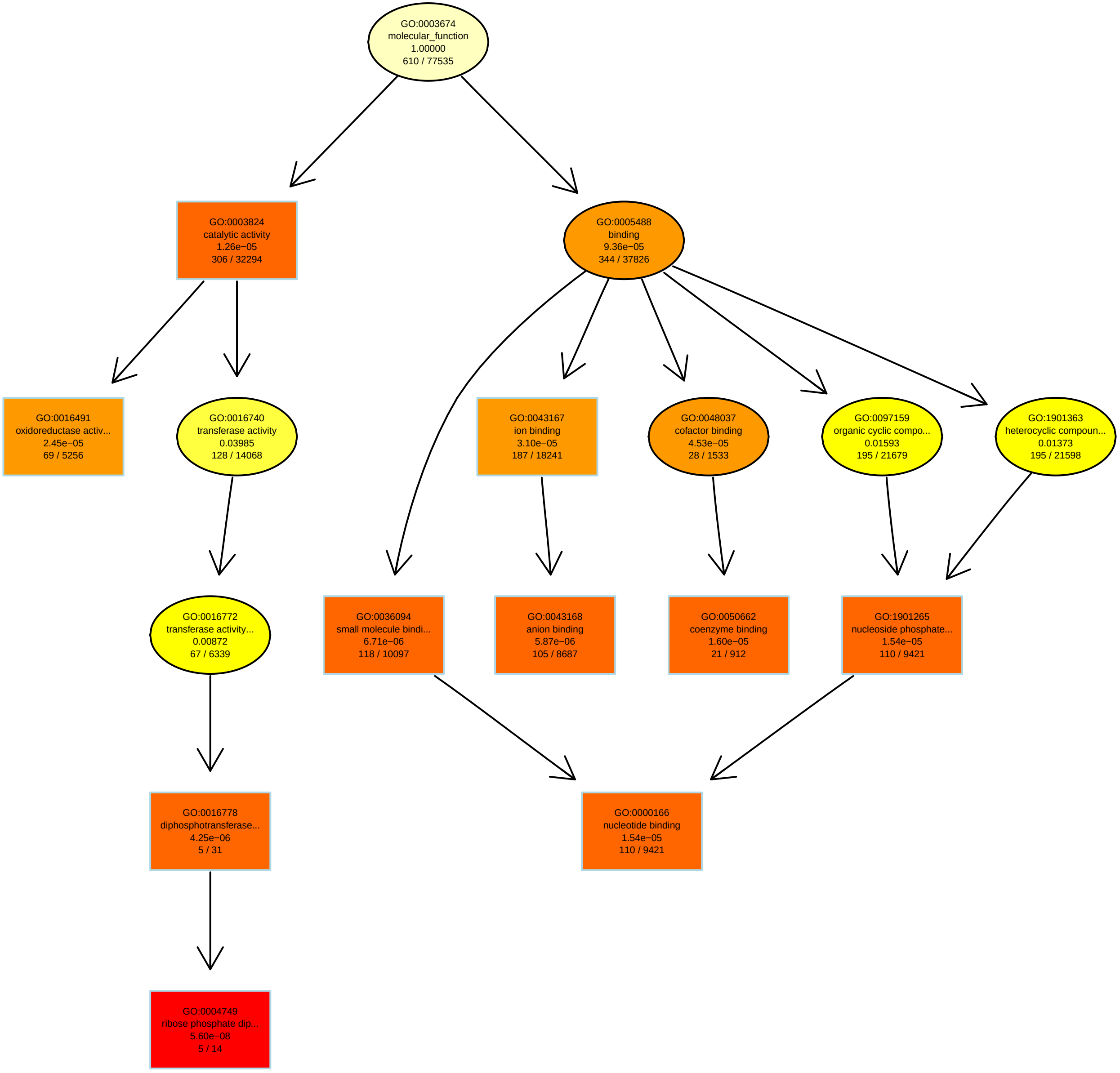
GO:0003674
molecular_function
1.00000
610 / 77535
GO:0003824
catalytic activity
1.26e−05
306 / 32294
GO:0005488
binding
9.36e−05
344 / 37826
GO:0016491
oxidoreductase activ...
2.45e−05
69 / 5256
GO:0016740
transferase activity
0.03985
128 / 14068
GO:0043167
ion binding
3.10e−05
187 / 18241
GO:0048037
cofactor binding
4.53e−05
28 / 1533
GO:0097159
organic cyclic compo...
0.01593
195 / 21679
GO:1901363
heterocyclic compoun...
0.01373
195 / 21598
GO:0016772
transferase activity...
0.00872
67 / 6339
GO:0036094
small molecule bindi...
6.71e−06
118 / 10097
GO:0043168
anion binding
5.87e−06
105 / 8687
GO:0050662
coenzyme binding
1.60e−05
21 / 912
GO:1901265
nucleoside phosphate...
1.54e−05
110 / 9421
GO:0016778
diphosphotransferase...
4.25e−06
5 / 31
GO:0000166
nucleotide binding
1.54e−05
110 / 9421
GO:0004749
ribose phosphate dip...
5.60e−08
5 / 14

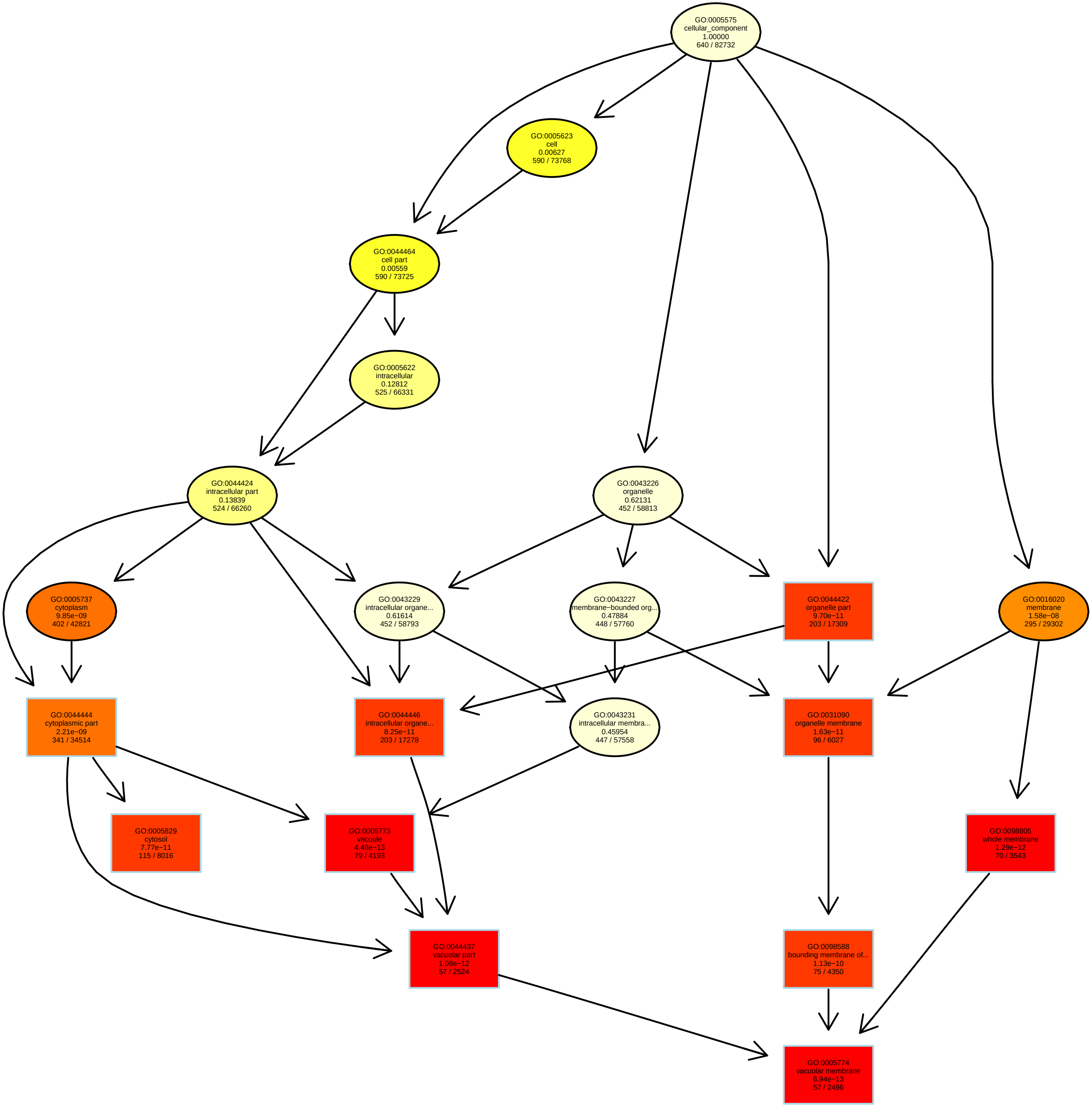

GO:0005575 cellular_component 1.00000 640 / 82732
GO:0005623 cell 0.00627 590 / 73768
GO:0044464 cell part 0.00559 590 / 73725
GO:0005622 intracellular 0.12812 525 / 66331
GO:0044424 intracellular part 0.13839 524 / 66260
GO:0043226 organelle 0.62131 452 / 58813
GO:0005737 cytoplasm 9.85e−09 402 / 42821
GO:0043229 intracellular organe... 0.61614 452 / 58793
GO:0043227 membrane−bounded org... 0.47884 448 / 57760
GO:0044422 organelle part 9.70e−11 203 / 17309
GO:0016020 membrane 1.58e−08 295 / 29302
GO:0044444 cytoplasmic part 2.21e−09 341 / 34514
GO:0044446 intracellular organe... 8.25e−11 203 / 17278
GO:0043231 intracellular membra... 0.45954 447 / 57558
GO:0031090 organelle membrane 1.63e−11 96 / 6027
GO:0005829 cytosol 7.77e−11 115 / 8016
GO:0005773 vacuole 4.46e−13 79 / 4193
GO:0098805 whole membrane 1.29e−12 70 / 3543
GO:0044437 vacuolar part 1.08e−12 57 / 2524
GO:0098588 bounding membrane of... 1.13e−10 75 / 4350
GO:0005774 vacuolar membrane 6.94e−13 57 / 2496
