# Supplementary material for: Differential Alternative Splicing Genes and Isoform Regulation Networks of Rapeseed (Brassica napus L.) Infected with Sclerotinia sclerotiorum
Source: Genes (Basel). 2020 Jul 13;11(7):784. doi: 10.3390/genes11070784 (PMC7397149; doi:10.3390/genes11070784)
Supplement: Supplementary file 1 [file genes-11-00784-s001.zip › Fig. S2 Enriched GO terms of genes in the 85 WGCNA modules.zip!/Fig. S2/CytoNodered-go.pdf]

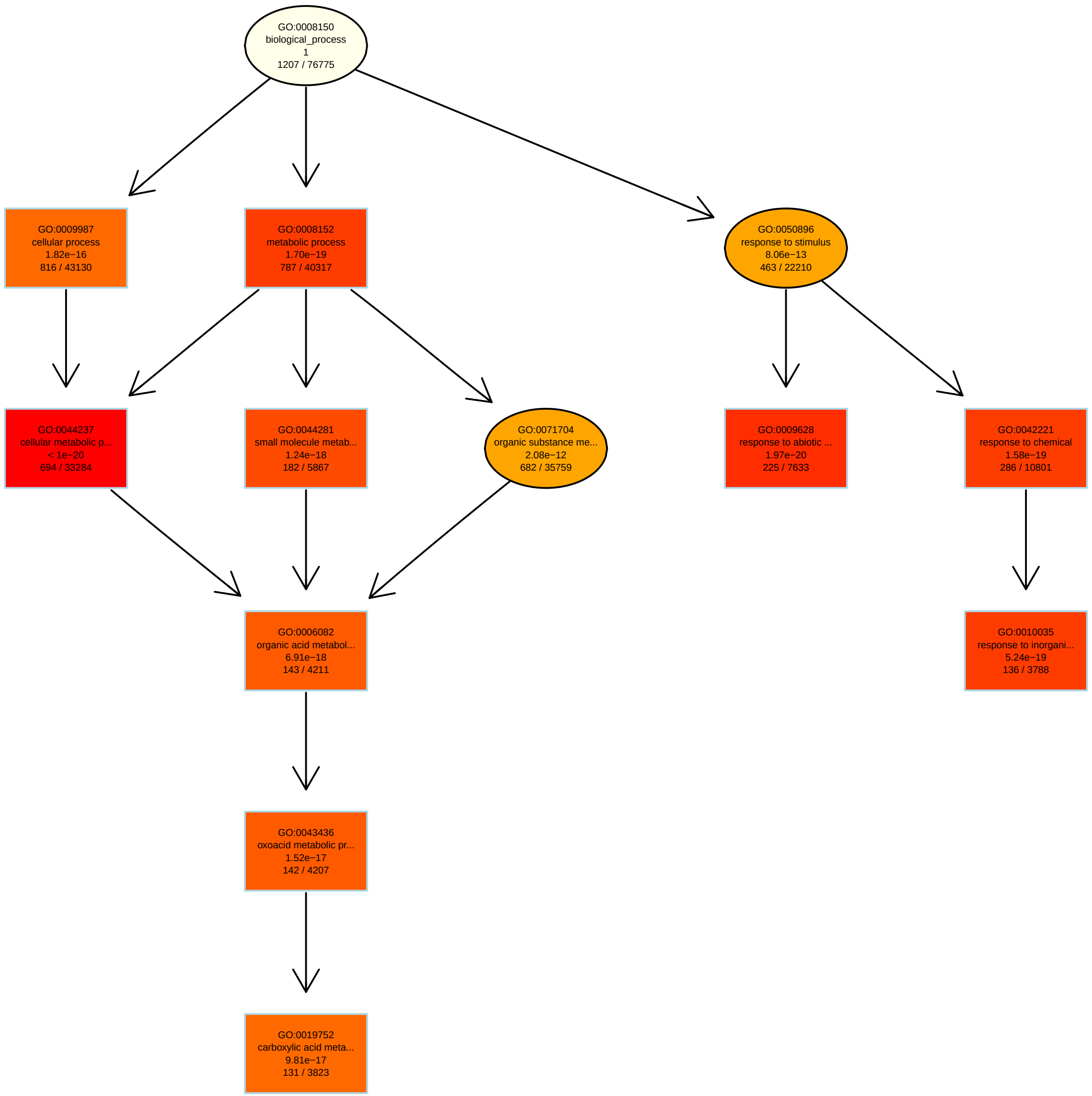

GO:0008150
biological_process
1
1207 / 76775
GO:0009987
cellular process
1.82e−16
816 / 43130
GO:0008152
metabolic process
1.70e−19
787 / 40317
GO:0050896
response to stimulus
8.06e−13
463 / 22210
GO:0044237
cellular metabolic p...
< 1e−20
694 / 33284
GO:0044281
small molecule metab...
1.24e−18
182 / 5867
GO:0071704
organic substance me...
2.08e−12
682 / 35759
GO:0009628
response to abiotic ...
1.97e−20
225 / 7633
GO:0042221
response to chemical
1.58e−19
286 / 10801
GO:0006082
organic acid metabol...
6.91e−18
143 / 4211
GO:0010035
response to inorgani...
5.24e−19
136 / 3788
GO:0043436
oxoacid metabolic pr...
1.52e−17
142 / 4207
GO:0019752
carboxylic acid meta...
9.81e−17
131 / 3823

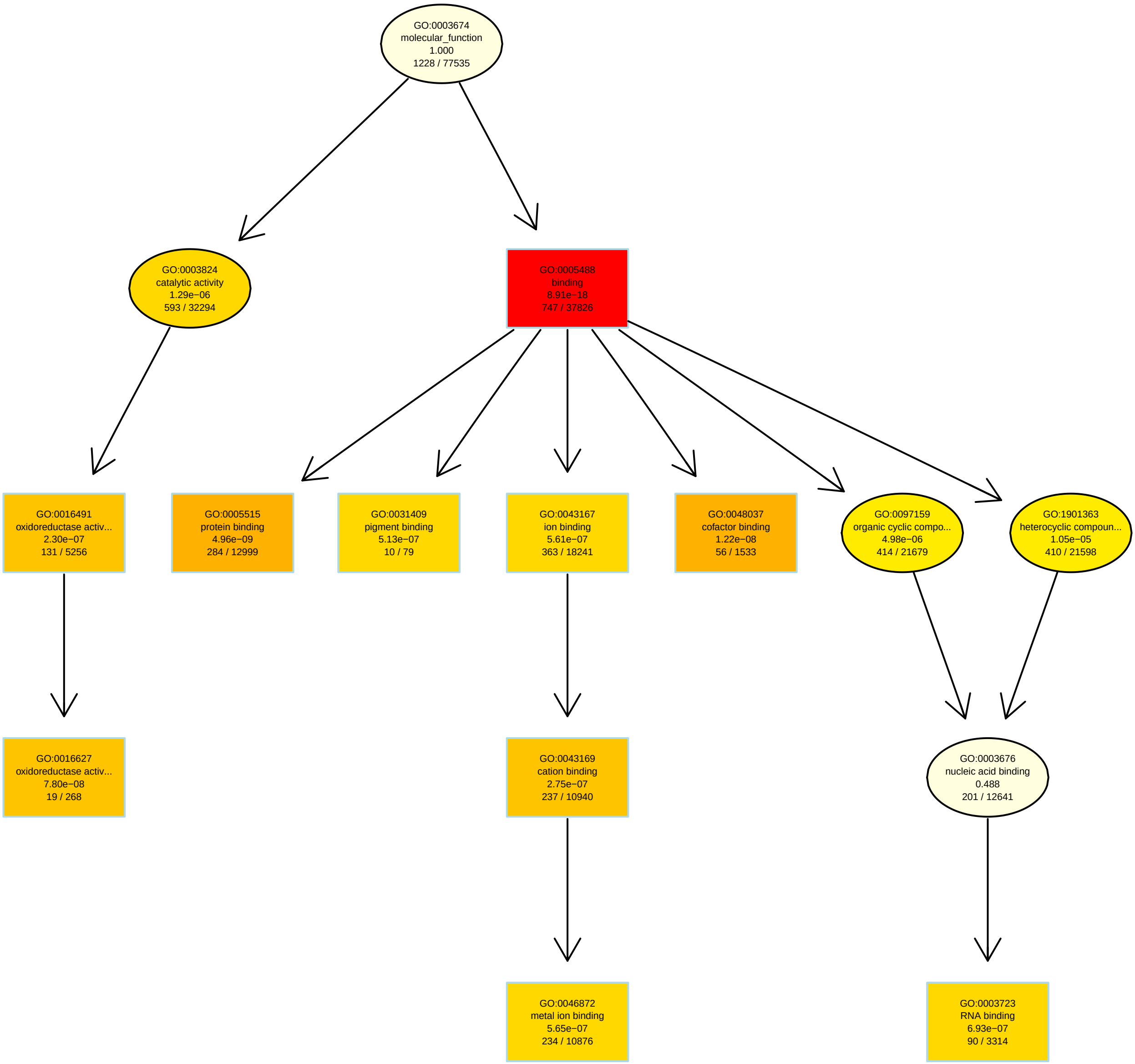
GO:0003674
molecular_function
1.000
1228 / 77535
GO:0003824
catalytic activity
1.29e−06
593 / 32294
GO:0005488
binding
8.91e−18
747 / 37826
GO:0016491
oxidoreductase activ...
2.30e−07
131 / 5256
GO:0005515
protein binding
4.96e−09
284 / 12999
GO:0031409
pigment binding
5.13e−07
10 / 79
GO:0043167
ion binding
5.61e−07
363 / 18241
GO:0048037
cofactor binding
1.22e−08
56 / 1533
GO:0097159
organic cyclic compo...
4.98e−06
414 / 21679
GO:1901363
heterocyclic compoun...
1.05e−05
410 / 21598
GO:0016627
oxidoreductase activ...
7.80e−08
19 / 268
GO:0043169
cation binding
2.75e−07
237 / 10940
GO:0003676
nucleic acid binding
0.488
201 / 12641
GO:0046872
metal ion binding
5.65e−07
234 / 10876
GO:0003723
RNA binding
6.93e−07
90 / 3314

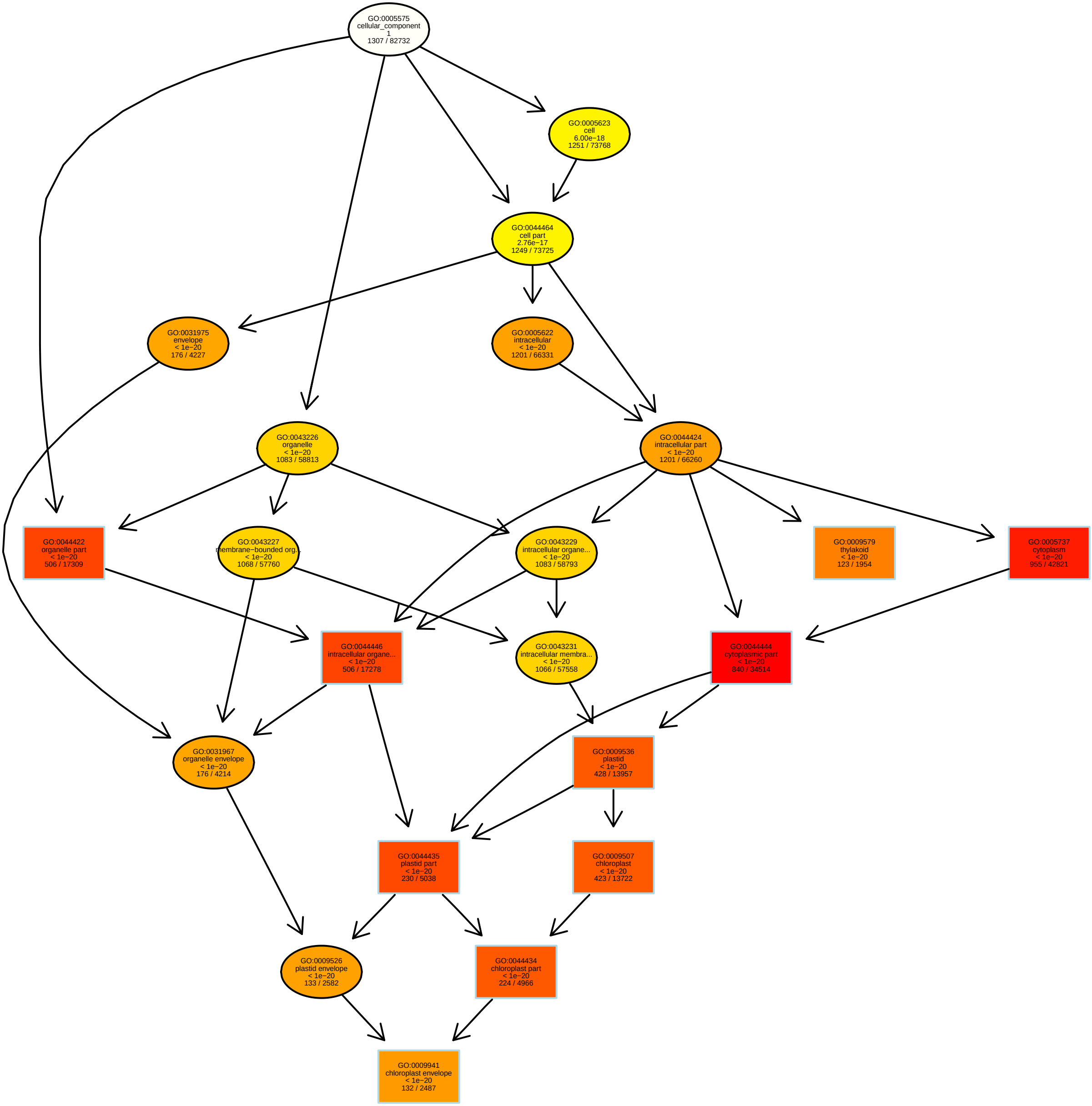
GO:0005575 cellular_component 1 1307 / 82732
GO:0005623 cell 6.00e−18 1251 / 73768
GO:0044464 cell part 2.76e−17 1249 / 73725
GO:0031975 envelope < 1e−20 176 / 4227
GO:0005622 intracellular < 1e−20 1201 / 66331
GO:0043226 organelle < 1e−20 1083 / 58813
GO:0044424 intracellular part < 1e−20 1201 / 66260
GO:0044422 organelle part < 1e−20 506 / 17309
GO:0043227 membrane−bounded org... < 1e−20 1068 / 57760
GO:0043229 intracellular organe... < 1e−20 1083 / 58793
GO:0009579 thylakoid < 1e−20 123 / 1954
GO:0005737 cytoplasm < 1e−20 955 / 42821
GO:0044446 intracellular organe... < 1e−20 506 / 17278
GO:0043231 intracellular membra... < 1e−20 1066 / 57558
GO:0044444 cytoplasmic part < 1e−20 840 / 34514
GO:0031967 organelle envelope < 1e−20 176 / 4214
GO:0009536 plastid < 1e−20 428 / 13957
GO:0044435 plastid part < 1e−20 230 / 5038
GO:0009507 chloroplast < 1e−20 423 / 13722
GO:0009526 plastid envelope < 1e−20 133 / 2582
GO:0044434 chloroplast part < 1e−20 224 / 4966
GO:0009941 chloroplast envelope < 1e−20 132 / 2487
