# Supplementary material for: Differential Alternative Splicing Genes and Isoform Regulation Networks of Rapeseed (Brassica napus L.) Infected with Sclerotinia sclerotiorum
Source: Genes (Basel). 2020 Jul 13;11(7):784. doi: 10.3390/genes11070784 (PMC7397149; doi:10.3390/genes11070784)
Supplement: Supplementary file 1 [file genes-11-00784-s001.zip › Fig. S2 Enriched GO terms of genes in the 85 WGCNA modules.zip!/Fig. S2/CytoNoderoyalblue-go.pdf]

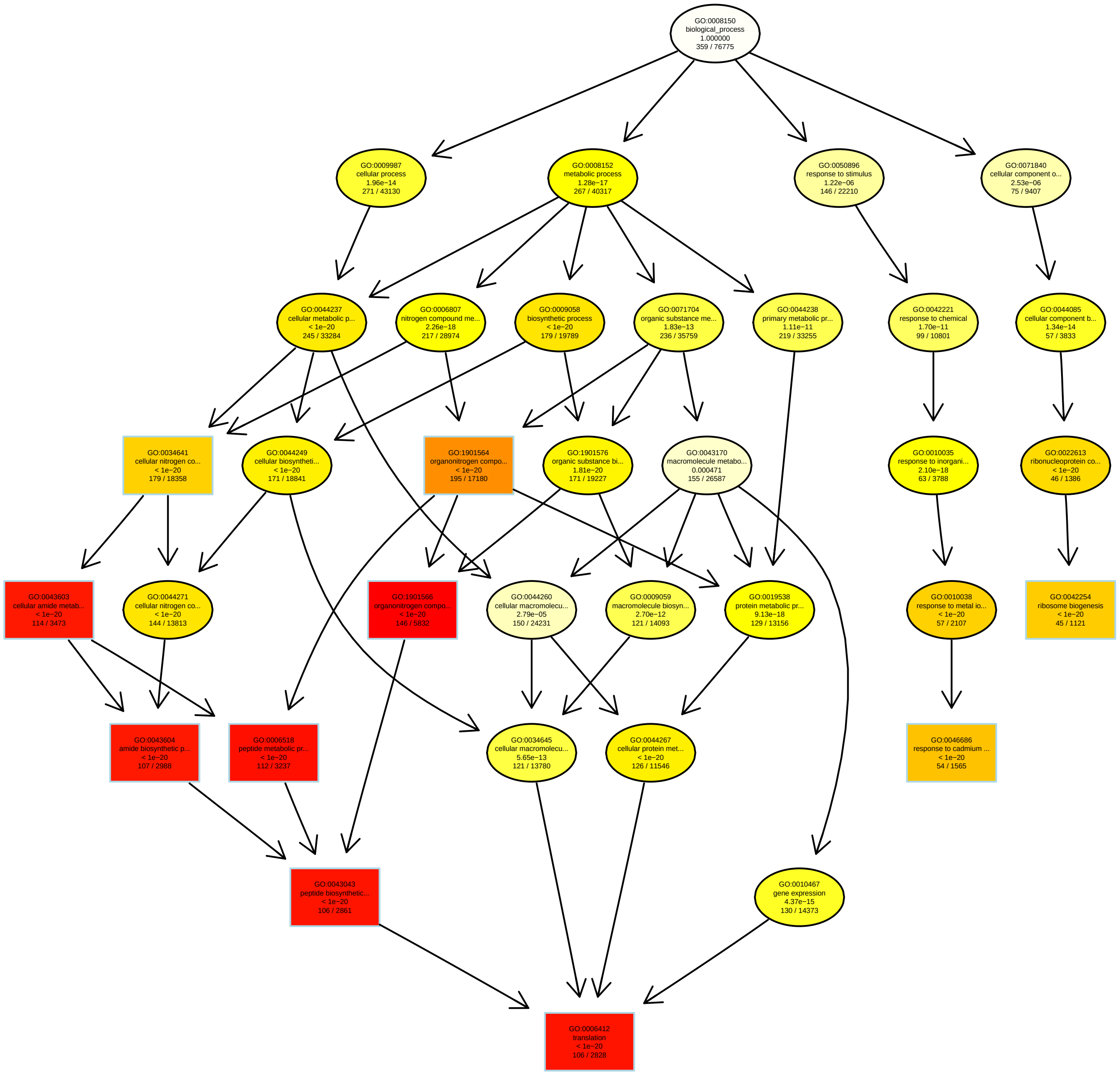
GO:0008150 biological_process 1.000000 359 / 76775
GO:0009987 cellular process 1.96e−14 271 / 43130
GO:0008152 metabolic process 1.28e−17 267 / 40317
GO:0050896 response to stimulus 1.22e−06 146 / 22210
GO:0071840 cellular component o... 2.53e−06 75 / 9407
GO:0044237 cellular metabolic p... < 1e−20 245 / 33284
GO:0006807 nitrogen compound me... 2.26e−18 217 / 28974
GO:0009058 biosynthetic process < 1e−20 179 / 19789
GO:0071704 organic substance me... 1.83e−13 236 / 35759
GO:0044238 primary metabolic pr... 1.11e−11 219 / 33255
GO:0042221 response to chemical 1.70e−11 99 / 10801
GO:0044085 cellular component b... 1.34e−14 57 / 3833
GO:0034641 cellular nitrogen co... < 1e−20 179 / 18358
GO:0044249 cellular biosyntheti... < 1e−20 171 / 18841
GO:1901564 organonitrogen compo... < 1e−20 195 / 17180
GO:1901576 organic substance bi... 1.81e−20 171 / 19227
GO:0043170 macromolecule metabo... 0.000471 155 / 26587
GO:0010035 response to inorgani... 2.10e−18 63 / 3788
GO:0022613 ribonucleoprotein co... < 1e−20 46 / 1386
GO:0043603 cellular amide metab... < 1e−20 114 / 3473
GO:0044271 cellular nitrogen co... < 1e−20 144 / 13813
GO:1901566 organonitrogen compo... < 1e−20 146 / 5832
GO:0044260 cellular macromolecu... 2.79e−05 150 / 24231
GO:0009059 macromolecule biosyn... 2.70e−12 121 / 14093
GO:0019538 protein metabolic pr... 9.13e−18 129 / 13156
GO:0010038 response to metal io... < 1e−20 57 / 2107
GO:0042254 ribosome biogenesis < 1e−20 45 / 1121
GO:0043604 amide biosynthetic p... < 1e−20 107 / 2988
GO:0006518 peptide metabolic pr... < 1e−20 112 / 3237
GO:0034645 cellular macromolecu... 5.65e−13 121 / 13780
GO:0044267 cellular protein met... < 1e−20 126 / 11546
GO:0046686 response to cadmium ... < 1e−20 54 / 1565
GO:0043043 peptide biosynthetic... < 1e−20 106 / 2861
GO:0010467 gene expression 4.37e−15 130 / 14373
GO:0006412 translation < 1e−20 106 / 2828

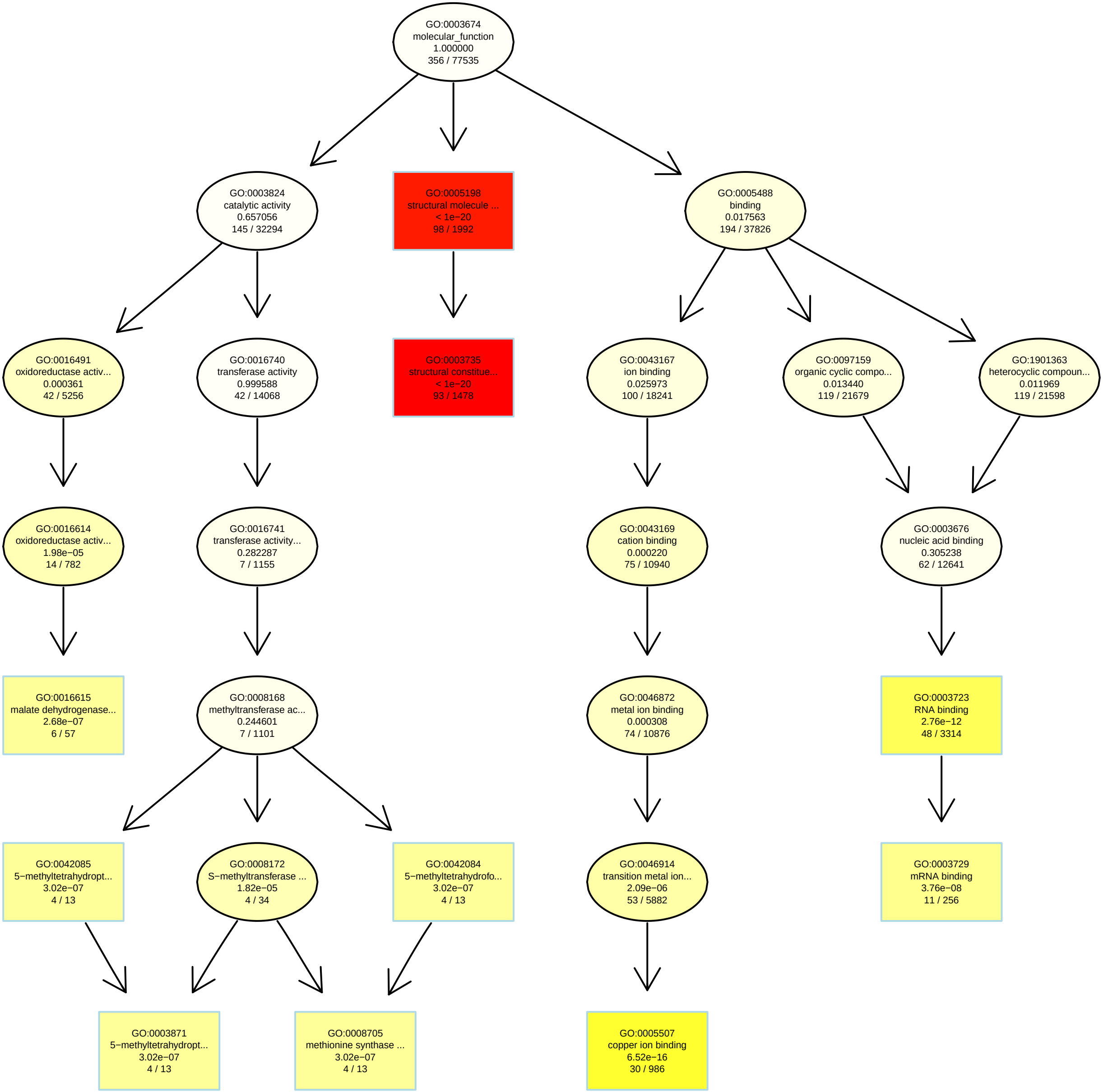
GO:0003674 molecular_function 1.000000 356 / 77535
GO:0003824 catalytic activity 0.657056 145 / 32294
GO:0005198 structural molecule ... < 1e−20 98 / 1992
GO:0005488 binding 0.017563 194 / 37826
GO:0016491 oxidoreductase activ... 0.000361 42 / 5256
GO:0016740 transferase activity 0.999588 42 / 14068
GO:0003735 structural constitue... < 1e−20 93 / 1478
GO:0043167 ion binding 0.025973 100 / 18241
GO:0097159 organic cyclic compo... 0.013440 119 / 21679
GO:1901363 heterocyclic compoun... 0.011969 119 / 21598
GO:0016614 oxidoreductase activ... 1.98e−05 14 / 782
GO:0016741 transferase activity... 0.282287 7 / 1155
GO:0043169 cation binding 0.000220 75 / 10940
GO:0003676 nucleic acid binding 0.305238 62 / 12641
GO:0016615 malate dehydrogenase... 2.68e−07 6 / 57
GO:0008168 methyltransferase ac... 0.244601 7 / 1101
GO:0046872 metal ion binding 0.000308 74 / 10876
GO:0003723 RNA binding 2.76e−12 48 / 3314
GO:0042085 5−methyltetrahydropt... 3.02e−07 4 / 13
GO:0008172 S−methyltransferase ... 1.82e−05 4 / 34
GO:0042084 5−methyltetrahydrofo... 3.02e−07 4 / 13
GO:0046914 transition metal ion... 2.09e−06 53 / 5882
GO:0003729 mRNA binding 3.76e−08 11 / 256
GO:0003871 5−methyltetrahydropt... 3.02e−07 4 / 13
GO:0008705 methionine synthase ... 3.02e−07 4 / 13
GO:0005507 copper ion binding 6.52e−16 30 / 986

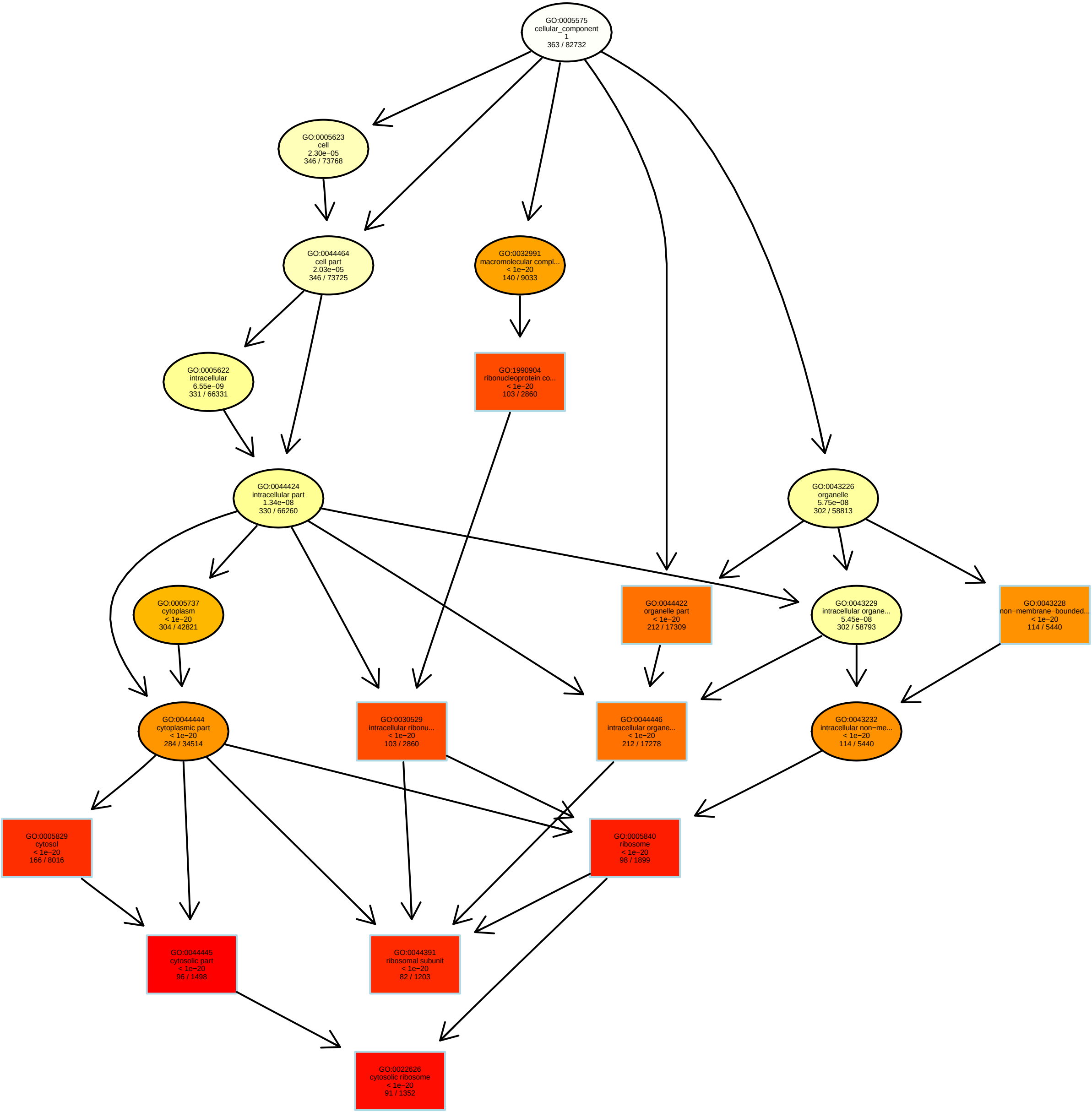

GO:0005575
cellular_component
1
363 / 82732
GO:0005623
cell
2.30e−05
346 / 73768
GO:0044464
cell part
2.03e−05
346 / 73725
GO:0032991
macromolecular compl...
< 1e−20
140 / 9033
GO:1990904
ribonucleoprotein co...
< 1e−20
103 / 2860
GO:0005622
intracellular
6.55e−09
331 / 66331
GO:0044424
intracellular part
1.34e−08
330 / 66260
GO:0043226
organelle
5.75e−08
302 / 58813
GO:0005737
cytoplasm
< 1e−20
304 / 42821
GO:0044422
organelle part
< 1e−20
212 / 17309
GO:0043229
intracellular organe...
5.45e−08
302 / 58793
GO:0043228
non−membrane−bounded...
< 1e−20
114 / 5440
GO:0044444
cytoplasmic part
< 1e−20
284 / 34514
GO:0030529
intracellular ribonu...
< 1e−20
103 / 2860
GO:0044446
intracellular organe...
< 1e−20
212 / 17278
GO:0043232
intracellular non−me...
< 1e−20
114 / 5440
GO:0005829
cytosol
< 1e−20
166 / 8016
GO:0005840
ribosome
< 1e−20
98 / 1899
GO:0044445
cytosolic part
< 1e−20
96 / 1498
GO:0044391
ribosomal subunit
< 1e−20
82 / 1203
GO:0022626
cytosolic ribosome
< 1e−20
91 / 1352
