# Supplementary material for: Differential Alternative Splicing Genes and Isoform Regulation Networks of Rapeseed (Brassica napus L.) Infected with Sclerotinia sclerotiorum
Source: Genes (Basel). 2020 Jul 13;11(7):784. doi: 10.3390/genes11070784 (PMC7397149; doi:10.3390/genes11070784)
Supplement: Supplementary file 1 [file genes-11-00784-s001.zip › Fig. S2 Enriched GO terms of genes in the 85 WGCNA modules.zip!/Fig. S2/CytoNodesaddlebrown-go.pdf]

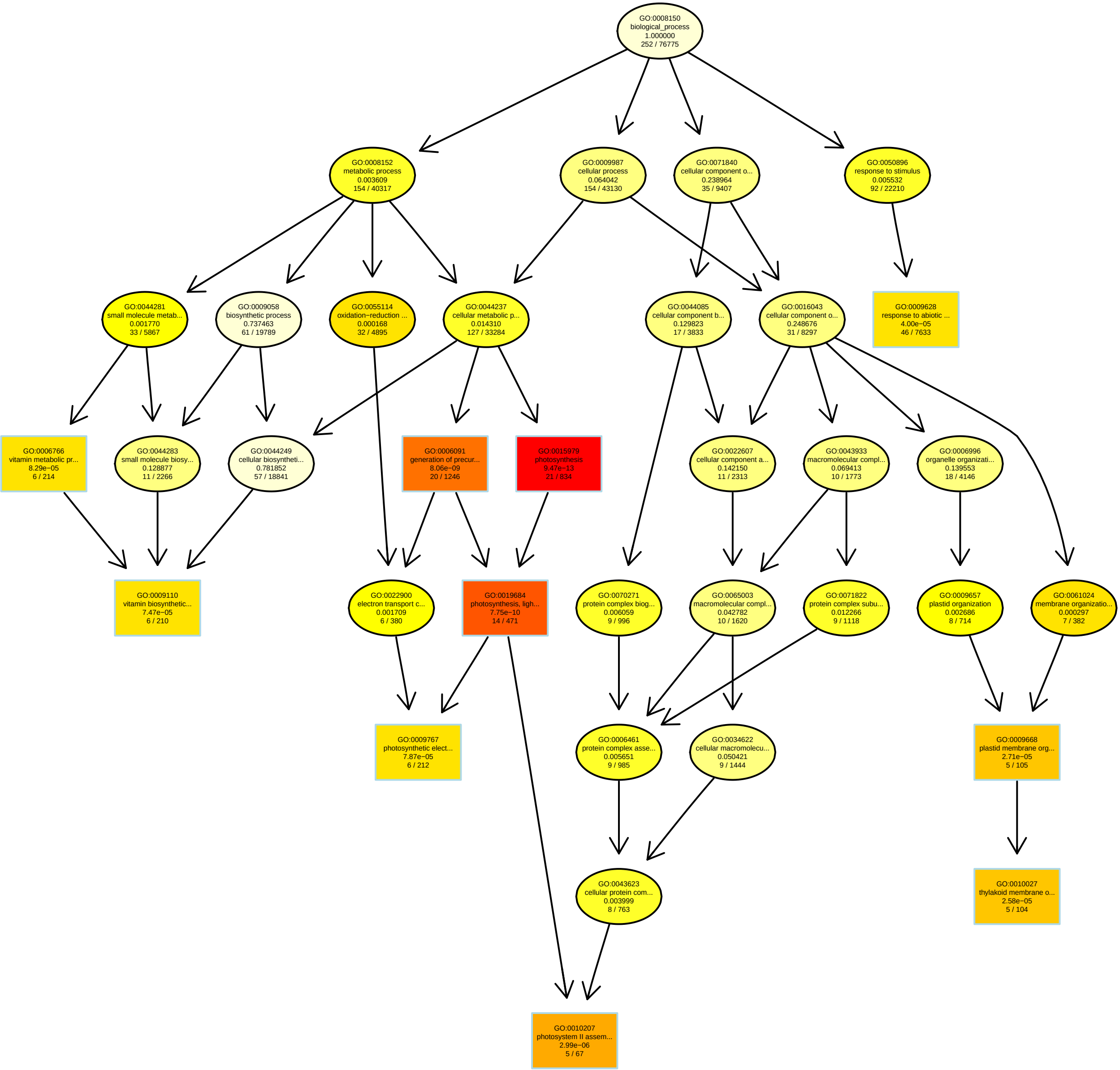

GO:0008150 biological_process 1.000000 252 / 76775
GO:0008152 metabolic process 0.003609 154 / 40317
GO:0009987 cellular process 0.064042 154 / 43130
GO:0071840 cellular component o... 0.238964 35 / 9407
GO:0050896 response to stimulus 0.005532 92 / 22210
GO:0044281 small molecule metab... 0.001770 33 / 5867
GO:0009058 biosynthetic process 0.737463 61 / 19789
GO:0055114 oxidation−reduction ... 0.000168 32 / 4895
GO:0044237 cellular metabolic p... 0.014310 127 / 33284
GO:0044085 cellular component b... 0.129823 17 / 3833
GO:0016043 cellular component o... 0.248676 31 / 8297
GO:0009628 response to abiotic ... 4.00e−05 46 / 7633
GO:0006766 vitamin metabolic pr... 8.29e−05 6 / 214
GO:0044283 small molecule biosy... 0.128877 11 / 2266
GO:0044249 cellular biosyntheti... 0.781852 57 / 18841
GO:0006091 generation of precur... 8.06e−09 20 / 1246
GO:0015979 photosynthesis 9.47e−13 21 / 834
GO:0022607 cellular component a... 0.142150 11 / 2313
GO:0043933 macromolecular compl... 0.069413 10 / 1773
GO:0006996 organelle organizati... 0.139553 18 / 4146
GO:0009110 vitamin biosynthetic... 7.47e−05 6 / 210
GO:0022900 electron transport c... 0.001709 6 / 380
GO:0019684 photosynthesis, ligh... 7.75e−10 14 / 471
GO:0070271 protein complex biog... 0.006059 9 / 996
GO:0065003 macromolecular compl... 0.042782 10 / 1620
GO:0071822 protein complex subu... 0.012266 9 / 1118
GO:0009657 plastid organization 0.002686 8 / 714
GO:0061024 membrane organizatio... 0.000297 7 / 382
GO:0009767 photosynthetic elect... 7.87e−05 6 / 212
GO:0006461 protein complex asse... 0.005651 9 / 985
GO:0034622 cellular macromolecu... 0.050421 9 / 1444
GO:0009668 plastid membrane org... 2.71e−05 5 / 105
GO:0043623 cellular protein com... 0.003999 8 / 763
GO:0010027 thylakoid membrane o... 2.58e−05 5 / 104
GO:0010207 photosystem II assem... 2.99e−06 5 / 67

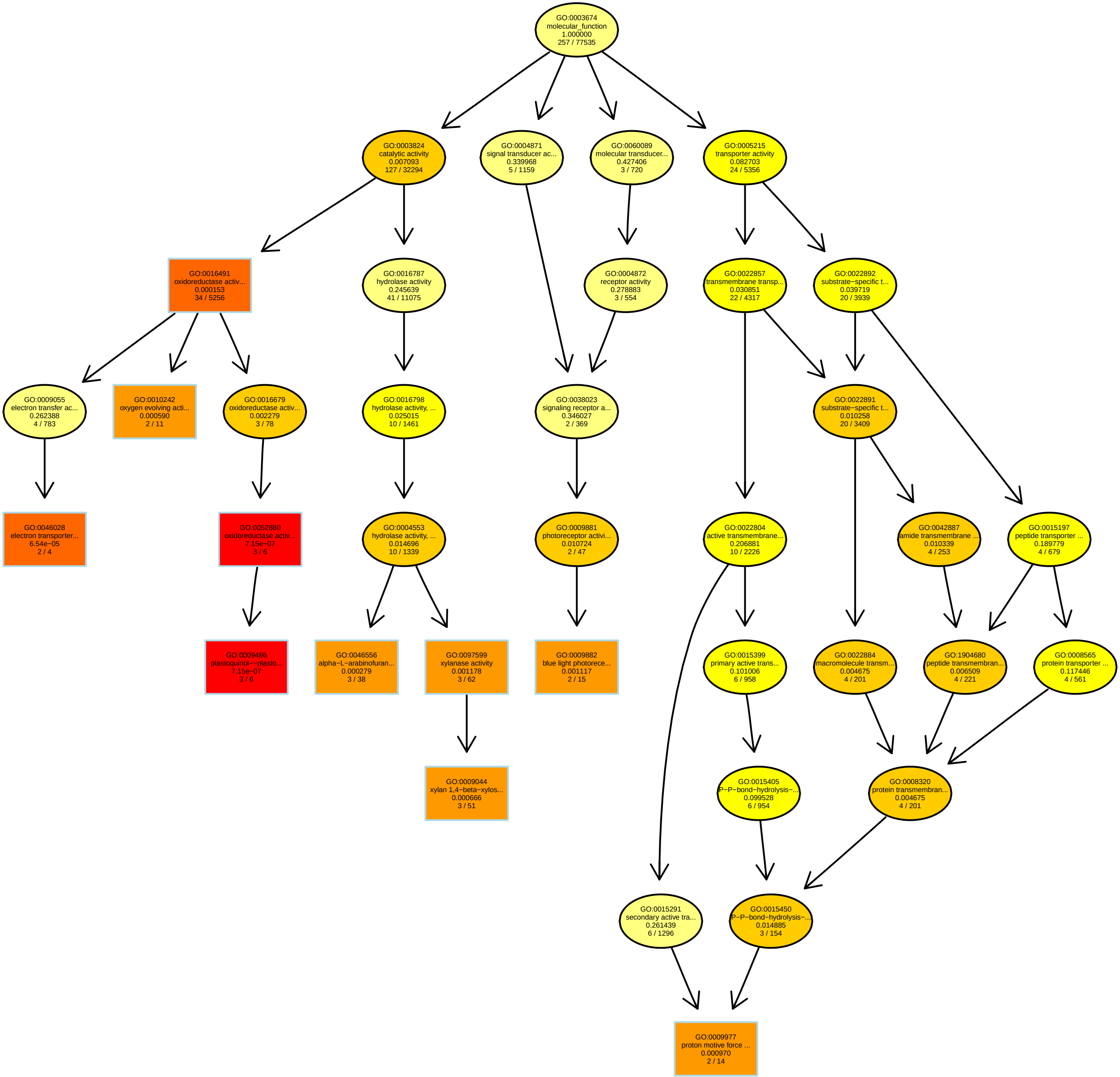
GO:0003674 molecular_function 1.000000 257 / 77535
GO:0003824 catalytic activity 0.007093 127 / 32294
GO:0004871 signal transducer ac... 0.339968 5 / 1159
GO:0060089 molecular transducer... 0.427406 3 / 720
GO:0005215 transporter activity 0.082703 24 / 5356
GO:0016491 oxidoreductase activ... 0.000153 34 / 5256
GO:0016787 hydrolase activity 0.245639 41 / 11075
GO:0004872 receptor activity 0.278883 3 / 554
GO:0022857 transmembrane transp... 0.030851 22 / 4317
GO:0022892 substrate−specific t... 0.039719 20 / 3939
GO:0009055 electron transfer ac... 0.262388 4 / 783
GO:0010242 oxygen evolving acti... 0.000590 2 / 11
GO:0016679 oxidoreductase activ... 0.002279 3 / 78
GO:0016798 hydrolase activity, ... 0.025015 10 / 1461
GO:0038023 signaling receptor a... 0.346027 2 / 369
GO:0022891 substrate−specific t... 0.010258 20 / 3409
GO:0046028 electron transporter... 6.54e−05 2 / 4
GO:0052880 oxidoreductase activ... 7.15e−07 3 / 6
GO:0004553 hydrolase activity, ... 0.014696 10 / 1339
GO:0009881 photoreceptor activi... 0.010724 2 / 47
GO:0022804 active transmembrane... 0.206881 10 / 2226
GO:0042887 amide transmembrane ... 0.010339 4 / 253
GO:0015197 peptide transporter ... 0.189779 4 / 679
GO:0009496 plastoquinol−−plasto... 7.15e−07 3 / 6
GO:0046556 alpha−L−arabinofuran... 0.000279 3 / 38
GO:0097599 xylanase activity 0.001178 3 / 62
GO:0009882 blue light photorece... 0.001117 2 / 15
GO:0015399 primary active trans... 0.101006 6 / 958
GO:0022884 macromolecule transm... 0.004675 4 / 201
GO:1904680 peptide transmembran... 0.006509 4 / 221
GO:0008565 protein transporter ... 0.117446 4 / 561
GO:0009044 xylan 1,4−beta−xylos... 0.000666 3 / 51
GO:0015405 P−P−bond−hydrolysis−... 0.099528 6 / 954
GO:0008320 protein transmembran... 0.004675 4 / 201
GO:0015291 secondary active tra... 0.261439 6 / 1296
GO:0015450 P−P−bond−hydrolysis−... 0.014885 3 / 154
GO:0009977 proton motive force ... 0.000970 2 / 14

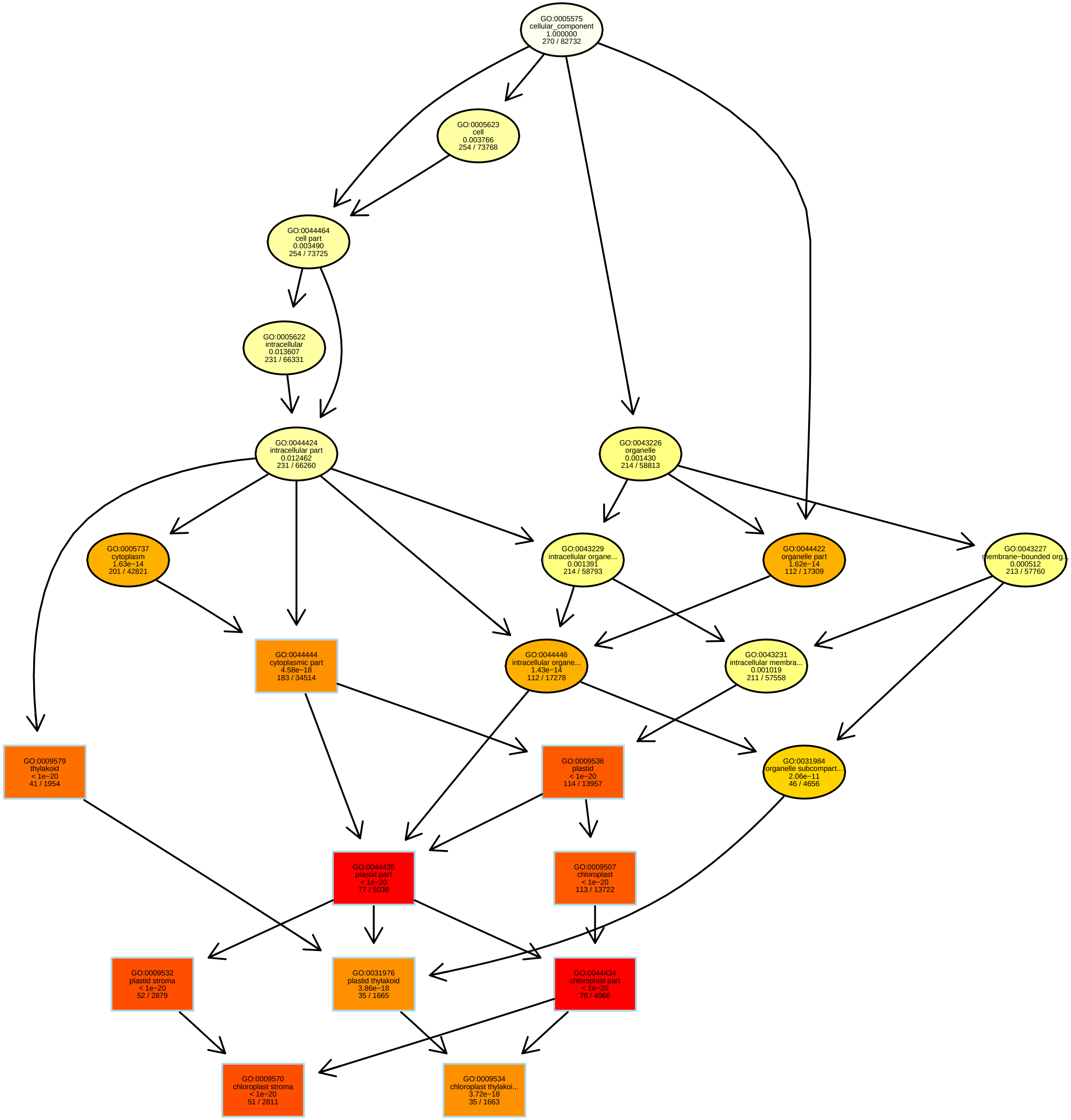

GO:0005575
cellular_component
1.000000
270 / 82732
GO:0005623
cell
0.003766
254 / 73768
GO:0044464
cell part
0.003490
254 / 73725
GO:0005622
intracellular
0.013607
231 / 66331
GO:0044424
intracellular part
0.012462
231 / 66260
GO:0043226
organelle
0.001430
214 / 58813
GO:0005737
cytoplasm
1.63e−14
201 / 42821
GO:0043229
intracellular organe...
0.001391
214 / 58793
GO:0044422
organelle part
1.62e−14
112 / 17309
GO:0043227
membrane−bounded org...
0.000512
213 / 57760
GO:0044444
cytoplasmic part
4.58e−18
183 / 34514
GO:0044446
intracellular organe...
1.43e−14
112 / 17278
GO:0043231
intracellular membra...
0.001019
211 / 57558
GO:0009579
thylakoid
< 1e−20
41 / 1954
GO:0009536
plastid
< 1e−20
114 / 13957
GO:0031984
organelle subcompart...
2.06e−11
46 / 4656
GO:0044435
plastid part
< 1e−20
77 / 5033
GO:0009507
chloroplast
< 1e−20
113 / 13722
GO:0009532
plastid stroma
< 1e−20
52 / 2879
GO:0031976
plastid thylakoid
3.86e−18
35 / 1665
GO:0044434
chloroplast part
< 1e−20
76 / 4966
GO:0009570
chloroplast stroma
< 1e−20
51 / 2811
GO:0009534
chloroplast thylakoi...
3.72e−18
35 / 1663
