# Supplementary material for: Differential Alternative Splicing Genes and Isoform Regulation Networks of Rapeseed (Brassica napus L.) Infected with Sclerotinia sclerotiorum
Source: Genes (Basel). 2020 Jul 13;11(7):784. doi: 10.3390/genes11070784 (PMC7397149; doi:10.3390/genes11070784)
Supplement: Supplementary file 1 [file genes-11-00784-s001.zip › Fig. S2 Enriched GO terms of genes in the 85 WGCNA modules.zip!/Fig. S2/CytoNodesalmon-go.pdf]

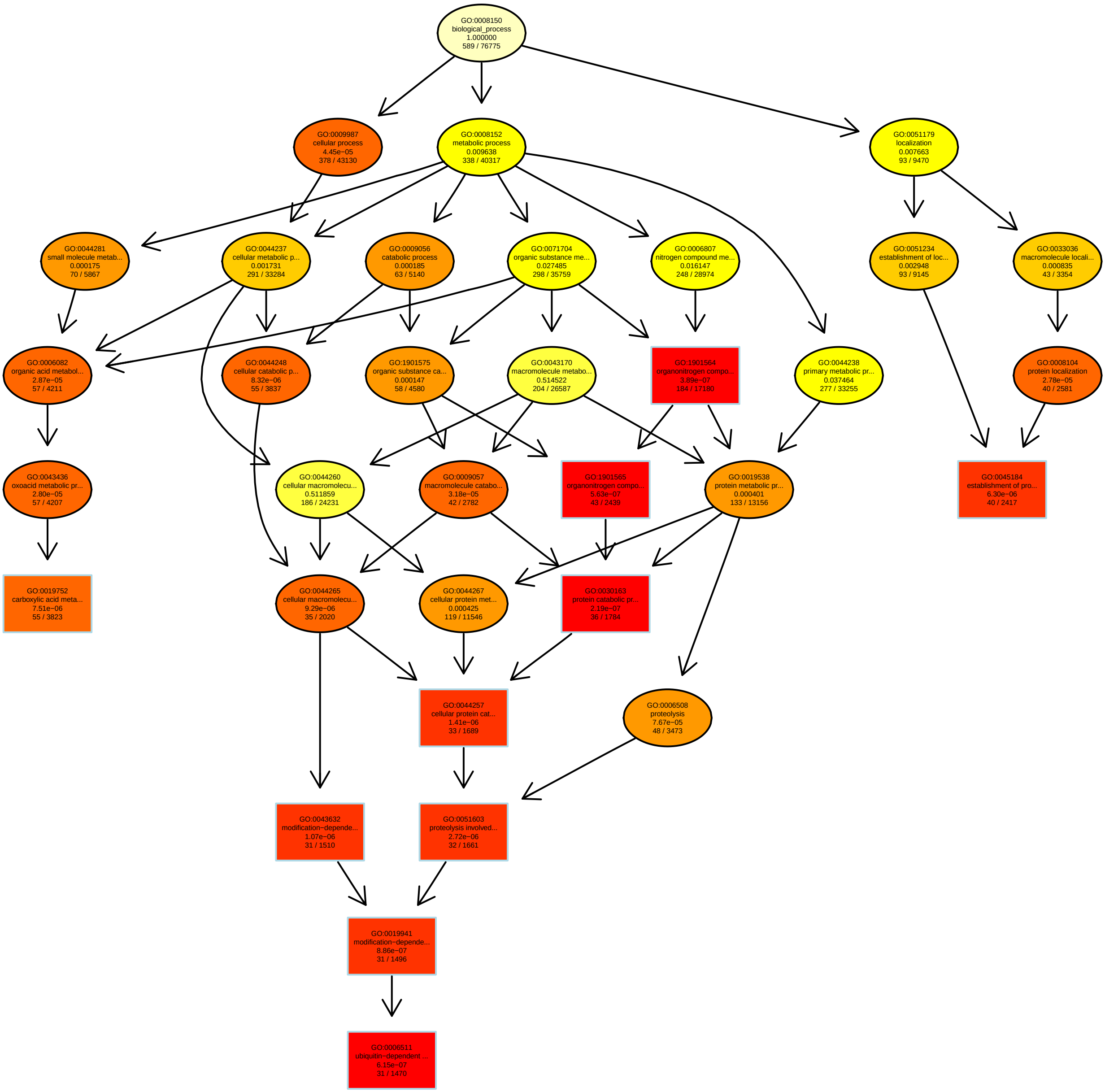

GO:0008150
biological_process
1.000000
589 / 76775
GO:0009987
cellular process
4.45e−05
378 / 43130
GO:0008152
metabolic process
0.009638
338 / 40317
GO:0051179
localization
0.007663
93 / 9470
GO:0044281
small molecule metab...
0.000175
70 / 5867
GO:0044237
cellular metabolic p...
0.001731
291 / 33284
GO:0009056
catabolic process
0.000185
63 / 5140
GO:0071704
organic substance me...
0.027485
298 / 35759
GO:0006807
nitrogen compound me...
0.016147
248 / 28974
GO:0051234
establishment of loc...
0.002948
93 / 9145
GO:0033036
macromolecule locali...
0.000835
43 / 3354
GO:0006082
organic acid metabol...
2.87e−05
57 / 4211
GO:0044248
cellular catabolic p...
8.32e−06
55 / 3837
GO:1901575
organic substance ca...
0.000147
58 / 4580
GO:0043170
macromolecule metabo...
0.514522
204 / 26587
GO:1901564
organonitrogen compo...
3.89e−07
184 / 17180
GO:0044238
primary metabolic pr...
0.037464
277 / 33255
GO:0008104
protein localization
2.78e−05
40 / 2581
GO:0043436
oxoacid metabolic pr...
2.80e−05
57 / 4207
GO:0044260
cellular macromolecu...
0.511859
186 / 24231
GO:0009057
macromolecule catabo...
3.18e−05
42 / 2782
GO:1901565
organonitrogen compo...
5.63e−07
43 / 2439
GO:0019538
protein metabolic pr...
0.000401
133 / 13156
GO:0045184
establishment of pro...
6.30e−06
40 / 2417
GO:0019752
carboxylic acid meta...
7.51e−06
55 / 3823
GO:0044265
cellular macromolecu...
9.29e−06
35 / 2020
GO:0044267
cellular protein met...
0.000425
119 / 11546
GO:0030163
protein catabolic pr...
2.19e−07
36 / 1784
GO:0044257
cellular protein cat...
1.41e−06
33 / 1689
GO:0006508
proteolysis
7.67e−05
48 / 3473
GO:0043632
modification−depende...
1.07e−06
31 / 1510
GO:0051603
proteolysis involved...
2.72e−06
32 / 1661
GO:0019941
modification−depende...
8.86e−07
31 / 1496
GO:0006511
ubiquitin−dependent ...
6.15e−07
31 / 1470

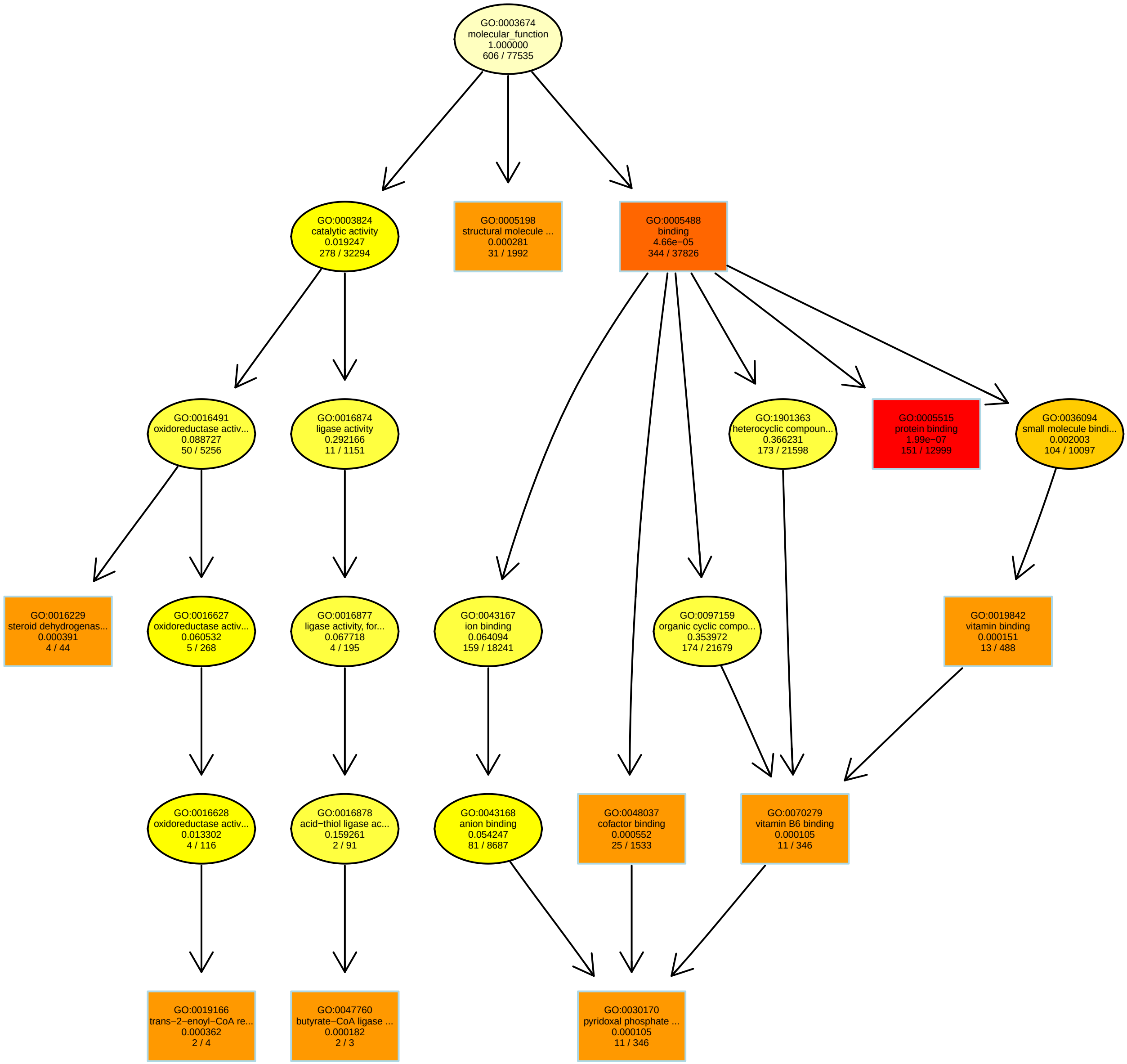

GO:0003674
molecular_function
1.000000
606 / 77535
GO:0003824
catalytic activity
0.019247
278 / 32294
GO:0005198
structural molecule ...
0.000281
31 / 1992
GO:0005488
binding
4.66e−05
344 / 37826
GO:0016491
oxidoreductase activ...
0.088727
50 / 5256
GO:0016874
ligase activity
0.292166
11 / 1151
GO:1901363
heterocyclic compoun...
0.366231
173 / 21598
GO:0005515
protein binding
1.99e−07
151 / 12999
GO:0036094
small molecule bindi...
0.002003
104 / 10097
GO:0016229
steroid dehydrogenas...
0.000391
4 / 44
GO:0016627
oxidoreductase activ...
0.060532
5 / 268
GO:0016877
ligase activity, for...
0.067718
4 / 195
GO:0043167
ion binding
0.064094
159 / 18241
GO:0097159
organic cyclic compo...
0.353972
174 / 21679
GO:0019842
vitamin binding
0.000151
13 / 488
GO:0016628
oxidoreductase activ...
0.013302
4 / 116
GO:0016878
acid−thiol ligase ac...
0.159261
2 / 91
GO:0043168
anion binding
0.054247
81 / 8687
GO:0048037
cofactor binding
0.000552
25 / 1533
GO:0070279
vitamin B6 binding
0.000105
11 / 346
GO:0019166
trans−2−enoyl−CoA re...
0.000362
2 / 4
GO:0047760
butyrate−CoA ligase ...
0.000182
2 / 3
GO:0030170
pyridoxal phosphate ...
0.000105
11 / 346

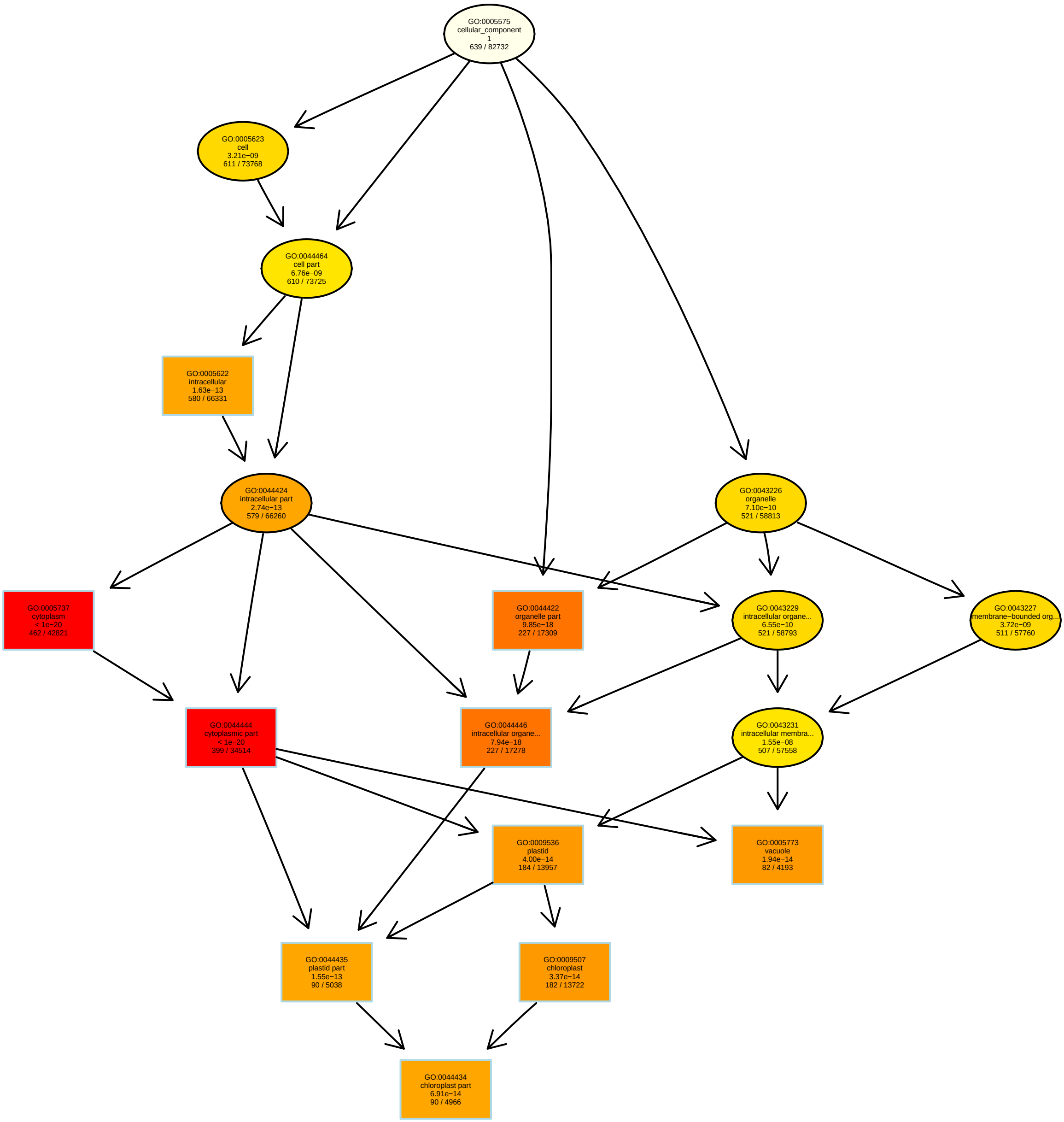
GO:0005575
cellular_component
1
639 / 82732
GO:0005623
cell
3.21e−09
611 / 73768
GO:0044464
cell part
6.76e−09
610 / 73725
GO:0005622
intracellular
1.63e−13
580 / 66331
GO:0044424
intracellular part
2.74e−13
579 / 66260
GO:0043226
organelle
7.10e−10
521 / 58813
GO:0005737
cytoplasm
< 1e−20
462 / 42821
GO:0044422
organelle part
9.85e−18
227 / 17309
GO:0043229
intracellular organe...
6.55e−10
521 / 58793
GO:0043227
membrane−bounded org...
3.72e−09
511 / 57760
GO:0044444
cytoplasmic part
< 1e−20
399 / 34514
GO:0044446
intracellular organe...
7.94e−18
227 / 17278
GO:0043231
intracellular membra...
1.55e−08
507 / 57558
GO:0009536
plastid
4.00e−14
184 / 13957
GO:0005773
vacuole
1.94e−14
82 / 4193
GO:0044435
plastid part
1.55e−13
90 / 5038
GO:0009507
chloroplast
3.37e−14
182 / 13722
GO:0044434
chloroplast part
6.91e−14
90 / 4966
