# Supplementary material for: Differential Alternative Splicing Genes and Isoform Regulation Networks of Rapeseed (Brassica napus L.) Infected with Sclerotinia sclerotiorum
Source: Genes (Basel). 2020 Jul 13;11(7):784. doi: 10.3390/genes11070784 (PMC7397149; doi:10.3390/genes11070784)
Supplement: Supplementary file 1 [file genes-11-00784-s001.zip › Fig. S2 Enriched GO terms of genes in the 85 WGCNA modules.zip!/Fig. S2/CytoNodesalmon2-go.pdf]

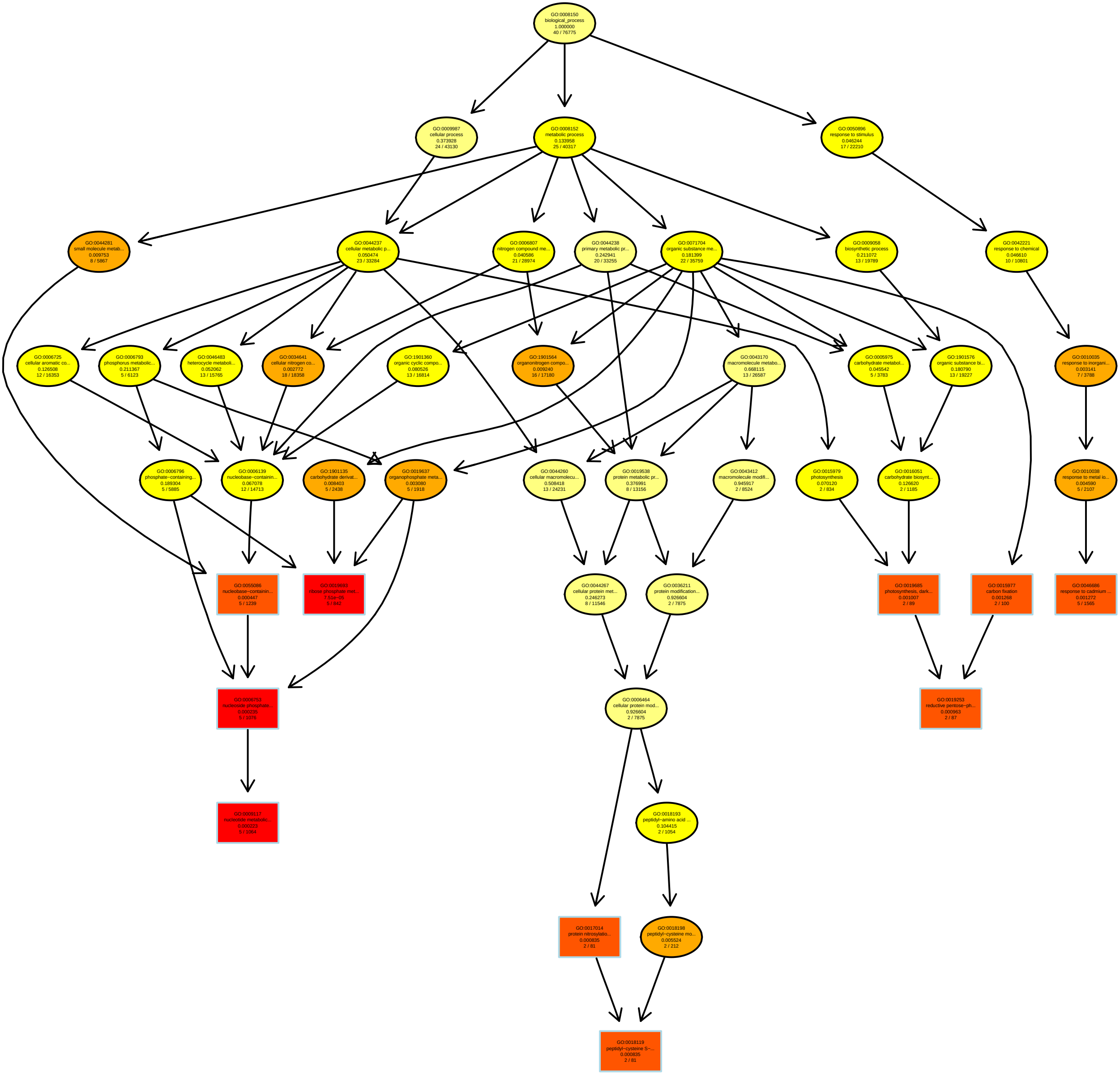
GO:0008150
biological_process
1.000000
40 / 76775
GO:0009987
cellular process
0.373928
24 / 43130
GO:0008152
metabolic process
0.133958
25 / 40317
GO:0050896
response to stimulus
0.046244
17 / 22210
GO:0044281
small molecule metab...
0.009753
8 / 5867
GO:0044237
cellular metabolic p...
0.050474
23 / 33284
GO:0006807
nitrogen compound me...
0.040586
21 / 28974
GO:0044238
primary metabolic pr...
0.242941
20 / 33255
GO:0071704
organic substance me...
0.181399
22 / 35759
GO:0009058
biosynthetic process
0.211072
13 / 19789
GO:0042221
response to chemical
0.046610
10 / 10801
GO:0006725
cellular aromatic co...
0.126508
12 / 16353
GO:0006793
phosphorus metabolic...
0.211367
5 / 6123
GO:0046483
heterocycle metaboli...
0.052062
13 / 15765
GO:0034641
cellular nitrogen co...
0.002772
18 / 18358
GO:1901360
organic cyclic compo...
0.080526
13 / 16814
GO:1901564
organonitrogen compo...
0.009240
16 / 17180
GO:0043170
macromolecule metabo...
0.668115
13 / 26587
GO:0005975
carbohydrate metabol...
0.045542
5 / 3783
GO:1901576
organic substance bi...
0.180790
13 / 19227
GO:0010035
response to inorgani...
0.003141
7 / 3788
GO:0006796
phosphate−containing...
0.189304
5 / 5885
GO:0006139
nucleobase−containin...
0.067078
12 / 14713
GO:1901135
carbohydrate derivat...
0.008403
5 / 2438
GO:0019637
organophosphate meta...
0.003080
5 / 1918
GO:0044260
cellular macromolecu...
0.508418
13 / 24231
GO:0019538
protein metabolic pr...
0.376991
8 / 13156
GO:0043412
macromolecule modifi...
0.945917
2 / 8524
GO:0015979
photosynthesis
0.070120
2 / 834
GO:0016051
carbohydrate biosynt...
0.126620
2 / 1185
GO:0010038
response to metal io...
0.004590
5 / 2107
GO:0055086
nucleobase−containin...
0.000447
5 / 1239
GO:0019693
ribose phosphate met...
7.51e−05
5 / 842
GO:0044267
cellular protein met...
0.246273
8 / 11546
GO:0036211
protein modification...
0.926604
2 / 7875
GO:0019685
photosynthesis, dark...
0.001007
2 / 89
GO:0015977
carbon fixation
0.001268
2 / 100
GO:0046686
response to cadmium ...
0.001272
5 / 1565
GO:0006753
nucleoside phosphate...
0.000235
5 / 1076
GO:0006464
cellular protein mod...
0.926604
2 / 7875
GO:0019253
reductive pentose−ph...
0.000963
2 / 87
GO:0009117
nucleotide metabolic...
0.000223
5 / 1064
GO:0018193
peptidyl−amino acid ...
0.104415
2 / 1054
GO:0017014
protein nitrosylatio...
0.000835
2 / 81
GO:0018198
peptidyl−cysteine mo...
0.005524
2 / 212
GO:0018119
peptidyl−cysteine S−...
0.000835
2 / 81

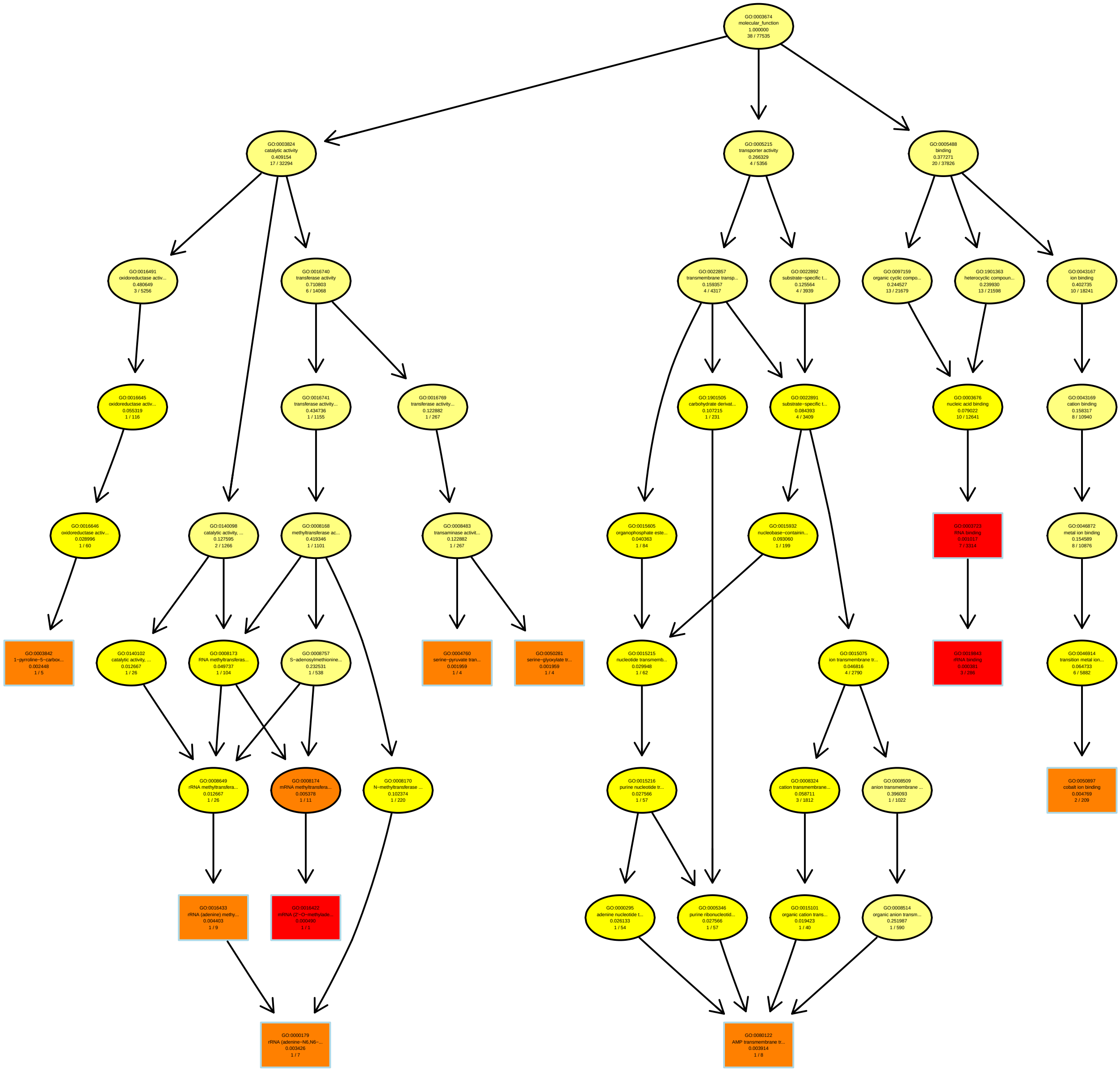
GO:0003674 molecular_function 1.000000 38 / 77535
GO:0003824 catalytic activity 0.409154 17 / 32294
GO:0005215 transporter activity 0.266329 4 / 5356
GO:0005488 binding 0.377271 20 / 37826
GO:0016491 oxidoreductase activ... 0.480649 3 / 5256
GO:0016740 transferase activity 0.710803 6 / 14068
GO:0022857 transmembrane transp... 0.159357 4 / 4317
GO:0022892 substrate−specific t... 0.125564 4 / 3939
GO:0097159 organic cyclic compo... 0.244527 13 / 21679
GO:1901363 heterocyclic compoun... 0.239930 13 / 21598
GO:0043167 ion binding 0.402735 10 / 18241
GO:0016645 oxidoreductase activ... 0.055319 1 / 116
GO:0016741 transferase activity... 0.434736 1 / 1155
GO:0016769 transferase activity... 0.122882 1 / 267
GO:1901505 carbohydrate derivat... 0.107215 1 / 231
GO:0022891 substrate−specific t... 0.084393 4 / 3409
GO:0003676 nucleic acid binding 0.079022 10 / 12641
GO:0043169 cation binding 0.158317 8 / 10940
GO:0016646 oxidoreductase activ... 0.028996 1 / 60
GO:0140098 catalytic activity, ... 0.127595 2 / 1266
GO:0008168 methyltransferase ac... 0.419346 1 / 1101
GO:0008483 transaminase activit... 0.122882 1 / 267
GO:0015605 organophosphate este... 0.040363 1 / 84
GO:0015932 nucleobase−containin... 0.093060 1 / 199
GO:0003723 RNA binding 0.001017 7 / 3314
GO:0046872 metal ion binding 0.154589 8 / 10876
GO:0003842 1−pyrroline−5−carbox... 0.002448 1 / 5
GO:0140102 catalytic activity, ... 0.012667 1 / 26
GO:0008173 RNA methyltransferas... 0.049737 1 / 104
GO:0008757 S−adenosylmethionine... 0.232531 1 / 538
GO:0004760 serine−pyruvate tran... 0.001959 1 / 4
GO:0050281 serine−glyoxylate tr... 0.001959 1 / 4
GO:0015215 nucleotide transmemb... 0.029948 1 / 62
GO:0015075 ion transmembrane tr... 0.046816 4 / 2790
GO:0019843 rRNA binding 0.000381 3 / 286
GO:0046914 transition metal ion... 0.064733 6 / 5882
GO:0008649 rRNA methyltransfera... 0.012667 1 / 26
GO:0008174 mRNA methyltransfera... 0.005378 1 / 11
GO:0008170 N−methyltransferase ... 0.102374 1 / 220
GO:0015216 purine nucleotide tr... 0.027566 1 / 57
GO:0008324 cation transmembrane... 0.058711 3 / 1812
GO:0008509 anion transmembrane ... 0.396093 1 / 1022
GO:0050897 cobalt ion binding 0.004769 2 / 209
GO:0016433 rRNA (adenine) methy... 0.004403 1 / 9
GO:0016422 mRNA (2'−O−methylade... 0.000490 1 / 1
GO:0000295 adenine nucleotide t... 0.026133 1 / 54
GO:0005346 purine ribonucleotid... 0.027566 1 / 57
GO:0015101 organic cation trans... 0.019423 1 / 40
GO:0008514 organic anion transm... 0.251987 1 / 590
GO:0000179 rRNA (adenine−N6,N6−... 0.003426 1 / 7
GO:0080122 AMP transmembrane tr... 0.003914 1 / 8

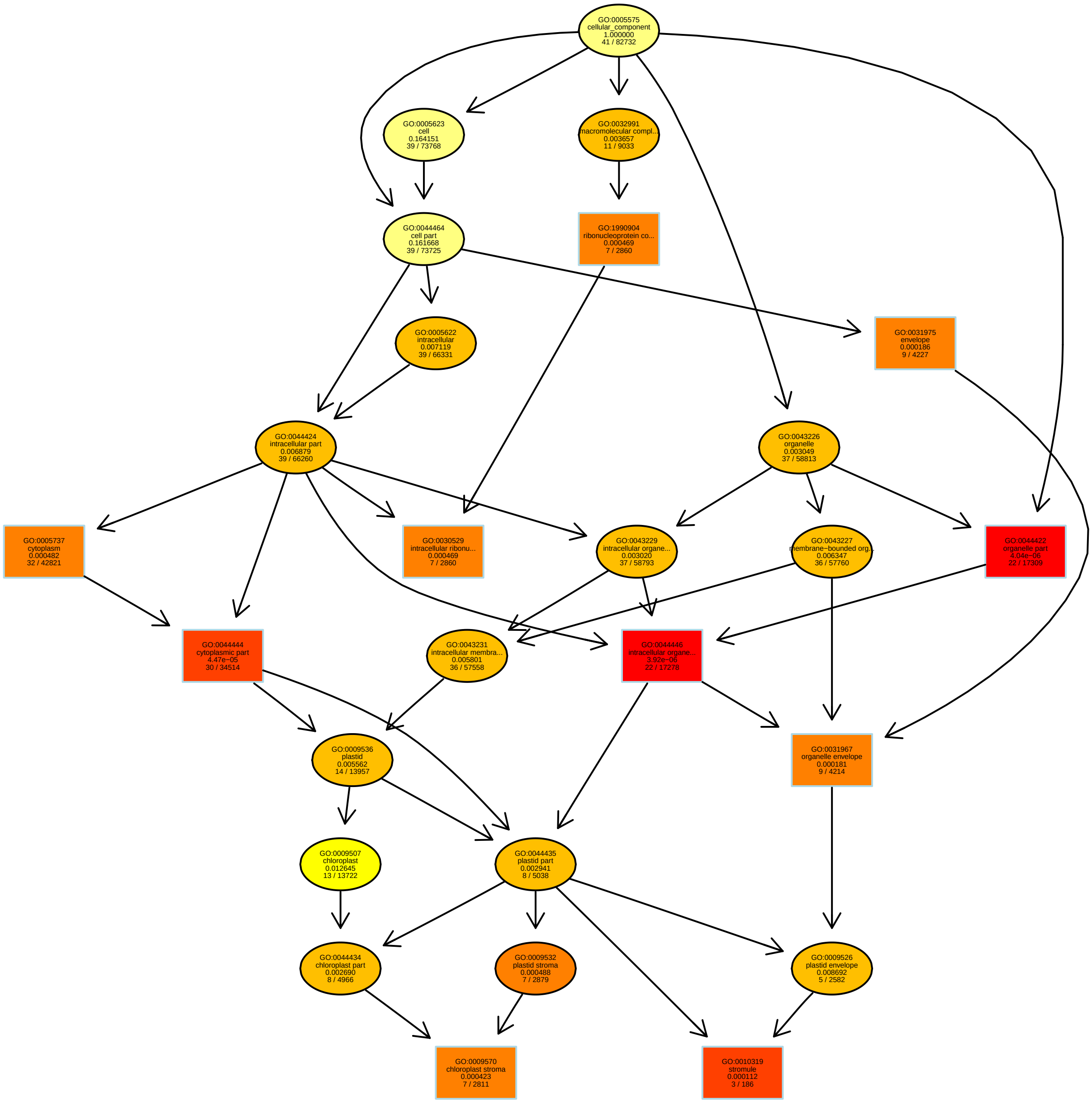
GO:0005575 cellular_component 1.000000 41 / 82732
GO:0005623 cell 0.164151 39 / 73768
GO:0032991 macromolecular compl... 0.003657 11 / 9033
GO:0044464 cell part 0.161668 39 / 73725
GO:1990904 ribonucleoprotein co... 0.000469 7 / 2860
GO:0005622 intracellular 0.007119 39 / 66331
GO:0031975 envelope 0.000186 9 / 4227
GO:0044424 intracellular part 0.006879 39 / 66260
GO:0043226 organelle 0.003049 37 / 58813
GO:0005737 cytoplasm 0.000482 32 / 42821
GO:0030529 intracellular ribonu... 0.000469 7 / 2860
GO:0043229 intracellular organe... 0.003020 37 / 58793
GO:0043227 membrane−bounded org... 0.006347 36 / 57760
GO:0044422 organelle part 4.04e−06 22 / 17309
GO:0044444 cytoplasmic part 4.47e−05 30 / 34514
GO:0043231 intracellular membra... 0.005801 36 / 57558
GO:0044446 intracellular organe... 3.92e−06 22 / 17278
GO:0009536 plastid 0.005562 14 / 13957
GO:0031967 organelle envelope 0.000181 9 / 4214
GO:0009507 chloroplast 0.012645 13 / 13722
GO:0044435 plastid part 0.002941 8 / 5038
GO:0044434 chloroplast part 0.002690 8 / 4966
GO:0009532 plastid stroma 0.000488 7 / 2879
GO:0009526 plastid envelope 0.008692 5 / 2582
GO:0009570 chloroplast stroma 0.000423 7 / 2811
GO:0010319 stromule 0.000112 3 / 186
