# Supplementary material for: Differential Alternative Splicing Genes and Isoform Regulation Networks of Rapeseed (Brassica napus L.) Infected with Sclerotinia sclerotiorum
Source: Genes (Basel). 2020 Jul 13;11(7):784. doi: 10.3390/genes11070784 (PMC7397149; doi:10.3390/genes11070784)
Supplement: Supplementary file 1 [file genes-11-00784-s001.zip › Fig. S2 Enriched GO terms of genes in the 85 WGCNA modules.zip!/Fig. S2/CytoNodesalmon4-go.pdf]

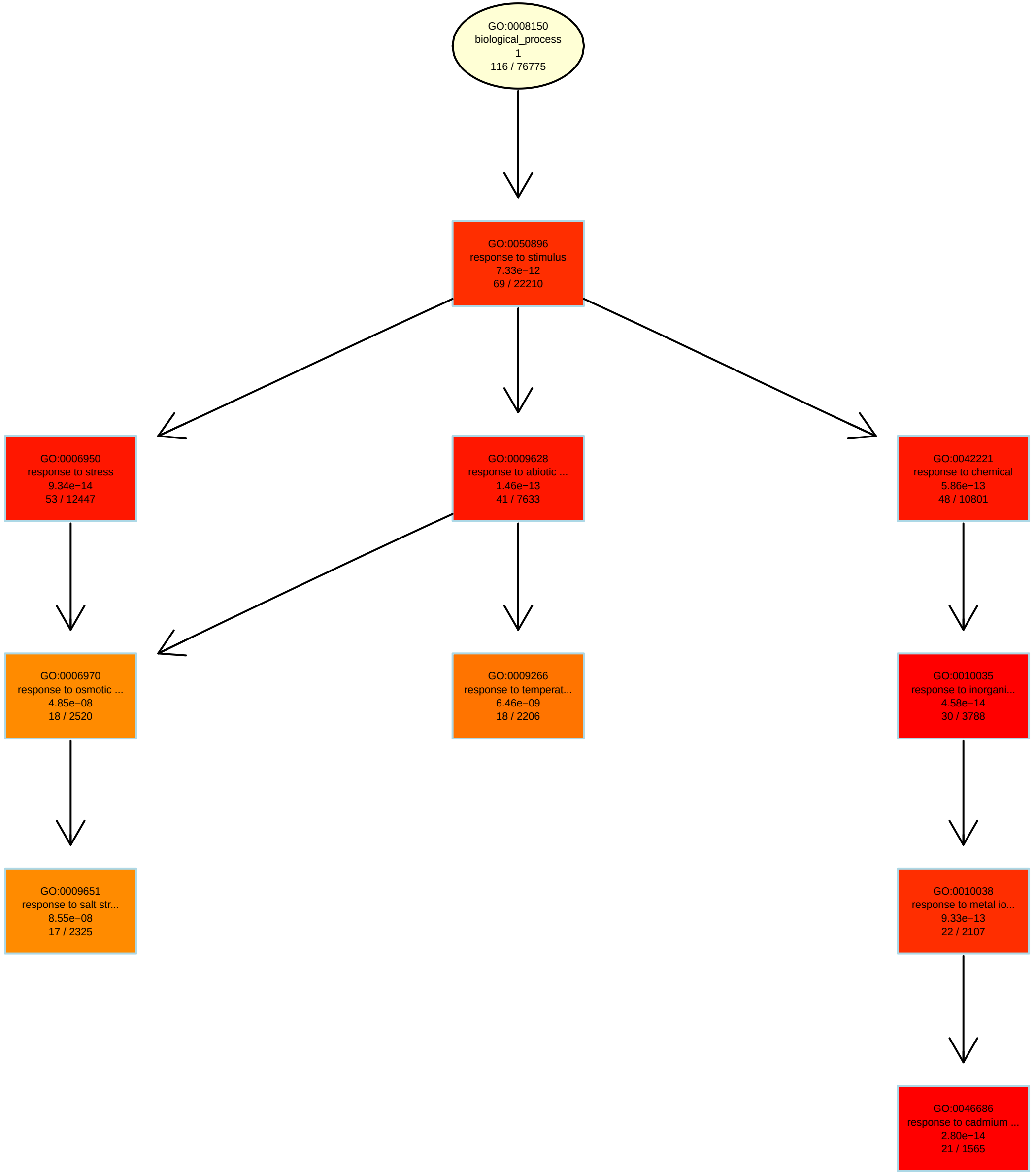
GO:0008150
biological_process
1
116 / 76775
GO:0050896
response to stimulus
7.33e−12
69 / 22210
GO:0006950
response to stress
9.34e−14
53 / 12447
GO:0009628
response to abiotic ...
1.46e−13
41 / 7633
GO:0042221
response to chemical
5.86e−13
48 / 10801
GO:0006970
response to osmotic ...
4.85e−08
18 / 2520
GO:0009266
response to temperat...
6.46e−09
18 / 2206
GO:0010035
response to inorgani...
4.58e−14
30 / 3788
GO:0009651
response to salt str...
8.55e−08
17 / 2325
GO:0010038
response to metal io...
9.33e−13
22 / 2107
GO:0046686
response to cadmium ...
2.80e−14
21 / 1565

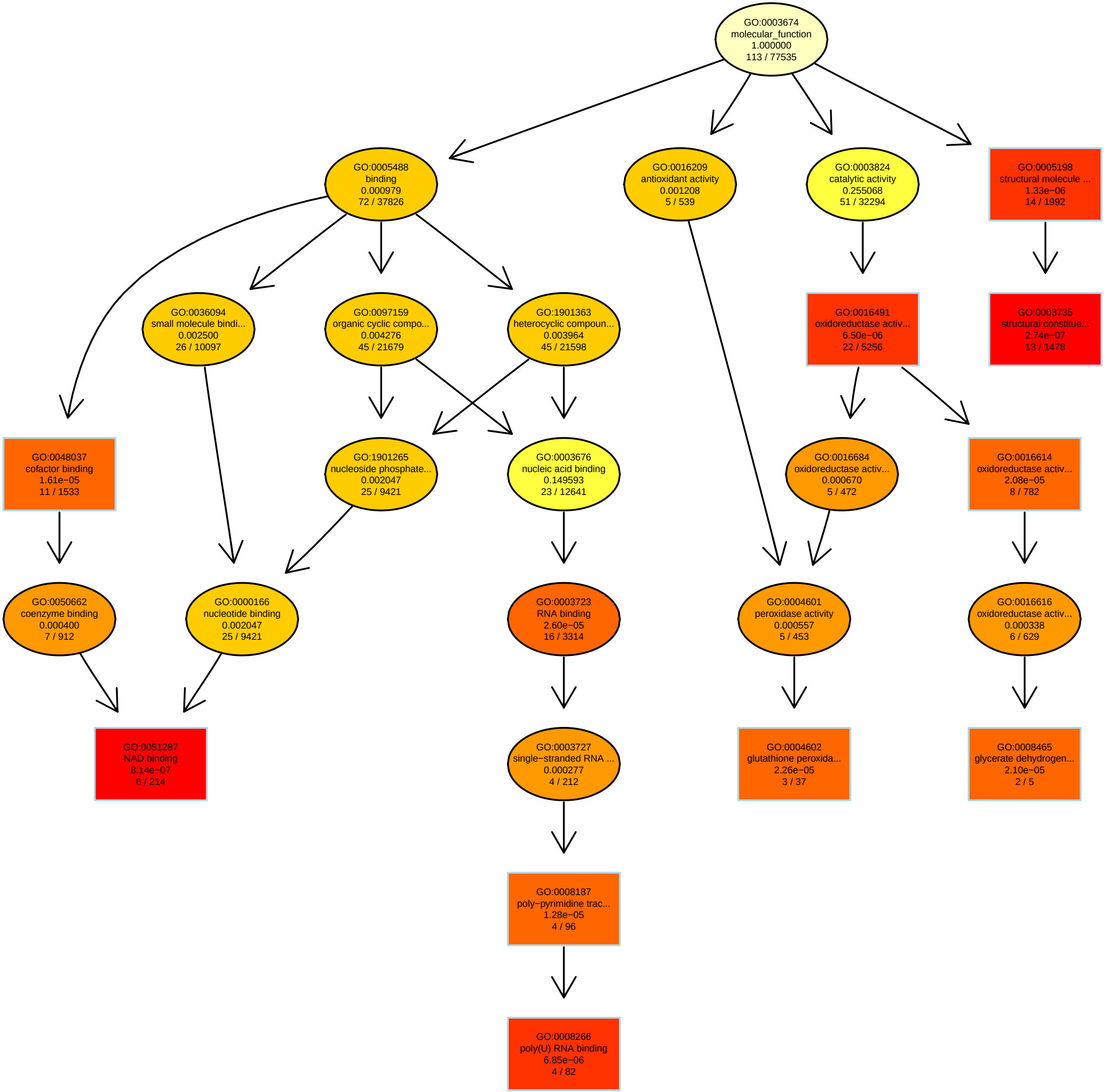

GO:0003674 molecular_function 1.000000 113 / 77535
GO:0005488 binding 0.000979 72 / 37826
GO:0016209 antioxidant activity 0.001208 5 / 539
GO:0003824 catalytic activity 0.255068 51 / 32294
GO:0005198 structural molecule ... 1.33e−06 14 / 1992
GO:0036094 small molecule bindi... 0.002500 26 / 10097
GO:0097159 organic cyclic compo... 0.004276 45 / 21679
GO:1901363 heterocyclic compoun... 0.003964 45 / 21598
GO:0016491 oxidoreductase activ... 6.50e−06 22 / 5256
GO:0003735 structural constitue... 2.74e−07 13 / 1478
GO:0048037 cofactor binding 1.61e−05 11 / 1533
GO:1901265 nucleoside phosphate... 0.002047 25 / 9421
GO:0003676 nucleic acid binding 0.149593 23 / 12641
GO:0016684 oxidoreductase activ... 0.000670 5 / 472
GO:0016614 oxidoreductase activ... 2.08e−05 8 / 782
GO:0050662 coenzyme binding 0.000400 7 / 912
GO:0000166 nucleotide binding 0.002047 25 / 9421
GO:0003723 RNA binding 2.60e−05 16 / 3314
GO:0004601 peroxidase activity 0.000557 5 / 453
GO:0016616 oxidoreductase activ... 0.000338 6 / 629
GO:0051287 NAD binding 8.14e−07 6 / 214
GO:0003727 single−stranded RNA ... 0.000277 4 / 212
GO:0004602 glutathione peroxida... 2.26e−05 3 / 37
GO:0008465 glycerate dehydrogen... 2.10e−05 2 / 5
GO:0008187 poly−pyrimidine trac... 1.28e−05 4 / 96
GO:0008266 poly(U) RNA binding 6.85e−06 4 / 82

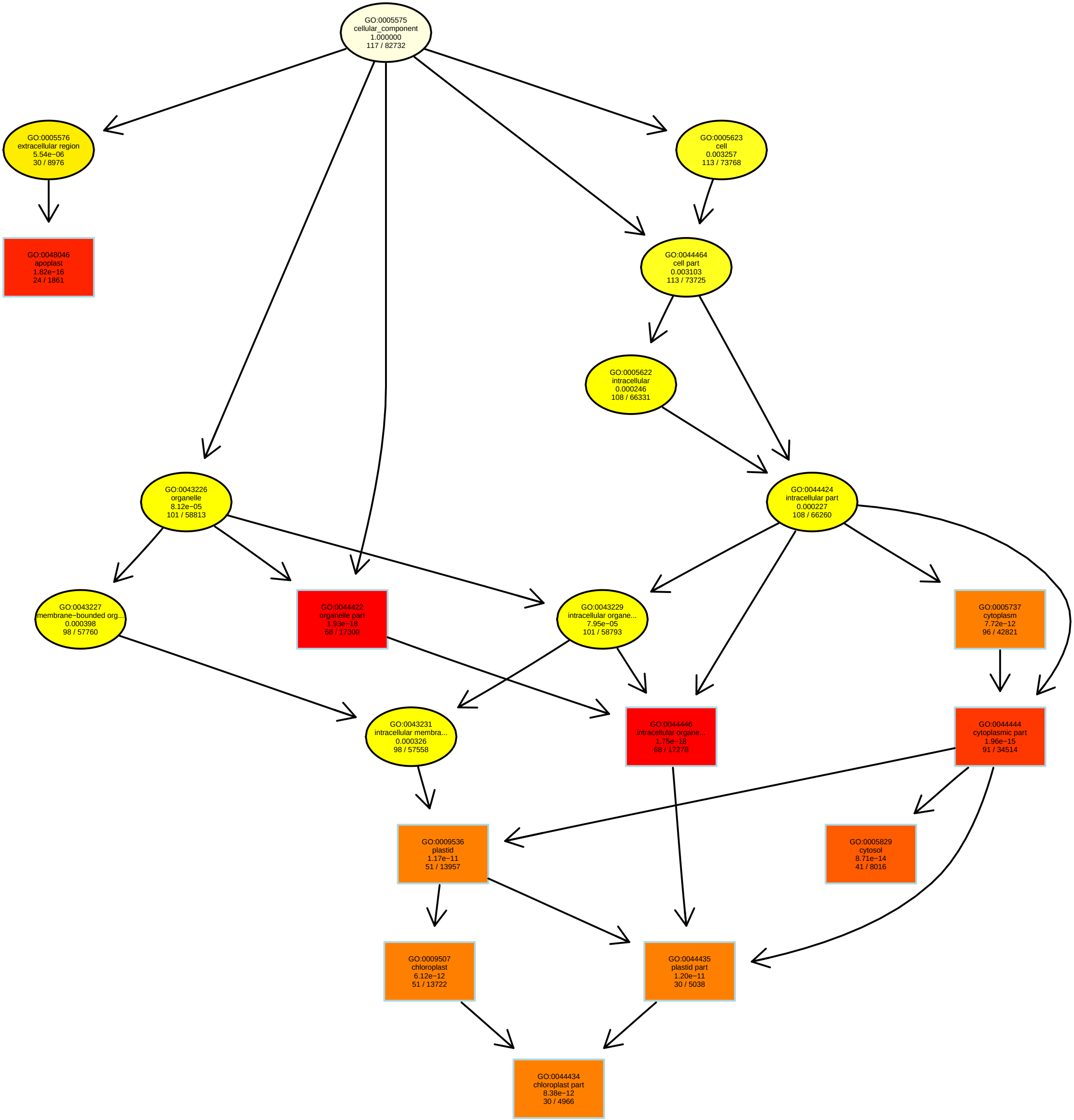
GO:0005575
cellular_component
1.000000
117 / 82732
GO:0005576
extracellular region
5.54e−06
30 / 8976
GO:0005623
cell
0.003257
113 / 73768
GO:0048046
apoplast
1.82e−16
24 / 1861
GO:0044464
cell part
0.003103
113 / 73725
GO:0005622
intracellular
0.000246
108 / 66331
GO:0043226
organelle
8.12e−05
101 / 58813
GO:0044424
intracellular part
0.000227
108 / 66260
GO:0043227
membrane−bounded org...
0.000398
98 / 57760
GO:0044422
organelle part
1.93e−18
68 / 17309
GO:0043229
intracellular organe...
7.95e−05
101 / 58793
GO:0005737
cytoplasm
7.72e−12
96 / 42821
GO:0043231
intracellular membra...
0.000326
98 / 57558
GO:0044446
intracellular organe...
1.75e−18
68 / 17278
GO:0044444
cytoplasmic part
1.96e−15
91 / 34514
GO:0009536
plastid
1.17e−11
51 / 13957
GO:0005829
cytosol
8.71e−14
41 / 8016
GO:0009507
chloroplast
6.12e−12
51 / 13722
GO:0044435
plastid part
1.20e−11
30 / 5038
GO:0044434
chloroplast part
8.38e−12
30 / 4966
