# Supplementary material for: Differential Alternative Splicing Genes and Isoform Regulation Networks of Rapeseed (Brassica napus L.) Infected with Sclerotinia sclerotiorum
Source: Genes (Basel). 2020 Jul 13;11(7):784. doi: 10.3390/genes11070784 (PMC7397149; doi:10.3390/genes11070784)
Supplement: Supplementary file 1 [file genes-11-00784-s001.zip › Fig. S2 Enriched GO terms of genes in the 85 WGCNA modules.zip!/Fig. S2/CytoNodesienna3-go.pdf]

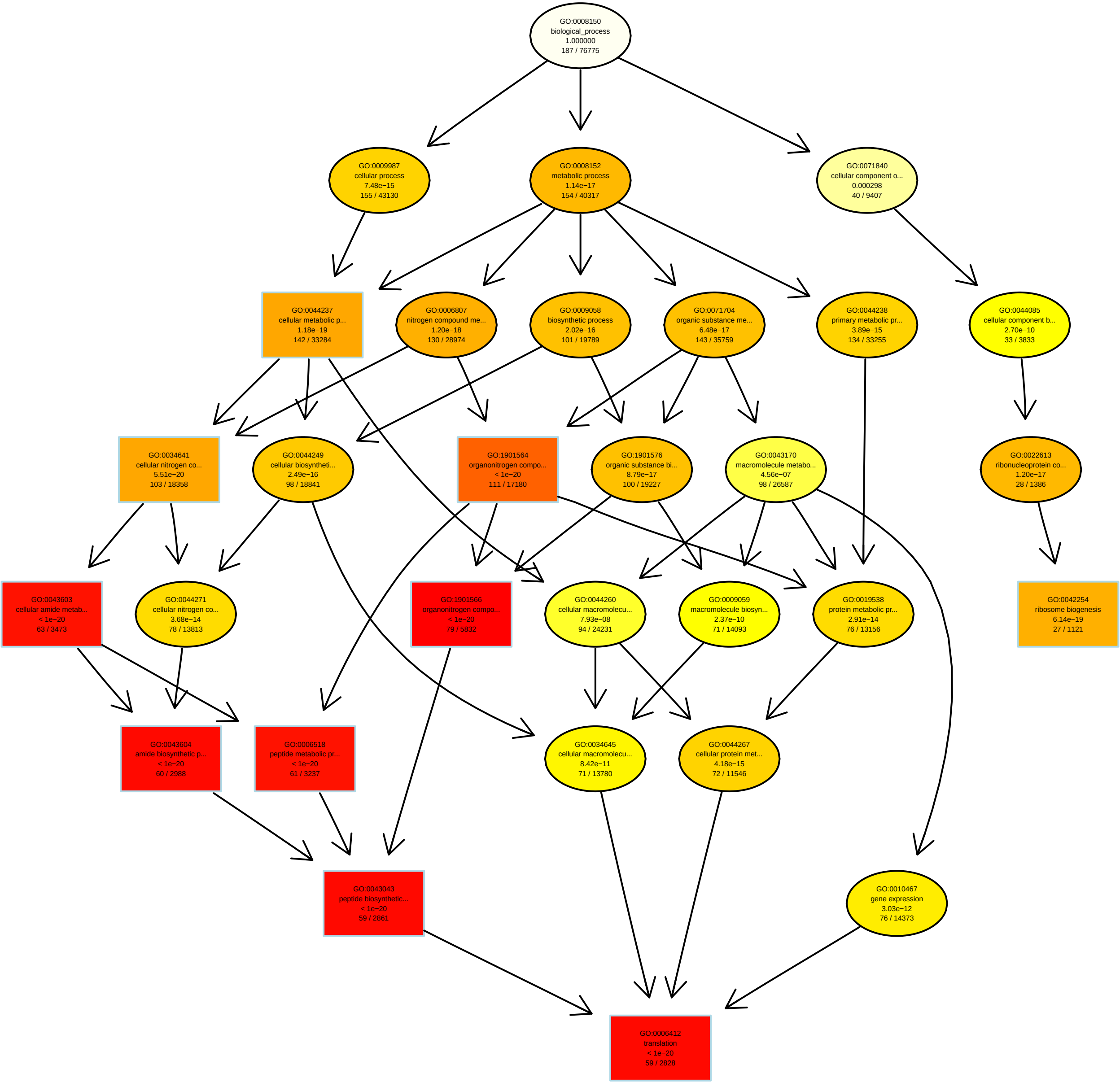
GO:0008150
biological_process
1.000000
187 / 76775
GO:0009987
cellular process
7.48e−15
155 / 43130
GO:0008152
metabolic process
1.14e−17
154 / 40317
GO:0071840
cellular component o...
0.000298
40 / 9407
GO:0044237
cellular metabolic p...
1.18e−19
142 / 33284
GO:0006807
nitrogen compound me...
1.20e−18
130 / 28974
GO:0009058
biosynthetic process
2.02e−16
101 / 19789
GO:0071704
organic substance me...
6.48e−17
143 / 35759
GO:0044238
primary metabolic pr...
3.89e−15
134 / 33255
GO:0044085
cellular component b...
2.70e−10
33 / 3833
GO:0034641
cellular nitrogen co...
5.51e−20
103 / 18358
GO:0044249
cellular biosyntheti...
2.49e−16
98 / 18841
GO:1901564
organonitrogen compo...
< 1e−20
111 / 17180
GO:1901576
organic substance bi...
8.79e−17
100 / 19227
GO:0043170
macromolecule metabo...
4.56e−07
98 / 26587
GO:0022613
ribonucleoprotein co...
1.20e−17
28 / 1386
GO:0043603
cellular amide metab...
< 1e−20
63 / 3473
GO:0044271
cellular nitrogen co...
3.68e−14
78 / 13813
GO:1901566
organonitrogen compo...
< 1e−20
79 / 5832
GO:0044260
cellular macromolecu...
7.93e−08
94 / 24231
GO:0009059
macromolecule biosyn...
2.37e−10
71 / 14093
GO:0019538
protein metabolic pr...
2.91e−14
76 / 13156
GO:0042254
ribosome biogenesis
6.14e−19
27 / 1121
GO:0043604
amide biosynthetic p...
< 1e−20
60 / 2988
GO:0006518
peptide metabolic pr...
< 1e−20
61 / 3237
GO:0034645
cellular macromolecu...
8.42e−11
71 / 13780
GO:0044267
cellular protein met...
4.18e−15
72 / 11546
GO:0043043
peptide biosynthetic...
< 1e−20
59 / 2861
GO:0010467
gene expression
3.03e−12
76 / 14373
GO:0006412
translation
< 1e−20
59 / 2828

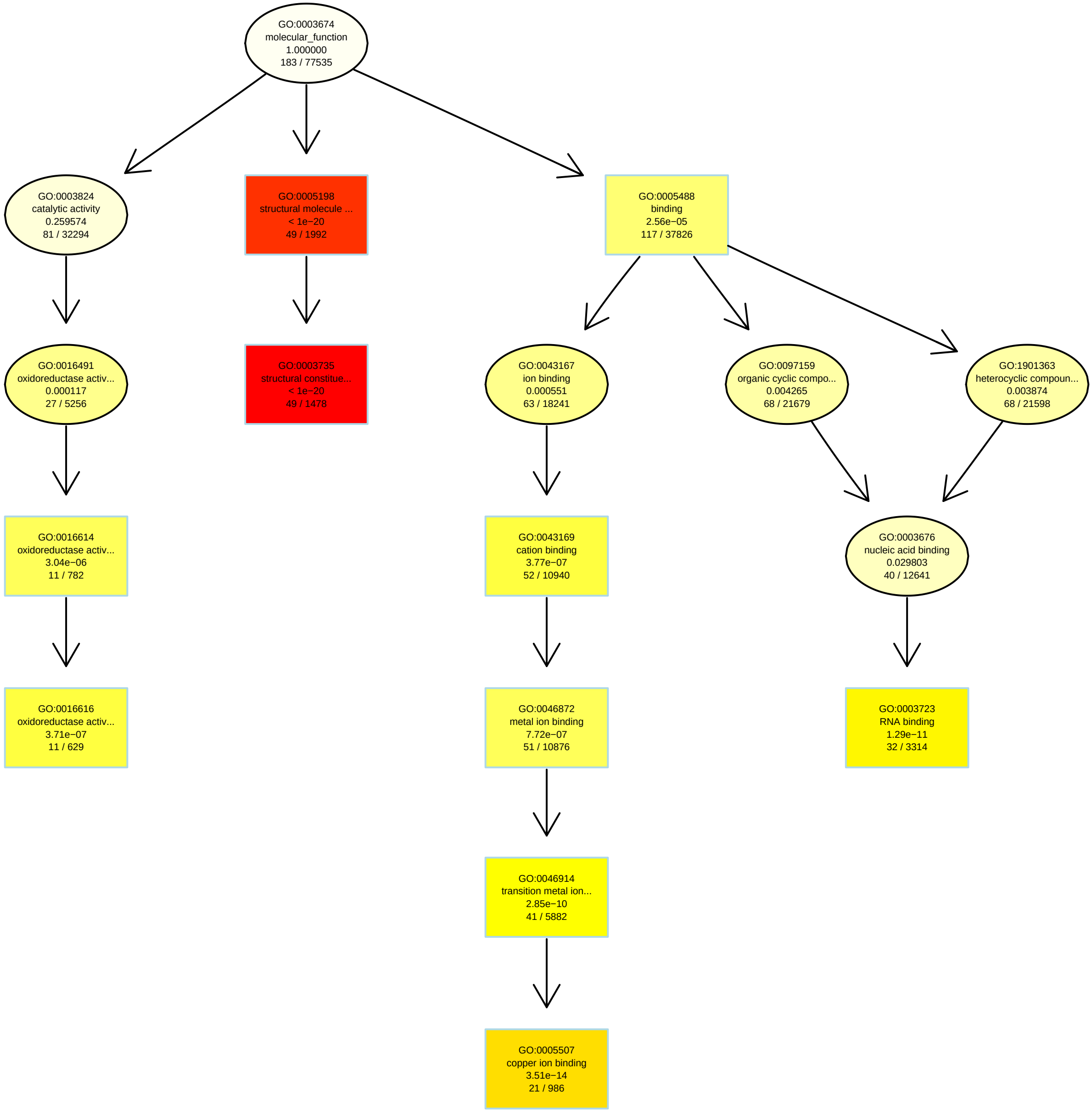
GO:0003674
molecular_function
1.000000
183 / 77535
GO:0003824
catalytic activity
0.259574
81 / 32294
GO:0005198
structural molecule ...
< 1e−20
49 / 1992
GO:0005488
binding
2.56e−05
117 / 37826
GO:0016491
oxidoreductase activ...
0.000117
27 / 5256
GO:0003735
structural constitue...
< 1e−20
49 / 1478
GO:0043167
ion binding
0.000551
63 / 18241
GO:0097159
organic cyclic compo...
0.004265
68 / 21679
GO:1901363
heterocyclic compoun...
0.003874
68 / 21598
GO:0016614
oxidoreductase activ...
3.04e−06
11 / 782
GO:0043169
cation binding
3.77e−07
52 / 10940
GO:0003676
nucleic acid binding
0.029803
40 / 12641
GO:0016616
oxidoreductase activ...
3.71e−07
11 / 629
GO:0046872
metal ion binding
7.72e−07
51 / 10876
GO:0003723
RNA binding
1.29e−11
32 / 3314
GO:0046914
transition metal ion...
2.85e−10
41 / 5882
GO:0005507
copper ion binding
3.51e−14
21 / 986

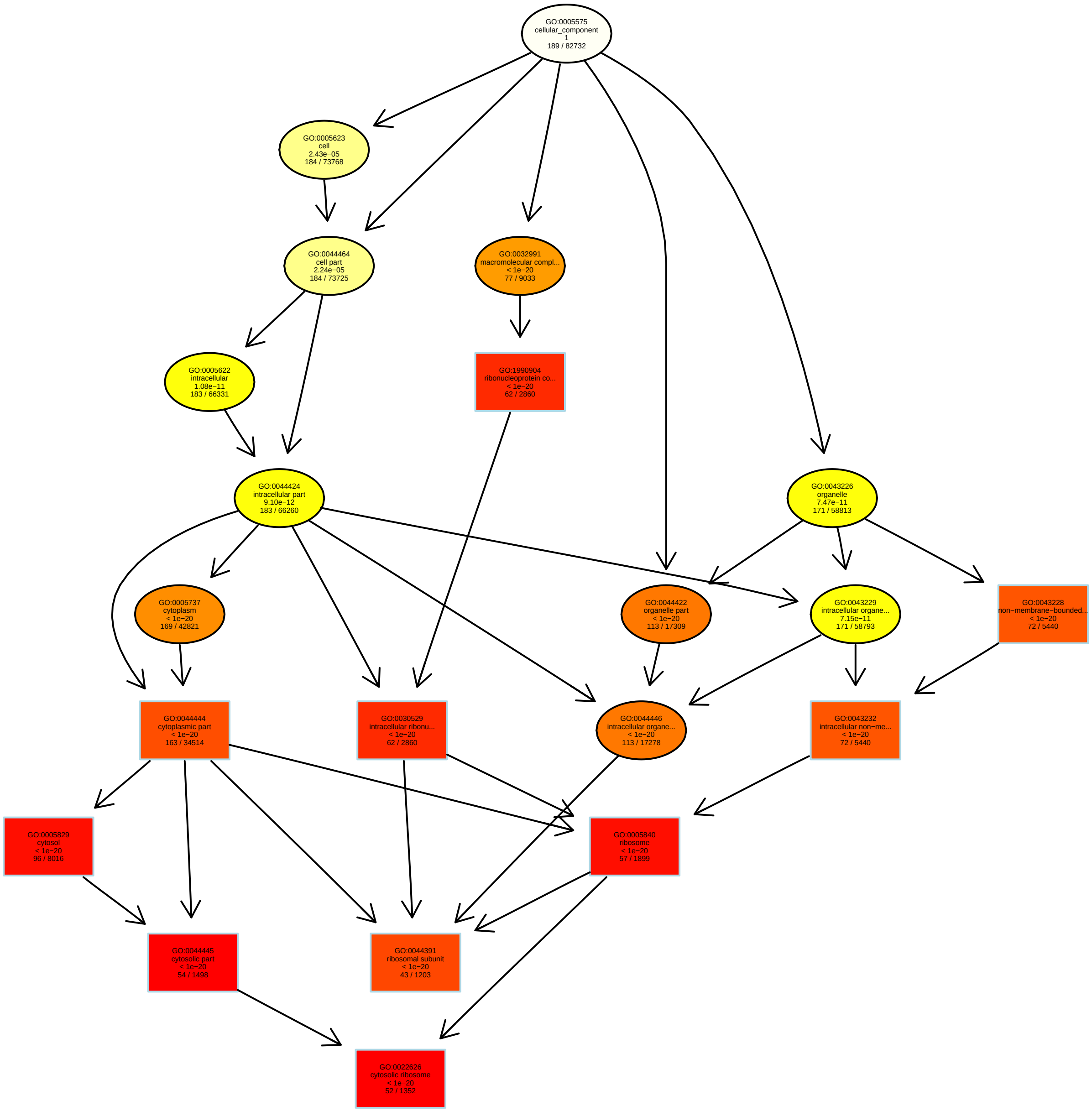
GO:0005575
cellular_component
1
189 / 82732
GO:0005623
cell
2.43e−05
184 / 73768
GO:0044464
cell part
2.24e−05
184 / 73725
GO:0032991
macromolecular compl...
< 1e−20
77 / 9033
GO:0005622
intracellular
1.08e−11
183 / 66331
GO:1990904
ribonucleoprotein co...
< 1e−20
62 / 2860
GO:0044424
intracellular part
9.10e−12
183 / 66260
GO:0043226
organelle
7.47e−11
171 / 58813
GO:0005737
cytoplasm
< 1e−20
169 / 42821
GO:0044422
organelle part
< 1e−20
113 / 17309
GO:0043229
intracellular organe...
7.15e−11
171 / 58793
GO:0043228
non−membrane−bounded...
< 1e−20
72 / 5440
GO:0044444
cytoplasmic part
< 1e−20
163 / 34514
GO:0030529
intracellular ribonu...
< 1e−20
62 / 2860
GO:0044446
intracellular organe...
< 1e−20
113 / 17278
GO:0043232
intracellular non−me...
< 1e−20
72 / 5440
GO:0005829
cytosol
< 1e−20
96 / 8016
GO:0005840
ribosome
< 1e−20
57 / 1899
GO:0044445
cytosolic part
< 1e−20
54 / 1498
GO:0044391
ribosomal subunit
< 1e−20
43 / 1203
GO:0022626
cytosolic ribosome
< 1e−20
52 / 1352
