# Supplementary material for: Differential Alternative Splicing Genes and Isoform Regulation Networks of Rapeseed (Brassica napus L.) Infected with Sclerotinia sclerotiorum
Source: Genes (Basel). 2020 Jul 13;11(7):784. doi: 10.3390/genes11070784 (PMC7397149; doi:10.3390/genes11070784)
Supplement: Supplementary file 1 [file genes-11-00784-s001.zip › Fig. S2 Enriched GO terms of genes in the 85 WGCNA modules.zip!/Fig. S2/CytoNodeskyblue-go.pdf]

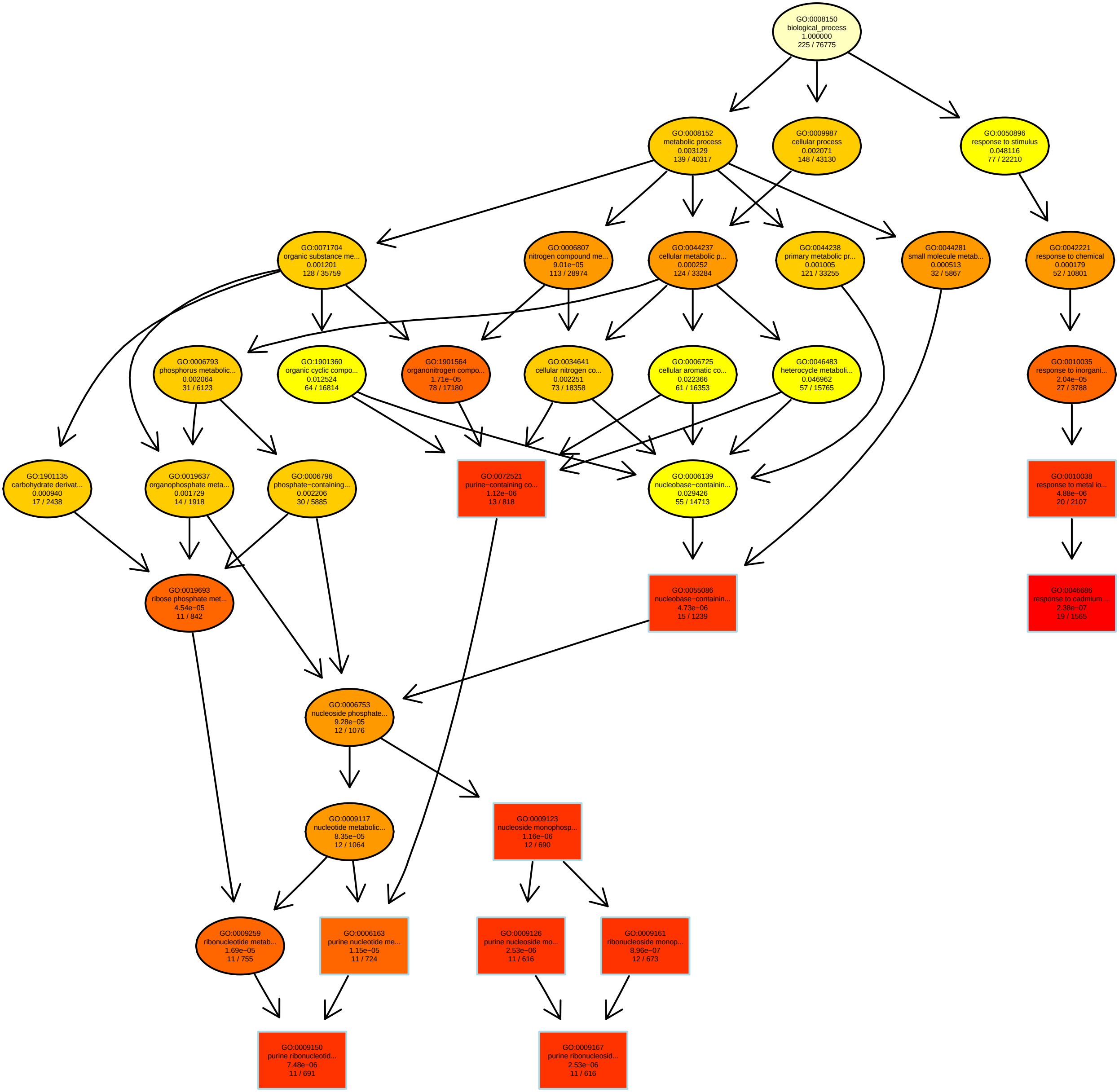
GO:0008150 biological_process 1.000000 225 / 76775
GO:0008152 metabolic process 0.003129 139 / 40317
GO:0009987 cellular process 0.002071 148 / 43130
GO:0050896 response to stimulus 0.048116 77 / 22210
GO:0071704 organic substance me... 0.001201 128 / 35759
GO:0006807 nitrogen compound me... 9.01e−05 113 / 28974
GO:0044237 cellular metabolic p... 0.000252 124 / 33284
GO:0044238 primary metabolic pr... 0.001005 121 / 33255
GO:0044281 small molecule metab... 0.000513 32 / 5867
GO:0042221 response to chemical 0.000179 52 / 10801
GO:0006793 phosphorus metabolic... 0.002064 31 / 6123
GO:1901360 organic cyclic compo... 0.012524 64 / 16814
GO:1901564 organonitrogen compo... 1.71e−05 78 / 17180
GO:0034641 cellular nitrogen co... 0.002251 73 / 18358
GO:0006725 cellular aromatic co... 0.022366 61 / 16353
GO:0046483 heterocycle metaboli... 0.046962 57 / 15765
GO:0010035 response to inorgani... 2.04e−05 27 / 3788
GO:1901135 carbohydrate derivat... 0.000940 17 / 2438
GO:0019637 organophosphate meta... 0.001729 14 / 1918
GO:0006796 phosphate−containing... 0.002206 30 / 5885
GO:0072521 purine−containing co... 1.12e−06 13 / 818
GO:0006139 nucleobase−containin... 0.029426 55 / 14713
GO:0010038 response to metal io... 4.88e−06 20 / 2107
GO:0019693 ribose phosphate met... 4.54e−05 11 / 842
GO:0055086 nucleobase−containin... 4.73e−06 15 / 1239
GO:0046686 response to cadmium ... 2.38e−07 19 / 1565
GO:0006753 nucleoside phosphate... 9.28e−05 12 / 1076
GO:0009117 nucleotide metabolic... 8.35e−05 12 / 1064
GO:0009123 nucleoside monophosp... 1.16e−06 12 / 690
GO:0009259 ribonucleotide metab... 1.69e−05 11 / 755
GO:0006163 purine nucleotide me... 1.15e−05 11 / 724
GO:0009126 purine nucleoside mo... 2.53e−06 11 / 616
GO:0009161 ribonucleoside monop... 8.96e−07 12 / 673
GO:0009150 purine ribonucleotid... 7.48e−06 11 / 691
GO:0009167 purine ribonucleosid... 2.53e−06 11 / 616

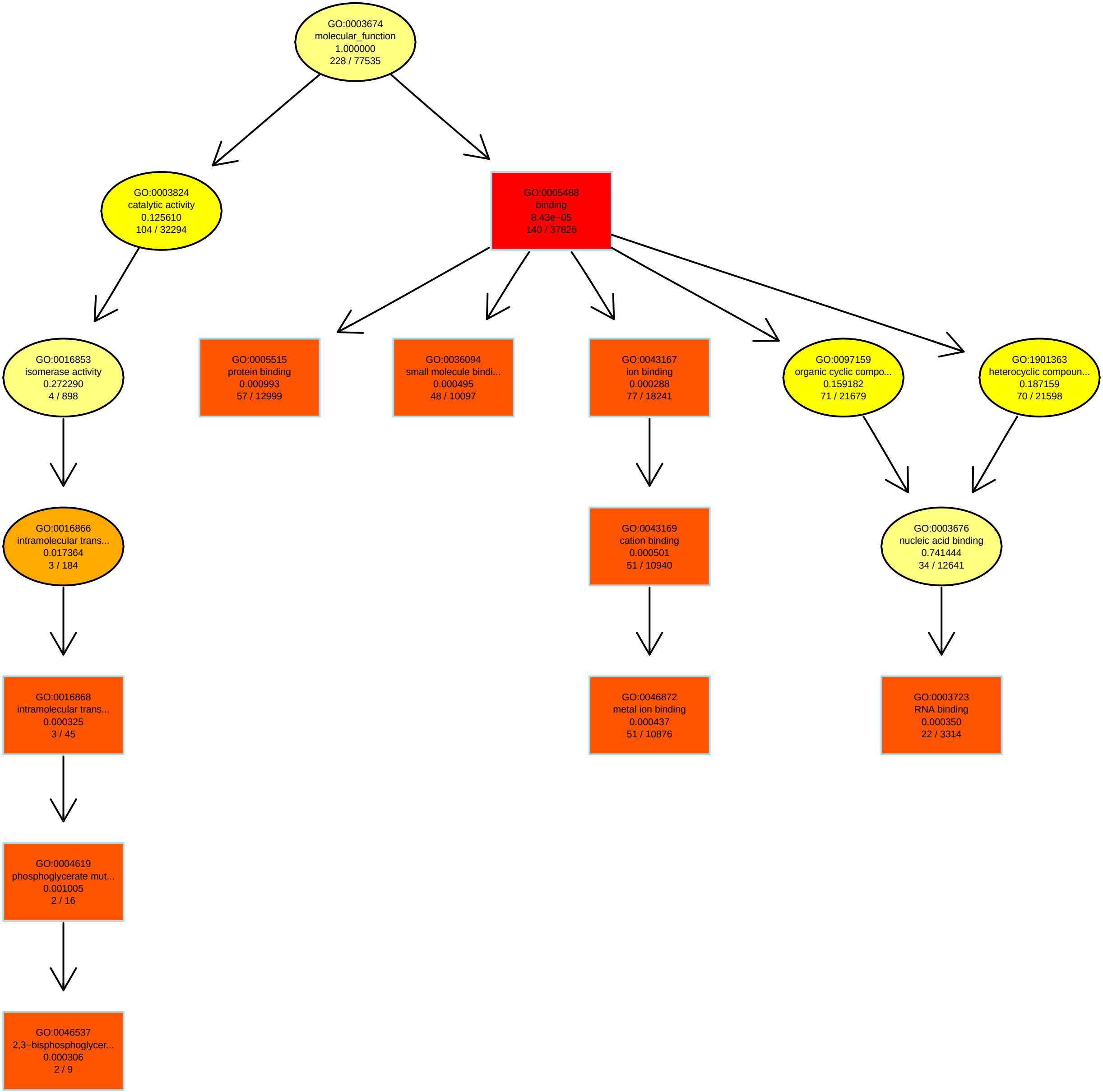
GO:0003674
molecular_function
1.000000
228 / 77535
GO:0003824
catalytic activity
0.125610
104 / 32294
GO:0005488
binding
8.43e−05
140 / 37826
GO:0016853
isomerase activity
0.272290
4 / 898
GO:0005515
protein binding
0.000993
57 / 12999
GO:0036094
small molecule bindi...
0.000495
48 / 10097
GO:0043167
ion binding
0.000288
77 / 18241
GO:0097159
organic cyclic compo...
0.159182
71 / 21679
GO:1901363
heterocyclic compoun...
0.187159
70 / 21598
GO:0016866
intramolecular trans...
0.017364
3 / 184
GO:0043169
cation binding
0.000501
51 / 10940
GO:0003676
nucleic acid binding
0.741444
34 / 12641
GO:0016868
intramolecular trans...
0.000325
3 / 45
GO:0046872
metal ion binding
0.000437
51 / 10876
GO:0003723
RNA binding
0.000350
22 / 3314
GO:0004619
phosphoglycerate mut...
0.001005
2 / 16
GO:0046537
2,3−bisphosphoglycer...
0.000306
2 / 9

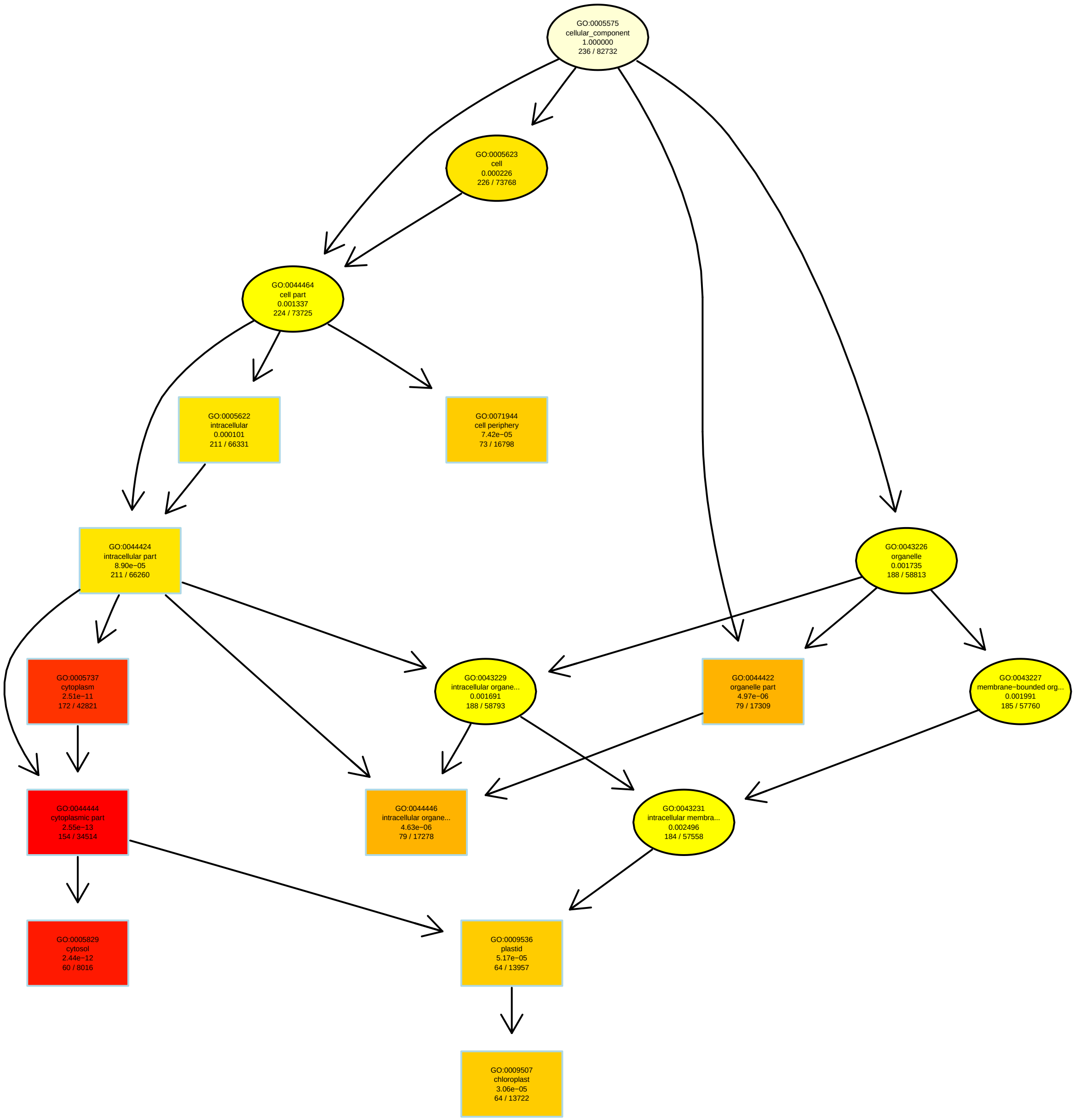
GO:0005575
cellular_component
1.000000
236 / 82732
GO:0005623
cell
0.000226
226 / 73768
GO:0044464
cell part
0.001337
224 / 73725
GO:0005622
intracellular
0.000101
211 / 66331
GO:0071944
cell periphery
7.42e−05
73 / 16798
GO:0044424
intracellular part
8.90e−05
211 / 66260
GO:0043226
organelle
0.001735
188 / 58813
GO:0005737
cytoplasm
2.51e−11
172 / 42821
GO:0043229
intracellular organe...
0.001691
188 / 58793
GO:0044422
organelle part
4.97e−06
79 / 17309
GO:0043227
membrane−bounded org...
0.001991
185 / 57760
GO:0044444
cytoplasmic part
2.55e−13
154 / 34514
GO:0044446
intracellular organe...
4.63e−06
79 / 17278
GO:0043231
intracellular membra...
0.002496
184 / 57558
GO:0005829
cytosol
2.44e−12
60 / 8016
GO:0009536
plastid
5.17e−05
64 / 13957
GO:0009507
chloroplast
3.06e−05
64 / 13722
