# Supplementary material for: Differential Alternative Splicing Genes and Isoform Regulation Networks of Rapeseed (Brassica napus L.) Infected with Sclerotinia sclerotiorum
Source: Genes (Basel). 2020 Jul 13;11(7):784. doi: 10.3390/genes11070784 (PMC7397149; doi:10.3390/genes11070784)
Supplement: Supplementary file 1 [file genes-11-00784-s001.zip › Fig. S2 Enriched GO terms of genes in the 85 WGCNA modules.zip!/Fig. S2/CytoNodeskyblue1-go.pdf]

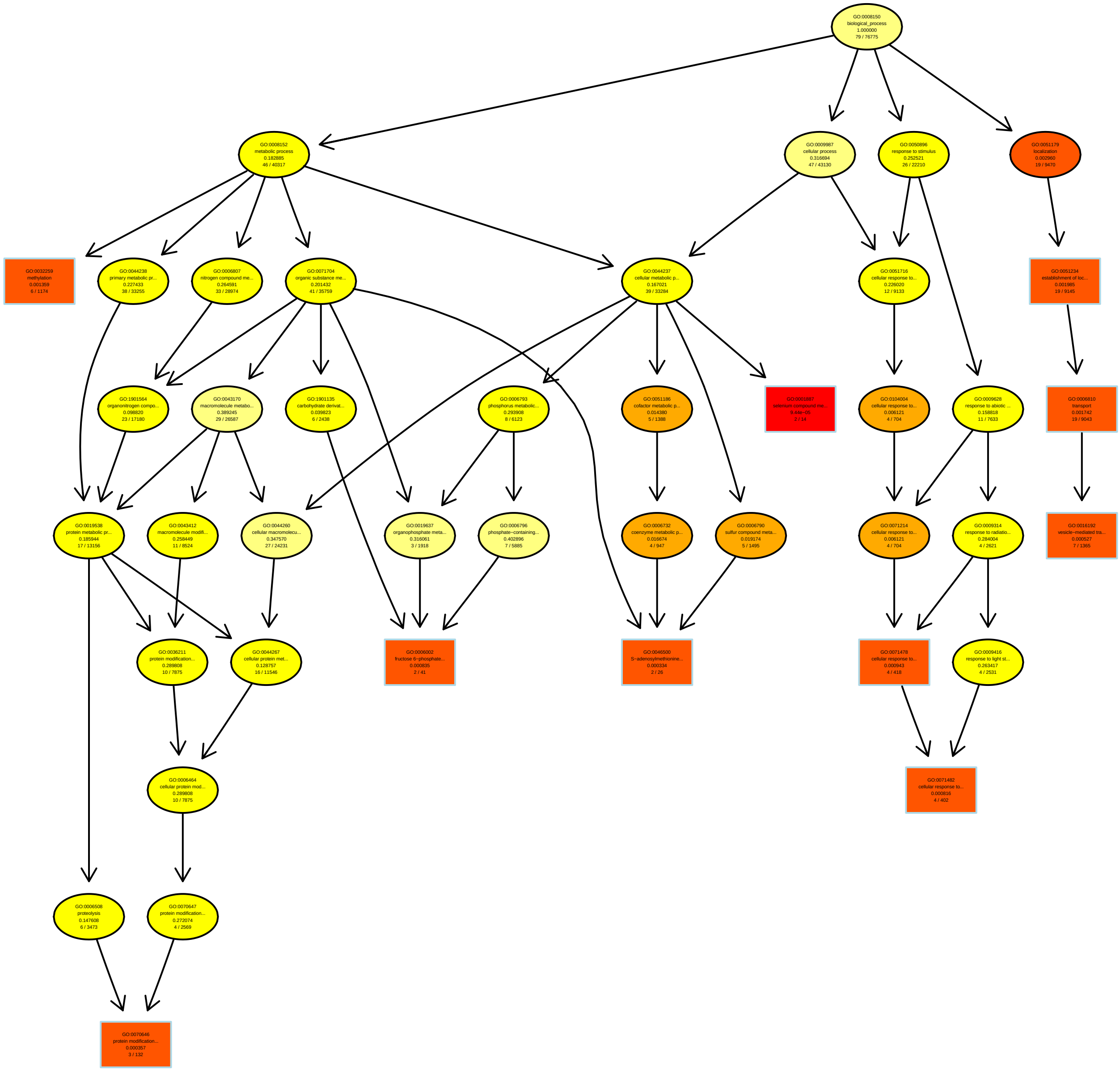
GO:0008150 biological_process 1.000000 79 / 76775
GO:0008152 metabolic process 0.182885 46 / 40317
GO:0009987 cellular process 0.316694 47 / 43130
GO:0050896 response to stimulus 0.252521 26 / 22210
GO:0051179 localization 0.002960 19 / 9470
GO:0032259 methylation 0.001359 6 / 1174
GO:0044238 primary metabolic pr... 0.227433 38 / 33255
GO:0006807 nitrogen compound me... 0.264591 33 / 28974
GO:0071704 organic substance me... 0.201432 41 / 35759
GO:0044237 cellular metabolic p... 0.167021 39 / 33284
GO:0051716 cellular response to... 0.226020 12 / 9133
GO:0051234 establishment of loc... 0.001985 19 / 9145
GO:1901564 organonitrogen compo... 0.098820 23 / 17180
GO:0043170 macromolecule metabo... 0.389245 29 / 26587
GO:1901135 carbohydrate derivat... 0.039823 6 / 2438
GO:0006793 phosphorus metabolic... 0.293908 8 / 6123
GO:0051186 cofactor metabolic p... 0.014380 5 / 1388
GO:0001887 selenium compound me... 9.44e−05 2 / 14
GO:0104004 cellular response to... 0.006121 4 / 704
GO:0009628 response to abiotic ... 0.158818 11 / 7633
GO:0006810 transport 0.001742 19 / 9043
GO:0019538 protein metabolic pr... 0.185944 17 / 13156
GO:0043412 macromolecule modifi... 0.258449 11 / 8524
GO:0044260 cellular macromolecu... 0.347570 27 / 24231
GO:0019637 organophosphate meta... 0.316061 3 / 1918
GO:0006796 phosphate−containing... 0.402896 7 / 5885
GO:0006732 coenzyme metabolic p... 0.016674 4 / 947
GO:0006790 sulfur compound meta... 0.019174 5 / 1495
GO:0071214 cellular response to... 0.006121 4 / 704
GO:0009314 response to radiatio... 0.284004 4 / 2621
GO:0016192 vesicle−mediated tra... 0.000527 7 / 1365
GO:0036211 protein modification... 0.289808 10 / 7875
GO:0044267 cellular protein met... 0.128757 16 / 11546
GO:0006002 fructose 6−phosphate... 0.000835 2 / 41
GO:0046500 S−adenosylmethionine... 0.000334 2 / 26
GO:0071478 cellular response to... 0.000943 4 / 418
GO:0009416 response to light st... 0.263417 4 / 2531
GO:0006464 cellular protein mod... 0.289808 10 / 7875
GO:0071482 cellular response to... 0.000816 4 / 402
GO:0006508 proteolysis 0.147608 6 / 3473
GO:0070647 protein modification... 0.272074 4 / 2569
GO:0070646 protein modification... 0.000357 3 / 132

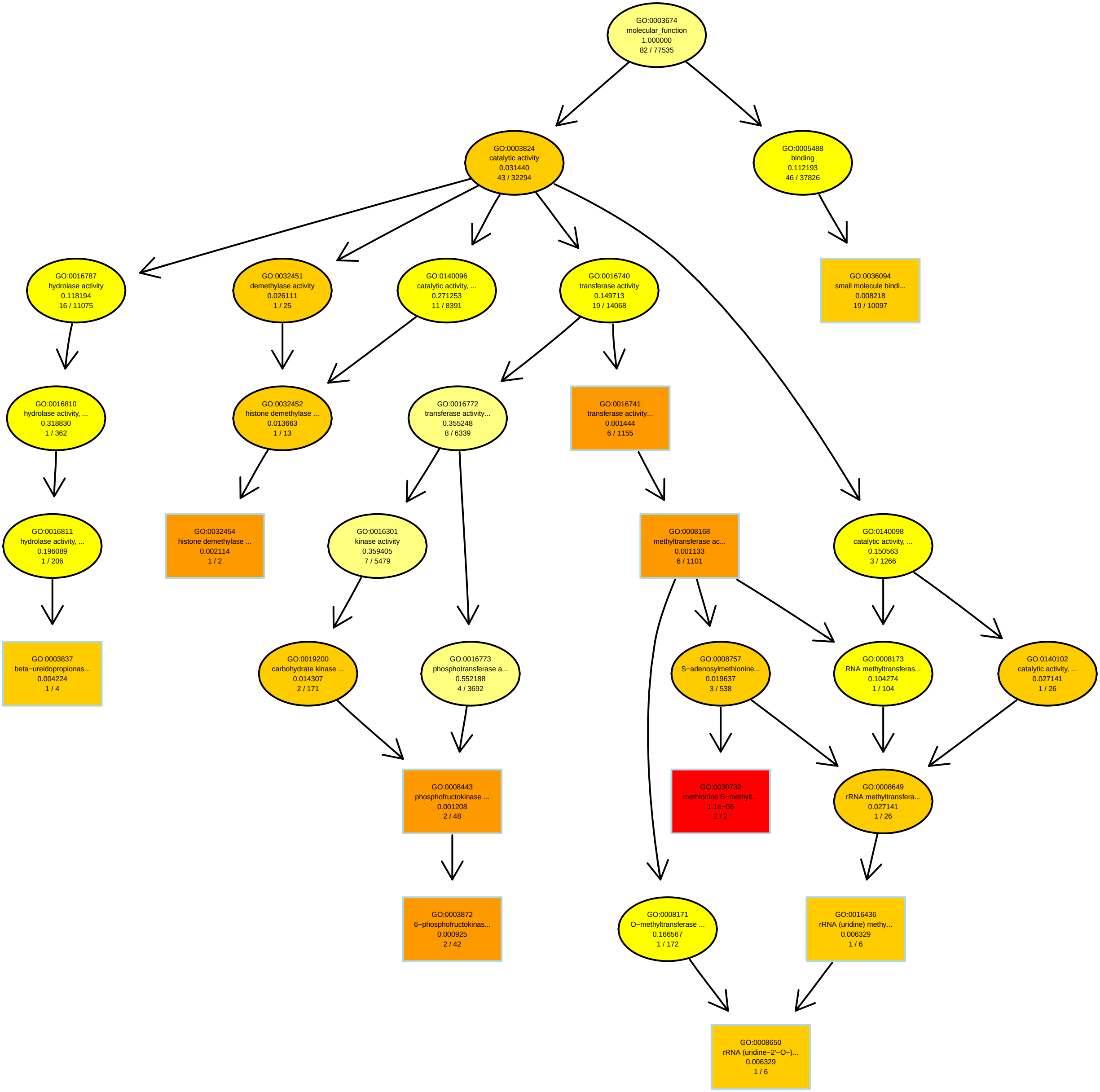
GO:0003674 molecular_function 1.000000 82 / 77535
GO:0003824 catalytic activity 0.031440 43 / 32294
GO:0005488 binding 0.112193 46 / 37826
GO:0016787 hydrolase activity 0.118194 16 / 11075
GO:0032451 demethylase activity 0.026111 1 / 25
GO:0140096 catalytic activity, ... 0.271253 11 / 8391
GO:0016740 transferase activity 0.149713 19 / 14068
GO:0036094 small molecule bindi... 0.008218 19 / 10097
GO:0016810 hydrolase activity, ... 0.318830 1 / 362
GO:0032452 histone demethylase ... 0.013663 1 / 13
GO:0016772 transferase activity... 0.355248 8 / 6339
GO:0016741 transferase activity... 0.001444 6 / 1155
GO:0016811 hydrolase activity, ... 0.196089 1 / 206
GO:0032454 histone demethylase ... 0.002114 1 / 2
GO:0016301 kinase activity 0.359405 7 / 5479
GO:0008168 methyltransferase ac... 0.001133 6 / 1101
GO:0140098 catalytic activity, ... 0.150563 3 / 1266
GO:0003837 beta−ureidopropionas... 0.004224 1 / 4
GO:0019200 carbohydrate kinase ... 0.014307 2 / 171
GO:0016773 phosphotransferase a... 0.552188 4 / 3692
GO:0008757 S−adenosylmethionine... 0.019637 3 / 538
GO:0008173 RNA methyltransferas... 0.104274 1 / 104
GO:0140102 catalytic activity, ... 0.027141 1 / 26
GO:0008443 phosphofructokinase ... 0.001208 2 / 48
GO:0030732 methionine S−methylt... 1.1e−05 2 / 2
GO:0008649 rRNA methyltransfera... 0.027141 1 / 26
GO:0003872 6−phosphofructokinas... 0.000925 2 / 42
GO:0008171 O−methyltransferase ... 0.166567 1 / 172
GO:0016436 rRNA (uridine) methy... 0.006329 1 / 6
GO:0008650 rRNA (uridine−2'−O−)... 0.006329 1 / 6

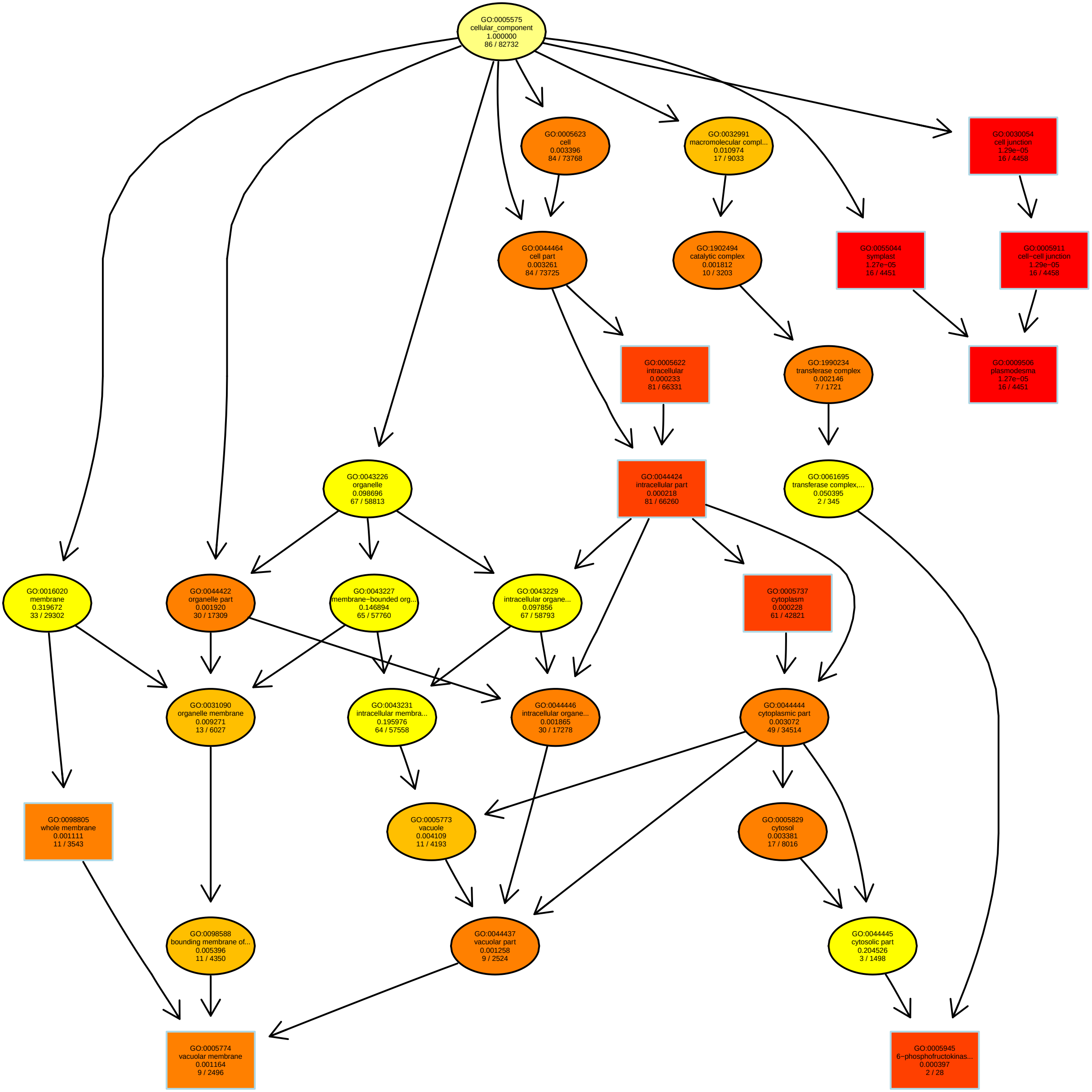
GO:0005575
cellular_component
1.000000
86 / 82732
GO:0005623
cell
0.003396
84 / 73768
GO:0032991
macromolecular compl...
0.010974
17 / 9033
GO:0030054
cell junction
1.29e−05
16 / 4458
GO:0044464
cell part
0.003261
84 / 73725
GO:1902494
catalytic complex
0.001812
10 / 3203
GO:0055044
symplast
1.27e−05
16 / 4451
GO:0005911
cell−cell junction
1.29e−05
16 / 4458
GO:0005622
intracellular
0.000233
81 / 66331
GO:1990234
transferase complex
0.002146
7 / 1721
GO:0009506
plasmodesma
1.27e−05
16 / 4451
GO:0043226
organelle
0.098696
67 / 58813
GO:0044424
intracellular part
0.000218
81 / 66260
GO:0061695
transferase complex,...
0.050395
2 / 345
GO:0016020
membrane
0.319672
33 / 29302
GO:0044422
organelle part
0.001920
30 / 17309
GO:0043227
membrane−bounded org...
0.146894
65 / 57760
GO:0043229
intracellular organe...
0.097856
67 / 58793
GO:0005737
cytoplasm
0.000228
61 / 42821
GO:0031090
organelle membrane
0.009271
13 / 6027
GO:0043231
intracellular membra...
0.195976
64 / 57558
GO:0044446
intracellular organe...
0.001865
30 / 17278
GO:0044444
cytoplasmic part
0.003072
49 / 34514
GO:0098805
whole membrane
0.001111
11 / 3543
GO:0005773
vacuole
0.004109
11 / 4193
GO:0005829
cytosol
0.003381
17 / 8016
GO:0098588
bounding membrane of...
0.005396
11 / 4350
GO:0044437
vacuolar part
0.001258
9 / 2524
GO:0044445
cytosolic part
0.204526
3 / 1498
GO:0005774
vacuolar membrane
0.001164
9 / 2496
GO:0005945
6−phosphofructokinas...
0.000397
2 / 28
