# Supplementary material for: Differential Alternative Splicing Genes and Isoform Regulation Networks of Rapeseed (Brassica napus L.) Infected with Sclerotinia sclerotiorum
Source: Genes (Basel). 2020 Jul 13;11(7):784. doi: 10.3390/genes11070784 (PMC7397149; doi:10.3390/genes11070784)
Supplement: Supplementary file 1 [file genes-11-00784-s001.zip › Fig. S2 Enriched GO terms of genes in the 85 WGCNA modules.zip!/Fig. S2/CytoNodeskyblue2-go.pdf]

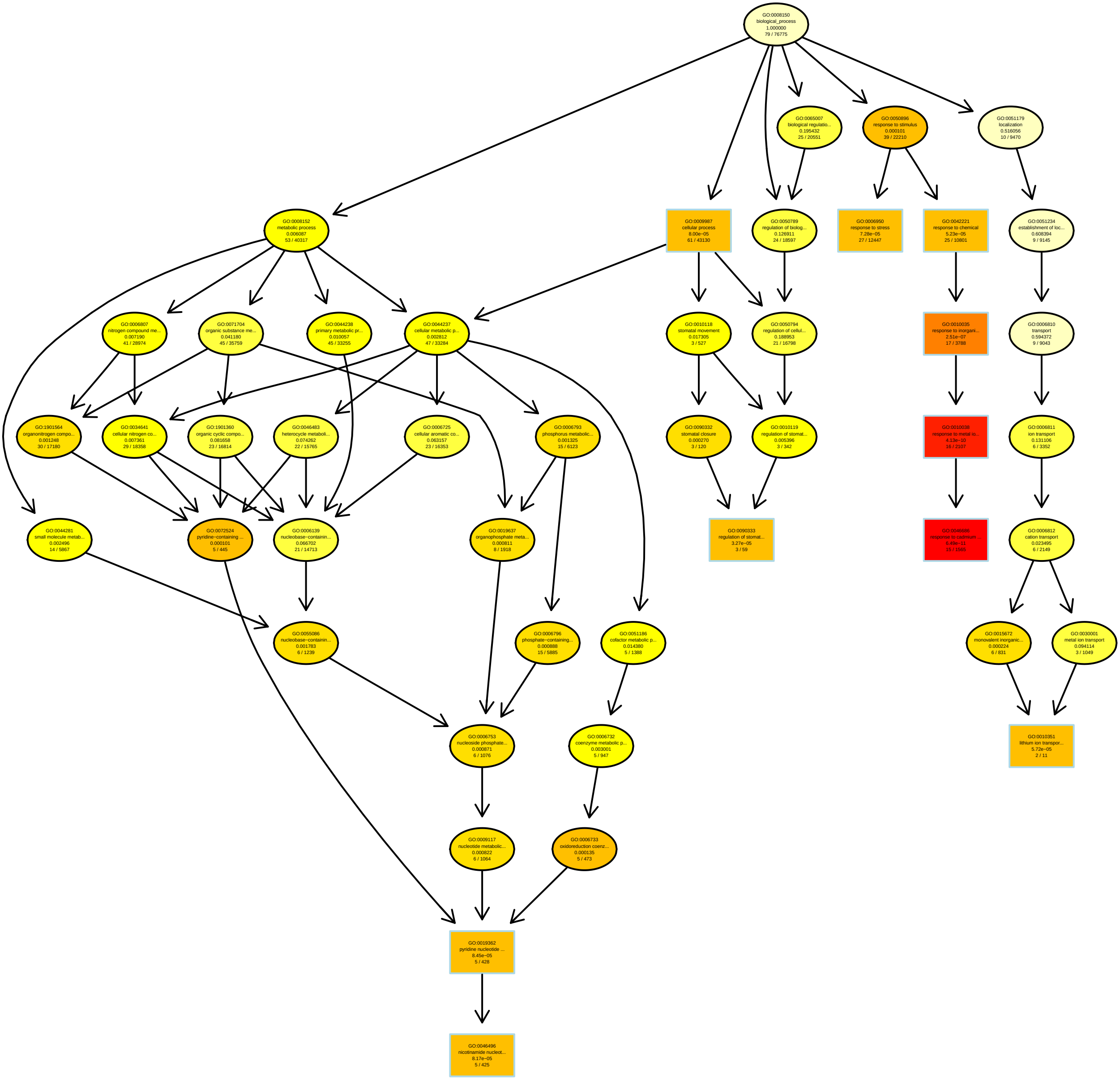
GO:0008150 biological_process 1.000000 79 / 76775
GO:0065007 biological regulatio... 0.195432 25 / 20551
GO:0050896 response to stimulus 0.000101 39 / 22210
GO:0051179 localization 0.516056 10 / 9470
GO:0008152 metabolic process 0.006087 53 / 40317
GO:0009987 cellular process 8.00e−05 61 / 43130
GO:0050789 regulation of biolog... 0.126911 24 / 18597
GO:0006950 response to stress 7.28e−05 27 / 12447
GO:0042221 response to chemical 5.23e−05 25 / 10801
GO:0051234 establishment of loc... 0.608394 9 / 9145
GO:0006807 nitrogen compound me... 0.007190 41 / 28974
GO:0071704 organic substance me... 0.041180 45 / 35759
GO:0044238 primary metabolic pr... 0.010057 45 / 33255
GO:0044237 cellular metabolic p... 0.002812 47 / 33284
GO:0010118 stomatal movement 0.017305 3 / 527
GO:0050794 regulation of cellul... 0.188953 21 / 16798
GO:0010035 response to inorgani... 2.51e−07 17 / 3788
GO:0006810 transport 0.594372 9 / 9043
GO:1901564 organonitrogen compo... 0.001248 30 / 17180
GO:0034641 cellular nitrogen co... 0.007361 29 / 18358
GO:1901360 organic cyclic compo... 0.081658 23 / 16814
GO:0046483 heterocycle metaboli... 0.074262 22 / 15765
GO:0006725 cellular aromatic co... 0.063157 23 / 16353
GO:0006793 phosphorus metabolic... 0.001325 15 / 6123
GO:0090332 stomatal closure 0.000270 3 / 120
GO:0010119 regulation of stomat... 0.005396 3 / 342
GO:0010038 response to metal io... 4.13e−10 16 / 2107
GO:0006811 ion transport 0.131106 6 / 3352
GO:0044281 small molecule metab... 0.002496 14 / 5867
GO:0072524 pyridine−containing ... 0.000101 5 / 445
GO:0006139 nucleobase−containin... 0.066702 21 / 14713
GO:0019637 organophosphate meta... 0.000811 8 / 1918
GO:0090333 regulation of stomat... 3.27e−05 3 / 59
GO:0046686 response to cadmium ... 6.49e−11 15 / 1565
GO:0006812 cation transport 0.023495 6 / 2149
GO:0055086 nucleobase−containin... 0.001783 6 / 1239
GO:0006796 phosphate−containing... 0.000888 15 / 5885
GO:0051186 cofactor metabolic p... 0.014380 5 / 1388
GO:0015672 monovalent inorganic... 0.000224 6 / 831
GO:0030001 metal ion transport 0.094114 3 / 1049
GO:0006753 nucleoside phosphate... 0.000871 6 / 1076
GO:0006732 coenzyme metabolic p... 0.003001 5 / 947
GO:0010351 lithium ion transpor... 5.72e−05 2 / 11
GO:0009117 nucleotide metabolic... 0.000822 6 / 1064
GO:0006733 oxidoreduction coenz... 0.000135 5 / 473
GO:0019362 pyridine nucleotide ... 8.45e−05 5 / 428
GO:0046496 nicotinamide nucleot... 8.17e−05 5 / 425

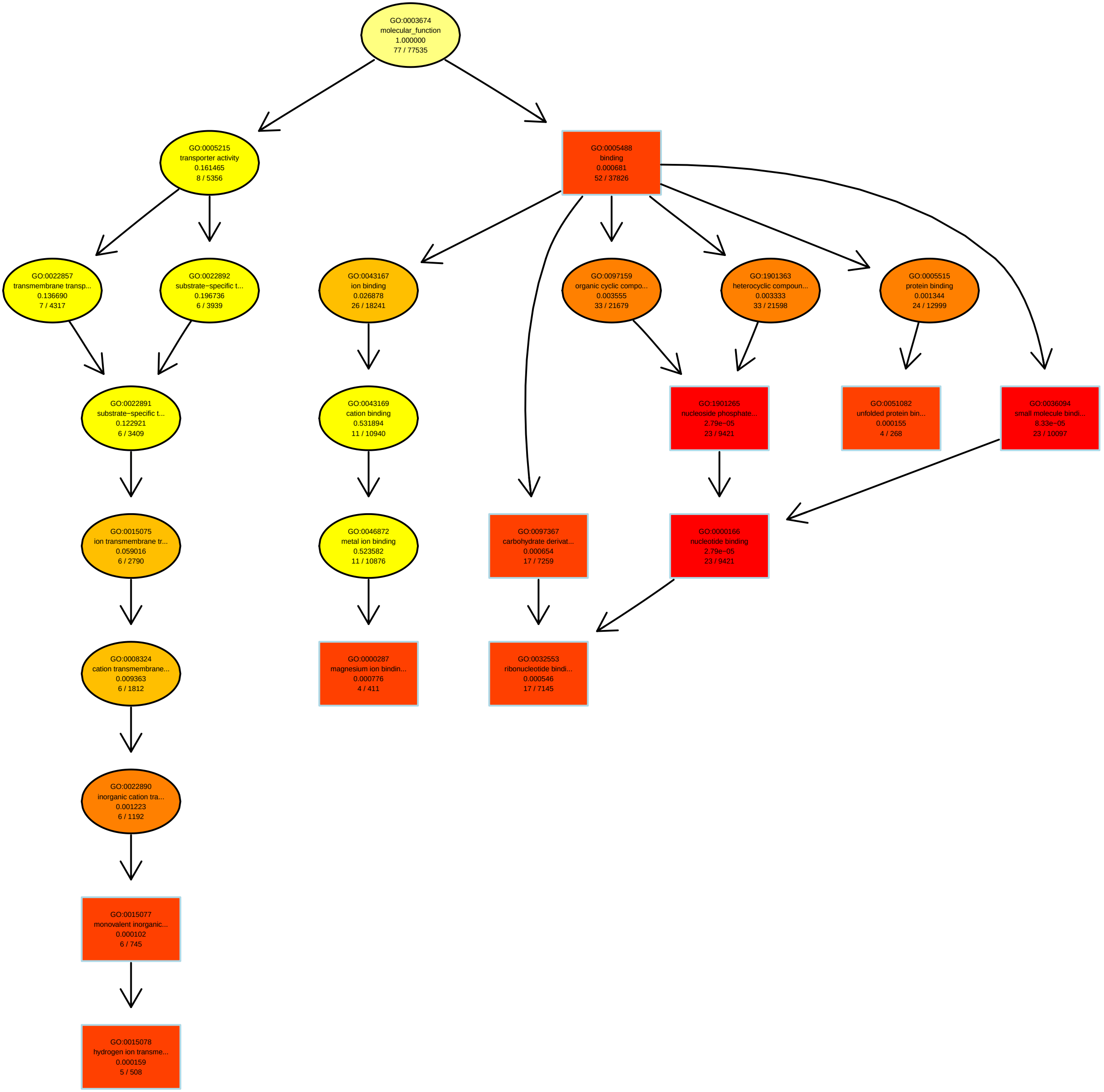
GO:0003674
molecular_function
1.000000
77 / 77535
GO:0005215
transporter activity
0.161465
8 / 5356
GO:0005488
binding
0.000681
52 / 37826
GO:0022857
transmembrane transp...
0.136690
7 / 4317
GO:0022892
substrate−specific t...
0.196736
6 / 3939
GO:0043167
ion binding
0.026878
26 / 18241
GO:0097159
organic cyclic compo...
0.003555
33 / 21679
GO:1901363
heterocyclic compoun...
0.003333
33 / 21598
GO:0005515
protein binding
0.001344
24 / 12999
GO:0022891
substrate−specific t...
0.122921
6 / 3409
GO:0043169
cation binding
0.531894
11 / 10940
GO:1901265
nucleoside phosphate...
2.79e−05
23 / 9421
GO:0051082
unfolded protein bin...
0.000155
4 / 268
GO:0036094
small molecule bindi...
8.33e−05
23 / 10097
GO:0015075
ion transmembrane tr...
0.059016
6 / 2790
GO:0046872
metal ion binding
0.523582
11 / 10876
GO:0097367
carbohydrate derivat...
0.000654
17 / 7259
GO:0000166
nucleotide binding
2.79e−05
23 / 9421
GO:0008324
cation transmembrane...
0.009363
6 / 1812
GO:0000287
magnesium ion bindin...
0.000776
4 / 411
GO:0032553
ribonucleotide bindi...
0.000546
17 / 7145
GO:0022890
inorganic cation tra...
0.001223
6 / 1192
GO:0015077
monovalent inorganic...
0.000102
6 / 745
GO:0015078
hydrogen ion transme...
0.000159
5 / 508

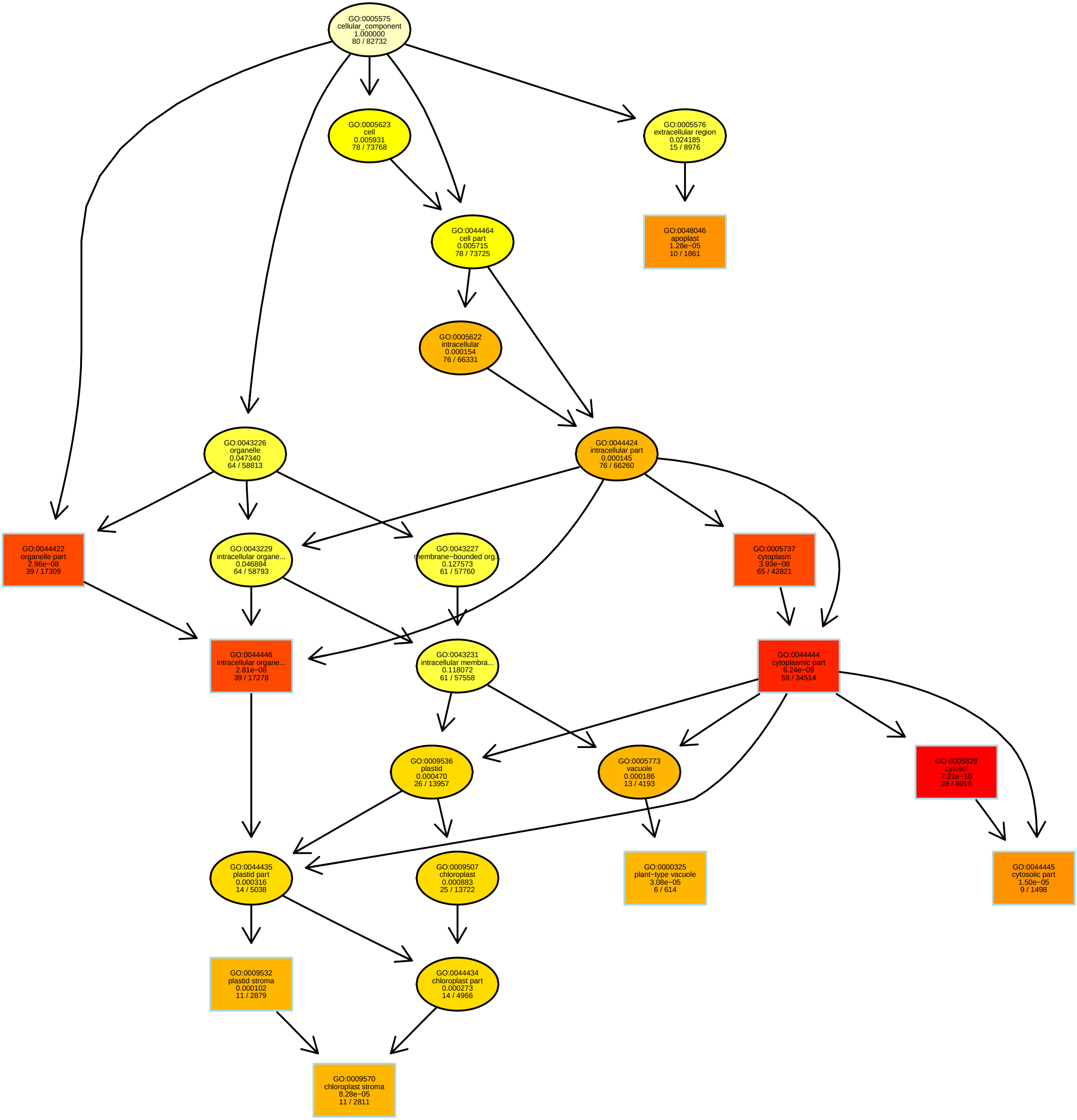
GO:0005575
cellular_component
1.000000
80 / 82732
GO:0005623
cell
0.005931
78 / 73768
GO:0005576
extracellular region
0.024185
15 / 8976
GO:0044464
cell part
0.005715
78 / 73725
GO:0048046
apoplast
1.28e−05
10 / 1861
GO:0005622
intracellular
0.000154
76 / 66331
GO:0043226
organelle
0.047340
64 / 58813
GO:0044424
intracellular part
0.000145
76 / 66260
GO:0044422
organelle part
2.96e−08
39 / 17309
GO:0043229
intracellular organe...
0.046884
64 / 58793
GO:0043227
membrane−bounded org...
0.127573
61 / 57760
GO:0005737
cytoplasm
3.93e−08
65 / 42821
GO:0044446
intracellular organe...
2.81e−08
39 / 17278
GO:0043231
intracellular membra...
0.118072
61 / 57558
GO:0044444
cytoplasmic part
6.24e−09
59 / 34514
GO:0009536
plastid
0.000470
26 / 13957
GO:0005773
vacuole
0.000186
13 / 4193
GO:0005829
cytosol
7.21e−10
28 / 8016
GO:0044435
plastid part
0.000316
14 / 5038
GO:0009507
chloroplast
0.000883
25 / 13722
GO:0000325
plant−type vacuole
3.08e−05
6 / 614
GO:0044445
cytosolic part
1.50e−05
9 / 1498
GO:0009532
plastid stroma
0.000102
11 / 2879
GO:0044434
chloroplast part
0.000273
14 / 4966
GO:0009570
chloroplast stroma
8.28e−05
11 / 2811
