# Supplementary material for: Differential Alternative Splicing Genes and Isoform Regulation Networks of Rapeseed (Brassica napus L.) Infected with Sclerotinia sclerotiorum
Source: Genes (Basel). 2020 Jul 13;11(7):784. doi: 10.3390/genes11070784 (PMC7397149; doi:10.3390/genes11070784)
Supplement: Supplementary file 1 [file genes-11-00784-s001.zip › Fig. S2 Enriched GO terms of genes in the 85 WGCNA modules.zip!/Fig. S2/CytoNodeskyblue3-go.pdf]

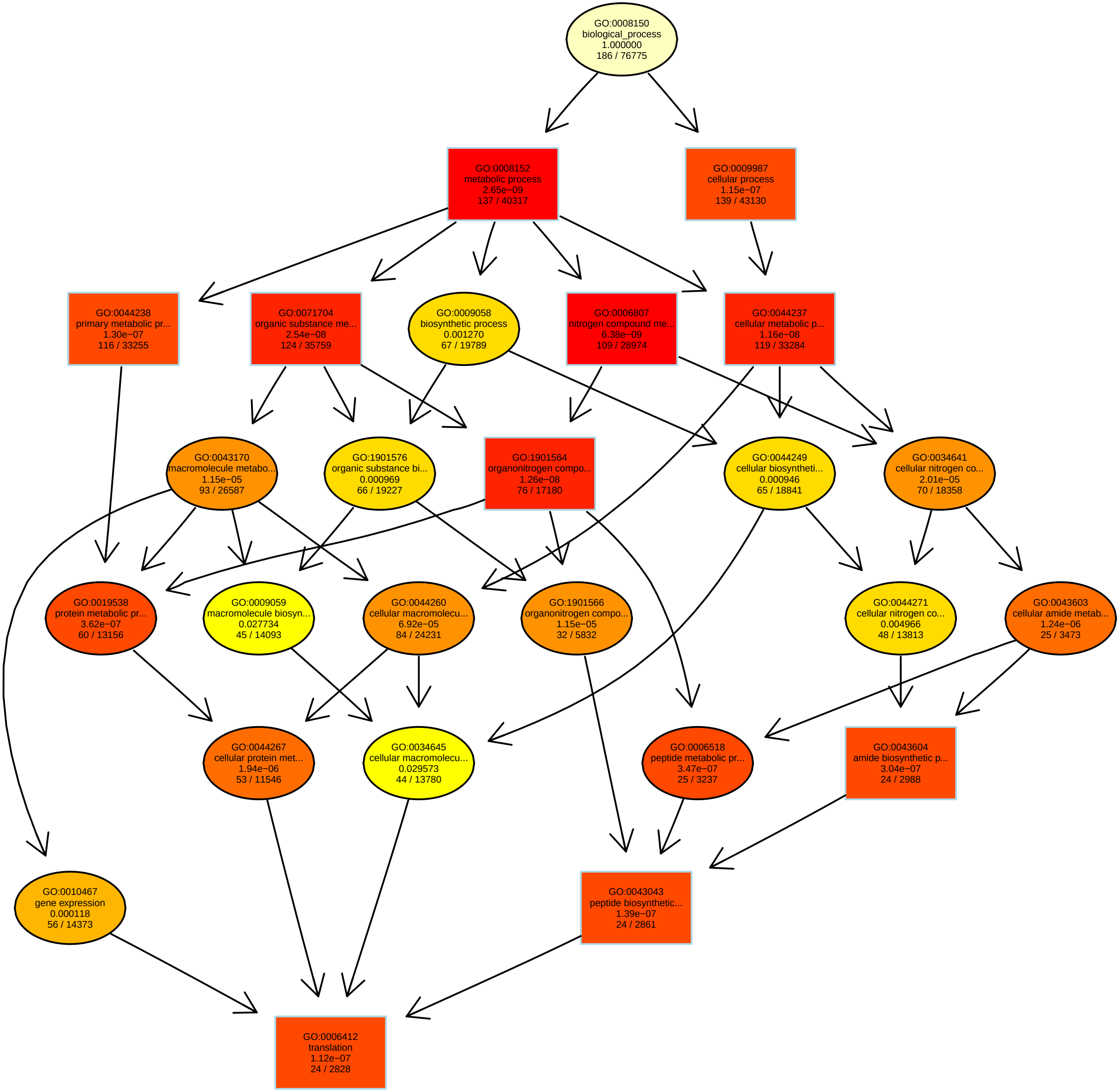
GO:0008150
biological_process
1.000000
186 / 76775
GO:0008152
metabolic process
2.65e−09
137 / 40317
GO:0009987
cellular process
1.15e−07
139 / 43130
GO:0044238
primary metabolic pr...
1.30e−07
116 / 33255
GO:0071704
organic substance me...
2.54e−08
124 / 35759
GO:0009058
biosynthetic process
0.001270
67 / 19789
GO:0006807
nitrogen compound me...
6.38e−09
109 / 28974
GO:0044237
cellular metabolic p...
1.16e−08
119 / 33284
GO:0043170
macromolecule metabo...
1.15e−05
93 / 26587
GO:1901576
organic substance bi...
0.000969
66 / 19227
GO:1901564
organonitrogen compo...
1.26e−08
76 / 17180
GO:0044249
cellular biosyntheti...
0.000946
65 / 18841
GO:0034641
cellular nitrogen co...
2.01e−05
70 / 18358
GO:0019538
protein metabolic pr...
3.62e−07
60 / 13156
GO:0009059
macromolecule biosyn...
0.027734
45 / 14093
GO:0044260
cellular macromolecu...
6.92e−05
84 / 24231
GO:1901566
organonitrogen compo...
1.15e−05
32 / 5832
GO:0044271
cellular nitrogen co...
0.004966
48 / 13813
GO:0043603
cellular amide metab...
1.24e−06
25 / 3473
GO:0044267
cellular protein met...
1.94e−06
53 / 11546
GO:0034645
cellular macromolecu...
0.029573
44 / 13780
GO:0006518
peptide metabolic pr...
3.47e−07
25 / 3237
GO:0043604
amide biosynthetic p...
3.04e−07
24 / 2988
GO:0010467
gene expression
0.000118
56 / 14373
GO:0043043
peptide biosynthetic...
1.39e−07
24 / 2861
GO:0006412
translation
1.12e−07
24 / 2828

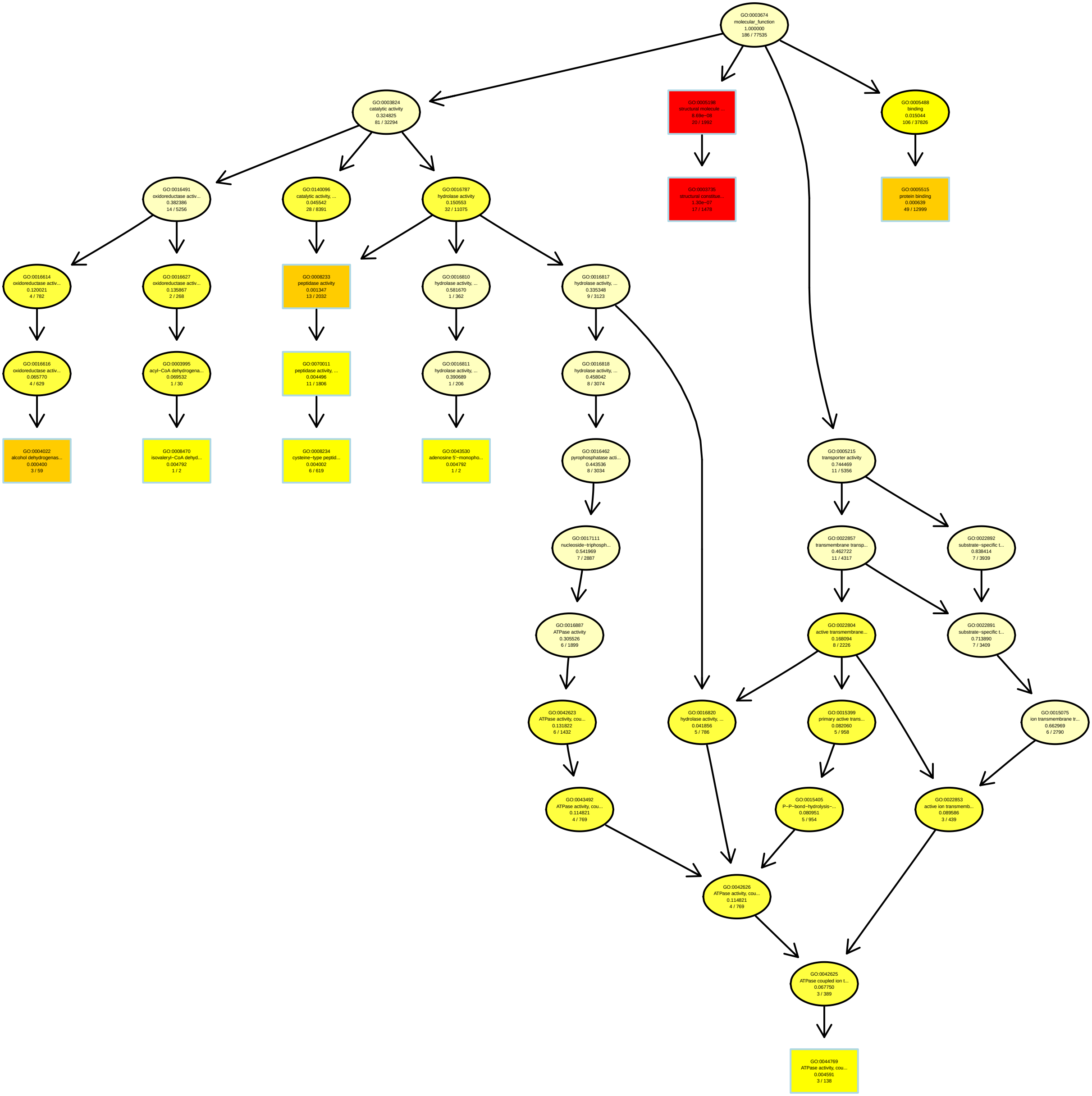
GO:0003674 molecular_function 1.000000 186 / 77535
GO:0003824 catalytic activity 0.324825 81 / 32294
GO:0005198 structural molecule ... 8.69e−08 20 / 1992
GO:0005488 binding 0.015044 106 / 37826
GO:0016491 oxidoreductase activ... 0.382386 14 / 5256
GO:0140096 catalytic activity, ... 0.045542 28 / 8391
GO:0016787 hydrolase activity 0.150553 32 / 11075
GO:0003735 structural constitue... 1.30e−07 17 / 1478
GO:0005515 protein binding 0.000639 49 / 12999
GO:0016614 oxidoreductase activ... 0.120021 4 / 782
GO:0016627 oxidoreductase activ... 0.135867 2 / 268
GO:0008233 peptidase activity 0.001347 13 / 2032
GO:0016810 hydrolase activity, ... 0.581670 1 / 362
GO:0016817 hydrolase activity, ... 0.335348 9 / 3123
GO:0016616 oxidoreductase activ... 0.065770 4 / 629
GO:0003995 acyl−CoA dehydrogena... 0.069532 1 / 30
GO:0070011 peptidase activity, ... 0.004496 11 / 1806
GO:0016811 hydrolase activity, ... 0.390689 1 / 206
GO:0016818 hydrolase activity, ... 0.458042 8 / 3074
GO:0004022 alcohol dehydrogenas... 0.000400 3 / 59
GO:0008470 isovaleryl−CoA dehyd... 0.004792 1 / 2
GO:0008234 cysteine−type peptid... 0.004002 6 / 619
GO:0043530 adenosine 5'−monopho... 0.004792 1 / 2
GO:0016462 pyrophosphatase acti... 0.443536 8 / 3034
GO:0005215 transporter activity 0.744469 11 / 5356
GO:0017111 nucleoside−triphosph... 0.541969 7 / 2887
GO:0022857 transmembrane transp... 0.462722 11 / 4317
GO:0022892 substrate−specific t... 0.838414 7 / 3939
GO:0016887 ATPase activity 0.305526 6 / 1899
GO:0022804 active transmembrane... 0.168094 8 / 2226
GO:0022891 substrate−specific t... 0.713890 7 / 3409
GO:0042623 ATPase activity, cou... 0.131822 6 / 1432
GO:0016820 hydrolase activity, ... 0.041856 5 / 786
GO:0015399 primary active trans... 0.082060 5 / 958
GO:0015075 ion transmembrane tr... 0.662969 6 / 2790
GO:0043492 ATPase activity, cou... 0.114821 4 / 769
GO:0015405 P−P−bond−hydrolysis−... 0.080951 5 / 954
GO:0022853 active ion transmemb... 0.089586 3 / 439
GO:0042626 ATPase activity, cou... 0.114821 4 / 769
GO:0042625 ATPase coupled ion t... 0.067750 3 / 389
GO:0044769 ATPase activity, cou... 0.004591 3 / 138

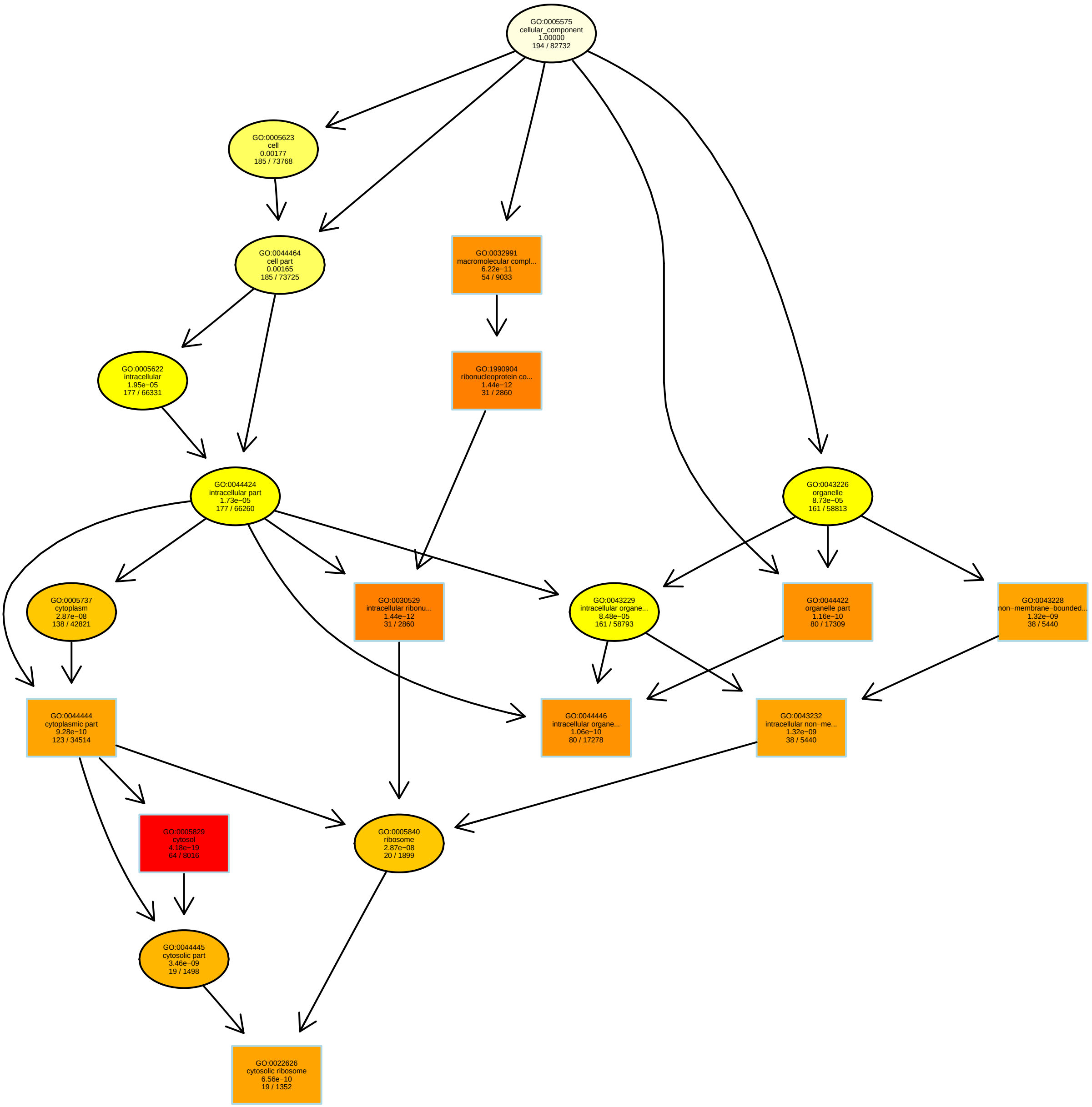
GO:0005575
cellular_component
1.00000
194 / 82732
GO:0005623
cell
0.00177
185 / 73768
GO:0044464
cell part
0.00165
185 / 73725
GO:0032991
macromolecular compl...
6.22e−11
54 / 9033
GO:1990904
ribonucleoprotein co...
1.44e−12
31 / 2860
GO:0005622
intracellular
1.95e−05
177 / 66331
GO:0044424
intracellular part
1.73e−05
177 / 66260
GO:0043226
organelle
8.73e−05
161 / 58813
GO:0005737
cytoplasm
2.87e−08
138 / 42821
GO:0030529
intracellular ribonu...
1.44e−12
31 / 2860
GO:0043229
intracellular organe...
8.48e−05
161 / 58793
GO:0044422
organelle part
1.16e−10
80 / 17309
GO:0043228
non−membrane−bounded...
1.32e−09
38 / 5440
GO:0044444
cytoplasmic part
9.28e−10
123 / 34514
GO:0044446
intracellular organe...
1.06e−10
80 / 17278
GO:0043232
intracellular non−me...
1.32e−09
38 / 5440
GO:0005829
cytosol
4.18e−19
64 / 8016
GO:0005840
ribosome
2.87e−08
20 / 1899
GO:0044445
cytosolic part
3.46e−09
19 / 1498
GO:0022626
cytosolic ribosome
6.56e−10
19 / 1352
