# Supplementary material for: Differential Alternative Splicing Genes and Isoform Regulation Networks of Rapeseed (Brassica napus L.) Infected with Sclerotinia sclerotiorum
Source: Genes (Basel). 2020 Jul 13;11(7):784. doi: 10.3390/genes11070784 (PMC7397149; doi:10.3390/genes11070784)
Supplement: Supplementary file 1 [file genes-11-00784-s001.zip › Fig. S2 Enriched GO terms of genes in the 85 WGCNA modules.zip!/Fig. S2/CytoNodesteelblue-go.pdf]

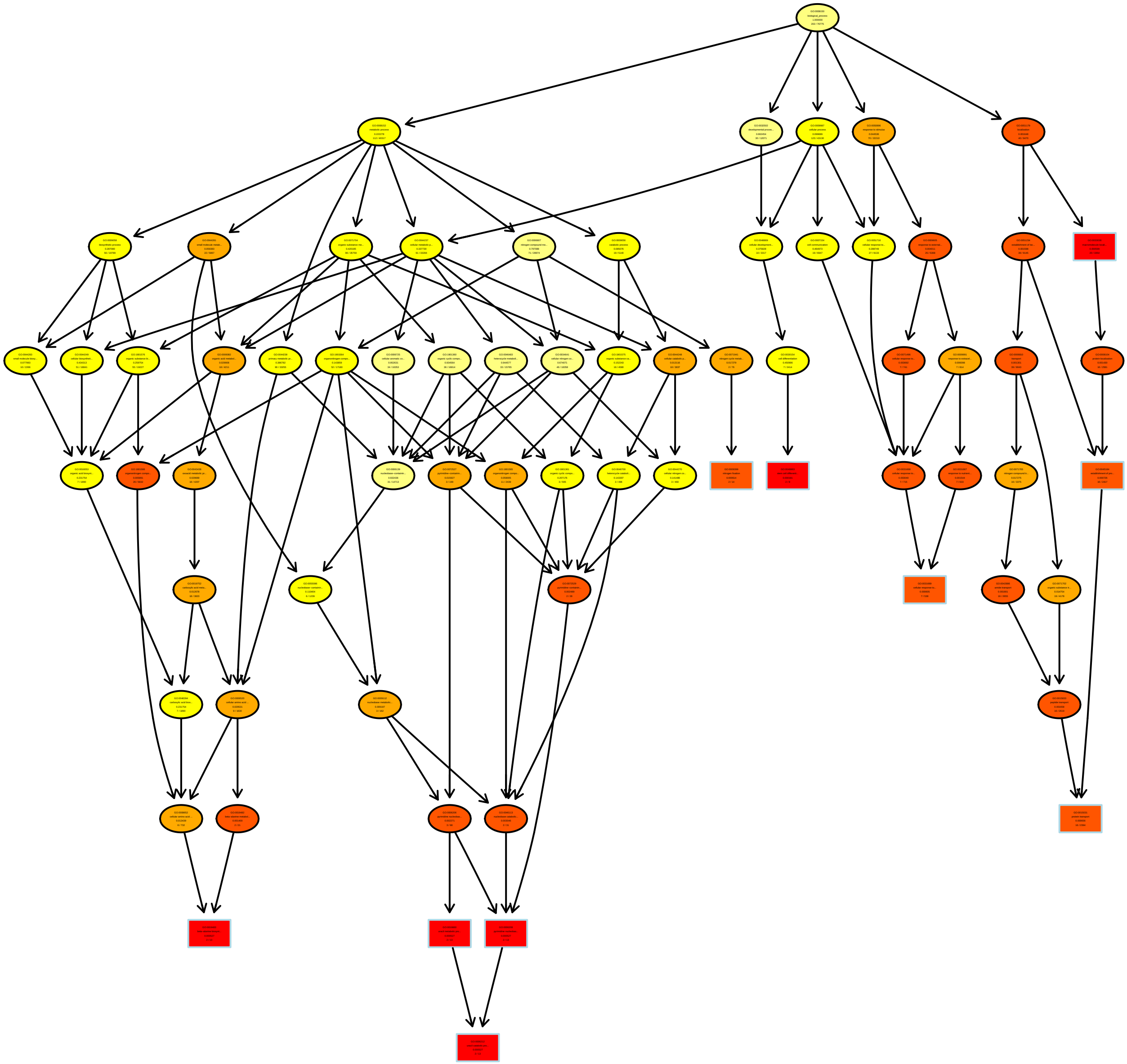

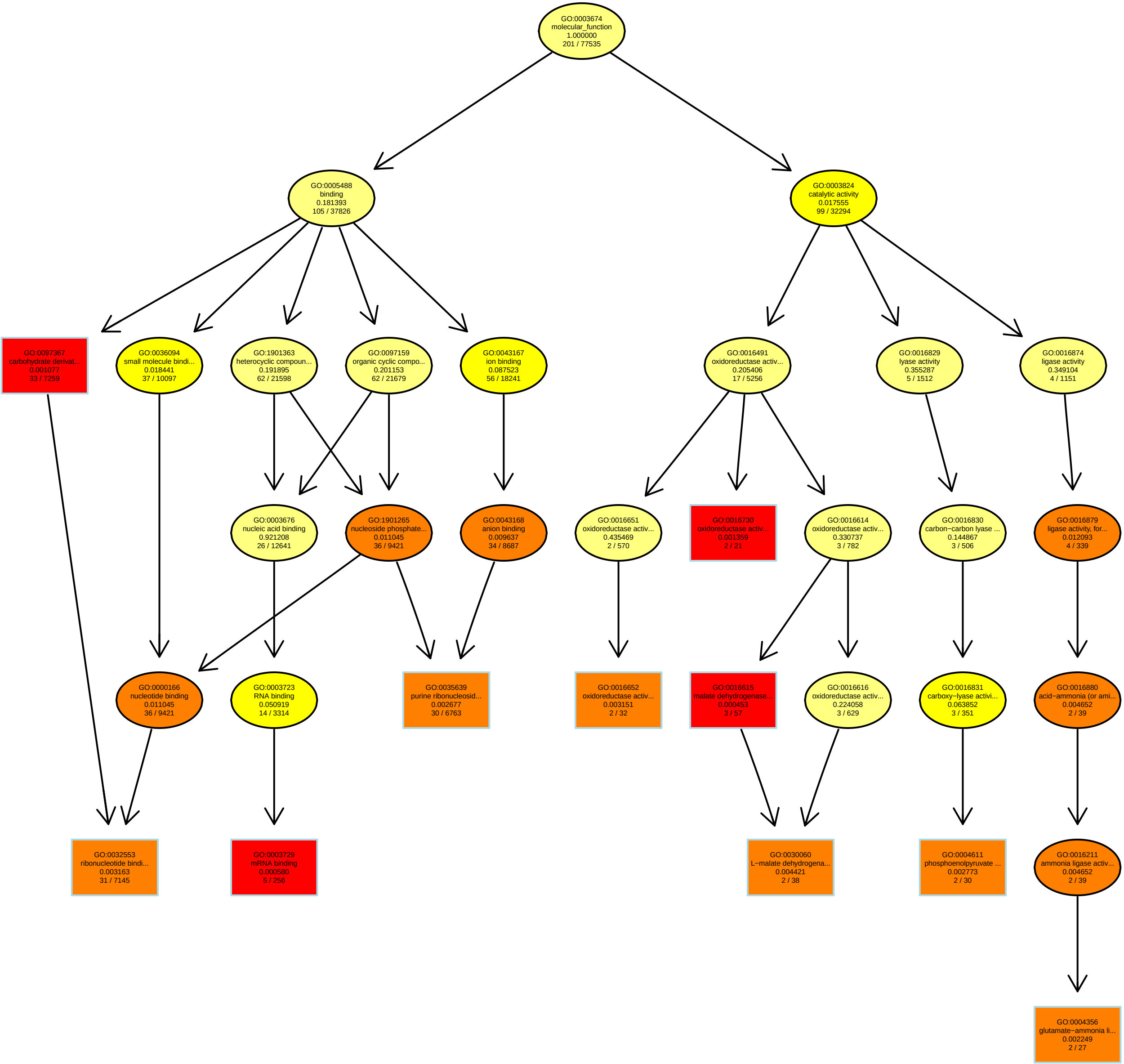

GO:0003674 molecular_function 1.000000 201 / 77535
GO:0005488 binding 0.181393 105 / 37826
GO:0003824 catalytic activity 0.017555 99 / 32294
GO:0097367 carbohydrate derivat... 0.001077 33 / 7259
GO:0036094 small molecule bindi... 0.018441 37 / 10097
GO:1901363 heterocyclic compoun... 0.191895 62 / 21598
GO:0097159 organic cyclic compo... 0.201153 62 / 21679
GO:0043167 ion binding 0.087523 56 / 18241
GO:0016491 oxidoreductase activ... 0.205406 17 / 5256
GO:0016829 lyase activity 0.355287 5 / 1512
GO:0016874 ligase activity 0.349104 4 / 1151
GO:0003676 nucleic acid binding 0.921208 26 / 12641
GO:1901265 nucleoside phosphate... 0.011045 36 / 9421
GO:0043168 anion binding 0.009637 34 / 8687
GO:0016651 oxidoreductase activ... 0.435469 2 / 570
GO:0016730 oxidoreductase activ... 0.001359 2 / 21
GO:0016614 oxidoreductase activ... 0.330737 3 / 782
GO:0016830 carbon−carbon lyase ... 0.144867 3 / 506
GO:0016879 ligase activity, for... 0.012093 4 / 339
GO:0000166 nucleotide binding 0.011045 36 / 9421
GO:0003723 RNA binding 0.050919 14 / 3314
GO:0035639 purine ribonucleosid... 0.002677 30 / 6763
GO:0016652 oxidoreductase activ... 0.003151 2 / 32
GO:0016615 malate dehydrogenase... 0.000453 3 / 57
GO:0016616 oxidoreductase activ... 0.224058 3 / 629
GO:0016831 carboxy−lyase activi... 0.063852 3 / 351
GO:0016880 acid−ammonia (or ami... 0.004652 2 / 39
GO:0032553 ribonucleotide bindi... 0.003163 31 / 7145
GO:0003729 mRNA binding 0.000580 5 / 256
GO:0030060 L−malate dehydrogena... 0.004421 2 / 38
GO:0004611 phosphoenolpyruvate ... 0.002773 2 / 30
GO:0016211 ammonia ligase activ... 0.004652 2 / 39
GO:0004356 glutamate−ammonia li... 0.002249 2 / 27

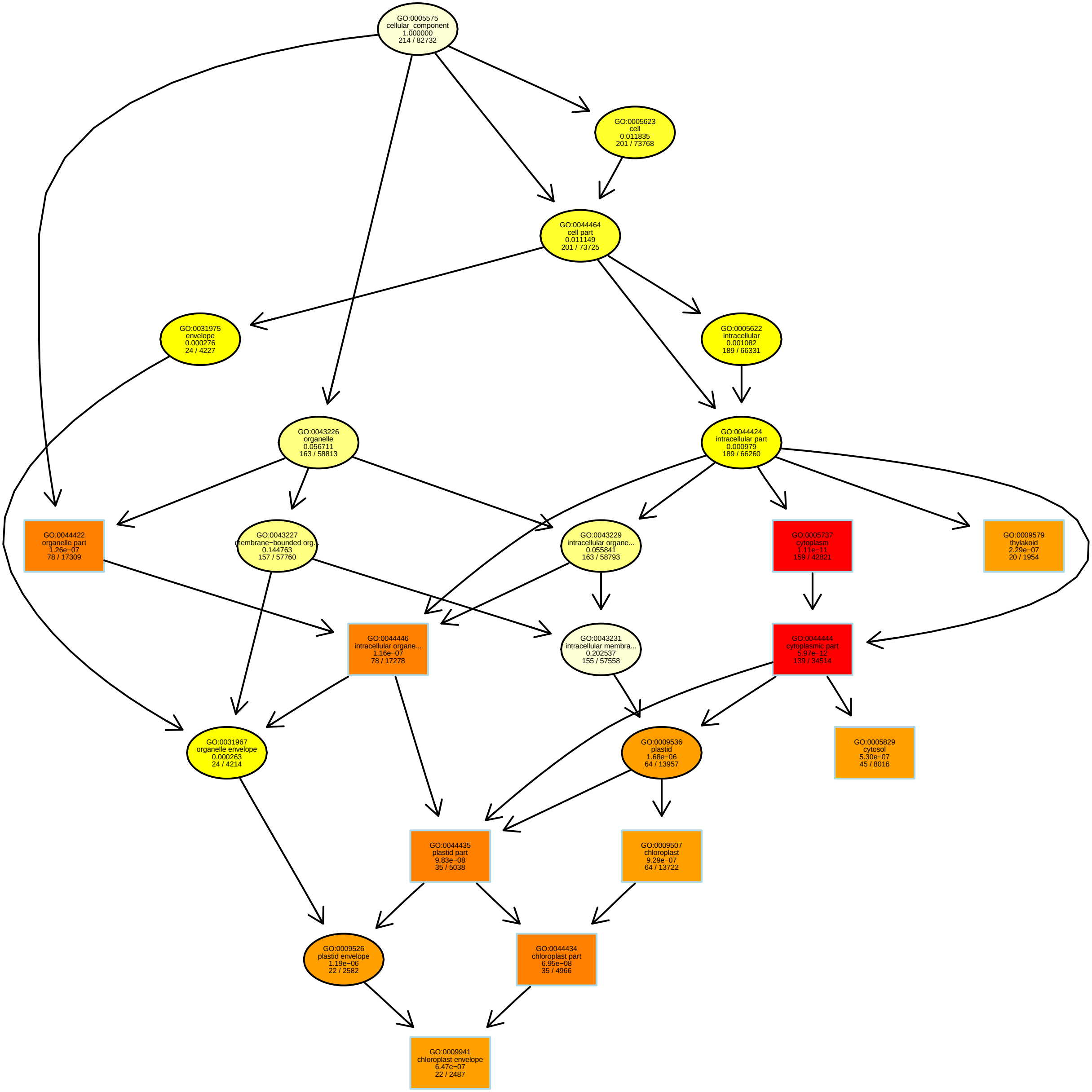
GO:0005575
cellular_component
1.000000
214 / 82732
GO:0005623
cell
0.011835
201 / 73768
GO:0044464
cell part
0.011149
201 / 73725
GO:0031975
envelope
0.000276
24 / 4227
GO:0005622
intracellular
0.001082
189 / 66331
GO:0043226
organelle
0.056711
163 / 58813
GO:0044424
intracellular part
0.000979
189 / 66260
GO:0044422
organelle part
1.26e−07
78 / 17309
GO:0043227
membrane−bounded org...
0.144763
157 / 57760
GO:0043229
intracellular organe...
0.055841
163 / 58793
GO:0005737
cytoplasm
1.11e−11
159 / 42821
GO:0009579
thylakoid
2.29e−07
20 / 1954
GO:0044446
intracellular organe...
1.16e−07
78 / 17278
GO:0043231
intracellular membra...
0.202537
155 / 57558
GO:0044444
cytoplasmic part
5.97e−12
139 / 34514
GO:0031967
organelle envelope
0.000263
24 / 4214
GO:0009536
plastid
1.68e−06
64 / 13957
GO:0005829
cytosol
5.30e−07
45 / 8016
GO:0044435
plastid part
9.83e−08
35 / 5038
GO:0009507
chloroplast
9.29e−07
64 / 13722
GO:0009526
plastid envelope
1.19e−06
22 / 2582
GO:0044434
chloroplast part
6.95e−08
35 / 4966
GO:0009941
chloroplast envelope
6.47e−07
22 / 2487
