# Supplementary material for: Differential Alternative Splicing Genes and Isoform Regulation Networks of Rapeseed (Brassica napus L.) Infected with Sclerotinia sclerotiorum
Source: Genes (Basel). 2020 Jul 13;11(7):784. doi: 10.3390/genes11070784 (PMC7397149; doi:10.3390/genes11070784)
Supplement: Supplementary file 1 [file genes-11-00784-s001.zip › Fig. S2 Enriched GO terms of genes in the 85 WGCNA modules.zip!/Fig. S2/CytoNodetan-go.pdf]

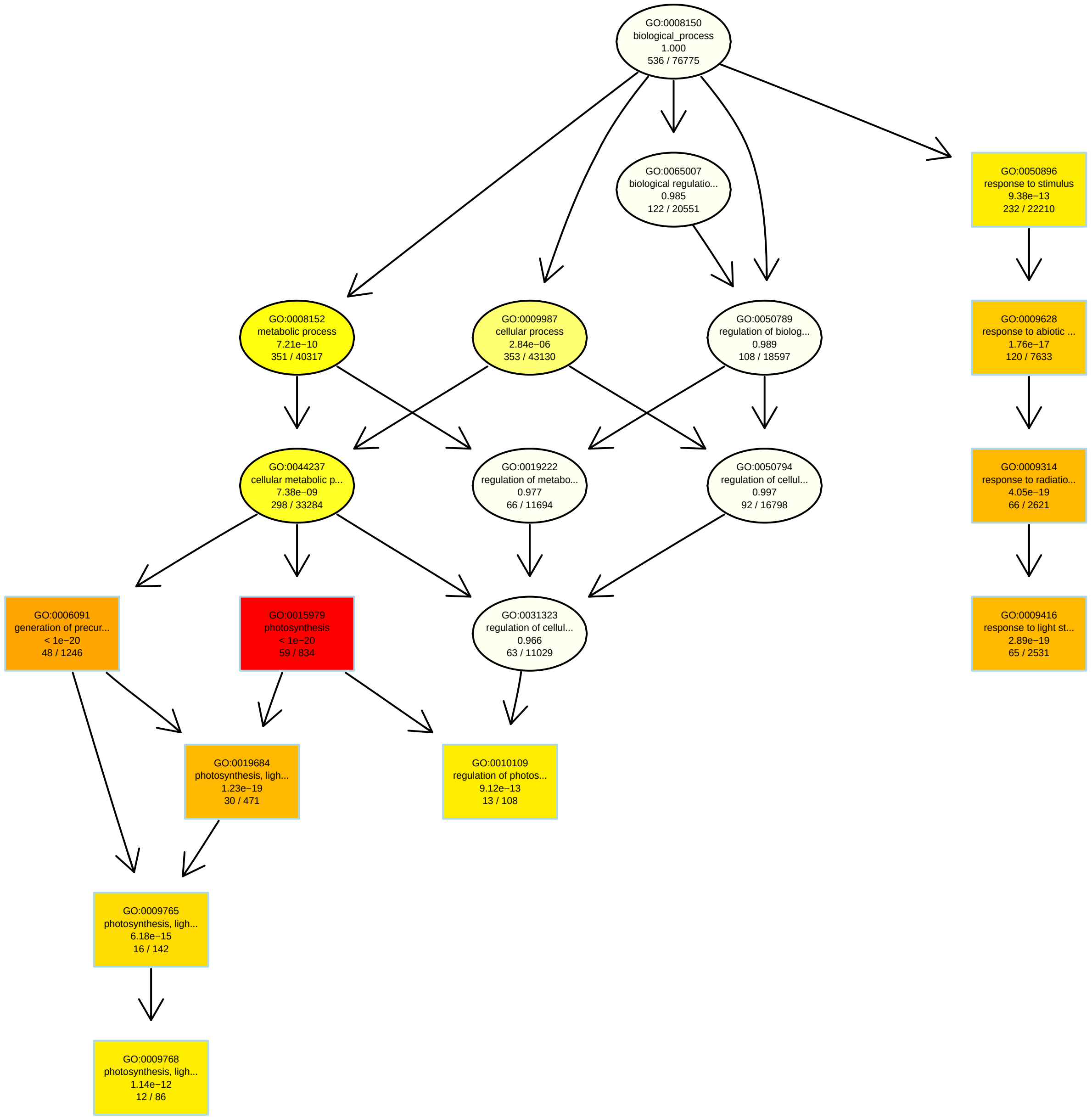

GO:0008150
biological_process
1.000
536 / 76775
GO:0065007
biological regulatio...
0.985
122 / 20551
GO:0050896
response to stimulus
9.38e−13
232 / 22210
GO:0008152
metabolic process
7.21e−10
351 / 40317
GO:0009987
cellular process
2.84e−06
353 / 43130
GO:0050789
regulation of biolog...
0.989
108 / 18597
GO:0009628
response to abiotic ...
1.76e−17
120 / 7633
GO:0044237
cellular metabolic p...
7.38e−09
298 / 33284
GO:0019222
regulation of metabo...
0.977
66 / 11694
GO:0050794
regulation of cellul...
0.997
92 / 16798
GO:0009314
response to radiatio...
4.05e−19
66 / 2621
GO:0006091
generation of precur...
< 1e−20
48 / 1246
GO:0015979
photosynthesis
< 1e−20
59 / 834
GO:0031323
regulation of cellul...
0.966
63 / 11029
GO:0009416
response to light st...
2.89e−19
65 / 2531
GO:0019684
photosynthesis, ligh...
1.23e−19
30 / 471
GO:0010109
regulation of photos...
9.12e−13
13 / 108
GO:0009765
photosynthesis, ligh...
6.18e−15
16 / 142
GO:0009768
photosynthesis, ligh...
1.14e−12
12 / 86

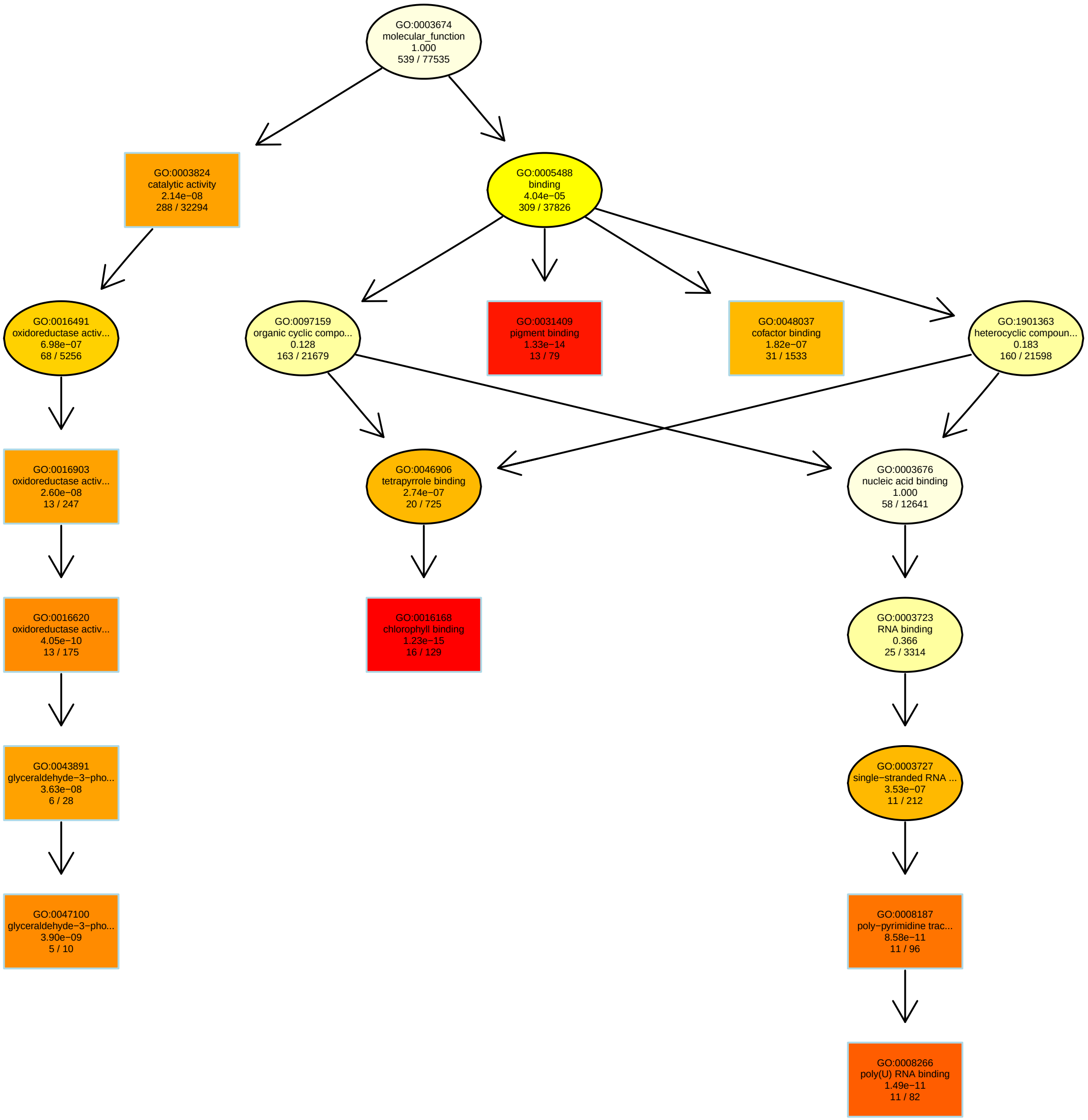
GO:0003674
molecular_function
1.000
539 / 77535
GO:0003824
catalytic activity
2.14e−08
288 / 32294
GO:0005488
binding
4.04e−05
309 / 37826
GO:0016491
oxidoreductase activ...
6.98e−07
68 / 5256
GO:0097159
organic cyclic compo...
0.128
163 / 21679
GO:0031409
pigment binding
1.33e−14
13 / 79
GO:0048037
cofactor binding
1.82e−07
31 / 1533
GO:1901363
heterocyclic compoun...
0.183
160 / 21598
GO:0016903
oxidoreductase activ...
2.60e−08
13 / 247
GO:0046906
tetrapyrrole binding
2.74e−07
20 / 725
GO:0003676
nucleic acid binding
1.000
58 / 12641
GO:0016620
oxidoreductase activ...
4.05e−10
13 / 175
GO:0016168
chlorophyll binding
1.23e−15
16 / 129
GO:0003723
RNA binding
0.366
25 / 3314
GO:0043891
glyceraldehyde−3−pho...
3.63e−08
6 / 28
GO:0003727
single−stranded RNA ...
3.53e−07
11 / 212
GO:0047100
glyceraldehyde−3−pho...
3.90e−09
5 / 10
GO:0008187
poly−pyrimidine trac...
8.58e−11
11 / 96
GO:0008266
poly(U) RNA binding
1.49e−11
11 / 82

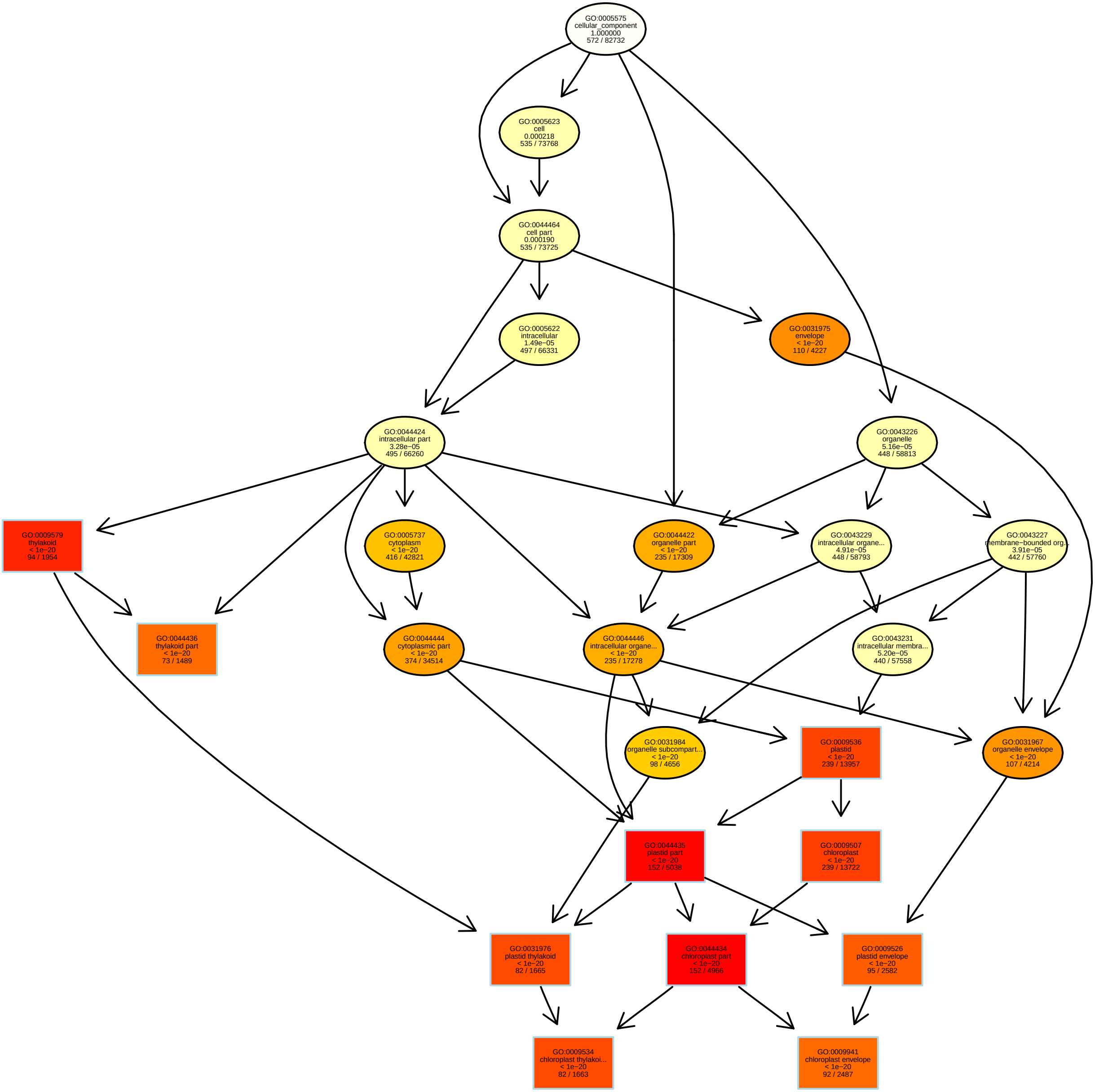
GO:0005575
cellular_component
1.000000
572 / 82732
GO:0005623
cell
0.000218
535 / 73768
GO:0044464
cell part
0.000190
535 / 73725
GO:0005622
intracellular
1.49e−05
497 / 66331
GO:0031975
envelope
< 1e−20
110 / 4227
GO:0044424
intracellular part
3.28e−05
495 / 66260
GO:0043226
organelle
5.16e−05
448 / 58813
GO:0009579
thylakoid
< 1e−20
94 / 1954
GO:0005737
cytoplasm
< 1e−20
416 / 42821
GO:0044422
organelle part
< 1e−20
235 / 17309
GO:0043229
intracellular organe...
4.91e−05
448 / 58793
GO:0043227
membrane−bounded org...
3.91e−05
442 / 57760
GO:0044436
thylakoid part
< 1e−20
73 / 1489
GO:0044444
cytoplasmic part
< 1e−20
374 / 34514
GO:0044446
intracellular organe...
< 1e−20
235 / 17278
GO:0043231
intracellular membra...
5.20e−05
440 / 57558
GO:0031984
organelle subcompart...
< 1e−20
98 / 4656
GO:0009536
plastid
< 1e−20
239 / 13957
GO:0031967
organelle envelope
< 1e−20
107 / 4214
GO:0044435
plastid part
< 1e−20
152 / 5038
GO:0009507
chloroplast
< 1e−20
239 / 13722
GO:0031976
plastid thylakoid
< 1e−20
82 / 1665
GO:0044434
chloroplast part
< 1e−20
152 / 4966
GO:0009526
plastid envelope
< 1e−20
95 / 2582
GO:0009534
chloroplast thylakoi...
< 1e−20
82 / 1663
GO:0009941
chloroplast envelope
< 1e−20
92 / 2487
