# Supplementary material for: Differential Alternative Splicing Genes and Isoform Regulation Networks of Rapeseed (Brassica napus L.) Infected with Sclerotinia sclerotiorum
Source: Genes (Basel). 2020 Jul 13;11(7):784. doi: 10.3390/genes11070784 (PMC7397149; doi:10.3390/genes11070784)
Supplement: Supplementary file 1 [file genes-11-00784-s001.zip › Fig. S2 Enriched GO terms of genes in the 85 WGCNA modules.zip!/Fig. S2/CytoNodethistle-go.pdf]

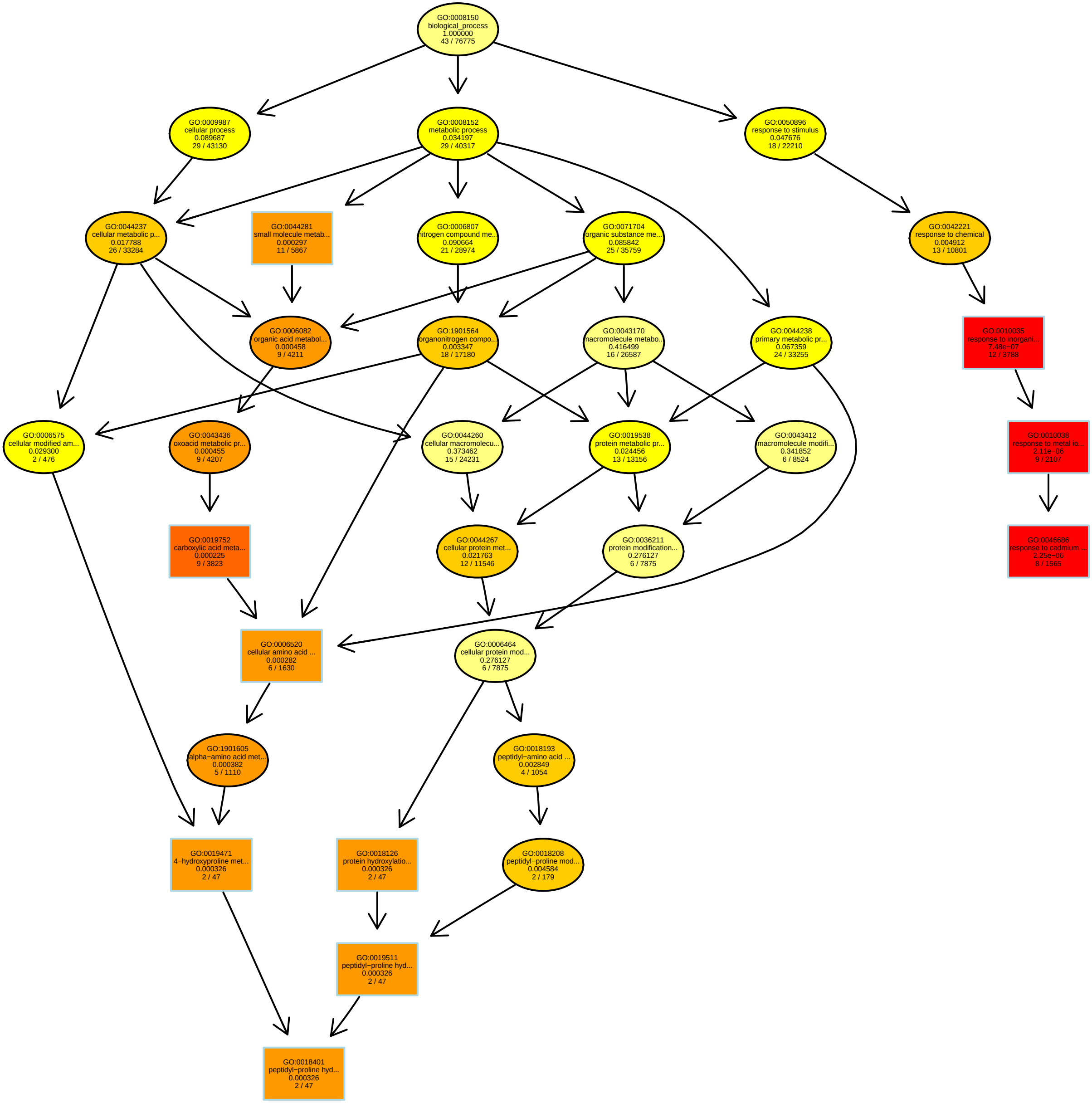
GO:0008150
biological_process
1.000000
43 / 76775
GO:0009987
cellular process
0.089687
29 / 43130
GO:0008152
metabolic process
0.034197
29 / 40317
GO:0050896
response to stimulus
0.047676
18 / 22210
GO:0044237
cellular metabolic p...
0.017788
26 / 33284
GO:0044281
small molecule metab...
0.000297
11 / 5867
GO:0006807
nitrogen compound me...
0.090664
21 / 28974
GO:0071704
organic substance me...
0.085842
25 / 35759
GO:0042221
response to chemical
0.004912
13 / 10801
GO:0006082
organic acid metabol...
0.000458
9 / 4211
GO:1901564
organonitrogen compo...
0.003347
18 / 17180
GO:0043170
macromolecule metabo...
0.416499
16 / 26587
GO:0044238
primary metabolic pr...
0.067359
24 / 33255
GO:0010035
response to inorgani...
7.48e−07
12 / 3788
GO:0006575
cellular modified am...
0.029300
2 / 476
GO:0043436
oxoacid metabolic pr...
0.000455
9 / 4207
GO:0044260
cellular macromolecu...
0.373462
15 / 24231
GO:0019538
protein metabolic pr...
0.024456
13 / 13156
GO:0043412
macromolecule modifi...
0.341852
6 / 8524
GO:0010038
response to metal io...
2.11e−06
9 / 2107
GO:0019752
carboxylic acid meta...
0.000225
9 / 3823
GO:0044267
cellular protein met...
0.021763
12 / 11546
GO:0036211
protein modification...
0.276127
6 / 7875
GO:0046686
response to cadmium ...
2.25e−06
8 / 1565
GO:0006520
cellular amino acid ...
0.000282
6 / 1630
GO:0006464
cellular protein mod...
0.276127
6 / 7875
GO:1901605
alpha−amino acid met...
0.000382
5 / 1110
GO:0018193
peptidyl−amino acid ...
0.002849
4 / 1054
GO:0019471
4−hydroxyproline met...
0.000326
2 / 47
GO:0018126
protein hydroxylatio...
0.000326
2 / 47
GO:0018208
peptidyl−proline mod...
0.004584
2 / 179
GO:0019511
peptidyl−proline hyd...
0.000326
2 / 47
GO:0018401
peptidyl−proline hyd...
0.000326
2 / 47

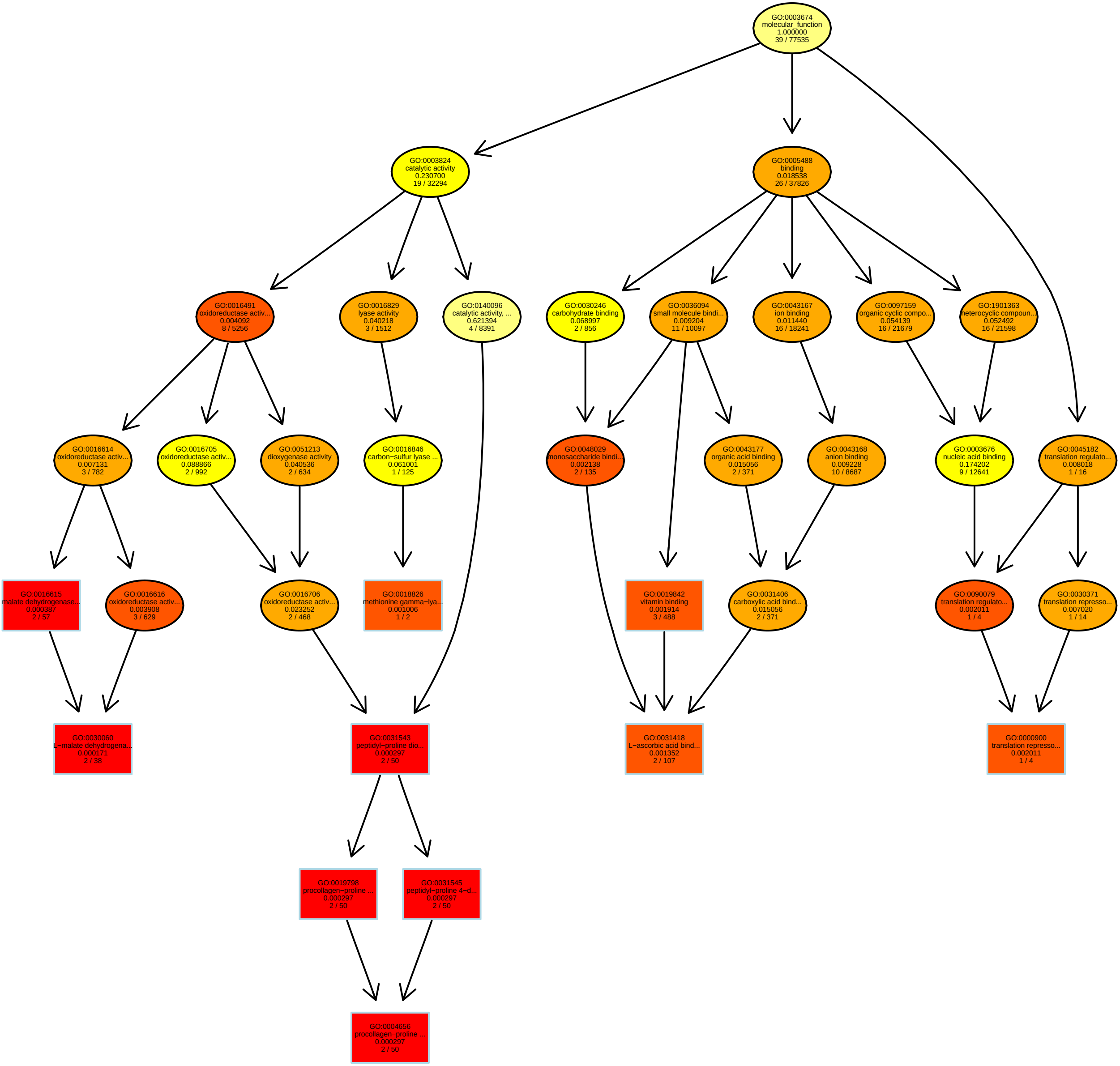
GO:0003674
molecular_function
1.000000
39 / 77535
GO:0003824
catalytic activity
0.230700
19 / 32294
GO:0005488
binding
0.018538
26 / 37826
GO:0016491
oxidoreductase activ...
0.004092
8 / 5256
GO:0016829
lyase activity
0.040218
3 / 1512
GO:0140096
catalytic activity, ...
0.621394
4 / 8391
GO:0030246
carbohydrate binding
0.068997
2 / 856
GO:0036094
small molecule bindi...
0.009204
11 / 10097
GO:0043167
ion binding
0.011440
16 / 18241
GO:0097159
organic cyclic compo...
0.054139
16 / 21679
GO:1901363
heterocyclic compoun...
0.052492
16 / 21598
GO:0016614
oxidoreductase activ...
0.007131
3 / 782
GO:0016705
oxidoreductase activ...
0.088866
2 / 992
GO:0051213
dioxygenase activity
0.040536
2 / 634
GO:0016846
carbon−sulfur lyase ...
0.061001
1 / 125
GO:0048029
monosaccharide bindi...
0.002138
2 / 135
GO:0043177
organic acid binding
0.015056
2 / 371
GO:0043168
anion binding
0.009228
10 / 8687
GO:0003676
nucleic acid binding
0.174202
9 / 12641
GO:0045182
translation regulato...
0.008018
1 / 16
GO:0016615
malate dehydrogenase ...
0.000387
2 / 57
GO:0016616
oxidoreductase activ...
0.003908
3 / 629
GO:0016706
oxidoreductase activ...
0.023252
2 / 468
GO:0018826
methionine gamma−lya...
0.001006
1 / 2
GO:0019842
vitamin binding
0.001914
3 / 488
GO:0031406
carboxylic acid bind...
0.015056
2 / 371
GO:0090079
translation regulato...
0.002011
1 / 4
GO:0030371
translation represso...
0.007020
1 / 14
GO:0030060
L−malate dehydrogena...
0.000171
2 / 38
GO:0031543
peptidyl−proline dio...
0.000297
2 / 50
GO:0031418
L−ascorbic acid bind...
0.001352
2 / 107
GO:0000900
translation represso...
0.002011
1 / 4
GO:0019798
procollagen−proline ...
0.000297
2 / 50
GO:0031545
peptidyl−proline 4−d...
0.000297
2 / 50
GO:0004656
procollagen−proline ...
0.000297
2 / 50

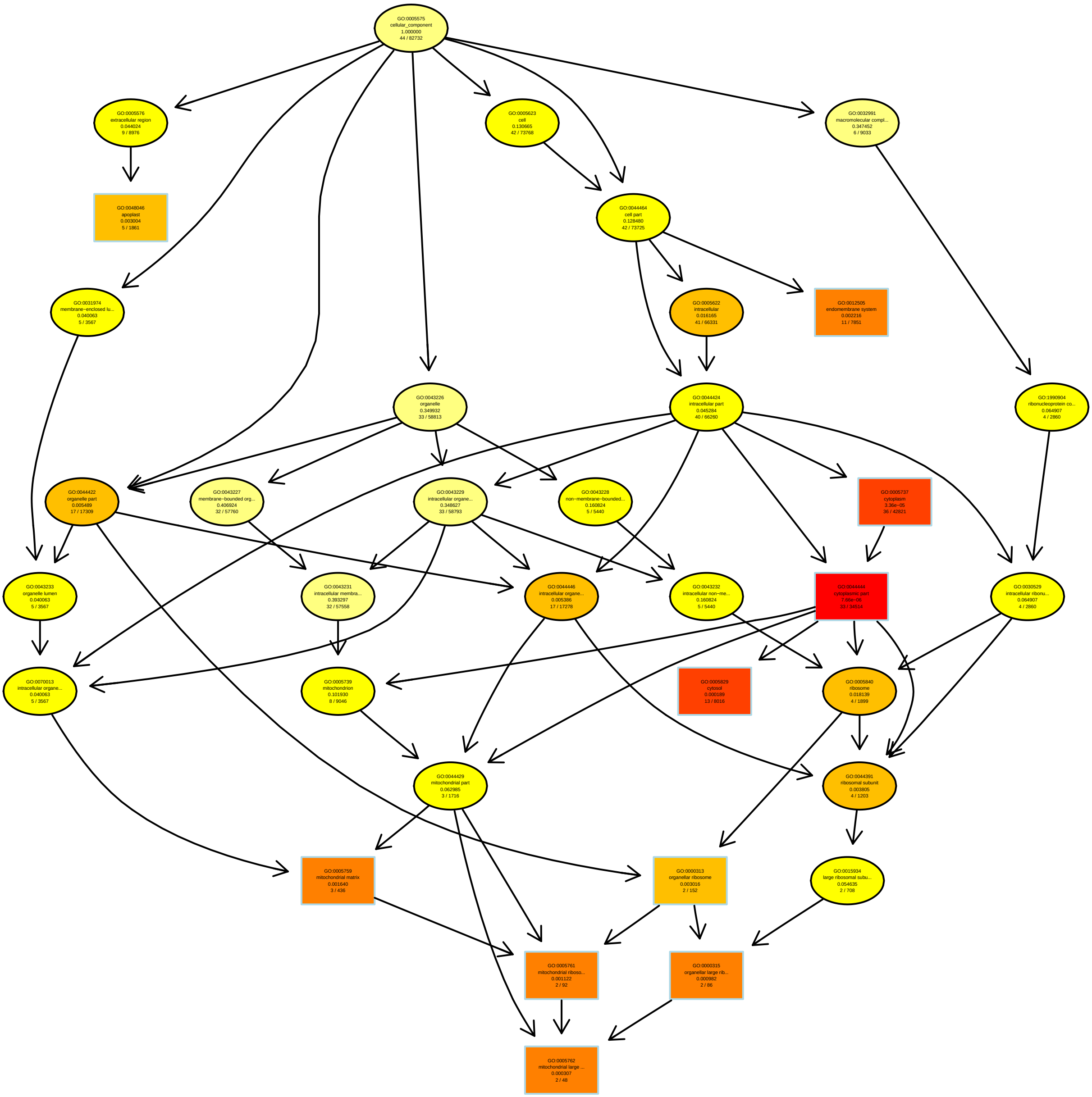
GO:0005575 cellular_component 1.000000 44 / 82732
GO:0005576 extracellular region 0.044024 9 / 8976
GO:0005623 cell 0.130665 42 / 73768
GO:0032991 macromolecular compl... 0.347452 6 / 9033
GO:0048046 apoplast 0.003004 5 / 1861
GO:0044464 cell part 0.128480 42 / 73725
GO:0031974 membrane−enclosed lu... 0.040063 5 / 3567
GO:0005622 intracellular 0.016165 41 / 66331
GO:0012505 endomembrane system 0.002216 11 / 7851
GO:0043226 organelle 0.349932 33 / 58813
GO:0044424 intracellular part 0.045284 40 / 66260
GO:1990904 ribonucleoprotein co... 0.064907 4 / 2860
GO:0044422 organelle part 0.005489 17 / 17309
GO:0043227 membrane−bounded org... 0.406924 32 / 57760
GO:0043229 intracellular organe... 0.348627 33 / 58793
GO:0043228 non−membrane−bounded... 0.160824 5 / 5440
GO:0005737 cytoplasm 3.36e−05 36 / 42821
GO:0043233 organelle lumen 0.040063 5 / 3567
GO:0043231 intracellular membra... 0.393297 32 / 57558
GO:0044446 intracellular organe... 0.005386 17 / 17278
GO:0043232 intracellular non−me... 0.160824 5 / 5440
GO:0044444 cytoplasmic part 7.66e−06 33 / 34514
GO:0030529 intracellular ribonu... 0.064907 4 / 2860
GO:0070013 intracellular organe... 0.040063 5 / 3567
GO:0005739 mitochondrion 0.101930 8 / 9046
GO:0005829 cytosol 0.000189 13 / 8016
GO:0005840 ribosome 0.018139 4 / 1899
GO:0044429 mitochondrial part 0.062985 3 / 1716
GO:0044391 ribosomal subunit 0.003805 4 / 1203
GO:0005759 mitochondrial matrix 0.001640 3 / 436
GO:0000313 organellar ribosome 0.003016 2 / 152
GO:0015934 large ribosomal subu... 0.054635 2 / 708
GO:0005761 mitochondrial riboso... 0.001122 2 / 92
GO:0000315 organellar large rib... 0.000982 2 / 86
GO:0005762 mitochondrial large ... 0.000307 2 / 48
