# Supplementary material for: Differential Alternative Splicing Genes and Isoform Regulation Networks of Rapeseed (Brassica napus L.) Infected with Sclerotinia sclerotiorum
Source: Genes (Basel). 2020 Jul 13;11(7):784. doi: 10.3390/genes11070784 (PMC7397149; doi:10.3390/genes11070784)
Supplement: Supplementary file 1 [file genes-11-00784-s001.zip › Fig. S2 Enriched GO terms of genes in the 85 WGCNA modules.zip!/Fig. S2/CytoNodethistle1-go.pdf]

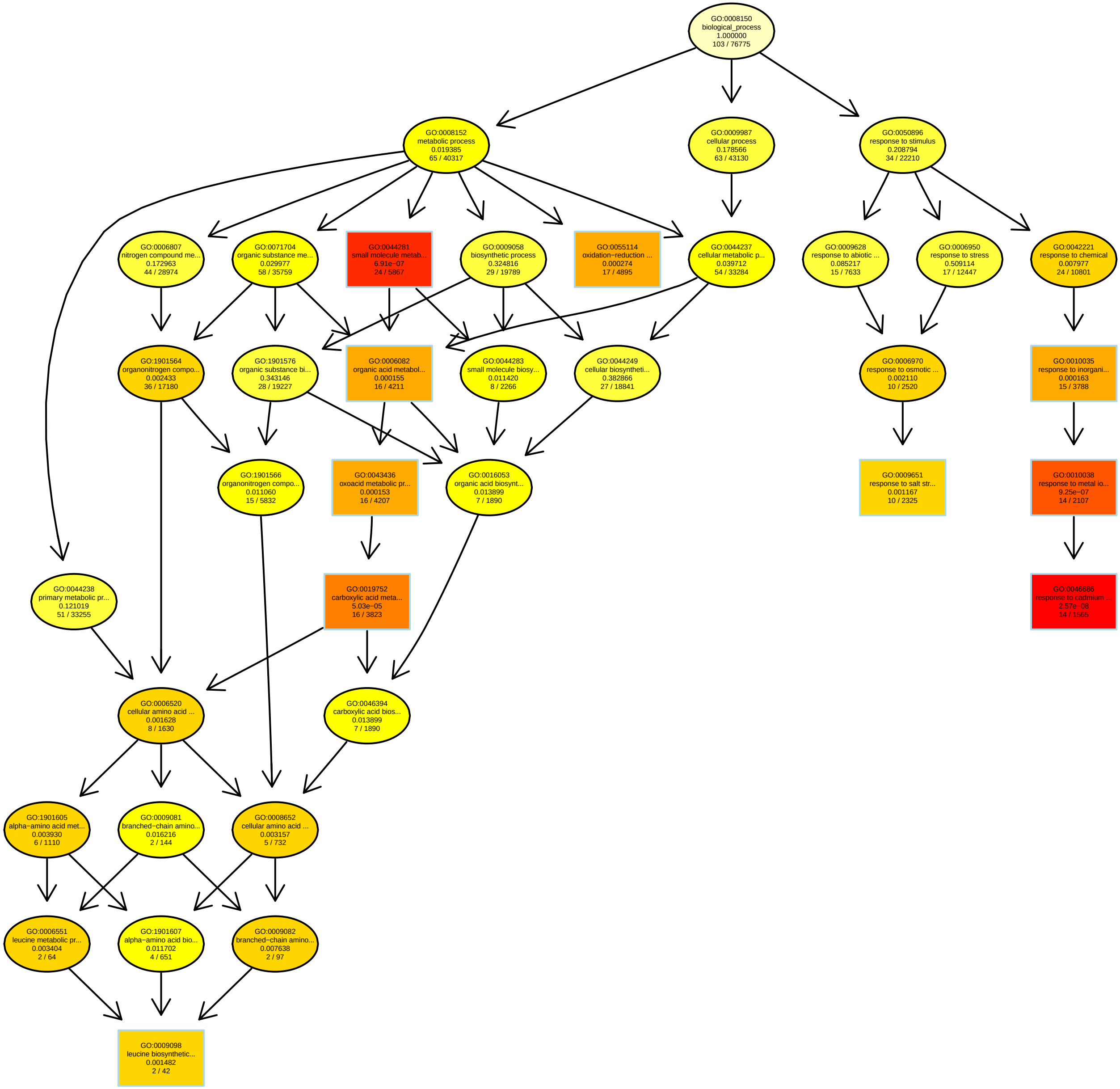
GO:0008150
biological_process
1.000000
103 / 76775
GO:0008152
metabolic process
0.019385
65 / 40317
GO:0009987
cellular process
0.178566
63 / 43130
GO:0050896
response to stimulus
0.208794
34 / 22210
GO:0006807
nitrogen compound me...
0.172963
44 / 28974
GO:0071704
organic substance me...
0.029977
58 / 35759
GO:0044281
small molecule metab...
6.91e−07
24 / 5867
GO:0009058
biosynthetic process
0.324816
29 / 19789
GO:0055114
oxidation−reduction ...
0.000274
17 / 4895
GO:0044237
cellular metabolic p...
0.039712
54 / 33284
GO:0009628
response to abiotic ...
0.085217
15 / 7633
GO:0006950
response to stress
0.509114
17 / 12447
GO:0042221
response to chemical
0.007977
24 / 10801
GO:1901564
organonitrogen compo...
0.002433
36 / 17180
GO:1901576
organic substance bi...
0.343146
28 / 19227
GO:0006082
organic acid metabol...
0.000155
16 / 4211
GO:0044283
small molecule biosy...
0.011420
8 / 2266
GO:0044249
cellular biosyntheti...
0.382866
27 / 18841
GO:0006970
response to osmotic ...
0.002110
10 / 2520
GO:0010035
response to inorgani...
0.000163
15 / 3788
GO:1901566
organonitrogen compo...
0.011060
15 / 5832
GO:0043436
oxoacid metabolic pr...
0.000153
16 / 4207
GO:0016053
organic acid biosynt...
0.013899
7 / 1890
GO:0009651
response to salt str...
0.001167
10 / 2325
GO:0010038
response to metal io...
9.25e−07
14 / 2107
GO:0044238
primary metabolic pr...
0.121019
51 / 33255
GO:0019752
carboxylic acid meta...
5.03e−05
16 / 3823
GO:0046686
response to cadmium ...
2.57e−08
14 / 1565
GO:0006520
cellular amino acid ...
0.001628
8 / 1630
GO:0046394
carboxylic acid bios...
0.013899
7 / 1890
GO:1901605
alpha−amino acid met...
0.003930
6 / 1110
GO:0009081
branched−chain amino...
0.016216
2 / 144
GO:0008652
cellular amino acid ...
0.003157
5 / 732
GO:0006551
leucine metabolic pr...
0.003404
2 / 64
GO:1901607
alpha−amino acid bio...
0.011702
4 / 651
GO:0009082
branched−chain amino...
0.007638
2 / 97
GO:0009098
leucine biosynthetic...
0.001482
2 / 42

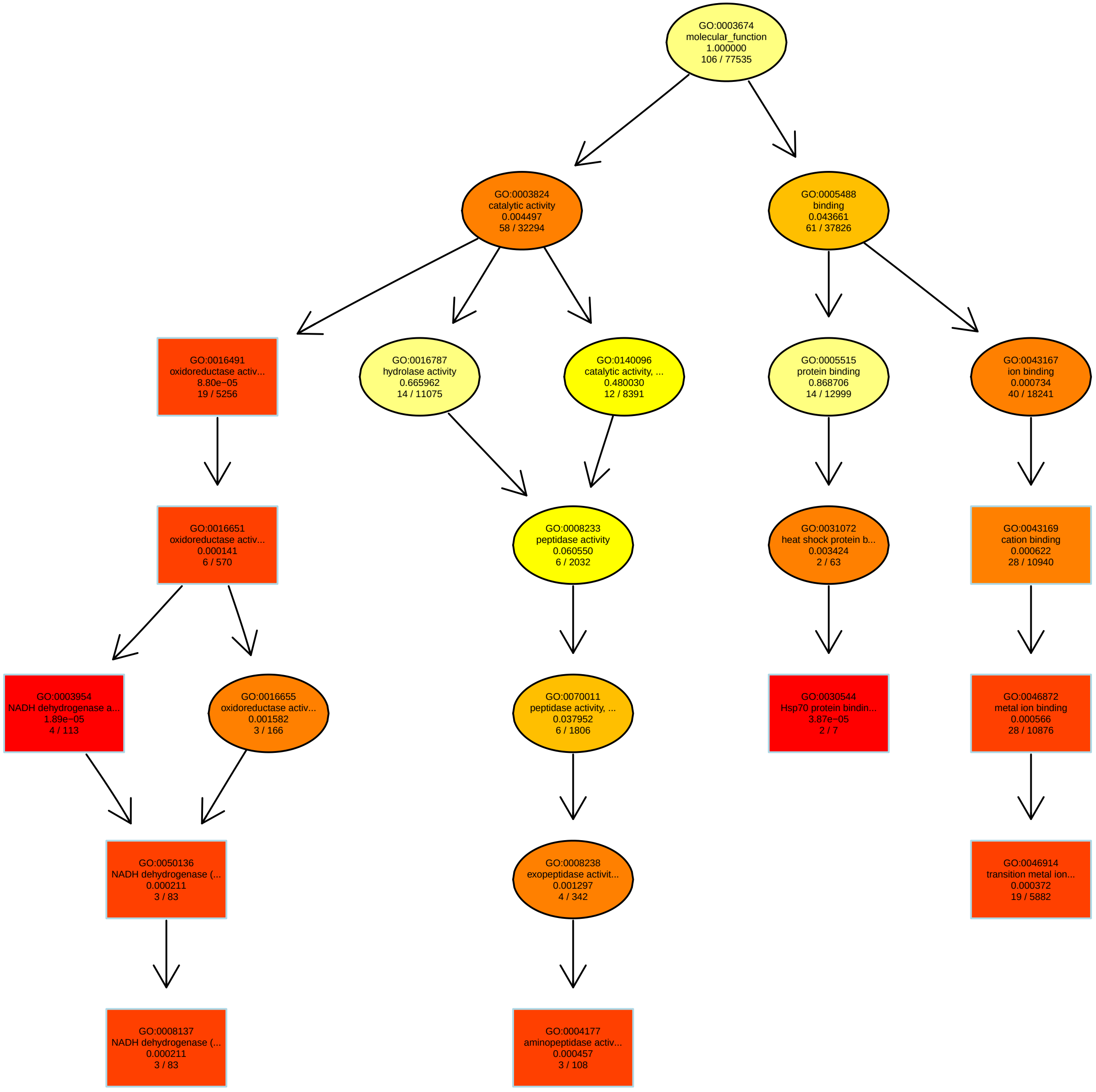

GO:0003674
molecular_function
1.000000
106 / 77535
GO:0003824
catalytic activity
0.004497
58 / 32294
GO:0005488
binding
0.043661
61 / 37826
GO:0016491
oxidoreductase activ...
8.80e−05
19 / 5256
GO:0016787
hydrolase activity
0.665962
14 / 11075
GO:0140096
catalytic activity, ...
0.480030
12 / 8391
GO:0005515
protein binding
0.868706
14 / 12999
GO:0043167
ion binding
0.000734
40 / 18241
GO:0016651
oxidoreductase activ...
0.000141
6 / 570
GO:0008233
peptidase activity
0.060550
6 / 2032
GO:0031072
heat shock protein b...
0.003424
2 / 63
GO:0043169
cation binding
0.000622
28 / 10940
GO:0003954
NADH dehydrogenase a...
1.89e−05
4 / 113
GO:0016655
oxidoreductase activ...
0.001582
3 / 166
GO:0070011
peptidase activity, ...
0.037952
6 / 1806
GO:0030544
Hsp70 protein bindin...
3.87e−05
2 / 7
GO:0046872
metal ion binding
0.000566
28 / 10876
GO:0050136
NADH dehydrogenase (...
0.000211
3 / 83
GO:0008238
exopeptidase activit...
0.001297
4 / 342
GO:0046914
transition metal ion...
0.000372
19 / 5882
GO:0008137
NADH dehydrogenase (...
0.000211
3 / 83
GO:0004177
aminopeptidase activ...
0.000457
3 / 108

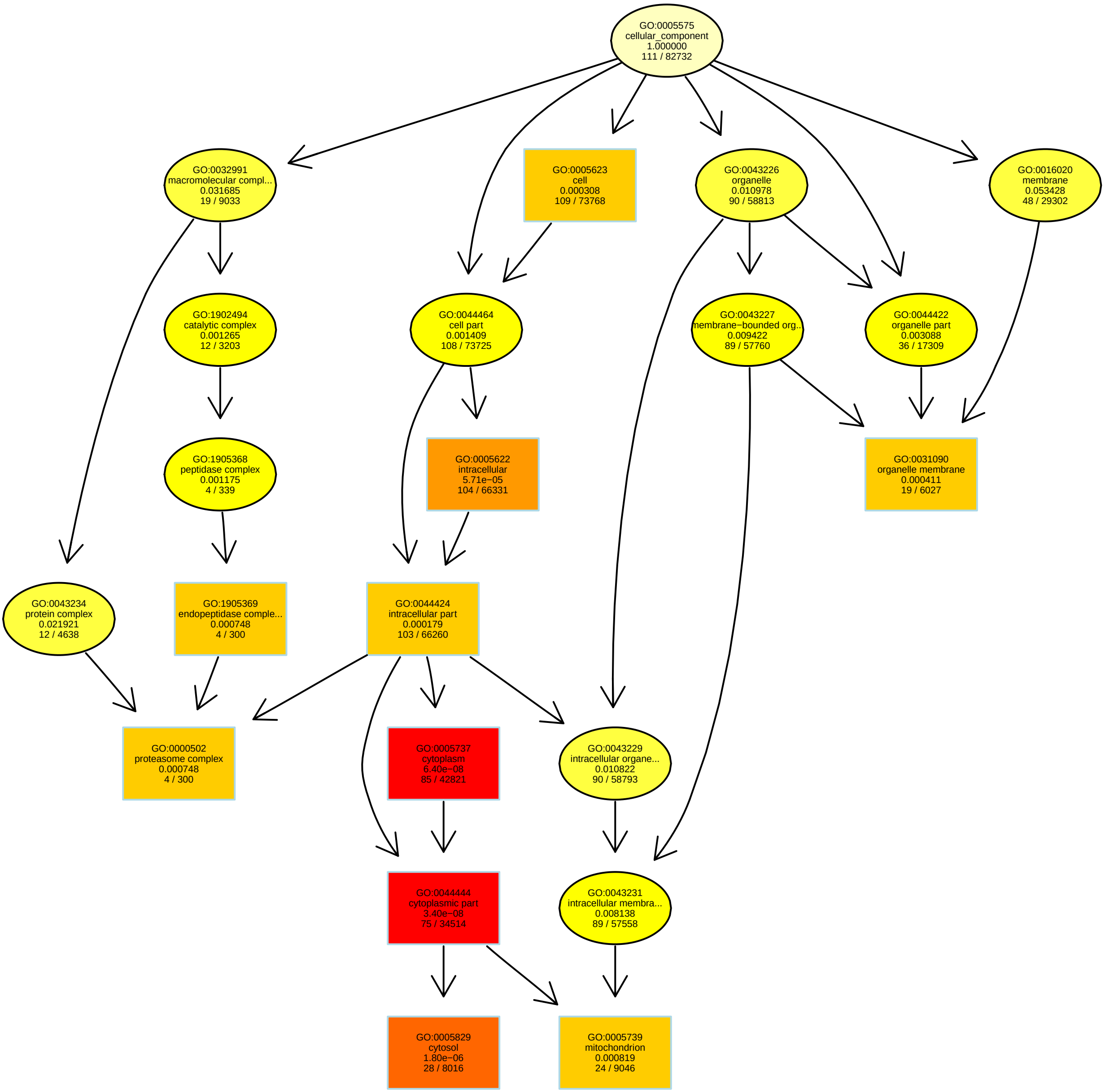
GO:0005575
cellular_component
1.000000
111 / 82732
GO:0032991
macromolecular compl...
0.031685
19 / 9033
GO:0005623
cell
0.000308
109 / 73768
GO:0043226
organelle
0.010978
90 / 58813
GO:0016020
membrane
0.053428
48 / 29302
GO:1902494
catalytic complex
0.001265
12 / 3203
GO:0044464
cell part
0.001409
108 / 73725
GO:0043227
membrane−bounded org...
0.009422
89 / 57760
GO:0044422
organelle part
0.003088
36 / 17309
GO:1905368
peptidase complex
0.001175
4 / 339
GO:0005622
intracellular
5.71e−05
104 / 66331
GO:0031090
organelle membrane
0.000411
19 / 6027
GO:0043234
protein complex
0.021921
12 / 4638
GO:1905369
endopeptidase comple...
0.000748
4 / 300
GO:0044424
intracellular part
0.000179
103 / 66260
GO:0000502
proteasome complex
0.000748
4 / 300
GO:0005737
cytoplasm
6.40e−08
85 / 42821
GO:0043229
intracellular organe...
0.010822
90 / 58793
GO:0044444
cytoplasmic part
3.40e−08
75 / 34514
GO:0043231
intracellular membra...
0.008138
89 / 57558
GO:0005829
cytosol
1.80e−06
28 / 8016
GO:0005739
mitochondrion
0.000819
24 / 9046
