# Supplementary material for: Differential Alternative Splicing Genes and Isoform Regulation Networks of Rapeseed (Brassica napus L.) Infected with Sclerotinia sclerotiorum
Source: Genes (Basel). 2020 Jul 13;11(7):784. doi: 10.3390/genes11070784 (PMC7397149; doi:10.3390/genes11070784)
Supplement: Supplementary file 1 [file genes-11-00784-s001.zip › Fig. S2 Enriched GO terms of genes in the 85 WGCNA modules.zip!/Fig. S2/CytoNodethistle2-go.pdf]

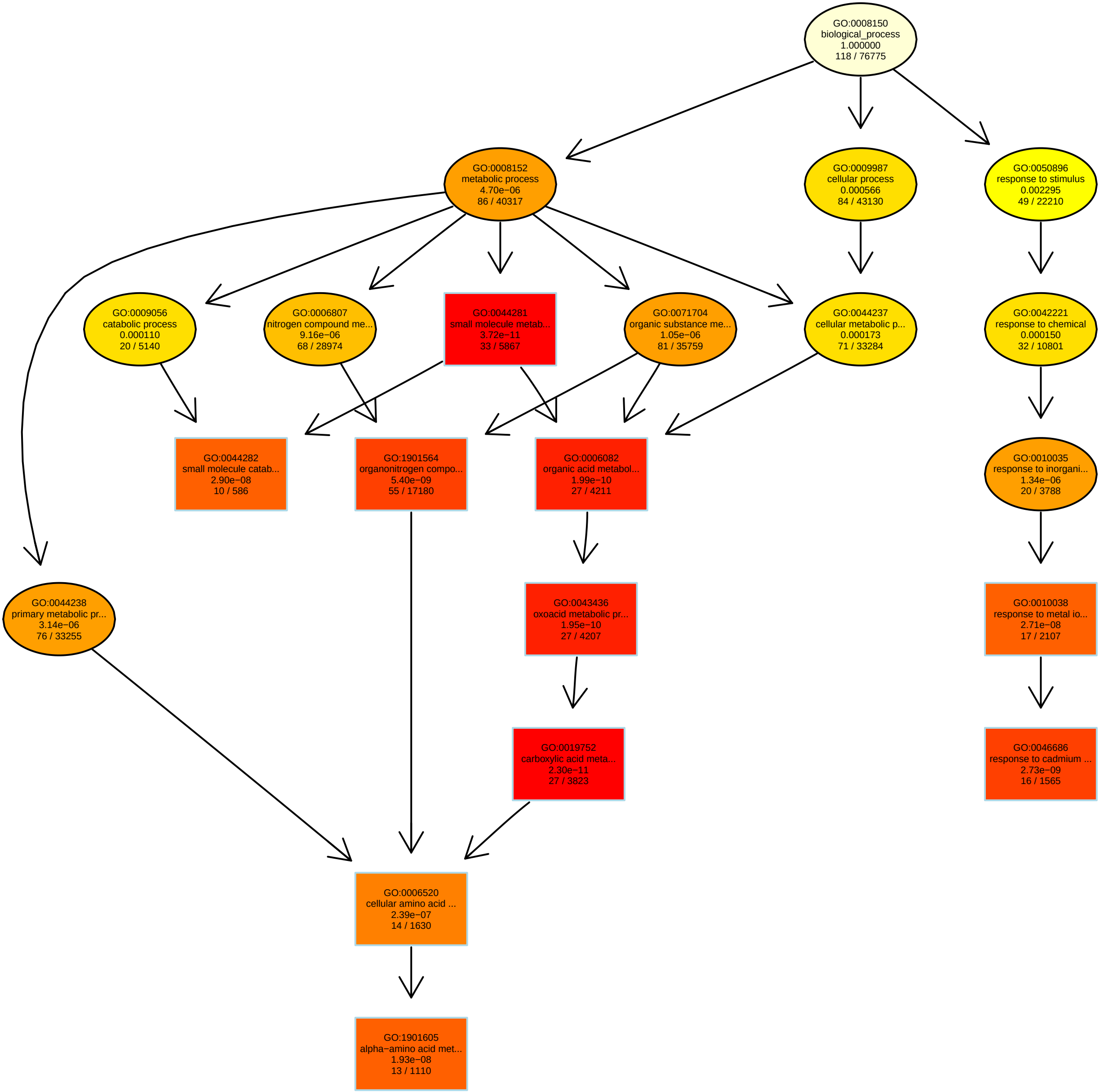

GO:0008150
biological_process
1.000000
118 / 76775
GO:0008152
metabolic process
4.70e−06
86 / 40317
GO:0009987
cellular process
0.000566
84 / 43130
GO:0050896
response to stimulus
0.002295
49 / 22210
GO:0009056
catabolic process
0.000110
20 / 5140
GO:0006807
nitrogen compound me...
9.16e−06
68 / 28974
GO:0044281
small molecule metab...
3.72e−11
33 / 5867
GO:0071704
organic substance me...
1.05e−06
81 / 35759
GO:0044237
cellular metabolic p...
0.000173
71 / 33284
GO:0042221
response to chemical
0.000150
32 / 10801
GO:0044282
small molecule catab...
2.90e−08
10 / 586
GO:1901564
organonitrogen compo...
5.40e−09
55 / 17180
GO:0006082
organic acid metabol...
1.99e−10
27 / 4211
GO:0010035
response to inorgani...
1.34e−06
20 / 3788
GO:0044238
primary metabolic pr...
3.14e−06
76 / 33255
GO:0043436
oxoacid metabolic pr...
1.95e−10
27 / 4207
GO:0010038
response to metal io...
2.71e−08
17 / 2107
GO:0019752
carboxylic acid meta...
2.30e−11
27 / 3823
GO:0046686
response to cadmium ...
2.73e−09
16 / 1565
GO:0006520
cellular amino acid ...
2.39e−07
14 / 1630
GO:1901605
alpha−amino acid met...
1.93e−08
13 / 1110

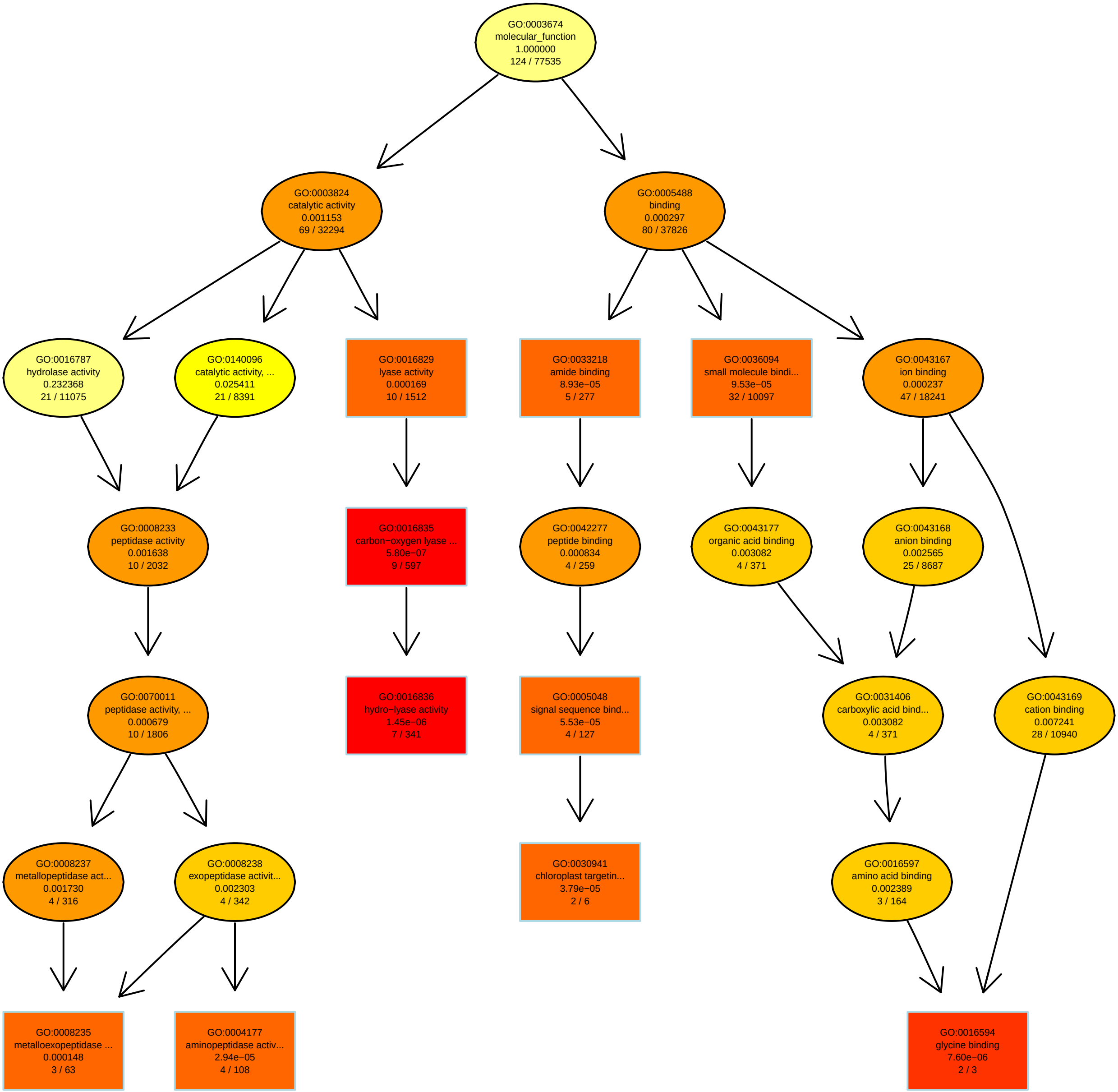
GO:0003674
molecular_function
1.000000
124 / 77535
GO:0003824
catalytic activity
0.001153
69 / 32294
GO:0005488
binding
0.000297
80 / 37826
GO:0016787
hydrolase activity
0.232368
21 / 11075
GO:0140096
catalytic activity, ...
0.025411
21 / 8391
GO:0016829
lyase activity
0.000169
10 / 1512
GO:0033218
amide binding
8.93e−05
5 / 277
GO:0036094
small molecule bindi...
9.53e−05
32 / 10097
GO:0043167
ion binding
0.000237
47 / 18241
GO:0008233
peptidase activity
0.001638
10 / 2032
GO:0016835
carbon−oxygen lyase ...
5.80e−07
9 / 597
GO:0042277
peptide binding
0.000834
4 / 259
GO:0043177
organic acid binding
0.003082
4 / 371
GO:0043168
anion binding
0.002565
25 / 8687
GO:0070011
peptidase activity, ...
0.000679
10 / 1806
GO:0016836
hydro−lyase activity
1.45e−06
7 / 341
GO:0005048
signal sequence bind...
5.53e−05
4 / 127
GO:0031406
carboxylic acid bind...
0.003082
4 / 371
GO:0043169
cation binding
0.007241
28 / 10940
GO:0008237
metallopeptidase act...
0.001730
4 / 316
GO:0008238
exopeptidase activit...
0.002303
4 / 342
GO:0030941
chloroplast targetin...
3.79e−05
2 / 6
GO:0016597
amino acid binding
0.002389
3 / 164
GO:0008235
metalloexopeptidase ...
0.000148
3 / 63
GO:0004177
aminopeptidase activ...
2.94e−05
4 / 108
GO:0016594
glycine binding
7.60e−06
2 / 3

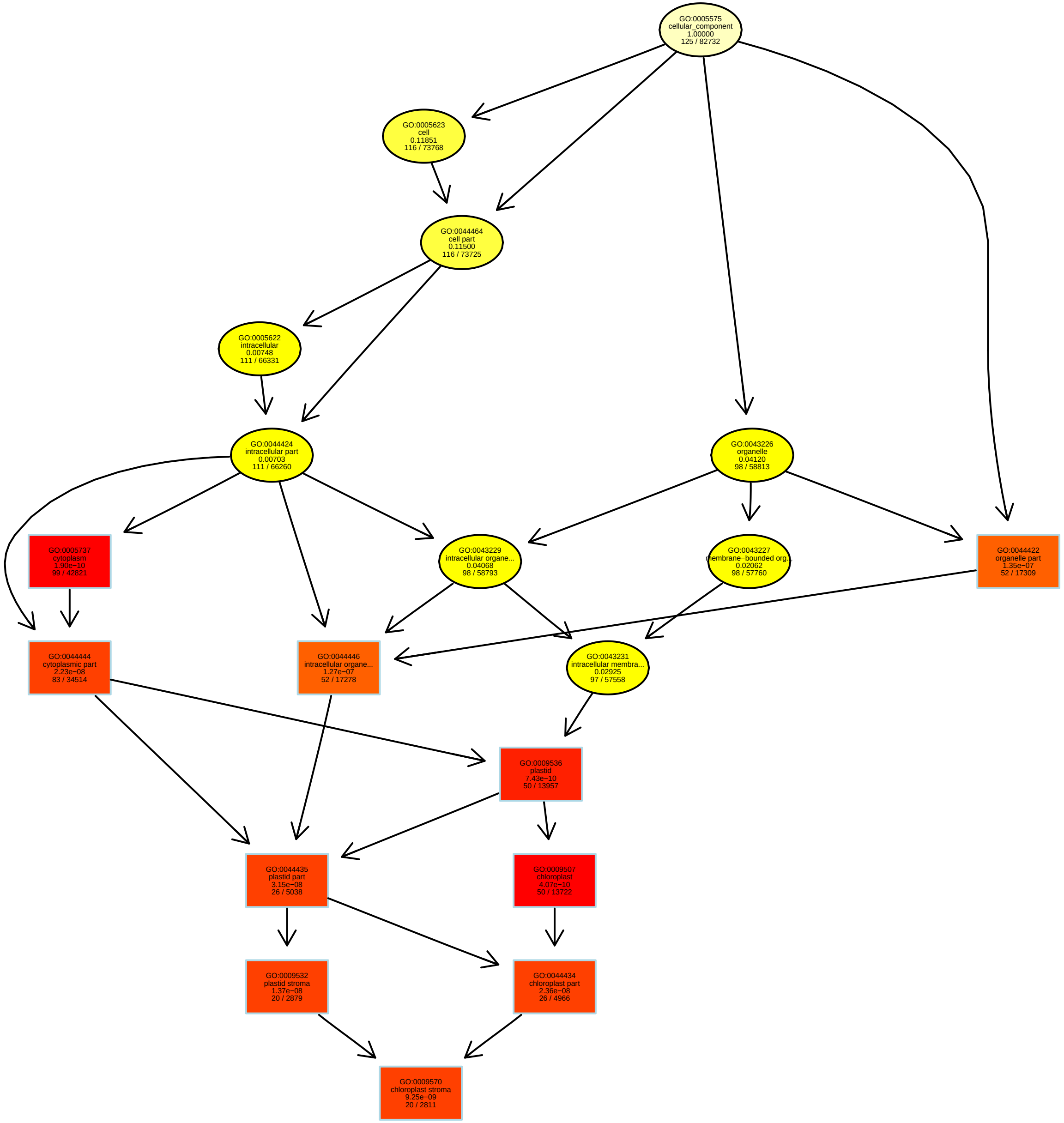
GO:0005575
cellular_component
1.00000
125 / 82732
GO:0005623
cell
0.11851
116 / 73768
GO:0044464
cell part
0.11500
116 / 73725
GO:0005622
intracellular
0.00748
111 / 66331
GO:0044424
intracellular part
0.00703
111 / 66260
GO:0043226
organelle
0.04120
98 / 58813
GO:0005737
cytoplasm
1.90e−10
99 / 42821
GO:0043229
intracellular organe...
0.04068
98 / 58793
GO:0043227
membrane−bounded org...
0.02062
98 / 57760
GO:0044422
organelle part
1.35e−07
52 / 17309
GO:0044444
cytoplasmic part
2.23e−08
83 / 34514
GO:0044446
intracellular organe...
1.27e−07
52 / 17278
GO:0043231
intracellular membra...
0.02925
97 / 57558
GO:0009536
plastid
7.43e−10
50 / 13957
GO:0044435
plastid part
3.15e−08
26 / 5038
GO:0009507
chloroplast
4.07e−10
50 / 13722
GO:0009532
plastid stroma
1.37e−08
20 / 2879
GO:0044434
chloroplast part
2.36e−08
26 / 4966
GO:0009570
chloroplast stroma
9.25e−09
20 / 2811
