# Supplementary material for: Differential Alternative Splicing Genes and Isoform Regulation Networks of Rapeseed (Brassica napus L.) Infected with Sclerotinia sclerotiorum
Source: Genes (Basel). 2020 Jul 13;11(7):784. doi: 10.3390/genes11070784 (PMC7397149; doi:10.3390/genes11070784)
Supplement: Supplementary file 1 [file genes-11-00784-s001.zip › Fig. S2 Enriched GO terms of genes in the 85 WGCNA modules.zip!/Fig. S2/CytoNodethistle3-go.pdf]

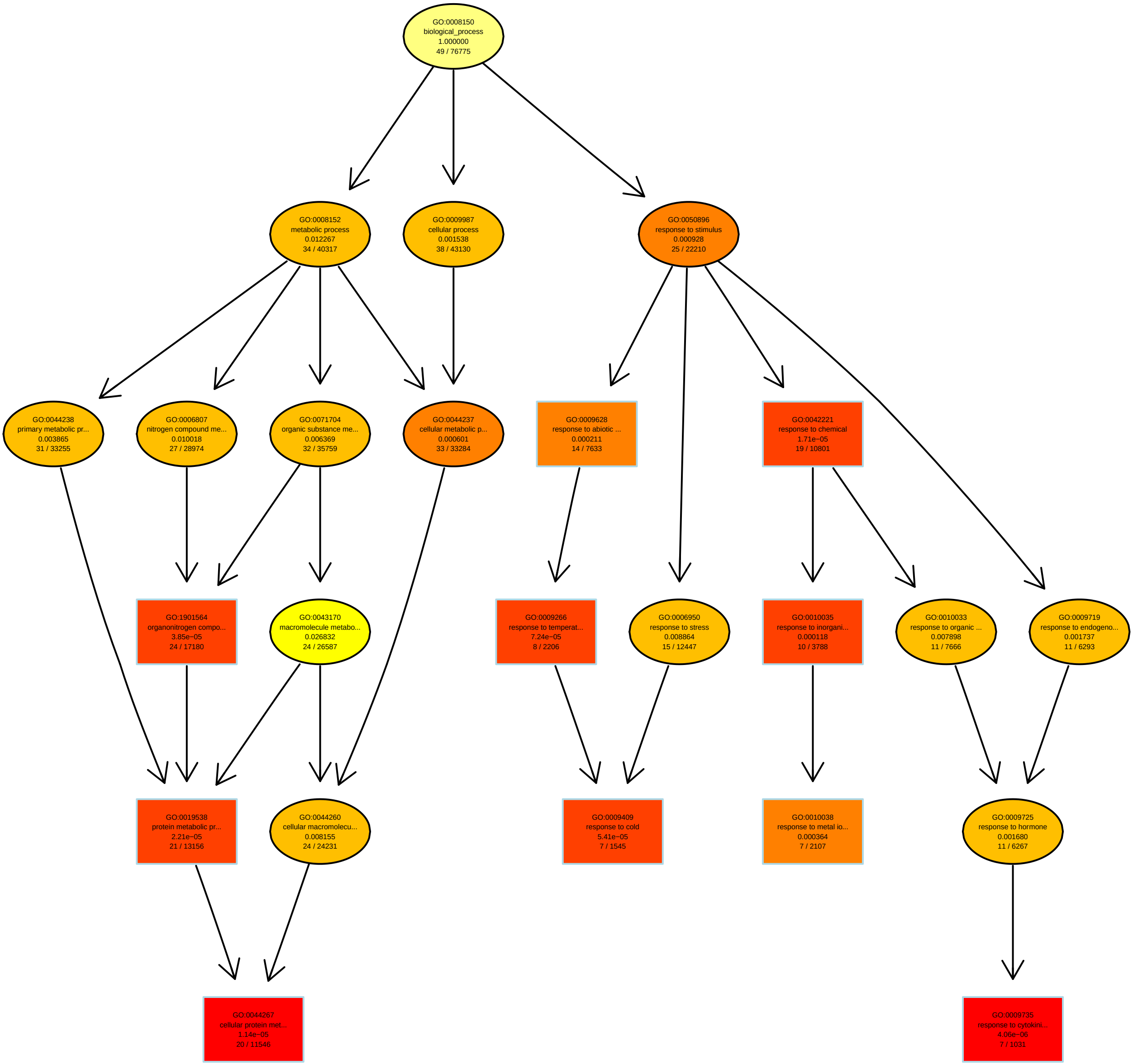
GO:0008150
biological_process
1.000000
49 / 76775
GO:0008152
metabolic process
0.012267
34 / 40317
GO:0009987
cellular process
0.001538
38 / 43130
GO:0050896
response to stimulus
0.000928
25 / 22210
GO:0044238
primary metabolic pr...
0.003865
31 / 33255
GO:0006807
nitrogen compound me...
0.010018
27 / 28974
GO:0071704
organic substance me...
0.006369
32 / 35759
GO:0044237
cellular metabolic p...
0.000601
33 / 33284
GO:0009628
response to abiotic ...
0.000211
14 / 7633
GO:0042221
response to chemical
1.71e−05
19 / 10801
GO:1901564
organonitrogen compo...
3.85e−05
24 / 17180
GO:0043170
macromolecule metabo...
0.026832
24 / 26587
GO:0009266
response to temperat...
7.24e−05
8 / 2206
GO:0006950
response to stress
0.008864
15 / 12447
GO:0010035
response to inorgani...
0.000118
10 / 3788
GO:0010033
response to organic ...
0.007898
11 / 7666
GO:0009719
response to endogeno...
0.001737
11 / 6293
GO:0019538
protein metabolic pr...
2.21e−05
21 / 13156
GO:0044260
cellular macromolecu...
0.008155
24 / 24231
GO:0009409
response to cold
5.41e−05
7 / 1545
GO:0010038
response to metal io...
0.000364
7 / 2107
GO:0009725
response to hormone
0.001680
11 / 6267
GO:0044267
cellular protein met...
1.14e−05
20 / 11546
GO:0009735
response to cytokini...
4.06e−06
7 / 1031

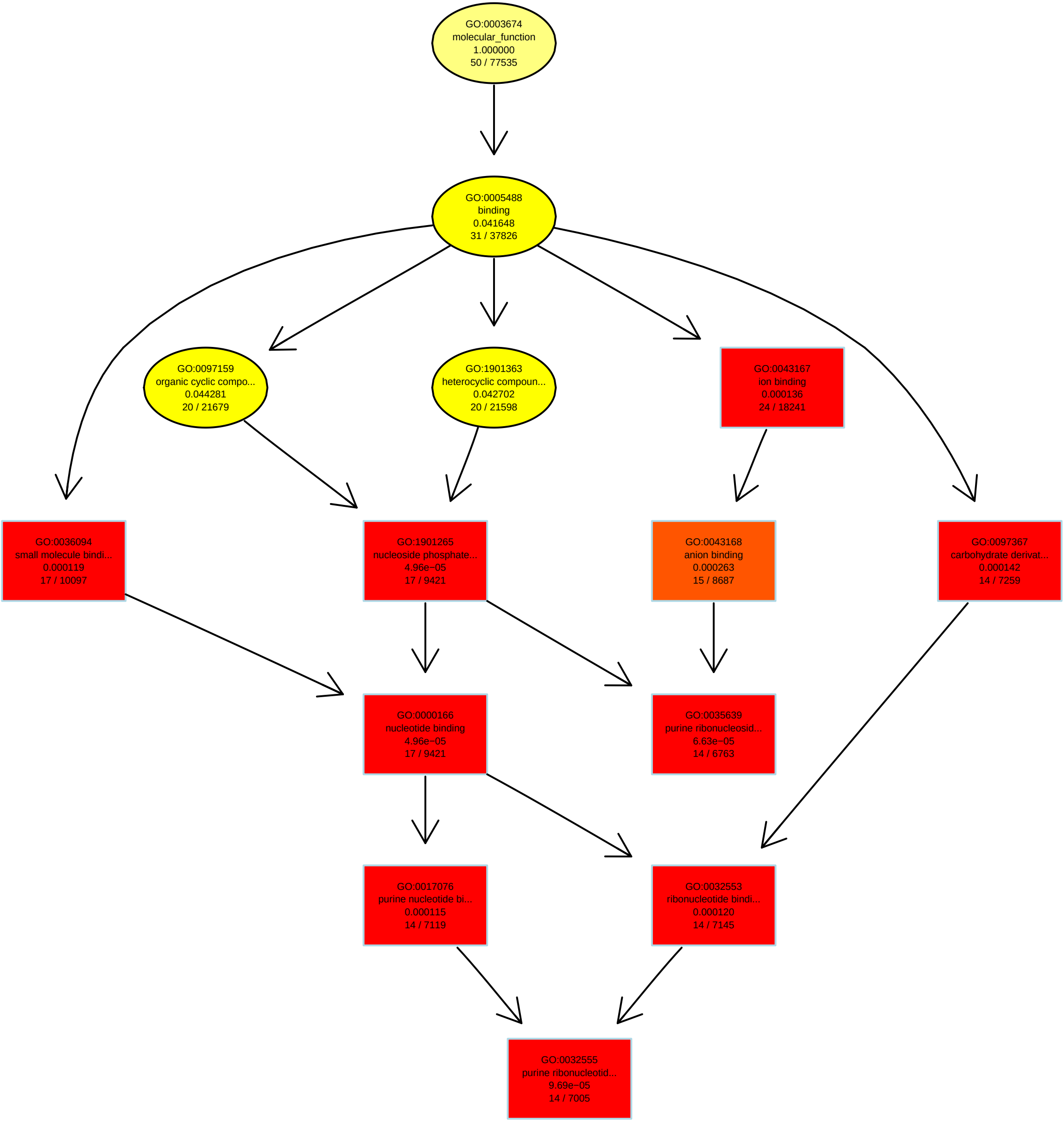
GO:0003674
molecular_function
1.000000
50 / 77535
GO:0005488
binding
0.041648
31 / 37826
GO:0097159
organic cyclic compo...
0.044281
20 / 21679
GO:1901363
heterocyclic compoun...
0.042702
20 / 21598
GO:0043167
ion binding
0.000136
24 / 18241
GO:0036094
small molecule bindi...
0.000119
17 / 10097
GO:1901265
nucleoside phosphate...
4.96e−05
17 / 9421
GO:0043168
anion binding
0.000263
15 / 8687
GO:0097367
carbohydrate derivat...
0.000142
14 / 7259
GO:0000166
nucleotide binding
4.96e−05
17 / 9421
GO:0035639
purine ribonucleosid...
6.63e−05
14 / 6763
GO:0017076
purine nucleotide bi...
0.000115
14 / 7119
GO:0032553
ribonucleotide bindi...
0.000120
14 / 7145
GO:0032555
purine ribonucleotid...
9.69e−05
14 / 7005

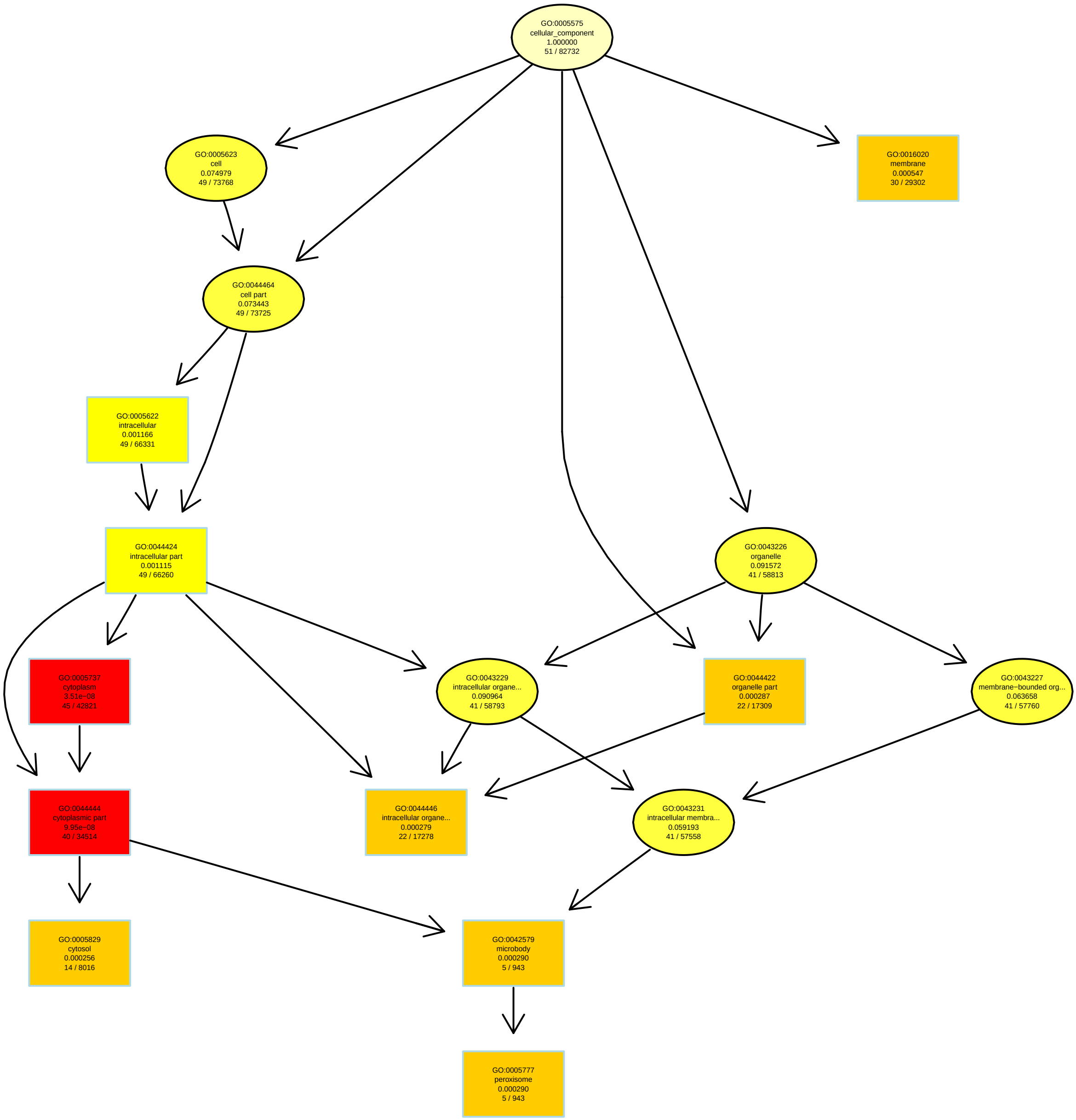
GO:0005575
cellular_component
1.000000
51 / 82732
GO:0005623
cell
0.074979
49 / 73768
GO:0016020
membrane
0.000547
30 / 29302
GO:0044464
cell part
0.073443
49 / 73725
GO:0005622
intracellular
0.001166
49 / 66331
GO:0044424
intracellular part
0.001115
49 / 66260
GO:0043226
organelle
0.091572
41 / 58813
GO:0005737
cytoplasm
3.51e−08
45 / 42821
GO:0043229
intracellular organe...
0.090964
41 / 58793
GO:0044422
organelle part
0.000287
22 / 17309
GO:0043227
membrane−bounded org...
0.063658
41 / 57760
GO:0044444
cytoplasmic part
9.95e−08
40 / 34514
GO:0044446
intracellular organe...
0.000279
22 / 17278
GO:0043231
intracellular membra...
0.059193
41 / 57558
GO:0005829
cytosol
0.000256
14 / 8016
GO:0042579
microbody
0.000290
5 / 943
GO:0005777
peroxisome
0.000290
5 / 943
