# Supplementary material for: Differential Alternative Splicing Genes and Isoform Regulation Networks of Rapeseed (Brassica napus L.) Infected with Sclerotinia sclerotiorum
Source: Genes (Basel). 2020 Jul 13;11(7):784. doi: 10.3390/genes11070784 (PMC7397149; doi:10.3390/genes11070784)
Supplement: Supplementary file 1 [file genes-11-00784-s001.zip › Fig. S2 Enriched GO terms of genes in the 85 WGCNA modules.zip!/Fig. S2/CytoNodeturquoise-go.pdf]

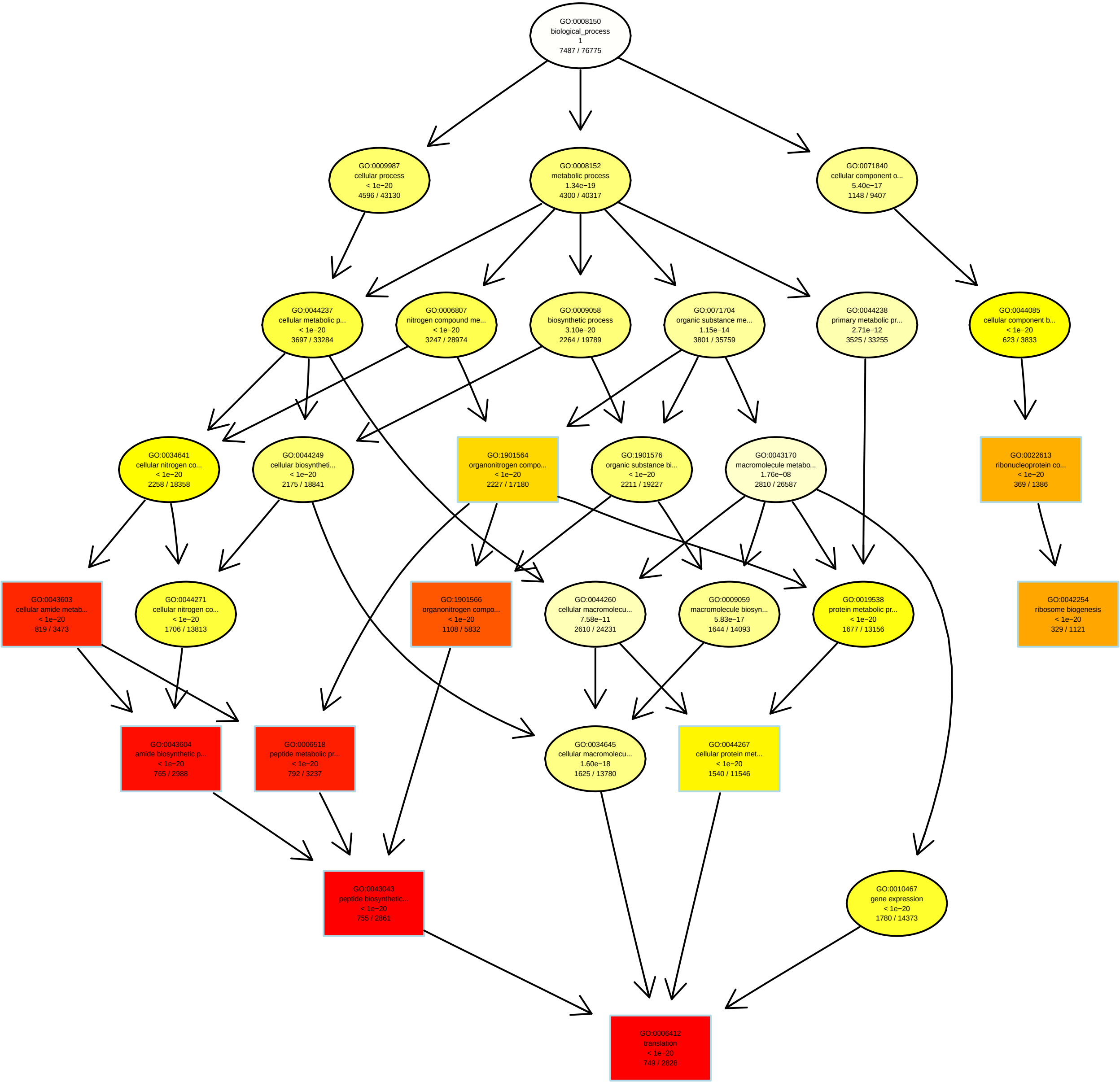
GO:0008150
biological_process
1
7487 / 76775
GO:0009987
cellular process
< 1e−20
4596 / 43130
GO:0008152
metabolic process
1.34e−19
4300 / 40317
GO:0071840
cellular component o...
5.40e−17
1148 / 9407
GO:0044237
cellular metabolic p...
< 1e−20
3697 / 33284
GO:0006807
nitrogen compound me...
< 1e−20
3247 / 28974
GO:0009058
biosynthetic process
3.10e−20
2264 / 19789
GO:0071704
organic substance me...
1.15e−14
3801 / 35759
GO:0044238
primary metabolic pr...
2.71e−12
3525 / 33255
GO:0044085
cellular component b...
< 1e−20
623 / 3833
GO:0034641
cellular nitrogen co...
< 1e−20
2258 / 18358
GO:0044249
cellular biosyntheti...
< 1e−20
2175 / 18841
GO:1901564
organonitrogen compo...
< 1e−20
2227 / 17180
GO:1901576
organic substance bi...
< 1e−20
2211 / 19227
GO:0043170
macromolecule metabo...
1.76e−08
2810 / 26587
GO:0022613
ribonucleoprotein co...
< 1e−20
369 / 1386
GO:0043603
cellular amide metab...
< 1e−20
819 / 3473
GO:0044271
cellular nitrogen co...
< 1e−20
1706 / 13813
GO:1901566
organonitrogen compo...
< 1e−20
1108 / 5832
GO:0044260
cellular macromolecu...
7.58e−11
2610 / 24231
GO:0009059
macromolecule biosyn...
5.83e−17
1644 / 14093
GO:0019538
protein metabolic pr...
< 1e−20
1677 / 13156
GO:0042254
ribosome biogenesis
< 1e−20
329 / 1121
GO:0043604
amide biosynthetic p...
< 1e−20
765 / 2988
GO:0006518
peptide metabolic pr...
< 1e−20
792 / 3237
GO:0034645
cellular macromolecu...
1.60e−18
1625 / 13780
GO:0044267
cellular protein met...
< 1e−20
1540 / 11546
GO:0043043
peptide biosynthetic...
< 1e−20
755 / 2861
GO:0010467
gene expression
< 1e−20
1780 / 14373
GO:0006412
translation
< 1e−20
749 / 2828

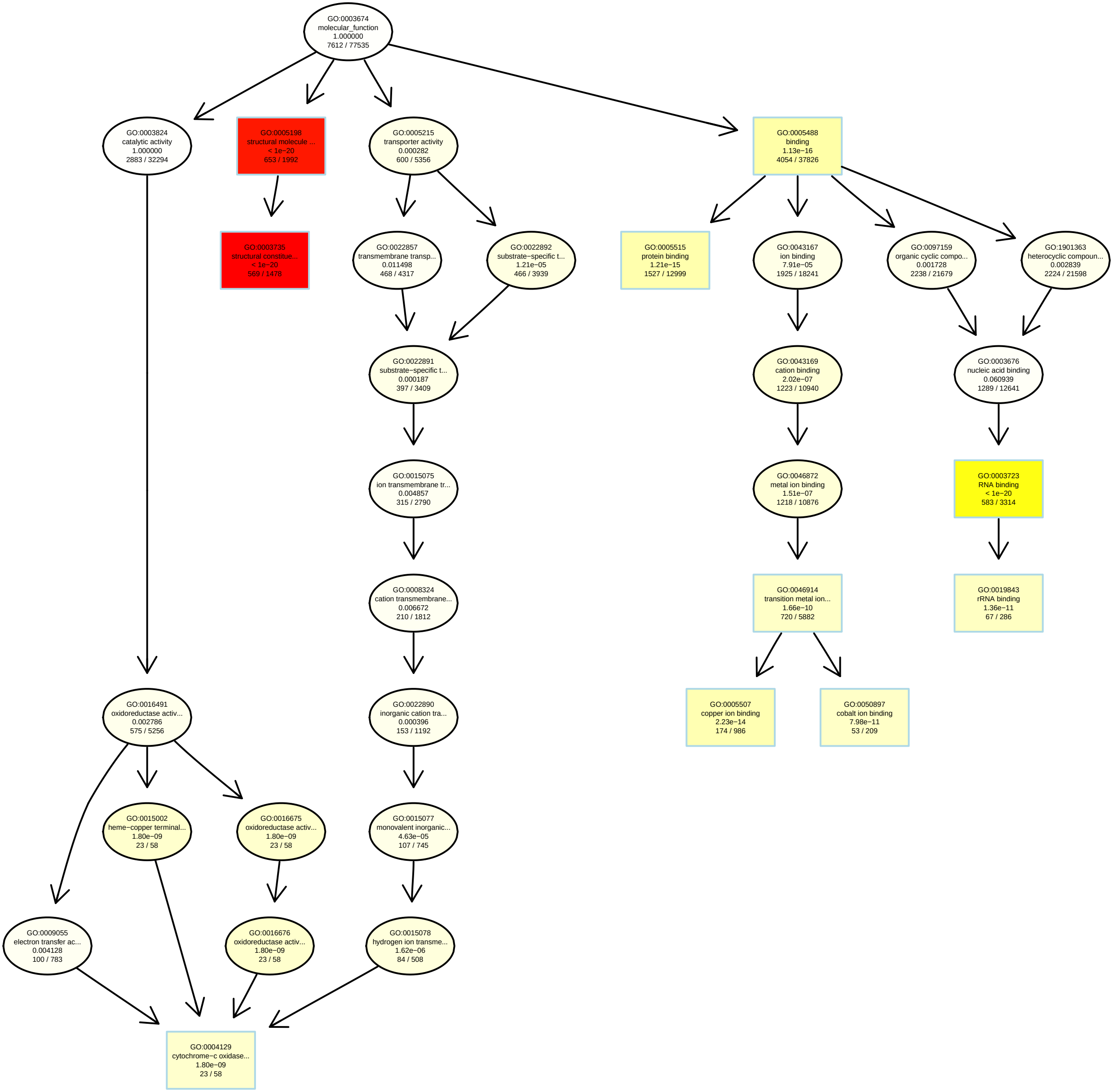
GO:0003674
molecular_function
1.000000
7612 / 77535
GO:0003824
catalytic activity
1.000000
2883 / 32294
GO:0005198
structural molecule ...
< 1e−20
653 / 1992
GO:0005215
transporter activity
0.000282
600 / 5356
GO:0005488
binding
1.13e−16
4054 / 37826
GO:0003735
structural constitue...
< 1e−20
569 / 1478
GO:0022857
transmembrane transp...
0.011498
468 / 4317
GO:0022892
substrate−specific t...
1.21e−05
466 / 3939
GO:0005515
protein binding
1.21e−15
1527 / 12999
GO:0043167
ion binding
7.91e−05
1925 / 18241
GO:0097159
organic cyclic compo...
0.001728
2238 / 21679
GO:1901363
heterocyclic compoun...
0.002839
2224 / 21598
GO:0022891
substrate−specific t...
0.000187
397 / 3409
GO:0043169
cation binding
2.02e−07
1223 / 10940
GO:0003676
nucleic acid binding
0.060939
1289 / 12641
GO:0015075
ion transmembrane tr...
0.004857
315 / 2790
GO:0046872
metal ion binding
1.51e−07
1218 / 10876
GO:0003723
RNA binding
< 1e−20
583 / 3314
GO:0008324
cation transmembrane...
0.006672
210 / 1812
GO:0046914
transition metal ion...
1.66e−10
720 / 5882
GO:0019843
rRNA binding
1.36e−11
67 / 286
GO:0016491
oxidoreductase activ...
0.002786
575 / 5256
GO:0022890
inorganic cation tra...
0.000396
153 / 1192
GO:0005507
copper ion binding
2.23e−14
174 / 986
GO:0050897
cobalt ion binding
7.98e−11
53 / 209
GO:0015002
heme−copper terminal...
1.80e−09
23 / 58
GO:0016675
oxidoreductase activ...
1.80e−09
23 / 58
GO:0015077
monovalent inorganic...
4.63e−05
107 / 745
GO:0009055
electron transfer ac...
0.004128
100 / 783
GO:0016676
oxidoreductase activ...
1.80e−09
23 / 58
GO:0015078
hydrogen ion transme...
1.62e−06
84 / 508
GO:0004129
cytochrome−c oxidase...
1.80e−09
23 / 58

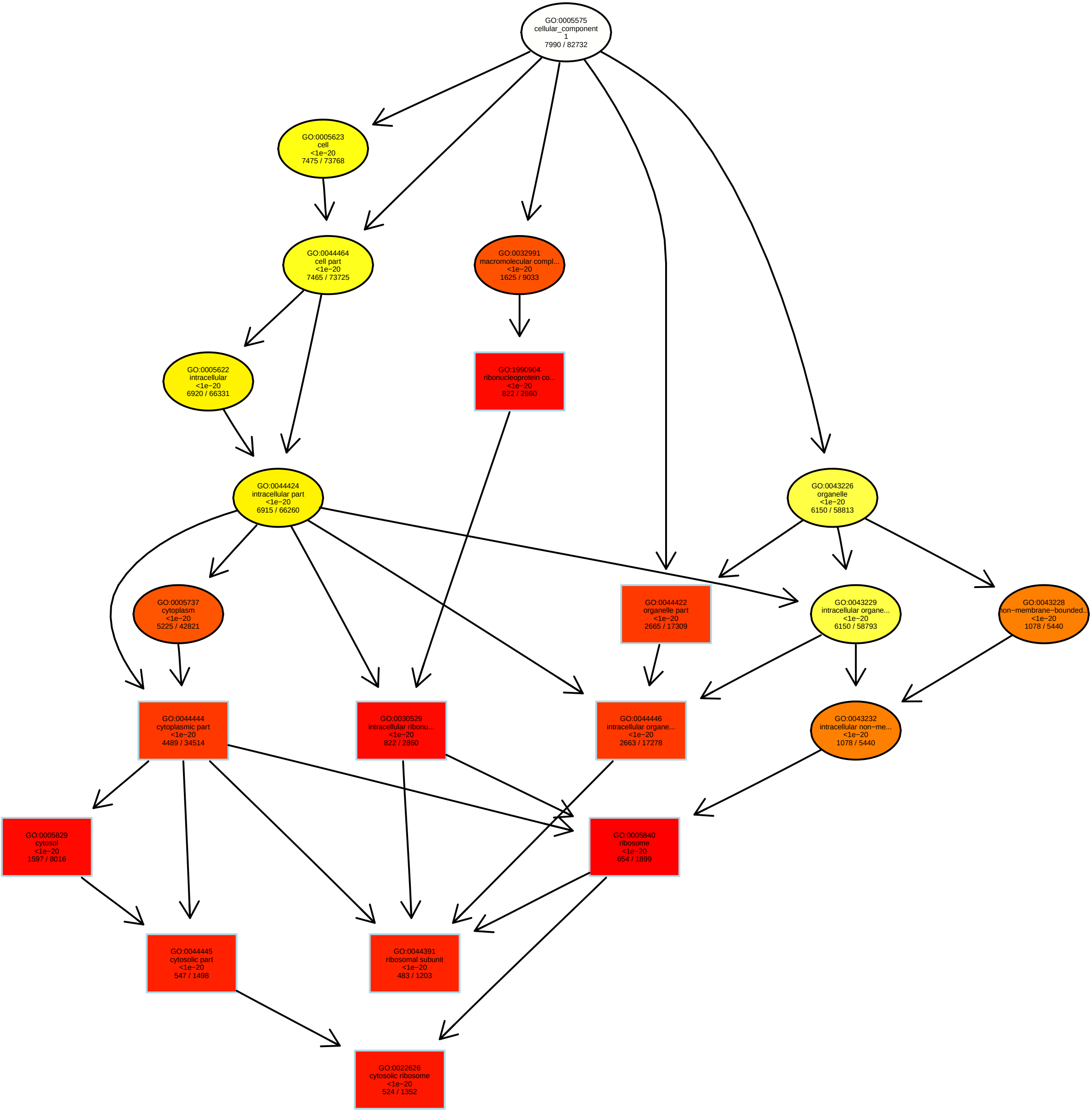

GO:0005575
cellular_component
1
7990 / 82732
GO:0005623
cell
<1e−20
7475 / 73768
GO:0044464
cell part
<1e−20
7465 / 73725
GO:0032991
macromolecular compl...
<1e−20
1625 / 9033
GO:0005622
intracellular
<1e−20
6920 / 66331
GO:1990904
ribonucleoprotein co...
<1e−20
822 / 2860
GO:0044424
intracellular part
<1e−20
6915 / 66260
GO:0043226
organelle
<1e−20
6150 / 58813
GO:0005737
cytoplasm
<1e−20
5225 / 42821
GO:0044422
organelle part
<1e−20
2665 / 17309
GO:0043229
intracellular organe...
<1e−20
6150 / 58793
GO:0043228
non−membrane−bounded...
<1e−20
1078 / 5440
GO:0044444
cytoplasmic part
<1e−20
4489 / 34514
GO:0030529
intracellular ribonu...
<1e−20
822 / 2860
GO:0044446
intracellular organe...
<1e−20
2663 / 17278
GO:0043232
intracellular non−me...
<1e−20
1078 / 5440
GO:0005829
cytosol
<1e−20
1597 / 8016
GO:0005840
ribosome
<1e−20
654 / 1899
GO:0044445
cytosolic part
<1e−20
547 / 1498
GO:0044391
ribosomal subunit
<1e−20
483 / 1203
GO:0022626
cytosolic ribosome
<1e−20
524 / 1352
