# Supplementary material for: Differential Alternative Splicing Genes and Isoform Regulation Networks of Rapeseed (Brassica napus L.) Infected with Sclerotinia sclerotiorum
Source: Genes (Basel). 2020 Jul 13;11(7):784. doi: 10.3390/genes11070784 (PMC7397149; doi:10.3390/genes11070784)
Supplement: Supplementary file 1 [file genes-11-00784-s001.zip › Fig. S2 Enriched GO terms of genes in the 85 WGCNA modules.zip!/Fig. S2/CytoNodeviolet-go.pdf]

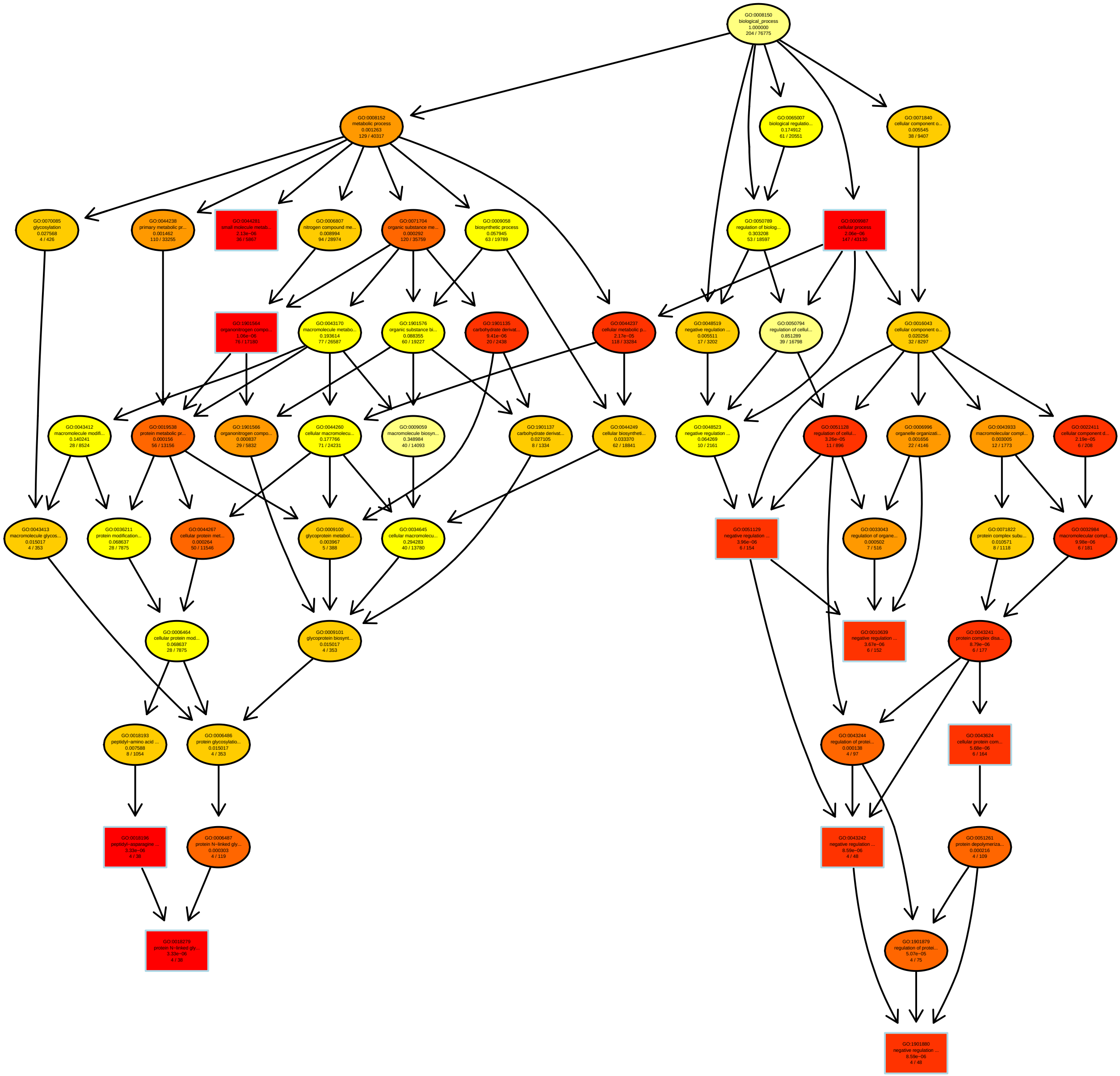
GO:0008150 biological_process 1.000000 204 / 76775
GO:0008152 metabolic process 0.001263 129 / 40317
GO:0065007 biological regulatio... 0.174912 61 / 20551
GO:0071840 cellular component o... 0.005545 38 / 9407
GO:0070085 glycosylation 0.027568 4 / 426
GO:0044238 primary metabolic pr... 0.001462 110 / 33255
GO:0044281 small molecule metab... 2.13e−06 36 / 5867
GO:0006807 nitrogen compound me... 0.008994 94 / 28974
GO:0071704 organic substance me... 0.000292 120 / 35759
GO:0009058 biosynthetic process 0.057945 63 / 19789
GO:0050789 regulation of biolog... 0.303208 53 / 18597
GO:0009987 cellular process 2.06e−06 147 / 43130
GO:1901564 organonitrogen compo... 1.06e−06 76 / 17180
GO:0043170 macromolecule metabo... 0.193614 77 / 26587
GO:1901576 organic substance bi... 0.088355 60 / 19227
GO:1901135 carbohydrate derivat... 9.41e−06 20 / 2438
GO:0044237 cellular metabolic p... 2.17e−05 118 / 33284
GO:0048519 negative regulation ... 0.005511 17 / 3202
GO:0050794 regulation of cellul... 0.851289 39 / 16798
GO:0016043 cellular component o... 0.020256 32 / 8297
GO:0043412 macromolecule modifi... 0.140241 28 / 8524
GO:0019538 protein metabolic pr... 0.000156 56 / 13156
GO:1901566 organonitrogen compo... 0.000837 29 / 5832
GO:0044260 cellular macromolecu... 0.177766 71 / 24231
GO:0009059 macromolecule biosyn... 0.348984 40 / 14093
GO:1901137 carbohydrate derivat... 0.027105 8 / 1334
GO:0044249 cellular biosyntheti... 0.033370 62 / 18841
GO:0048523 negative regulation ... 0.064269 10 / 2161
GO:0051128 regulation of cellul... 3.26e−05 11 / 896
GO:0006996 organelle organizati... 0.001656 22 / 4146
GO:0043933 macromolecular compl... 0.003005 12 / 1773
GO:0022411 cellular component d... 2.19e−05 6 / 208
GO:0043413 macromolecule glycos... 0.015017 4 / 353
GO:0036211 protein modification... 0.068637 28 / 7875
GO:0044267 cellular protein met... 0.000264 50 / 11546
GO:0009100 glycoprotein metabol... 0.003967 5 / 388
GO:0034645 cellular macromolecu... 0.294283 40 / 13780
GO:0051129 negative regulation ... 3.96e−06 6 / 154
GO:0033043 regulation of organe... 0.000502 7 / 516
GO:0071822 protein complex subu... 0.010571 8 / 1118
GO:0032984 macromolecular compl... 9.98e−06 6 / 181
GO:0006464 cellular protein mod... 0.068637 28 / 7875
GO:0009101 glycoprotein biosynt... 0.015017 4 / 353
GO:0010639 negative regulation ... 3.67e−06 6 / 152
GO:0043241 protein complex disa... 8.79e−06 6 / 177
GO:0018193 peptidyl−amino acid ... 0.007588 8 / 1054
GO:0006486 protein glycosylatio... 0.015017 4 / 353
GO:0043244 regulation of protei... 0.000138 4 / 97
GO:0043624 cellular protein com... 5.68e−06 6 / 164
GO:0018196 peptidyl−asparagine ... 3.33e−06 4 / 38
GO:0006487 protein N−linked gly... 0.000303 4 / 119
GO:0043242 negative regulation ... 8.59e−06 4 / 48
GO:0051261 protein depolymeriza... 0.000216 4 / 109
GO:0018279 protein N−linked gly... 3.33e−06 4 / 38
GO:1901879 regulation of protei... 5.07e−05 4 / 75
GO:1901880 negative regulation ... 8.59e−06 4 / 48

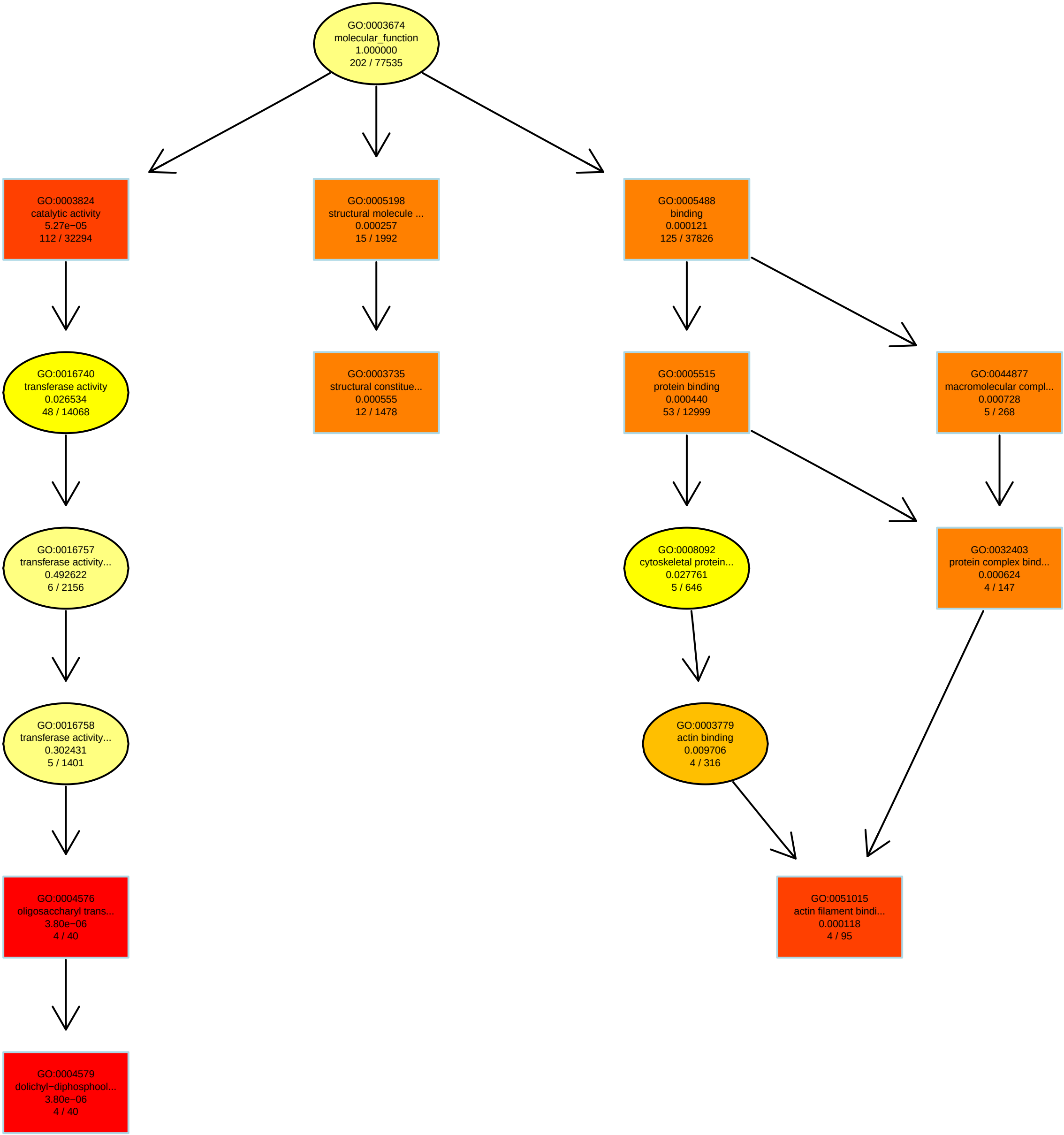

GO:0003674
molecular_function
1.000000
202 / 77535
GO:0003824
catalytic activity
5.27e−05
112 / 32294
GO:0005198
structural molecule ...
0.000257
15 / 1992
GO:0005488
binding
0.000121
125 / 37826
GO:0016740
transferase activity
0.026534
48 / 14068
GO:0003735
structural constitue...
0.000555
12 / 1478
GO:0005515
protein binding
0.000440
53 / 12999
GO:0044877
macromolecular compl...
0.000728
5 / 268
GO:0016757
transferase activity...
0.492622
6 / 2156
GO:0008092
cytoskeletal protein...
0.027761
5 / 646
GO:0032403
protein complex bind...
0.000624
4 / 147
GO:0016758
transferase activity...
0.302431
5 / 1401
GO:0003779
actin binding
0.009706
4 / 316
GO:0004576
oligosaccharyl trans...
3.80e−06
4 / 40
GO:0051015
actin filament bindi...
0.000118
4 / 95
GO:0004579
dolichyl−diphosphool...
3.80e−06
4 / 40

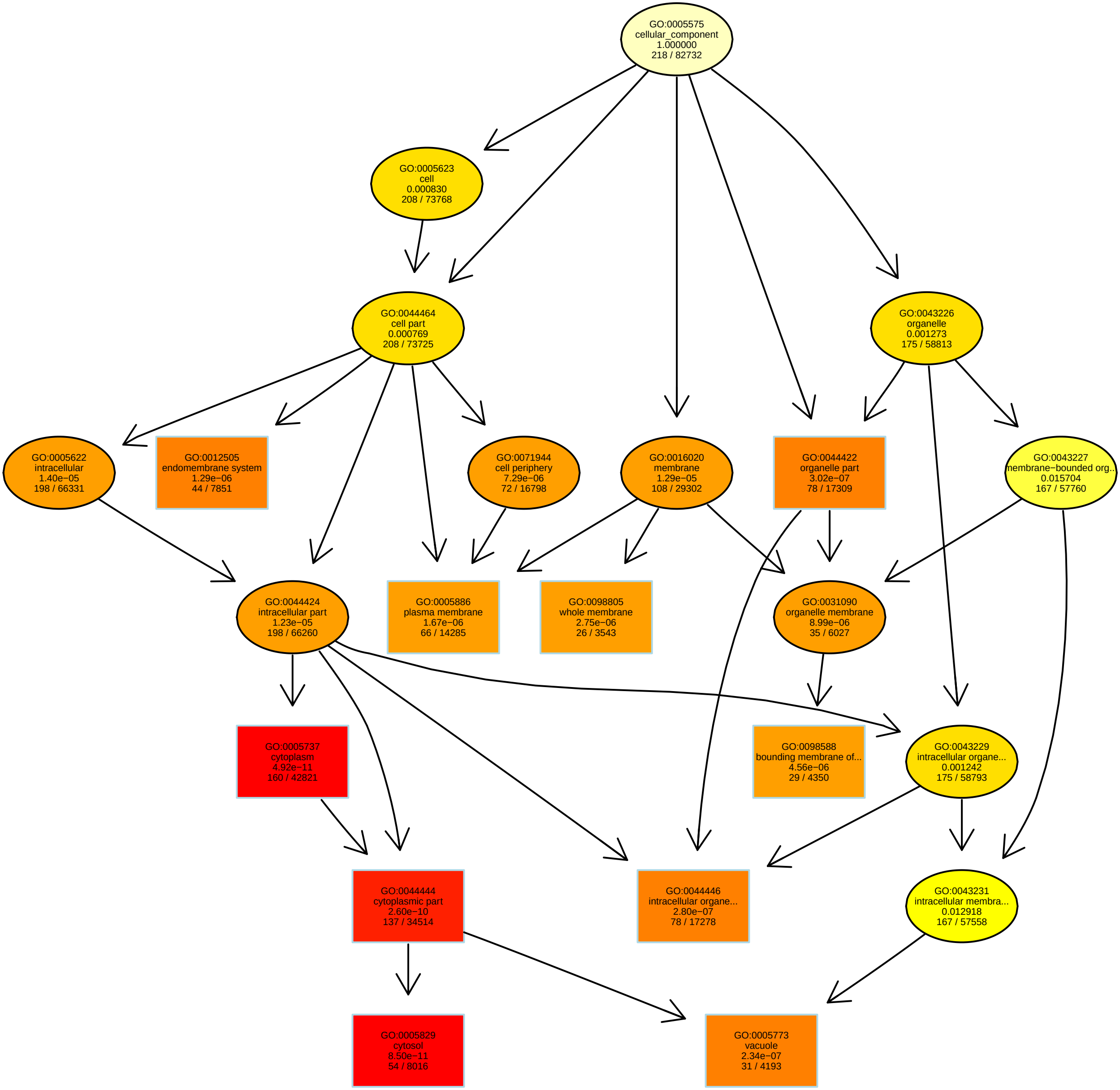
GO:0005575
cellular_component
1.000000
218 / 82732
GO:0005623
cell
0.000830
208 / 73768
GO:0044464
cell part
0.000769
208 / 73725
GO:0043226
organelle
0.001273
175 / 58813
GO:0005622
intracellular
1.40e−05
198 / 66331
GO:0012505
endomembrane system
1.29e−06
44 / 7851
GO:0071944
cell periphery
7.29e−06
72 / 16798
GO:0016020
membrane
1.29e−05
108 / 29302
GO:0044422
organelle part
3.02e−07
78 / 17309
GO:0043227
membrane−bounded org...
0.015704
167 / 57760
GO:0044424
intracellular part
1.23e−05
198 / 66260
GO:0005886
plasma membrane
1.67e−06
66 / 14285
GO:0098805
whole membrane
2.75e−06
26 / 3543
GO:0031090
organelle membrane
8.99e−06
35 / 6027
GO:0005737
cytoplasm
4.92e−11
160 / 42821
GO:0098588
bounding membrane of...
4.56e−06
29 / 4350
GO:0043229
intracellular organe...
0.001242
175 / 58793
GO:0044444
cytoplasmic part
2.60e−10
137 / 34514
GO:0044446
intracellular organe...
2.80e−07
78 / 17278
GO:0043231
intracellular membra...
0.012918
167 / 57558
GO:0005829
cytosol
8.50e−11
54 / 8016
GO:0005773
vacuole
2.34e−07
31 / 4193
