# Supplementary material for: Differential Alternative Splicing Genes and Isoform Regulation Networks of Rapeseed (Brassica napus L.) Infected with Sclerotinia sclerotiorum
Source: Genes (Basel). 2020 Jul 13;11(7):784. doi: 10.3390/genes11070784 (PMC7397149; doi:10.3390/genes11070784)
Supplement: Supplementary file 1 [file genes-11-00784-s001.zip › Fig. S2 Enriched GO terms of genes in the 85 WGCNA modules.zip!/Fig. S2/CytoNodewhite-go.pdf]

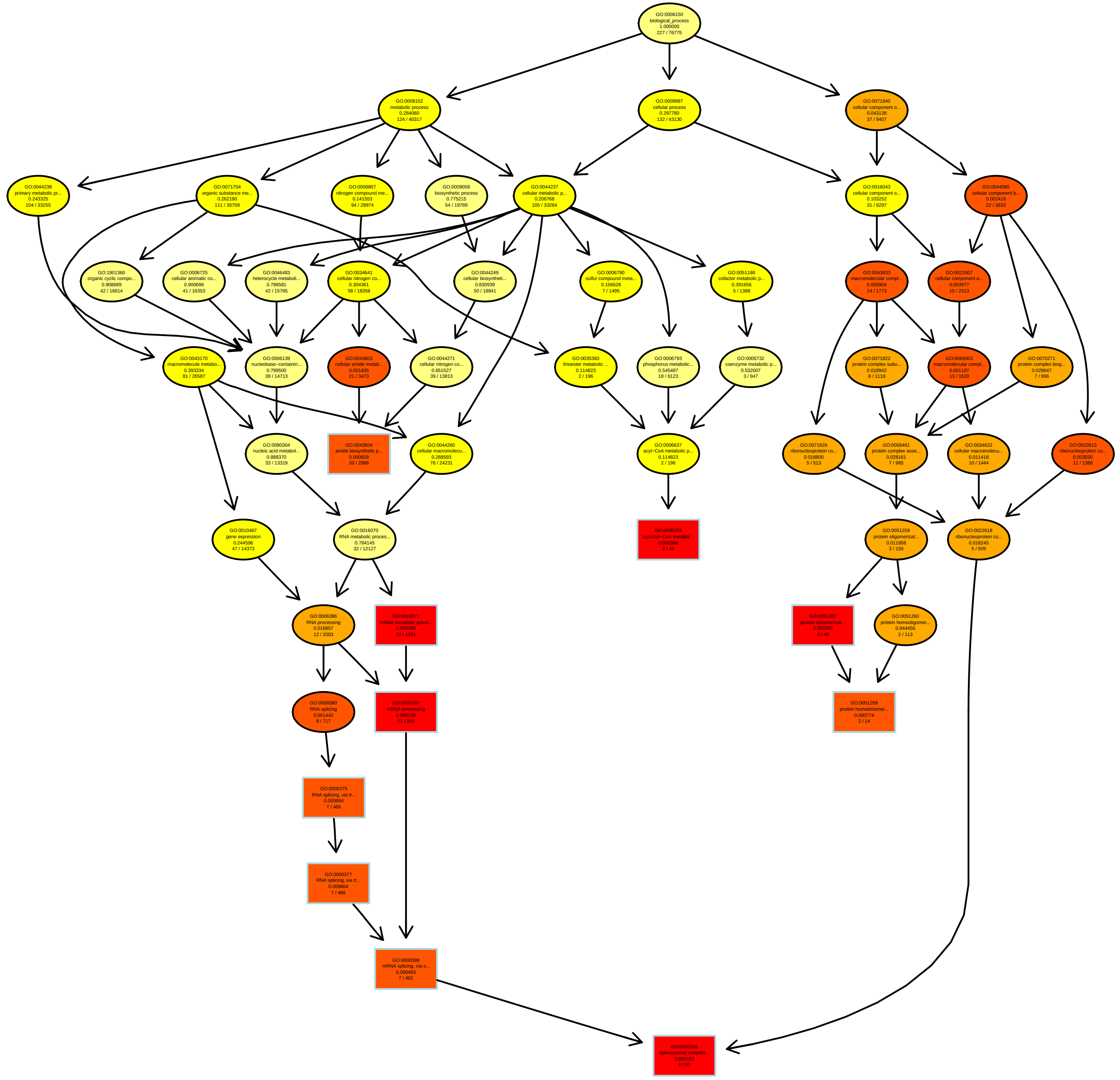

GO:0008150
biological_process
1.000000
227 / 76775
GO:0008152
metabolic process
0.284060
124 / 40317
GO:0009987
cellular process
0.297780
132 / 43130
GO:0071840
cellular component o...
0.043138
37 / 9407
GO:0044238
primary metabolic pr...
0.243325
104 / 33255
GO:0071704
organic substance me...
0.262190
111 / 35759
GO:0006807
nitrogen compound me...
0.141553
94 / 28974
GO:0009058
biosynthetic process
0.775215
54 / 19789
GO:0044237
cellular metabolic p...
0.206768
105 / 33284
GO:0016043
cellular component o...
0.103252
31 / 8297
GO:0044085
cellular component b...
0.002418
22 / 3833
GO:1901360
organic cyclic compo...
0.908689
42 / 16814
GO:0006725
cellular aromatic co...
0.900696
41 / 16353
GO:0046483
heterocycle metaboli...
0.798581
42 / 15765
GO:0034641
cellular nitrogen co...
0.304361
58 / 18358
GO:0044249
cellular biosyntheti...
0.830939
50 / 18841
GO:0006790
sulfur compound meta...
0.156628
7 / 1495
GO:0051186
cofactor metabolic p...
0.391656
5 / 1388
GO:0043933
macromolecular compl...
0.000904
14 / 1773
GO:0022607
cellular component a...
0.003977
15 / 2313
GO:0043170
macromolecule metabo...
0.393334
81 / 26587
GO:0006139
nucleobase−containin...
0.799500
39 / 14713
GO:0043603
cellular amide metab...
0.001635
21 / 3473
GO:0044271
cellular nitrogen co...
0.651527
39 / 13813
GO:0035383
thioester metabolic ...
0.114823
2 / 196
GO:0006793
phosphorus metabolic...
0.545487
18 / 6123
GO:0006732
coenzyme metabolic p...
0.532007
3 / 947
GO:0071822
protein complex subu...
0.018942
8 / 1118
GO:0065003
macromolecular compl...
0.001187
13 / 1620
GO:0070271
protein complex biog...
0.029647
7 / 996
GO:0090304
nucleic acid metabol...
0.888370
33 / 13319
GO:0043604
amide biosynthetic p...
0.000629
20 / 2988
GO:0044260
cellular macromolecu...
0.288593
76 / 24231
GO:0006637
acyl−CoA metabolic p...
0.114823
2 / 196
GO:0071826
ribonucleoprotein co...
0.018800
5 / 513
GO:0006461
protein complex asse...
0.028161
7 / 985
GO:0034622
cellular macromolecu...
0.011418
10 / 1444
GO:0022613
ribonucleoprotein co...
0.003030
11 / 1386
GO:0010467
gene expression
0.244596
47 / 14373
GO:0016070
RNA metabolic proces...
0.784145
32 / 12127
GO:0006104
succinyl−CoA metabol...
0.000386
2 / 10
GO:0051259
protein oligomerizat...
0.011958
3 / 159
GO:0022618
ribonucleoprotein co...
0.018245
5 / 509
GO:0006396
RNA processing
0.016857
12 / 2003
GO:0016071
mRNA metabolic proce...
0.000396
12 / 1251
GO:0051262
protein tetramerizat...
0.000352
3 / 46
GO:0051260
protein homooligomer...
0.044456
2 / 113
GO:0008380
RNA splicing
0.001442
8 / 717
GO:0006397
mRNA processing
0.000155
11 / 960
GO:0051289
protein homotetramer...
0.000774
2 / 14
GO:0000375
RNA splicing, via tr...
0.000664
7 / 486
GO:0000377
RNA splicing, via tr...
0.000664
7 / 486
GO:0000398
mRNA splicing, via s...
0.000493
7 / 462
GO:0000245
spliceosomal complex...
0.000163
4 / 91

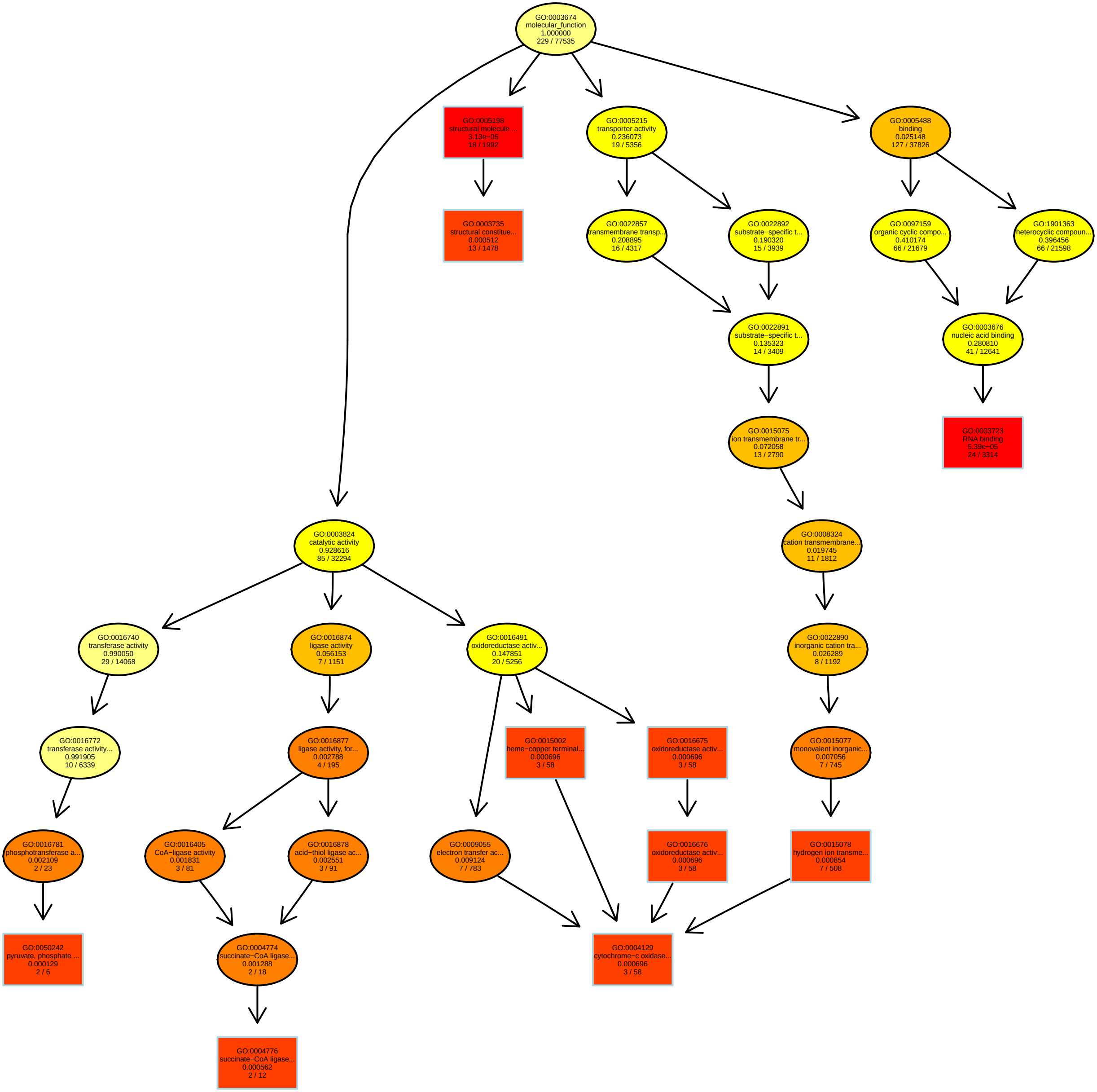
GO:0003674 molecular_function 1.000000 229 / 77535
GO:0005198 structural molecule ... 3.13e−05 18 / 1992
GO:0005215 transporter activity 0.236073 19 / 5356
GO:0005488 binding 0.025148 127 / 37826
GO:0003735 structural constitue... 0.000512 13 / 1478
GO:0022857 transmembrane transp... 0.208895 16 / 4317
GO:0022892 substrate−specific t... 0.190320 15 / 3939
GO:0097159 organic cyclic compo... 0.410174 66 / 21679
GO:1901363 heterocyclic compoun... 0.396456 66 / 21598
GO:0022891 substrate−specific t... 0.135323 14 / 3409
GO:0003676 nucleic acid binding 0.280810 41 / 12641
GO:0015075 ion transmembrane tr... 0.072058 13 / 2790
GO:0003723 RNA binding 5.39e−05 24 / 3314
GO:0003824 catalytic activity 0.928616 85 / 32294
GO:0008324 cation transmembrane... 0.019745 11 / 1812
GO:0016740 transferase activity 0.990050 29 / 14068
GO:0016874 ligase activity 0.056153 7 / 1151
GO:0016491 oxidoreductase activ... 0.147851 20 / 5256
GO:0022890 inorganic cation tra... 0.026289 8 / 1192
GO:0016772 transferase activity... 0.991905 10 / 6339
GO:0016877 ligase activity, for... 0.002788 4 / 195
GO:0015002 heme−copper terminal... 0.000696 3 / 58
GO:0016675 oxidoreductase activ... 0.000696 3 / 58
GO:0015077 monovalent inorganic... 0.007056 7 / 745
GO:0016781 phosphotransferase a... 0.002109 2 / 23
GO:0016405 CoA−ligase activity 0.001831 3 / 81
GO:0016878 acid−thiol ligase ac... 0.002551 3 / 91
GO:0009055 electron transfer ac... 0.009124 7 / 783
GO:0016676 oxidoreductase activ... 0.000696 3 / 58
GO:0015078 hydrogen ion transme... 0.000854 7 / 508
GO:0050242 pyruvate, phosphate ... 0.000129 2 / 6
GO:0004774 succinate−CoA ligase... 0.001288 2 / 18
GO:0004129 cytochrome−c oxidase... 0.000696 3 / 58
GO:0004776 succinate−CoA ligase... 0.000562 2 / 12

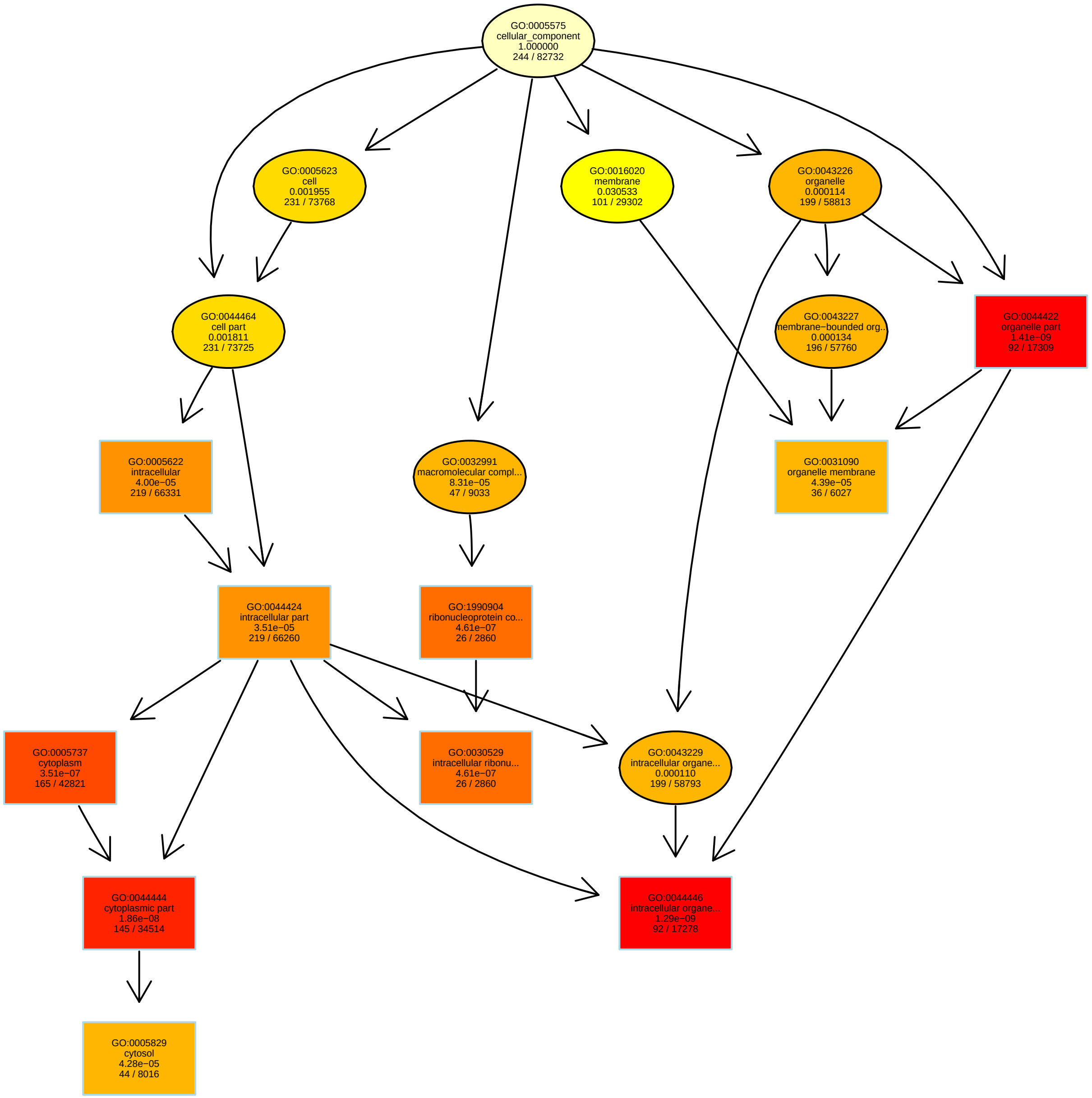
GO:0005575
cellular_component
1.000000
244 / 82732
GO:0005623
cell
0.001955
231 / 73768
GO:0016020
membrane
0.030533
101 / 29302
GO:0043226
organelle
0.000114
199 / 58813
GO:0044464
cell part
0.001811
231 / 73725
GO:0043227
membrane−bounded org...
0.000134
196 / 57760
GO:0044422
organelle part
1.41e−09
92 / 17309
GO:0005622
intracellular
4.00e−05
219 / 66331
GO:0032991
macromolecular compl...
8.31e−05
47 / 9033
GO:0031090
organelle membrane
4.39e−05
36 / 6027
GO:0044424
intracellular part
3.51e−05
219 / 66260
GO:1990904
ribonucleoprotein co...
4.61e−07
26 / 2860
GO:0005737
cytoplasm
3.51e−07
165 / 42821
GO:0030529
intracellular ribonu...
4.61e−07
26 / 2860
GO:0043229
intracellular organe...
0.000110
199 / 58793
GO:0044444
cytoplasmic part
1.86e−08
145 / 34514
GO:0044446
intracellular organe...
1.29e−09
92 / 17278
GO:0005829
cytosol
4.28e−05
44 / 8016
