# Supplementary material for: Differential Alternative Splicing Genes and Isoform Regulation Networks of Rapeseed (Brassica napus L.) Infected with Sclerotinia sclerotiorum
Source: Genes (Basel). 2020 Jul 13;11(7):784. doi: 10.3390/genes11070784 (PMC7397149; doi:10.3390/genes11070784)
Supplement: Supplementary file 1 [file genes-11-00784-s001.zip › Fig. S2 Enriched GO terms of genes in the 85 WGCNA modules.zip!/Fig. S2/CytoNodeyellow-go.pdf]

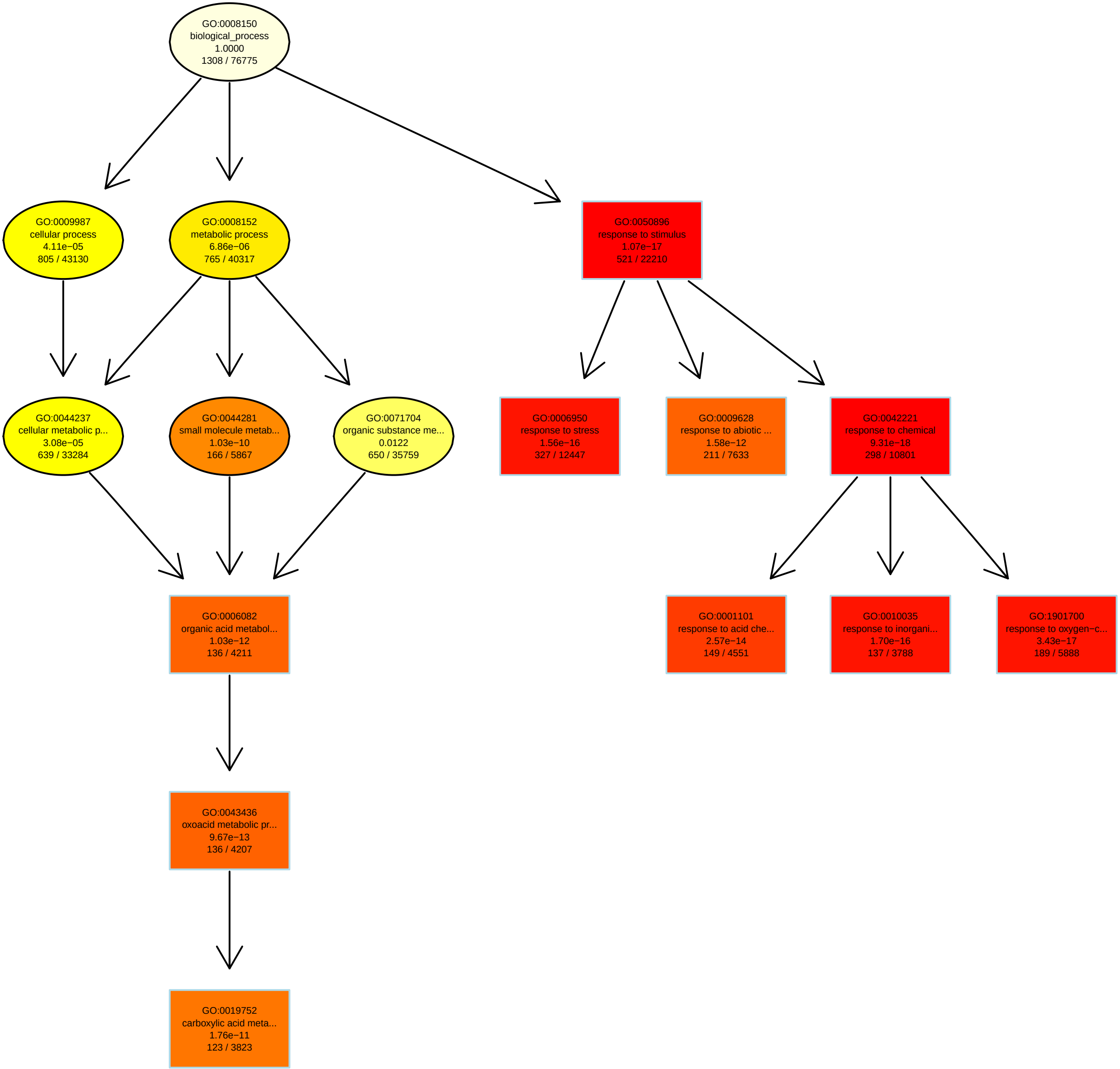
GO:0008150
biological_process
1.0000
1308 / 76775
GO:0009987
cellular process
4.11e−05
805 / 43130
GO:0008152
metabolic process
6.86e−06
765 / 40317
GO:0050896
response to stimulus
1.07e−17
521 / 22210
GO:0044237
cellular metabolic p...
3.08e−05
639 / 33284
GO:0044281
small molecule metab...
1.03e−10
166 / 5867
GO:0071704
organic substance me...
0.0122
650 / 35759
GO:0006950
response to stress
1.56e−16
327 / 12447
GO:0009628
response to abiotic ...
1.58e−12
211 / 7633
GO:0042221
response to chemical
9.31e−18
298 / 10801
GO:0006082
organic acid metabol...
1.03e−12
136 / 4211
GO:0001101
response to acid che...
2.57e−14
149 / 4551
GO:0010035
response to inorgani...
1.70e−16
137 / 3788
GO:1901700
response to oxygen−c...
3.43e−17
189 / 5888
GO:0043436
oxoacid metabolic pr...
9.67e−13
136 / 4207
GO:0019752
carboxylic acid meta...
1.76e−11
123 / 3823

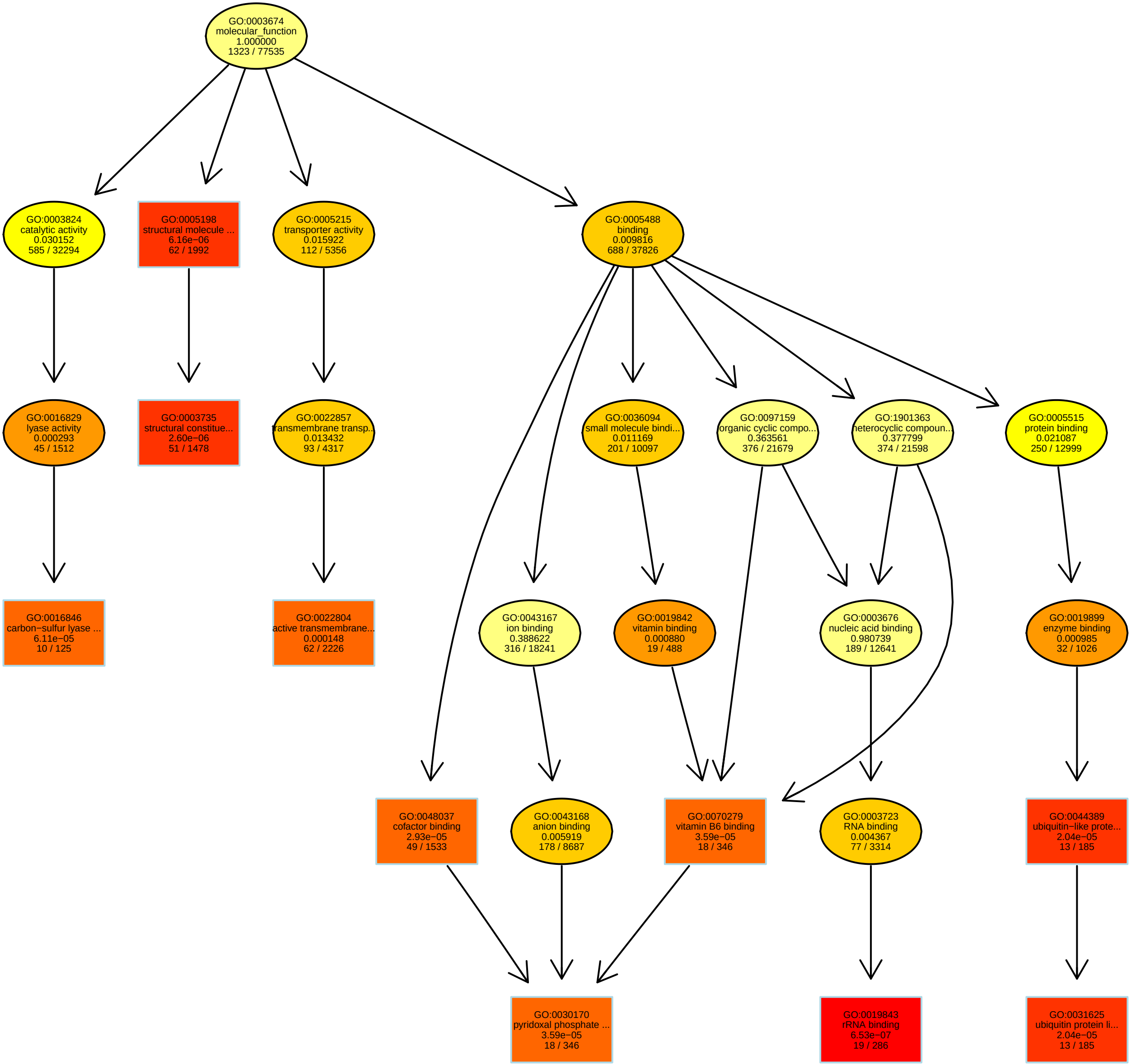
GO:0003674 molecular_function 1.000000 1323 / 77535
GO:0003824 catalytic activity 0.030152 585 / 32294
GO:0005198 structural molecule ... 6.16e−06 62 / 1992
GO:0005215 transporter activity 0.015922 112 / 5356
GO:0005488 binding 0.009816 688 / 37826
GO:0016829 lyase activity 0.000293 45 / 1512
GO:0003735 structural constitue... 2.60e−06 51 / 1478
GO:0022857 transmembrane transp... 0.013432 93 / 4317
GO:0036094 small molecule bindi... 0.011169 201 / 10097
GO:0097159 organic cyclic compo... 0.363561 376 / 21679
GO:1901363 heterocyclic compoun... 0.377799 374 / 21598
GO:0005515 protein binding 0.021087 250 / 12999
GO:0016846 carbon−sulfur lyase ... 6.11e−05 10 / 125
GO:0022804 active transmembrane... 0.000148 62 / 2226
GO:0043167 ion binding 0.388622 316 / 18241
GO:0019842 vitamin binding 0.000880 19 / 488
GO:0003676 nucleic acid binding 0.980739 189 / 12641
GO:0019899 enzyme binding 0.000985 32 / 1026
GO:0048037 cofactor binding 2.93e−05 49 / 1533
GO:0043168 anion binding 0.005919 178 / 8687
GO:0070279 vitamin B6 binding 3.59e−05 18 / 346
GO:0003723 RNA binding 0.004367 77 / 3314
GO:0044389 ubiquitin−like prote... 2.04e−05 13 / 185
GO:0030170 pyridoxal phosphate ... 3.59e−05 18 / 346
GO:0019843 rRNA binding 6.53e−07 19 / 286
GO:0031625 ubiquitin protein li... 2.04e−05 13 / 185

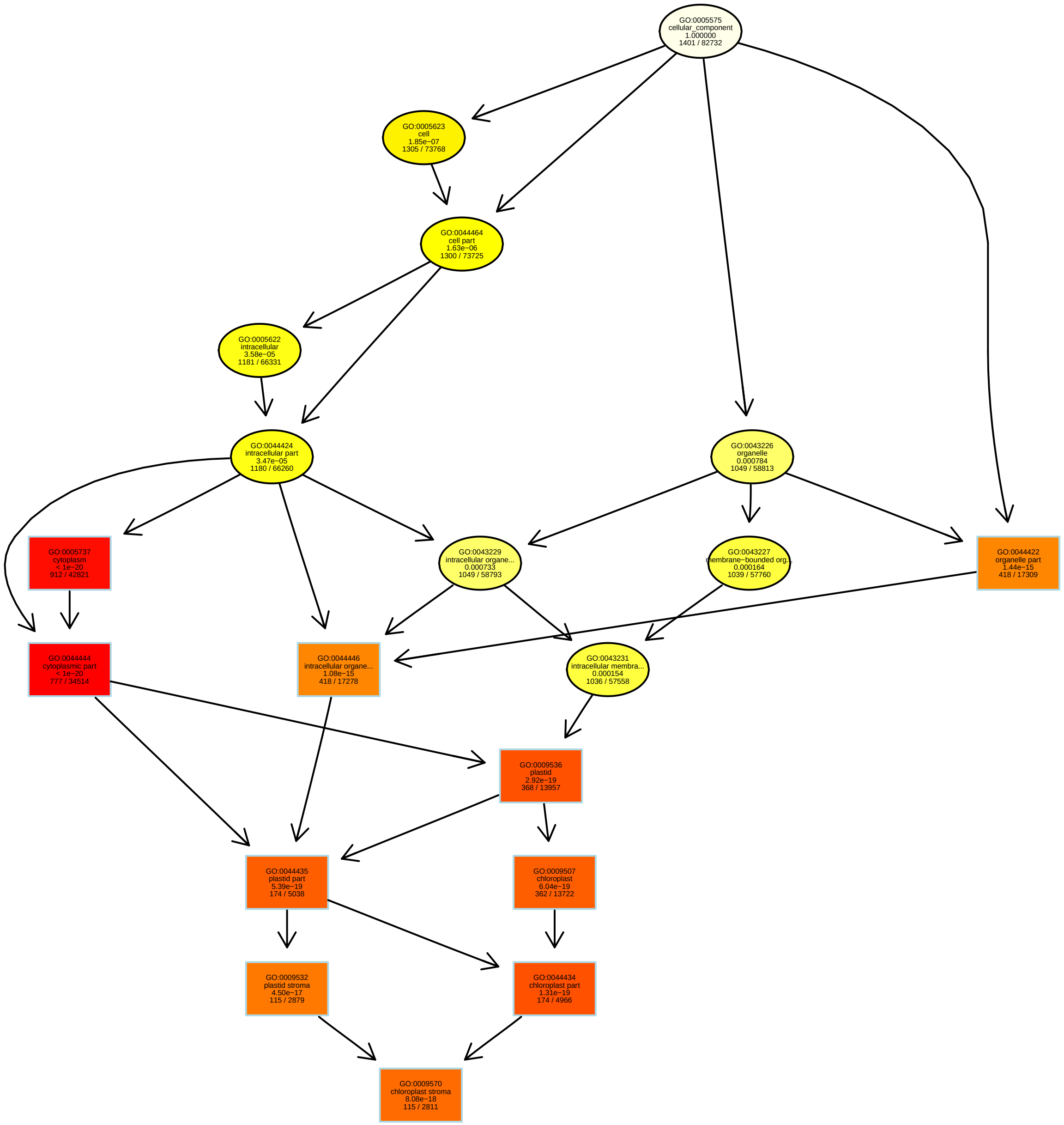

GO:0005575
cellular_component
1.000000
1401 / 82732
GO:0005623
cell
1.85e−07
1305 / 73768
GO:0044464
cell part
1.63e−06
1300 / 73725
GO:0005622
intracellular
3.58e−05
1181 / 66331
GO:0044424
intracellular part
3.47e−05
1180 / 66260
GO:0043226
organelle
0.000784
1049 / 58813
GO:0005737
cytoplasm
< 1e−20
912 / 42821
GO:0043229
intracellular organe...
0.000733
1049 / 58793
GO:0043227
membrane−bounded org...
0.000164
1039 / 57760
GO:0044422
organelle part
1.44e−15
418 / 17309
GO:0044444
cytoplasmic part
< 1e−20
777 / 34514
GO:0044446
intracellular organe...
1.08e−15
418 / 17278
GO:0043231
intracellular membra...
0.000154
1036 / 57558
GO:0009536
plastid
2.92e−19
368 / 13957
GO:0044435
plastid part
5.39e−19
174 / 5038
GO:0009507
chloroplast
6.04e−19
362 / 13722
GO:0009532
plastid stroma
4.50e−17
115 / 2879
GO:0044434
chloroplast part
1.31e−19
174 / 4966
GO:0009570
chloroplast stroma
8.08e−18
115 / 2811
