# Supplementary material for: Differential Alternative Splicing Genes and Isoform Regulation Networks of Rapeseed (Brassica napus L.) Infected with Sclerotinia sclerotiorum
Source: Genes (Basel). 2020 Jul 13;11(7):784. doi: 10.3390/genes11070784 (PMC7397149; doi:10.3390/genes11070784)
Supplement: Supplementary file 1 [file genes-11-00784-s001.zip › Fig. S2 Enriched GO terms of genes in the 85 WGCNA modules.zip!/Fig. S2/CytoNodeyellow4-go.pdf]

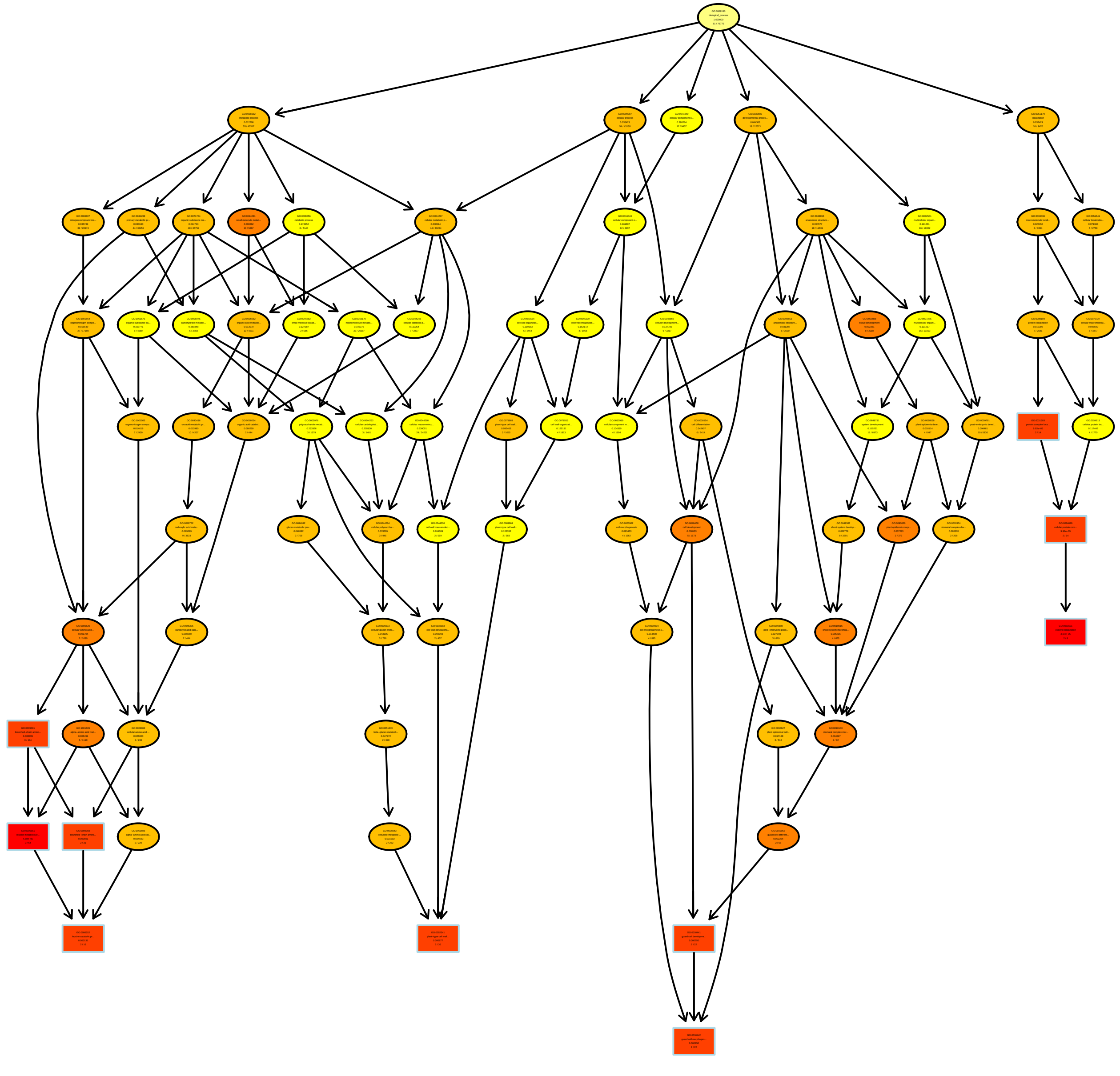

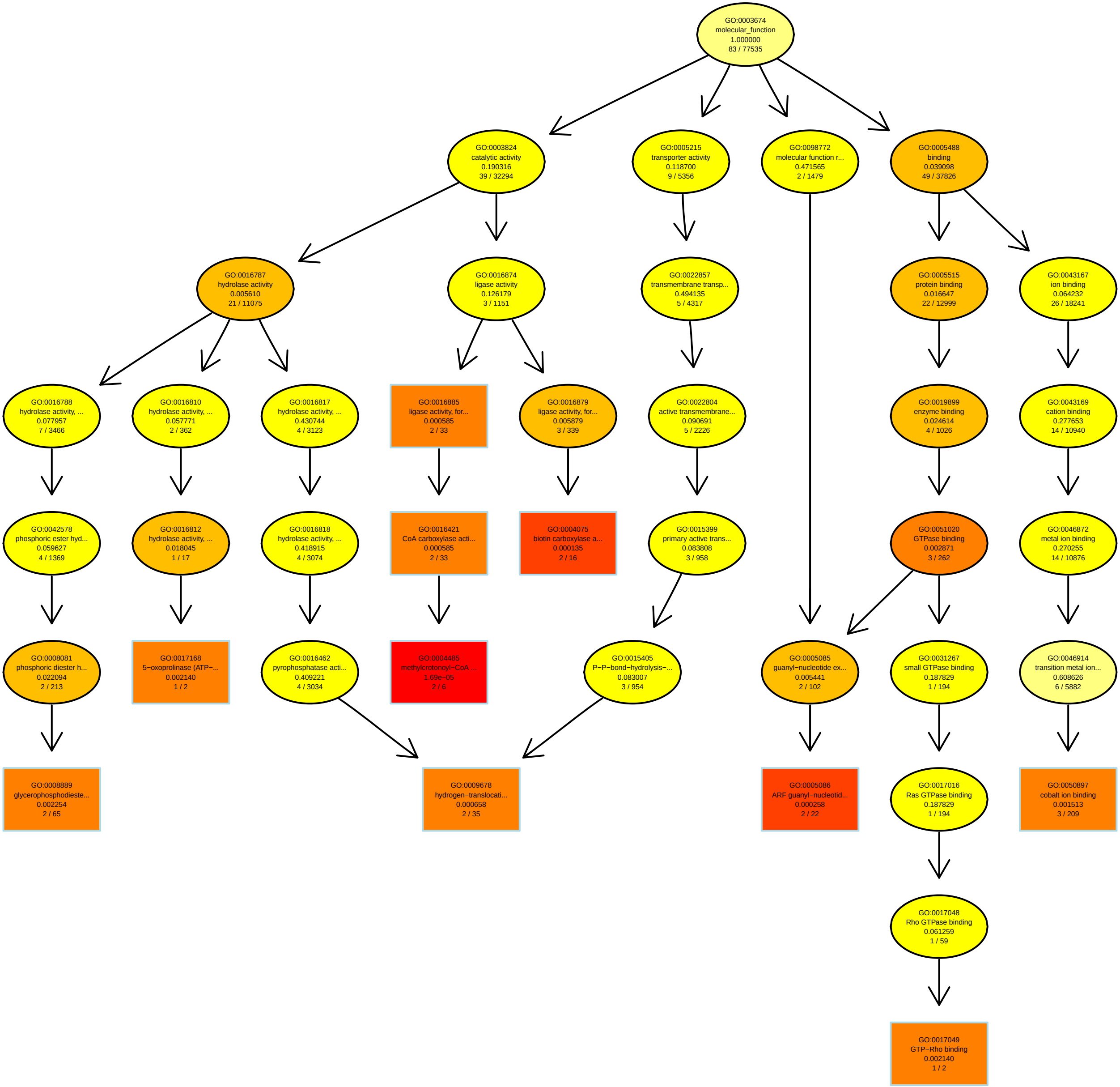
GO:0003674 molecular_function 1.000000 83 / 77535
GO:0003824 catalytic activity 0.190316 39 / 32294
GO:0005215 transporter activity 0.118700 9 / 5356
GO:0098772 molecular function r... 0.471565 2 / 1479
GO:0005488 binding 0.039098 49 / 37826
GO:0016787 hydrolase activity 0.005610 21 / 11075
GO:0016874 ligase activity 0.126179 3 / 1151
GO:0022857 transmembrane transp... 0.494135 5 / 4317
GO:0005515 protein binding 0.016647 22 / 12999
GO:0043167 ion binding 0.064232 26 / 18241
GO:0016788 hydrolase activity, ... 0.077957 7 / 3466
GO:0016810 hydrolase activity, ... 0.057771 2 / 362
GO:0016817 hydrolase activity, ... 0.430744 4 / 3123
GO:0016885 ligase activity, for... 0.000585 2 / 33
GO:0016879 ligase activity, for... 0.005879 3 / 339
GO:0022804 active transmembrane... 0.090691 5 / 2226
GO:0019899 enzyme binding 0.024614 4 / 1026
GO:0043169 cation binding 0.277653 14 / 10940
GO:0042578 phosphoric ester hyd... 0.059627 4 / 1369
GO:0016812 hydrolase activity, ... 0.018045 1 / 17
GO:0016818 hydrolase activity, ... 0.418915 4 / 3074
GO:0016421 CoA carboxylase acti... 0.000585 2 / 33
GO:0004075 biotin carboxylase a... 0.000135 2 / 16
GO:0015399 primary active trans... 0.083808 3 / 958
GO:0051020 GTPase binding 0.002871 3 / 262
GO:0046872 metal ion binding 0.270255 14 / 10876
GO:0008081 phosphoric diester h... 0.022094 2 / 213
GO:0017168 5−oxoprolinase (ATP−... 0.002140 1 / 2
GO:0016462 pyrophosphatase acti... 0.409221 4 / 3034
GO:0004485 methylcrotonoyl−CoA ... 1.69e−05 2 / 5
GO:0015405 P−P−bond−hydrolysis−... 0.083007 3 / 954
GO:0005085 guanyl−nucleotide ex... 0.005441 2 / 102
GO:0031267 small GTPase binding 0.187829 1 / 194
GO:0046914 transition metal ion... 0.608626 6 / 5882
GO:0008889 glycerophosphodieste... 0.002254 2 / 65
GO:0009678 hydrogen−translocati... 0.000658 2 / 35
GO:0005086 ARF guanyl−nucleotid... 0.000258 2 / 22
GO:0017016 Ras GTPase binding 0.187829 1 / 194
GO:0050897 cobalt ion binding 0.001513 3 / 209
GO:0017048 Rho GTPase binding 0.061259 1 / 59
GO:0017049 GTP−Rho binding 0.002140 1 / 2

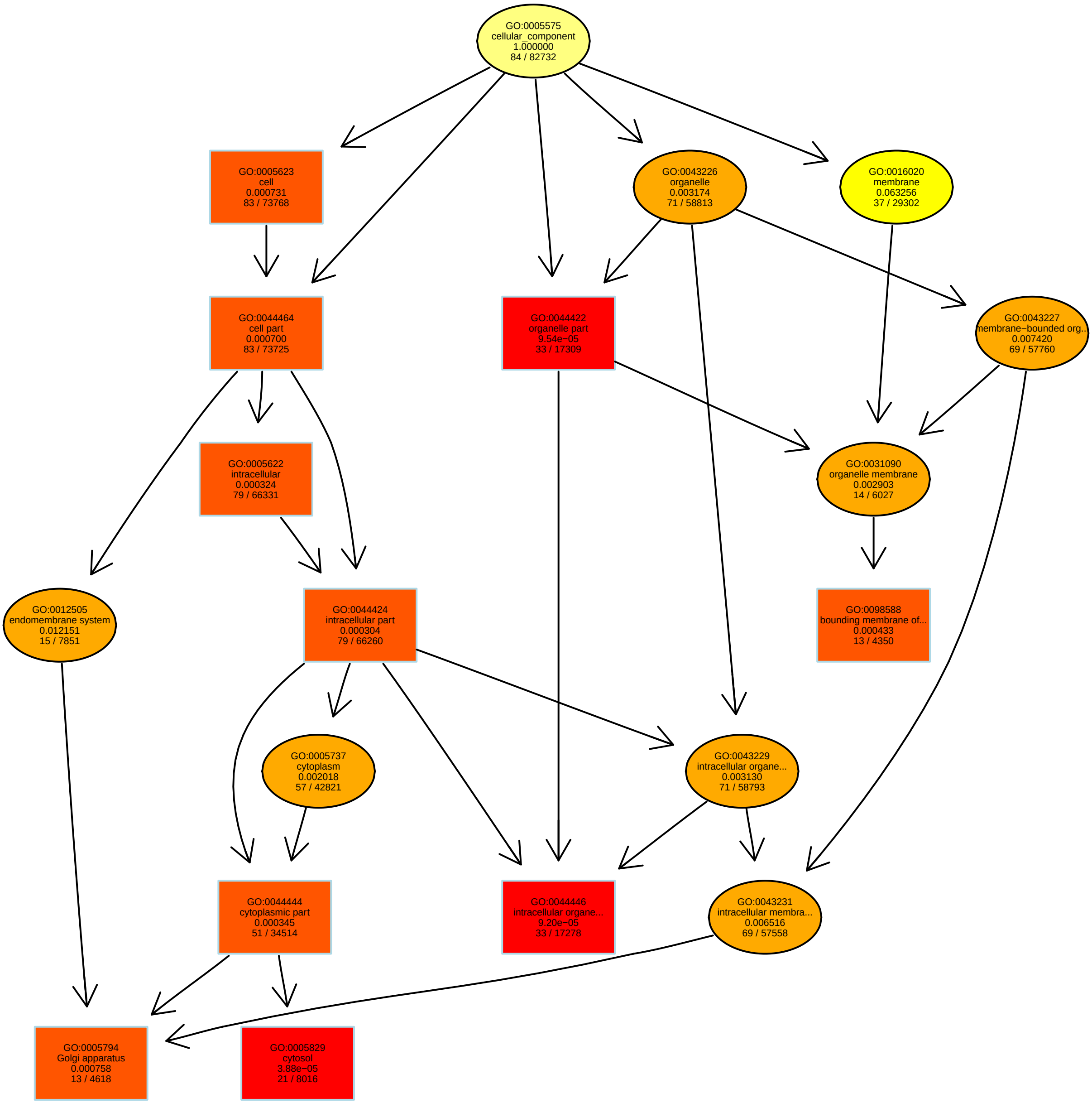
GO:0005575
cellular_component
1.000000
84 / 82732
GO:0005623
cell
0.000731
83 / 73768
GO:0043226
organelle
0.003174
71 / 58813
GO:0016020
membrane
0.063256
37 / 29302
GO:0044464
cell part
0.000700
83 / 73725
GO:0044422
organelle part
9.54e−05
33 / 17309
GO:0043227
membrane−bounded org...
0.007420
69 / 57760
GO:0005622
intracellular
0.000324
79 / 66331
GO:0031090
organelle membrane
0.002903
14 / 6027
GO:0012505
endomembrane system
0.012151
15 / 7851
GO:0044424
intracellular part
0.000304
79 / 66260
GO:0098588
bounding membrane of...
0.000433
13 / 4350
GO:0005737
cytoplasm
0.002018
57 / 42821
GO:0043229
intracellular organe...
0.003130
71 / 58793
GO:0044444
cytoplasmic part
0.000345
51 / 34514
GO:0044446
intracellular organe...
9.20e−05
33 / 17278
GO:0043231
intracellular membra...
0.006516
69 / 57558
GO:0005794
Golgi apparatus
0.000758
13 / 4618
GO:0005829
cytosol
3.88e−05
21 / 8016
