# Supplementary material for: Differential Alternative Splicing Genes and Isoform Regulation Networks of Rapeseed (Brassica napus L.) Infected with Sclerotinia sclerotiorum
Source: Genes (Basel). 2020 Jul 13;11(7):784. doi: 10.3390/genes11070784 (PMC7397149; doi:10.3390/genes11070784)
Supplement: Supplementary file 1 [file genes-11-00784-s001.zip › Fig. S2 Enriched GO terms of genes in the 85 WGCNA modules.zip!/Fig. S2/CytoNodeyellowgreen-go.pdf]

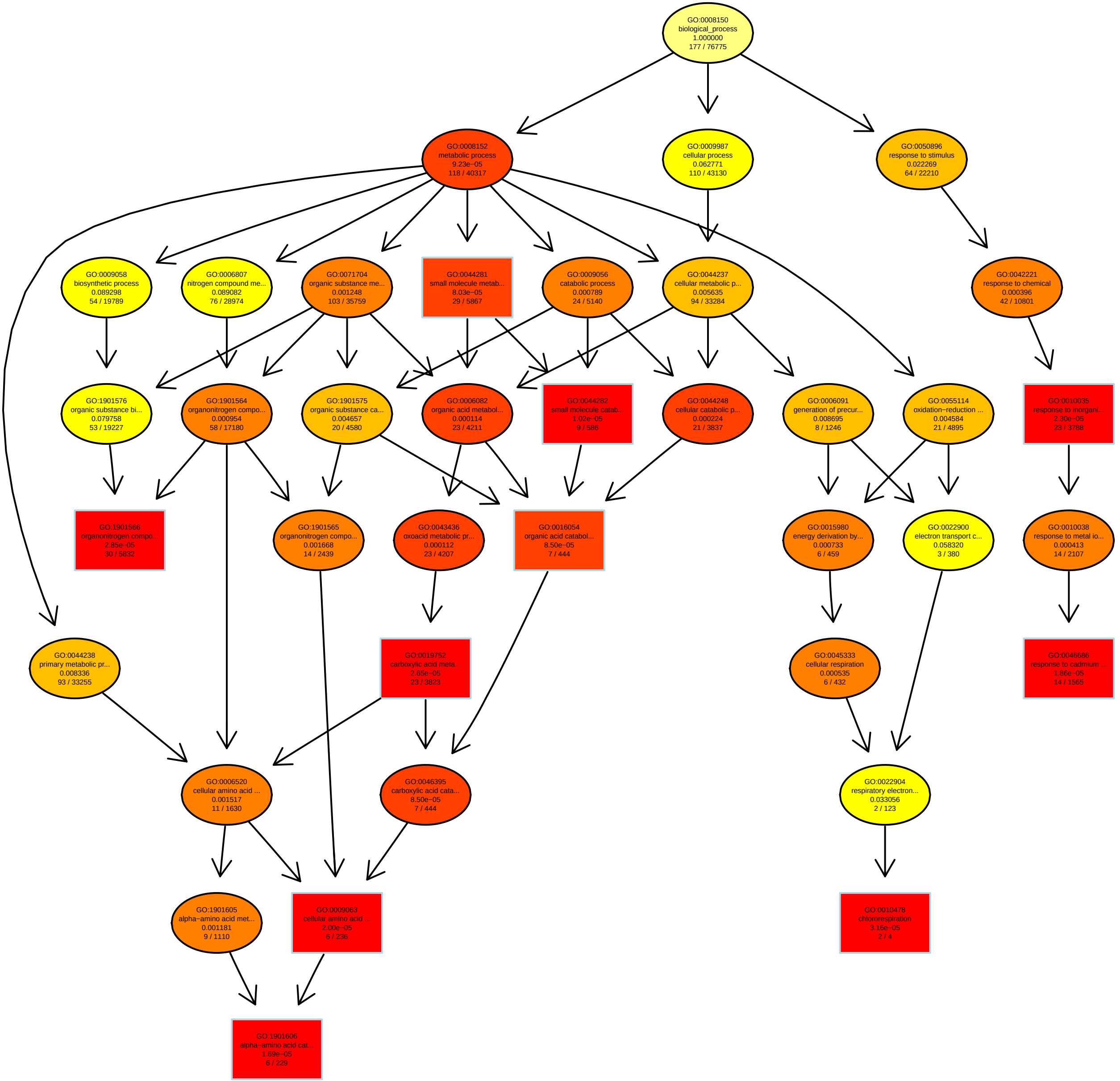
GO:0008150
biological_process
1.000000
177 / 76775
GO:0008152
metabolic process
9.23e−05
118 / 40317
GO:0009987
cellular process
0.062771
110 / 43130
GO:0050896
response to stimulus
0.022269
64 / 22210
GO:0009058
biosynthetic process
0.089298
54 / 19789
GO:0006807
nitrogen compound me...
0.089082
76 / 28974
GO:0071704
organic substance me...
0.001248
103 / 35759
GO:0044281
small molecule metab...
8.03e−05
29 / 5867
GO:0009056
catabolic process
0.000789
24 / 5140
GO:0044237
cellular metabolic p...
0.005635
94 / 33284
GO:0042221
response to chemical
0.000396
42 / 10801
GO:1901576
organic substance bi...
0.079758
53 / 19227
GO:1901564
organonitrogen compo...
0.000954
58 / 17180
GO:1901575
organic substance ca...
0.004657
20 / 4580
GO:0006082
organic acid metabol...
0.000114
23 / 4211
GO:0044282
small molecule catab...
1.02e−05
9 / 586
GO:0044248
cellular catabolic p...
0.000224
21 / 3837
GO:0006091
generation of precur...
0.008695
8 / 1246
GO:0055114
oxidation−reduction ...
0.004584
21 / 4895
GO:0010035
response to inorgani...
2.30e−05
23 / 3788
GO:1901566
organonitrogen compo...
2.85e−05
30 / 5832
GO:1901565
organonitrogen compo...
0.001668
14 / 2439
GO:0043436
oxoacid metabolic pr...
0.000112
23 / 4207
GO:0016054
organic acid catabol...
8.50e−05
7 / 444
GO:0015980
energy derivation by...
0.000733
6 / 459
GO:0022900
electron transport c...
0.058320
3 / 380
GO:0010038
response to metal io...
0.000413
14 / 2107
GO:0044238
primary metabolic pr...
0.008336
93 / 33255
GO:0019752
carboxylic acid meta...
2.65e−05
23 / 3823
GO:0045333
cellular respiration
0.000535
6 / 432
GO:0046686
response to cadmium ...
1.86e−05
14 / 1565
GO:0006520
cellular amino acid ...
0.001517
11 / 1630
GO:0046395
carboxylic acid cata...
8.50e−05
7 / 444
GO:0022904
respiratory electron...
0.033056
2 / 123
GO:1901605
alpha−amino acid met...
0.001181
9 / 1110
GO:0009063
cellular amino acid ...
2.00e−05
6 / 236
GO:0010478
chlororespiration
3.16e−05
2 / 4
GO:1901606
alpha−amino acid cat...
1.89e−05
6 / 229

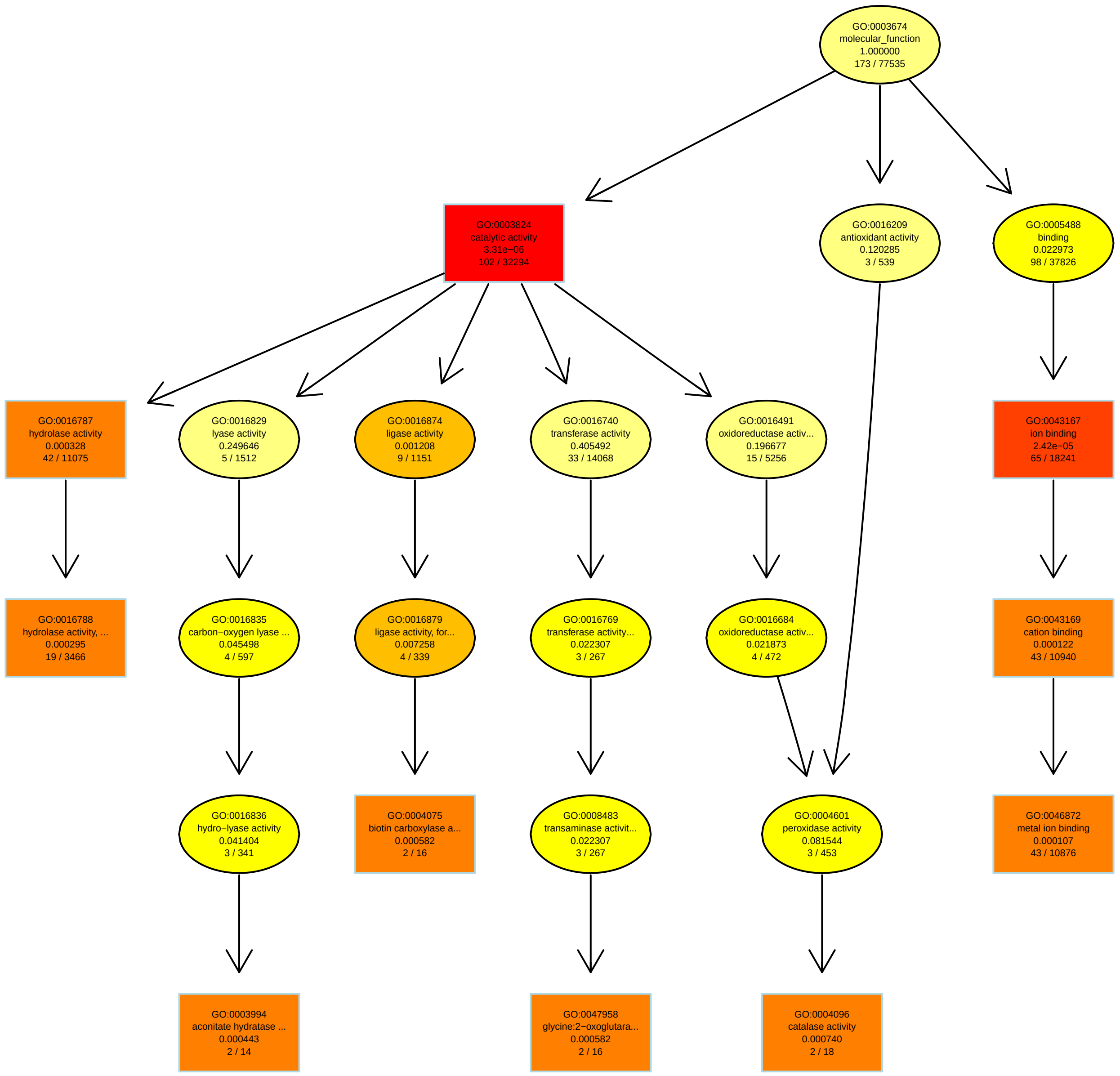

GO:0003674
molecular_function
1.000000
173 / 77535
GO:0003824
catalytic activity
3.31e−06
102 / 32294
GO:0016209
antioxidant activity
0.120285
3 / 539
GO:0005488
binding
0.022973
98 / 37826
GO:0016787
hydrolase activity
0.000328
42 / 11075
GO:0016829
lyase activity
0.249646
5 / 1512
GO:0016874
ligase activity
0.001208
9 / 1151
GO:0016740
transferase activity
0.405492
33 / 14068
GO:0016491
oxidoreductase activ...
0.196677
15 / 5256
GO:0043167
ion binding
2.42e−05
65 / 18241
GO:0016788
hydrolase activity, ...
0.000295
19 / 3466
GO:0016835
carbon−oxygen lyase ...
0.045498
4 / 597
GO:0016879
ligase activity, for...
0.007258
4 / 339
GO:0016769
transferase activity...
0.022307
3 / 267
GO:0016684
oxidoreductase activ...
0.021873
4 / 472
GO:0043169
cation binding
0.000122
43 / 10940
GO:0016836
hydro−lyase activity
0.041404
3 / 341
GO:0004075
biotin carboxylase a...
0.000582
2 / 16
GO:0008483
transaminase activit...
0.022307
3 / 267
GO:0004601
peroxidase activity
0.081544
3 / 453
GO:0046872
metal ion binding
0.000107
43 / 10876
GO:0003994
aconitate hydratase ...
0.000443
2 / 14
GO:0047958
glycine:2−oxoglutara...
0.000582
2 / 16
GO:0004096
catalase activity
0.000740
2 / 18

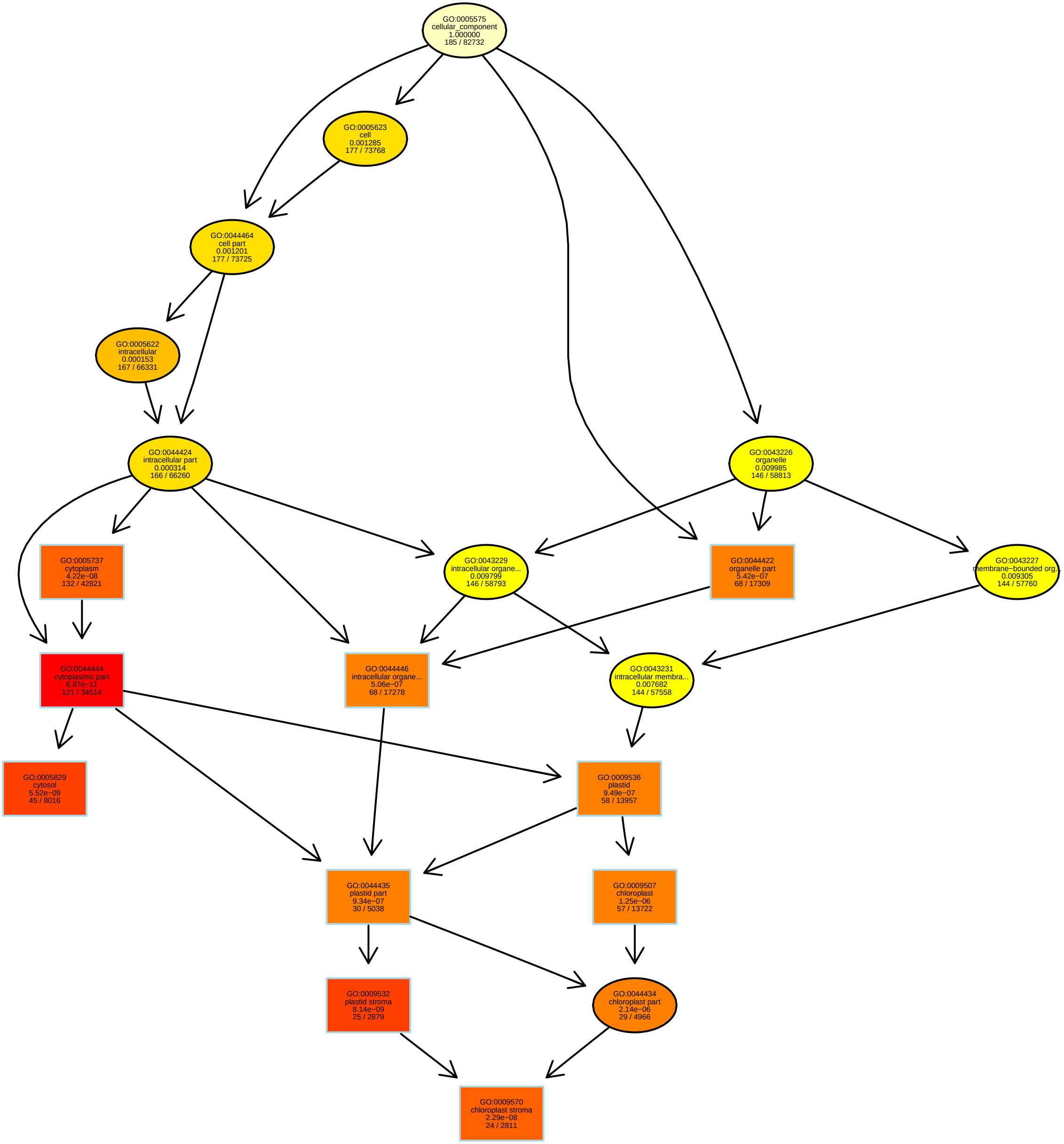
GO:0005575
cellular_component
1.000000
185 / 82732
GO:0005623
cell
0.001285
177 / 73768
GO:0044464
cell part
0.001201
177 / 73725
GO:0005622
intracellular
0.000153
167 / 66331
GO:0044424
intracellular part
0.000314
166 / 66260
GO:0043226
organelle
0.009985
146 / 58813
GO:0005737
cytoplasm
4.22e−08
132 / 42821
GO:0043229
intracellular organe...
0.009799
146 / 58793
GO:0044422
organelle part
5.42e−07
68 / 17309
GO:0043227
membrane−bounded org...
0.009305
144 / 57760
GO:0044444
cytoplasmic part
6.87e−11
121 / 34514
GO:0044446
intracellular organe...
5.06e−07
68 / 17278
GO:0043231
intracellular membra...
0.007682
144 / 57558
GO:0005829
cytosol
5.52e−09
45 / 8016
GO:0009536
plastid
9.49e−07
58 / 13957
GO:0044435
plastid part
9.34e−07
30 / 5038
GO:0009507
chloroplast
1.25e−06
57 / 13722
GO:0009532
plastid stroma
8.14e−09
25 / 2879
GO:0044434
chloroplast part
2.14e−06
29 / 4966
GO:0009570
chloroplast stroma
2.29e−08
24 / 2811
